# Holophytochrome-Interacting Proteins in *Physcomitrella*: Putative Actors in Phytochrome Cytoplasmic Signaling

**DOI:** 10.3389/fpls.2016.00613

**Published:** 2016-05-12

**Authors:** Anna Lena Ermert, Katharina Mailliet, Jon Hughes

**Affiliations:** Institute for Plant Physiology, Justus Liebig UniversityGiessen, Germany

**Keywords:** phytochrome, signal transduction, phototropin, *Physcomitrella patens*, yeast two-hybrid, split-YFP, bimolecular fluorescence complementation, protein–protein interaction

## Abstract

Phytochromes are the principle photoreceptors in light-regulated plant development, primarily acting via translocation of the light-activated photoreceptor into the nucleus and subsequent gene regulation. However, several independent lines of evidence indicate unambiguously that an additional cytoplasmic signaling mechanism must exist. Directional responses in filament tip cells of the moss *Physcomitrella patens* are steered by phy4 which has been shown to interact physically with the blue light receptor phototropin at the plasma membrane. This complex might perceive and transduce vectorial information leading to cytoskeleton reorganization and finally a directional growth response. We developed yeast two-hybrid procedures using photochemically functional, full-length phy4 as bait in *Physcomitrella* cDNA library screens and growth assays under different light conditions, revealing Pfr-dependent interactions possibly associated with phytochrome cytoplasmic signaling. Candidate proteins were then expressed *in planta* with fluorescent protein tags to determine their intracellular localization in darkness and red light. Of 14 candidates, 12 were confirmed to interact with phy4 *in planta* using bimolecular fluorescence complementation. We also used database information to study their expression patterns relative to those of phy4. We discuss the likely functional characteristics of these holophytochrome-interacting proteins (HIP’s) and their possible roles in signaling.

## Introduction

Plant phytochromes absorb predominantly red (R; ∼660 nm) and far-red (FR; ∼710–730 nm) light, thereby steering physiological responses including seed germination, de-etiolation, shade avoidance, and flowering. The R-absorbing Pr dark state is restricted to the cytoplasm and is physiologically inactive. In light it is converted to the physiologically active FR-absorbing Pfr state, which is then translocated from the cytoplasm to the nucleus where it regulates the transcription of numerous genes via direct interaction with master transcription factors such as the PIF’s, leading to the long-term responses mentioned above. However, several physiological responses cannot be explained by gene regulation. Firstly, responses like R/FR-dependent ion fluxes (Tanada, 1968) or cytoplasmic streaming (Takagi et al., 2003) occur within seconds, far too fast for such a mechanism. Secondly, the directional responses in higher plants mediated by the blue light (B) absorbing photoreceptor phototropin are modulated by phytochromes: although this might derive from phytochrome effects on gene expression, several of these responses are still seen in mutants in which nuclear translocation is defective, implying that Pfr is also physiologically active in the cytoplasm (Rösler et al., 2007; Kami et al., 2012). Indeed, *Arabidopsis* phyA and phyB Pfr bind and phosphorylate PKS1, a cytoplasmic protein, whereas phyB regulates translation of PORA by Pfr-dependent binding to PENTA1, also a cytoplasmic protein (Fankhauser et al., 1999; Paik et al., 2012). The fact that phytochromes can connect to signal transduction systems independently of transcription/translation is even more obvious in bryophytes. Phototropic growth of moss filament tip cells is steered by R in a FR-reversible manner, indicating that the photoreceptor is phytochrome. This cannot result from transcription/translation regulation because directional information would inevitably be lost in the process. Furthermore, gene targeting in *Physcomitrella* and *Ceratodon* showed that the response is steered by a specific phytochrome, phy4 (Mittmann et al., 2004, 2009). Thus phy4 must be associated with a signaling mechanism which faithfully transmits the vectorial information given by the incident light. Indeed, we found that phy4 interacts with phototropin at the plasma membrane (Jaedicke et al., 2012), suggesting a physical link to the machinery controlling cytoskeletal organization and tip growth (Meske and Hartmann, 1995; Meske et al., 1996). Chloroplast translocation too is regulated vectorially by phy4 (Kadota et al., 2000; Mittmann et al., 2004). Interestingly, in both the fern *Adiantum* and the alga *Mougeotia* chloroplast movements are also steered vectorially in a R/FR-reversible manner, indicative of phytochrome action – but in these cases via neochrome, a chimeric photoreceptor comprising a phytochrome sensory module attached to a phototropin. Remarkably, transgenic neochrome in *Arabidopsis* rescues phototropism in phototropin-deficient mutants – but the response is then seen in both B and R (Nozue et al., 1998; Kanegae et al., 2006; Kanegae and Kimura, 2015). The field of phytochrome cytoplasmic signaling has been reviewed recently (Hughes, 2013).

It remains unclear, however, how phytochrome cytoplasmic signals are transmitted. Even assuming that the phytochrome simply hijacks the phototropin-associated machinery is futile as little is known about phototropin signaling beyond NPH3 (Roberts et al., 2011). We therefore decided to search for *Physcomitrella* phy4 partners directly, describing our initial results here. We developed Y2H procedures using full-length, photochemically functional, R-activated phy4 holophytochrome as bait in cDNA library screens: to our knowledge this is the first report of such a procedure. The apparent interaction was checked with the full-length CDS model and R/FR reversibility tested. Candidate interactors were then investigated *in planta* regarding their intracellular localization in darkness (D) and R by fusing fluorescent tags to the N- and C-termini of the respective gene products. Finally *in planta* interaction with phy4 was investigated using bimolecular fluorescence complementation (split-YFP) methods. We thereby identified 14 putative holophytochrome interacting proteins (HIP’s), 12 of which were confirmed in split-YFP. Their possible roles in phytochrome cytoplasmic signaling are discussed.

## Materials and Methods

### Cloning and Y2H Procedures

The MatchMaker GAL4 Two-Hybrid System 3 (Clontech) was used to screen for potential cytoplasmic signaling partners of *Physcomitrella* phy4 (Pp3c27_7830V1.1). Full-length phy4 bait was cloned from first strand *Physcomitrella* cDNA into pGBKT7 and pBAC, a derivate of pBRIDGE (both Clontech), to yield hybrid proteins with the BD attached to the N- and C-termini (BD:phy4 and phy4:BD), respectively. A pre-transformed oligo dT-primed cDNA library in the prey vector pGADT7 was kindly provided by Hans Sommer (MPI Cologne) in yeast strain AH109. Single, double and screening transformations were performed as described (Agatep et al., 1998; Gietz and Woods, 2002). The library was either transformed with 40 μg BD:phy4 in a 40x scale or mated to Y187 yeast pre-transformed with phy4:BD. For mating, the bait strain was grown overnight, harvested and mixed in a in a 2:1 ratio with cells of the thawn library aliquot in 2x YPDA medium and incubated for 24 h at 40 rpm and 30°C. Transformation and mating mixtures were plated either with water or 0.5x YPDA on -Trp/-Leu/-His TSD medium with 2.5 mM 3-AT or with 0.25 mM 3-AT and 30 μM PCB, respectively. PCB was extracted from *Spirulina* and purified as described (Jaedicke et al., 2012). Plates were incubated for 14 days at 30°C in D or in 0.7 μmol m^-2^ s^-1^ Rc (660 nm LEDs). Fresh PCB was added 2–3 times during the incubation period. Positives were picked and transferred to a -Trp/-Leu DSD (double SD) masterplate and also further selected on TSD with 3-AT and on -Trp/-Leu/-His/-Ade QSD (quadruple SD) in the case of double transformation and TSD with PCB in case of library mating. DNA extracts were transformed into *E. coli*, the cloned pGADT7 DNA extracted, the inserts sequenced and finally identified by BLAST searches at NCBI^1^ and Cosmoss^2^. Based on EST and our own sequencing data, the likely best gene model was selected and the corresponding amino acid sequences were BLASTed against *Arabidopsis thaliana* and *Viridiplantae* non-redundant protein databases at NCBI in order to identify putative homologs in higher plants.

AD:HIP4 and AD:HIP6 constructs were cloned by Phusion amplification of cDNA, subcloning into pCR bluntII TOPO (Invitrogen) and ClaI/SacI cloning into pGADT7. All other constructs of candidate phy4-interacting partners were obtained by Gateway cloning. Full length entry clones were generated by Phusion amplification of cDNA in a 2-step PCR whereby attB1 and attB2 sites were attached to the product. For N-terminal and C-terminal tags, the CDS was amplified with and without stop codons, respectively. Gel-purified PCR products were cloned into pDONR207 with BP-clonase (Invitrogen). PRL1 and PLP entry clones were created by TA-cloning of PCR products into pCR8GW TOPO (Invitrogen). The resulting full length entry clones were used to create expression constructs for Y2H assays and cytological analyses via the LR reaction. Inserts were recombined into AD: and :AD prey vectors pGADT7g (Uetz and Grigoriev, 2005) and pGADCg (Stellberger et al., 2010), respectively, and transformed into yeast together with BD:phy4 and phy4:BD holophytochrome baits for semi-quantitative growth assays in order to verify the interaction and its potential light-dependency. For this, 2 × 10^5^ doubly transformed yeast cells were spotted on TSD medium with 3-AT and 30 μM PCB and incubated for 5 days in continuous 0.7 μmol m^-2^ s^-1^ (Rc) or 12 min/h red light (660 nm LEDs, 4 μmol m^-2^ s^-1^) pulses (Rp) or red followed by 12 min/h far-red (740 nm LEDs, 5 μmol m^-2^ s^-1^) pulses (Rp+FRp) or darkness (D). Various controls were included to ensure that appropriate interactions were being monitored (see also Jaedicke et al., 2012). Constitutive dimerization was demonstrated by BD:phy4 and phy4:BD baits combined with the AD:phy4 prey. As negative controls and for the adjustment of appropriate 3-AT concentrations, BD:phy4 and phy4:BD baits were combined with the empty pGADT7 vector (from this 2.5 mM or 1 mM 3-AT were used for doubly transformed yeast growth assays). As a control for successful holophytochrome formation, BD:phyA (in pGBKT7) and phyA:BD (in pBAC) baits were combined with AD:FHY1 (1 mM 3-AT) as this Y2H interaction is Pfr-dependent (Hiltbrunner et al., 2005).

For intracellular localization studies, each partner CDS was Gateway-cloned into modified destination vectors p2CGW7-2 × 35S, p2GWC7-2 × 35S, p2FGW7-2 × 35S, and p2GWF7-2 × 35S (Karimi et al., 2002), creating N- or C-terminal CFP- or GFP-fusions, respectively. For *in planta* interaction studies, the CDS was recombined into pSAT4-DEST-n(1-174)eYFP-CI(YFP_N_:) and pSAT4(A)-DEST-n(1-174)eYFP-N1(:YFP_N_) or in pSAT5-DEST-C(175-end)eYFP-CI (YFP_C_:) and pSAT5(A)-DEST-c(175-end)EYFP-N1 (:YFP_C_) destination vectors (Citovsky et al., 2006). phy4 pSAT constructs were created as described (Jaedicke et al., 2012). Negative controls for interaction were provided by empty YFP_C_ and YFP_N_ vectors in combination with the HIP pSAT expression plasmids.

### *In Planta* Localization and Interaction Studies

Biolistic transfection of *Physcomitrella* filaments was performed as described (Jaedicke et al., 2012). For subcellular protein localization studies, transfected filaments were incubated for 2 days in D and observed via fluorescence microscopy without pre-treatment and following 1 h R pre-treatment (660 nm LEDs, 3 μmol m^-2^ s^-1^). For split-YFP studies, plant material was incubated for 3 days in D and then subjected to confocal microscopy with and without analogous pre-treatments.

Transformed cells were identified by R fluorescence of the co-transfected nuclear marker mCherry:VirD2NLS using a Leica Z16 apo fluorescence macroscope. For localization and split-YFP studies, an automated DM6000b fluorescence microscope and a TCS SP2 AOBS confocal laser scanning microscope (both Leica) were used, respectively. For filter cube and look-up table details, see Jaedicke et al. (2012). Single scans of YFP fluorescence were recorded with 4x line and 6x frame average. In order to distinguish YFP signals from possible mCherry bleed-through, YFP emission detection was narrowed to 525–535 nm. The red mCherry- and chlorophyll autofluorescence and the transmission images were taken with 4x line and 2x frame average. Adobe Photoshop CS5 was used for image processing (enhancement of brightness and contrast).

### *In Silico* Analyses

The protein sequences derived from the relevant HIP gene models were further analyzed to identify domains and thus potential functions. Co- and possible synexpression with phy4 was investigated using data from eFP, Phytozome and Genevestigator with the help of ClustVis^3^^,^^4^^,^^5^^,^^6^ .

## Results and Discussion

### Y2H Library Screens Revealed 14 Putative HIP’s

We developed Y2H methods to screen for phy4-interacting proteins that might be involved in the phy4 cytoplasmic signaling pathway. Importantly, instead of photochemically impotent fragments, we established the use of functional, full-length holophytochrome as bait by feeding the transgenic yeast cells with PCB to allow auto assembly *in vivo*, as evidenced by R/FR reversible interaction of *Arabidopsis* phyA with FHY1 (**Supplementary Figure S1**) and, subsequently, of phy4 with various putative partners. We generated bait constructs with the BD N- and C-terminally fused to full-length phy4, carefully characterizing their homodimerization and autoactivation properties. The N-terminal fusion thereby showed a higher autoactivation of the *HIS3*-reporter than the C-terminally fused bait. Accordingly, 3-AT (a competitive inhibitor of the *HIS3* gene product) was included at 2.5 and 1 mM, respectively. Both phy4 baits showed constitutive homodimerization together with the phy4 prey fused N-terminally to the AD (**Supplementary Figure S1**). We screened the pre-transformed cDNA library with both apo-BD:phy4 and holo-phy4:BD in Rc via sequential double transformation and mating, respectively. Functionality of the mating protocol for holophytochrome formation was confirmed by mating Y187 phyA:BD to AH109 AD:FHY1 and selecting on PCB supplemented medium in Rc (**Supplementary Figure S1**). The yeast mating protocol evoked substantially less autoactivation than the transformation protocol, necessitating minimal 3-AT (0.25 mM only for phy4:BD). Therefore we expected also weak or temporary interactions with phy4 Pfr to be identifiable.

The 4.2 × 10^5^ clones were screened with phy4:BD bait, corresponding to about fourfold coverage of the *Physcomitrella* transcriptome. 1 × 10^4^ clones were additionally screened with BD:phy4 bait. The 69 and 75 clones, respectively, survived two rounds of selection and were sequenced, following which 54 and 23 yeast clones excluding duplicates were subjected to BLAST analyses at Cosmoss to identify the corresponding loci in the *Physcomitrella* genome (see **Supplementary Tables S1** and **S2**). Annotation was improved by domain analysis and by BLAST searches of the respective protein sequences against the *Viridiplantae* and *Arabidopsis* non-redundant protein sequence databases. On this basis, genes which were considered unlikely to be involved in signaling were not considered further, leaving candidates putatively involved in signaling processes, posttranslational modification, transcription/translation and likely membrane, transport- or cytoskeleton-associated proteins. We subsequently analyzed 11 and 4 candidates, respectively, by cloning the full-length cDNA into AD: and :AD prey plasmids and combining them in yeast with full-length photoactive BD:phy4 and phy4:BD bait in semiquantitative growth assays under different light conditions (**Supplementary Figure S2**). Only one now failed to interact with BD:phy4 and phy4:BD (data not shown), perhaps because a serendipitous binding site shown by the partial cDNA expression product was lost in the full length configuration. The remaining 14 candidates showed interaction with BD:phy4 and/or phy4:BD full-length holophytochrome bait either constitutively or, more commonly, in a Rp-enhanced (FRp-reversible) manner. These were thus provisionally termed holophytochrome-interacting proteins (HIP1–14).

### Verification and Characterization of HIP’s

The cellular localization of each putative HIP was subsequently studied *in planta* using *Physcomitrella* filaments transfected with appropriate fluorescent protein hybrid constructs in D and after R pre-treatment. As a rule the same cells were observed before and after illumination. Finally, interaction *in planta* between each putative HIP and phy4 was studied using bimolecular fluorescence complementation (in D and after R pre-treatment) with split-YFP hybrid proteins, whereby the cellular localization of the interaction itself was documented. In all cases, hybrids in which the additional domains were fused either to the N- or the C-termini of the putative HIP and phy4 were generated, thereby accounting both for possible functional artifacts arising from the fusion and for topological differences that might affect the interaction readout. Respective split-YFP negative controls are shown in **Supplementary Figure S3**. Twelve out of the 14 putative HIP’s were thereby proven to interact *in planta*. The two putative HIP’s which failed to show an *in planta* split-YFP signal might interact too weakly for YFP reconstitution or represent false positives from Y2H. The first of these, Pp3c19_20830V1.1 (initially named HIP2) represents a likely pirin-related protein (see HIP1 below). Also depending on the configuration, this protein localized to the cytoplasm or to cytoplasm and nucleus, respectively (see Supplemetary Figure S5). The second, Pp3c1_11190C1.1 (Cosmoss Version 3 accession number; initially named HIP10), encodes a protein with an N-terminal ankyrin- and a C-terminal BTB/POZ (Boad-complex, Tamtrac, Bric a brac/POx virus and ZF)-domain (see Supplemetary Figure S4). CFP-fusions were localized either in cytosolic speckles or uniformly in the cytoplasm and nucleus, depending on the FP-configuration. Our results for the 12 HIP’s confirmed *in planta* are described below.

#### HIP1 (Pp3c2_10320V1.1)

**FIGURE 1:**
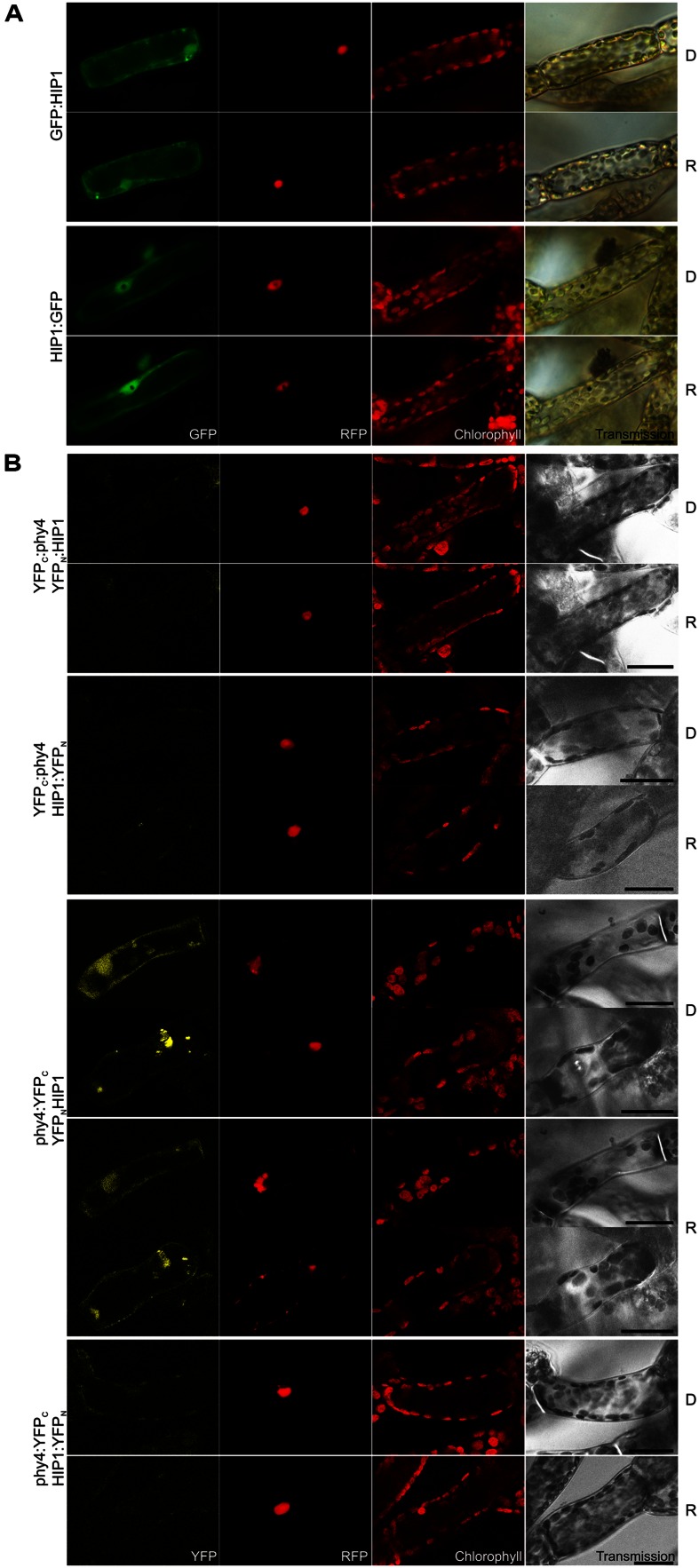
**HIP1 (Pp3c2_10320V1.1) intracellular localization **(A)** and split-YFP-studies of HIP1–phy4 interaction **(B)** each without (D) and with red pre-treatment (R) using fluorescence- and confocal microscopy, respectively.** **(A)** Green fluorescence of N- and C-terminal HIP1:GFP fusions monitored by GFP (column 1) and was visible in nucleus and cytoplasm in both cases; Red fluorescence of the co-transfected mCherry:NLS nuclear marker (RFP) is shown in column 2; Chlorophyll autofluorescence (chlorophyll) and a differential interference contrast image (DIC) is shown in columns 3 and 4, respectively. For each FP configuration cells were photographed before (D) and after red light pre-treatment (R). The apparent “hole” in the nucleus of the HIP1:GFP fluorescence images and the corresponding black dot in the DIC images represents a large gold particle. **(B)** All possible split-YFP configurations were analyzed without (D) and with red pre-treatments (R) via confocal microscopy as shown in column 1: YFP_C_:phy4–YFP_N_:HIP1 (rows 1+2), YFP_C_:phy4–HIP1:YFP_N_ (rows 3 and 4), phy4:YFP_C_–YFP_N_:HIP1 (rows 5–8) and phy4:YFP_C_–HIP1:YFP_N_ (rows 9 and 10)_._ Analogous to panel **(A)**, columns depict (from left to right) YFP fluorescence, RFP fluorescence from mCherry:NLS, chlorophyll autofluorescence and a transmission image. Two different signal patterns were observed for phy4:YFP_C_–YFP_N_:HIP1, thus representative images are shown for each. Other configurations yielded only background signals alongside channel bleed-through. Scale bars 30 μm.

Independent library screens revealed two different members of the **pirin** iron-containing subgroup of the **cupin superfamily**, (HIP1, 38.7 kDa) and Pp3c19_20830V1.1 (initially HIP2, 33.9 kDa). Semiquantitative studies of full length CDS models showed strong and weaker binding to phy4 holophytochrome in Y2H, respectively. Indeed except in the case of HIP1–BD:phy4, the interaction was R/FR reversible (**Supplementary Figure S2**). However, as Pp3c19_20830 showed insignificant split-YFP-signals (**Supplementary Figure S5B**) it was excluded as a genuine HIP. In *Physcomitrella* HIP1 was distributed in the cytoplasm and nucleus (**Figure 1A**). *In planta* interaction was verified for HIP1 in the phy4:YFP_C_–YFP_N_:HIP1 configuration (**Figure 1B**) although the result was ambiguous because the signal was detectable either in cytoplasm and nucleus or in cytosolic speckles and nucleus in different cells both in D and following R pre-treatment.

HIP1 is apparently expressed under all conditions and in all cell types although weekly in leaflets (see Supplementary Materials). The data provide little evidence for synexpression with phy4.

Holophytochrome-interacting protein 1 (like Pp3c19_20830) harbors an N-terminal cupin domain including the potential metal-coordinating residues H116, H118, H160, and E162 corresponding to H57, H59, H101, and E103 in the human sequence (Pang et al., 2004; Adams and Jia, 2005). The pirin-typical C-terminal domain (which resembles the cupin domain while lacking the metal-coordinating residues) is also present in HIP1 (but not Pp3c19_20830). Conserved in mammals, fungi, plants and prokaryotes, four and three genes encoding pirin-like proteins exist in *Arabidopsis* and *Physcomitrella*, respectively (see Summary **Table 1** in the Supplementary Material).

Pirin functions are quite diverse. Human, *Arabidopsis* and *E. coli* pirins have been shown to possess quercetinase activity (Adams and Jia, 2005). Quercetin – amongst other things – plays a role in UV acclimation (Hectors et al., 2014) and has been shown to inhibit polar auxin transport (Fischer et al., 1997; Kuhn et al., 2011; Buer et al., 2013) and thus might play a role in tropic responses (Lewis et al., 2011). Indeed, light-grown *prn1*^-^ *Arabidopsis* seedlings showed disoriented hypocotyl growth, whereas etiolated mutant seedlings showed enhanced UV-induced quercetin levels (Orozco-Nunnelly et al., 2014). In *Arabidopsis* quercetin is localized in and around the nucleus, in the endomembrane system and at the plasma membrane (Peer et al., 2001). Although this would correlate with the phy4–phototropin complex at the plasma membrane (Jaedicke et al., 2012) our data do not imply that HIP1 is localized at the plasma membrane (**Figure 1**). Diverse physiological effects have been associated with pirins in plants, including pathogen susceptibility, apoptosis, ABA-suppression of seed germination and B-induced *LHC* expression, possibly in association with G-proteins (Orzaez et al., 2001; Lapik and Kaufman, 2003; Warpeha et al., 2007; Zhang et al., 2014). Phytochrome signaling via G-proteins is controversial, however (Neuhaus et al., 1993; Okamoto et al., 2001; Jones et al., 2003; Urano et al., 2013). The *Physcomitrella* genome codes for at least one G-protein but this has not been studied to-date.

#### HIP3 (Pp3c10_4820V1.1)

**FIGURE 2:**
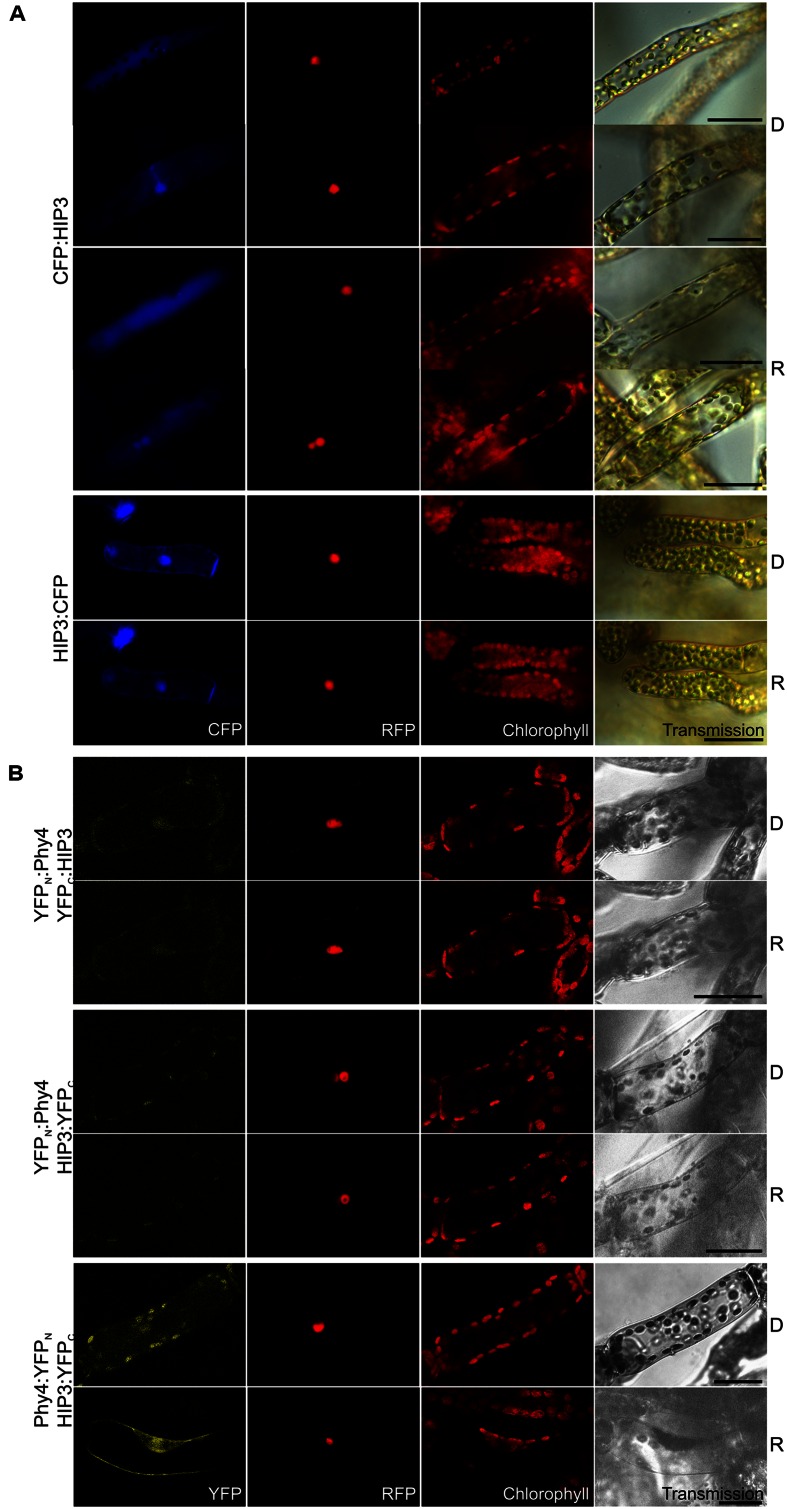
**HIP3 (Pp3c10_4820V1.1) intracellular localization **(A)** and split-YFP-studies of HIP3–phy4 interaction **(B)** each without (D) and with red pre-treatment (R) using fluorescence- and confocal microscopy, respectively.** Overall figure structure is analogous to **Figure 1**. **(A)** CFP:HIP3 (rows 1–4) and HIP3:CFP (rows 5 and 6). CFP:HIP3 localization was ambiguous, being observable either in the nucleus and cytoplasm (rows 2 and 4) or in the cytoplasm only (rows 1 and 3) in both conditions, whereas HIP3:CFP showed nuclear and cytoplasmic localization (column 1, CFP). **(B)** Split-YFP analyses of YFP_N_:phy4–YFP_C_:HIP3 (rows 1 and 2), YFP_N_:phy4–HIP3:YFP_C_ (rows 3 and 4) and phy4:YFP_N_–HIP3:YFP_C_ (rows 5 and 6) configurations. Significant nucleo-cytoplasmic signals were only seen for phy4:YFP_N_–HIP3:YFP_C_ after R pre-treatment (column 1, YFP; D and R here from separate experiments). All other configurations/conditions yielded only background signals alongside channel bleed-through. Scale bars 30 μm.

Holophytochrome-interacting protein 3 (37.4 kDa) carries CHY, CTCHY, and C3HC4 (referring to a specialized cysteine-histidine pattern) **RING-type ZF** domains. Y2H analysis with the full-length CDS showed weak but R-enhanced interaction with phy4 in all conformations. phy4:BD–ADg:HIP3 showed interaction in Rc but not in Rp (**Supplementary Figure S2**). *In planta*, although no particular changes in localization were seen upon irradiation, HIP3 showed cytosolic or nuclear and cytosolic localization in approximately half of the observed cells, respectively (CFP:HIP3) or cytosolic and nuclear (HIP3:CFP) localization, depending on the position of the CFP tag (see **Figure 2A**). Cytosolic localization is interesting because both fusion proteins might be small enough (37 kDa + 27 kDa tag) to enter the nucleus passively, at least as monomers. Conventional NLS and NES motifs are not apparent in the HIP3 sequence. *In planta* interaction was seen for phy4:YFP_N_–HIP3:YFP_C_ in the nucleus and the cytoplasm, consistent with the localization data, the other combinations yielding only background signals alongside channel bleed-through (see **Figure 2B**). Interestingly, the split-YFP signal only appeared after R pre-irradiation, implying Pfr-dependent interaction *in planta* as in Y2H.

Holophytochrome-interacting protein 3 is apparently expressed under all conditions and in all cell types except in dehydrated material (see Supplementary Materials). The data provide little evidence for synexpression with phy4.

Zinc finger-proteins are known to bind diverse ligands including DNA, RNA, lipids, and proteins (Laity et al., 2001; Matthews and Sunde, 2002), thereby executing functions in a variety of cellular processes such as transcription, translation, cytoskeleton organization, protein folding, and others. The CHY-ZF is named due to its conserved motif CxHY, the function of which is still unknown (Leng et al., 2003). The 40–60 residues comprising RING-type ZF’s, however, are considered to bind two zinc atoms and to mediate protein–protein interactions (Freemont, 1993; Borden and Freemont, 1996; Saurin et al., 1996). RING-type ZFs can also serve as E3-ubiquitin ligases in protein degradation and are thus commonly involved in gene regulation via repressor destruction in plants. BLAST searches indeed revealed high similarity between HIP3 and 4–6 other *Physcomitrella* and at least seven *Arabidopsis* proteins. For example, *Arabidopsis* RING FINGER AND CHY ZINC FINGER DOMAIN-CONTAINING PROTEIN 1 (AT5G22920) shows 63% identity in the 250 residue region of similarity. This nuclear-localized E3 ubiquitin ligase is involved in the regulation of stomatal opening, its activity being regulated via phosphorylation by SnRK2.6 (Ding et al., 2015). CHY and CTCHY and RING-type ZF protein (AT5G25560), a PGPD 14-like protein (Kosarev et al., 2002) of unknown function, shows 57% identity except at the N-terminus. Similarly, MYB30-INTERACTING E3 LIGASE 1 (AT5G18650) with 59% identity also except in the 64 residue N-terminal region) degrades the MYB30 transcription factor, a negative regulator of defense responses in *Arabidopsis* (Marino et al., 2013). Several known *Arabidopsis* phytochrome-interacting and/or signaling molecules are known to possess RING-ZF domains, for example RFI2, which plays a role in phytochrome-controlled seedling de-etiolation (Chen and Ni, 2006) or VOZ1, a phyB partner controlling flowering (Yasui et al., 2012). The central regulator of photomorphogenesis, the E3 ubiquitin ligase COP1 also contains a C3HC4-type RING-ZF motif. Moreover, the PENTA protein involved in translational regulation of PORA (the key light-regulated enzyme in chlorophyll biosynthesis) by interaction with phyB is also a C3H-type ZF protein (Paik et al., 2012).

#### HIP4/PRL1 (Pp3c16_8560E1.1)

**FIGURE 3:**
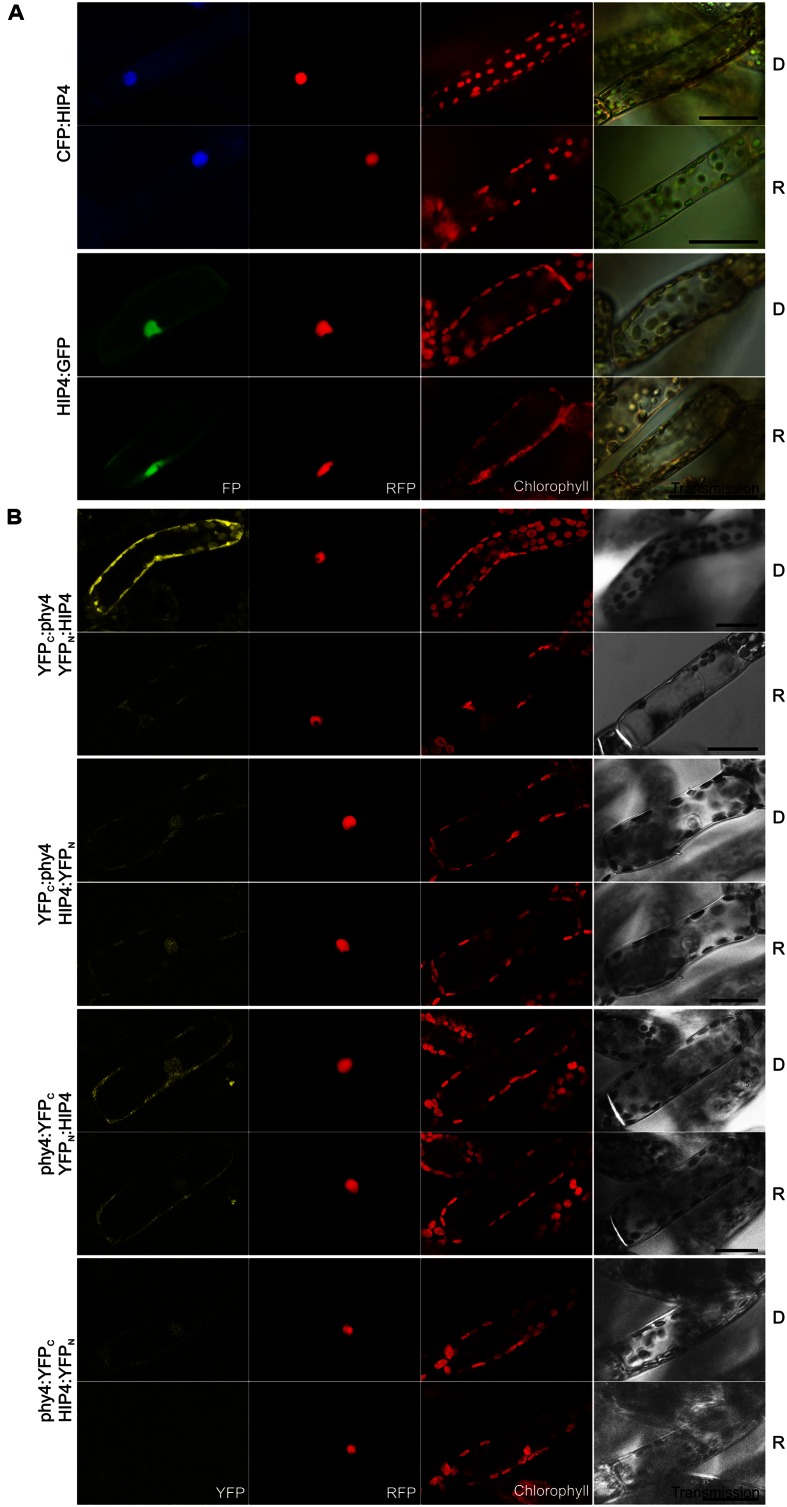
**HIP4 (Pp3c16_8560E1.1) intracellular localization **(A)** and split-YFP-studies of HIP4–phy4 interaction **(B)** each without (D) and with red pre-treatment (R) using fluorescence- and confocal microscopy, respectively.** Overall figure structure is analogous to **Figure 1**. **(A)** CFP:HIP4 (rows 1 and 2) was mostly nuclear and HIP4:GFP (rows 3 and 4) was distributed in nucleus and cytoplasm (column 1, FP). **(B)** All possible split-YFP configurations were analyzed in D and R, respectively: YFP_C_:phy4–YFP_N_:HIP4 (rows 1 and 2), YFP_C_:phy4–HIP4:YFP_N_ (rows 3 and 4), phy4:YFP_C_–YFP_N_:HIP4 (rows 5 and 6) and phy4:YFP_C_–HIP4:YFP_N_ (rows 7 and 8). Significant signals were observed for YFP_C_:phy4–YFP_N_:HIP4 (cytoplasmic signal), phy4:YFP_C_–YFP_N_:HIP4 (nucleo-cytoplasmic signal), and weakly for YFP_C_:phy4–HIP4:YFP_N_ (maybe nucleo-cytoplasmic signal) in both D and R (column 1, YFP). phy4:YFP_C_–HIP4:YFP_N_ yielded only background signals alongside channel bleed-through. Scale bars 30 μm.

Holophytochrome-interacting protein 4 (53.9 kDa) is encoded by ***PRL1*** sharing 62% sequence identity with its homolog in *Arabidopsis.* In Y2H studies full length HIP4/PRL1 interacted with holo phy4 in all four bait–prey configurations. Three interactions were R-enhanced and partially FR-reversible whereas BD:phy4–HIP4:ADg interacted constitutively (**Supplementary Figure S2**). Independently of the light treatment, HIP4:GFP localized to both nucleus and cytoplasm while CFP:HIP4 was concentrated in the nucleus (**Figure 3A**). Constitutive interaction was confirmed *in planta* in all combinations except phy4:YFP_C_–HIP4:YFP_N_ (**Figure 3B**), the reconstituted split-YFP signal being apparent in both nucleus and cytoplasm for phy4:YFP_C_–YFP_N_:HIP4 and, less clearly, YFP_C_:phy4–HIP4:YFP_N_, corresponding to the localization of HIP4:GFP. YFP_C_:phy4–YFP_N_:HIP4 interacted in the cytoplasm.

Holophytochrome-interacting protein 4 is apparently expressed under all conditions and in all cell types (see Supplementary Materials). The Genevestigator data for both treatments and anatomy provide some evidence for synexpression with phy4. This is not apparent for the other data, however.

PLEIOTROPIC REGULATORY LOCUS 1 is well-conserved amongst land plants, fungi, and animals including mammals. Two homologs are present in both *Physcomitrella* and *Arabidopsis* (HIP4/PRL1 & Pp3c5_15520V1.1 and PRL1 & PRL2, respectively). *Arabidopsis* PRL1 is an α-importin-interacting protein localizing both to the nucleus and associating with the nuclear envelope- and ER membranes (Nemeth et al., 1998). It harbors seven WD40 domains and a variant of the SV40-type NLS in the C-terminal region (residues 238–241), which is not constitutively recognized but rather dependent on phosphorylation of the NLS or the importin (Nemeth et al., 1998). Our localization studies of HIP4/PRL1 indicate that at least R does not affect recognition of the NLS and subsequent nuclear import since we do not observe any change in localization upon light treatment (**Figure 3A**). Although N-terminally fused CFP:HIP4 was exclusively nuclear, HIP4:GFP was seen in both the nucleus and cytoplasm, whereas YFP_C_:phy4–YFP_N_:HIP4 was exclusively cytoplasmic (**Figure 3B**). Perhaps phy4 binds the C-terminus of HIP4/PRL1, thereby hampering NLS recognition and effecting cytoplasmic localization.

The PRL1 WD40 domains probably constitute β-propellers, together forming a platform for protein–protein interactions. Such platforms are often involved in signal transduction and transcriptional regulation, as in the case of COP1 in repression of photomorphogenic development. *Arabidopsis* PRL1 seems to be involved in numerous responses, as the name implies. Initially reported to play a role in cell polarity determination by acting on the assembly of the actin cytoskeleton in fission yeast (Xia et al., 1996), PRL1 was later suggested to play a central role in integrating light signals with cytokinin- and sugar response pathways in plants (Nemeth et al., 1998). Indeed *prl1*^-^ hypocotyls are shorter than the wild type in darkness but not in light, representing a weak COP phenotype, consistent with PRL1 acting as a positive regulator of brassinosteroid synthesis (Szekeres et al., 1996; Nemeth et al., 1998). It was shown to promote BR-biosynthesis by interacting with different SnRK’s (see also HIP3 above) in light and dark, possibly by targeting them for degradation (Bhalerao et al., 1999; Lee et al., 2008; Flores-Perez et al., 2010). PRL1 is also involved in root meristem regulation possibly by restricting transcription of the homeobox transcription factor WOX5 to the quiescent center (Ji et al., 2015). Thus PRL1 in *Arabidopsis* might either represent a convergence center for integrating light, sugar, hormone, and other responses or be involved in distinct signaling pathways in different cells. SnRK photoregulation would be a possible function of HIP4/PRL1 in *Physcomitrella*, as SNF1 homologs are present (Thelander et al., 2004, 2007). Light, sugar and the phytohormones cytokinin and auxin are all closely involved in the developmental switches in *Physcomitrella* whereby filament cells develop into chloronemata, caulonemata, or gametophore buds (Thelander et al., 2004, 2007; Decker et al., 2006; von Schwartzenberg, 2006).

#### HIP5 (Pp3c7_3040V1.1)

**FIGURE 4:**
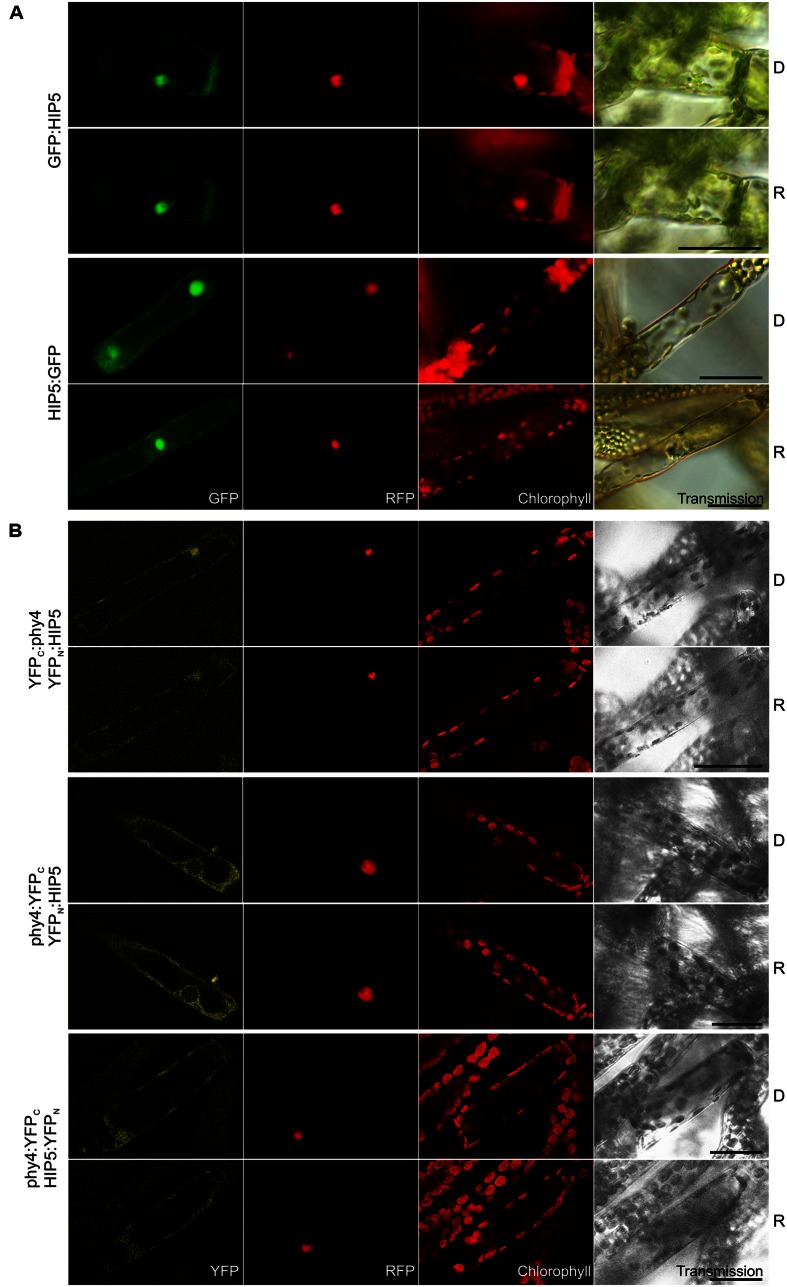
**HIP5 (Pp3c7_3040V1.1) intracellular localization **(A)** and split-YFP-studies of HIP5–phy4 interaction **(B)** each without (D) and with red pre-treatment (R) using fluorescence- and confocal microscopy, respectively.** Overall figure structure is analogous to **Figure 1**. **(A)** GFP:HIP5 (rows 1 and 2) and HIP5:GFP (rows 3 and 4) were distributed in the cytoplasm and in the nucleus (column 1, GFP). **(B)** Three split-YFP configurations were examined and showed different signal patterns/strengths (column 1, YFP): YFP_C_:phy4–YFP_N_:HIP5 (rows 1 and 2; maybe nucleo-cytoplasmic distribution), phy4:YFP_C_–YFP_N_:HIP5 (rows 3 and 4; cytoplasmic distribution) and phy4:YFP_C_–HIP5:YFP_N_ (rows 5 and 6; nucleo-cytoplasmic distribution). The apparent YFP_C_:phy4–YFP_N_:HIP5 signal might also represent RFP bleed-through in the YFP channel since the signal was very weak. Scale bars 30 μm.

Holophytochrome-interacting protein 5 (54.4 kDa) bears four **kelch repeats** toward the C-terminus. It has one homolog in *Physcomitrella* (Pp3c11_19970V1.1, see Summary **Table 1** in the Supplementary Material). The semi-quantitative Y2H interaction assay confirmed R-enhanced and FR-reversible interaction in all phy4–HIP5 configurations tested with the exception of BD:phy4–HIP5:ADg which showed no interaction (**Supplementary Figure S2**). HIP5 seems to be constitutively present in nucleus and cytoplasm irrespective of the position of the fluorescent tag (**Figure 4A**). Interestingly, an older and N-terminally shorter version of the HIP5 gene model showed exclusively cytoplasmic localization as N-terminal GFP-fusion, implying that the putative NLS resides at the very N-terminus (in the first 129 amino acids; not shown). phy4–HIP5 interaction was confirmed *in planta*, but in contrast to the Y2H data, interaction was not affected by light treatments (**Figure 4B**). Analogously to the HIP5 localization data, nuclear and cytoplasmic distribution was seen for phy4:YFP_C_–HIP5:YFP_N_ and perhaps also for YFP_C_:phy4–YFP_N_:HIP5 (although this fluorescence might represent RFP bleed-through in the YFP channel as the signal was very weak). Interestingly, the YFP-signal from the reconstituted phy4:YFP_C_–YFP_N_:HIP5 located exclusively to the cytoplasm, although GFP:HIP5 was apparent in both cytoplasm and nucleus.

Holophytochrome-interacting protein 5 is apparently expressed under all conditions and in all cell types (see Supplementary Materials). The Genevestigator data for treatments, anatomy and development provide some evidence for synexpression with phy4. This is not apparent for the eFP and Phytozome data, however.

The ∼50-residue kelch motif forms a blade-like fold comprising 4–6 anti-parallel β-sheets, often repeated to form a β-propeller structure frequently involved in protein–protein interactions. In fungi, various kelch repeat proteins associate with microtubules and actin nucleating proteins at cell termini, thereby establishing cell polarity (Mata and Nurse, 1997; Feierbach et al., 2004) whereas others are involved in polarized growth (Takeshita et al., 2008). We speculate therefore that HIP5 might play a similar role in phy4-directed vectorial growth. Indeed, amongst both fungal and *Arabidopsis* BLAST hits was a scruin – an actin crosslinker, implying a role in cytoskeleton organization consistent with the role of microfilaments in filament tip cell phototropism (Meske and Hartmann, 1995; Meske et al., 1996). On the other hand, HIP5 shares ∼35% sequence identity with many *Arabidopsis* F-box/kelch-repeat (FBK) proteins such as galactose oxidase/kelch repeat superfamily protein (AT1G14330), MIR2111-5p target protein (AT3G27150) and SKIP11 (AT2G02870), although these sequences rather align with the C-terminal region and thus the kelch repeats of HIP5 (64–71% of the query), whereas the first 135–175 amino acids of HIP5 are dissimilar to the *Arabidopsis* proteins. All three *Arabidopsis* FBK proteins are chloroplast- and/or nuclear-localized, interacting with SKP1-like proteins (Schumann et al., 2011). FBK kelch repeats for example are responsible for binding of target proteins to the SCF ubiquitin ligase complex in the degradation pathway (Cardozo and Pagano, 2004; Hua and Vierstra, 2011). The *Arabidopsis* and *Physcomitrella* genomes contain ca. 100 and 71 FBK genes, respectively (Schumann et al., 2011), several of which are known to be involved in photoresponses. The PAS/LOV-domain-containing ZEITLUPE, FKF1, LKP2 FBK’s are themselves photoreceptors (Sawa et al., 2007; Demarsy and Fankhauser, 2009; Takase et al., 2011), whereas AFR and CFK1 are positive regulators downstream of phyA and phyB, respectively (Wei et al., 1994a,b; Harmon and Kay, 2003; Franciosini et al., 2013). CFK1 seems to be absent from *Physcomitrella* although the COP9 signallosome with which CFK1 interacts is thought to be universal in eukaryotes (Wei et al., 1998), thus HIP5 might be involved in a similar regulatory system. Most FBK’s are nuclear although some are located in both cytoplasm and nucleus – as is the 26S proteasome (Schumann et al., 2011). Pp3c2_13250, Pp3c1_22300, Pp3c10_4990, and Pp3c14_4360, on the other hand, might represent *Arabidopsis* AFR homologs with 29–26% identity covering 72–100% of the query sequence.

#### HIP6 (Pp3c6_4480V1.1)

**FIGURE 5:**
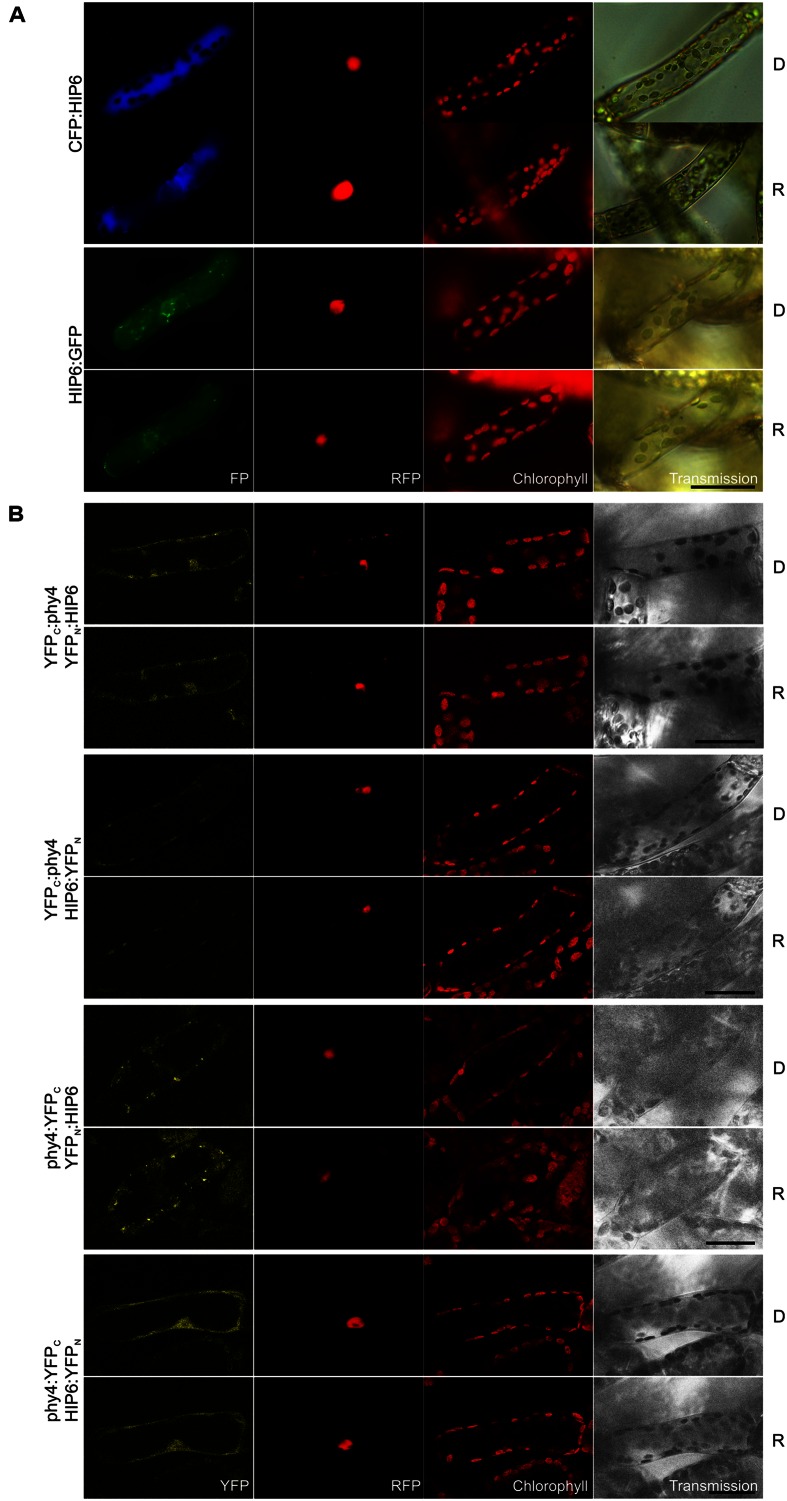
**HIP6 (Pp3c6_4480V1.1) intracellular localization **(A)** and split-YFP-studies of HIP6–phy4 interaction **(B)** each without (D) and with red pre-treatment (R) using fluorescence- and confocal microscopy, respectively.** Overall figure structure is analogous to **Figure 1**. **(A)** Intracellular localizations of CFP:HIP6 (rows 1 and 2) and HIP6:GFP (rows 3 and 4) were both localized in the cytoplasm, with HIP6:GFP showing subcellular foci (column 1, FP). **(B)** All four split-YFP configurations were investigated: YFP_C_:phy4–YFP_N_:HIP6 (rows 1 and 2), YFP_C_:phy4–HIP6:YFP_N_ (rows 3 and 4), phy4:YFP_C_–YFP_N_:HIP6 (rows 5 and 6), and phy4:YFP_C_–HIP6:YFP_N_ (rows 7 and 8). Significant YFP signals were apparent only for phy4:YFP_C_–YFP_N_:HIP6 (cytoplasm and cytoplasmic foci) and phy4:YFP_C_–HIP6:YFP_N_ (cytoplasm and nucleus; column 1, YFP), whereas apparent YFP_C_:phy4–YFP_N_:HIP6 nuclear fluorescence might represent RFP bleed-through and YFP_C_:phy4–HIP6:YFP_N_ only shows chloroplast bleed-through. Scale bars 30 μm.

Holophytochrome-interacting protein 6 (43.7 kDa) harbors a predicted transmembrane domain and a **p-loop containing sulfotransferase** domain at the N- and C-terminus, respectively. Interaction of HIP6 with phy4 in Y2H was constitutive in the BD:phy4–AD:HIP6 and phy4:BD–AD:HIP6 configurations, whereas phy4:BD–HIP6:ADg showed R/FR-reversible interaction (**Supplementary Figure S2**). *In planta* both N- and C-terminal fusion configurations localized exclusively to the cytoplasm, with HIP6:GFP concentrating in discrete regions (cytoplasmic foci; **Figure 5A**). This distribution was also observed for phy4:YFP_C_–YFP_N_:HIP6 interaction, whereas other split-YFP configurations (phy4:YFP_C_–HIP6:YFP_N_ and perhaps YFP_C_:phy4–YFP_N_:HIP6) rather showed cytoplasmic and weaker nuclear localization (**Figure 5B**; although narrower emission detection implied in the latter case that the apparent YFP nuclear signal might have arisen from RFP bleed-through; data not shown). Light pre-treatments had little effect on *in planta* interaction (also in the case of phy4:YFP_C_–HIP6:YFP_N_ corresponding to phy4:BD–HIP6:ADg in Y2H). In neither Y2H nor split-YFP *in planta* was significant interaction seen between N-terminally tagged phy4 and C-terminally tagged HIP6.

Holophytochrome-interacting protein 6 is apparently expressed under all conditions and in all cell types (see Supplementary Materials). The Genevestigator and Phytozome data provide some evidence for synexpression with phy4.

The p-loop domain is widespread in nature, using conserved motifs GXXXXGK(T/S) and ZZZ(D/E) (where X and Z represent arbitrary and hydrophobic residues, respectively) with a β-sheet followed by a glycine-rich loop and an α-helix to co-ordinate the ATP or GTP β-γ-phosphate moiety (Walker et al., 1982; Saraste et al., 1990; Milner-White et al., 1991; Ramakrishnan et al., 2002). Thus, HIP6 belongs to the superfamily of p-loop-containing nucleoside triphosphate hydrolases. This large group is involved in processes like replication, transcription, DNA repair, translation, signal transduction, protein localization, and -transport, signal-sequence recognition, membrane transport, chromosome partitioning, and activation of metabolites (Saraste et al., 1990; Vetter and Wittinghofer, 1999; Koonin et al., 2000). Apart from the common motifs and the p-loop structure itself, the sequences (even the core of the p-loop) and structures are extremely diverse, hampering (phylogenetic and) functional classification (Pathak et al., 2014). So far, only a few plant p-loop NTPases have been functionally characterized. Some are thought to be involved in pathogen defense responses and others in salinity stress tolerance (Fenyk et al., 2012; Cheung et al., 2013).

Plant sulfotransferases (SOT’s) are either soluble or transmembrane proteins that transfer the sulfate group from a 3′-phosphoadenosine-5′-phosphosulfate donor to proteins or smaller substrates including brassinosteroids, jasmonates, flavonoids, or salicylic acid (Klein and Papenbrock, 2004; Hirschmann et al., 2014). Intriguingly, plant SOT’s sulfate the flavonol quercetin (Varin et al., 1992), discussed above in connection with pirins. Quercetin and quercetin sulfates are thought to regulate root development by altering basipetal auxin transport, elongation growth and gravitropism (Faulkner and Rubery, 1992; Lewis et al., 2011). Whereas three HIP6 homologs are apparent in *Physcomitrella* (see Summary **Table 1** in the Supplementary Material), 22 SOT’s with different sulfotransfer-domain-types and diverse expression patterns have been identified in *Arabidopsis*. The hydroxyl-jasmonate sulfating SOT15 is transcriptionally controlled by the circadian clock and PIF4 and -5 (Yamashino et al., 2013) whereas SOT18 seems to be regulated by the phytochrome-controlled transcription factor HY5 (Huseby et al., 2013). Various *Arabidopsis* SOT’s are thought to be functional in such diverse processes as defense and stress responses, regulation of hormone synthesis or activity, signaling and developmental regulation (Rouleau et al., 1999; Gidda et al., 2003; Marsolais et al., 2007; Hirschmann et al., 2014). Mutation of *Arabidopsis* tyrosyl-protein sulfotransferase has pleiotropic defects such as in cotyledon and leaf size and pigmentation, flower number and root development (Komori et al., 2009) the latter being associated with disturbed auxin distribution due to altered PIN and auxin-biosynthesis gene expression (Zhou et al., 2010). The function of HIP6 is therefore unclear, especially as it shares highest similarity to the newly identified *Arabidopsis* SOT’s 19–21 (see Supplementary Materials) whose substrate specificity is unknown (Klein and Papenbrock, 2009; Hirschmann et al., 2014).

#### HIP7 (Pp3c17_9390V1.1)

**FIGURE 6:**
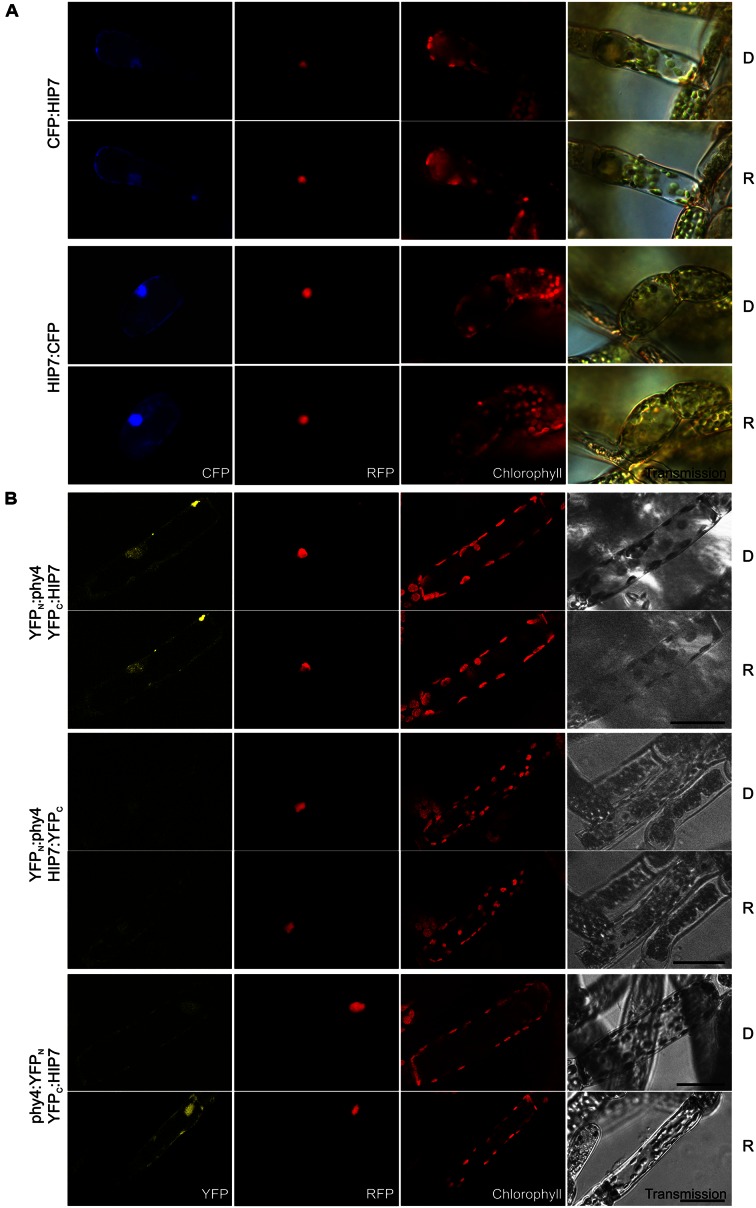
**HIP7 (Pp3c17_9390V1.1) intracellular localization **(A)** and split-YFP-studies of HIP7–phy4 interaction **(B)** each without (D) and with R pre-treatment (R) using fluorescence- and confocal microscopy, respectively.** Overall figure structure is analogous to **Figure 1**. **(A)** Intracellular localization of CFP:HIP7- (rows 1 and 2) and HIP7:CFP-fusions (rows 3 and 4) was observed, showing nuclear and cytoplasmic signal patterns (column 1, CFP). CFP:HIP7 in addition showed cytosolic foci in ∼40% of the cells observed. **(B)** Three of four possible split-YFP configurations were analyzed: YFP_N_:phy4–YFP_C_:HIP7 (rows 1 and 2), YFP_N_:phy4–HIP7:YFP_C_ (rows 3 and 4) and phy4:YFP_N_–YFP_C_:HIP7 (rows 5 and 6). YFP_N_:phy4–YFP_C_:HIP7, and phy4:YFP_N_–YFP_C_:HIP7 yielded significant split-YFP-signals mostly in nucleus and cytoplasm (column 1, YFP). In the latter case significant signals were only seen after R-pre-treatment (D and R conditions here being shown from independent experiments). Scale bars 30 μm.

The singleton HIP7 (31.5 kDa) is likely to represent a **Ser/Thr protein kinase**. In Y2H studies full length HIP7 interaction with phy4 was comparatively weak but nevertheless R-enhanced (**Supplementary Figure S2**), strongest growth being observed for the phy4:BD–ADg:HIP7 configuration. HIP7 fusions with CFP were intracellularly distributed in the nucleus and the cytoplasm (**Figure 6A**) with CFP:HIP7 in particular showing cytosolic foci in ∼40% of the cells observed (data not shown). In split-YFP, nuclear and cytoplasmic signals were seen for YFP_C_:HIP7 in combination with both YFP_N_:phy4 and phy4:YFP_N_, in accordance with the localization data (**Figure 6B**). Since the split-YFP signals were significantly weaker than those from the RFP:NLS nuclear marker, we checked for RFP bleed-through by narrowing detection to 10 nm around the emission maximum of YFP. As the fluorescence pattern was unchanged, we consider the signal to represent a *bona fide* nuclear interaction. Interestingly, the phy4:YFP_N_–YFP_C_:HIP7-interaction appeared to be R-dependent, the signal only being observable after illumination, implying Pfr-dependent binding *in planta*.

Holophytochrome-interacting protein 7 is apparently expressed under all conditions and in all cell types (see Supplementary Materials). Some Genevestigator data groups imply synexpression with phy4 as does the Phytozome data.

HIP7 shows low but significant similarity to various *Arabidopsis* Ser/Thr-protein kinases such as HT1 (AT1G62400), a kinase with an armadillo repeat domain (AT5G18700), a MAP kinase (AT1G73660), and a PAS-domain-containing protein tyrosine kinase (AT3G06620) with ∼40% query coverage and ∼30% sequence identity). HIP7-based BLAST searches of *Viridiplantae* proteins yielded many with somewhat more concordant similarity, most of which have been judged to be HT1 relatives. *Arabidopsis* HT1 is a protein kinase involved in the regulation of stomatal aperture (Hashimoto et al., 2006). Although stomatal opening is generally seen as a phototropin-mediated B effect, R is effective in maintaining stomatal opening, a response specifically impaired in *ht1*^-^ (Matrosova et al., 2015). Although the effect can largely be explained by photosynthetic CO_2_-depletion, a separate photoreceptor is probably also involved. Indeed, end-of-day FR treatment accelerated stomatal closure in *Phaseolus* (Holmes and Klein, 1985). Mosses develop stomata in the epidermis of the sporophyte: whereas they show responses to ABA similar to those of higher plants (Chater et al., 2011), light effects have not been studied. HIP7 might in any case be involved in other signaling pathways involving post-translational phosphorylation. Indeed, *Arabidopsis* HT1 is also expressed in stem, flower and even root tissues, implying diverse but still unknown cellular functions.

#### HIP8 (Pp3c11_25550V1.1)

**FIGURE 7:**
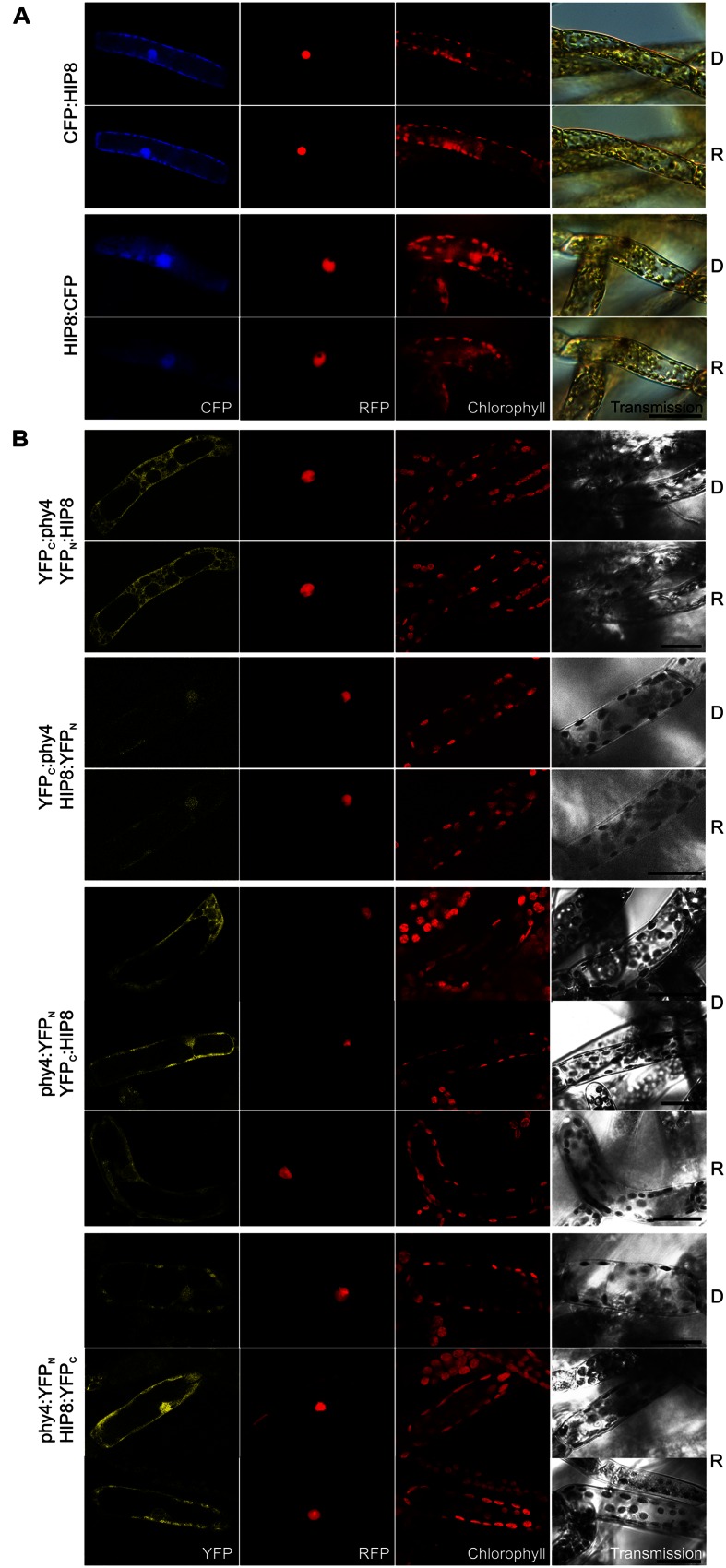
**HIP8 (Pp3c11_25550V1.1) intracellular localization **(A)** and split-YFP-studies of HIP8–phy4 interaction **(B)** each without (D) and with R pre-treatment (R) using fluorescence- and confocal microscopy, respectively.** Overall figure structure is analogous to **Figure 1**. **(A)** CFP:HIP8- (rows 1 and 2) and HIP8:CFP-fluorescence (rows 3 and 4) was observed in nucleus and cytoplasm (column 1, CFP). **(B)** All four split-YFP configurations were studied, however, in the case of YFP_N_:phy4–YFP_C_:HIP8 and YFP_N_:phy4–HIP8:YFP_C_ the negative controls showed significant signals (thus these configurations were replaced by YFP_C_:phy4–YFP_N_:HIP8 and YFP_C_:phy4–HIP8:YFP_N_). Analyses of YFP_C_:phy4–YFP_N_:HIP8 (rows 1 and 2), YFP_C_:phy4–HIP8:YFP_N_ (rows 3 and 4), phy4:YFP_N_–YFP_C_:HIP8 (rows 5–7; two different signal patterns in D) and phy4:YFP_N_–HIP8:YFP_C_ (rows 8–10; two different signal patterns in R) resulted in either nucleo-cytoplasmic or exclusively cytoplasmic signals in all configurations except YFP_C_:phy4–HIP8:YFP_N_ (column 1, YFP). Scale bars 30 μm.

Holophytochrome-interacting protein 8 (55.4 kDa) harbors a predicted protein kinase catalytic domain and several calcium-binding EF-hands and thus probably constitutes a **CDPK**. Y2H implied only weak interaction with phy4, but the signal was enhanced in Rc and stronger with BD:phy4 than with phy4:BD (**Supplementary Figure S2**). Expression of N- and C-terminal FP-fusions resulted in cytoplasmic and nuclear fluorescence signals approximately with equal signal intensities in both compartments with no apparent effect of light pre-treatment (**Figure 7A**). *In planta* interaction was verified for all combinations except YFP_C_:phy4–HIP8:YFP_N_ (**Figure 7B**). Cytoplasmic interaction was clearly enhanced by R pre-treatments in phy4:YFP_N_–YFP_C_:HIP8, although in phy4:YFP_N_–HIP8:YFP_C_ this was less pronounced. Although this implies that either the phy4–HIP8 complex is enhanced in or translocates to the cytoplasm in R, this behavior was only seen when the tag was fused to phy4 C-terminus: YFP_C_:phy4–YFP_N_:HIP8 interaction was exclusively cytoplasmic.

Holophytochrome-interacting protein 8 is apparently expressed under all conditions and in all cell types but shows strongly increased transcript levels during leaflet dedifferentiation (see Supplementary Materials). The Genevestigator data for treatments provide some evidence for synexpression with phy4 although this is not apparent for the other datasets.

The eukaryotic second messenger Ca^2+^ is involved in plant responses to various abiotic and biotic stimuli (Kudla et al., 2010), primary targets being Ca^2+^-channels and transporters (Wheeler and Brownlee, 2008). Ca^2+^ from the apoplast or the vacuole thereby briefly increases the local concentration near the plasma membrane or organelle membrane – a phenomenon known as the calcium signature. Prior to the discovery of Pfr-dependent nuclear translocation and its interaction with the PIF family of transcription factors in the nucleus, Ca^2+^ had been suggested to be a key player in phytochrome signaling, showing crucial roles in chloroplast development and LHC expression, phyA-mediated light regulation of asparagine synthase, R-induced stem protoplast swelling and phyA- and PIF3-mediated regulation of succinate dehydrogenase (Neuhaus et al., 1993, 1997; Bowler et al., 1994; Long and Iino, 2001; Eprintsev et al., 2013). Indeed, there is evidence that phytochromes initiate Ca^2+^ and protein phosphorylation signals via CDPK’s (Datta et al., 1985; Li et al., 1991). Furthermore, Ca^2+^ addition to *Cucumis* cotyledon extracts can mimic the R/FR effects on phosphorylation and at least two CDPK’s were present whose activities were modulated by phytochromes (Vidal et al., 2007). CDPK’s possess a N-terminal variable domain, a protein kinase-, an autoinhibitory and a C-terminal calmodulin-like domain, the latter typically consisting of two pairs of EF-hand motifs (Rutschmann et al., 2002; Christodoulou et al., 2004). CDPKs directly bind Ca^2+^, leading to a conformational change and kinase de-repression, thereby converting the Ca^2+^ signal into phosphorylation of metabolic enzymes, ion channels and transcription factors and thus into diverse downstream cellular responses (reviewed in Hamel et al., 2014). Kinase activity can also be regulated by (auto)phosphorylation/dephosphorylation, phospholipids and 14-3-3-proteins (reviewed in Cheng et al., 2002), however. Interestingly, the holo phy4:BD-screen also identified a 14-3-3 protein (see HIP14). Overall domain organization seems to be conserved in all CDPK’s, and land plant CDPK’s usually cluster in four distinct clades (Hamel et al., 2014). Comparison of the HIP8 amino acid sequence with the *Viridiplantae* protein database indeed revealed numerous orthologs with high levels of similarity, the *Arabidopsis* and *Physcomitrella* genomes encoding 34 and 25 CDPK’s, respectively (Hamel et al., 2014). The only moss representative characterized to-date seems to be associated with nutrient starvation (Mitra and Johri, 2000). Several *Arabidopsis* CDPK’s such as CPK1 - the most similar to HIP8 (covering 91% of the query with 65% identity) – are thought to be involved in ABA signaling, regulation of stomatal aperture, and stress responses (Mori et al., 2006; Ma and Wu, 2007; Zhu et al., 2007; Geiger et al., 2010; Zou et al., 2010; Brandt et al., 2012; Boudsocq and Sheen, 2013; Liese and Romeis, 2013). Although CPK1 is membrane-/peroxysome-localized and thus probably involved in oxidative stress and lipid metabolism (Dammann et al., 2003), this is not the case for HIP8 which thus probably has different functions. *Arabidopsis* CPK17 and perhaps other CDPKs on the other hand are involved in Ca^2+^-mediated pollen tube tip growth (Myers et al., 2009; Hamel et al., 2014). As *Physcomitrella* filament tip growth shows many similarities to that of pollen tubes including a tip-focused calcium gradient (Meske and Hartmann, 1995; Meske et al., 1996; see HIP13), we speculate that HIP8 might play a related role in phy4-steered phototropism. On the other hand, whereas *Arabidopsis* CPK17 is plasma membrane-localized (Hamel et al., 2014), this seems not to be the case for HIP8 (**Figure 7**). However, in both algae and higher plants several CDPKs are associated with the cortical actin cytoskeleton and cytoplasmic streaming (Putnam-Evans et al., 1989; McCurdy and Harmon, 1992). Light-induced cytoplasmic streaming is also mediated by phytochrome in the angiosperm *Vallisneria*. This Ca^2+^-and actin-dependent response is of particular interest regarding phytochrome cytoplasmic signaling as it is restricted to the area of irradiation and begins within 3 s of irradiation (Takagi et al., 2003), thereby ruling out a transcription-translation-based transduction mechanism. If the functions of actin-associated CDPKs and HIP8 are indeed related, it might represent the molecular link between the directional signal sensed by phytochrome and cytoskeleton-steered tip cell growth/bending response.

#### HIP9 (Pp1s254_10V6.2)

**FIGURE 8:**
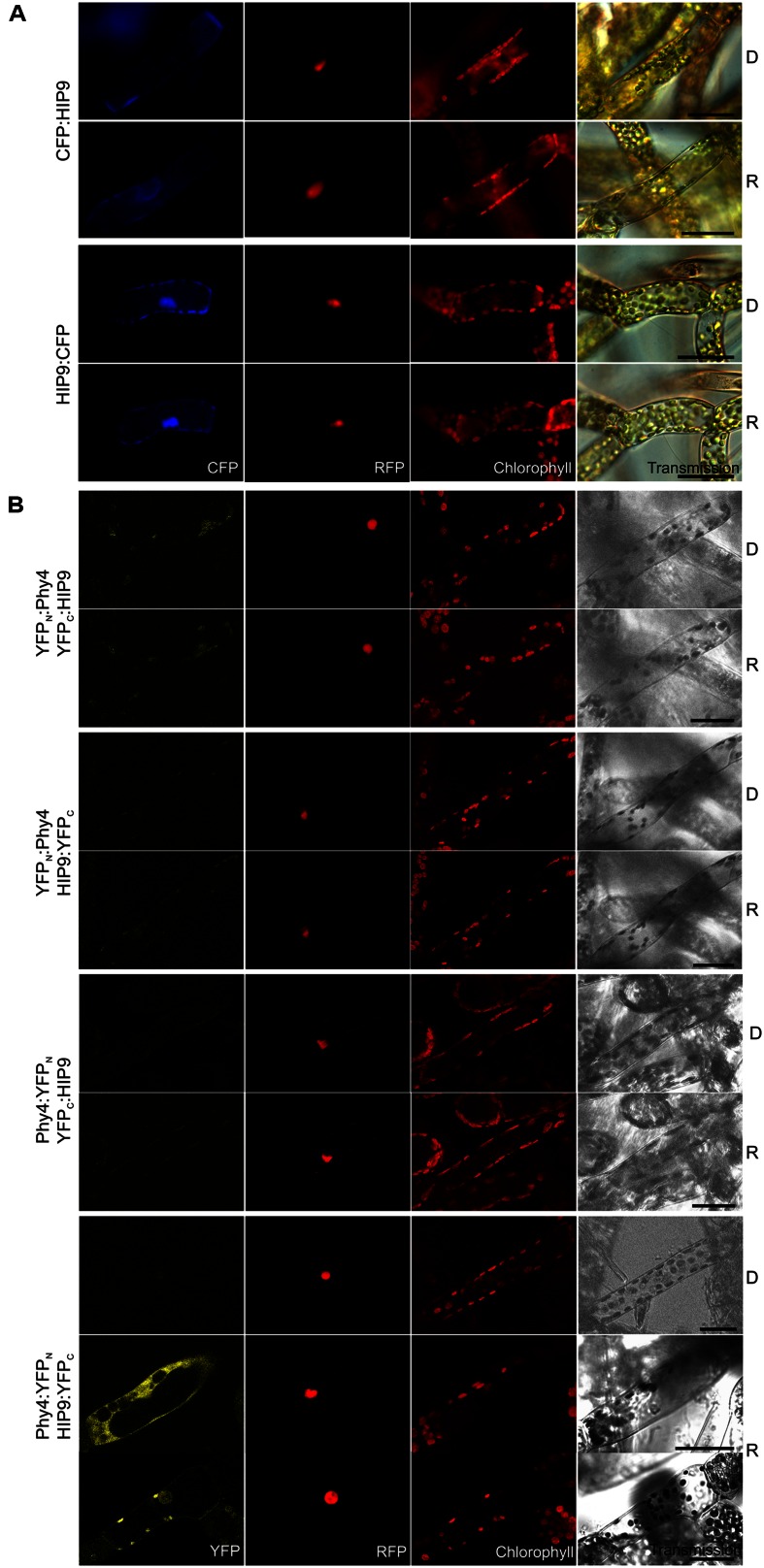
**HIP9 (Pp1s254_10V6.2) intracellular localization **(A)** and split-YFP-studies of HIP9–phy4 interaction **(B)** each without (D) and with R pre-treatment (R) using fluorescence- and confocal microscopy, respectively.** Overall figure structure is analogous to **Figure 1**. **(A)** CFP:HIP9- (rows 1 and 2) and HIP9:CFP-fluorescence (rows 3 and 4) was observed in the cytoplasm or the nucleus and cytoplasm, respectively (column 1, CFP). **(B)** All possible split-YFP configurations were investigated: YFP_N_:phy4–YFP_C_:HIP9 (rows 1 and 2), YFP_N_:phy4–HIP9:YFP_C_ (rows 3 and 4), phy4:YFP_N_–YFP_C_:HIP9 (rows 5 and 6) and phy4:YFP_N_–HIP9:YFP_C_ (rows 7–9; two distinct patterns in R). However, two different signal patterns (nucleus and cytoplasm or perhaps nucleus and plasma membrane) were only observed for phy4:YFP_N_–HIP9:YFP_C_ after R pre-treatment (column 1, YFP; D and R images here from separate experiments). Scale bars 30 μm.

Holophytochrome-interacting protein 9 (63.9 kDa) harbors a predicted **rhomboid peptidase S54 domain** and predicted transmembrane regions. Y2H studies with phy4 showed weak but R-enhanced interaction with full length HIP9 in all configurations tested (**Supplementary Figure S2**). *In planta* localization studies showed cytoplasmic and perhaps plasma membrane (CFP:HIP9) or cytoplasmic and nuclear localization with no sign of membrane association (HIP9:CFP; **Figure 8A**). Although the predicted protein size (64 kDa + 27 kDa tag) exceeds the nuclear pore exclusion limit, the C-terminal FP-fusion was able to enter the nucleus. Indeed, two NLS in the N-terminal region are predicted by NucPred^7^ (Brameier et al., 2007), so nuclear import is not unreasonable. Interaction was confirmed *in planta* only in the phy4:YFP_N_–HIP9:YFP_C_ configuration following R pre-treatment (in harmony with the Y2H data; **Figure 8B**). In this case, fluorescence was generally distributed between cytoplasm and nucleus although occasionally a signal at the plasma membrane was apparent.

Holophytochrome-interacting protein 5 is apparently expressed under all conditions and in all cell types (see Supplementary Materials). The data provided no evidence for synexpression with phy4, however.

Rhomboid domain proteases are ubiquitous intramembrane serine proteases (Urban et al., 2001; Koonin et al., 2003; Urban and Wolfe, 2005). According to crystal structures, RBL’s possess seven transmembrane helices forming a conical cavity accessible to the aqueous surroundings, enabling cleavage of a transmembrane helix with the help of serine and histidine residues forming a catalytic dyad (Kanaoka et al., 2005; Baker et al., 2007; Ben-Shem et al., 2007). The *Arabidopsis* and *Physcomitrella* genomes encode 17 and 16 RBL’s, respectively (Li et al., 2015), including the homolog (Pp3c22_8560V1.1, see Summary **Table 1** in the Supplementary Material). Of those in *Arabidopsis*, four show poorly conserved active sites, two localizing to the Golgi apparatus suggesting a role in secretion, while others localize to mitochondria, plastids, and perhaps also the endoplasmic reticulum and the plasma membrane, implying different functions (Kanaoka et al., 2005; Lemberg and Freeman, 2007; Kmiec-Wisniewska et al., 2008; Knopf et al., 2012; Thompson et al., 2012). Transcription factors represent potential targets, ∼10% of *Arabidopsis* transcription factors requiring release from membranes to become functional (Kim and Park, 2007; Seo et al., 2008). RBL’s have also been shown to be involved in Ca^2+^-mediated stress responses, fertility and flower development, structural and functional maintenance of chloroplast and mitochondrion integrity and mitochondrial retrograde signaling (Aguilar-Hernandez et al., 2011; Thompson et al., 2012; Ng et al., 2013). HIP9 is most similar to RBL 9 (At5g38510), predicted to be inactive due to the lack both catalytic dyad amino acids but retaining the glycine within the GxSx rhomboid protease motif, perhaps representing a modified active site (Lemberg and Freeman, 2007). The substrates and functions of this RBL group are not known, however. Thus whether HIP9 is indeed an RBL membrane protein with switchable protease activity is doubtful. In particular, rather than membrane integration, our data implies at most a weak association with the plasma membrane. Whereas this would correlate with the phy4–phot interaction at the plasma membrane (Jaedicke et al., 2012), an involvement of HIP9 in that signaling complex remains no more than a possibility.

#### HIP11 (Pp3c20_16210V1.1)

**FIGURE 9:**
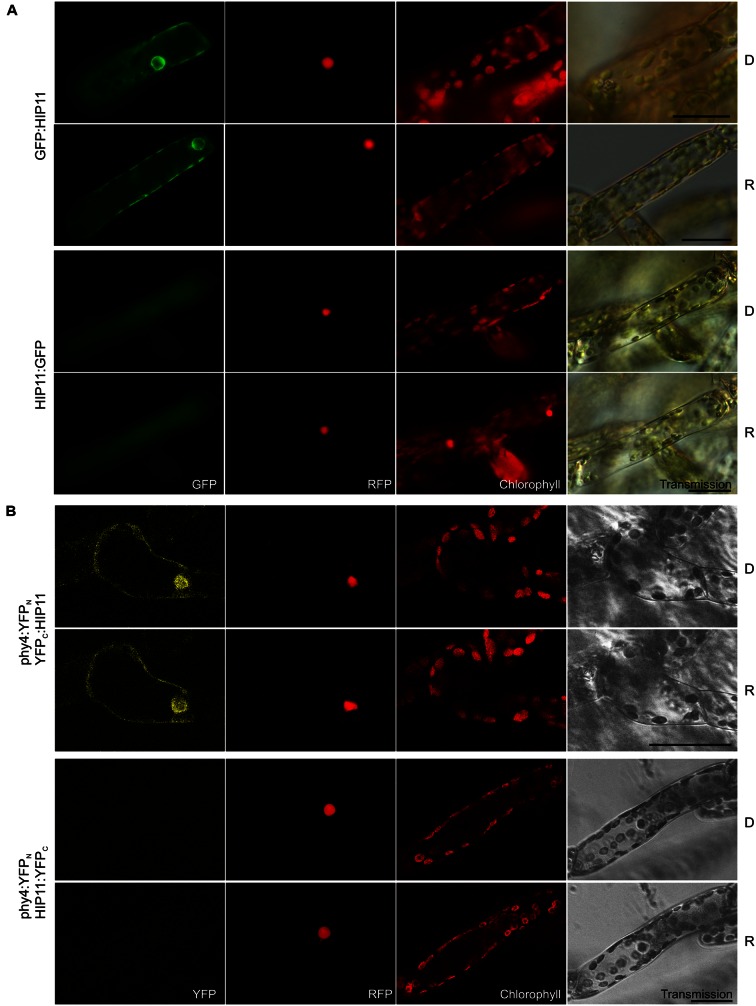
**HIP11 (Pp3c20_16210V1.1) intracellular localization **(A)** and split-YFP-studies of HIP11–phy4 interaction **(B)** each without (D) and with R pre-treatment (R) using fluorescence- and confocal microscopy, respectively.** Overall figure structure is analogous to **Figure 1**. **(A)** GFP:HIP11 (rows 1 and 2) and HIP11:GFP (rows 3 and 4) showed cytoplasmic and perinuclear or cytoplasmic localization, respectively (column 1, GFP). In a subset of cells cytoplasmic and nuclear localization could be observed in both cases (not shown). **(B)** Corresponding to the Y2H interaction behavior, split-YFP configurations in combination with C-terminally fused phy4 were analyzed: phy4:YFP_N_–YFP_C_:HIP11 (rows 1 and 2) and phy4:YFP_N_–HIP11:YFP_C_ (rows 3 and 4). (Peri)nuclear and cytoplasmic signals were observed in the former case alone (column 1, YFP). Scale bars 30 μm.

Holophytochrome-interacting protein 11, (20.2 kDa) shows low similarity to the *Arabidopsis* pfkB-like carbohydrate kinase family protein AT5G19150. A likely homolog in *Physcomitrella* is Pp3c7_8840V1.1. Holo-BD:phy4 interacted weakly with ADg:HIP11 and HIP11:ADg in Y2H, whereas interactions of holo-phy4:BD with HIP11 were light-enhanced but fully FR-reversible only in case of ADg:HIP11 (**Supplementary Figure S2**). GFP:HIP11 and HIP11:GFP mostly showed cytoplasmic/perinuclear and rather unstructured cytoplasmic localization, respectively (**Figure 9A**), although in a subset of cells cytoplasmic and nuclear localization could be observed in both cases (not shown). *In planta* interaction was verified in the phy4:YFP_N_–YFP_C_:HIP11 configuration, the split-YFP signal being apparent in the cytoplasm and perinuclear region (**Figure 9B**) in accordance with the localization data.

Holophytochrome-interacting protein 11 is apparently expressed very differently under different conditions and particularly weakly at early developmental stages (such as in spores and protonemata). There is little evidence for synexpression with phy4.

Although HIP11 was predicted by InterPro to carry an N-terminal transmembrane region (residues 7–25), we found no direct evidence of membrane-association. On the other hand, its small size and an NLS (RRRR, residues 112–115, Brameier et al., 2007) predicted by NucPred are consistent with the occasional nuclear localization we observed. As BLAST searches revealed only 31% identity within the 36% query coverage with respect to *Arabidopsis* AT5G19150 (whose function is unknown), HIP11 and Pp3c7_8840V1.1 might be functionally unique to mosses.

#### HIP12 (Pp3c13_15620V1.1)

**FIGURE 10:**
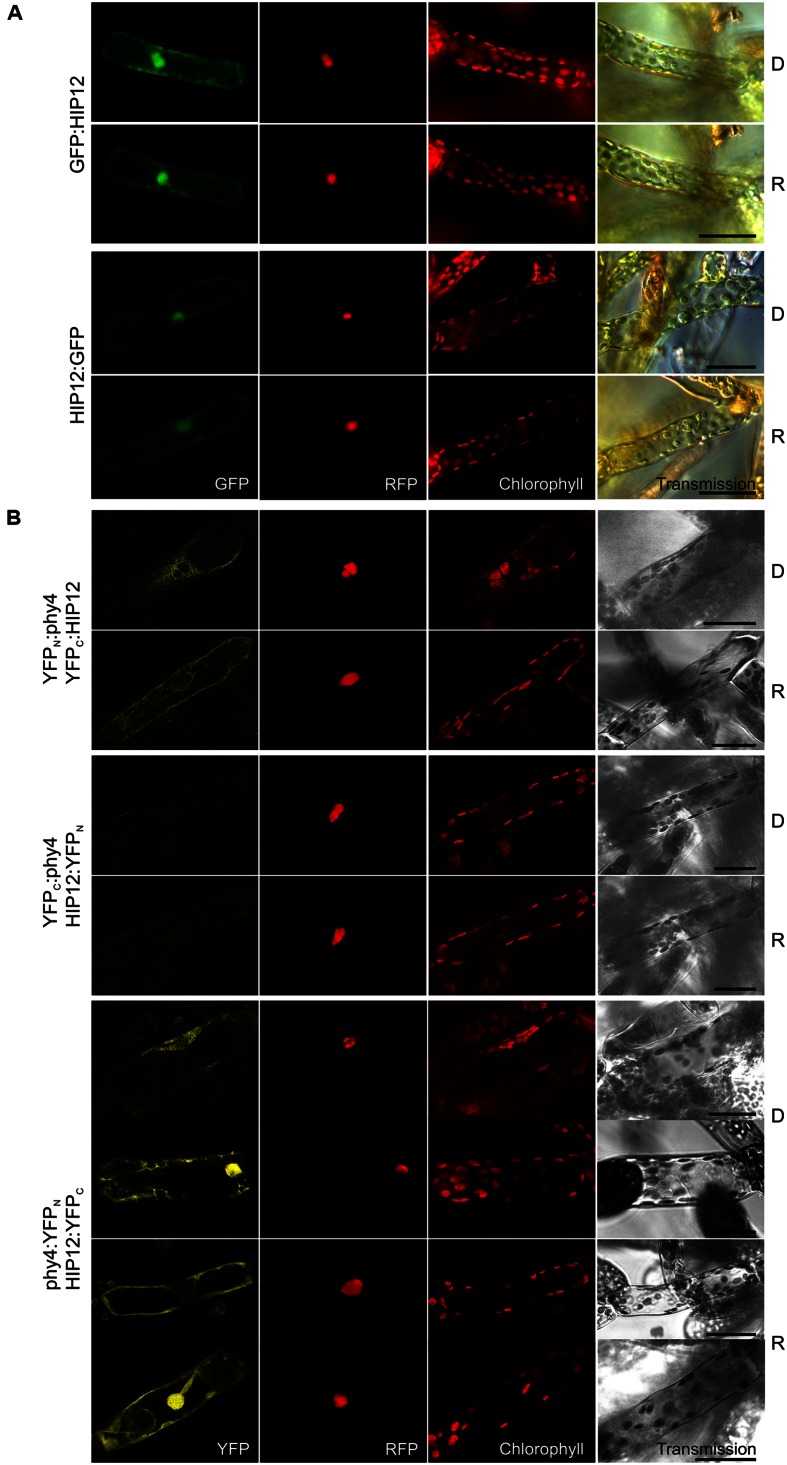
**HIP12 (Pp3c13_15620V1.1) intracellular localization **(A)** and split-YFP-studies of HIP12–phy4 interaction **(B)** each without (D) and with R pre-treatment (R) using fluorescence- and confocal microscopy, respectively.** Overall figure structure is analogous to **Figure 1**. **(A)** N-and C-terminally GFP-tagged HIP12 localized to nucleus and cytoplasm (rows 1 and 2; and 3 and 4, respectively; column 1, GFP). In some cases the HIP12:GFP signal was confined largely to the cytoplasm (not shown). **(B)** All four split-YFP configurations were tested, however, YFP_N_:phy4–HIP12:YFP_C_ and phy4:YFP_N_–YFP_C_:HIP12 were excluded because the respective negative controls showed significant signals. Thus the following configurations are shown: YFP_N_:phy4–YFP_C_:HIP12 (rows 1 and 2), YFP_C_:phy4–HIP12:YFP_N_ (rows 3 and 4) and phy4:YFP_N_–HIP12:YFP_C_ (rows 5–8; two distinct patterns in both conditions). All configurations except YFP_C_:phy4–HIP12:YFP_N_ yielded significant cytoplasmic or nucleo-cytoplasmic signals (column 1, YFP). Scale bars 30 μm.

Holophytochrome-interacting protein 12 (17.5 kDa) is the evolutionarily and structurally conserved **eukaryotic translation initiation factor 5A** (eIF-5A). In Y2H studies, interaction of HIP12/eIF-5A with phy4 was relatively weak but nevertheless light-induced, strong and FR-reversible and constitutive in cases of BD:phy4–ADg:HIP12/BD:phy4–HIP12:ADg, phy4:BD–ADg:HIP12, and phy4:BD–HIP12:ADg, respectively (**Supplementary Figure S2**). Irrespective of light pre-treatments or FP position, HIP12/eIF-5A was localized in the cytoplasm and nucleus (**Figure 10A**), presumably because the 18 kDa + 27 kDa FP chimera would be small enough to enter the nucleus passively, although in some HIP12:GFP-cells nuclear fluorescence was much weaker than in the cytoplasm (data not shown). Interaction was confirmed *in planta* although the site of localization was strongly dependent on the construct used (see **Figure 10B**). Whereas phy4:YFP_N_–HIP12:YFP_C_-interaction was seen in both nucleus and cytoplasm or cytoplasm only, YFP_N_:phy4–YFP_C_:HIP12 was apparent only in the cytoplasm. YFP_C_:phy4–HIP12:YFP_N_ on the other hand failed to show significant interaction. phy4:YFP_N_–YFP_C_:HIP12 was omitted from the figure as the negative control :YFP_N_–YFP_C_:HIP12 showed substantial fluorescence signals, implying an artifactual interaction.

Holophytochrome-interacting protein 12 is apparently strongly expressed in under most conditions and in most cell types except leaflets (see Supplementary Materials). According to Genevestigator HIP12 expression is only weakly affected by different treatments but increases strongly on dedifferentiation of the leaflet cells (see Supplementary Materials). The Genevestigator treatment data alone provided evidence for synexpression with phy4.

Basic local alignment search tool analysis revealed a likely HIP12 homolog in *Physcomitrella* (Pp3c12_3540V1.1) and three in *Arabidopsis*. Despite its name, eIF-5A is now generally considered to be involved in translation elongation rather than initiation (Kemper et al., 1976; Gregio et al., 2009; Patel et al., 2009; Saini et al., 2009; Tong et al., 2009). The association of phy4 with eIF-5A and thus translation provides an interesting possible connection to the reported association of phyB Pfr with the cytoplasmic translational regulator PENTA1 and associated inhibition of PORA translation (Paik et al., 2012). Interestingly, in fission yeast eIF-5A has been associated with organization of the cytoskeleton, cell polarity and bud formation (Weir and Yaffe, 2004; Zanelli and Valentini, 2005; Chatterjee et al., 2006), providing a potential link to steering of *Physcomitrella* tip cell growth by phy4 (see also HIP13 below).

The complex localization behavior of HIP12 we observed *in planta* correlates with observations in other systems. Mammalian eIF-5A is localized in the cytoplasm and perinuclear region where it interacts with ribosomes attached to the endoplasmic reticulum, forming a network-like structure (Shi et al., 1996) resembling the pattern we observed (see **Figure 10**). Post-translational conversion of a lysine residue to hypusine (Chen and Liu, 1997; Park et al., 1997) regulates eIF5-A’s activity and localization, however, the precursor being found in both cytoplasm and nucleus whereas the hypusinated eIF-5A localizes predominantly to the cytoplasm (Park et al., 1982; Chen, 1983; Torrelio et al., 1984; Park, 1989; Lee et al., 2009). In maize, phosphorylation of the terminal serine changes eIF-5A-2 localization, unphosphorylatable mutants localizing to nucleus and cytoplasm, while phospho-mimic mutants sequester to the nucleus (Lebska et al., 2010; Lewandowska-Gnatowska et al., 2011). Indeed it was suggested that the phyA-associated phosphatase PP2A (see HIP14 below) is responsible for the dephosphorylation. In *Arabidopsis* several isoforms have been identified and associated with xylem formation, abiotic stress tolerance and responses to cytokinin including senescence (Chamot and Kuhlemeier, 1992; Wang et al., 2001, 2012; Thompson et al., 2004; Feng et al., 2007; Liu et al., 2008; Ren et al., 2013). HIP12 is most similar to eIF-5A-3, however, which is preferentially expressed in the phloem but whose function is not known (Ma et al., 2010).

#### HIP13 (Pp3c1_23670V1.1)

**FIGURE 11:**
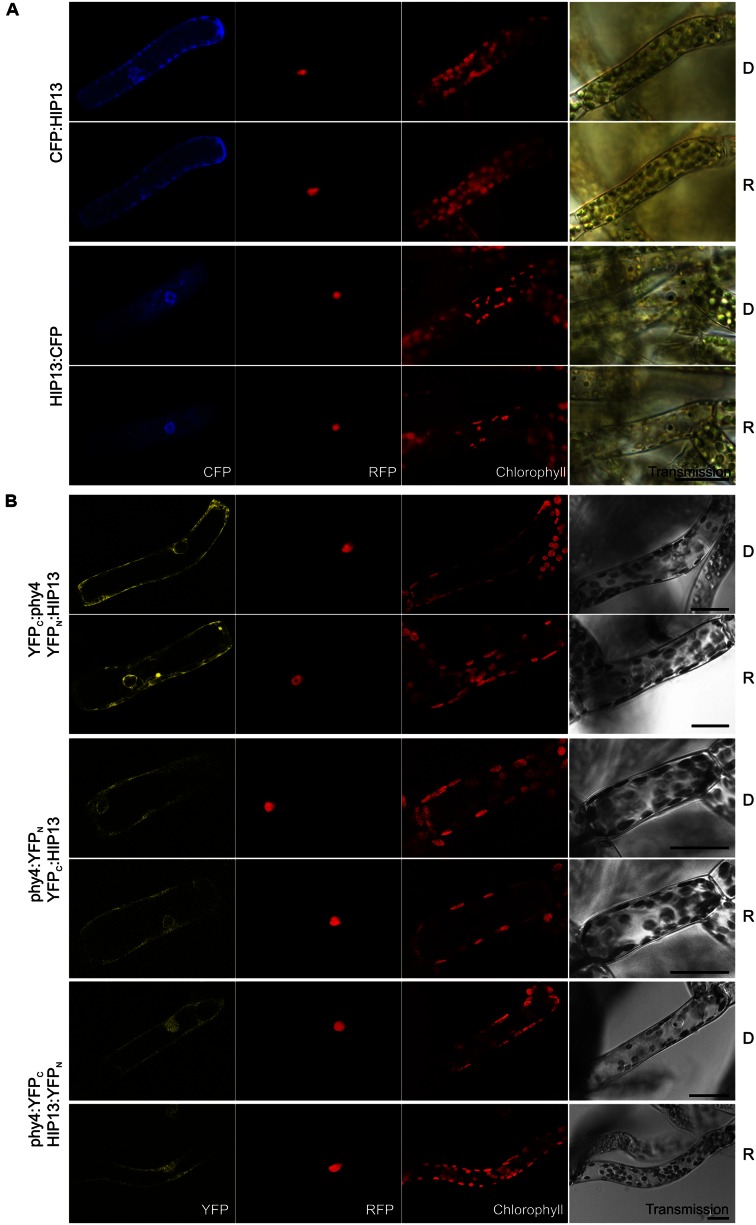
**HIP13 (Pp3c1_23670V1.1) intracellular localization **(A)** and split-YFP studies of HIP13–phy4 interaction **(B)** each without (D) and with R pre-treatment (R) using fluorescence- and confocal microscopy, respectively.** Overall figure structure is analogous to **Figure 1**. **(A)** Both CFP:HIP13 (rows 1 and 2) and HIP13:CFP (rows 3 and 4) were localized in the cytoplasm and perinuclear region (column 1, CFP). **(B)** All four split-YFP configurations were tested, however, YFP_C_:phy4–HIP13:YFP_N_ and phy4:YFP_C_–YFP_N_:HIP13 were excluded because the respective negative controls showed significant signals. Thus the following configurations are shown: YFP_C_:phy4–YFP_N_:HIP13 (rows 1 and 2), phy4:YFP_N_–YFP_C_:HIP13 (rows 3 and 4) and phy4:YFP_C_–HIP13:YFP_N_ (rows 5 and 6), all yielding significant cytoplasmic signals with the exception of phy4:YFP_C_–HIP13:YFP_N_ the signal which could be observed in nucleus and cytoplasm (column 1, YFP). Scalebars 30 μm.

Holophytochrome-interacting protein 13 (49.3 kDa) is the eukaryotic/archaeal **translation elongation factor subunit** **EF1α**. Y2H studies showed constitutive interaction with HIP13/EF1α in case of BD:phy4 and rather weak but R-enhanced interaction with phy4:BD, respectively (**Supplementary Figure S2**). Irrespective of FP-fusion configuration or light treatment, HIP13/EF1α localized to the cytoplasm (**Figure 11A**) and perinuclear region. Accordingly, most split-YFP signals also constitutively appeared in the cytoplasm and perinuclear region (YFP_C_:phy4–YFP_N_:HIP13 and phy4:YFP_N_-YFP_C_:HIP13, **Figure 11B**). In particular, YFP_C_:phy4–YFP_N_:HIP13 cytoplasmic signals resembled cytoskeleton-like substructures. phy4:YFP_C_–HIP13:YFP_N_ signals were additionally seen in the nucleus, however. As the negative controls YFP_C_: or YFP_N_: with HIP13:YFP_N_ or HIP13:YFP_C_, respectively, produced substantial signals in the cytoplasm and/or nucleus, the respective combinations with N-terminally fused phy4 were categorized as false positive signals and excluded from discussion.

Holophytochrome-interacting protein 13 is apparently expressed strongly under most conditions and in most cell types but weakly in spores and leaflets (see Supplementary Materials). There is little evidence for synexpression with phy4.

Translation elongation factor 1α is an extremely conserved multifunctional protein harboring an N-terminal RAS-like GTPase (also called p-loop NTPase) and C-terminal GTP EFTU-D2 and -D3 domains. The RAS-like GTPase domain comprises several nucleotide-binding sites whereas the D3-domain is responsible for actin binding (Gross and Kinzy, 2005). As the name implies, EF1α plays an essential role in translational elongation, thus interaction with phy4 might relate to phytochrome-regulated PENTA1-mediated phytochrome regulation of translation (Paik et al., 2012) as discussed for HIP12. On the other hand, EF1α has been shown to bind and bundle actin filaments in various species (Demma et al., 1990; Dharmawardhane et al., 1991; Bassell et al., 1994; Collings et al., 1994). EF1α constitutes 1–2% of total protein in normal growing cells (Slobin, 1980; Dharmawardhane et al., 1991), more than half of which is estimated to be associated with actin (Edmonds et al., 1995). Interaction with actin is dependent on both EF1α-GTP binding and phosphorylation status (Dharmawardhane et al., 1991; Yang and Boss, 1994; Izawa et al., 2000). EF1α activates phosphatidylinositol 4-kinase (Yang et al., 1993) thereby regulating the capping and severing of actin filaments proteins. It also serves as a substrate for Rho-associated and Ca^2+^-dependent protein kinases (Yang and Boss, 1994; Izawa et al., 2000). Moreover, EF1α binds and severs microtubules in a Ca^2+^/calmodulin-dependent manner in various organisms (Durso and Cyr, 1994; Kaur and Ruben, 1994; Shiina et al., 1994). Cytoskeleton-binding of EF1α probably links the translation machinery to the spatial organization of the cell whereby EF1α regulates transport and anchorage of mRNA and the compartmentalization of translation in eukaryotic cells (Gross and Kinzy, 2005).

Four and ∼11 EF1α-homologs exist in *Arabidopsis* and *Physcomitrella*, respectively (Ransom-Hodgkins, 2009). EF1α’s promiscuous actions in the cell, in particular its association with the cytoskeleton and possibly Ca^2+^ signaling, make it an interesting candidate for a role in phy4-mediated phototropism in filament tip cells. In unilateral R the Pfr distribution at the plasma membrane somehow generates a Ca^2+^-gradient at the illuminated side of the cell, shifting the site of F-actin synthesis (Hartmann and Weber, 1988; Meske and Hartmann, 1995; Meske et al., 1996). As a result, a directionally translocated actin cap forms, defining the new center of outgrowth corresponding to polarized vesicle transport along the actin bundles (Schnepf, 1986; Meske et al., 1996). More recently, profilin 2 has been shown to be involved in actin turnover and in actin cytoskeleton-mediated vesicular trafficking together with EF1α in *Arabidopsis* root cells (Takac et al., 2011). Indeed, profilin is essential for tip cell growth in *Physcomitrella* (Vidali et al., 2007). Profilin together with actin and CHUP1 are also central players in chloroplast movement responses in *Arabidopsis* (Schmidt von Braun, 2008; Schmidt von Braun and Schleiff, 2008). Chloroplast photorelocation is a cytoplasmic directional response mediated by phytochrome in mosses. Given that CHUP1 is also present in *Physcomitrella*, a machinery involving phytochrome, profilin, EF1α and the actin-filament network can be envisaged. It should be recalled, however, that the equivalent response in *Arabidopsis* is mediated by the blue light receptor phototropin, phytochrome playing a modulating role (DeBlasio et al., 2003; Luesse et al., 2010).

#### HIP14 (Pp3c3_8540C1.1)

**FIGURE 12:**
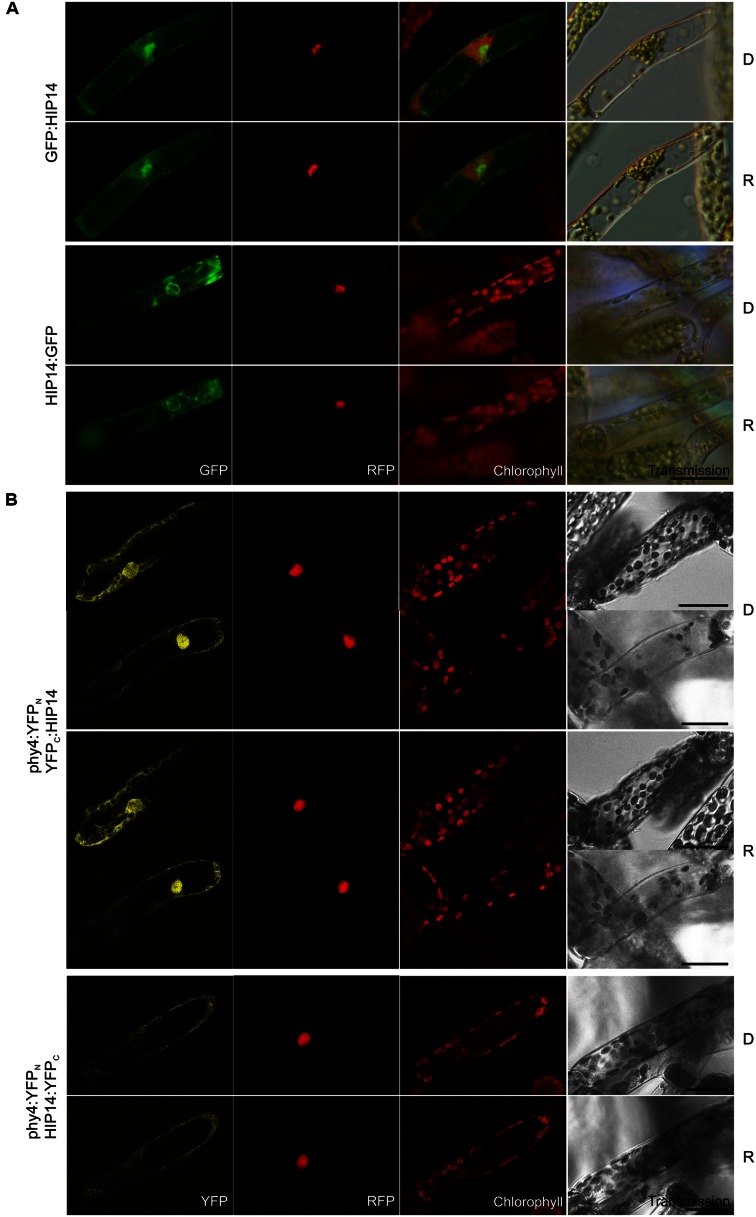
**HIP14 (Pp3c3_8540C1.1) intracellular localization **(A)** and split-YFP-studies of HIP14–phy4 interaction **(B)** each without (D) and with R pre-treatment (R) using fluorescence- and confocal microscopy, respectively.** Overall figure structure is analogous to **Figure 1**. **(A)** GFP:HIP14 (rows 1 and 2) and HIP14:GFP (rows 3 and 4) localized to cytoplasm and nucleus and to cytoplasm and perinuclear region showing a cytoskeleton-like pattern, respectively (column 1, GFP). **(B)** Corresponding to the Y2H interaction behavior, split-YFP configurations in combination with C-terminally fused phy4 were analyzed: phy4:YFP_N_–YFP_C_:HIP14 (rows 1–4; two distinct signal patterns in both conditions) and phy4:YFP_N_–HIP14:YFP_C_ (rows 5 and 6). All configurations tested yielded significant cytoplasmic or nucleo-cytoplasmic signals (column 1, YFP). Scale bars 30 μm.

Holophytochrome-interacting protein 14 (29.1 kDa) is a **14-3-3 protein** family member. Y2H analysis of phy4 interaction with the full-length protein model confirmed interaction only with the phy4:BD configuration (**Supplementary Figure S2**). *In planta* GFP:HIP14 localized to cytoplasm and nucleus, whereas the HIP14:GFP configuration was exclusively cytoplasmic showing a cytoskeleton-like pattern (**Figure 12A**) This pattern was not apparent in the GFP:HIP14 configuration. The HIP14-phy4 interaction was verified *in planta*: phy4:YFP_N_–YFP_C_:HIP14 on the one hand showed a strong nuclear and cytoplasmic signal, reflecting that of GFP:HIP14, and in addition showed an exclusively cytoplasmic, cytoskeleton-like pattern in a subset of cells (**Figure 12B**). The reconstituted phy4:YFP_N_–HIP14:YFP_C_ signal, like that of HIP14:GFP, was exclusively cytoplasmic although we did not observe cytoskeleton-like patterns.

Holophytochrome-interacting protein 14 is apparently expressed very differently under different conditions and in different cell types with little evidence for synexpression with phy4 (see Supplementary Materials).

Ubiquitous in eukaryotic cells (Isobe et al., 1991; Yaffe, 2002), 14-3-3 proteins possess a conserved central region involved in dimerization and interaction with client proteins via an amphipathic groove. 14-3-3-targets are mostly phosphothreonine and phosphoserine proteins harboring (R/K)(XX/S)X(S/T)^p^XP motifs, the conformation of which is affected by 14-3-3 binding, leading to changes in the target’s catalytic activity, binding to down-stream signaling components and/or intracellular localization (see de Boer et al., 2013). ∼10 other 14-3-3 proteins are encoded in the *Physcomitrella* genome. Comparison with the 13 14-3-3 family members in *Arabidopsis* showed HIP14 to be most similar to isoforms GF14 ψ and ω which are localized predominantly in the trichome nucleus and cytoplasm with cytoskeleton association (Paul et al., 2005), similarly to our *in planta* localization data for HIP14:GFP and split-YFP-data for phy4:YFP_N_–YFP_C_:HIP14 (see **Figures 12A,B**). Despite their sequence similarity, plant 14-3-3 proteins execute specific functions on account of cell- or stimulus-specific expression, differential phosphorylation or intracellular localization (Paul et al., 2005). Altogether >750 14-3-3 protein targets have been identified in *Arabidopsis*, thus their functions are diverse, including regulation of hormone signaling and responses to external stimuli such as light (see Denison et al., 2011). Certain 14-3-3 isoforms mediate stomatal opening via interaction with light-activated phototropins and subsequently binding to the phosphorylated/activated plasma membrane H^+^-ATPase (Kinoshita et al., 2003; Ueno et al., 2005; Sullivan et al., 2009; Hayashi et al., 2010, 2011; Tseng et al., 2012), while others may regulate chloroplast movement perhaps also by interaction with phototropin (Sullivan et al., 2009; Tseng et al., 2012). Additionally, 14-3-3 proteins are involved in photoperiodic control of flowering, probably by direct interaction with key players in floral induction such as CONSTANS and FT (florigen; Pnueli et al., 2001; Mayfield et al., 2007; Purwestri et al., 2009). Numerous 14-3-3 clients are involved in light signaling including RPT2, phot2, PP2A, COP1 and -8, CONSTANS-LIKE 1, and PIF3, as well as several other gibberellic acid/phytochrome signaling proteins (Shin et al., 2011). 14-3-3 κ^-^ and χ^-^ single and double null mutants resembled PIF3-overexpressors (Mayfield et al., 2007; Adams et al., 2014), implying a role of certain 14-3-3’s in plant phytochrome action perhaps by acting as antagonists/regulators of PIF3-dependent light-signaling. Further 14-3-3 clients included PRL1 (see HIP4 above) and phyB (Shin et al., 2011), providing intriguing corollaries to the present study. Moreover, whereas in Rc wild-type *Arabidopsis* seedlings show poor gravitropic orientation, 14-3-3 ν^-^ null mutants showed enhanced gravitropism, rather like those of *phyB*^-^ (Mayfield et al., 2007). Indeed, 14-3-3 protein association also with phototropin and cryptochrome signaling might imply involvement in different photoreceptor signaling complexes (see Paul et al., 2008), as was suggested for PKS1, NPH3, and PP2A (see Jaedicke et al., 2012). We speculate that the HIP14–phy4 interaction in *Physcomitrella* might be part of such a complex.

#### Comparison of phy4 and HIP Expression Data

Generally, partner proteins must be co-expressed (expressed in the same cells at the same times and under the same conditions) unless the absence of one or other is physiologically relevant. Furthermore, the expression levels of functionally associated genes are often correlated (synexpression). We therefore collected available expression data from eFP, Phytozome and Genevestigator for phy4 and HIP1-14 to investigate these aspects. phy4 is expressed to some extent in all analyzed developmental stages and conditions (see Supplementary Materials). Light effects are fairly small (∼twofold differences). Pairwise comparison of HIP and phy4 expression showed correlation implying synexpression in several cases (HIP4–8 and -12). We also performed a hierarchical clustering analysis of all three datasets according to condition and gene similarities to identify HIP’s with expression patterns similar to phy4 (see **Supplementary Figure S6**). The Genevestigator treatment effects on phy4 were most similar to HIP4 and -6 followed by -7 and -5. The most similar expression level patterns were for HIP12, -4, and -9 followed by -11 and -13 according to the Phytozome treatment data. The expression patterns of HIP4, -6, and -7 followed by -3 were most similar to phy4 according to the eFP developmental data and to HIP3 and -6 followed by -4, -7, and -9 according to Genevestigator anatomy data. Taken together, these comparisons imply synexpression of phy4 in particular with HIP4, -6, and -7, providing indirect support for their possible involvement in phy4 action.

## Conclusion

In summary, phy4 steers directional growth in moss filaments, thereby necessarily signaling in the cytoplasm rather than via transcriptional regulation in the nucleus. How this cytoplasmic signal arises is not known, but it might derive from the physical interaction of phy4 with phototropin at plasma membrane (Jaedicke et al., 2012). In the present study we identified and characterized a number of novel HIP’s, all of which are at least partly located in the cytoplasm as are their interactions with phy4. Most are conserved in higher plants. Our findings and their possible significance are discussed in more detail below.

In addition to the *Arabidopsis* phyA-FHY1 positive control, most of the HIP’s characterized in this work showed significant R-enhanced, FR-reversible binding in Y2H, indicating that at least the phytochrome-typical R/FR photochromicity and associated structural changes were significant in the growth readout. Notably, no FR-enhanced interaction was seen, in harmony with the established dogma that in plants Pr has no physiological function. phy4–phy4 homodimerization, presumably resulting from interactions between DHp-like domains, was also demonstrated. We therefore consider it likely that the full-length holophytochrome hybrid baits employed in our screens were functional and folded similarly to the native photoreceptor. This is rather surprising in the case of the four candidates identified in the BD:phy4 screen because it is unclear how the presumptive knot around the N-terminal PAS domain (Wagner et al., 2005; Essen et al., 2008) can be formed in the presence of the 17 kDa BD extension. Perhaps the BD and/or the loop through which it passes remain/s largely unfolded even after translation of the GAF domain has begun, allowing the knot to form normally. Alternatively, the extended peptide chain might fold back on itself to form a hairpin which alone protrudes through the loop. It is also possible that the holoprotein remains functional even if knot formation is blocked.

Despite our earlier discovery of the interaction between phy4 and phototropin at the plasma membrane *in planta*, we had quite expected PIF-like proteins to be found in the Y2H screens. This was not the case, however. One explanation might be that, in contrast to fragments, full-length holophytochrome bait is unattractive for such prey in the yeast nucleus. The selection for full-length cDNA’s in the construction of the library we used might also be a significant factor (61% of the prey sequences from the initial screen represented the full-length CDS according to the corresponding gene model). In the case of *Arabidopsis* phyB for example, the interaction seen with the PHL N-terminus was absent for full-length PHL (Endo et al., 2013). Alternatively, PIF-like transcription factors might not be substrates for phy4. However, as different Y2H screens with *Arabidopsis* phyA fragments found different partners, it is not surprising that we also found others for *Physcomitrella* phy4. In any case, even though in the initial screen 10 of the 108 sequences identified were duplicated at least once, the library was not exhaustively screened, so rare phy4 interactors are quite likely to have been missed.

Of the 14 putative HIP’s (full-length CDS gene models showing robust Y2H interaction with phy4 in at least one of the four possible hybrid configurations) only two failed to show split-YFP interaction *in planta*. This high rate of confirmation is probably partly because our Y2H screening protocol exploited mating rather than double transformation, significantly reducing background activation and thus the likelihood of false positives while enhancing sensitivity. We also suspect that our use of functional Pfr as bait in the initial screens increased the number of true interactors found. On the other hand, despite phy4–HIP interaction being apparent in both Y2H and *in planta*, false positives are still possible.

We emphasize that even a proven physical interaction does not necessarily represent a signaling route. Consequently, it is unlikely that all the HIP’s identified in this work are involved in phy4 signaling. None has been studied experimentally and most had not even been annotated. Summarizing our analyses of the HIP sequences:

(1) Of the HIP’s with possible associations with **general signaling** mechanisms, HIP1 is a likely pirin (a protein group suggested to associate phyA with G protein signaling), HIP4 and -5 probably form β-propeller structures and thus, like HIP3, might represent an E3 ubiquitin ligase, HIP7 is a likely signaling protein kinase and HIP8 is probably a Ca^2+^-dependent protein kinase. HIP13 too might be associated with Ca^2+^ signaling.
(2) HIP12 and -13 are likely to be involved in **translation** regulation. This correlates interestingly with phyB regulation of PORA translation by Pfr-dependent interaction with PENTA (Paik et al., 2012). Like HIP3, PENTA is a cytosolic zinc-finger protein.
(3) Various HIP’s might be associated with **posttranslational protein modification.** HIP7, -8, and perhaps -11 are likely Ser/Thr protein kinases, HIP6 is a likely sulfotransferase and HIP9 might represent an endopeptidase.
(4) HIP4, -5, -12, -13, and -14 are associated in various ways with the **cytoskeleton**. This is of particular significance because phy4-mediated vectorial effects (photo- and polarotropism in filament tip cells as well as chloroplast translocation – see Mittmann et al., 2004) are associated with microfilaments and their organization (Meske and Hartmann, 1995; Meske et al., 1996). In particular the 14-3-3 protein HIP14 is interesting in this regard.

These and other characteristics of the various HIP’s identified in this study are hypothetical, providing in many cases no more than hints as to their physiological roles. Obviously, the most appropriate way to clarify their true functions is to knockout the relevant loci by homologous recombination (Schaefer and Zrÿd, 1997) as was successful for phy4 itself (Mittmann et al., 2004). This work has been initiated and we have established different physiological assays to study effects on vectorial responses the results of which will clarify the putative involvement of HIP’s in phy4 signaling.

As discussed in the Section “Introduction,” whereas there is no doubt that the cytoplasmic signal retaining vectorial information derives from phytochromes in lower plants, it is unclear whether a related function exists in higher plants. Similarly, whereas phy4–phot interaction at the plasma membrane has been established and is likely to be associated with vectorial signaling in mosses, the situation in higher plants is less clear. We hope that useful clues regarding the latter and/or the evolution of phytochrome signaling will appear with elucidation of the moss system.

## Author Contributions

AE, KM, and JH designed research and; AE and KM performed experiments. AE and KM analyzed the data, AE and JH wrote and all authors critically read and corrected the manuscript.

## Conflict of Interest Statement

The authors declare that the research was conducted in the absence of any commercial or financial relationships that could be construed as a potential conflict of interest.

## Footnotes

1 www.ncbi.nlm.nih.gov

2 www.cosmoss.org

3 bar.utoronto.ca/efp_physcomitrella/cgi-bin/efpWeb.cgi

4 phytozome.jgi.doe.gov

5 www.genevestigator.com

6 biit.cs.ut.ee/clustvis/

7 www.sbc.su.se/~maccallr/nucpred/

## Abbreviations

AD: GAL4 transcription factor activation domain
AFR: ATTENUATED FAR RED RESPONSE
B: blue
BD: GAL4 transcription factor DNA binding domain
BLAST: basic local alignment search tool
CDPK: calcium-dependent protein kinase
CDS: coding DNA sequence
CFK1: COP9 INTERACTING F-BOX KELCH 1
CFP: cyan fluorescent protein
CHUP1: CHLOROPLAST UNUSUAL POSITIONING
CO: CONSTANS
COP: CONSTITUTIVELY PHOTOMORPHOGENIC
D: dark
DSD: double synthetic dropout
EF1α: translation elongation factor 1α
eIF-5A: eukaryotic translation initiation factor 5A
FBK: F-box/kelch
FHY1: FAR-RED-ELONGATED HYPOCOTYL 1
FKF1: FLAVIN-BINDING KELCH-REPEAT F-BOX 1
FP: fluorescent protein
FR: far-red
FT: FLOWERING LOCUS T (florigen)
GFP: green fluorescent protein
GPA1: G-protein α-subunit
HIP: holophytochrome-interacting protein
HT1: HIGH LEAF TEMPERATURE 1
LKP2: LOV-KELCH PROTEIN 2
NES: nuclear export signal/sequence
NLS: nuclear localization signal/sequence
NPH3: NON-PHOTOTROPIC HYPOCOTYL 3
PCB: phycocyanobilin
Pfr: far-red absorbing form of phytochrome
PHL: PHYTOCHROME-DEPENDENT LATE-FLOWERING
phot: phototropin holoprotein
phy: phytochrome holoprotein
PIF: phytochrome-interacting factor
PKS1: phytochrome kinase substrate 1
PORA: protochlorophyllide oxidoreductase A
PP2A: PROTEIN PHOSPHATASE 2A
Pr: red absorbing form of phytochrome
PRL1: PLEIOTROPIC REGULATORY LOCUS 1
PRN1: pirin 1
QSD: quadruple synthetic dropout
R: Rc/Rp, red, continuous red, pulsed red
RBL: rhomboid-like protein
RFI2: RED AND FAR RED INSENSITIVE 2
RFP: red fluorescent protein
RPT2: ROOT PHOTOTROPISM 2
SCF: SKP1p-Cdc53p (cullin)-RBX1-F-box
SnRK: SNF1-related protein kinase
SOT: sulfotransferase
TSD: triple synthetic dropout
VOZ1: VASCULAR PLANT ONE ZF 1
W: white
Y2H: yeast two-hybrid
YFP, YFP_N_/YFP_C_: yellow fluorescent protein, N-terminal region, C-terminal region
YPDA: yeast peptone dextrose adenine
ZF: zinc finger
