# Supplementary material for: Holophytochrome-Interacting Proteins in Physcomitrella: Putative Actors in Phytochrome Cytoplasmic Signaling
Source: Front Plant Sci. 2016 May 12;7:613. doi: 10.3389/fpls.2016.00613 (PMC4867686; doi:10.3389/fpls.2016.00613)
Supplement: Supplementary file 1 [file Data_Sheet_1.PDF]

Summary Table 1: Summary of HIP 1-14 *in silico* and experimental analyses including Cosmoss accession numbers, amino acid sequence length, protein sequence similarities in *Physcomitrella* and *Arabidopsis*, domains, interaction behaviour in Y2H, *in planta* localization, location of phy4 interaction & light effects, and hypothetical function.

HIP Nr. / Description	Cosmoss accession	Protein length [residues] / domains [spanning region]	*Physcomitrella* homologs	Putative *Arabidopsis* homologs	Y2H interaction behavior	Localization (N- / C-terminal FP-fusion)	Split-YFP pattern	R-dependent *in planta* interaction	Hypothetical function
HIP1 / Pirin	Pp3c2_0320 V1.1	352 / Pirin (61-350)<br>RmlC-like jelly roll fold (63-308)<br>RmlC-like cupin (65-344)<br>Pirin, N-terminal (83-177)<br>Pirin, C-terminal (230-335)	3 pirin-like (incl. HIP1):<br>Pp3c19_20830V1.1<br>Pp3c21_16130	4 pirins / pirin-like proteins:<br>AT3G59220 Pirin 1,<br>AT2G43120 pirin-like protein<br>AT1G50590 pirin-like protein<br>AT3G59260 pirin, putative	Constitutive or R/FR-reversible	Cytoplasm + nucleus	Cytoplasm & nucleus (occasional cytoplasmic foci)	No	Signal transduction, quercetinase activity, G-protein signaling
Pirin-like protein	Pp3c19_20830_V1.1	297 / Pirin (17-294)<br>RmlC-like jelly roll fold (59-294)<br>RmlC-like cupin (65-271)<br>Pirin, N-terminal (64-167)	See HIP1	see HIP1	R-enhanced, FR-reversible	Cytoplasm / cytoplasm + nucleus	Not verified	No	see HIP1
HIP3 / ZF-protein	Pp3c10_4820 V1.1	329 / CHY-type ZF (68-156)<br>CTCHY ZF (146-210)<br>RING-type ZF (210-254)<br>C3HC4 ZF (210-253)<br>RING/FYVE/PHD-type ZF (206-270)<br>rubredoxin-type fold (282-319)	5-7 (incl. HIP3):<br>Pp3c14_5600V1.1<br>Pp3c11_6970V1.1<br>Pp3c1_6070V1.1,<br>Pp3c2_31820V1.1<br>(Pp3c25_14500V1.1<br>Pp3c6_1960V1.1)	AT5G22920 RING finger and CHY ZF-domain-containing protein 1<br>AT5G25560 CHY and CTCHY and RING-type ZF protein<br>AT5G18650 MYB30-interacting E3 ligase<br>AT3G62970, AT3G18290, AT1G74770, AT1G18910	Weak but R-enhanced, FR-reversible	Cytoplasm or cytoplasm + nucleus / cytoplasm + nucleus	Cytoplasm + nucleus	Yes	Signal transduction protein interaction protein degradation ?
HIP4 / PRL1	Pp3c16_8560 E1.1	495 / WD40 / YVTN repeat-like-containing (164-471)<br>WD40-repeat-containing (169-480)<br>7x WD40 (173-212, 215-254, 257-296, 299-338, 341-379, 382-421 & 433-471)<br>G-protein beta WD-40 repeat (199-213, 283-297, 325-339)	2 (incl. HIP4),<br>Pp3c5_15520V1.1<br><br>many others with WD40 domains	AT4G15900 PRL1<br>AT3G16650 PRL2	Constitutive or R/FR-reversible	Nucleus / cytoplasm + nucleus	Cytoplasm or cytoplasm + nucleus	No	Signal transduction protein binding cytosceleton ?
HIP5 / Kelch repeat protein	Pp3c7_3040 V1.1	498 / galactose oxidase / kelch, beta-propeller (163-474)<br>Kelch-type beta propeller (198-483)<br>Kelch-related protein (203-373)<br>Kelch repeat type 1 (248-292, 280-326, 292-339, 327-374 & 339-388)<br>Kelch repeat type 2 (383-424)	2 (incl. HIP5);<br>Pp3c11_19970V1.1<br><br>22 others with lower similarities from res. 140	Many (highest similarity in kelch region only):<br>AT1G14330 galactose oxidase/ kelch repeat superfamily protein<br>AT1G26930 F-box/kelch repeat protein<br>AT5G60570 F-box/kelch repeat protein<br>AT3G27150 MIR2111-5p target protein<br>AT2G02870 F-box/kelch repeat protein SKIP11 + *etc.*	R-enhanced, FR-reversible	Cytoplasm + nucleus	Cytoplasm or cytoplasm + nucleus	No	Signal transduction protein binding protein degradation/ cytosceleton ?
HIP6 / P-loop protein	Pp3c6_4480 V1.1	379 / P-loop-containing nucleoside triphosphate hydrolase (123-358), sulfotransferase (134-337)	4 (incl. HIP6):<br>Pp3c5_24550V1.1<br>Pp3c25_12260V1.1<br>Pp3c16_22670V1.1	22 sulfotransferases (Hirschmann *et al.* 2014)<br>3 close homologs:<br>AT3G50620 AtSOT19<br>AT2G15730 AtSOT20<br>AT4G34420 AtSOT21<br>+ 2 hypothetical proteins / variants (CAB62491.1 & CAB36719.1)	Mostly constitutive	Cytoplasm / cytoplasm + cytoplasmic foci	Cytoplasm + cytoplasmic foci or cytoplasm (+ nucleus)	No	Signal transduction nucleoside triphosphate hydrolase sulfo-transferase
HIP7 / Ser/thr protein	Pp3c17_9390 V1.1	287 / protein kinase, catalytic domain (1-287 & 221-235)<br>serine / threonine- / dual specificity protein kinase, catalytic (37-273)<br>protein kinase-like domain (122-227)	Single copy<br><br>Many with weak similarity res. 100-220	Similarity to various *Arabidopsis* / viridiplantae proteins<br>e.g. S/T protein kinase HIGH LEAF TEMPERATURE 1 (HT1; AT1G62400)	R-enhanced	Cytoplasm + nucleus (+ cytoplasmic foci) / cytoplasm + nucleus	Cytoplasm + nucleus	Partially	Signal transduction protein phosphorylation
HIP8 / CDPK	Pp3c11_25550 V1.1	497 / protein kinase-like (31-302)<br>protein kinase (31-290)<br>S/T dual specificity protein kinase, catalytic (31-290)<br>4x Ca^{2+}-binding EF-hand (333-368, 369-404, 405-440 & 443-474)<br>EF-hand domain pair (330-475)	25 CDPKs (Hamel *et al.*, 2014)<br><br>~33 (incl. HIP8)	CPK1 and 33 other CDPKs<br>(Hamel *et al.*, 2014)	Weak but R-enhanced, FR-reversible	Cytoplasm + nucleus	Cytoplasm or cytoplasm + nucleus	No, perhaps R-induced cytoplasmic translocation	Ca^{2+}-signal transduction protein phosphorylation, cytosceleton ?
HIP9 / Rhomboid peptidase	Pp1s254_10 V6.2	580 / Peptidase S54, rhomboid superfamily (4-560)<br>Peptidase S54, rhomboid (369-507 & 323-506)	16 rhomboid-like (Li *et al.* 2015)<br>1 close homolog:<br>Pp3c22_8560V1.1<br>3 less similar:<br>Pp3c2_10440V1.1<br>Pp3c1_43050V1.1<br>Pp3c17_14100V1.1	17 RBL's, closest: At5g38510 (RBL9)	R-enhanced, FR-reversible	Cytoplasm (maybe plasma membrane) / cytoplasm + nucleus	Cytoplasm + nucleus or plasma membrane + nucleus	Yes	Membrane protein, maybe intra-membrane proteolysis or other enzymatic activity
Ankyrin- & BTB/POZ	Pp3c1_11190 C1.1	360 / ankyrin repeat-containing protein (33-99, 31-101, 36-156 & 36-103)<br>Ankyrin-repeat (31-61, 65-101)<br>SKP1/BTB/POZ (190-296)<br>BTB/POZ (202-300)	2 (incl. Pp3c1_11190):<br>Pp3c7_8840V1.1<br>7 less similar (res. 180-350)	Weak similarity to BTB/TAZ domain protein AT2G30600 (340-480)<br>Best similarity to *Selaginella moellendorffii* Selmo1_441307 & Selmo1_440857	Constitutive or R-enhanced	Cytoplasmic patches / cytoplasm + nucleus	Not verified	No	Signal transduction cytosceleton
HIP11 / unknown protein	Pp3c20_16210 V1.1	189 / transmembrane region (7-25)	Identical to Pp3c20_16230V1.1	Weak similarity to pfkB-like carbohydrate kinase AT5G19150	R-enhanced, mostly FR-reversible	Cytoplasm + perinuclear region (+ nucleus) / cytoplasm (+ nucleus)	Cytoplasm + perinuclear region	No	Possible signal transduction & phosphorylation
HIP12 / eIF-5A	Pp3c13_15620 V1.1	162 / translation elongation factor IF5A (1-159)<br>Ribosomal protein L2 domain 2 (15-84)<br>Translation protein SH3-like (15-93)<br>Nucleic acid binding, OB fold (86-157)<br>IF5A C-terminal (85-154)	2 (including HIP12)<br>Pp3c12_3540V1.1	3 isoforms of eIF-5A:<br>AT1G13950 eIF-5A<br>AT1G26630 eIF-5A-2<br>AT1G69410 eIF-5A-3	Constitutive or R/FR-reversible	Cytoplasm + nucleus	Cytoplasm or cytoplasm + nucleus	No	Translation, cytoskeleton ?
HIP13 / EF1α	Pp3c1_23670 V1.1	448 / translation elongation factor EF1A, eukaryotic / archaeal (1-433)<br>p-loop containing nucleoside triphosphate hydrolase (3-234 & 3-249)<br>TF, GTP-binding (5-230),<br>Translation protein β-barrel (231-322)<br>EFTu/EF1A, domain 2 (248-313)<br>EF1A/IFγ, C-terminal (326-429)<br>EFTu/EF1A, C-terminal (322-429)	11 (incl. HIP12)<br>Pp3c2_6160V1.1<br>Pp3c2_6690V1.1<br>Pp3c1_23750V1.1<br>Pp3c5_10430V1.1<br>Pp3c2_10310V1.1<br>Pp3c2_6650V1.1<br>Pp3c2_6770V1.1<br>Pp3c1_23850V1.1<br>Pp3c1_23900V1.1<br>Pp3c1_23920V1.1<br>16-23 less similar	AT1G07940 EF 1-alpha 1<br>AT1G07930 EF 1-alpha 2<br>AT1G07920 EF 1-alpha 3<br>AT5G60390 EF 1-alpha 4<br>other closer homologs:<br>AT1G18070 EF1A / IF2-gamma family protein<br>AT5G10630 putative EF 1-A / eIF-2-gamma<br>AT1G35550 EF Tu C-terminal domain-containing protein<br>AT4G02930 putative EF Tu<br>AT4G20360 RAB GTPase homolog E1b	Constitutive or R/FR-reversible	Cytoplasm + perinuclear region	Cytoplasm + perinuclear region or cytoplasm + nucleus	No	Translation cytoskeleton maybe Ca^{2+}-signaling
HIP14 / 14-3-3 protein	Pp3c3_8540 C1.1	258 / 14-3-3 protein (1-256)<br>14-3-3 (5-237, 6-239 & 5-248)	11 (incl. HIP14)<br>Pp3c10_20300V1.1<br>Pp3c12_18760V1.1<br>Pp3c4_20750V1.1<br>Pp3c10_14160V1.1<br>Pp3c10_14250V1.1<br>Pp3c3_8510V1.1<br>Pp3c23_10780V1.1<br>Pp3c23_11080V1.1<br>Pp3c24_7310V1.1<br>Pp3c24_7780V1.1	13 14-3-3-like proteins:<br>AT5G38480 GF14 psi<br>AT1G78300 GF14 omega<br>AT4G09000 GF14 chi<br>AT1G35160 GF14 phi<br>AT3G02520 GF14 nu<br>AT5G16050 GF14 upsilon<br>AT5G10450 GF14 lambda<br>AT5G65430 GF14 kappa<br>AT1G26480 GF4 iota<br>AT1G34760 GF14 omicron<br>AT2G42590 GF14 mu<br>AT1G22300 GF14 epsilon<br>AT1G22290 GF14 xi<br>AT2G10450 14-3-3 family protein<br>AT1G78220 general regulatory factor 13	Constitutive	Cytoplasm + nucleus / cytoplasm + perinuclear region (cyto-skeleton)	Cytoplasm (cyto-skeleton?) or cytoplasm + nucleus	No	Signal transduction protein interaction phospho-sensing
