# Supplementary material for: Holophytochrome-Interacting Proteins in Physcomitrella: Putative Actors in Phytochrome Cytoplasmic Signaling
Source: Front Plant Sci. 2016 May 12;7:613. doi: 10.3389/fpls.2016.00613 (PMC4867686; doi:10.3389/fpls.2016.00613)
Supplement: Supplementary file 2 [file Data_Sheet_2.ZIP › SI/SI (HIP10).pdf]

*Supplementary Material*

# Holophytochrome-interacting proteins in *Physcomitrella*: putative actors in phytochrome cytoplasmic signaling

**Anna Lena Ermert, Katharina Mailliet, and Jon Hughes***

*** Correspondence:** jon.hughes@uni-giessen.de

## (HIP10) Pp3c1_11190C1.1

```
ATGGCTGCCGAGAAGAGCCACGCTGTGGATGAGGTTACTGCTGAGGTCGAAGCCGTGGGACTTGCGGAGGACCCCGGTAGG
AACTTGAAGCTGGACCAAGCGACTTTCTTCCGGTGTGTGGAGAATGGTAACCTTTCAGCTCTAACAGCTTTGCTTAAGAAC
CGTAAGGTGGACATCAACGCATACAATGATGAGGGCGTGACGTGTCTGCACGTTGCATTGTACAAGTATGAGGAGTCGCGG
AGTCTGGACATGGTGAAGTTCCTACTTGCACATGGCGCTAATATCTCACTCAAGGCAGCTGTGATGCCATCTGCGCACAAA
ATTTCTATTATCCGCCACGGTCACGGTGGCAGTGTCGCCAATCCACTCGAAACCAAGAAGGTTGTTTTTGATCAGAAGACT
CCTCTTCTTGTGGCGCTGGAGCTGAAATCGGCGCTTTACCTGAAAGGGTGGGAGTACCGGCATTGGGACGAGATGTTGAGG
ATTTTAGCAGACGCAACCATTGAGCACCACGCTAAATCTCCTGAACTAAAGGGAGAAAGGCTTCCTTACATTGTGATTCAG
AAGAATTGGGGTAATGTGTTCGCAAGTGGGAAACATGAGACGATTGAAGTGTGGGCAGAGGACAAGAGTATTACTGTGCTG
AAGCTCCTCCTTACTGGCGCATCAAAGATTCTTAAACTCAATATCGAGAATCCAGACGTACAATATTCGAATAGATTGGAC
ATCAAGGAGGCGTCGTTTAATATTACCAAGGCCATGATGAAGTTTCTTTACACTGGAAAGGTTGAAGCCGAGTTTCTTGAA
CACAGGGGTCTAGATCTCCTATCTGCGGCTCACAAGTATGGAATCAAGAGCTTGATGTGGGTTTGTGAGGATTCAATTCGT
GCTACACCGGATAATTGGATCAAGCTTCTATCCACGGCCATGGAGTGCAACTCAGATATGCTAGCTCTCAAATGTGCTCAA
TCGATAAAGGAAGTGATGGACGAACGACATGAAAAACACACCAGTTTGAAGAAGAGCTATTCAAATATGCACAATGTCCCT
AACCAACTCTTCACTTCATTTCCGTGA
```

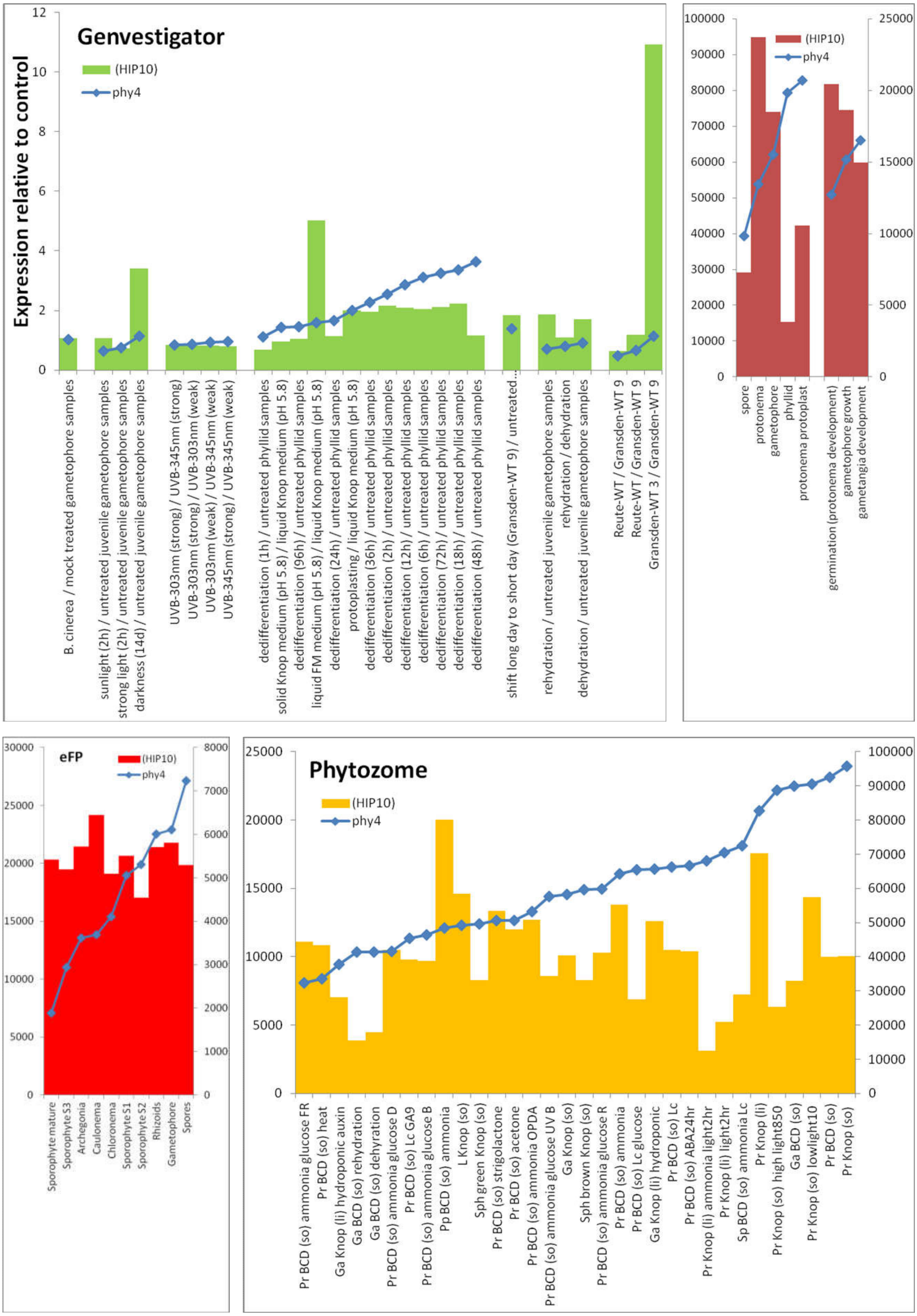
Genvestigator
Expression relative to control
(HIP10)
phy4
B. cinerea / mock treated gametophore samples
sunlight (2h) / untreated juvenile gametophore samples
strong light (2h) / untreated juvenile gametophore samples
darkness (14d) / untreated juvenile gametophore samples
UVB-303nm (strong) / UVB-345nm (strong)
UVB-303nm (strong) / UVB-303nm (weak)
UVB-303nm (weak) / UVB-345nm (weak)
UVB-345nm (strong) / UVB-345nm (weak)
dedifferentiation (1h) / untreated phyllid samples
solid Knop medium (pH 5.8) / liquid Knop medium (pH 5.8)
dedifferentiation (96h) / untreated phyllid samples
liquid FM medium (pH 5.8) / liquid Knop medium (pH 5.8)
dedifferentiation (24h) / untreated phyllid samples
protoplasting / liquid Knop medium (pH 5.8)
dedifferentiation (36h) / untreated phyllid samples
dedifferentiation (2h) / untreated phyllid samples
dedifferentiation (12h) / untreated phyllid samples
dedifferentiation (6h) / untreated phyllid samples
dedifferentiation (72h) / untreated phyllid samples
dedifferentiation (18h) / untreated phyllid samples
dedifferentiation (48h) / untreated phyllid samples
shift long day to short day (Gransden-WT 9) / untreated...
rehydration / untreated juvenile gametophore samples
rehydration / dehydration
dehydration / untreated juvenile gametophore samples
Reute-WT / Gransden-WT 9
Reute-WT / Gransden-WT 9
Gransden-WT 3 / Gransden-WT 9
(HIP10)
phy4
spore
protonema
gametophore
phyllid
protonema protoplast
germination (protonema development)
gametophore growth
gametangia development
eFP
(HIP10)
phy4
Sporophyte mature
Sporophyte S3
Archegonia
Caulonema
Chloronema
Sporophyte S1
Sporophyte S2
Rhizoids
Gametophore
Spores
Phytozome
(HIP10)
phy4
Pr BCD (so) ammonia glucose FR
Pr BCD (so) heat
Ga Knop (li) hydroponic auxin
Ga BCD (so) rehydration
Ga BCD (so) dehyration
Pr BCD (so) ammonia glucose D
Pr BCD (so) Lc GA9
Pr BCD (so) ammonia glucose B
Pp BCD (so) ammonia
L Knop (so)
Sph green Knop (so)
Pr BCD (so) strigolactone
Pr BCD (so) acetone
Pr BCD (so) ammonia OPDA
Pr BCD (so) ammonia glucose UV B
Ga Knop (so)
Sph brown Knop (so)
Pr BCD (so) ammonia glucose R
Pr BCD (so) ammonia
Pr BCD (so) Lc glucose
Ga Knop (li) hydroponic
Pr BCD (so) Lc
Pr BCD (so) ABA24hr
Pr Knop (li) ammonia light2hr
Pr Knop (li) light2hr
Sp BCD (so) ammonia Lc
Pr Knop (li)
Pr Knop (so) high light850
Ga BCD (so)
Pr Knop (so) lowlight10
Pr BCD (so)
Pr Knop (so)

# Pp3c1_11190C1.1 (formerly HIP10) alignment tree

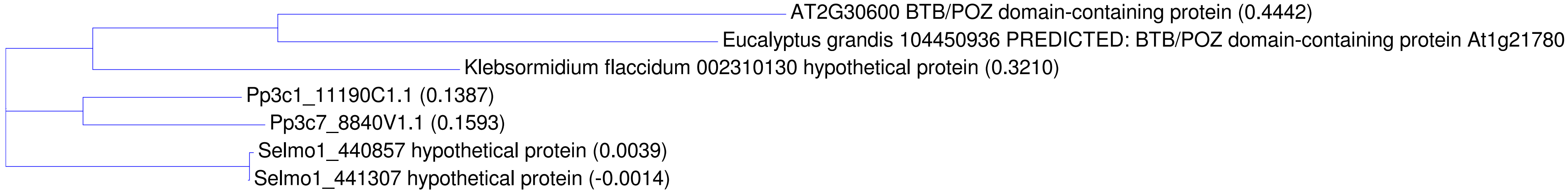

# Pp3c1_11190C1.1 (formerly HIP10) alignment

```
                                                       (1)  1       10                  20                  30                  40                  50                  62
AT2G30600 BTB/POZ domain-containing protein            (1)  MVAAKENKFLTVAPFECAWSDDLKFREAGRGCVAFDAFAHNDVTVVFRENVGTQHYHYKKDN
Eucalyptus grandis PREDICTED: BTB/POZ protein At1g21780 (1) --------------------------------------------------------------
Klebsormidium flaccidum 002310130 hypothetical protein (1)  --------------------------------------------------------------
Pp3c1_11190C1.1                                        (1)  --------------------------------------------------------------
Pp3c7_8840V1.1                                         (1)  --------------------------------------------------------------
Selmo1_440857 hypothetical protein                     (1)  --------------------------------------------------------------
Selmo1_441307 hypothetical protein                     (1)  --------------------------------------------------------------
Consensus                                              (1)

                                                       (63) 63      70                  80                  90                  100                 110                 124
AT2G30600 BTB/POZ domain-containing protein            (63) SPHYIVIIGSNRNRRLKIQVDGKSVVDEEASDLCRCSLEFQSYWISIYDGLISIGKGRYPFQ
Eucalyptus grandis PREDICTED: BTB/POZ protein At1g21780 (1) --------------------------------------------------------------
Klebsormidium flaccidum 002310130 hypothetical protein (1)  -------------------------------------------------------------M
Pp3c1_11190C1.1                                        (1)  --------------------------------------------------------------
Pp3c7_8840V1.1                                         (1)  --------------------------------------------------------------
Selmo1_440857 hypothetical protein                     (1)  ------MADERLEPVPRLLEGDQALPIADPQELPPKLESLESVDLSGSLGKESLLSAGAAAS
Selmo1_441307 hypothetical protein                     (1)  ------MADERLEPVPRLLEGDQALPIADPQELPPKLESLESVDLSGSLGKESLLSAGAAAS
Consensus                                              (63)

                                                                                                              ankyrin-repeat
                                                       (125) 125  130                 140                 150                 160                 170                 186
AT2G30600 BTB/POZ domain-containing protein            (125) NLVFKWQDPKPNCNVQYVGLSSWDKHVGYRNVSVFPVTHNHILLWKQVDCREVRGDESGDEK
Eucalyptus grandis PREDICTED: BTB/POZ protein At1g21780 (1)  ---MADSKVETLSRLAQWRIDGFGPSSCRKSEPFKMGIWNWHLLVE-----------RNRYL
Klebsormidium flaccidum 002310130 hypothetical protein (2)   AEENSAAEALESMHISHPGTDNAGAAGQE--KEKELNQATFFHCVEAQNAKTLQILTRTRQV
Pp3c1_11190C1.1                                        (1)   ----MAAEKSHAVDEVTAEVEAVGLAEDPG-RNLKLDQATFFRCVENGNLSALTALLKNRKV
Pp3c7_8840V1.1                                         (1)   ---MAAETDNVTVAEVRTELGAVGLADDAG-QNLVLDQATFFRCVENGNLSVLSALLNNRKV
Selmo1_440857 hypothetical protein                     (57)  SPRLSASAASPRLDQVKPSVEVVGLSDDAG-HDLRLDQATFFRCVEDGNVTALEGLLKNRKL
Selmo1_441307 hypothetical protein                     (57)  SPRSSASAASPRLDQVKPSVEVVGLSDDAG-HDLRLDQATFFRCVEDGNVTALEGLLKNRKL
Consensus                                              (125)     SAS     L  V   VE VGLADD G   LKLDQATFFRCVE GNLS L GLLKNRKL
                                                                                                 Ankyrin-repeat-containing protein
```

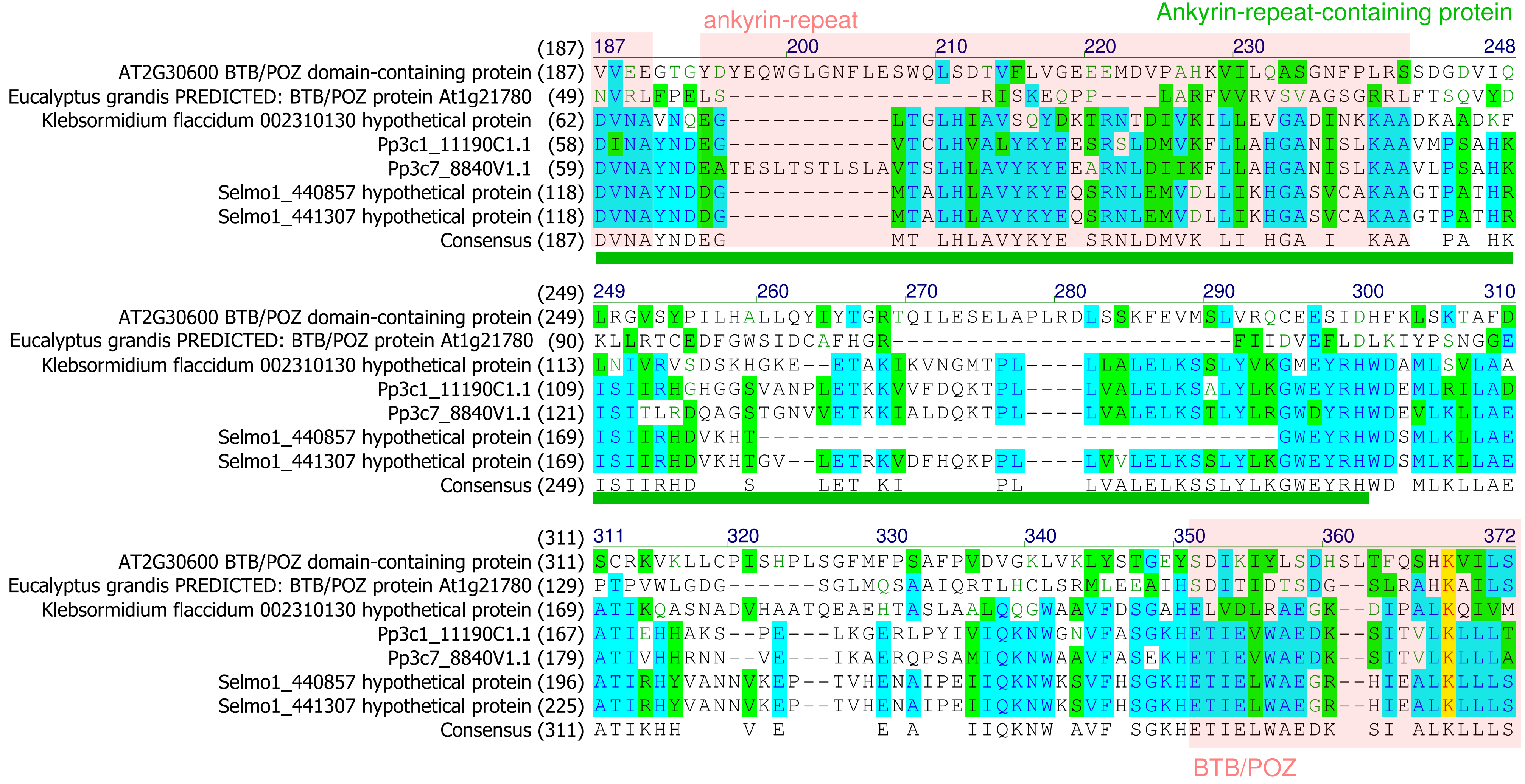
ankyrin-repeat
Ankyrin-repeat-containing protein
(187) 187 200 210 220 230 248
AT2G30600 BTB/POZ domain-containing protein (187) VVEEGTGYDYEQWGLGNFLESWQLSDTVFLVGEEEMDVPAHKVILQASGNFPLRSSDGDVIQ
Eucalyptus grandis PREDICTED: BTB/POZ protein At1g21780 (49) NVRLFPELS-----------------RISKEQPP----LARFVVRVSVAGSGRRLFTSQVYD
Klebsormidium flaccidum 002310130 hypothetical protein (62) DVNAVNQEG-----------LTGLHIAVSQYDKTRNTDIVKILLEVGADINKKAADKAADKF
Pp3c1_11190C1.1 (58) DINAYNDEG-----------VTCLHVALYKYEESRSLDMVKFLLAHGANISLKAAVMPSAHK
Pp3c7_8840V1.1 (59) DVNAYNDEATESLTSTLSLAVTSLHLAVYKYEEARNLDIIKFLLAHGANISLKAAVLPSAHK
Selmo1_440857 hypothetical protein (118) DVNAYNDDG-----------MTALHLAVYKYEQSRNLEMVDLLIKHGASVCAKAAGTPATHR
Selmo1_441307 hypothetical protein (118) DVNAYNDDG-----------MTALHLAVYKYEQSRNLEMVDLLIKHGASVCAKAAGTPATHR
Consensus (187) DVNAYNDEG MT LHLAVYKYE SRNLDMVK LI HGA I KAA PA HK
(249) 249 260 270 280 290 300 310
AT2G30600 BTB/POZ domain-containing protein (249) LRGVSYPILHALLQYIYTGRTQILESELAPLRDLSSKFEVMSLVRQCEESIDHFKLSKTAFD
Eucalyptus grandis PREDICTED: BTB/POZ protein At1g21780 (90) KLLRTCEDFGWSIDCAFHGR-----------------------FIIDVEFLDLKIYPSNGGE
Klebsormidium flaccidum 002310130 hypothetical protein (113) LNIVRVSDSKHGKE--ETAKIKVNGMTPL----LLALELKSSLYVKGMEYRHWDAMLSVLAA
Pp3c1_11190C1.1 (109) ISIIRHGHGGSVANPLETKKVVFDQKTPL----LVALELKSALYLKGWEYRHWDEMLRILAD
Pp3c7_8840V1.1 (121) ISITLRDQAGSTGNVVETKKIALDQKTPL----LVALELKSTLYLRGWDYRHWDEVLKLLAE
Selmo1_440857 hypothetical protein (169) ISIIRHDVKHT--------------------------------GWEYRHWDSMLKLLAE
Selmo1_441307 hypothetical protein (169) ISIIRHDVKHTGV--LETRKVDFHQKPPL----LVVLELKSSLYLKGWEYRHWDSMLKLLAE
Consensus (249) ISIIRHD S LET KI PL LVALELKSSLYLKGWEYRHWD MLKLLAE
(311) 311 320 330 340 350 360 372
AT2G30600 BTB/POZ domain-containing protein (311) SCRKVKLLCPISHPLSGFMFPSAFPVDVGKLVKLYSTGEYSDIKIYLSDHSLTFQSHKVILS
Eucalyptus grandis PREDICTED: BTB/POZ protein At1g21780 (129) PTPVWLGDG------SGLMQSAAIQRTLHCLSRMLEEAIHSDITIDTSDG--SLRAHKAILS
Klebsormidium flaccidum 002310130 hypothetical protein (169) ATIKQASNADVHAATQEAEHTASLAALQQGWAAVFDSGAHELVDLRAEGK--DIPALKQIVM
Pp3c1_11190C1.1 (167) ATIEHHAKS--PE---LKGERLPYIVIQKNWGNVFASGKHETIEVWAEDK--SITVLKLLLT
Pp3c7_8840V1.1 (179) ATIVHHRNN--VE---IKAERQPSAMIQKNWAAVFASEKHETIEVWAEDK--SITVLKLLLA
Selmo1_440857 hypothetical protein (196) ATIRHYVANNVKEP--TVHENAIPEIIQKNWKSVFHSGKHETIELWAEGR--HIEALKLLLS
Selmo1_441307 hypothetical protein (225) ATIRHYVANNVKEP--TVHENAIPEIIQKNWKSVFHSGKHETIELWAEGR--HIEALKLLLS
Consensus (311) ATIKHH V E E A IIQKNW AVF SGKHETIELWAEDK SI ALKLLLS
BTB/POZ

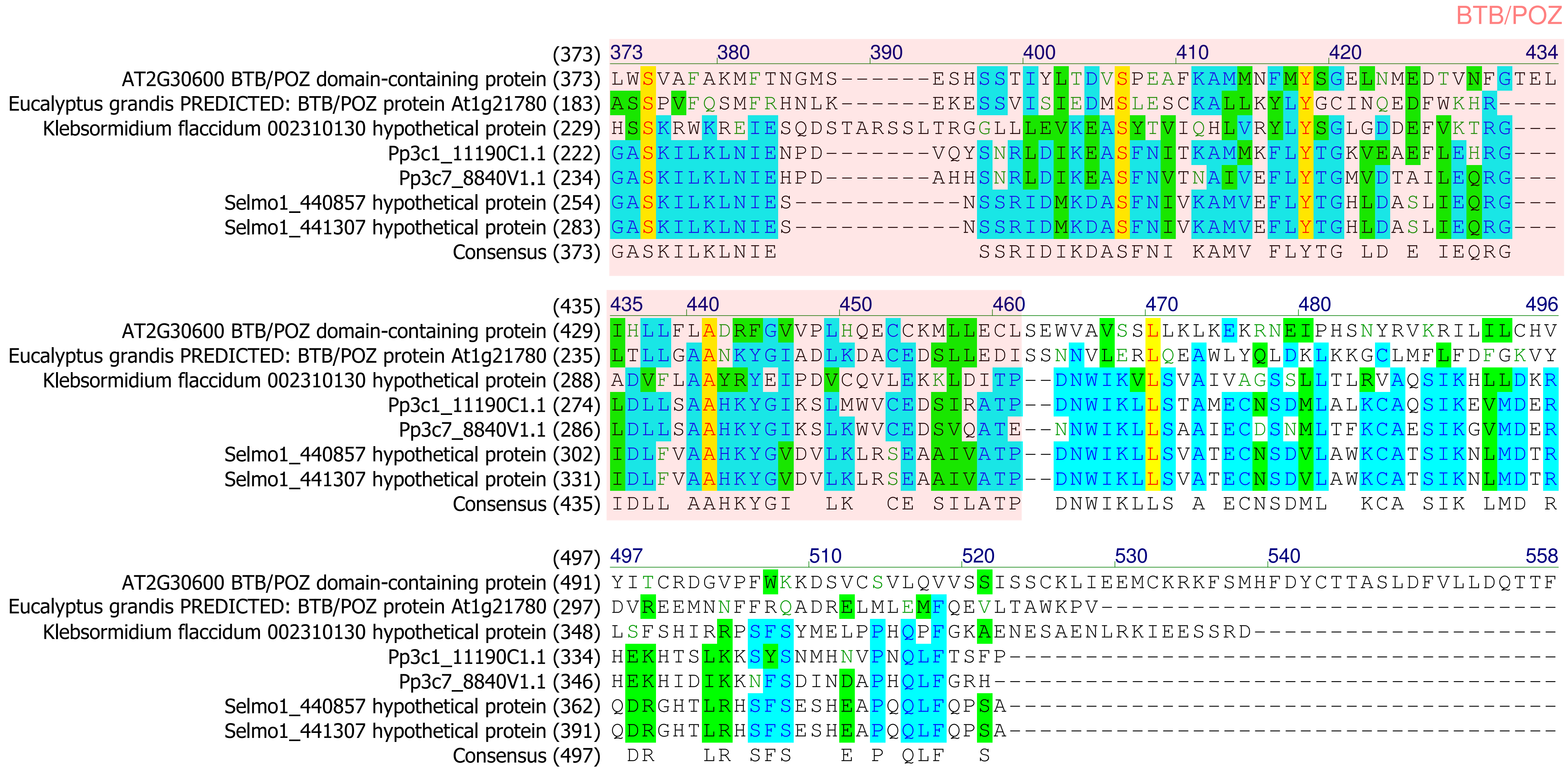
BTB/POZ
(373) 373 380 390 400 410 420 434
AT2G30600 BTB/POZ domain-containing protein (373) LWSVAFAKMFTNGMS------ESHSSTIYLTDVSPEAFKAMMNFMYSGELNMEDTVNFGTEL
Eucalyptus grandis PREDICTED: BTB/POZ protein At1g21780 (183) ASSPVFQSMFRHNLK------EKESSVISIEDMSLESCKALLKYLYGCINQEDFWKHR----
Klebsormidium flaccidum 002310130 hypothetical protein (229) HSSKRWKREIESQDSTARSSLTRGGLLLEVKEASYTVIQHLVRYLYSGLGDDEFVKTRG---
Pp3c1_11190C1.1 (222) GASKILKLNIENPD-------VQYSNRLDIKEASFNITKAMMKFLYTGKVEAEFLEHRG---
Pp3c7_8840V1.1 (234) GASKILKLNIEHPD-------AHHSNRLDIKEASFNVTNAIVEFLYTGMVDTAILEQRG---
Selmo1_440857 hypothetical protein (254) GASKILKLNIES-----------NSSRIDMKDASFNIVKAMVEFLYTGHLDASLIEQRG---
Selmo1_441307 hypothetical protein (283) GASKILKLNIES-----------NSSRIDMKDASFNIVKAMVEFLYTGHLDASLIEQRG---
Consensus (373) GASKILKLNIE SSRIDIKDASFNI KAMV FLYTG LD E IEQRG
(435) 435 440 450 460 470 480 496
AT2G30600 BTB/POZ domain-containing protein (429) IHLLFLADRFGVVPLHQECCKMLLECLSEWVAVSSLLKLKEKRNEIPHSNYRVKRILILCHV
Eucalyptus grandis PREDICTED: BTB/POZ protein At1g21780 (235) LTLLGAANKYGIADLKDACEDSLLEDISSNNVLERLQEAWLYQLDKLKKGCLMFLFDFGKVY
Klebsormidium flaccidum 002310130 hypothetical protein (288) ADVFLAAYRYEIPDVCQVLEKKLDITP--DNWIKVLSVAIVAGSSLLTLRVAQSIKHLLDKR
Pp3c1_11190C1.1 (274) LDLLSAAHKYGIKSLMWVCEDSIRATP--DNWIKLLSTAMECNSDMLALKCAQSIKEVMDER
Pp3c7_8840V1.1 (286) LDLLSAAHKYGIKSLKWVCEDSVQATE--NNWIKLLSAAIECDSNMLTFKCAESIKGVMDER
Selmo1_440857 hypothetical protein (302) IDLFVAAHKYGVDVLKLRSEAAIVATP--DNWIKLLSVATECNSDVLAWKCATSIKNLMDTR
Selmo1_441307 hypothetical protein (331) IDLFVAAHKYGVDVLKLRSEAAIVATP--DNWIKLLSVATECNSDVLAWKCATSIKNLMDTR
Consensus (435) IDLL AAHKYGI LK CE SILATP DNWIKLLS A ECNSDML KCA SIK LMD R
(497) 497 510 520 530 540 558
AT2G30600 BTB/POZ domain-containing protein (491) YITCRDGVPFWKKDSVCSVLQVVSSISSCKLIEEMCKRKFSMHFDYCTTASLDFVLLDQTTF
Eucalyptus grandis PREDICTED: BTB/POZ protein At1g21780 (297) DVREEMNNFFRQADRELMLEMFQEVLTAWKPV------------------------------
Klebsormidium flaccidum 002310130 hypothetical protein (348) LSFSHIRRPSFSYMELPPHQPFGKAENESAENLRKIEESSRD--------------------
Pp3c1_11190C1.1 (334) HEKHTSLKKSYSNMHNVPNQLFTSFP------------------------------------
Pp3c7_8840V1.1 (346) HEKHIDIKKNFSDINDAPHQLFGRH-------------------------------------
Selmo1_440857 hypothetical protein (362) QDRGHTLRHSFSESHEAPQQLFQPSA------------------------------------
Selmo1_441307 hypothetical protein (391) QDRGHTLRHSFSESHEAPQQLFQPSA------------------------------------
Consensus (497) DR LR SFS E P QLF S

```
                                                        (559) 559       570       580       590       600       610       620
          AT2G30600 BTB/POZ domain-containing protein (553) SDILESADLTVTSEEKILNAVLMWCMKAEESHSWGVIDEMMNYADPKSLFKERLQSLDDLLP
Eucalyptus grandis PREDICTED: BTB/POZ protein At1g21780 (329) --------------------------------------------------------------
  Klebsormidium flaccidum 002310130 hypothetical protein (390) --------------------------------------------------------------
                                         Pp3c1_11190C1.1 (360) --------------------------------------------------------------
                                          Pp3c7_8840V1.1 (371) --------------------------------------------------------------
                   Selmo1_440857 hypothetical protein (388) --------------------------------------------------------------
                   Selmo1_441307 hypothetical protein (417) --------------------------------------------------------------
                                               Consensus (559)

                                                        (621) 621      630       640       650       660       670         682
          AT2G30600 BTB/POZ domain-containing protein (615) HVRFSLLPYELLKRLENSNLSKEIPVFNRLLKEAASFLTSGLISPGNEPISRFQHRRSSFKE
Eucalyptus grandis PREDICTED: BTB/POZ protein At1g21780 (329) --------------------------------------------------------------
  Klebsormidium flaccidum 002310130 hypothetical protein (390) --------------------------------------------------------------
                                         Pp3c1_11190C1.1 (360) --------------------------------------------------------------
                                          Pp3c7_8840V1.1 (371) --------------------------------------------------------------
                   Selmo1_440857 hypothetical protein (388) --------------------------------------------------------------
                   Selmo1_441307 hypothetical protein (417) --------------------------------------------------------------
                                               Consensus (621)

                                                        (683) 683    690       700       710       720       730           744
          AT2G30600 BTB/POZ domain-containing protein (677) LQYIRDGDSNGVLHFVGTSYGSHQWVNPVLAKKINITSSSPTSRFTDPKALASKAYAGTSFA
Eucalyptus grandis PREDICTED: BTB/POZ protein At1g21780 (329) --------------------------------------------------------------
  Klebsormidium flaccidum 002310130 hypothetical protein (390) --------------------------------------------------------------
                                         Pp3c1_11190C1.1 (360) --------------------------------------------------------------
                                          Pp3c7_8840V1.1 (371) --------------------------------------------------------------
                   Selmo1_440857 hypothetical protein (388) --------------------------------------------------------------
                   Selmo1_441307 hypothetical protein (417) --------------------------------------------------------------
                                               Consensus (683)
```

```
                                                      (745) 745  750       760       770       780       790                806
AT2G30600 BTB/POZ domain-containing protein           (739) GPRMEDGHISSWWVVDLGEEHQLMCNYYTFRQDGSRAFTRFWKFQGSMDGKTWTDLRVHEDD
Eucalyptus grandis PREDICTED: BTB/POZ protein At1g21780 (329) --------------------------------------------------------------
Klebsormidium flaccidum 002310130 hypothetical protein (390) --------------------------------------------------------------
Pp3c1_11190C1.1                                       (360) --------------------------------------------------------------
Pp3c7_8840V1.1                                        (371) --------------------------------------------------------------
Selmo1_440857 hypothetical protein                    (388) --------------------------------------------------------------
Selmo1_441307 hypothetical protein                    (417) --------------------------------------------------------------
Consensus                                             (745)

                                                      (807) 807         820       830       840       850       861
AT2G30600 BTB/POZ domain-containing protein           (801) QTMCKAGQFASWPITAANALLPFRFFRLVLTGPTADTSTPWNFCICYLELYGYFR
Eucalyptus grandis PREDICTED: BTB/POZ protein At1g21780 (329) -------------------------------------------------------
Klebsormidium flaccidum 002310130 hypothetical protein (390) -------------------------------------------------------
Pp3c1_11190C1.1                                       (360) -------------------------------------------------------
Pp3c7_8840V1.1                                        (371) -------------------------------------------------------
Selmo1_440857 hypothetical protein                    (388) -------------------------------------------------------
Selmo1_441307 hypothetical protein                    (417) -------------------------------------------------------
Consensus                                             (807)
```
