# Supplementary material for: Holophytochrome-Interacting Proteins in Physcomitrella: Putative Actors in Phytochrome Cytoplasmic Signaling
Source: Front Plant Sci. 2016 May 12;7:613. doi: 10.3389/fpls.2016.00613 (PMC4867686; doi:10.3389/fpls.2016.00613)
Supplement: Supplementary file 2 [file Data_Sheet_2.ZIP › SI/SI (HIP2).pdf]

Supplementary Material

Holophytochrome-interacting proteins in *Physcomitrella*: putative actors in phytochrome cytoplasmic signaling

Anna Lena Ermert, Katharina Mailliet, and Jon Hughes*

*** Correspondence:** jon.hughes@uni-giessen.de

(HIP2) Pp3c19_20830V1.1

```
ATGTGTGCATTCTTTCTCCATCTGAAGAGTACCCCTTATTTTCTCATTTTAGTGCTTTGCGTTTTGTACCCGTTCATCATG
TTTTTCCTCTCCAGGAAAACTCCTCCAGTGGCAGAGAAGTCTGTCCTTCTGCATCCCTCGCAGAAGATGAGGCACATTCCA
GCGAAGTGTCTTCACGTTTCCACGCCTACATGGTGGTTGGAGAGCCGCTTTCATTTCAGTTTTGCAGAGTACTATAACCCG
GCAAACCAAGAGTTTGGAGTTCTGCGAGTTCTTAACGACGATCTTGTCAAGCCTCAGGCAGGGTTCGGTACTCACGGTCAT
CGTGACATGGAAATCTTCACGTACATTGTGGATGGAAAACTGACCCACAGAGACAGCATTGGGACGTCTGAGACCTTGGGT
CGTGGCTCGGTTCAGTACATGAGTGCGGGGACAGGGATTAGACATTCTGAGATGAACAACGGCGATGACTTGTTGCGCTTC
TTACAAATATGGATCAAGCCTGACAGATATGGTCTCAAGCCGAATTATGGTTCACGTGTATTCAAGAAAGACGACCGTCAC
AACAAGCTGCAGCACGTGTTGACAGATTTCAAAAGATACGAGAGCGAGAAAGATGCTGGTGAGGGTGTTATCCCCATCCAC
CAGGACTGCAATATTTATGTATCCGAGGCCGATGCAGGTGTTGTACAAGACTTCGTGCTTGCTAAGAAACGGCAAGCCTAT
ATGGTGTGCATTGAGGGTAAACTTTCTTTAAGTGAGAAGGTGCAGCTGGACTTTCGTGATGCGGTTGAGATAACGGCGGGA
GCTGTAGAAGATCTACCACTGAAGCTCAAGGCTGATGAGAATGTGGGCGCACACTATATTATAATCGAGATGGCTCTGGCG
TAA
```

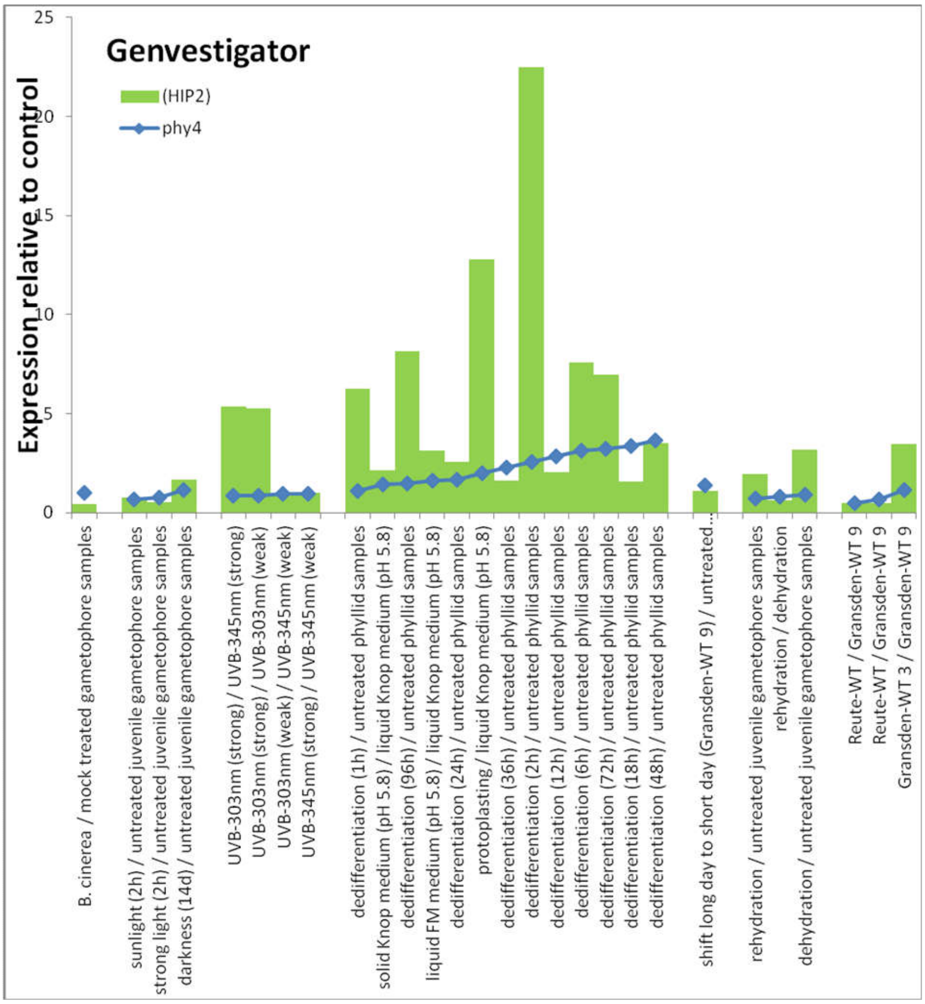
Genvestigator
(HIP2)
phy4
Expression relative to control
0
5
10
15
20
25
B. cinerea / mock treated gametophore samples
sunlight (2h) / untreated juvenile gametophore samples
strong light (2h) / untreated juvenile gametophore samples
darkness (14d) / untreated juvenile gametophore samples
UVB-303nm (strong) / UVB-345nm (strong)
UVB-303nm (strong) / UVB-303nm (weak)
UVB-303nm (weak) / UVB-345nm (weak)
UVB-345nm (strong) / UVB-345nm (weak)
dedifferentiation (1h) / untreated phyllid samples
solid Knop medium (pH 5.8) / liquid Knop medium (pH 5.8)
dedifferentiation (96h) / untreated phyllid samples
liquid FM medium (pH 5.8) / liquid Knop medium (pH 5.8)
dedifferentiation (24h) / untreated phyllid samples
protoplasting / liquid Knop medium (pH 5.8)
dedifferentiation (36h) / untreated phyllid samples
dedifferentiation (2h) / untreated phyllid samples
dedifferentiation (12h) / untreated phyllid samples
dedifferentiation (6h) / untreated phyllid samples
dedifferentiation (72h) / untreated phyllid samples
dedifferentiation (18h) / untreated phyllid samples
dedifferentiation (48h) / untreated phyllid samples
shift long day to short day (Gransden-WT 9) / untreated...
rehydration / untreated juvenile gametophore samples
rehydration / dehydration
dehydration / untreated juvenile gametophore samples
Reute-WT / Gransden-WT 9
Reute-WT / Gransden-WT 9
Gransden-WT 3 / Gransden-WT 9

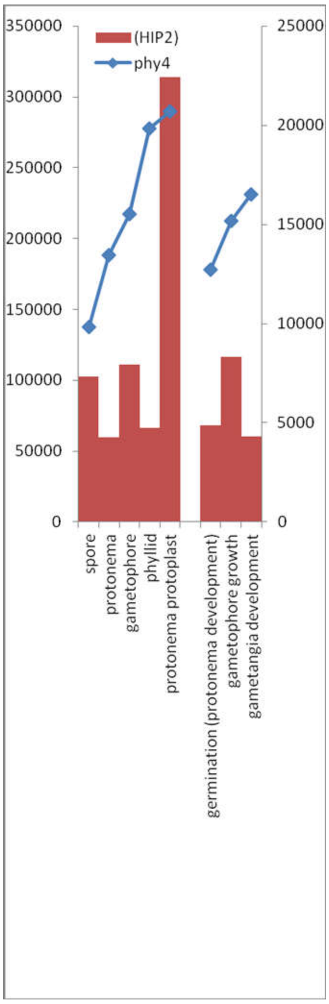
(HIP2)
phy4
0
50000
100000
150000
200000
250000
300000
350000
0
5000
10000
15000
20000
25000
spore
protonema
gametophore
phyllid
protonema protoplast
germination (protonema development)
gametophore growth
gametangia development

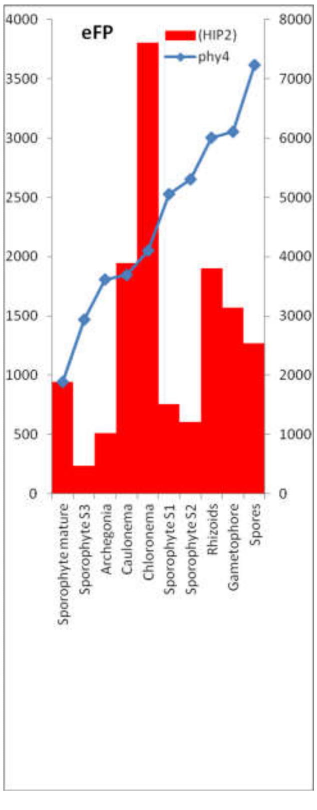
eFP
(HIP2)
phy4
0
500
1000
1500
2000
2500
3000
3500
4000
0
1000
2000
3000
4000
5000
6000
7000
8000
Sporophyte mature
Sporophyte S3
Archegonia
Caulonema
Chloronema
Sporophyte S1
Sporophyte S2
Rhizoids
Gametophore
Spores

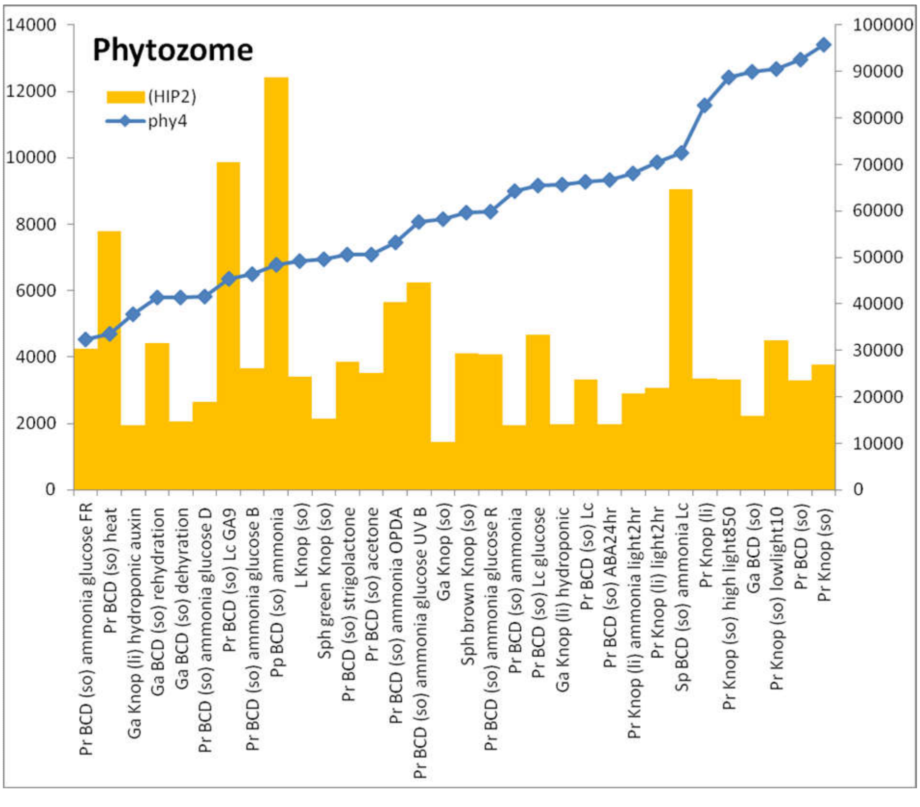
Phytozome
(HIP2)
phy4
0
2000
4000
6000
8000
10000
12000
14000
0
10000
20000
30000
40000
50000
60000
70000
80000
90000
100000
Pr BCD (so) ammonia glucose FR
Pr BCD (so) heat
Ga Knop (li) hydroponic auxin
Ga BCD (so) rehydration
Ga BCD (so) dehyration
Pr BCD (so) ammonia glucose D
Pr BCD (so) Lc GA9
Pr BCD (so) ammonia glucose B
Pp BCD (so) ammonia
L Knop (so)
Sph green Knop (so)
Pr BCD (so) strigolactone
Pr BCD (so) acetone
Pr BCD (so) ammonia OPDA
Pr BCD (so) ammonia glucose UV B
Ga Knop (so)
Sph brown Knop (so)
Pr BCD (so) ammonia glucose R
Pr BCD (so) ammonia
Pr BCD (so) Lc glucose
Ga Knop (li) hydroponic
Pr BCD (so) Lc
Pr BCD (so) ABA24hr
Pr Knop (li) ammonia light2hr
Pr Knop (li) light2hr
Sp BCD (so) ammonia Lc
Pr Knop (li)
Pr Knop (so) high light850
Ga BCD (so)
Pr Knop (so) lowlight10
Pr BCD (so)
Pr Knop (so)
