# Supplementary material for: Holophytochrome-Interacting Proteins in Physcomitrella: Putative Actors in Phytochrome Cytoplasmic Signaling
Source: Front Plant Sci. 2016 May 12;7:613. doi: 10.3389/fpls.2016.00613 (PMC4867686; doi:10.3389/fpls.2016.00613)
Supplement: Supplementary file 2 [file Data_Sheet_2.ZIP › SI/SI Figures S1 - S6 and Tables S1 & S2.pdf]

Supplementary Material

Holophytochrome-interacting proteins in *Physcomitrella*: putative actors in phytochrome cytoplasmic signaling

Anna Lena Ermert, Katharina Mailliet, and Jon Hughes*

*** Correspondence:** jon.hughes@uni-giessen.de

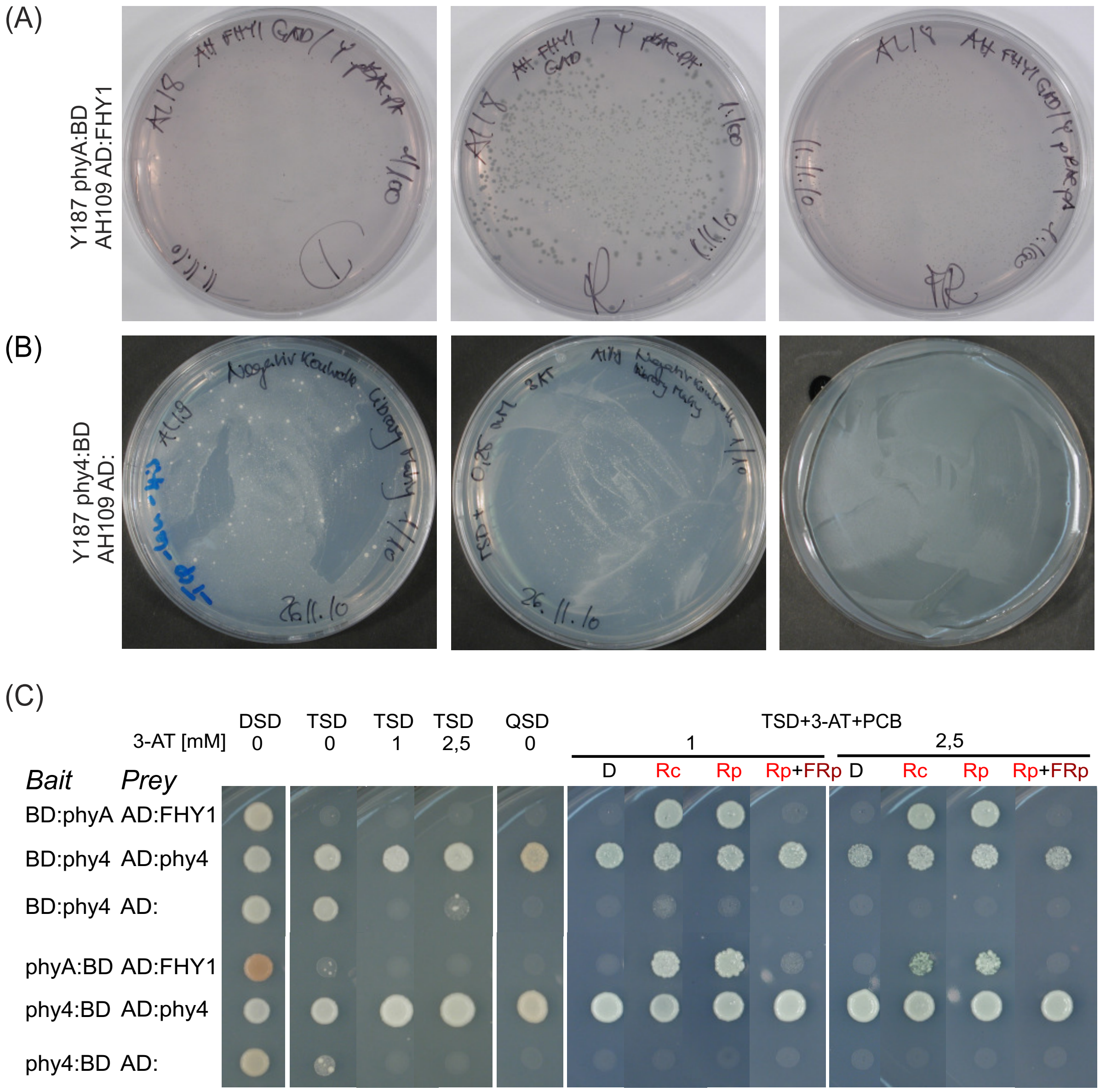

Supplementary Figure S1. Yeast two-hybrid controls: light-dependent positive control for chromophore uptake holophytochrome assembly in mating **(A)**, negative control in mating **(B)** and controls for semi-quantitative holophytochrome yeast growth assays **(C)**. **(A)** Y187 (mat α) PHYA:BD was mated to AH109 (mat a) AD:FHY, a 1:100 dilution of the mating mixture was plated on TSD medium with 30 µM PCB and plates incubated in D (left), 0.7 µmol m^{-2} s^{-1} R (middle) or 3 µmol m^{-2} s^{-1} FR (right). R/FR reversible interaction of phyA:BD–AD:FHY1 is apparent, indicating successful PCB uptake and assembly of the PHYA:BD hybrid apoprotein to the functional phyA:BD holoprotein. **(B)** As a negative control, Y187 transformed with the screening bait construct PHY4:BD was mated to AH109 AD: in the same ratio as the screening mating (2:1). 1:10-dilutions were plated on TSD without 3-AT (left), on TSD with 0.25 mM 3-AT (middle) and on TSD with 0.25 mM 3-AT and 30 µM PCB and incubated in 0.7 µmol m^{-2} s^{-1} R (right, screening conditions). The negative control produced no significant growth under screening conditions. **(C)** For growth assay controls, 2 x 10^5 cells of doubly-transformed yeast was spotted onto different media without PCB (DSD, TSD, TSD with 1 mM 3-AT, TSD with 2.5 mM 3-AT and QSD) and on TSD medium with 30 µM PCB containing different 3-AT concentrations (1 mM for phyA baits and phy4:BD and 2.5 mM for BD:phy4). PCB plates were incubated in D, Rc (0.8 µmol m^{-2} s^{-1} R) , Rp (4 µmol m^{-2} s^{-1} R for 12 min + 48 min D) and Rp+FRp (12 min Rp + 12 min FR, 5 µmol m^{-2} s^{-1} + 36 min D). Analogous to the mating positive control (A), spotting positive controls BD:phyA–AD:FHY1 and phyA:BD-AD:FHY1 show strong light-dependent interaction only in presence of PCB (see 1µM 3-AT plates). BD:phy4 and phy4:BD both constitutively and strongly dimerize with AD:phy4 even at very high 3-AT concentrations (see phy4:BD–AD:phy4 on 2.5 mM 3-AT). BD:phy4 and phy4:BD in combination with AD: constitute negative controls, which showed no significant growth on all 3-AT plates without and with PCB.

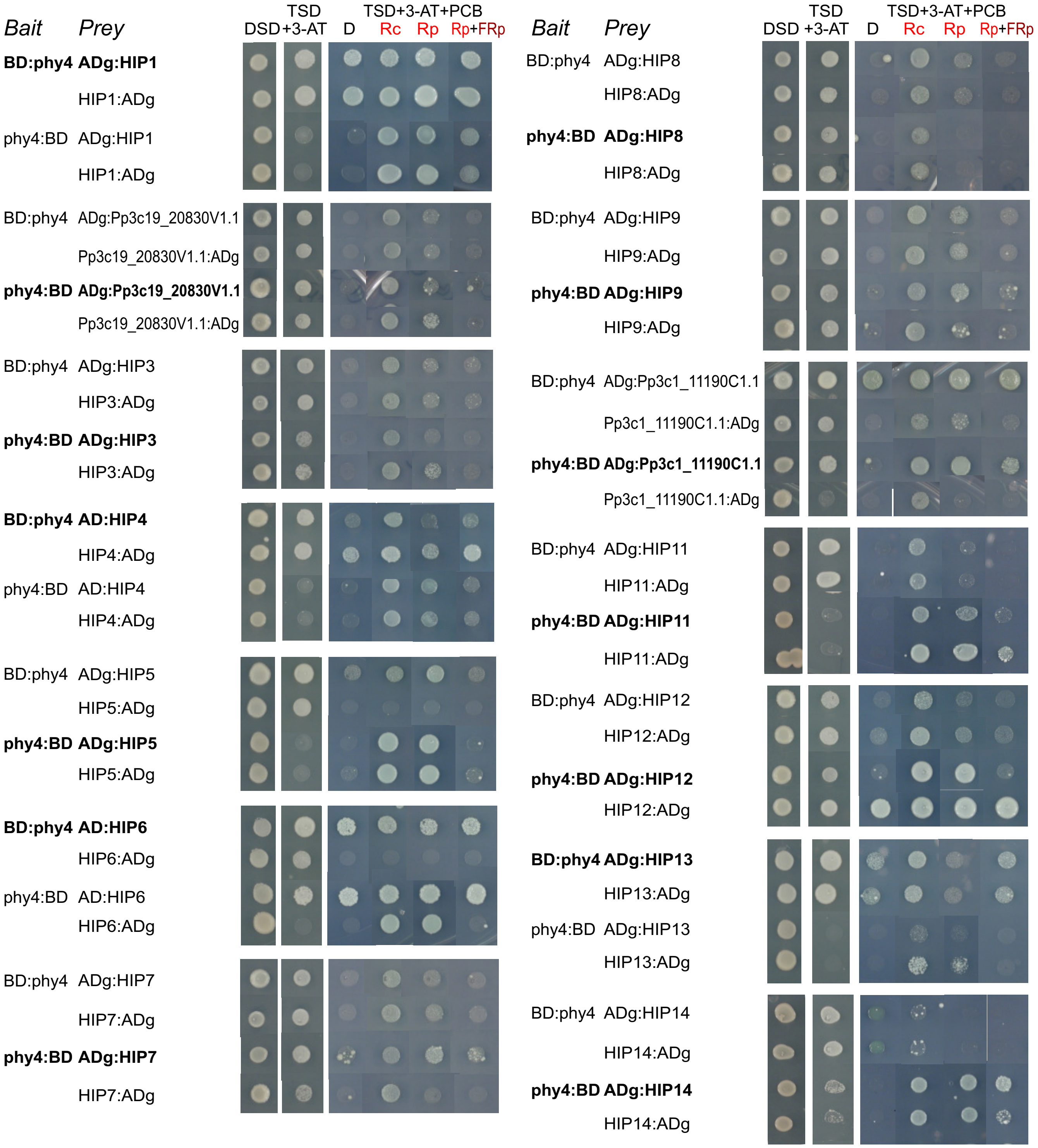
Bait
Prey
DSD
TSD +3-AT
TSD+3-AT+PCB
D
Rc
Rp
Rp+FRp
BD:phy4 ADg:HIP1
HIP1:ADg
phy4:BD ADg:HIP1
HIP1:ADg
BD:phy4 ADg:Pp3c19_20830V1.1
Pp3c19_20830V1.1:ADg
phy4:BD ADg:Pp3c19_20830V1.1
Pp3c19_20830V1.1:ADg
BD:phy4 ADg:HIP3
HIP3:ADg
phy4:BD ADg:HIP3
HIP3:ADg
BD:phy4 AD:HIP4
HIP4:ADg
phy4:BD AD:HIP4
HIP4:ADg
BD:phy4 ADg:HIP5
HIP5:ADg
phy4:BD ADg:HIP5
HIP5:ADg
BD:phy4 AD:HIP6
HIP6:ADg
phy4:BD AD:HIP6
HIP6:ADg
BD:phy4 ADg:HIP7
HIP7:ADg
phy4:BD ADg:HIP7
HIP7:ADg
Bait
Prey
DSD
TSD +3-AT
TSD+3-AT+PCB
D
Rc
Rp
Rp+FRp
BD:phy4 ADg:HIP8
HIP8:ADg
phy4:BD ADg:HIP8
HIP8:ADg
BD:phy4 ADg:HIP9
HIP9:ADg
phy4:BD ADg:HIP9
HIP9:ADg
BD:phy4 ADg:Pp3c1_11190C1.1
Pp3c1_11190C1.1:ADg
phy4:BD ADg:Pp3c1_11190C1.1
Pp3c1_11190C1.1:ADg
BD:phy4 ADg:HIP11
HIP11:ADg
phy4:BD ADg:HIP11
HIP11:ADg
BD:phy4 ADg:HIP12
HIP12:ADg
phy4:BD ADg:HIP12
HIP12:ADg
BD:phy4 ADg:HIP13
HIP13:ADg
phy4:BD ADg:HIP13
HIP13:ADg
BD:phy4 ADg:HIP14
HIP14:ADg
phy4:BD ADg:HIP14
HIP14:ADg

Supplementary Figure S2. Semiquantitative Y2H growth assay using full length holo phy4 baits and HIP1-14 preys in different light conditions (D, Rc, Rp, Rp+FRp, see Materials & Methods and SI Fig. 1 legend). Full length HIP CDS sequences were cloned in AD: and :AD fusion vectors and combined with BD:PHY4 or PHY4:BD constructs via yeast double transformation, thus four possible bait/prey combinations per HIP. Pp3c19_20830V1.1 and Pp3c1_11190C1.1 correspond to the provisionally-named HIP's 2 and 10, respectively. 2 x 10^5 doubly-transformed yeast cells were spotted onto non-selective DSD medium, selective TSD+3-AT medium and TSD with 3-AT and PCB medium for incubation in different light conditions. For the BD:phy4 bait and for the phy4:BD bait 2.5 mM and 1 mM 3-AT were used, respectively.

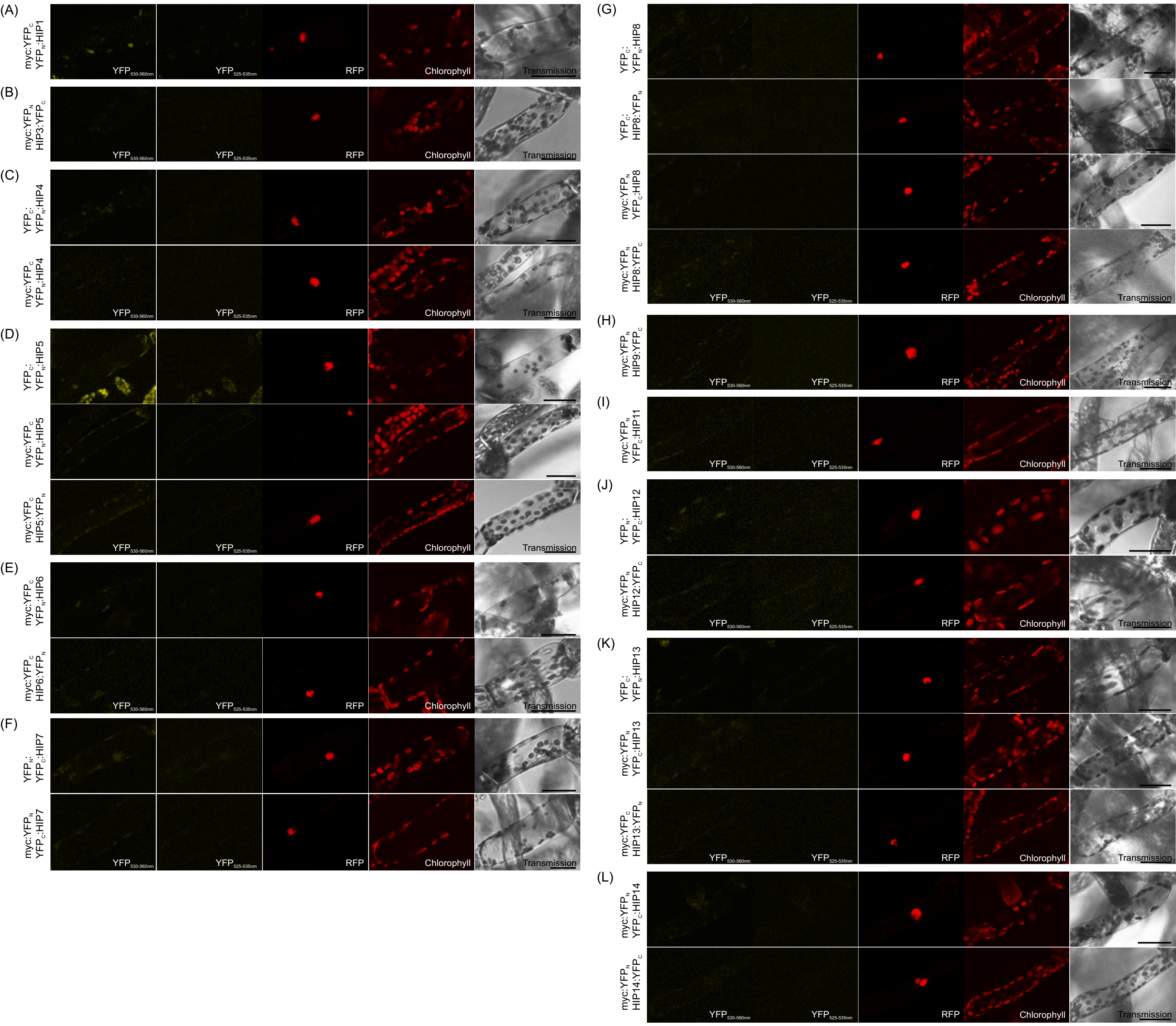

(A)
myc:YFPC
YFPN:HIP1
(B)
myc:YFPN
HIP3:YFPC
(C)
YFPC:
YFPN:HIP4
myc:YFPC
YFPN:HIP4
(D)
YFPC:
YFPN:HIP5
myc:YFPC
YFPN:HIP5
myc:YFPC
HIP5:YFPN
(E)
myc:YFPC
YFPN:HIP6
myc:YFPC
HIP6:YFPN
(F)
YFPN:
YFPC:HIP7
myc:YFPN
YFPC:HIP7
(G)
YFPC:
YFPN:HIP8
YFPC:
HIP8:YFPN
myc:YFPN
YFPC:HIP8
myc:YFPN
HIP8:YFPC
(H)
myc:YFPN
HIP9:YFPC
(I)
myc:YFPN
YFPC:HIP11
(J)
YFPN:
YFPC:HIP12
myc:YFPN
HIP12:YFPC
(K)
YFPC:
YFPN:HIP13
myc:YFPN
YFPC:HIP13
myc:YFPC
HIP13:YFPN
(L)
myc:YFPN
YFPC:HIP14
myc:YFPN
HIP14:YFPC
YFP530-560nm
YFP525-535nm
RFP
Chlorophyll
Transmission

Supplementary Figure S3. Split-YFP negative controls. As negative controls for phy4–HIP *in planta* split-YFP interaction assay configurations that yielded significant signals, the phy4 construct was replaced by the respective empty destination vector (N-terminal fusions) or by myc:YFP_N / myc:YFP_C, respectively. Negative controls were observed via confocal microscopy for HIP1 **(A)**, HIP3 **(B)**, HIP4 **(C)**, HIP5 **(D)**, HIP6 **(E)**, HIP7 **(F)**, HIP8 **(G)**, HIP9 **(H)**, HIP11 **(I)**, HIP12 **(J)**, HIP13 **(K)** and HIP14 **(L)**. Column 1: YFP emission corresponding to main text figures. Column 2: narrowed YFP emission detection between 525 and 535 nm for distinction between mCherry:NLS nuclear marker and/or chloroplast bleed-through into the YFP channel ($YFP_{525\text{-}535}$). Columns 3-5 comprise the RFP fluorescence of the nuclear marker (RFP), chlorophyll autofluorescence (Chlorophyll) and a transmission image (Transmission), respectively. All negative controls were performed without R pre-treatment with exception of myc:YFP_N–YFP_C:HIP7 and myc:YFP_N–HIP9:YFP_C (~1h R), since those phy4–HIP interactions showed light-induced interaction *in planta*. Scale bars 30 µm.

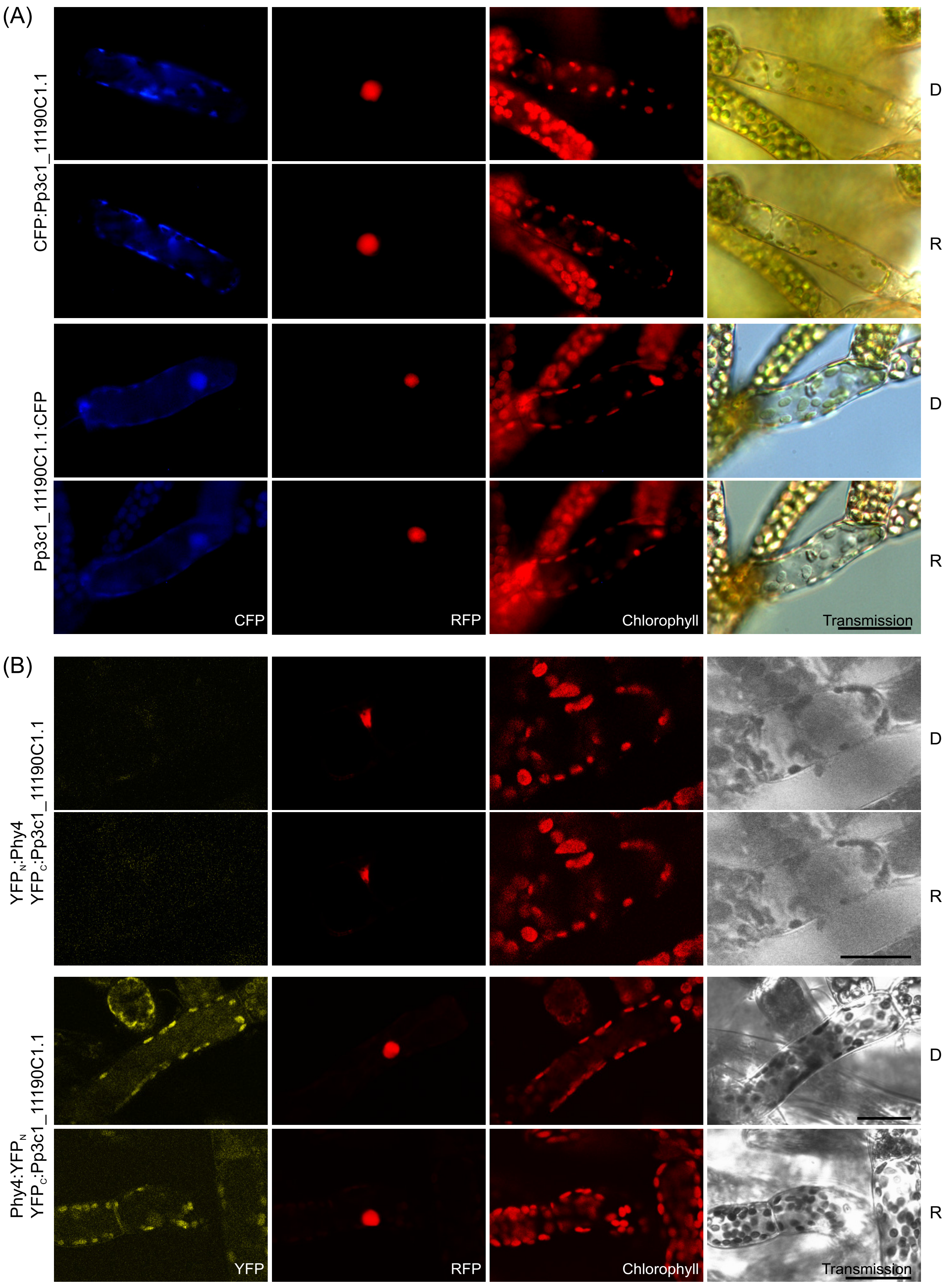
(A)
CFP:Pp3c1_11190C1.1
Pp3c1_11190C1.1:CFP
D
R
D
R
CFP
RFP
Chlorophyll
Transmission
(B)
YFP$_N$:Phy4
YFP$_C$:Pp3c1_11190C1.1
Phy4:YFP$_N$
YFP$_C$:Pp3c1_11190C1.1
D
R
D
R
YFP
RFP
Chlorophyll
Transmission

Supplementary Figure S4. Pp3c1_11190C1.1 (HIP10) intracellular localization (A) and split-YFP-studies (B) each without (D) and with R-pretreatment (R) using fluorescence- and confocal microscopy, respectively. Overall figure structure for panels A and B is analogous to Figure 1 of the main text. **(A)** CFP was fused to the N- (rows 1 & 2) and C- terminus (rows 3 & 4) of the full length CDS for intracellular localization analysis. **(B)** Corresponding to the Y2H interaction behavior, split-YFP configurations of YFP_C:Pp3c1_11190C1.1 in combination with N- and C-terminally fused phy4 were analyzed: YFP_N:phy4–YFP_C:Pp3c1_11190C1.1 (rows 1 & 2) and phy4:YFP_N–YFP_C:Pp3c1_11190C1.1 (rows 3 & 4). None of the tested configurations yielded significant signals, thus this protein was omitted from the main text. In both panels A and B, columns 3-5 show the RFP fluorescence of the nuclear marker, chlorophyll autofluorescence and the transmission image, respectively. Scale bars 30 µm.

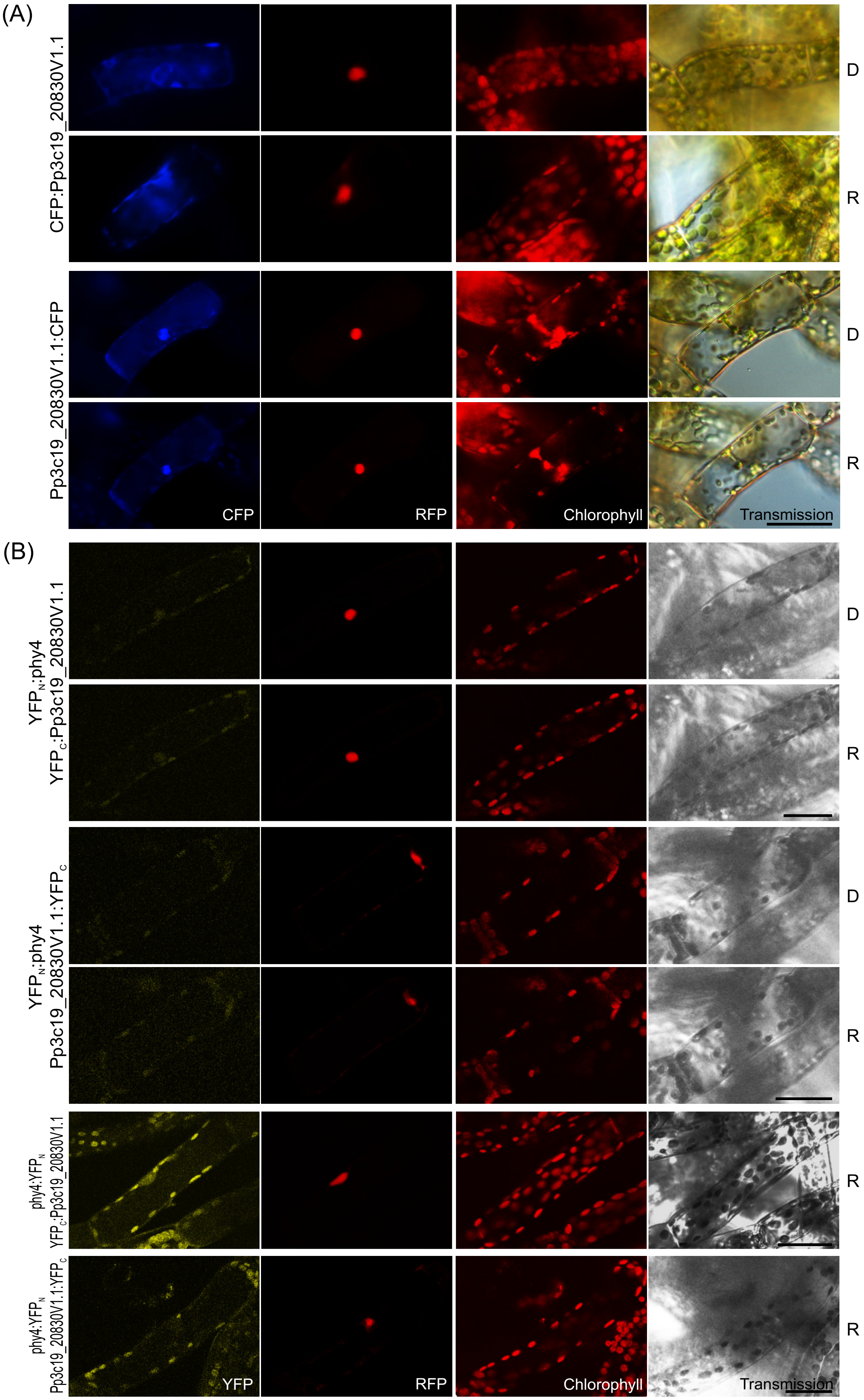

(A)
CFP:Pp3c19_20830V1.1
Pp3c19_20830V1.1:CFP
D
R
D
R
CFP
RFP
Chlorophyll
Transmission
(B)
YFP$_N$:phy4
YFP$_C$:Pp3c19_20830V1.1
YFP$_N$:phy4
Pp3c19_20830V1.1:YFP$_C$
phy4:YFP$_N$
YFP$_C$:Pp3c19_20830V1.1
phy4:YFP$_N$
Pp3c19_20830V1.1:YFP$_C$
D
R
D
R
R
R
YFP
RFP
Chlorophyll
Transmission

Supplementary Figure S5. Pp3c19_20830V1.1 (HIP2) intracellular localization (A) and split-YFP studies (B) each without (D) and with red pre-treatment (R) using fluorescence and confocal microscopy, respectively. Overall figure structure for panels A and B is analogous to Figure 1 of the main text / SI Figure 4. **(A)** CFP was fused to the N- (rows 1+2) and C-terminus (rows 3 & 4) of the full length CDS for intracellular localization analysis. **(B)** All possible split-YFP configurations were investigated: YFP_N:phy4–YFP_C:Pp3c19_20830V1.1 (rows 1 & 2), YFP_N:phy4–Pp3c19_20830V1.1:YFP_C (rows 3 & 4), phy4:YFP_N–YFP_C:Pp3c19_20830V1.1 (row 5, R only) and phy4:YFP_N–Pp3c19_20830V1.1:YFP_C (row 6, R only). No significant YFP-signal was observed, thus Pp3c19_20830V1.1 was omitted from the main text. Scale bars 30 µm.

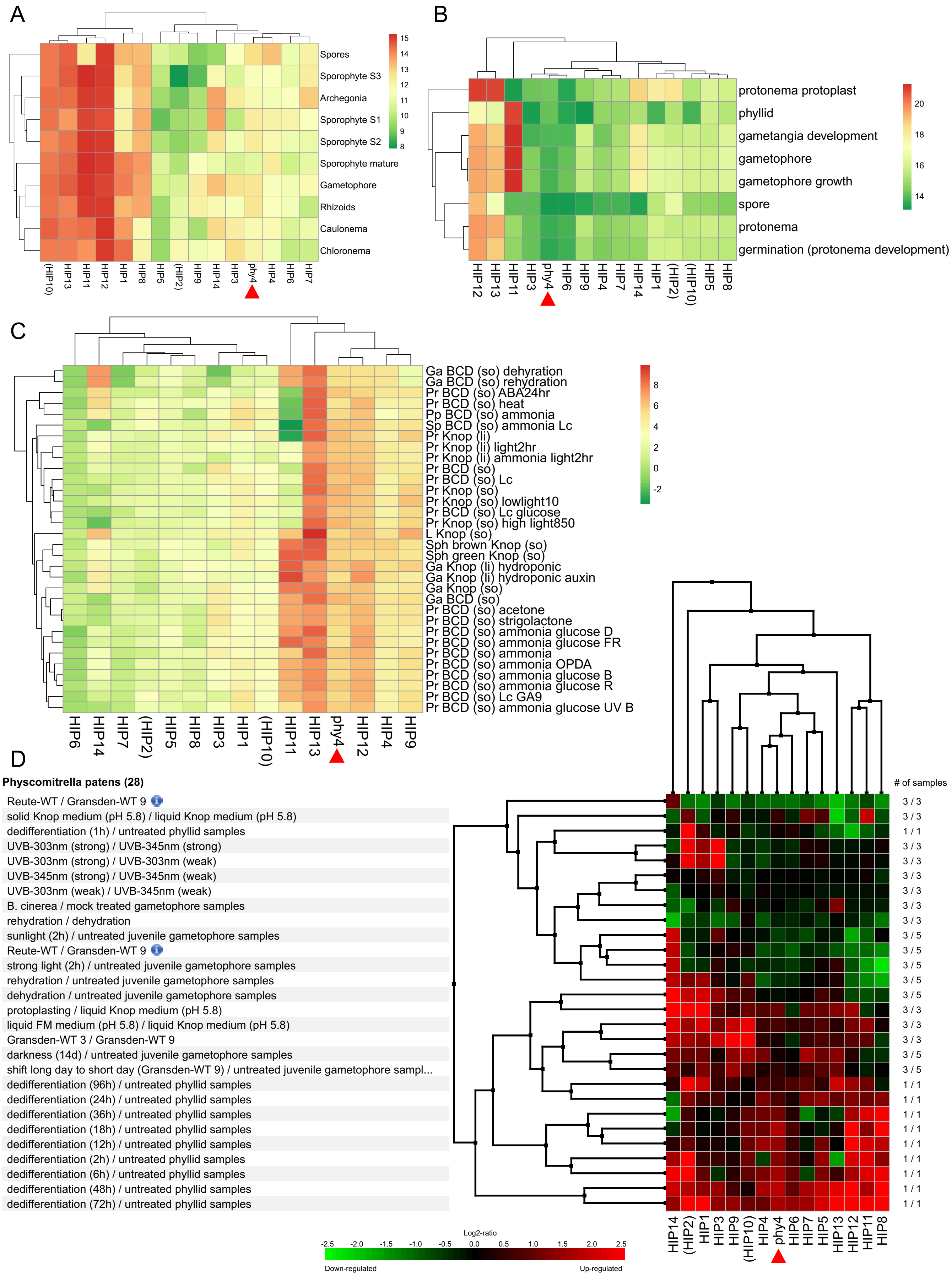

A
Spores
Sporophyte S3
Archegonia
Sporophyte S1
Sporophyte S2
Sporophyte mature
Gametophore
Rhizoids
Caulonema
Chloronema
(HIP10) HIP13 HIP11 HIP12 HIP1 HIP8 HIP5 (HIP2) HIP9 HIP14 HIP3 phy4 HIP4 HIP6 HIP7
B
protonema protoplast
phyllid
gametangia development
gametophore
gametophore growth
spore
protonema
germination (protonema development)
HIP12 HIP13 HIP11 HIP3 phy4 HIP6 HIP9 HIP4 HIP7 HIP14 HIP1 (HIP2) (HIP10) HIP5 HIP8
C
Ga BCD (so) dehyration
Ga BCD (so) rehydration
Pr BCD (so) ABA24hr
Pr BCD (so) heat
Pp BCD (so) ammonia
Sp BCD (so) ammonia Lc
Pr Knop (li)
Pr Knop (li) light2hr
Pr Knop (li) ammonia light2hr
Pr BCD (so)
Pr BCD (so) Lc
Pr Knop (so)
Pr Knop (so) lowlight10
Pr BCD (so) Lc glucose
Pr Knop (so) high light850
L Knop (so)
Sph brown Knop (so)
Sph green Knop (so)
Ga Knop (li) hydroponic
Ga Knop (li) hydroponic auxin
Ga Knop (so)
Ga BCD (so)
Pr BCD (so) acetone
Pr BCD (so) strigolactone
Pr BCD (so) ammonia glucose D
Pr BCD (so) ammonia glucose FR
Pr BCD (so) ammonia
Pr BCD (so) ammonia OPDA
Pr BCD (so) ammonia glucose B
Pr BCD (so) ammonia glucose R
Pr BCD (so) Lc GA9
Pr BCD (so) ammonia glucose UV B
HIP6 HIP14 HIP7 (HIP2) HIP5 HIP8 HIP3 HIP1 (HIP10) HIP11 HIP13 phy4 HIP12 HIP4 HIP9
D
Physcomitrella patens (28)
# of samples
Reute-WT / Gransden-WT 9 — 3 / 3
solid Knop medium (pH 5.8) / liquid Knop medium (pH 5.8) — 3 / 3
dedifferentiation (1h) / untreated phyllid samples — 1 / 1
UVB-303nm (strong) / UVB-345nm (strong) — 3 / 3
UVB-303nm (strong) / UVB-303nm (weak) — 3 / 3
UVB-345nm (strong) / UVB-345nm (weak) — 3 / 3
UVB-303nm (weak) / UVB-345nm (weak) — 3 / 3
B. cinerea / mock treated gametophore samples — 3 / 3
rehydration / dehydration — 3 / 3
sunlight (2h) / untreated juvenile gametophore samples — 3 / 5
Reute-WT / Gransden-WT 9 — 3 / 5
strong light (2h) / untreated juvenile gametophore samples — 3 / 5
rehydration / untreated juvenile gametophore samples — 3 / 5
dehydration / untreated juvenile gametophore samples — 3 / 5
protoplasting / liquid Knop medium (pH 5.8) — 3 / 3
liquid FM medium (pH 5.8) / liquid Knop medium (pH 5.8) — 3 / 3
Gransden-WT 3 / Gransden-WT 9 — 3 / 3
darkness (14d) / untreated juvenile gametophore samples — 3 / 5
shift long day to short day (Gransden-WT 9) / untreated juvenile gametophore sampl... — 3 / 5
dedifferentiation (96h) / untreated phyllid samples — 1 / 1
dedifferentiation (24h) / untreated phyllid samples — 1 / 1
dedifferentiation (36h) / untreated phyllid samples — 1 / 1
dedifferentiation (18h) / untreated phyllid samples — 1 / 1
dedifferentiation (12h) / untreated phyllid samples — 1 / 1
dedifferentiation (2h) / untreated phyllid samples — 1 / 1
dedifferentiation (6h) / untreated phyllid samples — 1 / 1
dedifferentiation (48h) / untreated phyllid samples — 1 / 1
dedifferentiation (72h) / untreated phyllid samples — 1 / 1
Log2-ratio
-2.5 -2.0 -1.5 -1.0 -0.5 0.0 0.5 1.0 1.5 2.0 2.5
Down-regulated
Up-regulated
HIP14 (HIP2) HIP1 HIP3 HIP9 (HIP10) HIP4 phy4 HIP6 HIP7 HIP5 HIP13 HIP12 HIP11 HIP8

Supplementary Figure S6. Hierarchical clustering of phy4 and HIP expression data retrieved from eFP browser (A) and Genevestigator (B) for anatomy/developmental stages and from Phytozome (C) and Genevestigator (D) for different treatments. Data were log2 transformed and clustered using ClustVis (A,B & C) or Genevestigator (D), respectively in all cases applying the Manhattan distance metric. **(A)** Arithmetic mean of triplicate RMA-normalized eFP microarray data. **(B)** Mean Genevestigator microarray data for anatomy and developmental stages. **(C)** Phytozome11 FPKM (fragments per kb exon per million reads) RNAseq values for different conditions. In **(D)**, the Genevestigator mean log2 microarray data for different conditions are shown as relative values of a condition relative to the log2 value of appropriate control mean. If the resulting values were < 1, the negative reciprocal was calculated to produce a scale from negative values (down-regulation) via 0 (no expression change) to positive values (up-regulation).

Supplementary Table S1. Putative interactors obtained from the apo BD:PHY4 oligo-dT library Y2H screen

24 sequences from 23 yeast clones (excluding duplicates) were analyzed; Columns 2 and 3 indicate the yeast clone number and the Cosmoss accession number, respectively. Column 4 summarizes the possible protein function mostly inferred from domain and ortholog analyses. The last two columns describe the results of localization prediction and the corresponding HIP- and main text figure numbers where applicable, respectively.

Nr.	Yeast clone	Cosmoss Accession	Gene description (inferred from search of domains or orthologs; if not indicated otherwise)	Localization prediction (WOLFPsort if not indicated otherwise)	HIP Nr. Fig. Nr.
1	#1.7	Pp1s109_35V2.1	Expressed protein of unknown function	cytoplasm	
2	#2.3	Pp1s38_285V6.1 / Pp3c1_7540E5.1	Putative wound responsive protein, DUF 151 superfamily protein	cytoplasm; Plant-mPloc: chloroplast	
3	#8.5	Pp1s230_33V5.1	GIY-YIG superfamily protein	extracellular, mitochrondrium	
4	#8.7	Pp1s19_140V6.1 / Pp3c18_9180V3.4	Serine/threonine protein kinase; similarity to ceratodon phytochrome C-terminus	Plant-mPloc: chloroplast/cytoplasm/nucleus.	
5	#9.2	Pp1s419_7V6.1 / Pp3c15_13010C1.1	Lipoxygenase familiy protein	cytoplasm, mitochrondrium; Plant-mPloc: cytoplasm	
6	#10.3	Pp1s47_42V6.1 / Pp3c3_23700C1.1	40S ribosomal protein, ribosomal S15 protein	cytoplasm; Plant-mPloc: nucleus	
7	#11.5	Pp3c7_12270P2.5	60S ribosomal protein L13a	cytoplasm; Plant-mPloc: chloroplast	
8	#12.5	Pp3c1_35670E2.1	Putative fatty acid desaturase	nucleus; Plant-mPloc: nucleus	
9	#16.1	Pp3c6_4480 e.g. V3.5	P-loop protein	cytoplasm	HIP6, Fig. 5
10	#21.3	Pp1s291_39E1.1 / Pp3c15_6300E1.1	Hydroxymethylbilane synthase (phorphobilinogen deaminase)	Plant-mPloc: chloroplast	
11	#25.3	Pp1s66_233V6.1 / Pp3c15_24440E1.1	Pore protein 24 kD	secreted; Plant-mPloc: cell wall	
12	#31.4	Pp1s480_1E1.1/V6.1	Predicted protein transport protein, ACE1-Sec16-like protein, RGPR-related protein	Plant-mPloc: nucleus	
13	#32.2	Pp1s52_258V2.1	Putative dehydrin	nucleus	
14	#33.7	Pp1s169_71V2.1 / Pp3c16_8560E1.1	Pleiotropic regulatory locus 1 (PRL1)	cytoplasm	HIP4, Fig. 3
15	#34.1	Pp1s238_57V2.1	Cystein protease inhibitor (possibly a PRL1 interactor)	cytoplasm / nucleus	
16	#39.3	Pp1s459_1V2.1	RuBisCO small subunit 1A	chloroplast	
17	#42.8	Pp3c3_2620E1.1	PsbQ family, oxygen evolving complex of photosystem II subunit	chloroplast; Plant-mPloc: chloroplast	

			PsbQ		
18	#46.3	Pp1s517_8V2.1	DNA topoisomerase II - gyrase	nucleus	
19	#47.8	Pp1s134_155E1.1 / Pp3c8_22400V3.6	VQ-motif-containing protein; uncharacterized	Plant-mPLoc: nucleus	
20	#53.2	Pp1s77_65V2.1	PSI reaction center subunit II, 20 kD subunit	chloroplast	
21	#54.1	Pp3c1_23670V1.1	Elongation factor 1 alpha, EF1-α	cytoplasm	HIP13, Fig. 11
22	#61.4	Pp3c2_10320V1.1	Pirin, cupin superfamily protein	cytoplasm /At mitochondrial	HIP1, Fig. 1
23	#66.5	Pp1s74_203V2.1	OTU-like cystein protease family protein	cytoplasm	
24	#68.6	Pp3c513520E1.1	Putative metallothionine	Plant-mPLoc: cell membrane, cell wall, golgi apparatus, nucleus	

Supplementary Table S2. Putative interactors obtained from the holo phy4:BD oligo dT library mating screen

60 sequences from 54 yeast clones (excluding duplicates) were analyzed. The columns are as in Table S1. The "unknown" indicates that no noteworthy domain and ortholog information could be obtained, whereas "uncharacterized protein" indicates that proteins with similarities to the respective *Physcomitrella* sequence have no known function.

**Nr.**	**Yeast clone**	**Cosmoss Accession**	**Gene description (inferred from annotation or domain- or ortholog- analysis)**	**Localization prediction**	**HIP Nr. Fig. Nr.**
1	2.1	Pp3s107_40E1.1	Photosystem I P700 chlorophyll a apoprotein A1	Plant-mPLoc: chloroplast; TargetP: secretory pathway	
2	2.2	Pp1s271_60V6.1	F-box/kelch-repeat protein	Plant-mPLoc: chloroplast; TargetP: other	
3	2.3	Pp3c15_20330J1.1	TP53-regulating kinase	Plant-mPLoc: nucleus; TargetP: other	
4	2.6	Pp1s227_44V6.1	Phytocyanin, plastocyanin-like, Cu-binding-like	Plant-mPLoc: cell membrane; TargetP: secretory pathway	
5	2.7	Pp3c13_11180V1.1	U6 snRNA-associated Sm-like protein LSm4	Plant-mPLoc: nucleus; TargetP: other	
6	2.8	Pp1s215_81V6.1 / Pp3c25_1430C1.1	40S ribosomal protein, ribosomal protein S13	Plant-mPLoc: mitochondrium, nucleus; TargetP: other	
7	2.11	Pp1s326_44V6.1	NADH:flavin oxidoreductase / NADH oxidase, N-terminal with a old yellow enzyme (OYE)-like FMN binding domain	Plant-mPLoc: cytoplasm; TargetP: other	
8	2.16	Pp1s149_88V6.1	Glycosyl transferase, family 14	Plant-mPLoc: chloroplast; TargetP: mitochondrium	
9	2.16	Pp1s140_63V6.1 / Pp3c3_8540C1.1	14-3-3 protein	Plant-mPLoc: nucleus; TargetP: other; WoLFPSORT: nucleus / plastid:	HIP14, Fig. 12
10	2.17	Pp1s139_105V6.1 / Pp3c1_11190C1.1	Ankyrin-BTB/POZ-domain protein	Plant-mPLoc: nucleus; WoLFPSORT cytoplasm	HIP10; no split-YFP signal, SI Fig. 4
11	e.g. 2.19	Pp1s34_442V6.2	Unknown	Plant-mPLoc: nucleus WoLFPSORT: chloroplast; TargetP: secretory pathway	
12	2.20	Pp1s340_22V6.1	Ribosomal RNA processing Brix-domain protein	Plant-mPLoc: chloroplast; TargetP: mitochondrium	
13	2.21	Pp1s287_61V6.2	ATPase, F1 complex, gamma subunit	Plant-mPLoc: chloroplast; TargetP: chloropolast	

14	2.25	Pp1s52_261V6.1	Unknown	Plant-mPLoc: cytoplasm, nucleus; TargetP: other	
15	2.27	Pp1s52_157V6.1	Chlorophyll A-B binding protein, plant	Plant-mPLoc: chloroplast; TargetP: chloroplast	
16	2.29	Pp1s45_25V6.2 / Pp3c1_3610C1.2	Uncharacterized protein	Plant-mPLoc: cell membrane; WoLFPSORT: cytoplasm; TargetP: secretory pathway	
17	2.30	Pp1s316_6V6.1; 3´UTR maybe shorter	40S ribosomal protein, ribosomal protein S11	Plant-mPLoc: chloroplast, cytoplasm; TargetP: other	
18	e.g. 2.35	Pp1s44_116V6.1 / Pp3c20_16210V1.1	Unknown	Plant-mPLoc: cell membrane; WoLFPSORT: extracellular; TargetP: secretory pathway	HIP11, Fig. 9
19	2.36	Pp1s112_169V6.1	Cytochrome b6-f complex iron-sulfur subunit	Plant-mPLoc: chloroplast; TargetP: chloroplast	
20	2.37	Pp1s55_66E3.1	Late embryogenesis abundant (plants) LEA-related	Plant-mPLoc: nucleus; TargetP: chloroplast	
21	2.38	Pp1s6_313V6.1	Chlorophyll A-B binding protein, plant	Plant-mPLoc: chloroplast; TargetP: chloroplast	
22	2.38	probably Pp3c11_970V3.4	Autophagy related protein 27	Plant-mPLoc: cell membrane; WoLFPSORT: extracellular; TargetP: secretory pathway	
23	2.39	Pp3c23_440V3.1	4-hydroxy-3-methylbut-2-enyl diphosphate reductase type 1	Plant-mPLoc: chloroplast; TargetP: chloroplast	
24	2.39	Pp1s138_79V6.1 / Pp3c11_25550V1.1	CDPK, Calcium-dependent protein kinase, "CPK17"	Plant-mPLoc: nucleus; TargetP:other; WoLFPSORT: cytoplam	HIP8, Fig. 7
25	2.40	Pp1s201_78E1.1 UTRs maybe longer	NADH-ubiquinone reductase complex 1 MLRQ subunit	Plant-mPLoc: chloroplast; TargetP: secretory pathway	
26	2.42	Pp1s109_232V6.2	Pepsin-retropepsin-like aspartate protease	Plant-mPLoc: cytoplasm; TargetP: other	
27	2.43	Pp3c10_4820V1.1	Zinc finger protein (RING-type and CHY-type)	Plant-mPLoc: cytoplasm; WoLFPSORT: nucleus; TargetP; other; GO: cytplasmic membrane-coated vesicle	HIP3, Fig. 2
28	2.45	Pp3c1_14300V1.1/ Pp1s86_25V6.1	60 S ribosomal protein, ribosomal Protein L41	TargetP: other	
29	2. 46	Pp1s444_27V6.1	Rhodopsintail-protein	Plant-mPLoc: nucleus; WoLFPSORT: extracellular; TargetP: mitochondrium	

30	2.47	Pp1s135_79V6.1	Component of photosystem II PsbP, oxygen evolving complex	Plant-mPLoc: chloroplast; TargetP: chloroplast	
31	2.48	Pp1s43_118V6.5	Carbonic anhydrase	Plant-mPLoc: golgi, nucleus; TargetP: other	
32	2.50	Pp3c7_3040V1.1	Kelch-repeat protein	Plant-mPLoc: nucleus; WoLFPSORT: nucleus; TargetP: other	HIP5, Fig. 4
33	2.50	Pp1s202_35E1.1 / Pp3c17_9390 V1.1but last intron in 5´UTR is not spliced	Ser/Thr protein kinase	Plant-mPLoc: nucleus, cytoplasm; WoLFPSORT: chloroplast; TargetP: other	HIP7 Fig. 6
34	2.52	Pp1s67_30V6.4	Maybe a histone-lysine N-methyltransferase (MEDEA) oder wall-associated receptor kinase	Plant-mPLoc: cytoplasm, cell membrane; WoLFPSORT: mitochondrium; TargetP: other	
35	2.52	Pp1s64_4V6.1 / Pp3c5_7930 V3.1	Cold shock protein with a zinc finger (CCHC-type), nucleic acid binding	Plant-mPLoc: nucleus; TargetP: other	
36	2.54	Pp1s254_10V6.2 (V3 models worse)	Rhomboid intramembrane serine protease	Plant-mPLoc: cell membrane; WoLFPSORT chloroplast; TargetP: mitochondrium; Cosmoss GO: integral membrane protein	HIP9; Fig. 8
37	2.55	Pp1s249_62V6.1	Cyclophilin-like peptidyl-prolyl cis-trans isomerase	Plant-mPLoc: cytoplasm; WoLFPSORT: cytoplasm; TargetP: other	Y2H not verified
38	2.56	Pp1s98_53V6.1	Unknown	Plant-mPLoc: chloroplast, mitochondrium, nucleus, peroxisome; TargetP: mitochondrium	
39	2.58	maybe Pp3c4_13110V1.1	Unknown	Plant-mPLoc: cell membrane, chloroplast	
40	2.59	Pp1s1_74F1.1 / Pp3c2_37000C1.2 or V3.1	Uncharacterized protein	Plant-mPLoc: nucleus; WoLFPSORT cytoplasm; TargetP: secretory pathway; GO: endomembrane system, integral membrane protein, ER	
41	2.60	Pp1s22_322V6.1	Bet v1 like (birch pollen allergen), Polyketide cyclase / dehydrase	Plant-mPLoc: cytoplasm; WoLFPSORT: cytoplasm; TargetP: other	
42	2.60	Pp1s193_54E3.1	CASC3/Barentsz eIF4AIII binding protein	Plant-mPLoc: nucleus; TargetP: other	
43	2.61	maybe Pp1s27_223E3.1	Unknown	Plant-mPLoc: nucleus; TargetP: secretory pathway	

44	2.63	Pp1s61_255V6.2	Trigger factor-like protein	Plant-mPLoc: chloroplast, mitochondrium, nucleus; TargetP: chloroplast	
45	2.65	Pp1s158_181V2.1 / Pp3c13_15620V1.1	Translation initiation/elongation factor eIF-5A	Plant-mPLoc: cytoplasm, mitochondrium; WoLFPSORT: cytoplasm; V2.1 TargetP: other	HIP12; Fig. 10
46	2.66	Pp1s101_226V2.1	Major intrinsic protein, maybe aquaporin or a tonoplast intrinsic protein	Plant-mPLoc: cell membrane, vacuole; Target P: other	
47	2.67	Pp1s209_115V6.2	Autophagy protein Atg8 ubiquitin-like	Plant-mPLoc: cytoplasm, nucleus; WoLFPSORT: cytoplasm; TargetP: other	
48	2.69	Pp1s1_489V6.1	60S ribosomal protein, ribosomal protein L29	Plant-mPLoc: chloroplast; TargetP: chloropolast	
49	2.70	Pp1s30_87V6.3	Unknown	Plant-mPLoc: cell membrane, nucleus; TargetP: secretory pathway	
50	2.72	Pp1s472_14V6.1 / Pp1s472_15V6.1 (2 Loci with identical CDS)	Chlorophyll A-B binding protein, plant	Plant-mPLoc: chloroplast; TargetP: chloroplast	
51	2.73	Pp1s59_79E1.1 or E2.1	maybe component of NADH:ubiqinone-oxidoreduktase-complex I	Plant-mPLoc: cell membrane, chloroplast, cytoplasm, mitochondrium, nucleus, peroxisome; WoLFPSORT: chloroplast; TargetP: mitochondrium	
52	2.74	Pp3c13_16130V1.1	Ribulose bisphosphate carboxylase small chain	Plant-mPLoc: chloroplast; TargetP: other	
53	2.77	Pp1s206_11V6.1	Photosystem I PsaH, reaction centre subunit VI	Plant-mPLoc: chloroplast; TargetP: secretory pathway	
54	2.78	Pp1s347_22V6.2	Maybe a thioredoxin-like protein	Plant-mPLoc: nucleus; TargetP: other	
55	2.81	Pp1s10_389V6.1	50S ribosomal protein, ribosomal protein L19	Plant-mPLoc: chloroplast; TargetP: chloroplast	
56	2.82	Pp3c23_6600V1.1	Thiamine-biosynthesis Thi4 protein	Plant-mPLoc: chloroplast, mitochondrium; TargetP: chloroplast	
57	1.34	Pp1s68_239V6.1	N-(5'phosphoribosyl) anthranilate isomerase (PRAI)	Plant-mPLoc: chloroplast, TargetP: other	
58	1.79	Pp1s3_512V6.1	60 S ribosomal protein, ribosomal protein L37e	Plant-mPLoc: chloroplast, mitochondrium, nucleus; TargetP: chloroplast	

59	K6	probably Pp1s59_196G2.1	Uncharacterized protein	Plant-mPLoc: nucleus; WoLFPSORT nucleus; TargetP: other	
60	K12	Pp1s433_10V2.1 / Pp3c19_20830V1.1	Pirin-like protein	Plant-mPLoc: cytoplasm; WoLFPSORT: chloroplast, vakuole, ER; TargetP: secretory pathway	HIP2; no split-YFP signal, SI Fig. 5
