# Supplementary material for: Holophytochrome-Interacting Proteins in Physcomitrella: Putative Actors in Phytochrome Cytoplasmic Signaling
Source: Front Plant Sci. 2016 May 12;7:613. doi: 10.3389/fpls.2016.00613 (PMC4867686; doi:10.3389/fpls.2016.00613)
Supplement: Supplementary file 2 [file Data_Sheet_2.ZIP › SI/SI HIP1.pdf]

Supplementary Material

Holophytochrome-interacting proteins in *Physcomitrella*: putative actors in phytochrome cytoplasmic signaling

Anna Lena Ermert, Katharina Mailliet, and Jon Hughes*

*** Correspondence:** jon.hughes@uni-giessen.de

HIP1 (Pp3c2_10320V1.1)

```
ATGTTTTCCGCTTGTGCTCTCAGAGTTTCTCCGATTCCTCACTTGTGCGACGTAATTGTGGGTGCTAAGAGTCTATTCGTG
AGGAGGGATTTCGTGTCCCGTGTTCGATTTGCGGAGGTTGGATCTCGGCGTGGTAGGAGTTGGGTTGGTGTACGTGCCATG
GGTAGCGTTCCCGCTGATTTCTCTAAACCCCGGAAGGTTGTGAAGAAAGTCCTCTCTCAAGCGCAGCCCGAGGGCGATGGC
GCAACCGTGCGACGAAGCATTGGCAGGCCGGAGTTGAAGCAGCTGGACCCTTTCCTGCTTCTAGACTATTTTGAAGCTTCT
GGGCCCGCTGGTTTTCCCGATCATCCTCATCGAGGATTTGAGACCGTCACGTACATGCTTCAGGGGTCCTTTACGCATGAA
GATTTTGCGGGTCATAAGGGCATTCTCGAAGCCGGTGACGTTCAGTGGATGACAGCTGGACGTGGAATAGTACACAGCGAA
ATGCCGTCGCCAGTAGGAGTTCAGAGGGGTCTGCAATTGTGGGTCAATTTGGCAGGAAAAGATAAAATGATTGAGCCAAAT
TACCAAGAACTAAAAGCTAAAGACATACCGAGGATTGAAAAAGATGGCGTGGAAGTTGTCATAATTGCTGGGGAATCTTTT
GGCGTCAAGTCACCGGTGTACACTCGCACCCCAACCATGTACTTGGATTTCTACCTACAGCCAGGCGCCTCGTTACACCAA
GCCATTCCCGAAGGATGGAACGCCTTCACCTTTGTCCTGAAGGGGAGCATGGTGTTTGGGAAGGAGGACGCACCACCAATT
GGTCCAAGTCATACAGTTGTCCTAAGTGATGGTGACGGTCTCAGTGCTTGGAACAAGGGCACAGAACCTGCTCAGTTTGTG
CTAGTAGGTGGGAAGCCTTTGAATGAACCTGTAGCGCAATACGGCCCCTTTGTGATGAATACTCAGGCGCAACTCATGGAA
GCTGTTAGAGACTATCAGTATGGTAAGAATGGGTTCGAAAGAGCTCACTCTTGGAGATCAGAGGCCAAGGCCCAGTCCACA
CCATAG
```

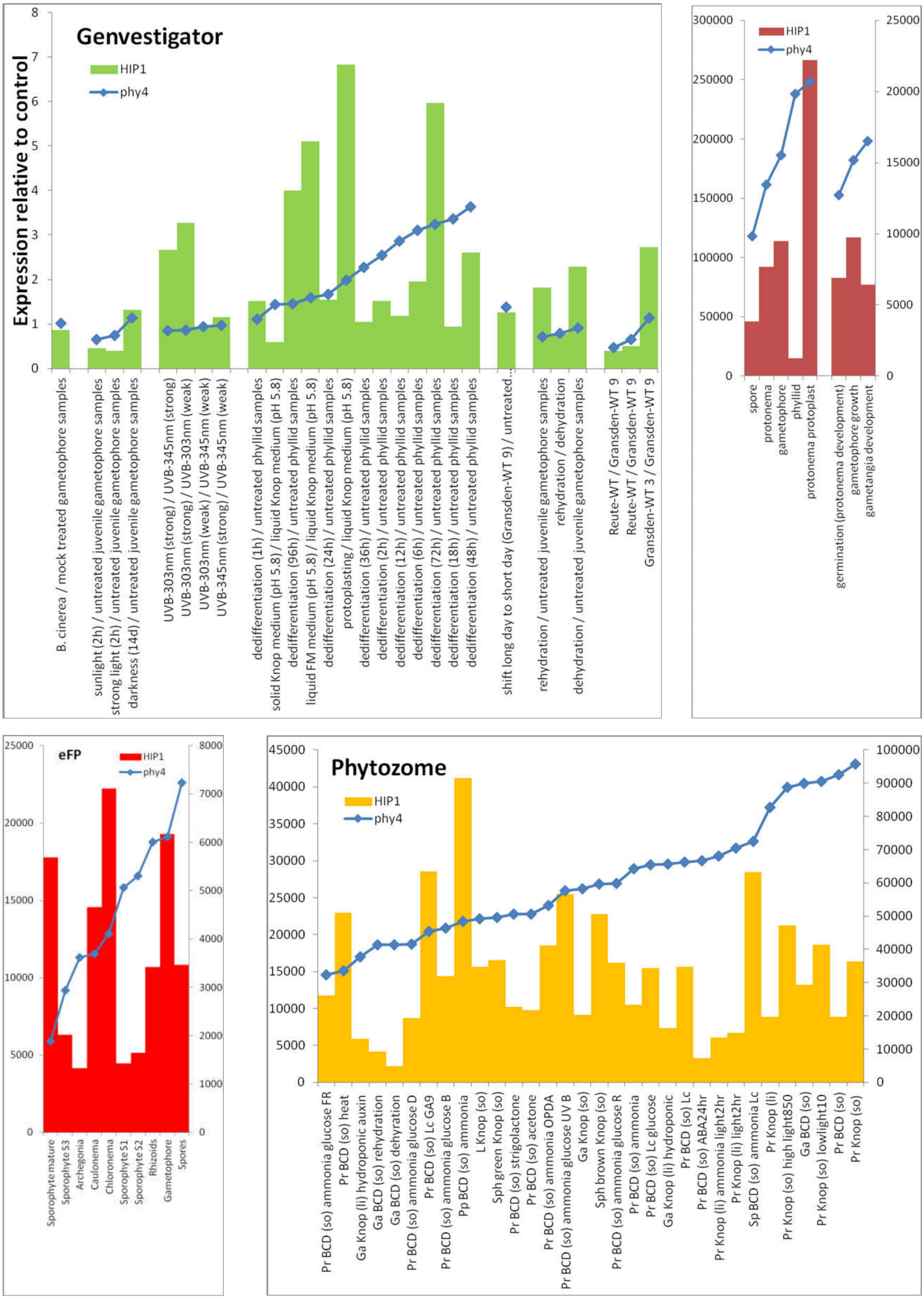
Genvestigator
HIP1
phy4
Expression relative to control
B. cinerea / mock treated gametophore samples
sunlight (2h) / untreated juvenile gametophore samples
strong light (2h) / untreated juvenile gametophore samples
darkness (14d) / untreated juvenile gametophore samples
UVB-303nm (strong) / UVB-345nm (strong)
UVB-303nm (strong) / UVB-303nm (weak)
UVB-303nm (weak) / UVB-345nm (weak)
UVB-345nm (strong) / UVB-345nm (weak)
dedifferentiation (1h) / untreated phyllid samples
solid Knop medium (pH 5.8) / liquid Knop medium (pH 5.8)
dedifferentiation (96h) / untreated phyllid samples
liquid FM medium (pH 5.8) / liquid Knop medium (pH 5.8)
dedifferentiation (24h) / untreated phyllid samples
protoplasting / liquid Knop medium (pH 5.8)
dedifferentiation (36h) / untreated phyllid samples
dedifferentiation (2h) / untreated phyllid samples
dedifferentiation (12h) / untreated phyllid samples
dedifferentiation (6h) / untreated phyllid samples
dedifferentiation (72h) / untreated phyllid samples
dedifferentiation (18h) / untreated phyllid samples
dedifferentiation (48h) / untreated phyllid samples
shift long day to short day (Gransden-WT 9) / untreated...
rehydration / untreated juvenile gametophore samples
rehydration / dehydration
dehydration / untreated juvenile gametophore samples
Reute-WT / Gransden-WT 9
Reute-WT / Gransden-WT 9
Gransden-WT 3 / Gransden-WT 9
HIP1
phy4
spore
protonema
gametophore
phyllid
protonema protoplast
germination (protonema development)
gametophore growth
gametangia development
eFP
HIP1
phy4
Sporophyte mature
Sporophyte S3
Archegonia
Caulonema
Chloronema
Sporophyte S1
Sporophyte S2
Rhizoids
Gametophore
Spores
Phytozome
HIP1
phy4
Pr BCD (so) ammonia glucose FR
Pr BCD (so) heat
Ga Knop (li) hydroponic auxin
Ga BCD (so) rehydration
Ga BCD (so) dehydration
Pr BCD (so) ammonia glucose D
Pr BCD (so) Lc GA9
Pr BCD (so) ammonia glucose B
Pp BCD (so) ammonia
L Knop (so)
Sph green Knop (so)
Pr BCD (so) strigolactone
Pr BCD (so) acetone
Pr BCD (so) ammonia OPDA
Pr BCD (so) ammonia glucose UV B
Ga Knop (so)
Sph brown Knop (so)
Pr BCD (so) ammonia glucose R
Pr BCD (so) ammonia
Pr BCD (so) Lc glucose
Ga Knop (li) hydroponic
Pr BCD (so) Lc
Pr BCD (so) ABA24hr
Pr Knop (li) ammonia light2hr
Pr Knop (li) light2hr
Sp BCD (so) ammonia Lc
Pr Knop (li)
Pr Knop (so) high light850
Ga BCD (so)
Pr Knop (so) lowlight10
Pr BCD (so)
Pr Knop (so)

HIP1 and Pp3c19_20830 (formerly HIP2) alignment tree

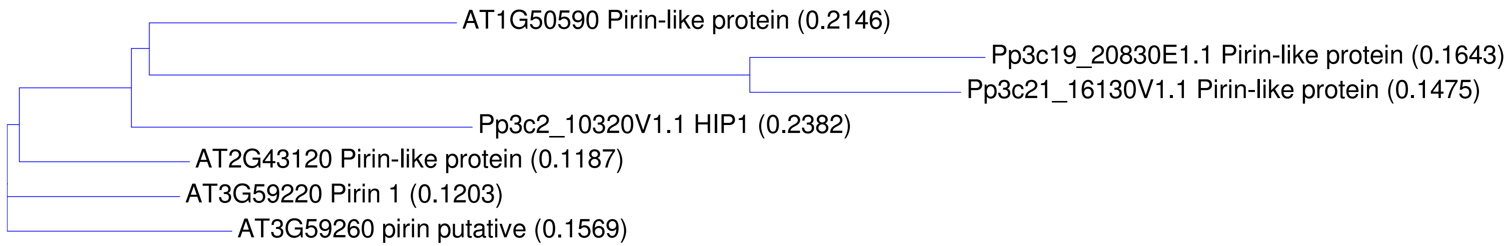

HIP1 and Pp3c19_20830 (formerly HIP2) alignment

```
                                       (1)   1       10        20        30        40        50        60            74
       AT1G50590 Pirin-like protein    (1)   --------------------------------------------------------------------------
Pp3c19_20830E1.1 Pirin-like protein    (1)   -------------------------------------------MCAFFLHLKSTPYFLILVLCVLYPFIMFFLS
Pp3c21_16130V1.1 Pirin-like protein    (1)   MRPQSVFRSVDLAFSTFGVKGWWRKIGVQLLPATSKGIFAARGLCNFHCELSASELCSSRGVKRIVPLVG--AS
             Pp3c2_10320V1.1 HIP1    (1)   -------------------------MFSACALRVSPIPHLCDVIVGAKSLFVRRDFVSRVRFAEVGSRRGRSWV
       AT2G43120 Pirin-like protein    (1)   -----------------------------------------------------MRAAINRANSLGGLFSFRFIR
                AT3G59220 Pirin 1    (1)   --------------------------------------------------------------------------
          AT3G59260 pirin putative    (1)   --------------------------------------------------------------------------
                          Consensus    (1)

                                      (75)   75   80        90        100       110       120       130            148
       AT1G50590 Pirin-like protein    (1)   ---MPISEKSSATNTRLVVKKLFARQLHEGFGAVVRRS--------IGRFEFRYFDPFLVLDEFSVSAPAGFPD
Pp3c19_20830E1.1 Pirin-like protein   (32)   RKTPPVAEKSVLLHPSQKMRHIPAKCLHVSTPTWWLESRFHFSFAEYYNPANQEFGVLRVLNDDLVKPQAGFGT
Pp3c21_16130V1.1 Pirin-like protein   (73)   AREAFTSSSSPQSSEIMTMRHIPANSLYVSTPTWWLESRFHFSFAEYYNPANSEFGVLRVLNDDLVKPKAGFGT
             Pp3c2_10320V1.1 HIP1   (50)   GVRAMGSVPADFSKPRKVVKKVLSQAQPEGDGATVRRS--------IGRPELKQLDPFLLLDYFEASGPAGFPD
       AT2G43120 Pirin-like protein   (22)   NIKSMSSSTSQDFVSRPVIKKVFAKLQKEGDGAVVRRG--------ISRSEQKLLDPFLMLDEFSVSPPAGFPD
                AT3G59220 Pirin 1    (1)   ------MTYENNSVPRIVIKKVLAKLEKEGEGAVVRNG--------ITKIDQKLLDPFVLLVEFSFSLSAGFPD
          AT3G59260 pirin putative    (1)   ------------------MKVMVLSLGKASP----------------SKSDHELLDPFVSLVEFSVSPPGGFKD
                          Consensus   (75)         S  S  S  R VMKKV AK   EG GA VR S        I R ENK LDPFLVL EFSVSPPAGFPD

                                     (149)   149        160       170       180       190       200       210      222
       AT1G50590 Pirin-like protein   (64)   HPHRGFETVTYMLEGEILHEDCEGHKGVIREGGLQWMTAGKGIVHSEMPSSNSNGITHNKGLQLWINLSSRQKL
Pp3c19_20830E1.1 Pirin-like protein  (106)   HGHRDMEIFTYIVDGKLTHRDSIGTSETLGRGSVQYMSAGTGIRHSEMNNGD----DLLRFLQIWIKPDRYGLK
Pp3c21_16130V1.1 Pirin-like protein  (147)   HSHRDMEIFTYVVQGNLTHKDSIGTSETLGRGAVQYMSAGSGIRHSEMNSSD----ELLRFLQIWIKPNTRGLK
             Pp3c2_10320V1.1 HIP1  (116)   HPHRGFETVTYMLQGSFTHEDFAGHKGILEAGDVQWMTAGRGIVHSEMPSPVG----VQRGLQLWVNLAGKDKM
       AT2G43120 Pirin-like protein   (88)   HPHRGFETVTYVLEGGITHQDFKGHKGTIYAGDVQWMTAGRGIIHSEMPEEE-----VNKGLQLWINLSSNEKM
                AT3G59220 Pirin 1   (61)   HPHRGFESVTYMLQGGIIHKDPKGHKGTIQAGDVQWMTAGRGIIHSEFPEEE-----VNNGLQLWINLPSTEKM
          AT3G59260 pirin putative   (41)   HPHRGFESVTYMFQGGIIHQDCNGNKGTIHEGDVQWMTAGRGIIHSEMPEEQ-----VNKGLQLWINLPSSAKM
                          Consensus  (149)   HPHRGFETVTYMLQG ITH D  GHKGTI  GDVQWMTAGRGIIHSEMP  D     VNKGLQLWINL S  KM
```

Pirin, N-terminal domain

RmlC-like cupin

```
                                      (223) 223    230                 240                 250                 260                 270                 280                       296
       AT1G50590 Pirin-like protein (138) VEPSYQEIESKD-IAETEKDGVRVRVIAGEWNGVKSKICTRTPTMYLDFTLSPGSRISQPIPLHWNAFVYVLQG
Pp3c19_20830E1.1 Pirin-like protein (176) PNYGSRVFKKDDRHNKLQHVLTDFKRYESEKDAGEGVIPIHQDCNIYVSEADAGVVQDFVLAKKRQAYMVCIEG
Pp3c21_16130V1.1 Pirin-like protein (217) PNYGSRIFNKDDRHNKIQHVLTDFNKIEGEKDSGQGVIPIHQDCNMYVSEADPGVAQDFVLSKNRQAYLVCIEG
             Pp3c2_10320V1.1 HIP1 (186) IEPNYQELKAKD-IPRIEKDGVEVVIIAGESFGVKSPVYTRTPTMYLDFYLQPGASLHQAIPEGWNAFTFVLKG
       AT2G43120 Pirin-like protein (157) IEPNYQELSHSD-IPKAEQNGVEVKVIAGESMGIQSPVYTRTPTMFLDFTLQPGAQIHQNVPESWNAFAYILES
                AT3G59220 Pirin 1 (130) TEPKYKELSSLD-IPRAEENGVEVKVIAGDSMGIKSPVYTRTPTMFLDFTLKPGSQTHQTVPESWTAFAYIIEG
         AT3G59260 pirin putative (110) IEPKNIEISSSE-IPSADDYGVEVKVIAGESMGVKSPFYTKTPIMFLDFTLDPKAQTHQAVPESWTAFAYIVEG
                        Consensus (223) IEP Y EL S D IPK E  GVEVKVIAGES GVKSPIYTRTPTMFLDFTL PGA  HQ VPE WNAF YIIEG

                                      (297) 297                     310                 320                 330                 340                 350                 360           370
       AT1G50590 Pirin-like protein (211) -HGHFGDSKLQHSAAAAHHLLVLGLGGDMLEAWNGSDSGLPLRFILVAGEPIGEPMVQFGPFVMNTQEEIDETI
Pp3c19_20830E1.1 Pirin-like protein (250) -------------KLSLSEKVQLDFR-DAVEITAGAVEDLPLKLKADENVGAHYIIIEMALA------------
Pp3c21_16130V1.1 Pirin-like protein (291) -------------KLSVSDLVNLDTR-DAVEIRAKTSEDMLLQLKADKDVGAHFLLIEMALG------------
             Pp3c2_10320V1.1 HIP1 (259) --S-MVFGKEDAPPIGPSHTVVLSDG-DGLSAWNKGTE--PAQFVLVGGKPLNEPVAQYGPFVMNTQAQLMEAV
       AT2G43120 Pirin-like protein (230) GEGGGVFSSSNSSPIPAHSVVVFGPGNDGVSVWNKSSS-KQLRFVLIAGEPIGEPVVQYGPFVMNTQAEIDMTI
                AT3G59220 Pirin 1 (203) DEG--VFGSLNSSAISAHHVVVFGPG-DLVSVWNKSTS-RSLRFLLIAGEPIGEPVVQCGPFVMNSQAEIDMAF
         AT3G59260 pirin putative (183) DEG--VFSSSDSSTVQAHNVVVFGTG-DEVSVWNTSNS-RPLRFLLIAGEPIGEPVVQHGPFVMNSQDEIEMTI
                        Consensus (297)   G  VF    SS ISAH VVVLG G DAVSVWNKSSS  PLRFLLIAGEPIGEPVVQ GPFVMNTQ EID  I

                                      (371) 371      380                    397
       AT1G50590 Pirin-like protein (284) DDFENFRNGFEKARHWKSQAASALGLF
Pp3c19_20830E1.1 Pirin-like protein (298) ---------------------------
Pp3c21_16130V1.1 Pirin-like protein (339) ---------------------------
             Pp3c2_10320V1.1 HIP1 (327) RDYQYGKNGFERAHSWRSEAKAQSTP-
       AT2G43120 Pirin-like protein (303) EDYHYGKNGFEMAKYWRSQ--------
                AT3G59220 Pirin 1 (273) DDYQNAKNGFEMAKC------------
         AT3G59260 pirin putative (253) GDYRNGMNGFEMAKHWRSE--------
                        Consensus (371)  DY  GKNGFE AK WRS
```

Pirin, C-terminal domain

RmlC-like cupin
