# Supplementary material for: Holophytochrome-Interacting Proteins in Physcomitrella: Putative Actors in Phytochrome Cytoplasmic Signaling
Source: Front Plant Sci. 2016 May 12;7:613. doi: 10.3389/fpls.2016.00613 (PMC4867686; doi:10.3389/fpls.2016.00613)
Supplement: Supplementary file 2 [file Data_Sheet_2.ZIP › SI/SI HIP11.pdf]

frontiers

Supplementary Material

Holophytochrome-interacting proteins in Physcomitrella: putative actors in phytochrome cytoplasmic signaling

Anna Lena Ermert, Katharina Mailliet, and Jon Hughes*

*** Correspondence:** jon.hughes@uni-giessen.de

HIP11 (Pp3c20_16210V1.1)

```
ATGAAGAAGACTACGTGTTTCAAGCTGCTGCTGGTCATTGCTGCGTTGGTGGTCACCACGCTGCCAGCTGCAGTCGAAGCT
GTGGATATCCAGGGCTTCGACAATTCTGCTGCCTGTACGGGCACAGGATACACCTTCCCAGGAATTGCGCAGCGAACTTGT
GCGGCGTTCACGAATGAAGGATCCATTTTGATCAGAGACCTTTCCAGCTGTCAGACCGGCAGAGCTTACCGCAACGGAGGA
TGCACGACTGAGGTGGGAAATGGGAATGGCCCGACTGTGTGGTGCTTCGTCGGTGGCTCATACACAGGAGCGGCCTGGTTC
AATAACTGCAGAAGACGTCGACTTCAGGCGGACAACAGCCACGAATCCTGCACATCTACCTCTGCTCCCAACGGTGTCCAC
TACACTGAGCACCTCTCCAAGGGATCATGGATCCTGCACTCTGACAATGCCACAGAACTCATGGCCGAACTCAAAAAAATT
GTCGATGAAGAGAAAGTGAACTGGCTCAAAGCTCGCGGTGCCTATCTTATTCCTGGGAGCGACACAATTGAATACGTCTAA
```

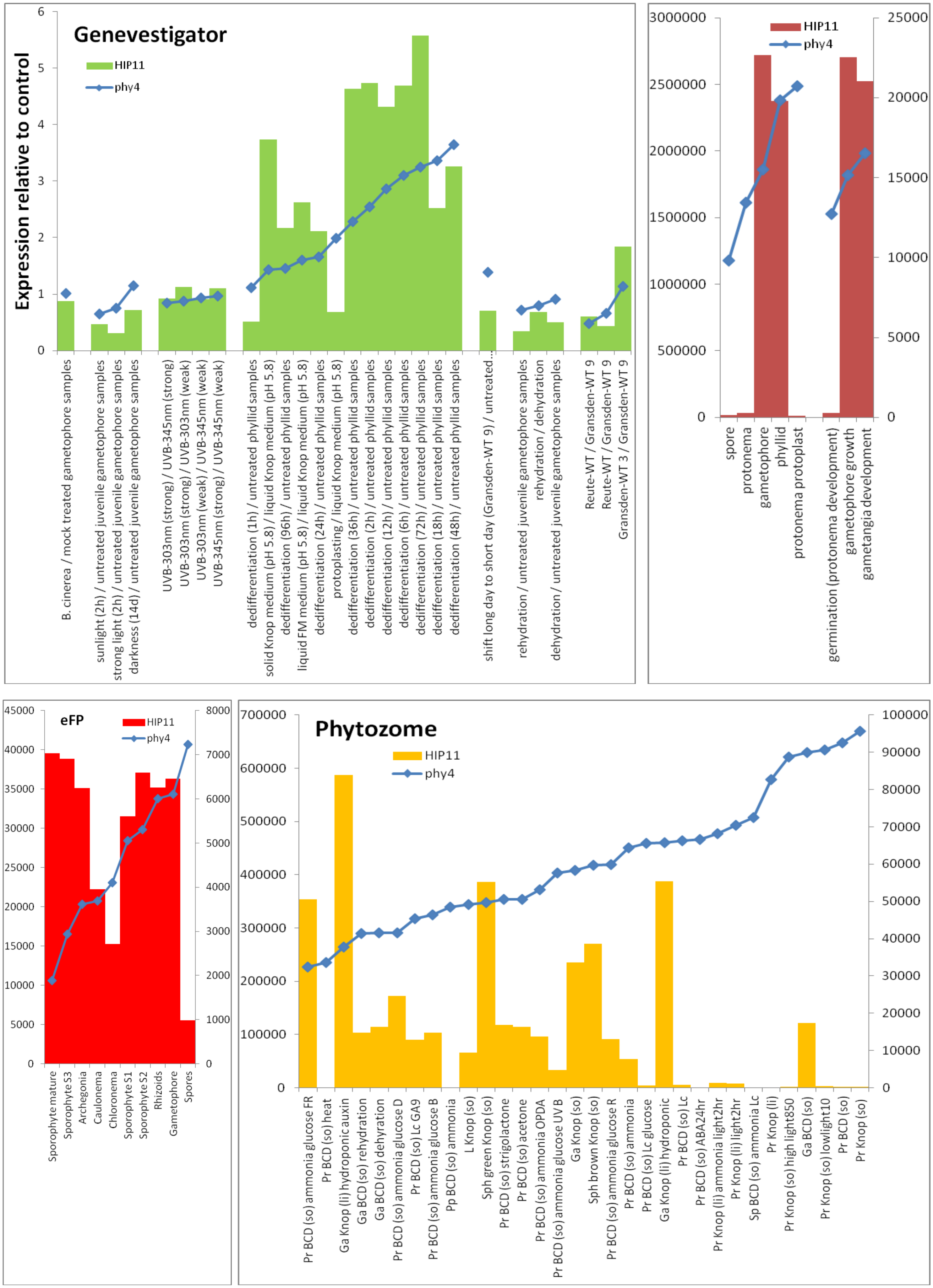
Genevestigator
Expression relative to control
HIP11
phy4
B. cinerea / mock treated gametophore samples
sunlight (2h) / untreated juvenile gametophore samples
strong light (2h) / untreated juvenile gametophore samples
darkness (14d) / untreated juvenile gametophore samples
UVB-303nm (strong) / UVB-345nm (strong)
UVB-303nm (strong) / UVB-303nm (weak)
UVB-303nm (weak) / UVB-345nm (weak)
UVB-345nm (strong) / UVB-345nm (weak)
dedifferentiation (1h) / untreated phyllid samples
solid Knop medium (pH 5.8) / liquid Knop medium (pH 5.8)
dedifferentiation (96h) / untreated phyllid samples
liquid FM medium (pH 5.8) / liquid Knop medium (pH 5.8)
dedifferentiation (24h) / untreated phyllid samples
protoplasting / liquid Knop medium (pH 5.8)
dedifferentiation (36h) / untreated phyllid samples
dedifferentiation (2h) / untreated phyllid samples
dedifferentiation (12h) / untreated phyllid samples
dedifferentiation (6h) / untreated phyllid samples
dedifferentiation (72h) / untreated phyllid samples
dedifferentiation (18h) / untreated phyllid samples
dedifferentiation (48h) / untreated phyllid samples
shift long day to short day (Gransden-WT 9) / untreated...
rehydration / untreated juvenile gametophore samples
rehydration / dehydration
dehydration / untreated juvenile gametophore samples
Reute-WT / Gransden-WT 9
Reute-WT / Gransden-WT 9
Gransden-WT 3 / Gransden-WT 9
HIP11
phy4
spore
protonema
gametophore
phyllid
protonema protoplast
germination (protonema development)
gametophore growth
gametangia development
eFP
HIP11
phy4
Sporophyte mature
Sporophyte S3
Archegonia
Caulonema
Chloronema
Sporophyte S1
Sporophyte S2
Rhizoids
Gametophore
Spores
Phytozome
HIP11
phy4
Pr BCD (so) ammonia glucose FR
Pr BCD (so) heat
Ga Knop (li) hydroponic auxin
Ga BCD (so) rehydration
Ga BCD (so) dehyration
Pr BCD (so) ammonia glucose D
Pr BCD (so) Lc GA9
Pr BCD (so) ammonia glucose B
Pp BCD (so) ammonia
L Knop (so)
Sph green Knop (so)
Pr BCD (so) strigolactone
Pr BCD (so) acetone
Pr BCD (so) ammonia OPDA
Pr BCD (so) ammonia glucose UV B
Ga Knop (so)
Sph brown Knop (so)
Pr BCD (so) ammonia glucose R
Pr BCD (so) ammonia
Pr BCD (so) Lc glucose
Ga Knop (li) hydroponic
Pr BCD (so) Lc
Pr BCD (so) ABA24hr
Pr Knop (li) ammonia light2hr
Pr Knop (li) light2hr
Sp BCD (so) ammonia Lc
Pr Knop (li)
Pr Knop (so) high light850
Ga BCD (so)
Pr Knop (so) lowlight10
Pr BCD (so)
Pr Knop (so)

HIP11 alignment

transmembrane region

```
                                                       (1)  1       10        20        30        40        50            65
AT5G19150 pfkB-like carbohydrate kinase family protein (1)  -------MLVKPSIISGLVRLTSHSPSSSSSVLRRQEFLVRTLCGSPIIRAMSSTSEADAESVLR
                            Pp3c20_16210V1.1 HIP11 (1)  MKKTTCFKLLLVIAALVVTTLPAAVEAVDIQGFDNSAACTGTGYTFPGIAQRTCAAFTNEGSILI
                                  Pp3c20_16230V1.1 (1)  MKKTTCFKLLLVIAALVVTTLPAAVEAVDIQGFDNSAACTGTGYTFPGIAQRTCAAFTNEGSILI
                                         Consensus (1)  MKKTTCFKLLLVIAALVVTTLPAAVEAVDIQGFDNSAACTGTGYTFPGIAQRTCAAFTNEGSILI

                                                      (66)  66            80        90        100       110       120      130
AT5G19150 pfkB-like carbohydrate kinase family protein (59)  TVTPSLDLKRHKGQAGKIAVIGG-------CR---EYTGAPYFAAISALKIGADLSHVFCTKDAA
                           Pp3c20_16210V1.1 HIP11 (66)  RDLSSCQTGRAYRNGGCTTEVGNGNGPTVWCFVGGSYTGAAWFNNCRRRRLQADNSHESCTSTSA
                                 Pp3c20_16230V1.1 (66)  RDLSSCQTGRAYRNGGCTTEVGNGNGPTVWCFVGGSYTGAAWFNNCRRRRLQADNSHESCTSTSA
                                        Consensus (66)  RDLSSCQTGRAYRNGGCTTEVGNGNGPTVWCFVGGSYTGAAWFNNCRRRRLQADNSHESCTSTSA

                                                     (131)  131      140       150       160       170       180            195
AT5G19150 pfkB-like carbohydrate kinase family protein (114) PVIKSYSPELIVHPVLEESYSISQLSEEDKREVQDKVLGEVGKWMERFDCLVIGPGLGRDPFLLE
                          Pp3c20_16210V1.1 HIP11 (131)  PNGVHYTEHLSKGSWILHSDNATELMAELKKIVDEEKV----NWLKARGAYLIPGSDTIEYV---
                                Pp3c20_16230V1.1 (131)  PNGVHYTEHLSKGSWILHSDNATELMAELKKIVDEEKV----NWLKARGAYLIPGSDTIEYV---
                                       Consensus (131)  PNGVHYTEHLSKGSWILHSDNATELMAELKKIVDEEKV    NWLKARGAYLIPGSDTIEYV

                                                     (196)  196           210       220       230       240       250      260
AT5G19150 pfkB-like carbohydrate kinase family protein (179) CVSIIMLLAKKSNVPFVIDGDGLFLVTNSIDLVHSYPLAVLTPNVNEYKRLVQKVLNCEVDEQNA
                          Pp3c20_16210V1.1 HIP11 (189)  -----------------------------------------------------------------
                                Pp3c20_16230V1.1 (189)  -----------------------------------------------------------------
                                       Consensus (196)

                                                     (261)  261      270       280       290       300       310            325
AT5G19150 pfkB-like carbohydrate kinase family protein (244) EDQLRSLAKQIGGVTILRKGKSDLISNGETVKSVSIYGSPRRCGGQGDILSGGVAVFLSWAQQLK
                          Pp3c20_16210V1.1 HIP11 (189)  -----------------------------------------------------------------
                                Pp3c20_16230V1.1 (189)  -----------------------------------------------------------------
                                       Consensus (261)
```

```
                                                  (326) 326           340       350       360       370        382
AT5G19150 pfkB-like carbohydrate kinase family protein (309) SDPESPSENPAILGCIAASGLLRKAASLAFTKHKRSTLTSDIIECLGESLEDICPAS
                          Pp3c20_16210V1.1 HIP11 (189) ---------------------------------------------------------
                                Pp3c20_16230V1.1 (189) ---------------------------------------------------------
                                       Consensus (326)
```
