# Supplementary material for: Holophytochrome-Interacting Proteins in Physcomitrella: Putative Actors in Phytochrome Cytoplasmic Signaling
Source: Front Plant Sci. 2016 May 12;7:613. doi: 10.3389/fpls.2016.00613 (PMC4867686; doi:10.3389/fpls.2016.00613)
Supplement: Supplementary file 2 [file Data_Sheet_2.ZIP › SI/SI HIP13.pdf]

Supplementary Material

Holophytochrome-interacting proteins in Physcomitrella: putative actors in phytochrome cytoplasmic signaling

Anna Lena Ermert, Katharina Mailliet, and Jon Hughes*

*** Correspondence:** jon.hughes@uni-giessen.de

HIP13/EF1α (Pp3c1_23670V1.1)

ATGGGTAAGGAGAAGGTGCACATCAATATCGTGGTCATCGGCCATGTCGACTCTGGCAAGTCGACGACCACTGGGCACCTC
ATCTACAAGTTGGGAGGTATCGACAAGCGTGTGATCGAGCGTTTCGAGAAGGAGGCGGCGGAGATGAACAAGCGTTCGTTC
AAGTATGCGTGGGTGCTGGACAAGCTGAAGGCAGAGCGCGAGCGTGGTATCACGATTGATATTGCGCTGTGGAAGTTCGAG
ACCATGAAGTACTACTGCACTGTGATCGACGCGCCCGGACATCGCGATTTCATCAAGAACATGATCACGGGAACGTCGCAG
GCAGACTGCGCTGTGCTGATCATCGACTCGACGACGGGAGGGTTCGAGGCCGGTATCTCGAAGGATGGGCAGACGCGTGAG
CACGCACTGCTGGCGTTCACTTTGGGAGTGAAGCAGATGATTTGCTGCTGCAACAAGATGGACGCGACCACCCCGAAGTAC
AGCAAGGCCCGATATGAGGAGATCGTGAAGGAGGTGTCGGCGTACCTGAAGAAGGTGGGGTACAACCCGGAGAAGGTTCCG
TTCGTGCCGATCTCAGGGTTCGAGGGCGACAACATGATCGAGAGGTCGACGAACCTGGACTGGTACAAGGGCCCGACTCTG
TTGGAGGCGTTGGACAACGTGTCAGAGCCGAAGCGGCCATCTGACAAGCCTCTGAGGCTGCCGCTTCAGGACGTGTACAAG
ATTGGAGGCATCGGAACTGTGCCAGTGGGACGGGTGGAGACAGGAATCATCAGGCCAGGTATGTTGGTGTGCTTCGCACCG
ACGGGACTGACGACTGAGGTGAAGTCGGTGGAGATGCATCACGAGTCGATGCCTGAGGCGCACCCAGGTGACAACGTCGGG
TTCAACGTGAAGAACGTGGCGGTGAAGGACCTGAAGAGAGGGTACGTGGCCTCAGACTCGAAGAACGACCCTGCCAAGGAG
GCAGCCAACTTCACGGCGCAGGTTATCATCATGAACCACCCGGGACAGATCGGGAACGGTTACGCACCAGTGCTGGATTGC
CACACGTCGCACATTGCGGTGAAGTTCGCGGAGATCTTGACGAAGGTGGACAGGCGATCGGGTAAGGAGCTGGAGAAGGAG
CCGAAGTTTTTGAAGAACGGGGACGCAGGGTTCGTGAAGATGATTCCGACGAAGCCGATGACAGTGGAGACGTTCGCGGAG
TACCCACCATTGGGACGTTTCGCGGTGCGTGATATGAGGCAGACGGTGGCTGTGGGTGTTATCAAGGCCGTAGAGAAGAAG
GAACCCACCGGCGCCAAGGTGACGAAGGCCGCTGCGAAGAAGAAGTGA

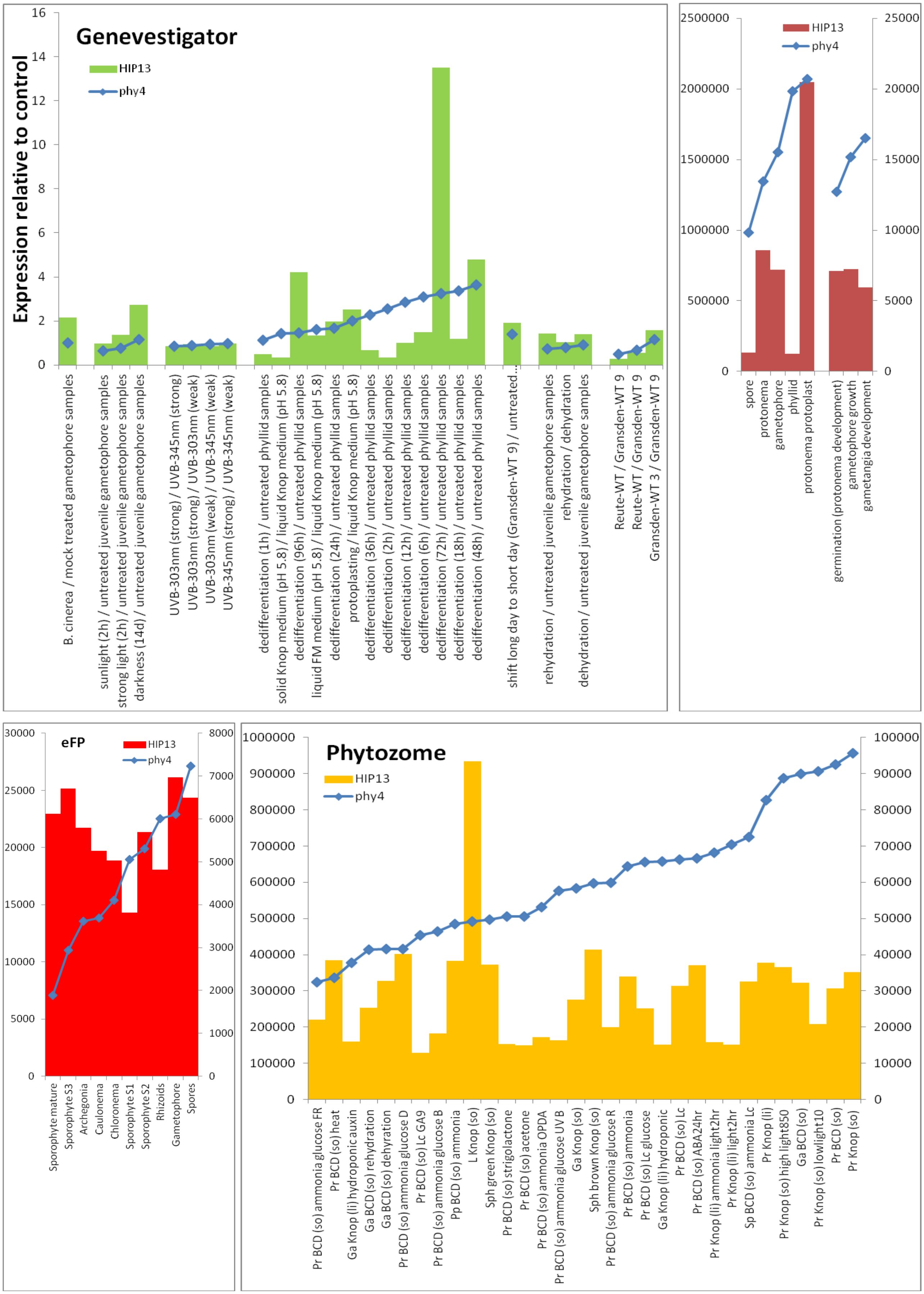
Genevestigator
Expression relative to control
HIP13
phy4
B. cinerea / mock treated gametophore samples
sunlight (2h) / untreated juvenile gametophore samples
strong light (2h) / untreated juvenile gametophore samples
darkness (14d) / untreated juvenile gametophore samples
UVB-303nm (strong) / UVB-345nm (strong)
UVB-303nm (strong) / UVB-303nm (weak)
UVB-303nm (weak) / UVB-345nm (weak)
UVB-345nm (strong) / UVB-345nm (weak)
dedifferentiation (1h) / untreated phyllid samples
solid Knop medium (pH 5.8) / liquid Knop medium (pH 5.8)
dedifferentiation (96h) / untreated phyllid samples
liquid FM medium (pH 5.8) / liquid Knop medium (pH 5.8)
dedifferentiation (24h) / untreated phyllid samples
protoplasting / liquid Knop medium (pH 5.8)
dedifferentiation (36h) / untreated phyllid samples
dedifferentiation (2h) / untreated phyllid samples
dedifferentiation (12h) / untreated phyllid samples
dedifferentiation (6h) / untreated phyllid samples
dedifferentiation (72h) / untreated phyllid samples
dedifferentiation (18h) / untreated phyllid samples
dedifferentiation (48h) / untreated phyllid samples
shift long day to short day (Gransden-WT 9) / untreated...
rehydration / untreated juvenile gametophore samples
rehydration / dehydration
dehydration / untreated juvenile gametophore samples
Reute-WT / Gransden-WT 9
Reute-WT / Gransden-WT 9
Gransden-WT 3 / Gransden-WT 9
HIP13
phy4
spore
protonema
gametophore
phyllid
protonema protoplast
germination (protonema development)
gametophore growth
gametangia development
eFP
HIP13
phy4
Sporophyte mature
Sporophyte S3
Archegonia
Caulonema
Chloronema
Sporophyte S1
Sporophyte S2
Rhizoids
Gametophore
Spores
Phytozome
HIP13
phy4
Pr BCD (so) ammonia glucose FR
Pr BCD (so) heat
Ga Knop (li) hydroponic auxin
Ga BCD (so) rehydration
Ga BCD (so) dehyration
Pr BCD (so) ammonia glucose D
Pr BCD (so) Lc GA9
Pr BCD (so) ammonia glucose B
Pp BCD (so) ammonia
L Knop (so)
Sph green Knop (so)
Pr BCD (so) strigolactone
Pr BCD (so) acetone
Pr BCD (so) ammonia OPDA
Pr BCD (so) ammonia glucose UV B
Ga Knop (so)
Sph brown Knop (so)
Pr BCD (so) ammonia glucose R
Pr BCD (so) ammonia
Pr BCD (so) Lc glucose
Ga Knop (li) hydroponic
Pr BCD (so) Lc
Pr BCD (so) ABA24hr
Pr Knop (li) ammonia light2hr
Pr Knop (li) light2hr
Sp BCD (so) ammonia Lc
Pr Knop (li)
Pr Knop (so) high light850
Ga BCD (so)
Pr Knop (so) lowlight10
Pr BCD (so)
Pr Knop (so)

HIP13 / EF1A alignment tree

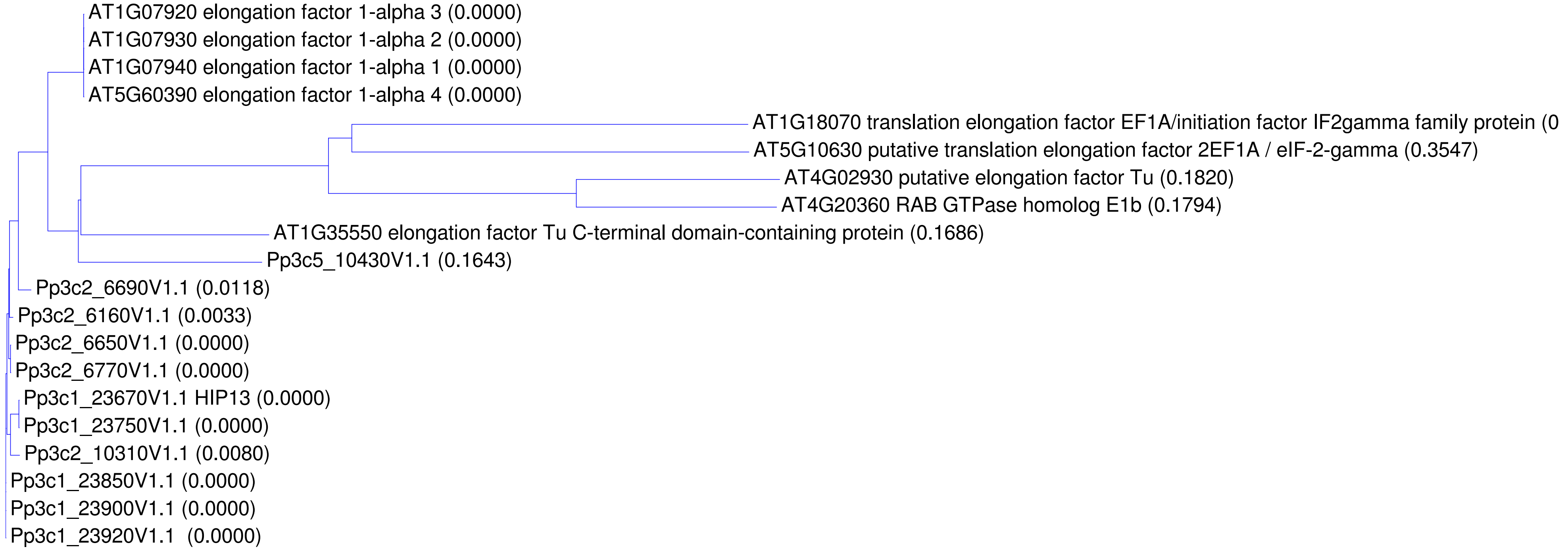

HIP13 / EF1A alignment

```
                                                          (1) 1       10        20        30        40        50            64
                AT1G07920 elongation factor 1-alpha 3     (1) ----------------------------------------------------------------
                AT1G07930 elongation factor 1-alpha 2     (1) ----------------------------------------------------------------
                AT1G07940 elongation factor 1-alpha 1     (1) ----------------------------------------------------------------
                AT5G60390 elongation factor 1-alpha 4     (1) ----------------------------------------------------------------
            AT1G18070 EF1A/IF2 gamma family protein       (1) ----------------------------------------------------------------
            AT5G10630 putative 2EF1A / eIF-2-gamma        (1) MPRKGLSNFDDYDDGFDDDDDAFDYDYDVDIDEHEEEAAAEPKEEIAKTQGLWRCAICTYDNVE
            AT4G02930 putative elongation factor Tu        (1) ----------------------------------------------------------------
                AT4G20360 RAB GTPase homolog E1b           (1) ----------------------------------------------------------------
AT1G35550 EF Tu C-terminal domain-containing protein       (1) ----------------------------------------------------------------
                                Pp3c5_10430V1.1           (1) ----------------------------------------------------------------
                                Pp3c2_6690V1.1            (1) ----------------------------------------------------------------
                                Pp3c2_6160V1.1            (1) ----------------------------------------------------------------
                                Pp3c2_6650V1.1            (1) ----------------------------------------------------------------
                                Pp3c2_6770V1.1            (1) ----------------------------------------------------------------
                          Pp3c1_23670V1.1 HIP13           (1) ----------------------------------------------------------------
                                Pp3c1_23750V1.1           (1) ----------------------------------------------------------------
                                Pp3c2_10310V1.1           (1) ----------------------------------------------------------------
                                Pp3c1_23850V1.1           (1) ----------------------------------------------------------------
                                Pp3c1_23900V1.1           (1) ----------------------------------------------------------------
                                Pp3c1_23920V1.1           (1) ----------------------------------------------------------------
                                      Consensus           (1)
```

Sequence	Start	65 … 128
	(65)	65 70 80 90 100 110 128
AT1G07920 elongation factor 1-alpha 3	(1)	--
AT1G07930 elongation factor 1-alpha 2	(1)	--
AT1G07940 elongation factor 1-alpha 1	(1)	--
AT5G60390 elongation factor 1-alpha 4	(1)	--
AT1G18070 EF1A/IF2 gamma family protein	(1)	--MDLE
AT5G10630 putative 2EF1A / eIF-2-gamma	(65)	TMFVCDICGVLRHPVAGNQSINKNTAPFKFDAPSPDDLVSNGLTSSKTGPKGSGDASMRQKEKQ
AT4G02930 putative elongation factor Tu	(1)	--
AT4G20360 RAB GTPase homolog E1b	(1)	--
AT1G35550 EF Tu C-terminal domain-containing protein	(1)	--
Pp3c5_10430V1.1	(1)	--
Pp3c2_6690V1.1	(1)	--
Pp3c2_6160V1.1	(1)	--
Pp3c2_6650V1.1	(1)	--
Pp3c2_6770V1.1	(1)	--
Pp3c1_23670V1.1 HIP13	(1)	--
Pp3c1_23750V1.1	(1)	--
Pp3c2_10310V1.1	(1)	--
Pp3c1_23850V1.1	(1)	--
Pp3c1_23900V1.1	(1)	--
Pp3c1_23920V1.1	(1)	--
Consensus	(65)	

```
                                                 (129) 129       140       150       160       170       180          192
AT1G07920 elongation factor 1-alpha 3              (1) ----------------------------------------------------------------
AT1G07930 elongation factor 1-alpha 2              (1) ----------------------------------------------------------------
AT1G07940 elongation factor 1-alpha 1              (1) ----------------------------------------------------------------
AT5G60390 elongation factor 1-alpha 4              (1) ----------------------------------------------------------------
AT1G18070 EF1A/IF2 gamma family protein            (5) AEIRALQLESADENNGVV----------IPEVHNSHEVENLDKAPEDLKDEVQESIPVPDEQEA
AT5G10630 putative 2EF1A / eIF-2-gamma           (129) DSVEQKPLKKGGDSSETSSRGRHDKLDDKGGAGGIKSGKSLPKAKADMSNETSSSSKYMETSES
AT4G02930 putative elongation factor Tu           (1) ------------------------------MASVVLRNPSSKRLVPFSSQIYSRCGASVTS---
AT4G20360 RAB GTPase homolog E1b                  (1) ----------------------------MAISAPAACSSSSRILCSYSSPSPSLCPAISTSGKL
AT1G35550 EF Tu C-terminal domain-containing protein (1) ----------------------------------------------------------------
Pp3c5_10430V1.1                                    (1) ----------------------------------------------------------------
Pp3c2_6690V1.1                                     (1) ----------------------------------------------------------------
Pp3c2_6160V1.1                                     (1) ----------------------------------------------------------------
Pp3c2_6650V1.1                                     (1) ----------------------------------------------------------------
Pp3c2_6770V1.1                                     (1) ----------------------------------------------------------------
Pp3c1_23670V1.1 HIP13                              (1) ----------------------------------------------------------------
Pp3c1_23750V1.1                                    (1) ----------------------------------------------------------------
Pp3c2_10310V1.1                                    (1) ----------------------------------------------------------------
Pp3c1_23850V1.1                                    (1) ----------------------------------------------------------------
Pp3c1_23900V1.1                                    (1) ----------------------------------------------------------------
Pp3c1_23920V1.1                                    (1) ----------------------------------------------------------------
Consensus                                        (129)
```

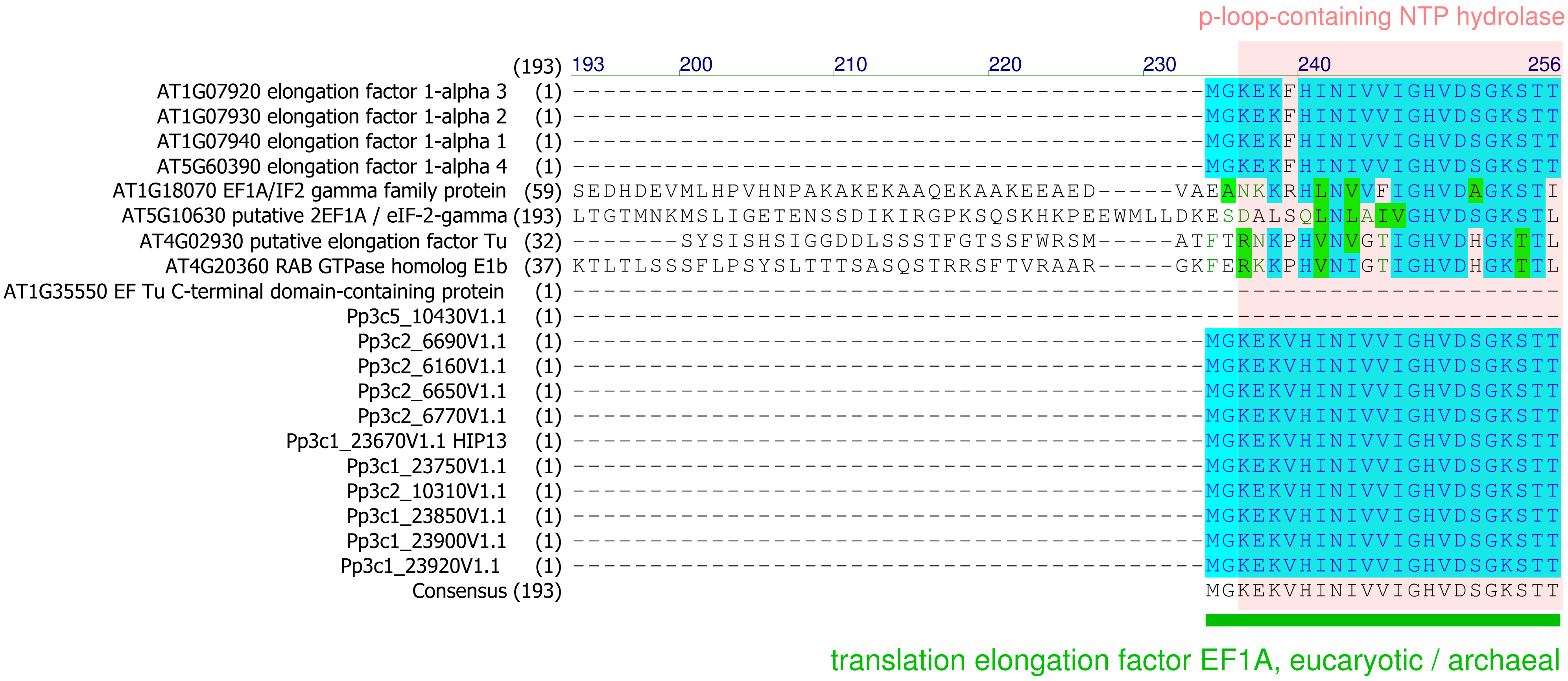
p-loop-containing NTP hydrolase
(193) 193 200 210 220 230 240 256
AT1G07920 elongation factor 1-alpha 3 (1) ---MGKEKFHINIVVIGHVDSGKSTT
AT1G07930 elongation factor 1-alpha 2 (1) ---MGKEKFHINIVVIGHVDSGKSTT
AT1G07940 elongation factor 1-alpha 1 (1) ---MGKEKFHINIVVIGHVDSGKSTT
AT5G60390 elongation factor 1-alpha 4 (1) ---MGKEKFHINIVVIGHVDSGKSTT
AT1G18070 EF1A/IF2 gamma family protein (59) SEDHDEVMLHPVHNPAKAKEKAAQEKAAKEEAED-----VAEANKKRHLNVVFIGHVDAGKSTI
AT5G10630 putative 2EF1A / eIF-2-gamma (193) LTGTMNKMSLIGETENSSDIKIRGPKSQSKHKPEEWMLLDKESDALSQLNLAIVGHVDSGKSTL
AT4G02930 putative elongation factor Tu (32) -------SYSISHSIGGDDLSSSTFGTSSFWRSM-----ATFTRNKPHVNVGTIGHVDHGKTTL
AT4G20360 RAB GTPase homolog E1b (37) KTLTLSSSFLPSYSLTTTSASQSTRRSFTVRAAR-----GKFERKKPHVNIGTIGHVDHGKTTL
AT1G35550 EF Tu C-terminal domain-containing protein (1) --
Pp3c5_10430V1.1 (1) --
Pp3c2_6690V1.1 (1) ---MGKEKVHINIVVIGHVDSGKSTT
Pp3c2_6160V1.1 (1) ---MGKEKVHINIVVIGHVDSGKSTT
Pp3c2_6650V1.1 (1) ---MGKEKVHINIVVIGHVDSGKSTT
Pp3c2_6770V1.1 (1) ---MGKEKVHINIVVIGHVDSGKSTT
Pp3c1_23670V1.1 HIP13 (1) ---MGKEKVHINIVVIGHVDSGKSTT
Pp3c1_23750V1.1 (1) ---MGKEKVHINIVVIGHVDSGKSTT
Pp3c2_10310V1.1 (1) ---MGKEKVHINIVVIGHVDSGKSTT
Pp3c1_23850V1.1 (1) ---MGKEKVHINIVVIGHVDSGKSTT
Pp3c1_23900V1.1 (1) ---MGKEKVHINIVVIGHVDSGKSTT
Pp3c1_23920V1.1 (1) ---MGKEKVHINIVVIGHVDSGKSTT
Consensus (193) MGKEKVHINIVVIGHVDSGKSTT
translation elongation factor EF1A, eucaryotic / archaeal

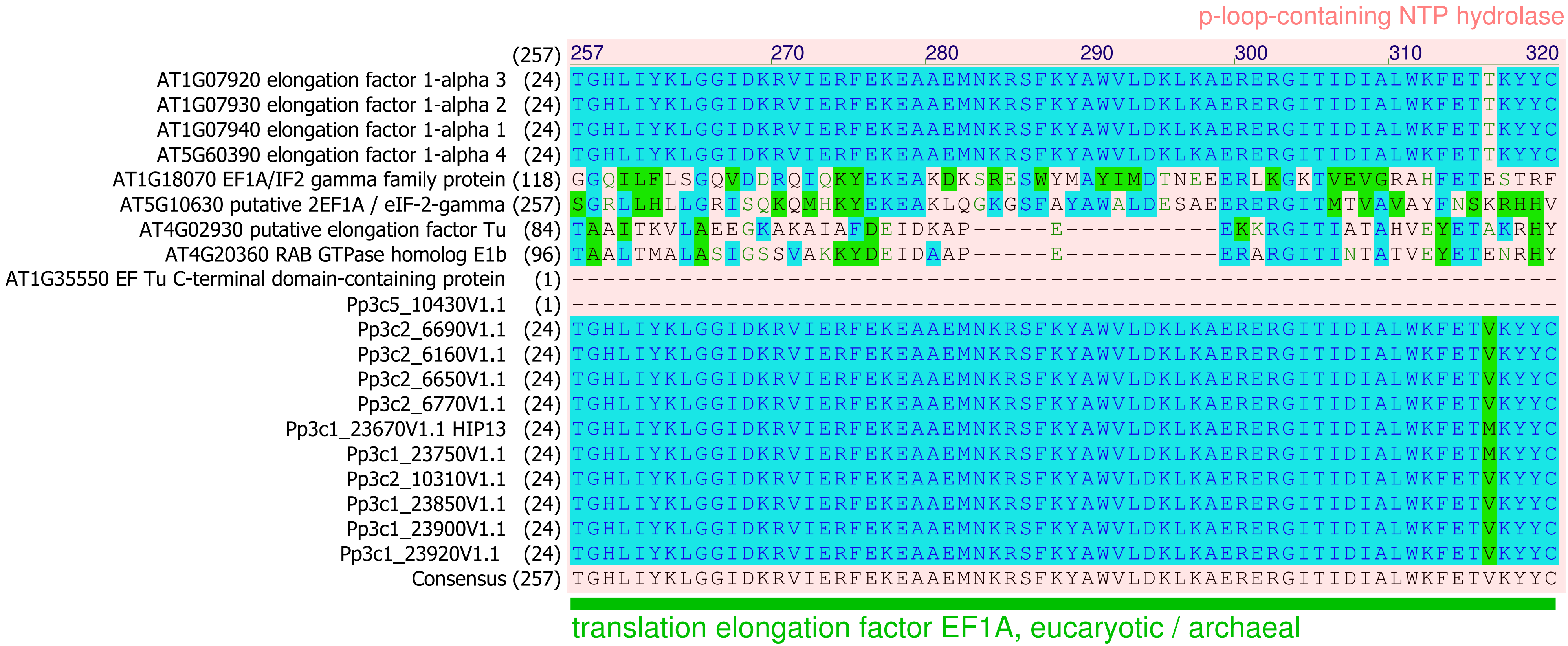
p-loop-containing NTP hydrolase
(257) 257 270 280 290 300 310 320
AT1G07920 elongation factor 1-alpha 3 (24) TGHLIYKLGGIDKRVIERFEKEAAEMNKRSFKYAWVLDKLKAERERGITIDIALWKFETTKYYC
AT1G07930 elongation factor 1-alpha 2 (24) TGHLIYKLGGIDKRVIERFEKEAAEMNKRSFKYAWVLDKLKAERERGITIDIALWKFETTKYYC
AT1G07940 elongation factor 1-alpha 1 (24) TGHLIYKLGGIDKRVIERFEKEAAEMNKRSFKYAWVLDKLKAERERGITIDIALWKFETTKYYC
AT5G60390 elongation factor 1-alpha 4 (24) TGHLIYKLGGIDKRVIERFEKEAAEMNKRSFKYAWVLDKLKAERERGITIDIALWKFETTKYYC
AT1G18070 EF1A/IF2 gamma family protein (118) GGQILFLSGQVDDRQIQKYEKEAKDKSRESWYMAYIMDTNEEERLKGKTVEVGRAHFETESTRF
AT5G10630 putative 2EF1A / eIF-2-gamma (257) SGRLLHLLGRISQKQMHKYEKEAKLQGKGSFAYAWALDESAEERERGITMTVAVAYFNSKRHHV
AT4G02930 putative elongation factor Tu (84) TAAITKVLAEEGKAKAIAFDEIDKAP-----E----------EKKRGITIATAHVEYETAKRHY
AT4G20360 RAB GTPase homolog E1b (96) TAALTMALASIGSSVAKKYDEIDAAP-----E----------ERARGITINTATVEYETENRHY
AT1G35550 EF Tu C-terminal domain-containing protein (1) --
Pp3c5_10430V1.1 (1) --
Pp3c2_6690V1.1 (24) TGHLIYKLGGIDKRVIERFEKEAAEMNKRSFKYAWVLDKLKAERERGITIDIALWKFETVKYYC
Pp3c2_6160V1.1 (24) TGHLIYKLGGIDKRVIERFEKEAAEMNKRSFKYAWVLDKLKAERERGITIDIALWKFETVKYYC
Pp3c2_6650V1.1 (24) TGHLIYKLGGIDKRVIERFEKEAAEMNKRSFKYAWVLDKLKAERERGITIDIALWKFETVKYYC
Pp3c2_6770V1.1 (24) TGHLIYKLGGIDKRVIERFEKEAAEMNKRSFKYAWVLDKLKAERERGITIDIALWKFETVKYYC
Pp3c1_23670V1.1 HIP13 (24) TGHLIYKLGGIDKRVIERFEKEAAEMNKRSFKYAWVLDKLKAERERGITIDIALWKFETMKYYC
Pp3c1_23750V1.1 (24) TGHLIYKLGGIDKRVIERFEKEAAEMNKRSFKYAWVLDKLKAERERGITIDIALWKFETMKYYC
Pp3c2_10310V1.1 (24) TGHLIYKLGGIDKRVIERFEKEAAEMNKRSFKYAWVLDKLKAERERGITIDIALWKFETVKYYC
Pp3c1_23850V1.1 (24) TGHLIYKLGGIDKRVIERFEKEAAEMNKRSFKYAWVLDKLKAERERGITIDIALWKFETVKYYC
Pp3c1_23900V1.1 (24) TGHLIYKLGGIDKRVIERFEKEAAEMNKRSFKYAWVLDKLKAERERGITIDIALWKFETVKYYC
Pp3c1_23920V1.1 (24) TGHLIYKLGGIDKRVIERFEKEAAEMNKRSFKYAWVLDKLKAERERGITIDIALWKFETVKYYC
Consensus (257) TGHLIYKLGGIDKRVIERFEKEAAEMNKRSFKYAWVLDKLKAERERGITIDIALWKFETVKYYC
translation elongation factor EF1A, eucaryotic / archaeal

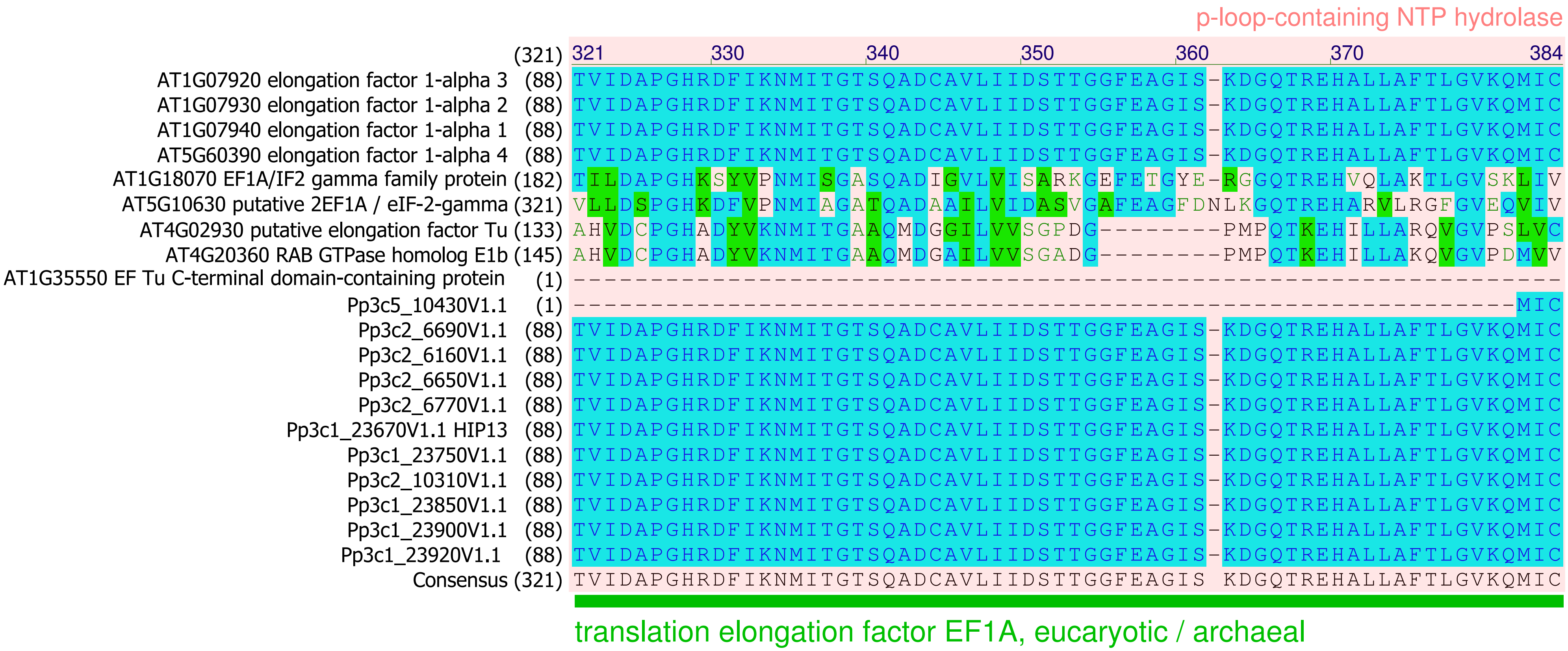

p-loop-containing NTP hydrolase
(321) 321 330 340 350 360 370 384
AT1G07920 elongation factor 1-alpha 3 (88) TVIDAPGHRDFIKNMITGTSQADCAVLIIDSTTGGFEAGIS-KDGQTREHALLAFTLGVKQMIC
AT1G07930 elongation factor 1-alpha 2 (88) TVIDAPGHRDFIKNMITGTSQADCAVLIIDSTTGGFEAGIS-KDGQTREHALLAFTLGVKQMIC
AT1G07940 elongation factor 1-alpha 1 (88) TVIDAPGHRDFIKNMITGTSQADCAVLIIDSTTGGFEAGIS-KDGQTREHALLAFTLGVKQMIC
AT5G60390 elongation factor 1-alpha 4 (88) TVIDAPGHRDFIKNMITGTSQADCAVLIIDSTTGGFEAGIS-KDGQTREHALLAFTLGVKQMIC
AT1G18070 EF1A/IF2 gamma family protein (182) TILDAPGHKSYVPNMISGASQADIGVLVISARKGEFETGYE-RGGQTREHVQLAKTLGVSKLIV
AT5G10630 putative 2EF1A / eIF-2-gamma (321) VLLDSPGHKDFVPNMIAGATQADAAILVIDASVGAFEAGFDNLKGQTREHARVLRGFGVEQVIV
AT4G02930 putative elongation factor Tu (133) AHVDCPGHADYVKNMITGAAQMDGGILVVSGPDG--------PMPQTKEHILLARQVGVPSLVC
AT4G20360 RAB GTPase homolog E1b (145) AHVDCPGHADYVKNMITGAAQMDGAILVVSGADG--------PMPQTKEHILLAKQVGVPDMVV
AT1G35550 EF Tu C-terminal domain-containing protein (1) --
Pp3c5_10430V1.1 (1) ---MIC
Pp3c2_6690V1.1 (88) TVIDAPGHRDFIKNMITGTSQADCAVLIIDSTTGGFEAGIS-KDGQTREHALLAFTLGVKQMIC
Pp3c2_6160V1.1 (88) TVIDAPGHRDFIKNMITGTSQADCAVLIIDSTTGGFEAGIS-KDGQTREHALLAFTLGVKQMIC
Pp3c2_6650V1.1 (88) TVIDAPGHRDFIKNMITGTSQADCAVLIIDSTTGGFEAGIS-KDGQTREHALLAFTLGVKQMIC
Pp3c2_6770V1.1 (88) TVIDAPGHRDFIKNMITGTSQADCAVLIIDSTTGGFEAGIS-KDGQTREHALLAFTLGVKQMIC
Pp3c1_23670V1.1 HIP13 (88) TVIDAPGHRDFIKNMITGTSQADCAVLIIDSTTGGFEAGIS-KDGQTREHALLAFTLGVKQMIC
Pp3c1_23750V1.1 (88) TVIDAPGHRDFIKNMITGTSQADCAVLIIDSTTGGFEAGIS-KDGQTREHALLAFTLGVKQMIC
Pp3c2_10310V1.1 (88) TVIDAPGHRDFIKNMITGTSQADCAVLIIDSTTGGFEAGIS-KDGQTREHALLAFTLGVKQMIC
Pp3c1_23850V1.1 (88) TVIDAPGHRDFIKNMITGTSQADCAVLIIDSTTGGFEAGIS-KDGQTREHALLAFTLGVKQMIC
Pp3c1_23900V1.1 (88) TVIDAPGHRDFIKNMITGTSQADCAVLIIDSTTGGFEAGIS-KDGQTREHALLAFTLGVKQMIC
Pp3c1_23920V1.1 (88) TVIDAPGHRDFIKNMITGTSQADCAVLIIDSTTGGFEAGIS-KDGQTREHALLAFTLGVKQMIC
Consensus (321) TVIDAPGHRDFIKNMITGTSQADCAVLIIDSTTGGFEAGIS KDGQTREHALLAFTLGVKQMIC
translation elongation factor EF1A, eucaryotic / archaeal

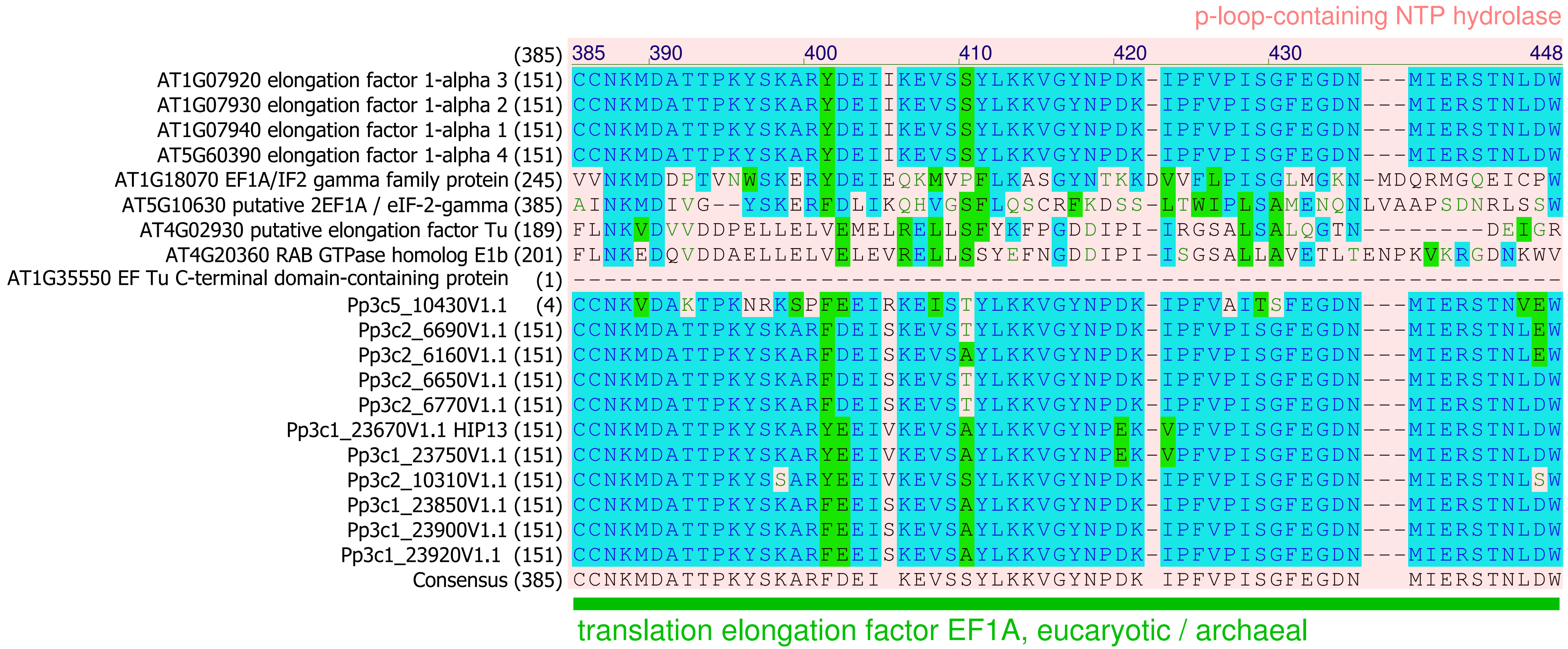
p-loop-containing NTP hydrolase
(385) 385 390 400 410 420 430 448
AT1G07920 elongation factor 1-alpha 3 (151) CCNKMDATTPKYSKARYDEIIKEVSSYLKKVGYNPDK-IPFVPISGFEGDN---MIERSTNLDW
AT1G07930 elongation factor 1-alpha 2 (151) CCNKMDATTPKYSKARYDEIIKEVSSYLKKVGYNPDK-IPFVPISGFEGDN---MIERSTNLDW
AT1G07940 elongation factor 1-alpha 1 (151) CCNKMDATTPKYSKARYDEIIKEVSSYLKKVGYNPDK-IPFVPISGFEGDN---MIERSTNLDW
AT5G60390 elongation factor 1-alpha 4 (151) CCNKMDATTPKYSKARYDEIIKEVSSYLKKVGYNPDK-IPFVPISGFEGDN---MIERSTNLDW
AT1G18070 EF1A/IF2 gamma family protein (245) VVNKMDDPTVNWSKERYDEIEQKMVPFLKASGYNTKKDVVFLPISGLMGKN-MDQRMGQEICPW
AT5G10630 putative 2EF1A / eIF-2-gamma (385) AINKMDIVG--YSKERFDLIKQHVGSFLQSCRFKDSS-LTWIPLSAMENQNLVAAPSDNRLSSW
AT4G02930 putative elongation factor Tu (189) FLNKVDVVDDPELLELVEMELRELLSFYKFPGDDIPI-IRGSALSALQGTN--------DEIGR
AT4G20360 RAB GTPase homolog E1b (201) FLNKEDQVDDAELLELVELEVRELLSSYEFNGDDIPI-ISGSALLAVETLTENPKVKRGDNKWV
AT1G35550 EF Tu C-terminal domain-containing protein (1) --
Pp3c5_10430V1.1 (4) CCNKVDAKTPKNRKSPFEEIRKEISTYLKKVGYNPDK-IPFVAITSFEGDN---MIERSTNVEW
Pp3c2_6690V1.1 (151) CCNKMDATTPKYSKARFDEISKEVSTYLKKVGYNPDK-IPFVPISGFEGDN---MIERSTNLEW
Pp3c2_6160V1.1 (151) CCNKMDATTPKYSKARFDEISKEVSAYLKKVGYNPDK-IPFVPISGFEGDN---MIERSTNLEW
Pp3c2_6650V1.1 (151) CCNKMDATTPKYSKARFDEISKEVSTYLKKVGYNPDK-IPFVPISGFEGDN---MIERSTNLDW
Pp3c2_6770V1.1 (151) CCNKMDATTPKYSKARFDEISKEVSTYLKKVGYNPDK-IPFVPISGFEGDN---MIERSTNLDW
Pp3c1_23670V1.1 HIP13 (151) CCNKMDATTPKYSKARYEEIVKEVSAYLKKVGYNPEK-VPFVPISGFEGDN---MIERSTNLDW
Pp3c1_23750V1.1 (151) CCNKMDATTPKYSKARYEEIVKEVSAYLKKVGYNPEK-VPFVPISGFEGDN---MIERSTNLDW
Pp3c2_10310V1.1 (151) CCNKMDATTPKYSSARYEEIVKEVSSYLKKVGYNPDK-IPFVPISGFEGDN---MIERSTNLSW
Pp3c1_23850V1.1 (151) CCNKMDATTPKYSKARFEEISKEVSAYLKKVGYNPDK-IPFVPISGFEGDN---MIERSTNLDW
Pp3c1_23900V1.1 (151) CCNKMDATTPKYSKARFEEISKEVSAYLKKVGYNPDK-IPFVPISGFEGDN---MIERSTNLDW
Pp3c1_23920V1.1 (151) CCNKMDATTPKYSKARFEEISKEVSAYLKKVGYNPDK-IPFVPISGFEGDN---MIERSTNLDW
Consensus (385) CCNKMDATTPKYSKARFDEI KEVSSYLKKVGYNPDK IPFVPISGFEGDN MIERSTNLDW
translation elongation factor EF1A, eucaryotic / archaeal

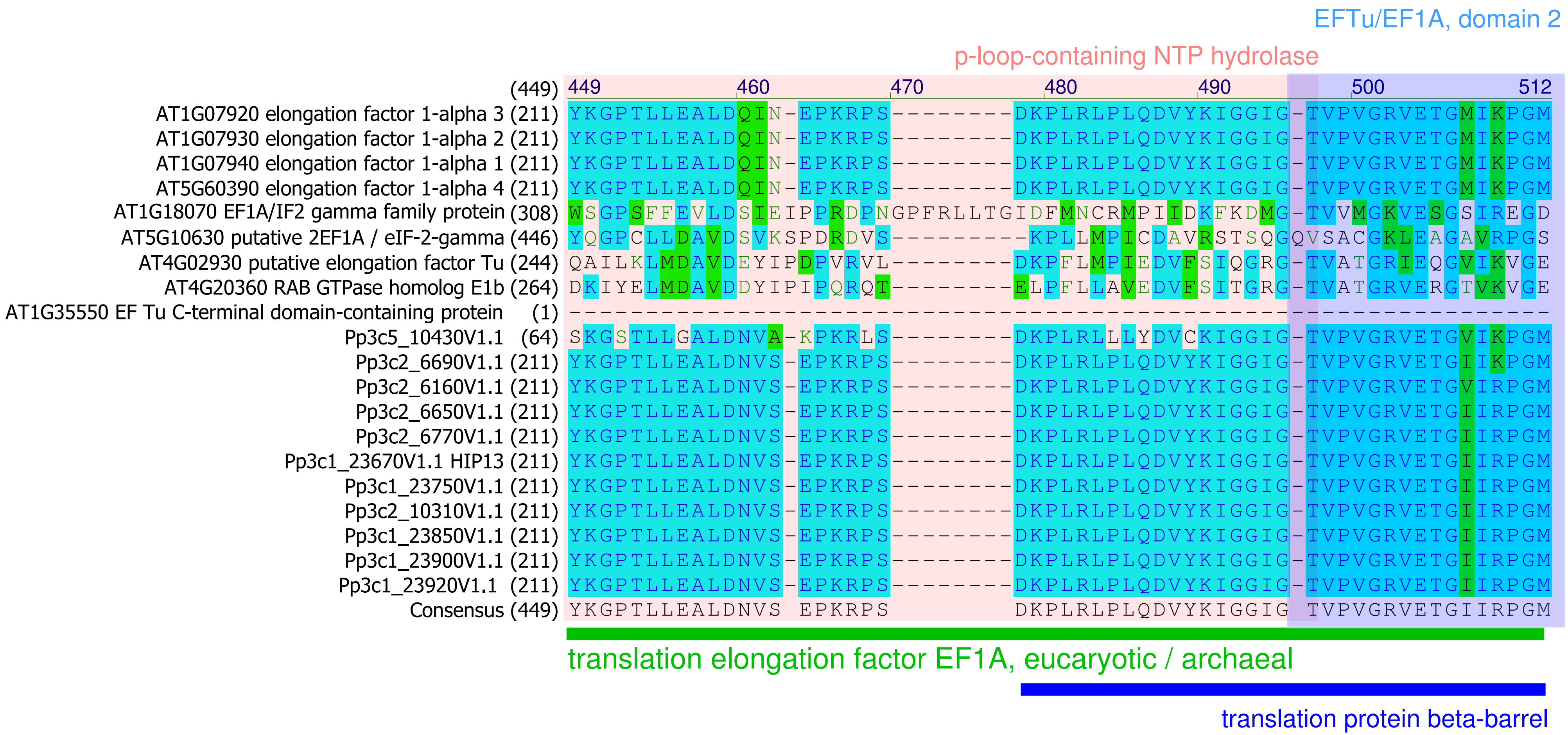

EFTu/EF1A, domain 2
p-loop-containing NTP hydrolase
(449) 449 460 470 480 490 500 512
AT1G07920 elongation factor 1-alpha 3 (211) YKGPTLLEALDQIN-EPKRPS--------DKPLRLPLQDVYKIGGIG-TVPVGRVETGMIKPGM
AT1G07930 elongation factor 1-alpha 2 (211) YKGPTLLEALDQIN-EPKRPS--------DKPLRLPLQDVYKIGGIG-TVPVGRVETGMIKPGM
AT1G07940 elongation factor 1-alpha 1 (211) YKGPTLLEALDQIN-EPKRPS--------DKPLRLPLQDVYKIGGIG-TVPVGRVETGMIKPGM
AT5G60390 elongation factor 1-alpha 4 (211) YKGPTLLEALDQIN-EPKRPS--------DKPLRLPLQDVYKIGGIG-TVPVGRVETGMIKPGM
AT1G18070 EF1A/IF2 gamma family protein (308) WSGPSFFEVLDSIEIPPRDPNGPFRLLTGIDFMNCRMPIIDKFKDMG-TVVMGKVESGSIREGD
AT5G10630 putative 2EF1A / eIF-2-gamma (446) YQGPCLLDAVDSVKSPDRDVS---------KPLLMPICDAVRSTSQGQVSACGKLEAGAVRPGS
AT4G02930 putative elongation factor Tu (244) QAILKLMDAVDEYIPDPVRVL--------DKPFLMPIEDVFSIQGRG-TVATGRIEQGVIKVGE
AT4G20360 RAB GTPase homolog E1b (264) DKIYELMDAVDDYIPIPQRQT--------ELPFLLAVEDVFSITGRG-TVATGRVERGTVKVGE
AT1G35550 EF Tu C-terminal domain-containing protein (1) --
Pp3c5_10430V1.1 (64) SKGSTLLGALDNVA-KPKRLS--------DKPLRLLLYDVCKIGGIG-TVPVGRVETGVIKPGM
Pp3c2_6690V1.1 (211) YKGPTLLEALDNVS-EPKRPS--------DKPLRLPLQDVYKIGGIG-TVPVGRVETGIIKPGM
Pp3c2_6160V1.1 (211) YKGPTLLEALDNVS-EPKRPS--------DKPLRLPLQDVYKIGGIG-TVPVGRVETGVIRPGM
Pp3c2_6650V1.1 (211) YKGPTLLEALDNVS-EPKRPS--------DKPLRLPLQDVYKIGGIG-TVPVGRVETGIIRPGM
Pp3c2_6770V1.1 (211) YKGPTLLEALDNVS-EPKRPS--------DKPLRLPLQDVYKIGGIG-TVPVGRVETGIIRPGM
Pp3c1_23670V1.1 HIP13 (211) YKGPTLLEALDNVS-EPKRPS--------DKPLRLPLQDVYKIGGIG-TVPVGRVETGIIRPGM
Pp3c1_23750V1.1 (211) YKGPTLLEALDNVS-EPKRPS--------DKPLRLPLQDVYKIGGIG-TVPVGRVETGIIRPGM
Pp3c2_10310V1.1 (211) YKGPTLLEALDNVS-EPKRPS--------DKPLRLPLQDVYKIGGIG-TVPVGRVETGIIRPGM
Pp3c1_23850V1.1 (211) YKGPTLLEALDNVS-EPKRPS--------DKPLRLPLQDVYKIGGIG-TVPVGRVETGIIRPGM
Pp3c1_23900V1.1 (211) YKGPTLLEALDNVS-EPKRPS--------DKPLRLPLQDVYKIGGIG-TVPVGRVETGIIRPGM
Pp3c1_23920V1.1 (211) YKGPTLLEALDNVS-EPKRPS--------DKPLRLPLQDVYKIGGIG-TVPVGRVETGIIRPGM
Consensus (449) YKGPTLLEALDNVS EPKRPS DKPLRLPLQDVYKIGGIG TVPVGRVETGIIRPGM
translation elongation factor EF1A, eucaryotic / archaeal
translation protein beta-barrel

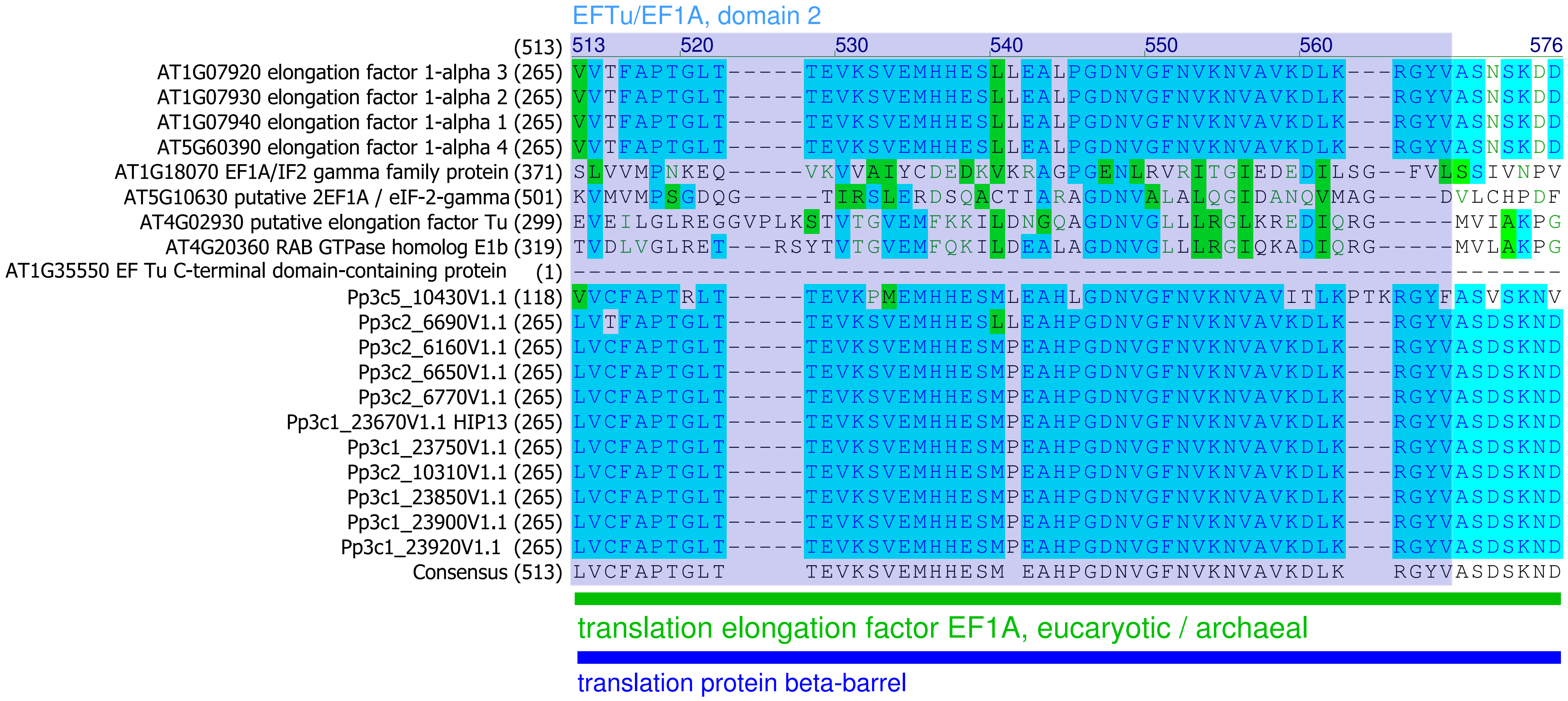
EFTu/EF1A, domain 2
(513) 513 520 530 540 550 560 576
AT1G07920 elongation factor 1-alpha 3 (265) VVTFAPTGLT-----TEVKSVEMHHESLLEALPGDNVGFNVKNVAVKDLK---RGYVASNSKDD
AT1G07930 elongation factor 1-alpha 2 (265) VVTFAPTGLT-----TEVKSVEMHHESLLEALPGDNVGFNVKNVAVKDLK---RGYVASNSKDD
AT1G07940 elongation factor 1-alpha 1 (265) VVTFAPTGLT-----TEVKSVEMHHESLLEALPGDNVGFNVKNVAVKDLK---RGYVASNSKDD
AT5G60390 elongation factor 1-alpha 4 (265) VVTFAPTGLT-----TEVKSVEMHHESLLEALPGDNVGFNVKNVAVKDLK---RGYVASNSKDD
AT1G18070 EF1A/IF2 gamma family protein (371) SLVVMPNKEQ-----VKVVAIYCDEDKVKRAGPGENLRVRITGIEDEDILSG--FVLSSIVNPV
AT5G10630 putative 2EF1A / eIF-2-gamma (501) KVMVMPSGDQG----TIRSLERDSQACTIARAGDNVALALQGIDANQVMAG---DVLCHPDF
AT4G02930 putative elongation factor Tu (299) EVEILGLREGGVPLKSTVTGVEMFKKILDNGQAGDNVGLLLRGLKREDIQRG-----MVIAKPG
AT4G20360 RAB GTPase homolog E1b (319) TVDLVGLRET---RSYTVTGVEMFQKILDEALAGDNVGLLLRGIQKADIQRG-----MVLAKPG
AT1G35550 EF Tu C-terminal domain-containing protein (1) --
Pp3c5_10430V1.1 (118) VVCFAPTRLT-----TEVKPMEMHHESMLEAHLGDNVGFNVKNVAVITLKPTKRGYFASVSKNV
Pp3c2_6690V1.1 (265) LVTFAPTGLT-----TEVKSVEMHHESLLEAHPGDNVGFNVKNVAVKDLK---RGYVASDSKND
Pp3c2_6160V1.1 (265) LVCFAPTGLT-----TEVKSVEMHHESMPEAHPGDNVGFNVKNVAVKDLK---RGYVASDSKND
Pp3c2_6650V1.1 (265) LVCFAPTGLT-----TEVKSVEMHHESMPEAHPGDNVGFNVKNVAVKDLK---RGYVASDSKND
Pp3c2_6770V1.1 (265) LVCFAPTGLT-----TEVKSVEMHHESMPEAHPGDNVGFNVKNVAVKDLK---RGYVASDSKND
Pp3c1_23670V1.1 HIP13 (265) LVCFAPTGLT-----TEVKSVEMHHESMPEAHPGDNVGFNVKNVAVKDLK---RGYVASDSKND
Pp3c1_23750V1.1 (265) LVCFAPTGLT-----TEVKSVEMHHESMPEAHPGDNVGFNVKNVAVKDLK---RGYVASDSKND
Pp3c2_10310V1.1 (265) LVCFAPTGLT-----TEVKSVEMHHESMPEAHPGDNVGFNVKNVAVKDLK---RGYVASDSKND
Pp3c1_23850V1.1 (265) LVCFAPTGLT-----TEVKSVEMHHESMPEAHPGDNVGFNVKNVAVKDLK---RGYVASDSKND
Pp3c1_23900V1.1 (265) LVCFAPTGLT-----TEVKSVEMHHESMPEAHPGDNVGFNVKNVAVKDLK---RGYVASDSKND
Pp3c1_23920V1.1 (265) LVCFAPTGLT-----TEVKSVEMHHESMPEAHPGDNVGFNVKNVAVKDLK---RGYVASDSKND
Consensus (513) LVCFAPTGLT TEVKSVEMHHESM EAHPGDNVGFNVKNVAVKDLK RGYVASDSKND
translation elongation factor EF1A, eucaryotic / archaeal
translation protein beta-barrel

EFTu/EF1A, C-terminal

```
                                                         (577) 577         590       600       610       620       630       640
AT1G07920 elongation factor 1-alpha 3                    (321) PAKGAANFTSQVIIMNHPGQIGNGYAPVLDCHTSHIAVKFSEILTKIDRRSGKEIEKEPKFLKN
AT1G07930 elongation factor 1-alpha 2                    (321) PAKGAANFTSQVIIMNHPGQIGNGYAPVLDCHTSHIAVKFSEILTKIDRRSGKEIEKEPKFLKN
AT1G07940 elongation factor 1-alpha 1                    (321) PAKGAANFTSQVIIMNHPGQIGNGYAPVLDCHTSHIAVKFSEILTKIDRRSGKEIEKEPKFLKN
AT5G60390 elongation factor 1-alpha 4                    (321) PAKGAANFTSQVIIMNHPGQIGNGYAPVLDCHTSHIAVKFSEILTKIDRRSGKEIEKEPKFLKN
AT1G18070 EF1A/IF2 gamma family protein                  (428) PAVTEFVAQLQILELLDNAIFTAGYKAILHIHAVVEECEIIELKSQIDLKTRKPMKKKVLFVKN
AT5G10630 putative 2EF1A / eIF-2-gamma                   (556) PVSVATHLELMVLVLEGATPILLGSQLEFHVHHAKEAATVVKLVAMLDPKTGQPTKKSPRCLTA
AT4G02930 putative elongation factor Tu                 (358) SCKTYKKFEAEIYVLTK--DEGGRHTAFFSNYRPQFYLRTADITGKVELPEN------VKMVMP
AT4G20360 RAB GTPase homolog E1b                         (375) SITPHTKFEAIIYVLKK--EEGGRHSPFFAGYRPQFYMRTTDVTGKVTKIMNDKDE-ESKMVMP
AT1G35550 EF Tu C-terminal domain-containing protein       (1) --------------MNHLGQIKNGYTPVLDCHTSHIAVKFSEILTKIDWRTGHEIEKEPKFLKN
Pp3c5_10430V1.1                                        (177) SARGTANFTSQVMIMNHLVQIDNGYAPVHDCHTSHIAAKSAEILIKVDRRSDKELEKEPSF---
Pp3c2_6690V1.1                                         (321) PAKEAANFTAQVIIMNHPGQIGNGYAPVLDCHTSHIAVKFAEILTKVDRRSGKELEKEPKFLKN
Pp3c2_6160V1.1                                         (321) PAKEAANFTAQVIIMNHPGQIGNGYAPVLDCHTSHIAVKFAEILTKVDRRSGKELEKEPKFLKN
Pp3c2_6650V1.1                                         (321) PAKEAANFTAQVIIMNHPGQIGNGYAPVLDCHTSHIAVKFAEILTKVDRRSGKELEKEPKFLKN
Pp3c2_6770V1.1                                         (321) PAKEAANFTAQVIIMNHPGQIGNGYAPVLDCHTSHIAVKFAEILTKVDRRSGKELEKEPKFLKN
Pp3c1_23670V1.1 HIP13                                  (321) PAKEAANFTAQVIIMNHPGQIGNGYAPVLDCHTSHIAVKFAEILTKVDRRSGKELEKEPKFLKN
Pp3c1_23750V1.1                                        (321) PAKEAANFTAQVIIMNHPGQIGNGYAPVLDCHTSHIAVKFAEILTKVDRRSGKELEKEPKFLKN
Pp3c2_10310V1.1                                        (321) PAKEAANFTAQVIIMNHPGQIGNGYAPVLDCHTSHIAVKFAEILTKVDRRSGKELEKEPKFLKN
Pp3c1_23850V1.1                                        (321) PAKEAANFTAQVIIMNHPGQIGNGYAPVLDCHTSHIAVKFAEILTKVDRRSGKELEKEPKFLKN
Pp3c1_23900V1.1                                        (321) PAKEAANFTAQVIIMNHPGQIGNGYAPVLDCHTSHIAVKFAEILTKVDRRSGKELEKEPKFLKN
Pp3c1_23920V1.1                                        (321) PAKEAANFTAQVIIMNHPGQIGNGYAPVLDCHTSHIAVKFAEILTKVDRRSGKELEKEPKFLKN
Consensus                                              (577) PAKEAANFTAQVIIMNHPGQIGNGYAPVLDCHTSHIAVKFAEILTKVDRRSGKELEKEPKFLKN
```

translation elongation factor EF1A, eucaryotic / archaeal

translation protein beta-barrel

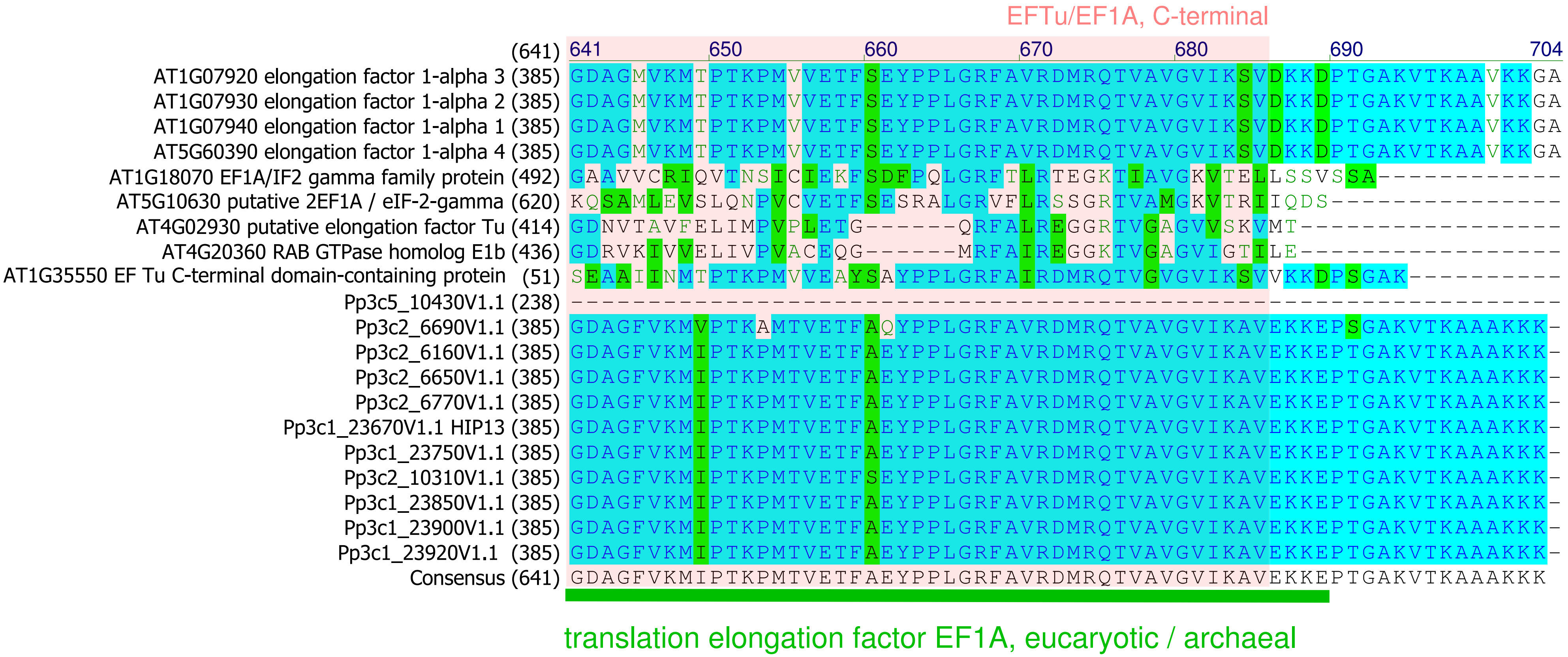

EFTu/EF1A, C-terminal
(641) 641 650 660 670 680 690 704
AT1G07920 elongation factor 1-alpha 3 (385) GDAGMVKMTPTKPMVVETFSEYPPLGRFAVRDMRQTVAVGVIKSVDKKDPTGAKVTKAAVKKGA
AT1G07930 elongation factor 1-alpha 2 (385) GDAGMVKMTPTKPMVVETFSEYPPLGRFAVRDMRQTVAVGVIKSVDKKDPTGAKVTKAAVKKGA
AT1G07940 elongation factor 1-alpha 1 (385) GDAGMVKMTPTKPMVVETFSEYPPLGRFAVRDMRQTVAVGVIKSVDKKDPTGAKVTKAAVKKGA
AT5G60390 elongation factor 1-alpha 4 (385) GDAGMVKMTPTKPMVVETFSEYPPLGRFAVRDMRQTVAVGVIKSVDKKDPTGAKVTKAAVKKGA
AT1G18070 EF1A/IF2 gamma family protein (492) GAAVVCRIQVTNSICIEKFSDFPQLGRFTLRTEGKTIAVGKVTELLSSVSSA------------
AT5G10630 putative 2EF1A / eIF-2-gamma (620) KQSAMLEVSLQNPVCVETFSESRALGRVFLRSSGRTVAMGKVTRIIQDS---------------
AT4G02930 putative elongation factor Tu (414) GDNVTAVFELIMPVPLETG------QRFALREGGRTVGAGVVSKVMT-----------------
AT4G20360 RAB GTPase homolog E1b (436) GDRVKIVVELIVPVACEQG------MRFAIREGGKTVGAGVIGTILE-----------------
AT1G35550 EF Tu C-terminal domain-containing protein (51) SEAAIINMTPTKPMVVEAYSAYPPLGRFAIRDMRQTVGVGVIKSVVKKDPSGAK----------
Pp3c5_10430V1.1 (238) --
Pp3c2_6690V1.1 (385) GDAGFVKMVPTKAMTVETFAQYPPLGRFAVRDMRQTVAVGVIKAVEKKEPSGAKVTKAAAKKK-
Pp3c2_6160V1.1 (385) GDAGFVKMIPTKPMTVETFAEYPPLGRFAVRDMRQTVAVGVIKAVEKKEPTGAKVTKAAAKKK-
Pp3c2_6650V1.1 (385) GDAGFVKMIPTKPMTVETFAEYPPLGRFAVRDMRQTVAVGVIKAVEKKEPTGAKVTKAAAKKK-
Pp3c2_6770V1.1 (385) GDAGFVKMIPTKPMTVETFAEYPPLGRFAVRDMRQTVAVGVIKAVEKKEPTGAKVTKAAAKKK-
Pp3c1_23670V1.1 HIP13 (385) GDAGFVKMIPTKPMTVETFAEYPPLGRFAVRDMRQTVAVGVIKAVEKKEPTGAKVTKAAAKKK-
Pp3c1_23750V1.1 (385) GDAGFVKMIPTKPMTVETFAEYPPLGRFAVRDMRQTVAVGVIKAVEKKEPTGAKVTKAAAKKK-
Pp3c2_10310V1.1 (385) GDAGFVKMIPTKPMTVETFSEYPPLGRFAVRDMRQTVAVGVIKAVEKKEPTGAKVTKAAAKKK-
Pp3c1_23850V1.1 (385) GDAGFVKMIPTKPMTVETFAEYPPLGRFAVRDMRQTVAVGVIKAVEKKEPTGAKVTKAAAKKK-
Pp3c1_23900V1.1 (385) GDAGFVKMIPTKPMTVETFAEYPPLGRFAVRDMRQTVAVGVIKAVEKKEPTGAKVTKAAAKKK-
Pp3c1_23920V1.1 (385) GDAGFVKMIPTKPMTVETFAEYPPLGRFAVRDMRQTVAVGVIKAVEKKEPTGAKVTKAAAKKK-
Consensus (641) GDAGFVKMIPTKPMTVETFAEYPPLGRFAVRDMRQTVAVGVIKAVEKKEPTGAKVTKAAAKKK
translation elongation factor EF1A, eucaryotic / archaeal

	(705)	705
AT1G07920 elongation factor 1-alpha 3	(449)	K
AT1G07930 elongation factor 1-alpha 2	(449)	K
AT1G07940 elongation factor 1-alpha 1	(449)	K
AT5G60390 elongation factor 1-alpha 4	(449)	K
AT1G18070 EF1A/IF2 gamma family protein	(544)	-
AT5G10630 putative 2EF1A / eIF-2-gamma	(669)	-
AT4G02930 putative elongation factor Tu	(455)	-
AT4G20360 RAB GTPase homolog E1b	(477)	-
AT1G35550 EF Tu C-terminal domain-containing protein	(105)	-
Pp3c5_10430V1.1	(238)	-
Pp3c2_6690V1.1	(448)	-
Pp3c2_6160V1.1	(448)	-
Pp3c2_6650V1.1	(448)	-
Pp3c2_6770V1.1	(448)	-
Pp3c1_23670V1.1 HIP13	(448)	-
Pp3c1_23750V1.1	(448)	-
Pp3c2_10310V1.1	(448)	-
Pp3c1_23850V1.1	(448)	-
Pp3c1_23900V1.1	(448)	-
Pp3c1_23920V1.1	(448)	-
Consensus	(705)	
