# Supplementary material for: Holophytochrome-Interacting Proteins in Physcomitrella: Putative Actors in Phytochrome Cytoplasmic Signaling
Source: Front Plant Sci. 2016 May 12;7:613. doi: 10.3389/fpls.2016.00613 (PMC4867686; doi:10.3389/fpls.2016.00613)
Supplement: Supplementary file 2 [file Data_Sheet_2.ZIP › SI/SI HIP14.pdf]

Supplementary Material

Holophytochrome-interacting proteins in Physcomitrella: putative actors in phytochrome cytoplasmic signaling

Anna Lena Ermert, Katharina Mailliet, and Jon Hughes*

*** Correspondence:** jon.hughes@uni-giessen.de

HIP14 (Pp3c3_8540C1.1)

```
ATGACGGAGCTACGAGAGGAAAATGTGTACATGGCTAAGCTCGCCGAGCAGGCGGAGCGGTACGATGAGATGGTGGAAGCC
ATGGAGAATGTGGTAAAGGCGGTGGAGAACGAGGAGCTGACCGTGGAGGAGCGGAACCTGTTGTCGGTGGCGTTTAAGAAC
GTGATTGGTGCGAGGAGGGCGTCGTGGCGGATCATCTCTTCCATCGAGCAGAAGGAAGAGGCCAAGGGGTCTGAGGAGCAC
GTCGCTGCTATTAAGGAGTACCGATCCAAAGTAGAGGCTGAGTTGAGCACCATCTGTGACACTATATTGAAGCTTTTGGAC
TCGCACCTGATCCCGTCCTCCACCTCGGGGGAGTCGAAGGTTTTTTACTTGAAAATGAAGGGAGACTATCACAGGTACCTG
GCTGAGTTCAAAGCCGGCGCTGAGAGAAAAGAGGCAGCTGAGGCTACATTGCACGCGTACAAGCATGCACAAGACATTTCA
ACGACAGAGTTGGCGTCCACACATCCTATCAGATTGGGATTGGCTTTGAATTTTTCTGTCTTTTACTACGAAATTTTGGTT
TCTCCAGACCGAGCATGCCATCTTGCCAAGCAGGCTTTTGATGAAGCTATCTCTGAGTTAGATACCTTAGGAGAGGAATCC
TACAAAGACAGTACTCTCATTATGCAGCTGCTCCGGGATAACCTTACCTTATGGACTTCAGATATGCAGGACGACATTGGT
GAAGAAGGAAAGGATTCCAAGGTCGAAGATGCTGATGACCACTAG
```

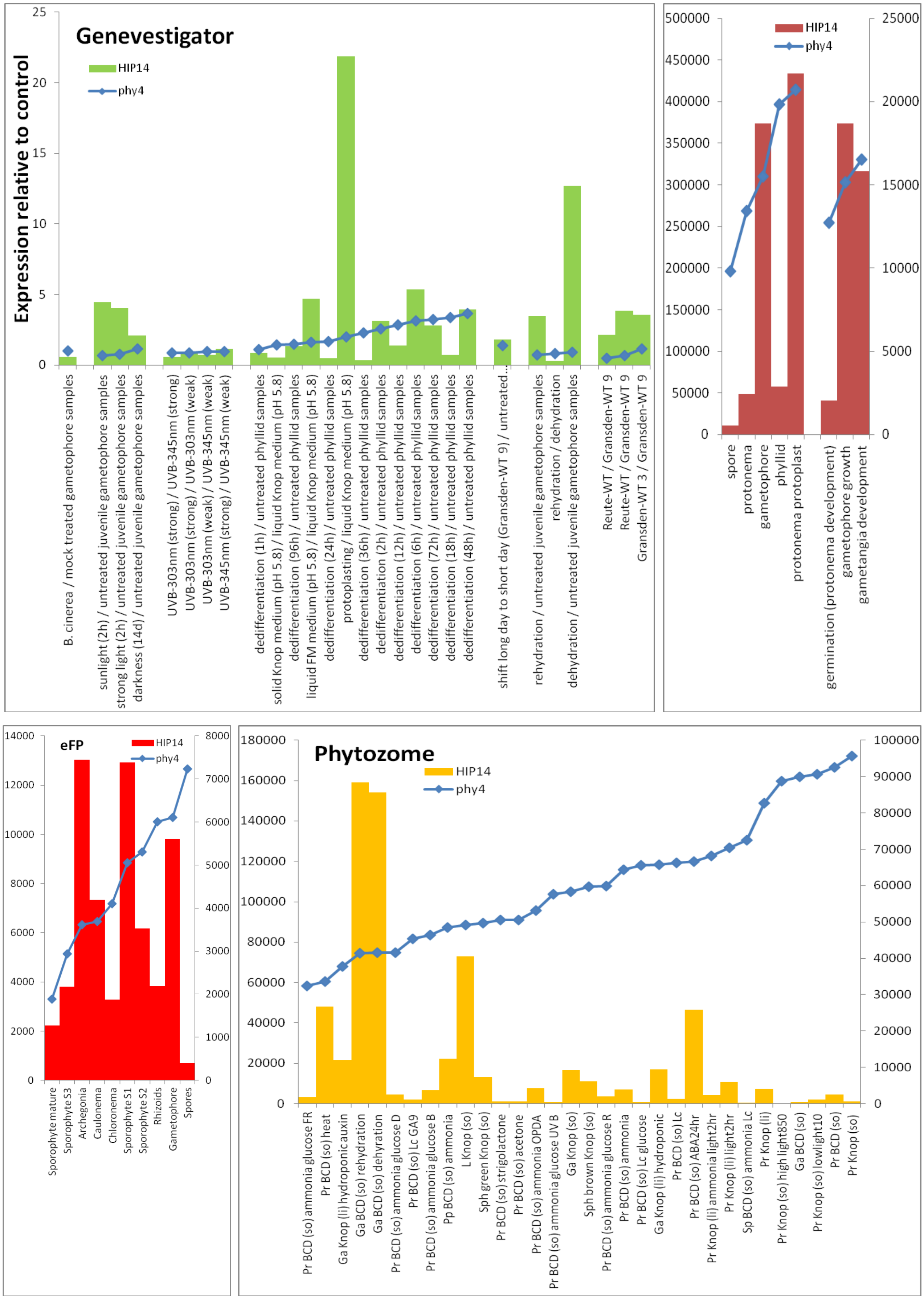

Genevestigator
HIP14
phy4
Expression relative to control
25
20
15
10
5
0
B. cinerea / mock treated gametophore samples
sunlight (2h) / untreated juvenile gametophore samples
strong light (2h) / untreated juvenile gametophore samples
darkness (14d) / untreated juvenile gametophore samples
UVB-303nm (strong) / UVB-345nm (strong)
UVB-303nm (strong) / UVB-303nm (weak)
UVB-303nm (weak) / UVB-345nm (weak)
UVB-345nm (strong) / UVB-345nm (weak)
dedifferentiation (1h) / untreated phyllid samples
solid Knop medium (pH 5.8) / liquid Knop medium (pH 5.8)
dedifferentiation (96h) / untreated phyllid samples
liquid FM medium (pH 5.8) / liquid Knop medium (pH 5.8)
dedifferentiation (24h) / untreated phyllid samples
protoplasting / liquid Knop medium (pH 5.8)
dedifferentiation (36h) / untreated phyllid samples
dedifferentiation (2h) / untreated phyllid samples
dedifferentiation (12h) / untreated phyllid samples
dedifferentiation (6h) / untreated phyllid samples
dedifferentiation (72h) / untreated phyllid samples
dedifferentiation (18h) / untreated phyllid samples
dedifferentiation (48h) / untreated phyllid samples
shift long day to short day (Gransden-WT 9) / untreated...
rehydration / untreated juvenile gametophore samples
rehydration / dehydration
dehydration / untreated juvenile gametophore samples
Reute-WT / Gransden-WT 9
Reute-WT / Gransden-WT 9
Gransden-WT 3 / Gransden-WT 9
HIP14
phy4
500000
450000
400000
350000
300000
250000
200000
150000
100000
50000
0
25000
20000
15000
10000
5000
0
spore
protonema
gametophore
phyllid
protonema protoplast
germination (protonema development)
gametophore growth
gametangia development
eFP
HIP14
phy4
14000
12000
10000
8000
6000
4000
2000
0
8000
7000
6000
5000
4000
3000
2000
1000
0
Sporophyte mature
Sporophyte S3
Archegonia
Caulonema
Chloronema
Sporophyte S1
Sporophyte S2
Rhizoids
Gametophore
Spores
Phytozome
HIP14
phy4
180000
160000
140000
120000
100000
80000
60000
40000
20000
0
100000
90000
80000
70000
60000
50000
40000
30000
20000
10000
0
Pr BCD (so) ammonia glucose FR
Pr BCD (so) heat
Ga Knop (li) hydroponic auxin
Ga BCD (so) rehydration
Ga BCD (so) dehydration
Pr BCD (so) ammonia glucose D
Pr BCD (so) Lc GA9
Pr BCD (so) ammonia glucose B
Pp BCD (so) ammonia
L Knop (so)
Sph green Knop (so)
Pr BCD (so) strigolactone
Pr BCD (so) acetone
Pr BCD (so) ammonia OPDA
Pr BCD (so) ammonia glucose UV B
Ga Knop (so)
Sph brown Knop (so)
Pr BCD (so) ammonia glucose R
Pr BCD (so) ammonia
Pr BCD (so) Lc glucose
Ga Knop (li) hydroponic
Pr BCD (so) Lc
Pr BCD (so) ABA24hr
Pr Knop (li) ammonia light2hr
Pr Knop (li) light2hr
Sp BCD (so) ammonia Lc
Pr Knop (li)
Pr Knop (so) high light850
Ga BCD (so)
Pr Knop (so) lowlight10
Pr BCD (so)
Pr Knop (so)

HIP14 alignment tree

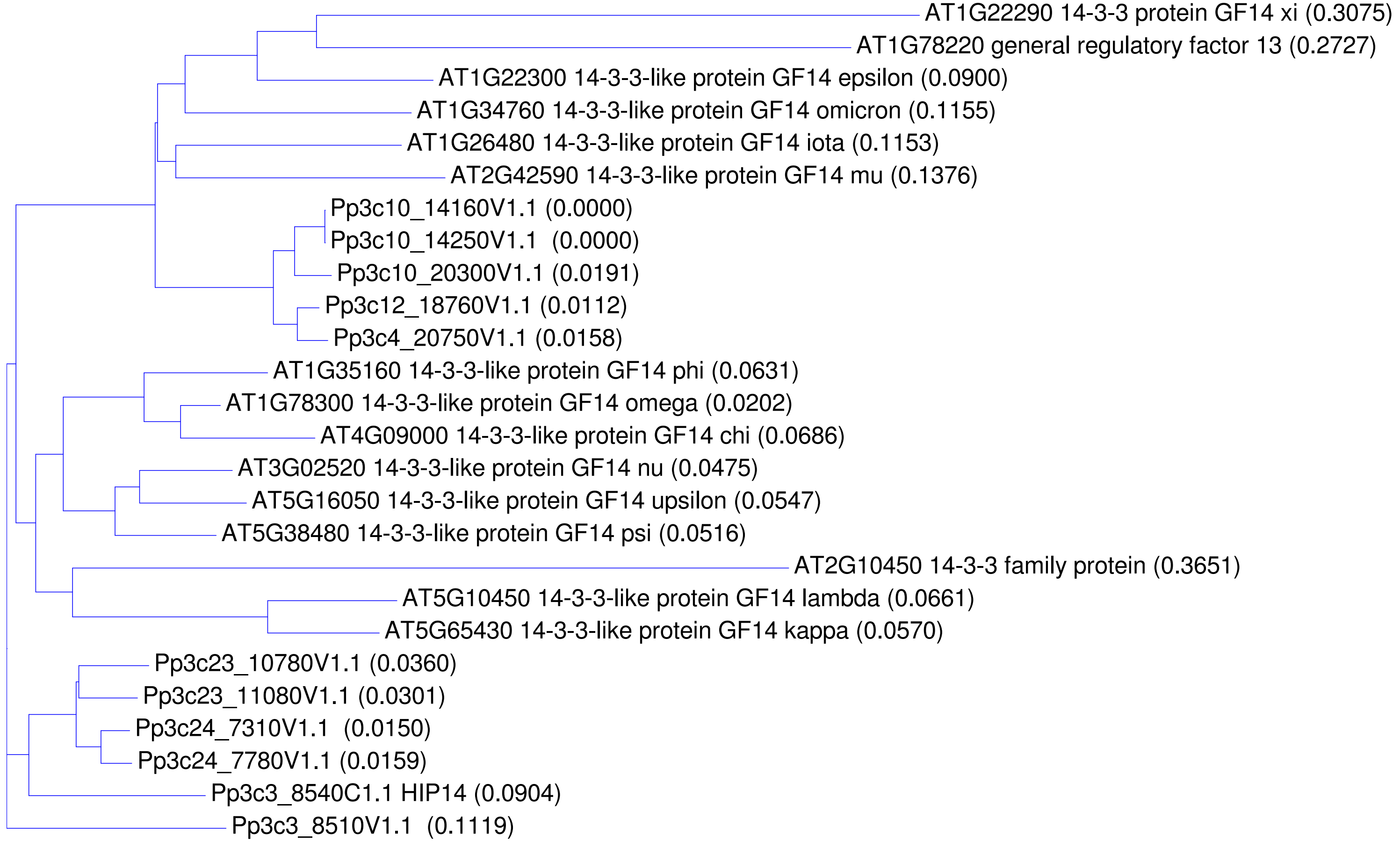

HIP14 alignment

14-3-3 domain

```
                                                   1        10        20        30        40        50                 69
AT1G22290 14-3-3 protein GF14 xi               (1) ------MENEQSTHVHFASLSSSSERYNETFEEIKKAMKKSVQLKAELSAKERNLVSVGYKNVISARRA
AT1G78220 general regulatory factor 13         (1) ------MENEREKLIYLAKLGCQAGRY----DDVMKSMRKVCELDIELSEEERDLLTTGYKNVMEAKRV
AT1G22300 14-3-3-like protein GF14 epsilon    (1) ------MENEREKQVYLAKLSEQTERY----DEMVEAMKKVAQLDVELTVEERNLVSVGYKNVIGARRA
AT1G34760 14-3-3-like protein GF14 omicron    (1) ------MENERAKQVYLAKLNEQAERY----DEMVEAMKKVAALDVELTIEERNLLSVGYKNVIGARRA
AT1G26480 14-3-3-like protein GF14 iota       (1) -MSSSGSDKERETFVYMAKLSEQAERY----DEMVETMKKVARVNSELTVEERNLLSVGYKNVIGARRA
AT2G42590 14-3-3-like protein GF14 mu         (1) ----MGSGKERDTFVYLAKLSEQAERY----EEMVESMKSVAKLNVDLTVEERNLLSVGYKNVIGSRRA
Pp3c10_14160V1.1                               (1) ----MSTEKERESYVYMAKLAEQAERY----DEMVESMKKVAKLDVELTVEERNLLSVGYKNVIGARRA
Pp3c10_14250V1.1                               (1) ----MSTEKERESYVYMAKLAEQAERY----DEMVESMKKVAKLDVELTVEERNLLSVGYKNVIGARRA
Pp3c10_20300V1.1                               (1) ----MSTEKERESQVYMAKLAEQAERY----DEMVESMKKVAKLDVELTVEERNLLSVGYKNVIGARRA
Pp3c12_18760V1.1                               (1) ----MSTEKERESYVYMAKLAEQAERY----DEMVESMKKVAKLDVELTVEERNLLSVGYKNVIGARRA
Pp3c4_20750V1.1                                (1) ----MSAEKERESYVYMAKLAEQAERY----DEMVESMKKVAKLDVELTVEERNLLSVGYKNVIGARRA
AT1G35160 14-3-3-like protein GF14 phi        (1) MAAPPASSSAREEFVYLAKLAEQAERY-EEMVEFMEKVAEAVD-KDELTVEERNLLSVAYKNVIGARRA
AT1G78300 14-3-3-like protein GF14 omega      (1) ------MASGREEFVYMAKLAEQAERY-EEMVEFMEKVSAAVD-GDELTVEERNLLSVAYKNVIGARRA
AT4G09000 14-3-3-like protein GF14 chi        (1) -MATPGASSARDEFVYMAKLAEQAERY-EEMVEFMEKVAKAVD-KDELTVEERNLLSVAYKNVIGARRA
AT3G02520 14-3-3-like protein GF14 nu         (1) ------MSSSREENVYLAKLAEQAERY-EEMVEFMEKVAKTVD-TDELTVEERNLLSVAYKNVIGARRA
AT5G16050 14-3-3-like protein GF14 upsilon    (1) ----MSSDSSREENVYLAKLAEQAERY-EEMVEFMEKVAKTVE-TEELTVEERNLLSVAYKNVIGARRA
AT5G38480 14-3-3-like protein GF14 psi        (1) -------MSTREENVYMAKLAEQAERY-EEMVEFMEKVAKTVD-VEELSVEERNLLSVAYKNVIGARRA
AT2G10450 14-3-3 family protein               (1) ---------------------------------------------------------------------
AT5G10450 14-3-3-like protein GF14 lambda     (1) ----MAATLGRDQYVYMAKLAEQAERY-EEMVQFMEQLVTGATPAEELTVEERNLLSVAYKNVIGSLRA
AT5G65430 14-3-3-like protein GF14 kappa      (1) ----MATTLSRDQYVYMAKLAEQAERY-EEMVQFMEQLVSGATPAGELTVEERNLLSVAYKNVIGSLRA
Pp3c23_10780V1.1                               (1) -----MATEAREENVYMAKLAEQAERY-DEMVEAMEKVAKTVD-TEELTVEERNLLSVAYKNVVGARRA
Pp3c23_11080V1.1                               (1) -----MATEAREENVYMAKLAEQAERY-DEMVEAMEKVAKSVD-TEELTVEERNLLSVAYKNVIGARRA
Pp3c24_7310V1.1                                (1) -----MATEAREENVYMAKLAEQAERY-DEMVEAMEKVAKTVD-TEELTVEERNLLSVAYKNVIGARRA
Pp3c24_7780V1.1                                (1) -----MATEVREENVYMAKLAEQAERY-DEMVEAMEKVAKTVD-TEELTVEERNLLSVAYKNVIGARRA
Pp3c3_8540C1.1 HIP14                           (1) ------MTELREENVYMAKLAEQAERY-DEMVEAMENVVKAVE-NEELTVEERNLLSVAFKNVIGARRA
Pp3c3_8510V1.1                                 (1) -----MATDSRESNVYMAKLAEQAERY-DEMVEAMEKVAMTAD-IAELTIEERNLLSVAYKNVIGARRA
Consensus                                      (1)           RE  VYMAKLAEQAERY EEMVE ME V K  D   ELTVEERNLLSVAYKNVIGARRA
```

14-3-3 protein

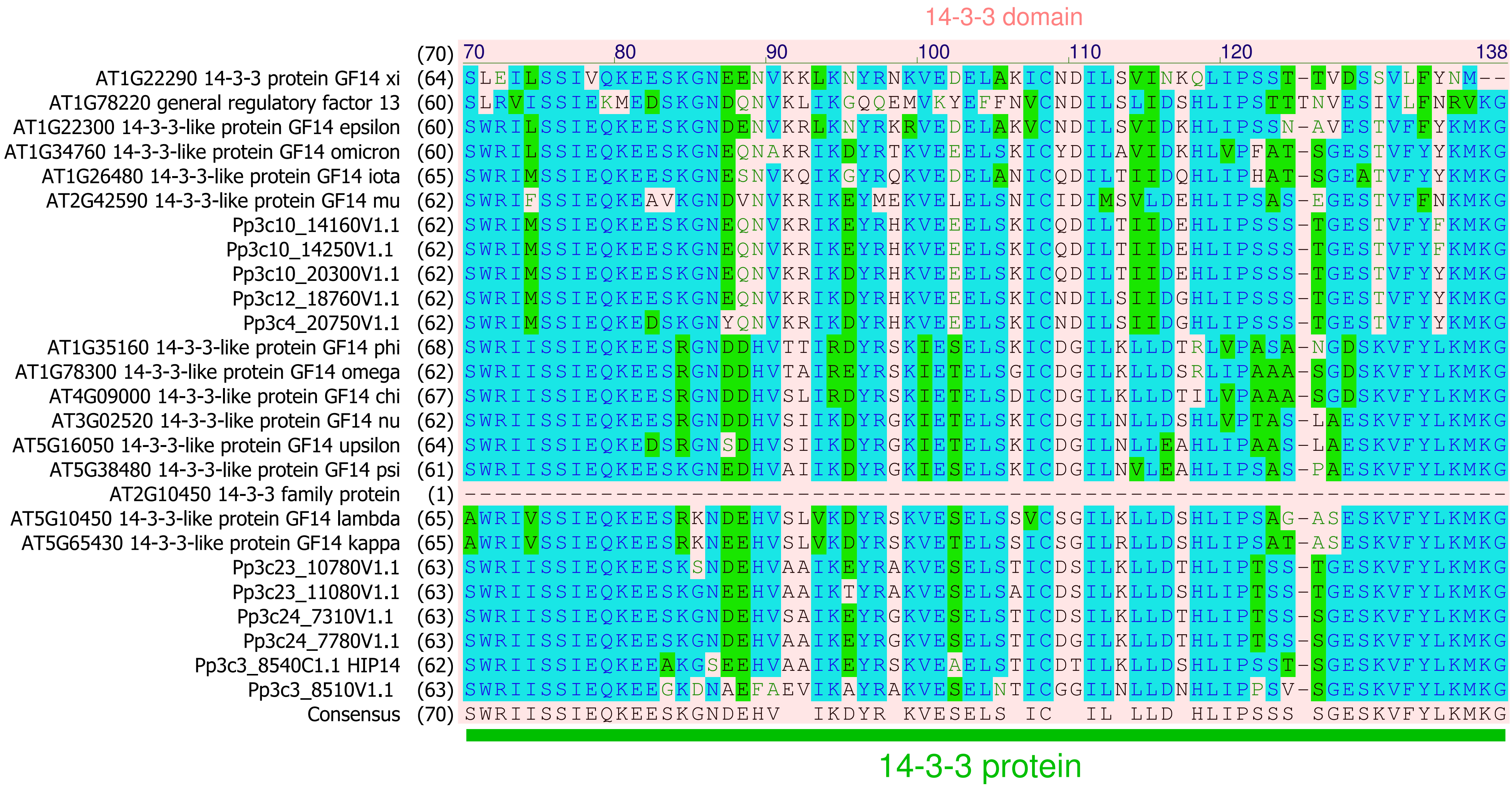
14-3-3 domain
(70) 70 80 90 100 110 120 138
AT1G22290 14-3-3 protein GF14 xi (64) SLEILSSIVQKEESKGNEENVKKLKNYRNKVEDELAKICNDILSVINKQLIPSST-TVDSSVLFYNM--
AT1G78220 general regulatory factor 13 (60) SLRVISSIEKMEDSKGNDQNVKLIKGQQEMVKYEFFNVCNDILSLIDSHLIPSTTTNVESIVLFNRVKG
AT1G22300 14-3-3-like protein GF14 epsilon (60) SWRILSSIEQKEESKGNDENVKRLKNYRKRVEDELAKVCNDILSVIDKHLIPSSN-AVESTVFFYKMKG
AT1G34760 14-3-3-like protein GF14 omicron (60) SWRILSSIEQKEESKGNEQNAKRIKDYRTKVEEELSKICYDILAVIDKHLVPFAT-SGESTVFYYKMKG
AT1G26480 14-3-3-like protein GF14 iota (65) SWRIMSSIEQKEESKGNESNVKQIKGYRQKVEDELANICQDILTIIDQHLIPHAT-SGEATVFYYKMKG
AT2G42590 14-3-3-like protein GF14 mu (62) SWRIFSSIEQKEAVKGNDVNVKRIKEYMEKVELELSNICIDIMSVLDEHLIPSAS-EGESTVFFNKMKG
Pp3c10_14160V1.1 (62) SWRIMSSIEQKEESKGNEQNVKRIKEYRHKVEEELSKICQDILTIIDEHLIPSSS-TGESTVFYFKMKG
Pp3c10_14250V1.1 (62) SWRIMSSIEQKEESKGNEQNVKRIKEYRHKVEEELSKICQDILTIIDEHLIPSSS-TGESTVFYFKMKG
Pp3c10_20300V1.1 (62) SWRIMSSIEQKEESKGNEQNVKRIKDYRHKVEEELSKICQDILTIIDEHLIPSSS-TGESTVFYYKMKG
Pp3c12_18760V1.1 (62) SWRIMSSIEQKEESKGNEQNVKRIKDYRHKVEEELSKICNDILSIIDGHLIPSSS-TGESTVFYYKMKG
Pp3c4_20750V1.1 (62) SWRIMSSIEQKEDSKGNYQNVKRIKDYRHKVEEELSKICNDILSIIDGHLIPSSS-TGESTVFYYKMKG
AT1G35160 14-3-3-like protein GF14 phi (68) SWRIISSIEQKEESRGNDDHVTTIRDYRSKIESELSKICDGILKLLDTRLVPASA-NGDSKVFYLKMKG
AT1G78300 14-3-3-like protein GF14 omega (62) SWRIISSIEQKEESRGNDDHVTAIREYRSKIETELSGICDGILKLLDSRLIPAAA-SGDSKVFYLKMKG
AT4G09000 14-3-3-like protein GF14 chi (67) SWRIISSIEQKEESRGNDDHVSLIRDYRSKIETELSDICDGILKLLDTILVPAAA-SGDSKVFYLKMKG
AT3G02520 14-3-3-like protein GF14 nu (62) SWRIISSIEQKEESRGNDDHVSIIKDYRGKIETELSKICDGILNLLDSHLVPTAS-LAESKVFYLKMKG
AT5G16050 14-3-3-like protein GF14 upsilon (64) SWRIISSIEQKEDSRGNSDHVSIIKDYRGKIETELSKICDGILNLLEAHLIPAAS-LAESKVFYLKMKG
AT5G38480 14-3-3-like protein GF14 psi (61) SWRIISSIEQKEESKGNEDHVAIIKDYRGKIESELSKICDGILNVLEAHLIPSAS-PAESKVFYLKMKG
AT2G10450 14-3-3 family protein (1) ---
AT5G10450 14-3-3-like protein GF14 lambda (65) AWRIVSSIEQKEESRKNDEHVSLVKDYRSKVESELSSVCSGILKLLDSHLIPSAG-ASESKVFYLKMKG
AT5G65430 14-3-3-like protein GF14 kappa (65) AWRIVSSIEQKEESRKNEEHVSLVKDYRSKVETELSSICSGILRLLDSHLIPSAT-ASESKVFYLKMKG
Pp3c23_10780V1.1 (63) SWRIISSIEQKEESKSNDEHVAAIKEYRAKVESELSTICDSILKLLDTHLIPTSS-TGESKVFYLKMKG
Pp3c23_11080V1.1 (63) SWRIISSIEQKEESKGNEEHVAAIKTYRAKVESELSAICDSILKLLDSHLIPTSS-TGESKVFYLKMKG
Pp3c24_7310V1.1 (63) SWRIISSIEQKEESKGNDEHVSAIKEYRGKVESELSTICDSILKLLDTHLIPTSS-SGESKVFYLKMKG
Pp3c24_7780V1.1 (63) SWRIISSIEQKEESKGNDEHVAAIKEYRGKVESELSTICDGILKLLDTHLIPTSS-SGESKVFYLKMKG
Pp3c3_8540C1.1 HIP14 (62) SWRIISSIEQKEEAKGSEEHVAAIKEYRSKVEAELSTICDTILKLLDSHLIPSST-SGESKVFYLKMKG
Pp3c3_8510V1.1 (63) SWRIISSIEQKEEGKDNAEFAEVIKAYRAKVESELNTICGGILNLLDNHLIPPSV-SGESKVFYLKMKG
Consensus (70) SWRIISSIEQKEESKGNDEHV IKDYR KVESELS IC IL LLD HLIPSSS SGESKVFYLKMKG
14-3-3 protein

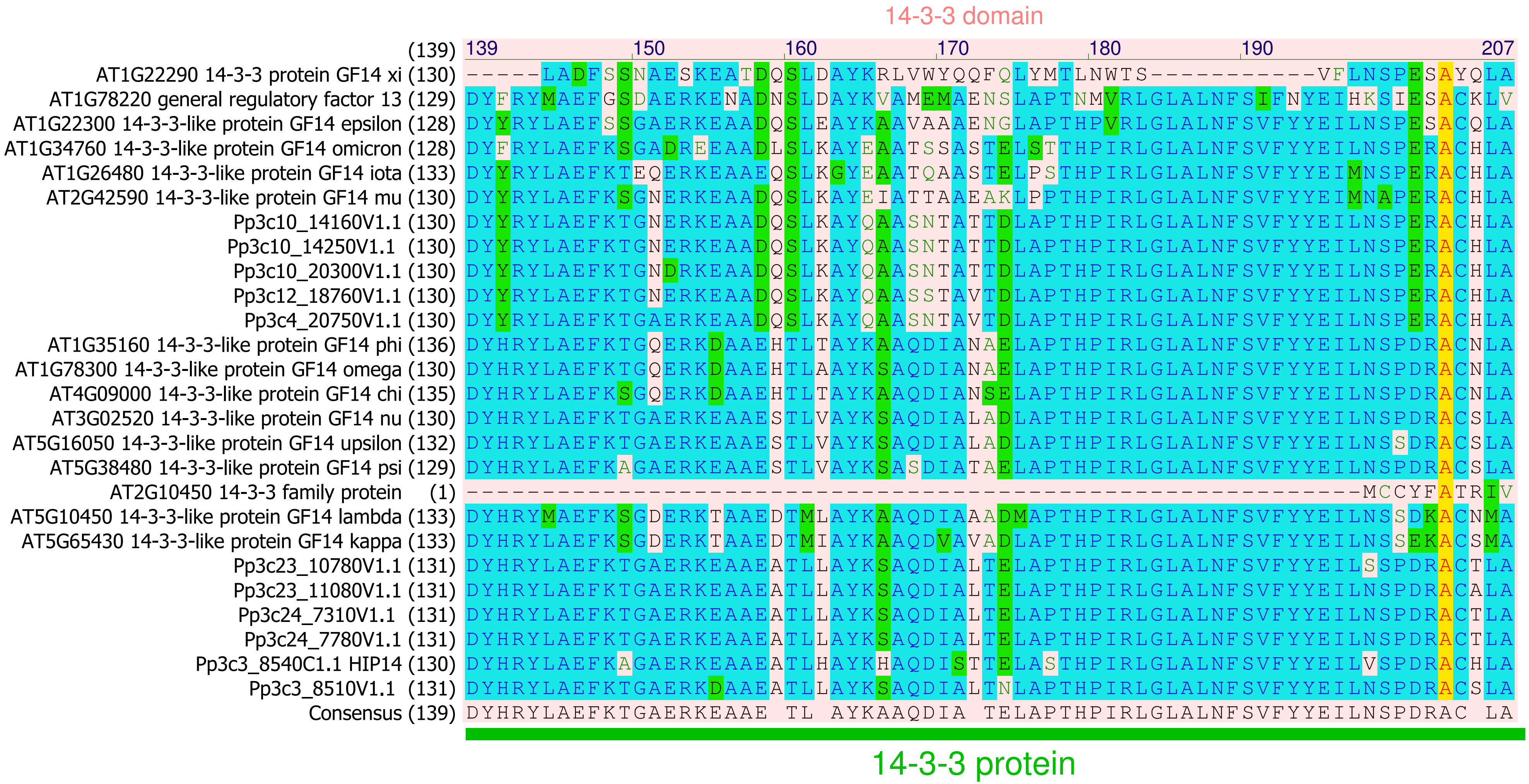

14-3-3 domain
(139) 139 150 160 170 180 190 207
AT1G22290 14-3-3 protein GF14 xi (130) -----LADFSSNAESKEATDQSLDAYKRLVWYQQFQLYMTLNWTS-----------VFLNSPESAYQLA
AT1G78220 general regulatory factor 13 (129) DYFRYMAEFGSDAERKENADNSLDAYKVAMEMAENSLAPTNMVRLGLALNFSIFNYEIHKSIESACKLV
AT1G22300 14-3-3-like protein GF14 epsilon (128) DYYRYLAEFSSGAERKEAADQSLEAYKAAVAAAENGLAPTHPVRLGLALNFSVFYYEILNSPESACQLA
AT1G34760 14-3-3-like protein GF14 omicron (128) DYFRYLAEFKSGADREEAADLSLKAYEAATSSASTELSTTHPIRLGLALNFSVFYYEILNSPERACHLA
AT1G26480 14-3-3-like protein GF14 iota (133) DYYRYLAEFKTEQERKEAAEQSLKGYEAATQAASTELPSTHPIRLGLALNFSVFYYEIMNSPERACHLA
AT2G42590 14-3-3-like protein GF14 mu (130) DYYRYLAEFKSGNERKEAADQSLKAYEIATTAAEAKLPPTHPIRLGLALNFSVFYYEIMNAPERACHLA
Pp3c10_14160V1.1 (130) DYYRYLAEFKTGNERKEAADQSLKAYQAASNTATTDLAPTHPIRLGLALNFSVFYYEILNSPERACHLA
Pp3c10_14250V1.1 (130) DYYRYLAEFKTGNERKEAADQSLKAYQAASNTATTDLAPTHPIRLGLALNFSVFYYEILNSPERACHLA
Pp3c10_20300V1.1 (130) DYYRYLAEFKTGNDRKEAADQSLKAYQAASNTATTDLAPTHPIRLGLALNFSVFYYEILNSPERACHLA
Pp3c12_18760V1.1 (130) DYYRYLAEFKTGNERKEAADQSLKAYQAASSTAVTDLAPTHPIRLGLALNFSVFYYEILNSPERACHLA
Pp3c4_20750V1.1 (130) DYYRYLAEFKTGAERKEAADQSLKAYQAASNTAVTDLAPTHPIRLGLALNFSVFYYEILNSPERACHLA
AT1G35160 14-3-3-like protein GF14 phi (136) DYHRYLAEFKTGQERKDAAEHTLTAYKAAQDIANAELAPTHPIRLGLALNFSVFYYEILNSPDRACNLA
AT1G78300 14-3-3-like protein GF14 omega (130) DYHRYLAEFKTGQERKDAAEHTLAAYKSAQDIANAELAPTHPIRLGLALNFSVFYYEILNSPDRACNLA
AT4G09000 14-3-3-like protein GF14 chi (135) DYHRYLAEFKSGQERKDAAEHTLTAYKAAQDIANSELAPTHPIRLGLALNFSVFYYEILNSPDRACNLA
AT3G02520 14-3-3-like protein GF14 nu (130) DYHRYLAEFKTGAERKEAAESTLVAYKSAQDIALADLAPTHPIRLGLALNFSVFYYEILNSPDRACSLA
AT5G16050 14-3-3-like protein GF14 upsilon (132) DYHRYLAEFKTGAERKEAAESTLVAYKSAQDIALADLAPTHPIRLGLALNFSVFYYEILNSSDRACSLA
AT5G38480 14-3-3-like protein GF14 psi (129) DYHRYLAEFKAGAERKEAAESTLVAYKSASDIATAELAPTHPIRLGLALNFSVFYYEILNSPDRACSLA
AT2G10450 14-3-3 family protein (1) ---MCCYFATRIV
AT5G10450 14-3-3-like protein GF14 lambda (133) DYHRYMAEFKSGDERKTAAEDTMLAYKAAQDIAADMAPTHPIRLGLALNFSVFYYEILNSSDKACNMA
AT5G65430 14-3-3-like protein GF14 kappa (133) DYHRYLAEFKSGDERKTAAEDTMIAYKAAQDVAVADLAPTHPIRLGLALNFSVFYYEILNSSEKACSMA
Pp3c23_10780V1.1 (131) DYHRYLAEFKTGAERKEAAEATLLAYKSAQDIALTELAPTHPIRLGLALNFSVFYYEILSSPDRACTLA
Pp3c23_11080V1.1 (131) DYHRYLAEFKTGAERKEAAEATLLAYKSAQDIALTELAPTHPIRLGLALNFSVFYYEILNSPDRACALA
Pp3c24_7310V1.1 (131) DYHRYLAEFKTGAERKEAAEATLLAYKSAQDIALTELAPTHPIRLGLALNFSVFYYEILNSPDRACTLA
Pp3c24_7780V1.1 (131) DYHRYLAEFKTGAERKEAAEATLLAYKSAQDIALTELAPTHPIRLGLALNFSVFYYEILNSPDRACTLA
Pp3c3_8540C1.1 HIP14 (130) DYHRYLAEFKAGAERKEAAEATLHAYKHAQDISTTELASTHPIRLGLALNFSVFYYEILVSPDRACHLA
Pp3c3_8510V1.1 (131) DYHRYLAEFKTGAERKDAAEATLLAYKSAQDIALTNLAPTHPIRLGLALNFSVFYYEILNSPDRACSLA
Consensus (139) DYHRYLAEFKTGAERKEAAE TL AYKAAQDIA TELAPTHPIRLGLALNFSVFYYEILNSPDRAC LA
14-3-3 protein

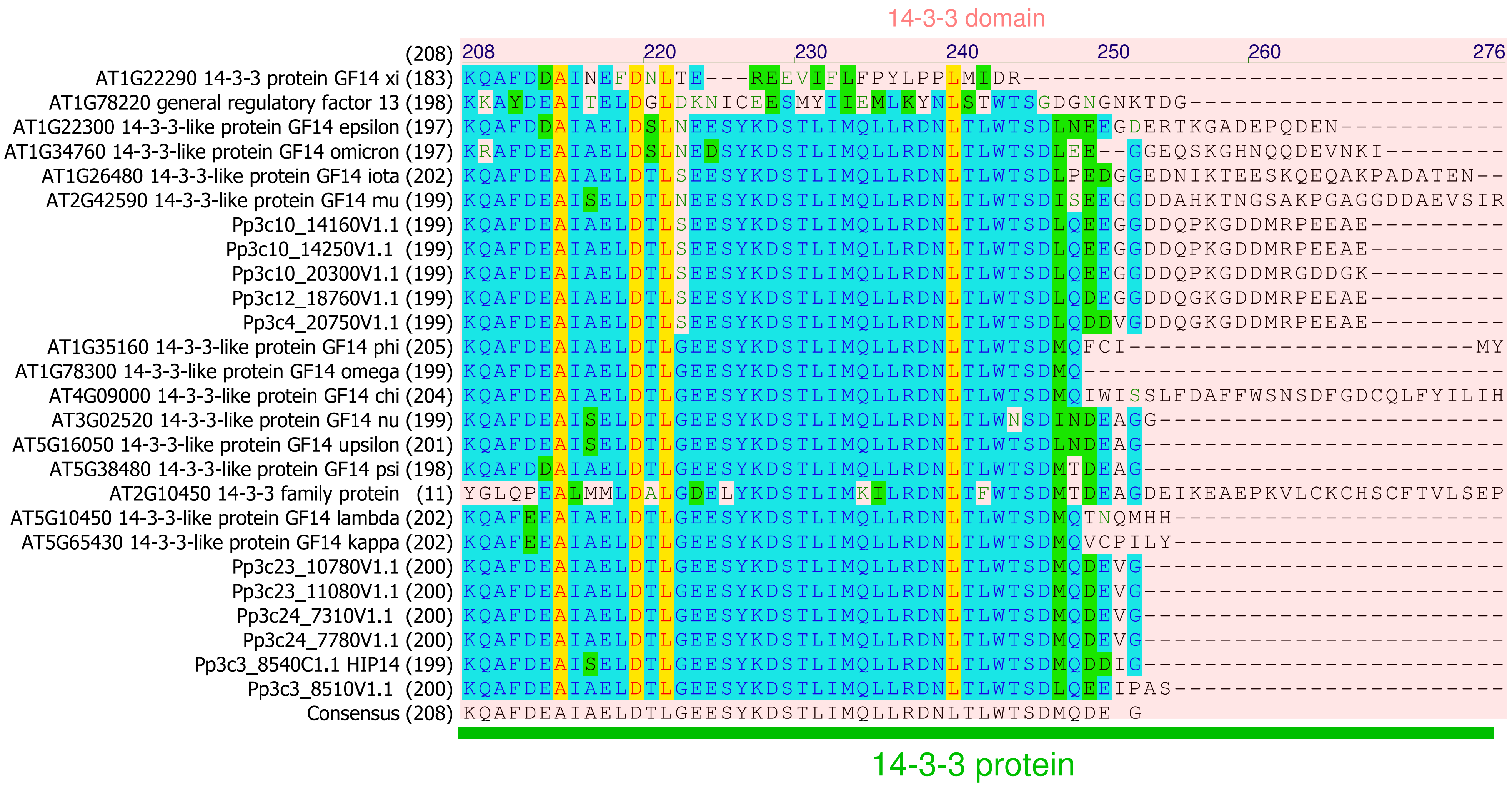

14-3-3 domain
(208) 208 220 230 240 250 260 276
AT1G22290 14-3-3 protein GF14 xi (183) KQAFDDAINEFDNLTE---REEVIFLFPYLPPLMIDR--------------------------------
AT1G78220 general regulatory factor 13 (198) KKAYDEAITELDGLDKNICEESMYIIEMLKYNLSTWTSGDGNGNKTDG---------------------
AT1G22300 14-3-3-like protein GF14 epsilon (197) KQAFDDAIAELDSLNEESYKDSTLIMQLLRDNLTLWTSDLNEEGDERTKGADEPQDEN-----------
AT1G34760 14-3-3-like protein GF14 omicron (197) KRAFDEAIAELDSLNEDSYKDSTLIMQLLRDNLTLWTSDLEE--GGEQSKGHNQQDEVNKI--------
AT1G26480 14-3-3-like protein GF14 iota (202) KQAFDEAIAELDTLSEESYKDSTLIMQLLRDNLTLWTSDLPEDGGEDNIKTEESKQEQAKPADATEN--
AT2G42590 14-3-3-like protein GF14 mu (199) KQAFDEAISELDTLNEESYKDSTLIMQLLRDNLTLWTSDISEEGGDDAHKTNGSAKPGAGGDDAEVSIR
Pp3c10_14160V1.1 (199) KQAFDEAIAELDTLSEESYKDSTLIMQLLRDNLTLWTSDLQEEGGDDQPKGDDMRPEEAE---------
Pp3c10_14250V1.1 (199) KQAFDEAIAELDTLSEESYKDSTLIMQLLRDNLTLWTSDLQEEGGDDQPKGDDMRPEEAE---------
Pp3c10_20300V1.1 (199) KQAFDEAIAELDTLSEESYKDSTLIMQLLRDNLTLWTSDLQEEGGDDQPKGDDMRGDDGK---------
Pp3c12_18760V1.1 (199) KQAFDEAIAELDTLSEESYKDSTLIMQLLRDNLTLWTSDLQDEGGDDQGKGDDMRPEEAE---------
Pp3c4_20750V1.1 (199) KQAFDEAIAELDTLSEESYKDSTLIMQLLRDNLTLWTSDLQDDVGDDQGKGDDMRPEEAE---------
AT1G35160 14-3-3-like protein GF14 phi (205) KQAFDEAIAELDTLGEESYKDSTLIMQLLRDNLTLWTSDMQFCI-----------------------MY
AT1G78300 14-3-3-like protein GF14 omega (199) KQAFDEAIAELDTLGEESYKDSTLIMQLLRDNLTLWTSDMQ----------------------------
AT4G09000 14-3-3-like protein GF14 chi (204) KQAFDEAIAELDTLGEESYKDSTLIMQLLRDNLTLWTSDMQIWISSLFDAFFWSNSDFGDCQLFYILIH
AT3G02520 14-3-3-like protein GF14 nu (199) KQAFDEAISELDTLGEESYKDSTLIMQLLRDNLTLWNSDINDEAGG-----------------------
AT5G16050 14-3-3-like protein GF14 upsilon (201) KQAFDEAISELDTLGEESYKDSTLIMQLLRDNLTLWTSDLNDEAG------------------------
AT5G38480 14-3-3-like protein GF14 psi (198) KQAFDDAIAELDTLGEESYKDSTLIMQLLRDNLTLWTSDMTDEAG------------------------
AT2G10450 14-3-3 family protein (11) YGLQPEALMMLDALGDELYKDSTLIMKILRDNLTFWTSDMTDEAGDEIKEAEPKVLCKCHSCFTVLSEP
AT5G10450 14-3-3-like protein GF14 lambda (202) KQAFEEAIAELDTLGEESYKDSTLIMQLLRDNLTLWTSDMQTNQMHH----------------------
AT5G65430 14-3-3-like protein GF14 kappa (202) KQAFEEAIAELDTLGEESYKDSTLIMQLLRDNLTLWTSDMQVCPILY----------------------
Pp3c23_10780V1.1 (200) KQAFDEAIAELDTLGEESYKDSTLIMQLLRDNLTLWTSDMQDEVG------------------------
Pp3c23_11080V1.1 (200) KQAFDEAIAELDTLGEESYKDSTLIMQLLRDNLTLWTSDMQDEVG------------------------
Pp3c24_7310V1.1 (200) KQAFDEAIAELDTLGEESYKDSTLIMQLLRDNLTLWTSDMQDEVG------------------------
Pp3c24_7780V1.1 (200) KQAFDEAIAELDTLGEESYKDSTLIMQLLRDNLTLWTSDMQDEVG------------------------
Pp3c3_8540C1.1 HIP14 (199) KQAFDEAISELDTLGEESYKDSTLIMQLLRDNLTLWTSDMQDDIG------------------------
Pp3c3_8510V1.1 (200) KQAFDEAIAELDTLGEESYKDSTLIMQLLRDNLTLWTSDLQEEIPAS----------------------
Consensus (208) KQAFDEAIAELDTLGEESYKDSTLIMQLLRDNLTLWTSDMQDE G
14-3-3 protein

14-3-3 domain

```
                                               (277) 277          290       300       310              327
AT1G22290 14-3-3 protein GF14 xi               (217) ---------------------------------------------------
AT1G78220 general regulatory factor 13         (246) ---------------------------------------------------
AT1G22300 14-3-3-like protein GF14 epsilon     (255) ---------------------------------------------------
AT1G34760 14-3-3-like protein GF14 omicron     (256) ---------------------------------------------------
AT1G26480 14-3-3-like protein GF14 iota        (269) ---------------------------------------------------
AT2G42590 14-3-3-like protein GF14 mu          (268) DSFTRGKSN------------------------------------------
Pp3c10_14160V1.1                               (259) ---------------------------------------------------
Pp3c10_14250V1.1                               (259) ---------------------------------------------------
Pp3c10_20300V1.1                               (259) ---------------------------------------------------
Pp3c12_18760V1.1                               (259) ---------------------------------------------------
Pp3c4_20750V1.1                                (259) ---------------------------------------------------
AT1G35160 14-3-3-like protein GF14 phi          (251) DNTLMSFLVLISDSVVLISTINQDESPEEIKEAA--APKPAEEQKEI----
AT1G78300 14-3-3-like protein GF14 omega        (240) -----------------------DDAADEIKEAA--APKPTEEQQ------
AT4G09000 14-3-3-like protein GF14 chi          (273) SSILLSCGLILIMMILSNVFFNQDDVADDIKEAAPAAAKPADEQQS-----
AT3G02520 14-3-3-like protein GF14 nu           (245) ---------------------------DEIKEASKHEPEEGKPAETGQ---
AT5G16050 14-3-3-like protein GF14 upsilon      (246) ---------------------------DDIKEAPKEVQKVDEQAQPPPSQ-
AT5G38480 14-3-3-like protein GF14 psi          (243) ---------------------------DEIKEASKPDGAE-----------
AT2G10450 14-3-3 family protein                 (80) KHS------------------------------------------------
AT5G10450 14-3-3-like protein GF14 lambda       (249) --------------------------IRDIKEHVKTEITAKPCVLSYYYSM
AT5G65430 14-3-3-like protein GF14 kappa        (249) --------------------------FHIQLHHQKLAY-------------
Pp3c23_10780V1.1                               (245) ---------------------------ADDKEKVVEAED------------
Pp3c23_11080V1.1                               (245) ---------------------------ADDKDAKIEEAEH-----------
Pp3c24_7310V1.1                                (245) ---------------------------PEVKDAKVDDAEH-----------
Pp3c24_7780V1.1                                (245) ---------------------------AEDKDTKVDDVEN-----------
Pp3c3_8540C1.1 HIP14                           (244) ---------------------------EEGKDSKVEDADDH----------
Pp3c3_8510V1.1                                 (247) --------------------------SEAPQAEVKVEAE------------
Consensus                                      (277)
```

14-3-3 protein
