# Supplementary material for: Holophytochrome-Interacting Proteins in Physcomitrella: Putative Actors in Phytochrome Cytoplasmic Signaling
Source: Front Plant Sci. 2016 May 12;7:613. doi: 10.3389/fpls.2016.00613 (PMC4867686; doi:10.3389/fpls.2016.00613)
Supplement: Supplementary file 2 [file Data_Sheet_2.ZIP › SI/SI HIP3.pdf]

Supplementary Material

Holophytochrome-interacting proteins in Physcomitrella: putative actors in phytochrome cytoplasmic signaling

Anna Lena Ermert, Katharina Mailliet, and Jon Hughes*

*** Correspondence:** jon.hughes@uni-giessen.de

HIP3 (Pp3c10_4820V1.1)

```
ATGGCCGTCCTCGCAGTCCGAATGGAGGCTCTCCTCTCTCCATTGATGAGTGGGGAGCAGTTTCGCTATGCTTACAACAAT
GTCGCAGCGCATTCTCACCATCATGTCGTTGCGGGACATACTAGCTGTTTAGACTCCGATGTGGAGGATGACGATGATGAT
GATTTTACGGAAGACCTTGTCCAAGCTTGGAAGTTGTCTATTGGAGCCAATCGCTACGGGTGTACACACTACAAAAGGAGA
TGCAAGATCCGTGCTCCGTGCTGCAATGAAGTCTTCGACTGCCGGCATTGTCATAATGAAGCCAAGAGTGTTAATGAAACA
GACGACAAAAAACGCCACGAGATTGACCGGCACCTCGTTGAAAAGGTCATCTGTTCATTGTGCGACCATGAGCAAAATGTA
CAACAAGTATGTGAAAAGTGCGGGGTCTGTATGGGCGAATTCTTCTGCTCGAAGTGCAACTTCTTTGACGATGATACCTCG
AAGGATCAATATCATTGCGACAAATGTGGGATCTGCAGAACTGGTGGACGTGACAACTTTTTTCATTGTGATCGTTGTGGC
TGTTGCTACTCAGTTAAATTACGCGAGGGTCACACCTGTGTTGAGAAGTCTATGCACCAAGACTGTCCAGTTTGCATGGAG
TACATGTTCGACTCACTGAAGGACATCACAGTTTTAACGTGTGGTCATACTTTGCACCTGGAATGTCTACAAGAGATGCAT
AGTCATTATAAGTACAATTGTCCACTTTGCAACAAGTCCGTTTGCGATATGTCATCTGTCTGGAAGGAGATTGACGAAGAG
ATTGCTGCAACTCAAATGCCAGCAAATGAAATGAGAATGGTTTGGGTCTTCTGTAATGATTGTGGTGCCACAAATGAAGTG
CAATATCATCATGTTGGACAGAAGTGTGGAACTTGTCCATCTTACAACACTCGTCCCACAGATGCTCCAGCCTCACTTGCC
TCATCTAGATCGTAA
```

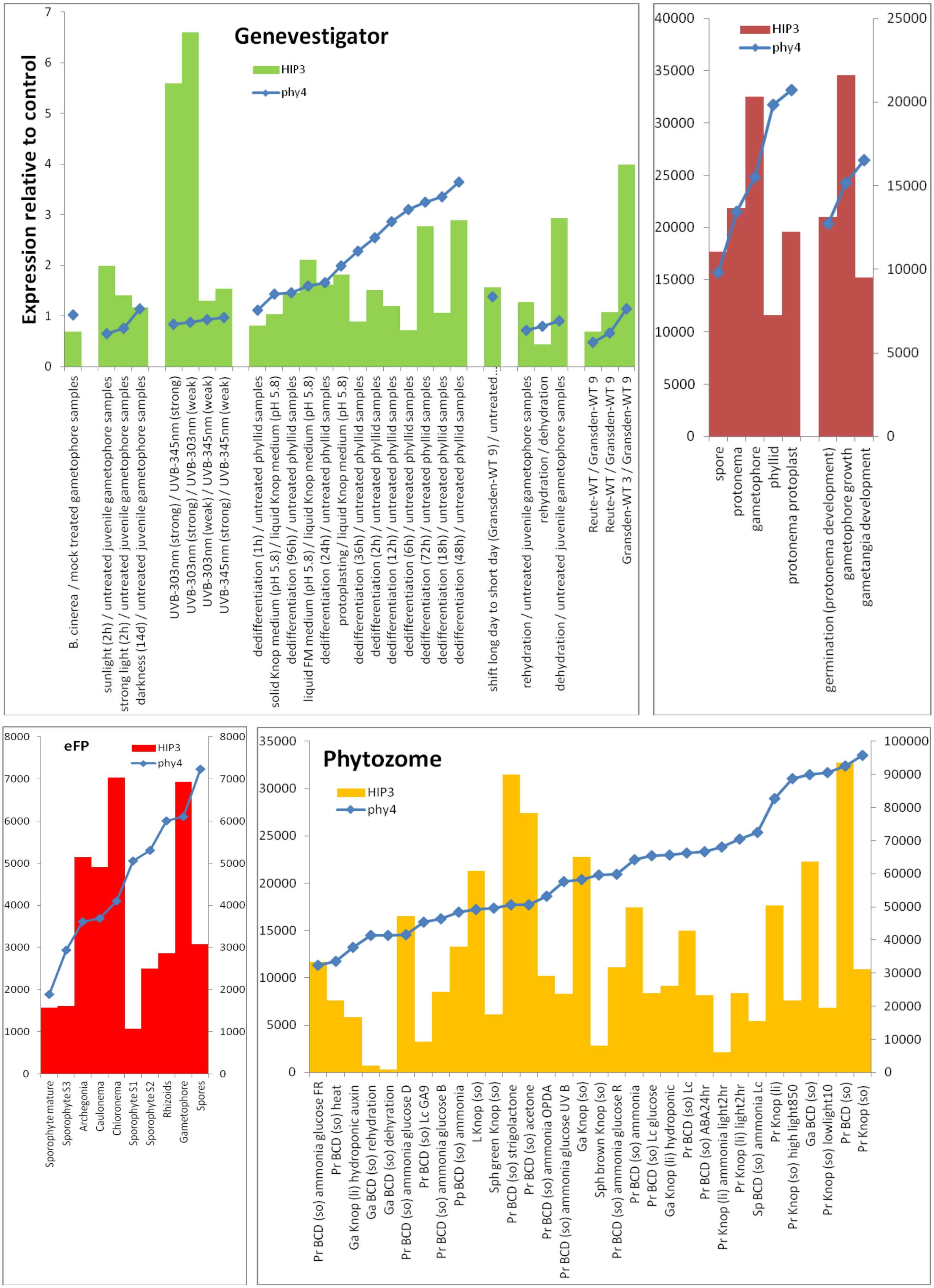
Genevestigator
HIP3
phy4
Expression relative to control
0
1
2
3
4
5
6
7
B. cinerea / mock treated gametophore samples
sunlight (2h) / untreated juvenile gametophore samples
strong light (2h) / untreated juvenile gametophore samples
darkness (14d) / untreated juvenile gametophore samples
UVB-303nm (strong) / UVB-345nm (strong)
UVB-303nm (strong) / UVB-303nm (weak)
UVB-303nm (weak) / UVB-345nm (weak)
UVB-345nm (strong) / UVB-345nm (weak)
dedifferentiation (1h) / untreated phyllid samples
solid Knop medium (pH 5.8) / liquid Knop medium (pH 5.8)
dedifferentiation (96h) / untreated phyllid samples
liquid FM medium (pH 5.8) / liquid Knop medium (pH 5.8)
dedifferentiation (24h) / untreated phyllid samples
protoplasting / liquid Knop medium (pH 5.8)
dedifferentiation (36h) / untreated phyllid samples
dedifferentiation (2h) / untreated phyllid samples
dedifferentiation (12h) / untreated phyllid samples
dedifferentiation (6h) / untreated phyllid samples
dedifferentiation (72h) / untreated phyllid samples
dedifferentiation (18h) / untreated phyllid samples
dedifferentiation (48h) / untreated phyllid samples
shift long day to short day (Gransden-WT 9) / untreated…
rehydration / untreated juvenile gametophore samples
rehydration / dehydration
dehydration / untreated juvenile gametophore samples
Reute-WT / Gransden-WT 9
Reute-WT / Gransden-WT 9
Gransden-WT 3 / Gransden-WT 9
HIP3
phy4
0
5000
10000
15000
20000
25000
30000
35000
40000
0
5000
10000
15000
20000
25000
spore
protonema
gametophore
phyllid
protonema protoplast
germination (protonema development)
gametophore growth
gametangia development
eFP
HIP3
phy4
0
1000
2000
3000
4000
5000
6000
7000
8000
Sporophyte mature
Sporophyte S3
Archegonia
Caulonema
Chloronema
Sporophyte S1
Sporophyte S2
Rhizoids
Gametophore
Spores
Phytozome
HIP3
phy4
0
5000
10000
15000
20000
25000
30000
35000
0
10000
20000
30000
40000
50000
60000
70000
80000
90000
100000
Pr BCD (so) ammonia glucose FR
Pr BCD (so) heat
Ga Knop (li) hydroponic auxin
Ga BCD (so) rehydration
Ga BCD (so) dehyration
Pr BCD (so) ammonia glucose D
Pr BCD (so) Lc GA9
Pr BCD (so) ammonia glucose B
Pp BCD (so) ammonia
L Knop (so)
Sph green Knop (so)
Pr BCD (so) strigolactone
Pr BCD (so) acetone
Pr BCD (so) ammonia OPDA
Pr BCD (so) ammonia glucose UV B
Ga Knop (so)
Sph brown Knop (so)
Pr BCD (so) ammonia glucose R
Pr BCD (so) ammonia
Pr BCD (so) Lc glucose
Ga Knop (li) hydroponic
Pr BCD (so) Lc
Pr BCD (so) ABA24hr
Pr Knop (li) ammonia light2hr
Pr Knop (li) light2hr
Sp BCD (so) ammonia Lc
Pr Knop (li)
Pr Knop (so) high light850
Ga BCD (so)
Pr Knop (so) lowlight10
Pr BCD (so)
Pr Knop (so)

HIP3 alignment tree

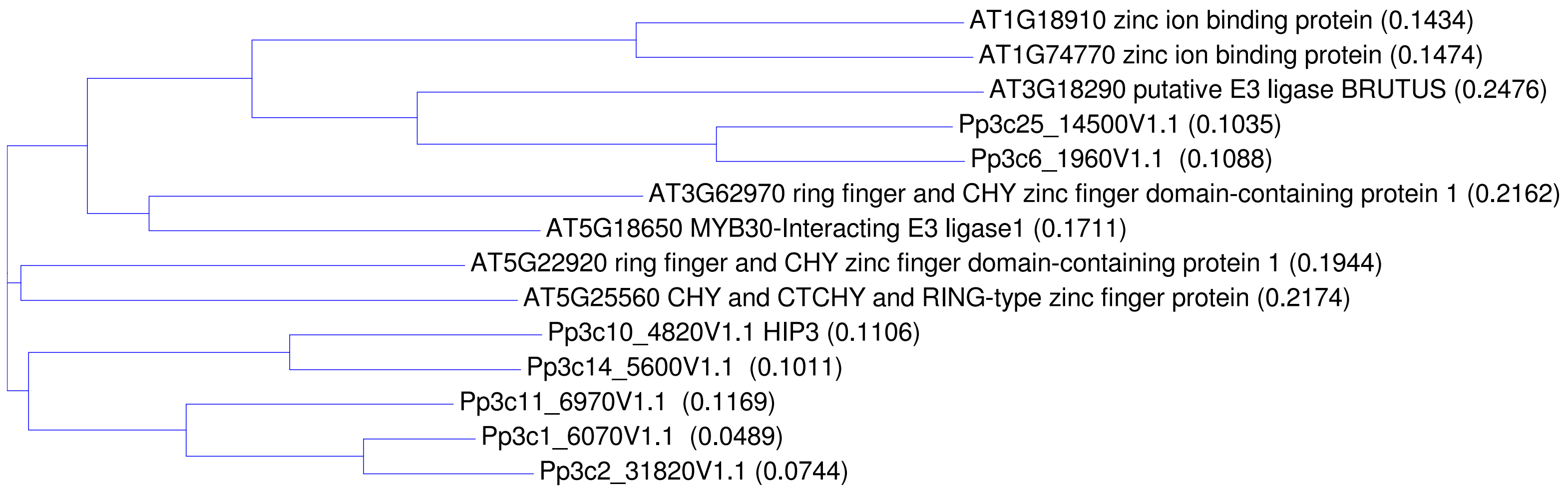

HIP3 alignment

	(1)	1 10 20 30 40 50 62
AT1G18910 zinc ion binding protein	(1)	------------------MGVGDPLPLPPEKNRREVNKPPDIASTSSSSASAVNNARLSDAP
AT1G74770 zinc ion binding protein	(1)	------------------MGGGNLHSLPPEN-----------ASVSASYAVTVGNTKLSDAP
AT3G18290 putative E3 ligase BRUTUS	(1)	MATPLPDFETARGGGAVASSSTTVLPSSVSSS--------SSSSRPLPVANSFSDDAEEISP
Pp3c25_14500V1.1	(1)	--------------------MATSVALPQSFQ--------PISTPLITALGMGMSCVPLASP
Pp3c6_1960V1.1	(1)	--------------------MATSVALPQTFQ--------PVPTPLITALGMGASCVPLASP
AT3G62970 ring finger and CHY zf-containing protein 1	(1)	--
AT5G18650 MYB30-Interacting E3 ligase1	(1)	--
AT5G22920 ring finger and CHY zf-containing protein 1	(1)	--
AT5G25560 CHY and CTCHY and RING-type zf protein	(1)	--
Pp3c10_4820V1.1 HIP3	(1)	--
Pp3c14_5600V1.1	(1)	--
Pp3c11_6970V1.1	(1)	--
Pp3c1_6070V1.1	(1)	--
Pp3c2_31820V1.1	(1)	--
Consensus	(1)	

	(63)	63 70 80 90 100 110 124
AT1G18910 zinc ion binding protein	(45)	ILLFVYFHKAFRAQLAELQFLAGDTVRSG---SDLAVELRSKFEFLKLVYKYHSAAEDEVIF
AT1G74770 zinc ion binding protein	(34)	VLFFVYCHKAFRAQLVELRRFATDAAEADSFSGDLAVELSRKFEFLKLVYKYHSAAEDEVIF
AT3G18290 putative E3 ligase BRUTUS	(55)	ILIFLFFHKAVCSELEALHRLALEFATGH---HVDLRLLRERYRFLRSIYKHHCNAEDEVIF
Pp3c25_14500V1.1	(35)	IIQFVFFHKAIRVEVGRLHRDALAVENGT---EEDIHALIDRYRFLRTIYKHFCSAEDEVIL
Pp3c6_1960V1.1	(35)	ITEFVFFHKAIRVEMDRLHRDALAVENGT---DKDIQNFIDRYIFLRTVYKHYCSAEDEVIL
AT3G62970 ring finger and CHY zf-containing protein 1	(1)	--
AT5G18650 MYB30-Interacting E3 ligase1	(1)	--
AT5G22920 ring finger and CHY zf-containing protein 1	(1)	--
AT5G25560 CHY and CTCHY and RING-type zf protein	(1)	--
Pp3c10_4820V1.1 HIP3	(1)	--
Pp3c14_5600V1.1	(1)	--
Pp3c11_6970V1.1	(1)	--
Pp3c1_6070V1.1	(1)	--
Pp3c2_31820V1.1	(1)	--
Consensus	(63)	

		125 130 140 150 160 170 186
	(125)	
AT1G18910 zinc ion binding protein	(104)	SALDTRVKNIVFNYSLEHDATDDLFTSVFHWLNVLEEEQGNRA-DVLREVVLCIGTIQSSIC
AT1G74770 zinc ion binding protein	(96)	LALDKRVKNIVSNYSLEHAGTDDLFTSIFHWLHVLEEEIGSRS-DVLREVILCIGTIQSSIC
AT3G18290 putative E3 ligase BRUTUS	(114)	SALDIRVKNVAQTYSLEHKGESNLFDHLFELLNSATETD--ES--YRRELARSTGALQTSVS
Pp3c25_14500V1.1	(94)	PALDSRVKNVAHSYSLEHKVESDQFDQIAQLLSNPLAEDGRVASSLHSQLGSCTEALHTTLC
Pp3c6_1960V1.1	(94)	PALDSRVKNVAHSYSLEHKVESDLFDQIASLLSAALAEKGKVASTLHSDLVCCTQALHTTLC
AT3G62970 ring finger and CHY zf-containing protein 1	(1)	--
AT5G18650 MYB30-Interacting E3 ligase1	(1)	--
AT5G22920 ring finger and CHY zf-containing protein 1	(1)	--
AT5G25560 CHY and CTCHY and RING-type zf protein	(1)	--
Pp3c10_4820V1.1 HIP3	(1)	--
Pp3c14_5600V1.1	(1)	--
Pp3c11_6970V1.1	(1)	--
Pp3c1_6070V1.1	(1)	--
Pp3c2_31820V1.1	(1)	--
Consensus	(125)	

		187 200 210 220 230 248
	(187)	
AT1G18910 zinc ion binding protein	(165)	QHMLKEERQVFPLMIENFSFEEQASLVWQFICSVPVMVLEEIFPWMTSLLSPKEKSEVETCF
AT1G74770 zinc ion binding protein	(157)	QHMLKEERQVFPLLIEKFSFREQASLVWQFICSVPVMVLEDFLPWMISHLSHEEKIEVENCI
AT3G18290 putative E3 ligase BRUTUS	(172)	QHLAKEQKQVFPLLIEKFKYEEQAYIVWRFLCSIPVNMLAVFLPWISSSISVDESKEMQTCL
Pp3c25_14500V1.1	(156)	QHLSKEEEQVFPLLMQHFTYKEQAGLVWQFICCIPVNLMEKFLPWLASSLSDEDRQQMVAVM
Pp3c6_1960V1.1	(156)	QHLAKEEEQVFPLLMQHFTYKEQTGLVWQFICSIPVNLLEKFLPWLASSLSDEERQQMVAVM
AT3G62970 ring finger and CHY zf-containing protein 1	(1)	--
AT5G18650 MYB30-Interacting E3 ligase1	(1)	--
AT5G22920 ring finger and CHY zf-containing protein 1	(1)	--
AT5G25560 CHY and CTCHY and RING-type zf protein	(1)	--
Pp3c10_4820V1.1 HIP3	(1)	--
Pp3c14_5600V1.1	(1)	--
Pp3c11_6970V1.1	(1)	--
Pp3c1_6070V1.1	(1)	--
Pp3c2_31820V1.1	(1)	--
Consensus	(187)	

```
                                                      (249) 249       260       270       280       290       300       310
AT1G18910 zinc ion binding protein                    (227) KEVVPNELSLQLVINSWLIDDSQSSLTALTKIMKGVQSVEVSENMTNSQTNSSSSGVFQRFW
AT1G74770 zinc ion binding protein                    (219) KDVAPNEDSLQQVISSWLLDDSQSSCGTPTEIMKGVQYVNVSKSLKKSPESHPSSGCFQRFW
AT3G18290 putative E3 ligase BRUTUS                    (234) KKIVPGEKLLQQVIFTWLGGKSNTVASCRIEDSMFQCCLDSSSSMLPCKAS--REQCACEGS
Pp3c25_14500V1.1                                      (218) CEVVPPEELLQQVVLAWLRGGNRAAVSDDTLKSSRDAESAAWAAIDSRVAGDLAMTWELDRE
Pp3c6_1960V1.1                                        (218) REVVPPEELLQQVILAWLRGGNRAIDAYNCPRSDLDAKAVAWASIDSKVAG--DLTWALDRD
AT3G62970 ring finger and CHY  zf-containing protein 1  (1) --------------------------------------------------------------
AT5G18650 MYB30-Interacting E3 ligase1                  (1) --------------------------------------------------------------
AT5G22920 ring finger and CHY  zf-containing protein 1  (1) --------------------------------------------------------------
AT5G25560 CHY and CTCHY and RING-type zf protein        (1) --------------------------------------------------------------
Pp3c10_4820V1.1 HIP3                                    (1) --------------------------------------------------------------
Pp3c14_5600V1.1                                         (1) --------------------------------------------------------------
Pp3c11_6970V1.1                                         (1) --------------------------------------------------------------
Pp3c1_6070V1.1                                          (1) --------------------------------------------------------------
Pp3c2_31820V1.1                                         (1) --------------------------------------------------------------
Consensus                                             (249)

                                                      (311) 311      320       330       340       350       360         372
AT1G18910 zinc ion binding protein                    (289) QWSKKMSFSSPNTGHI----LVHGIHLWHNAIRKDLVDIQKGLCQLTFPS--LSLDLNVLVV
AT1G74770 zinc ion binding protein                    (281) EWSKKS-LSIPNVGRS----PIHGLRLFQNAIEKDLRDIQEGLCQAKFQT--LILDLDVLMA
AT3G18290 putative E3 ligase BRUTUS                    (294) KIGKRKYPELTNFGSSDTLHPVDEIKLWHKSINKEMKEIADEARKIQLSG--DFSDLSAFDE
Pp3c25_14500V1.1                                      (280) SSMKKTDLQTKSLKSGPLVSPLRELLYWHNAIRKELKEIAEEAAQIQPLGALSPAKLTAFIE
Pp3c6_1960V1.1                                        (278) SSVNQGNLHPKLLRSRPLVSPLKELLYWHNAIRKELQEIAEQARQIQPRGGLSLAKLTAFIE
AT3G62970 ring finger and CHY  zf-containing protein 1  (1) --------------------------------------------------------------
AT5G18650 MYB30-Interacting E3 ligase1                  (1) --------------------------------------------------------------
AT5G22920 ring finger and CHY  zf-containing protein 1  (1) --------------------------------------------------------------
AT5G25560 CHY and CTCHY and RING-type zf protein        (1) --------------------------------------------------------------
Pp3c10_4820V1.1 HIP3                                    (1) --------------------------------------------------------------
Pp3c14_5600V1.1                                         (1) --------------------------------------------------------------
Pp3c11_6970V1.1                                         (1) --------------------------------------------------------------
Pp3c1_6070V1.1                                          (1) --------------------------------------------------------------
Pp3c2_31820V1.1                                         (1) --------------------------------------------------------------
Consensus                                             (311)
```

```
                                                     (373) 373    380       390       400       410       420           434
              AT1G18910 zinc ion binding protein     (345) RLNFLADVLIFYSNAFKTFFYPVFEDMVDQQHSSSSKQFTIDGHVENFKKSLDLETRAG---
              AT1G74770 zinc ion binding protein     (336) RLNFLADVLVSYSNAFKKFFHPVLEEMTARRSS-TAKQFNIDDCLENFQRLLYKSADDKT--
       AT3G18290 putative E3 ligase BRUTUS           (354) RLQYIAEVCIFHSLAEDKIIFP----AVDGEFSFSEEHDEEENQFNEFRCLIENIKSAGASS
                              Pp3c25_14500V1.1       (342) RSQFLADVCNFQSSAEDKLMCPTLHQKVQERVTYLMDHAEKDRRFEDVLCLLKGVQAAVNNS
                                Pp3c6_1960V1.1       (340) RSQFLADVCNFQSSAEDKLMCPSLHKKVQERVTYLMDHAEKDRRFEDVLRLLEGVQDAVNNS
AT3G62970 ring finger and CHY  zf-containing protein 1 (1) --------------------------------------------------------------
      AT5G18650 MYB30-Interacting E3 ligase1          (1) --------------------------------------------------------------
AT5G22920 ring finger and CHY  zf-containing protein 1 (1) --------------------------------------------------------------
   AT5G25560 CHY and CTCHY and RING-type zf protein  (1) --------------------------------------------------------------
                         Pp3c10_4820V1.1 HIP3        (1) --------------------------------------------------------------
                               Pp3c14_5600V1.1       (1) --------------------------------------------------------------
                               Pp3c11_6970V1.1       (1) --------------------------------------------------------------
                                Pp3c1_6070V1.1       (1) --------------------------------------------------------------
                               Pp3c2_31820V1.1       (1) --------------------------------------------------------------
                                     Consensus     (373)

                                                     (435) 435  440       450       460       470       480             496
              AT1G18910 zinc ion binding protein     (404) --SDNFVITLQEKLESLILTVAKQFSIEETEVFPIISKNCNIEMQRQLLYRSIHFLPLGLLK
              AT1G74770 zinc ion binding protein     (395) -KTDNFLLQLQEELESLIIQVTKQFAIQRTEVFPIISKNCNHEMQKQLLYTSIHVLPLGLLK
       AT3G18290 putative E3 ligase BRUTUS           (412) TSAAEFYTKLCSHADQIMETIQRHFHNEEIQVLPLARKNFSFKRQQELLYQSLCIMPLRLIE
                              Pp3c25_14500V1.1       (404) TTVTELHRELCEKAELIVESIQQHLLEEEE-VFRFTKSQCSIEEQRALLYQSLRVMPLKLLE
                                Pp3c6_1960V1.1       (402) ATVAQLHSELCKRAELIVESIQQHLLEEEE-VLHYPQSHCSVEEQRVLLYRSLRLMPLKLLE
AT3G62970 ring finger and CHY zf-containing protein 1  (1) --------------------------------------------------------------
      AT5G18650 MYB30-Interacting E3 ligase1          (1) --------------------------------------------------------------
AT5G22920 ring finger and CHY  zf-containing protein 1 (1) --------------------------------------------------------------
   AT5G25560 CHY and CTCHY and RING-type zf protein  (1) --------------------------------------------------------------
                         Pp3c10_4820V1.1 HIP3        (1) --------------------------------------------------------------
                               Pp3c14_5600V1.1       (1) --------------------------------------------------------------
                               Pp3c11_6970V1.1       (1) --------------------------------------------------------------
                                Pp3c1_6070V1.1       (1) --------------------------------------------------------------
                               Pp3c2_31820V1.1       (1) --------------------------------------------------------------
                                     Consensus     (435)
```

```
                                                   (497) 497         510       520       530       540               558
AT1G18910 zinc ion binding protein                 (464) CVIMWFSAQLPEDECQSIIHYLSSEDSFPNKPFAHLLLQWFRFGYSGKTPVESFWNELSFMF
AT1G74770 zinc ion binding protein                 (456) CVILWFSAHLSEEESQSILHFLSLEDSSPKKSFPRLLLQWLRFGYSGKTSVERFWKQLDVMF
AT3G18290 putative E3 ligase BRUTUS                 (474) RVLPWLTASLTEDEAKNFLKNLQAGAPKSDVALVTLFSGWACKGR-------KAGECLS---
Pp3c25_14500V1.1                                   (465) RVLPWLVGVLNEDEAKEMLQNLRLAAPAEDMALVTLFSGWACKGHPQNLSSTGNIRCLSSTK
Pp3c6_1960V1.1                                     (463) RVLPWLVAILSEDETKVMLENLRLAAPVEDNALVTLFSDWACKGHPKHFSSTGSIRCLS-TS
AT3G62970 ring finger and CHY  zf-containing protein 1 (1) --------------------------------------------------------------
AT5G18650 MYB30-Interacting E3 ligase1              (1) --------------------------------------------------------------
AT5G22920 ring finger and CHY  zf-containing protein 1 (1) --------------------------------------------------------------
AT5G25560 CHY and CTCHY and RING-type zf protein   (1) --------------------------------------------------------------
Pp3c10_4820V1.1 HIP3                                 (1) --------------------------------------------------------------
Pp3c14_5600V1.1                                      (1) --------------------------------------------------------------
Pp3c11_6970V1.1                                      (1) --------------------------------------------------------------
Pp3c1_6070V1.1                                       (1) --------------------------------------------------------------
Pp3c2_31820V1.1                                      (1) --------------------------------------------------------------
Consensus                                          (497)

                                                   (559) 559         570       580       590       600       610       620
AT1G18910 zinc ion binding protein                 (526) KPRCSFEEELTEEASGSFFQQSPQKLFKVSDPYS------------------------MDPP
AT1G74770 zinc ion binding protein                 (518) KVRCSCQKEHTEEASGSFSNQTQLQLCKVSKDVYP-----------RKKDKSSTCFMSMDLA
AT3G18290 putative E3 ligase BRUTUS                 (526) PNGNGLCPVKTLSNIKEVNLQSCNACASVPCTSR--------STKSCCQHQDKRPAKRTAVL
Pp3c25_14500V1.1                                   (527) VDDCPVMRMKLNAGIGSKYLQSGSILPPPDSNQNSVDVGTVHGPDDSLETLHTRAPKRPRWG
Pp3c6_1960V1.1                                     (524) QDECSVKRMKFNAGSRPVNMQNG---STIPSKHNSVDMGTVHGPDDSPEIFDTRASKRPRGS
AT3G62970 ring finger and CHY  zf-containing protein 1 (1) --------------------------------------------------------------
AT5G18650 MYB30-Interacting E3 ligase1              (1) --------------------------------------------------------------
AT5G22920 ring finger and CHY  zf-containing protein 1 (1) --------------------------------------------------------------
AT5G25560 CHY and CTCHY and RING-type zf protein   (1) --------------------------------------------------------------
Pp3c10_4820V1.1 HIP3                                 (1) --------------------------------------------------------------
Pp3c14_5600V1.1                                      (1) --------------------------------------------------------------
Pp3c11_6970V1.1                                      (1) --------------------------------------------------------------
Pp3c1_6070V1.1                                       (1) --------------------------------------------------------------
Pp3c2_31820V1.1                                      (1) --------------------------------------------------------------
Consensus                                          (559)
```

```
                                                    (621) 621      630       640       650       660       670         682
AT1G18910 zinc ion binding protein                  (564) AGYMNETPYSSAMNQQILIPGKLRPLLHLPDLFG----------------------------
AT1G74770 zinc ion binding protein                  (569) VGDMYETPYSSRMNQQMTFSGKLKPPLHLPDFFG----------------------------
AT3G18290 putative E3 ligase BRUTUS                 (580) SCEKKTTPHSTEVANGCKPSGNGRSCCVPDLGVNNNCLELGSLPAAKAMRSSSLNSAAPALN
Pp3c25_14500V1.1                                    (589) EVEFKGGDNLREVDKSRDRCGGSSACCAPGLGCG----SISARSSGTALLSYSTSSASSSLS
Pp3c6_1960V1.1                                      (583) EFEDASIDYPTKDTTSKNSCEGAARCCAPGLGCG----SLTTRPSGLAIKPYTASLNSSSSN
AT3G62970 ring finger and CHY  zf-containing protein 1 (1) --------------------------------------------------------------
AT5G18650 MYB30-Interacting E3 ligase1                (1) --------------------------------------------------------------
AT5G22920 ring finger and CHY  zf-containing protein 1 (1) --------------------------------------------------------------
AT5G25560 CHY and CTCHY and RING-type zf protein      (1) --------------------------------------------------------------
Pp3c10_4820V1.1 HIP3                                  (1) --------------------------------------------------------------
Pp3c14_5600V1.1                                       (1) --------------------------------------------------------------
Pp3c11_6970V1.1                                       (1) --------------------------------------------------------------
Pp3c1_6070V1.1                                        (1) --------------------------------------------------------------
Pp3c2_31820V1.1                                       (1) --------------------------------------------------------------
Consensus                                           (621)

                                                    (683) 683    690       700       710       720       730           744
AT1G18910 zinc ion binding protein                  (598) -------DKTIGEHLTMDLKPIDLIFYFHKAMKKDLDYLVRGS-ARLATDYSFLGEFQQRFH
AT1G74770 zinc ion binding protein                  (603) -------EKNMDDPMIMDVKPIDLLFFFHKAMKMDLDYLVCGS-TRLAADFRFLAEFQQRFH
AT3G18290 putative E3 ligase BRUTUS                 (642) SSLFIWEMDSNSFGTGHAERPVATIFKFHKAISKDLEFLDVESGKLIDCDGTFIRQFIGRFH
Pp3c25_14500V1.1                                    (647) SGLFGWGSDRGLTSG-SGPKPIDHIFQFHKAIRKDLEYLDSESAKLADCDEDFLRQFQGRFQ
Pp3c6_1960V1.1                                      (641) SELFGWVNDSGPTCD-PGPKPIDHIFQFHKAIRKDLEYLDSESAKLADCDEDFLRQFQGRFQ
AT3G62970 ring finger and CHY  zf-containing protein 1 (1) --------------------------------------------------------------
AT5G18650 MYB30-Interacting E3 ligase1                (1) --------------------------------------------------------------
AT5G22920 ring finger and CHY zf-containing protein 1  (1) --------------------------------------------------------------
AT5G25560 CHY and CTCHY and RING-type zf protein      (1) --------------------------------------------------------------
Pp3c10_4820V1.1 HIP3                                  (1) --------------------------------------------------------------
Pp3c14_5600V1.1                                       (1) --------------------------------------------------------------
Pp3c11_6970V1.1                                       (1) --------------------------------------------------------------
Pp3c1_6070V1.1                                        (1) --------------------------------------------------------------
Pp3c2_31820V1.1                                       (1) --------------------------------------------------------------
Consensus                                           (683)
```

```
                                                       (745) 745  750       760       770       780       790            806
                     AT1G18910 zinc ion binding protein (652) LIKFLYQIHSDAEDEIAFPALEAKGKLQNISQSYSIDHELEVEHLNKVSFLLNELAELNMLV
                     AT1G74770 zinc ion binding protein (657) MIKFLYQIHSDAEDEIAFPALEAKGQLKNISHSFSIDHELETKHFDKVSFILNEMSELNMLV
                AT3G18290 putative E3 ligase BRUTUS (704) LLWGFYKAHSNAEDDILFPALESKETLHNVSHSYTLDHKQEEKLFGDIYSVLTELSILHEKL
                            Pp3c25_14500V1.1 (708) FLWGLYRAHSNAEDDIVFPALEAKEALHNVSHSYTIDHKQEEQLFKDIAEVRILAN------
                              Pp3c6_1960V1.1 (702) FLWGLYRAHSNAEDDIVFPALEAKEALHNVSHSYTIDHKQEEQLFKDIAEVLVELSTLHTRM
AT3G62970 ring finger and CHY  zf-containing protein 1 (1) --------------------------------------------------------------
            AT5G18650 MYB30-Interacting E3 ligase1 (1) --------------------------------------------------------------
AT5G22920 ring finger and CHY  zf-containing protein 1 (1) --------------------------------------------------------------
   AT5G25560 CHY and CTCHY and RING-type zf protein (1) --------------------------------------------------------------
                      Pp3c10_4820V1.1 HIP3 (1) --------------------------------------------------------------
                          Pp3c14_5600V1.1 (1) --------------------------------------------------------------
                          Pp3c11_6970V1.1 (1) --------------------------------------------------------------
                           Pp3c1_6070V1.1 (1) --------------------------------------------------------------
                          Pp3c2_31820V1.1 (1) --------------------------------------------------------------
                                Consensus (745)

                                                       (807) 807          820       830       840       850                 868
                     AT1G18910 zinc ion binding protein (714) LDHKN----------------------------VKYEKLCMSLQDICKSIHKLLSEHLHREE
                     AT1G74770 zinc ion binding protein (719) STINTTAADHDR---K-----------------MKYERLCLSLREICKSMHKLLSEHIQHEE
                AT3G18290 putative E3 ligase BRUTUS (766) QSDSMMEDIAQTDTVRTDIDNGDCNKKYN--------ELATKLQGMCKSIKITLDQHIFLEE
                            Pp3c25_14500V1.1 (764) -----------------------------------------KLQRMCKSIKISLDHHVTREE
                              Pp3c6_1960V1.1 (764) HTQAIPTFTSDMENGKDELKQDCMEKETSKAEMIQRRALAGKLQRMCKSIRVSLDHHVSREE
AT3G62970 ring finger and CHY  zf-containing protein 1 (1) --------------------------------------------------------------
            AT5G18650 MYB30-Interacting E3 ligase1 (1) --------------------------------------------------------------
 AT5G22920 ring finger and CHY zf-containing protein 1 (1) --------------------------------------------------------------
   AT5G25560 CHY and CTCHY and RING-type zf protein (1) --------------------------------------------------------------
                      Pp3c10_4820V1.1 HIP3 (1) --------------------------------------------------------------
                          Pp3c14_5600V1.1 (1) --------------------------------------------------------------
                          Pp3c11_6970V1.1 (1) --------------------------------------------------------------
                           Pp3c1_6070V1.1 (1) --------------------------------------------------------------
                          Pp3c2_31820V1.1 (1) --------------------------------------------------------------
                                Consensus (807)
```

```
                                                       (869) 869        880       890       900       910       920       930
AT1G18910 zinc ion binding protein                     (748) TELWCLFRDCFTIEEQEKIIACMLGRISGEILQDMIPWLMESLIPDEQHAVMSLWRQATRKT
AT1G74770 zinc ion binding protein                     (761) TELWGLFRNCFSIEEQEKIIGCMLGRISGEILQDMIPWLMESLTSDEQLAAMSLWRQATRKT
AT3G18290 putative E3 ligase BRUTUS                     (820) LELWPLFDKHFSIQEQDKIVGRIIGTTGAEVLQSMLPWVTSALSEDEQNRMMDTWKQATKNT
Pp3c25_14500V1.1                                       (785) EELWPLFDVHFSIEEQDEIVGRIIGTTGAEVLQSMLPWVTTALTEDEQNIMMDTLRQATRNT
Pp3c6_1960V1.1                                         (826) HELWPLFDVHFSIEEQDQIVGRIIGTTGAEVLQSMLPWITTALSEHEQNIMMDTLRQATRNT
AT3G62970 ring finger and CHY  zf-containing protein 1   (1) --------------------------------------------------------------
AT5G18650 MYB30-Interacting E3 ligase1                   (1) --------------------------------------------------------------
AT5G22920 ring finger and CHY  zf-containing protein 1   (1) --------------------------------------------------------------
AT5G25560 CHY and CTCHY and RING-type zf protein         (1) --------------------------------------------------------------
Pp3c10_4820V1.1 HIP3                                     (1) --------------------------------------------------------------
Pp3c14_5600V1.1                                          (1) --------------------------------------------------------------
Pp3c11_6970V1.1                                          (1) --------------------------------------------------------------
Pp3c1_6070V1.1                                           (1) --------------------------------------------------------------
Pp3c2_31820V1.1                                          (1) --------------------------------------------------------------
Consensus                                              (869)

                                                       (931) 931      940       950       960       970       980         992
AT1G18910 zinc ion binding protein                     (810) MFGEWLTEWYNSHAVEEETEEANKDPSENSDPLDVVWSYLFEGAADEYKG--SICSKPLEET
AT1G74770 zinc ion binding protein                     (823) MFVEWLTEWYNGHVLQEEAGEANNDPFGDSDPLEIVWKYLFEASADGEKGSMRSSLLKLPKT
AT3G18290 putative E3 ligase BRUTUS                     (882) MFDEWLNECWKGSPDSSSTETSKP-----------------------------SPQ------
Pp3c25_14500V1.1                                       (847) MFDKWLRAWWKDNPASSSDTTISTENQSVPSASSSESLQMVVDYFSKEAISVGTTTE-----
Pp3c6_1960V1.1                                         (888) MFDKWLQAWWKNNPTSNSNTVESSEKHSVPHMGTSESLQMVVDYLSKGVVDTGEVTGDEKLR
AT3G62970 ring finger and CHY zf-containing protein 1    (1) --------------------------------------------------------------
AT5G18650 MYB30-Interacting E3 ligase1                   (1) --------------------------------------------------------------
AT5G22920 ring finger and CHY zf-containing protein 1    (1) --------------------------------------------------------------
AT5G25560 CHY and CTCHY and RING-type zf protein         (1) --------------------------------------------------------------
Pp3c10_4820V1.1 HIP3                                     (1) --------------------------------------------------------------
Pp3c14_5600V1.1                                          (1) --------------------------------------------------------------
Pp3c11_6970V1.1                                          (1) --------------------------------------------------------------
Pp3c1_6070V1.1                                           (1) --------------------------------------------------------------
Pp3c2_31820V1.1                                          (1) --------------------------------------------------------------
Consensus                                              (931)
```

```
                                                      (993)  993    1000      1010      1020      1030      1040          1054
                AT1G18910 zinc ion binding protein    (870)  ELKGIMNKPLGKAAPNNKVEFGNKEENHLEISGSKKVCTGADETKYKEQTDSNAQAFQMSHN
                AT1G74770 zinc ion binding protein    (885)  NFTGIMNQPP--PN--YKVEVGKKEEKDLERSESKKICRGSNQEGDKEQTDK------MSQK
              AT3G18290 putative E3 ligase BRUTUS    (909)  -----------------------------KDNDHQEILDQSG--------------------
                               Pp3c25_14500V1.1    (904)  -------ESSIHSGSGFPSNICSDDEDRQVTTMDKAITDQGSK-------------------
                                 Pp3c6_1960V1.1    (950)  TLDACNHEGVIQSDSVHANTCGTDDEDMHAANLSKPTADHANR-------------------
AT3G62970 ring finger and CHY zf-containing protein 1 (1)  --------------------------------------------------------------
           AT5G18650 MYB30-Interacting E3 ligase1    (1)  --------------------------------------------------------------
AT5G22920 ring finger and CHY zf-containing protein 1 (1)  --------------------------------------------------------------
   AT5G25560 CHY and CTCHY and RING-type zf protein   (1)  --------------------------------------------------------------
                          Pp3c10_4820V1.1 HIP3        (1)  --------------------------------------------------------------
                                Pp3c14_5600V1.1       (1)  --------------------------------------------------------------
                                Pp3c11_6970V1.1       (1)  --------------------------------------------------------------
                                 Pp3c1_6070V1.1       (1)  --------------------------------------------------------------
                                Pp3c2_31820V1.1       (1)  -----------------------------------------------------MAYVSLHLL
                                      Consensus     (993)

                                                     (1055)  1055 1060      1070      1080      1090      1100            1116
                AT1G18910 zinc ion binding protein    (932)  TSQSGQDSRYECLLSMSQEDVEATIRRISRDSSLDPQKKSYIIQNLLMSRWIATQRIYNLEP
                AT1G74770 zinc ion binding protein    (937)  VSQFGPSKKYEQLLTMSEEELVVVIKKISCDSSLDPQKKDYIKQNLLMSRWNISQRTYNLEP
              AT3G18290 putative E3 ligase BRUTUS    (922)  ---ELFKPGWKDIFRMNQNELEAEIRKVYQDSTLDPRRKDYLVQNWRTSRWIAAQQKLPKEA
                               Pp3c25_14500V1.1    (940)  --DAVFKPGWSDIFRMNQKELEAAIRKVSSDSSLDPRRKAYLMQNLMTSRWIASQQHVSHEK
                                 Pp3c6_1960V1.1    (993)  --YAVFKPGWRDIFRMNQIELEAAIRKVSSDSSLDPRRKAYLMQNLMTSRWIAAQQLVSNEM
AT3G62970 ring finger and CHY zf-containing protein 1 (1)  -----------------------------------------------------MGGSASLQS
           AT5G18650 MYB30-Interacting E3 ligase1    (1)  --------------------------------------------------------------
AT5G22920 ring finger and CHY zf-containing protein 1 (1)  --------------------------------------MDMGFHE----------NEQNQ--
   AT5G25560 CHY and CTCHY and RING-type zf protein   (1)  -------------MGDVYFNHFAQQLQMSDQE--K---GEMSRHS-------HPHSINEESE
                          Pp3c10_4820V1.1 HIP3        (1)  -------------MAVLAVRMEALLSPLMSGEQFRYAYNNVAAHSHHHVVAGHTSCLDSDVE
                                Pp3c14_5600V1.1       (1)  -------------MAVVAVRMESLLGPLVNGEQFRHGYSNVGAHSRQPVVAAHTGCSDSDSE
                                Pp3c11_6970V1.1       (1)  --------------------------------------------------------------
                                 Pp3c1_6070V1.1       (1)  ----------------------------------------MTTFE-------GEEKALTQDV
                                Pp3c2_31820V1.1      (10)  TRHDHLSQNLSPPPHFNPFFFRYSPDALSPFKASLPEQSNMADFE-------GEEKVLAQDV
                                      Consensus    (1055)                 M    L A I   L                               E
```

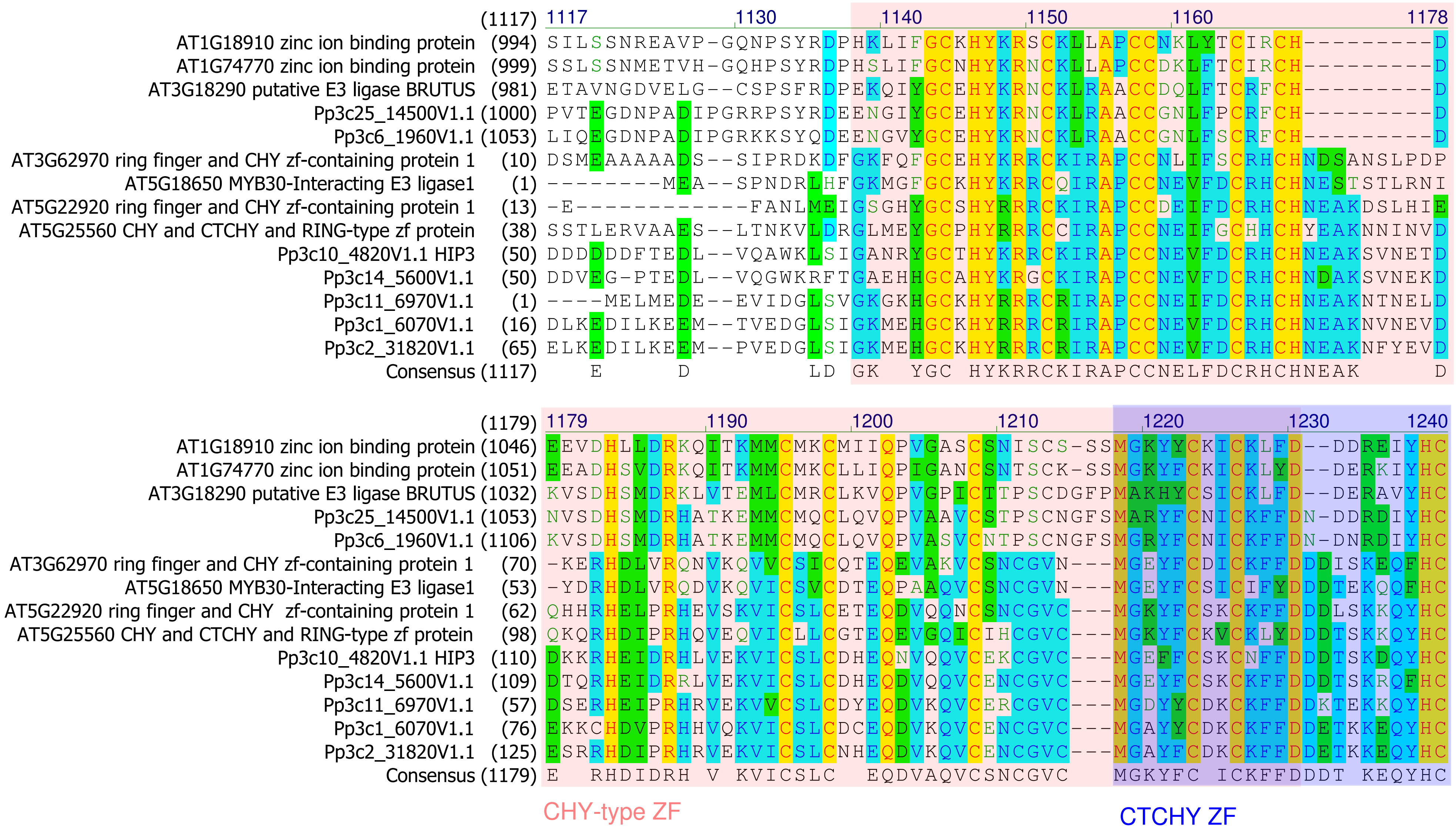
(1117) 1117 1130 1140 1150 1160 1178
AT1G18910 zinc ion binding protein (994) SILSSNREAVP-GQNPSYRDPHKLIFGCKHYKRSCKLLAPCCNKLYTCIRCH---------D
AT1G74770 zinc ion binding protein (999) SSLSSNMETVH-GQHPSYRDPHSLIFGCNHYKRNCKLLAPCCDKLFTCIRCH---------D
AT3G18290 putative E3 ligase BRUTUS (981) ETAVNGDVELG--CSPSFRDPEKQIYGCEHYKRNCKLRAACCDQLFTCRFCH---------D
Pp3c25_14500V1.1 (1000) PVTEGDNPADIPGRRPSYRDEENGIYGCEHYKRNCKLRAACCGNLFPCRFCH---------D
Pp3c6_1960V1.1 (1053) LIQEGDNPADIPGRKKSYQDEENGVYGCEHYKRNCKLRAACCGNLFSCRFCH---------D
AT3G62970 ring finger and CHY zf-containing protein 1 (10) DSMEAAAAADS--SIPRDKDFGKFQFGCEHYKRRCKIRAPCCNLIFSCRHCHNDSANSLPDP
AT5G18650 MYB30-Interacting E3 ligase1 (1) --------MEA--SPNDRLHFGKMGFGCKHYKRRCQIRAPCCNEVFDCRHCHNESTSTLRNI
AT5G22920 ring finger and CHY zf-containing protein 1 (13) -E------------FANLMEIGSGHYGCSHYRRRCKIRAPCCDEIFDCRHCHNEAKDSLHIE
AT5G25560 CHY and CTCHY and RING-type zf protein (38) SSTLERVAAES--LTNKVLDRGLMEYGCPHYRRRCCIRAPCCNEIFGCHHCHYEAKNNINVD
Pp3c10_4820V1.1 HIP3 (50) DDDDDDFTEDL--VQAWKLSIGANRYGCTHYKRRCKIRAPCCNEVFDCRHCHNEAKSVNETD
Pp3c14_5600V1.1 (50) DDVEG-PTEDL--VQGWKRFTGAEHHGCAHYKRGCKIRAPCCNEVFDCRHCHNDAKSVNEKD
Pp3c11_6970V1.1 (1) ----MELMEDE--EVIDGLSVGKGKHGCKHYRRRCRIRAPCCNEIFDCRHCHNEAKNTNELD
Pp3c1_6070V1.1 (16) DLKEDILKEEM--TVEDGLSIGKMEHGCKHYRRRCRIRAPCCNEVFDCRHCHNEAKNVNEVD
Pp3c2_31820V1.1 (65) ELKEDILKEEM--PVEDGLSIGKMEHGCKHYRRRCRIRAPCCNEVFDCRHCHNEAKNFYEVD
Consensus (1117) E D LD GK YGC HYKRRCKIRAPCCNELFDCRHCHNEAK D
(1179) 1179 1190 1200 1210 1220 1230 1240
AT1G18910 zinc ion binding protein (1046) EEVDHLLDRKQITKMMCMKCMIIQPVGASCSNISCS-SSMGKYYCKICKLFD--DDREIYHC
AT1G74770 zinc ion binding protein (1051) EEADHSVDRKQITKMMCMKCLLIQPIGANCSNTSCK-SSMGKYFCKICKLYD--DERKIYHC
AT3G18290 putative E3 ligase BRUTUS (1032) KVSDHSMDRKLVTEMLCMRCLKVQPVGPICTTPSCDGFPMAKHYCSICKLFD--DERAVYHC
Pp3c25_14500V1.1 (1053) NVSDHSMDRHATKEMMCMQCLQVQPVAAVCSTPSCNGFSMARYFCNICKFFDN-DDRDIYHC
Pp3c6_1960V1.1 (1106) KVSDHSMDRHATKEMMCMQCLQVQPVASVCNTPSCNGFSMGRYFCNICKFFDN-DNRDIYHC
AT3G62970 ring finger and CHY zf-containing protein 1 (70) -KERHDLVRQNVKQVVCSICQTEQEVAKVCSNCGVN---MGEYFCDICKFFDDDISKEQFHC
AT5G18650 MYB30-Interacting E3 ligase1 (53) -YDRHDLVRQDVKQVICSVCDTEQPAAQVCSNCGVN---MGEYFCSICIFYDDDTEKQQFHC
AT5G22920 ring finger and CHY zf-containing protein 1 (62) QHHRHELPRHEVSKVICSLCETEQDVQQNCSNCGVC---MGKYFCSKCKFFDDDLSKKQYHC
AT5G25560 CHY and CTCHY and RING-type zf protein (98) QKQRHDIPRHQVEQVICLLCGTEQEVGQICIHCGVC---MGKYFCKVCKLYDDDTSKKQYHC
Pp3c10_4820V1.1 HIP3 (110) DKKRHEIDRHLVEKVICSLCDHEQNVQQVCEKCGVC---MGEFFCSKCNFFDDDTSKDQYHC
Pp3c14_5600V1.1 (109) DTQRHEIDRRLVEKVICSLCDHEQDVQQVCENCGVC---MGEYFCSKCKFFDDDTSKRQFHC
Pp3c11_6970V1.1 (57) DSERHEIPRHRVEKVVCSLCDYEQDVKQVCERCGVC---MGDYYCDKCKFFDDKTEKKQYHC
Pp3c1_6070V1.1 (76) EKKCHDVPRHHVQKVICSLCDCEQDVKQVCENCGVC---MGAYYCDKCKFFDDETKKEQYHC
Pp3c2_31820V1.1 (125) ESRRHDIPRHRVEKVICSLCNHEQDVKQVCENCGVC---MGAYFCDKCKFFDDETKKEQYHC
Consensus (1179) E RHDIDRH V KVICSLC EQDVAQVCSNCGVC MGKYFC ICKFFDDDT KEQYHC
CHY-type ZF
CTCHY ZF

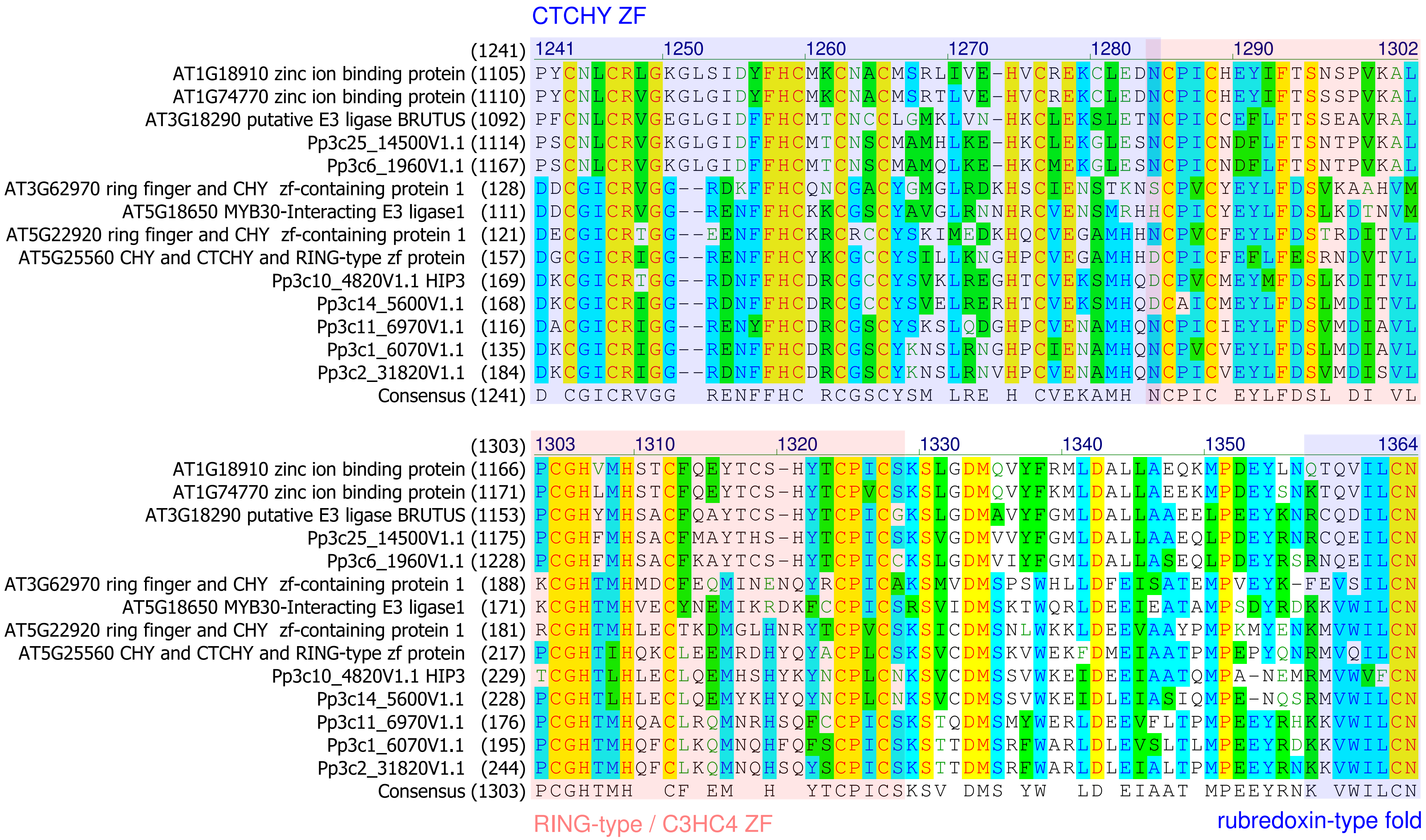
CTCHY ZF
(1241) 1241 1250 1260 1270 1280 1290 1302
AT1G18910 zinc ion binding protein (1105) PYCNLCRLGKGLSIDYFHCMKCNACMSRLIVE-HVCREKCLEDNCPICHEYIFTSNSPVKAL
AT1G74770 zinc ion binding protein (1110) PYCNLCRVGKGLGIDYFHCMKCNACMSRTLVE-HVCREKCLEDNCPICHEYIFTSSSPVKAL
AT3G18290 putative E3 ligase BRUTUS (1092) PFCNLCRVGEGLGIDFFHCMTCNCCLGMKLVN-HKCLEKSLETNCPICCEFLFTSSEAVRAL
Pp3c25_14500V1.1 (1114) PSCNLCRVGKGLGIDFFHCMTCNSCMAMHLKE-HKCLEKGLESNCPICNDFLFTSNTPVKAL
Pp3c6_1960V1.1 (1167) PSCNLCRVGKGLGIDFFHCMTCNSCMAMQLKE-HKCMEKGLESNCPICNDFLFTSNTPVKAL
AT3G62970 ring finger and CHY zf-containing protein 1 (128) DDCGICRVGG--RDKFFHCQNCGACYGMGLRDKHSCIENSTKNSCPVCYEYLFDSVKAAHVM
AT5G18650 MYB30-Interacting E3 ligase1 (111) DDCGICRVGG--RENFFHCKKCGSCYAVGLRNNHRCVENSMRHHCPICYEYLFDSLKDTNVM
AT5G22920 ring finger and CHY zf-containing protein 1 (121) DECGICRTGG--EENFFHCKRCRCCYSKIMEDKHQCVEGAMHHNCPVCFEYLFDSTRDITVL
AT5G25560 CHY and CTCHY and RING-type zf protein (157) DGCGICRIGG--RENFFHCYKCGCCYSILLKNGHPCVEGAMHHDCPICFEFLFESRNDVTVL
Pp3c10_4820V1.1 HIP3 (169) DKCGICRTGG--RDNFFHCDRCGCCYSVKLREGHTCVEKSMHQDCPVCMEYMFDSLKDITVL
Pp3c14_5600V1.1 (168) DKCGICRIGG--RDNFFHCDRCGCCYSVELRERHTCVEKSMHQDCAICMEYLFDSLMDITVL
Pp3c11_6970V1.1 (116) DACGICRIGG--RENYFHCDRCGSCYSKSLQDGHPCVENAMHQNCPICIEYLFDSVMDIAVL
Pp3c1_6070V1.1 (135) DKCGICRIGG--RENFFHCDRCGSCYKNSLRNGHPCIENAMHQNCPVCVEYLFDSLMDIAVL
Pp3c2_31820V1.1 (184) DKCGICRIGG--RDNFFHCDRCGSCYKNSLRNVHPCVENAMHQNCPICVEYLFDSVMDISVL
Consensus (1241) D CGICRVGG RENFFHC RCGSCYSM LRE H CVEKAMH NCPIC EYLFDSL DI VL
(1303) 1303 1310 1320 1330 1340 1350 1364
AT1G18910 zinc ion binding protein (1166) PCGHVMHSTCFQEYTCS-HYTCPICSKSLGDMQVYFRMLDALLAEQKMPDEYLNQTQVILCN
AT1G74770 zinc ion binding protein (1171) PCGHLMHSTCFQEYTCS-HYTCPVCSKSLGDMQVYFKMLDALLAEEKMPDEYSNKTQVILCN
AT3G18290 putative E3 ligase BRUTUS (1153) PCGHYMHSACFQAYTCS-HYTCPICGKSLGDMAVYFGMLDALLAAEELPEEYKNRCQDILCN
Pp3c25_14500V1.1 (1175) PCGHFMHSACFMAYTHS-HYTCPICSKSVGDMVVYFGMLDALLAAEQLPDEYRNRCQEILCN
Pp3c6_1960V1.1 (1228) PCGHFMHSACFKAYTCS-HYTCPICCKSLGDMVIYFGMLDALLASEQLPDEYRSRNQEILCN
AT3G62970 ring finger and CHY zf-containing protein 1 (188) KCGHTMHMDCFEQMINENQYRCPICAKSMVDMSPSWHLLDFEISATEMPVEYK-FEVSILCN
AT5G18650 MYB30-Interacting E3 ligase1 (171) KCGHTMHVECYNEMIKRDKFCCPICSRSVIDMSKTWQRLDEEIEATAMPSDYRDKKVWILCN
AT5G22920 ring finger and CHY zf-containing protein 1 (181) RCGHTMHLECTKDMGLHNRYTCPVCSKSICDMSNLWKKLDEEVAAYPMPKMYENKMVWILCN
AT5G25560 CHY and CTCHY and RING-type zf protein (217) PCGHTIHQKCLEEMRDHYQYACPLCSKSVCDMSKVWEKFDMEIAATPMPEPYQNRMVQILCN
Pp3c10_4820V1.1 HIP3 (229) TCGHTLHLECLQEMHSHYKYNCPLCNKSVCDMSSVWKEIDEEIAATQMPA-NEMRMVWVFCN
Pp3c14_5600V1.1 (228) PCGHTLHLECLQEMYKHYQYNCPLCNKSVCDMSSVWKEIDLEIASIQMPE-NQSRMVWILCN
Pp3c11_6970V1.1 (176) PCGHTMHQACLRQMNRHSQFCCPICSKSTQDMSMYWERLDEEVFLTPMPEEYRHKKVWILCN
Pp3c1_6070V1.1 (195) PCGHTMHQFCLKQMNQHFQFSCPICSKSTTDMSRFWARLDLEVSLTLMPEEYRDKKVWILCN
Pp3c2_31820V1.1 (244) PCGHTMHQFCLKQMNQHSQYSCPICSKSTTDMSRFWARLDLEIALTPMPEEYRNKKVWILCN
Consensus (1303) PCGHTMH CF EM H YTCPICSKSV DMS YW LD EIAAT MPEEYRNK VWILCN
RING-type / C3HC4 ZF
rubredoxin-type fold

```
                                                      (1365) 1365 1370      1380      1390      1400      1410           1426
AT1G18910 zinc ion binding protein                    (1227) DCGRKGNAPYHWLYHKCSSCASYNTRLF----------------------------------
AT1G74770 zinc ion binding protein                    (1232) DCGRKGNAPYHWLYHKCTTCGSYNSRLL----------------------------------
AT3G18290 putative E3 ligase BRUTUS                    (1214) DCERKGTTRFHWLYHKCGSCGSYNTRVIKSETIPPDCSTSS---------------------
Pp3c25_14500V1.1                                      (1236) DCEQKSTAPFHWLYHKCEGCGSYNTRTI----------------------------------
Pp3c6_1960V1.1                                        (1289) DCEQKATAPFHWLYHKCGGCGSYNTRAI----------------------------------
AT3G62970 ring finger and CHY zf-containing protein 1  (249)  DCNKGSKAMFHILGHKCSDCGSYNTRRISTPQDPVSETE-----------------------
AT5G18650 MYB30-Interacting E3 ligase1                (233)  DCNDTTEVHFHIIGQKCGHCRSYNTRAIAPPVLPQ---------------------------
AT5G22920 ring finger and CHY zf-containing protein 1  (243)  DCGSNTNVRFHLIAHKCSSCGSYNTRQTQRGSDSHSCSSGMPQVVGSTG-------------
AT5G25560 CHY and CTCHY and RING-type zf protein      (279)  DCGKKAEVQYHVVAQKCPNCKSYNTRQTRG--------------------------------
Pp3c10_4820V1.1 HIP3                                  (290)  DCGATNEVQYHHVGQKCGTCPSYNTRPTDAPASLASSRS-----------------------
Pp3c14_5600V1.1                                       (289)  DCGAKNEVRYHVVGQKCGTCPSYNTRLTESPASRTPS-------------------------
Pp3c11_6970V1.1                                       (238)  DCGTTSDVNYHVIGQKCPECGSYNTRSTCAPSTGRLAEPMSPDGVSPESVGPSISTSVGSLP
Pp3c1_6070V1.1                                        (257)  DCGRTSDVHYHVLGHKCAGCGSYNTRSTPPPSTSR---------------------------
Pp3c2_31820V1.1                                       (306)  DCGTTCDVYYHVLGHKCAGCGSYNTRSASPPSSSRRVDRTLSLVGSLSDQMGNLTTVAEISA
Consensus                                             (1365) DCG K  EV YHVLGHKCG CGSYNTR    P
                                                                                rubredoxin-type fold

                                                      (1427) 1427 1431
AT1G18910 zinc ion binding protein                    (1255) -----
AT1G74770 zinc ion binding protein                    (1260) -----
AT3G18290 putative E3 ligase BRUTUS                    (1255) -----
Pp3c25_14500V1.1                                      (1264) -----
Pp3c6_1960V1.1                                        (1317) -----
AT3G62970 ring finger and CHY zf-containing protein 1  (288)  -----
AT5G18650 MYB30-Interacting E3 ligase1                (268)  -----
AT5G22920 ring finger and CHY zf-containing protein 1  (292)  -----
AT5G25560 CHY and CTCHY and RING-type zf protein      (309)  -----
Pp3c10_4820V1.1 HIP3                                  (329)  -----
Pp3c14_5600V1.1                                       (326)  -----
Pp3c11_6970V1.1                                       (300)  NEMGT
Pp3c1_6070V1.1                                        (292)  -----
Pp3c2_31820V1.1                                       (368)  E----
Consensus                                             (1427)
```
