# Supplementary material for: Holophytochrome-Interacting Proteins in Physcomitrella: Putative Actors in Phytochrome Cytoplasmic Signaling
Source: Front Plant Sci. 2016 May 12;7:613. doi: 10.3389/fpls.2016.00613 (PMC4867686; doi:10.3389/fpls.2016.00613)
Supplement: Supplementary file 2 [file Data_Sheet_2.ZIP › SI/SI HIP4.pdf]

frontiers

Supplementary Material

Holophytochrome-interacting proteins in *Physcomitrella*: putative actors in phytochrome cytoplasmic signaling

Anna Lena Ermert, Katharina Mailliet, and Jon Hughes*

*** Correspondence:** jon.hughes@uni-giessen.de

HIP4/PRL1 (Pp3c16_8560E1.1)

```
ATGCCGGCCTCGGGGGGAGGCAGCACCGAGGTGCTGGAGGCTGTGGAGCCCCAGTCCCTGAAGAAGCTCTCCCTCAAGTCC
TTGAAGCGCGCCTTGGATATCTTCGCGCCTACTCATGGCGATCGCAACGGCGCGATGCCTGAGAGTTGCAAAATTCGTATC
AGTTGCAAGGTGAATGATGAGTATGCTGCTGTCAAGGACATGCCGGCTGCAAGCACTAGGGAGAATGTTGGTGCAAAACCA
GGAGATAATGGACTCCAAAGCTTGCGAGTCCCTGGCTCAGAGCAGCCTACTACAAATGCAGGACCAGCATCTGGAAAAGGA
GGAGGCAAGCAACTTGCATTGGTGCCTGCTCGGGCTCAACCTCCAGTTCCAGCACCGGTTGTTGGTGCTGCTCCTGCAGCT
CCTGCTGTGGATCGTACTTCTACAGCTATTGCATCGACTGACCGACATCAACCAAGCGCTGCGGTTATGAAGAGGCTTCCT
AGTAAATGGCCAAAGCCTGTCTGGCATCCACCTTGGGTGAACTATAGGGTTATTAGTGGGCACTTAGGGTGGGTGCGGTCG
ATAGCATTTGATCCTGGAAATGAATGGTTTTGTACAGGCTCAGCTGATAGGACCATCAAGATTTGGGATAGTGGAACTGGA
CAGCTGAAACTTACTTTAACTGGCCATATCGAGCAAGTTCGAGGCCTTGCAGTGAGCGCCCGGCATCCTTATTTATTCTCA
GCTGGTGATGACAAACAGGTCAAATGTTGGGATCTTGAATACAATAAGGTTATTCGGTCATATCATGGTCATTTGAGTGGA
GTTTATTGCTTAGCTCTTCATCCTACACTTGATATTCTCATGACTGGGGGTCGAGATTCAGTGTGCCGAGTATGGGACATT
AGAACCAAGGCCCAGGTGTTTGCATTATCAGGACATGAGAACACGGTCTGCTCTGTGATTACGCAGGCAACTGATCCACAA
GTTGTAACAGGATCACACGACACAACTATTAAACTTTGGGATCTTGCTGCAGGAAAAACTATGTCGACGCTGACATTTCAC
AAAAAGTCAGTCAGAGCATTAGCGATGCATCCTTTTGAACATACTTTTACTTCAGCGTCAGCGGACAACATTAAGAAGTTC
CGGCTACCAAAGGGAGACTTTTTGCATAACATGCTTTCTCAGCAGCGGACAATTGTTAACTGCATGTCTATCAACGAAGAT
AATGTAATGGTATCAGCAGGTGATAATGGAAGTTTGTGGTTTTGGGATTACAAAAGTGGGCATAACTTCCAGCAAGCGCAG
ACAATCGTGCAGCCTGGTTCTCTGGATAGTGAGGCCGGAATTTATGCTCTTTCGTATGATCAGACAGGGTCTCGGTTGATT
ACGTGTGAAGCAGACAAGACTATTAAATTTTGGAAGGAAGACGAAACAGCTACACCAGAATCACATCCTGTACACTTTAGG
CCGCCGAAGGACATGCGCCGGTTCTAA
```

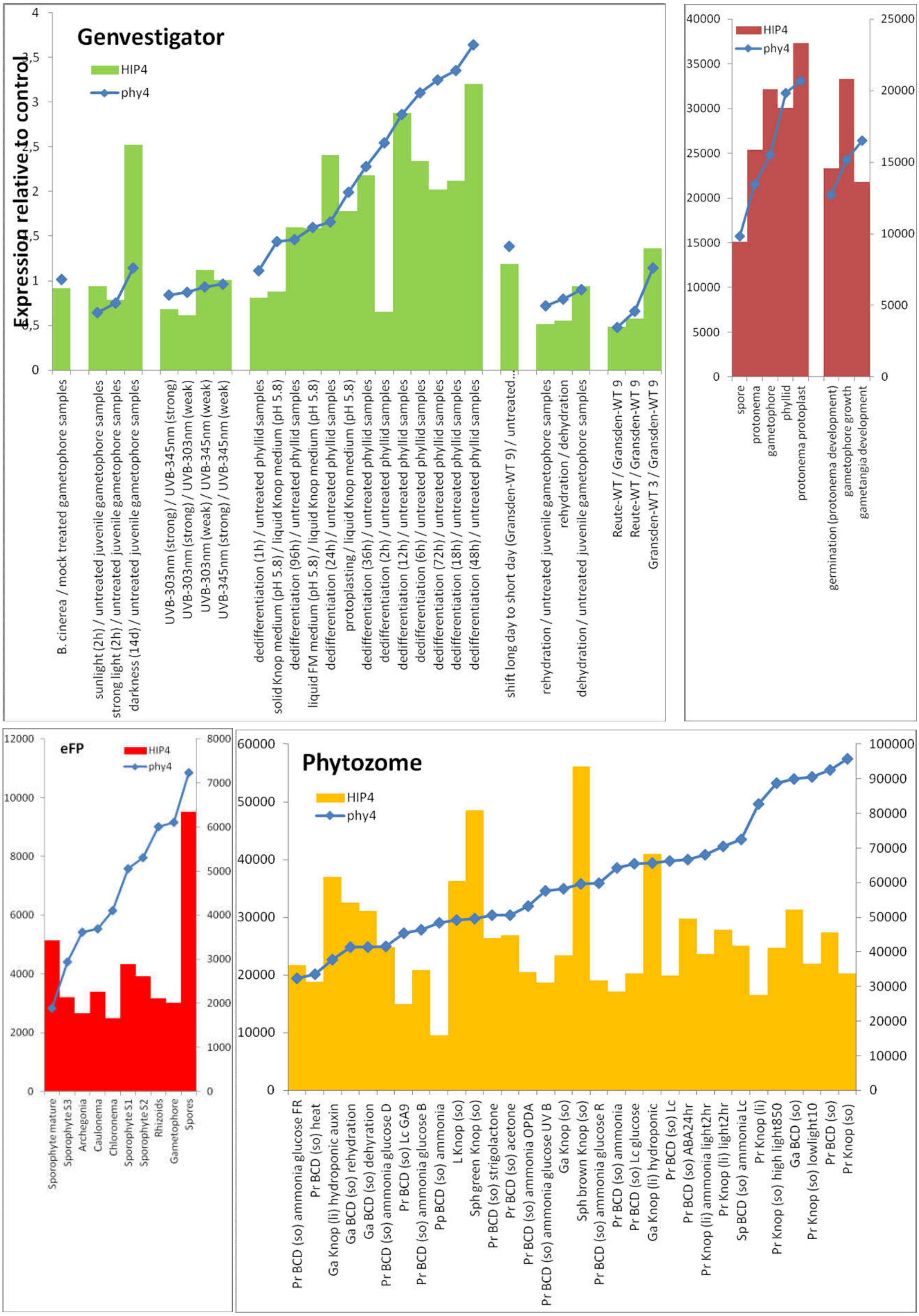
Genvestigator
Expression relative to control
HIP4
phy4
B. cinerea / mock treated gametophore samples
sunlight (2h) / untreated juvenile gametophore samples
strong light (2h) / untreated juvenile gametophore samples
darkness (14d) / untreated juvenile gametophore samples
UVB-303nm (strong) / UVB-345nm (strong)
UVB-303nm (strong) / UVB-303nm (weak)
UVB-303nm (weak) / UVB-345nm (weak)
UVB-345nm (strong) / UVB-345nm (weak)
dedifferentiation (1h) / untreated phyllid samples
solid Knop medium (pH 5.8) / liquid Knop medium (pH 5.8)
dedifferentiation (96h) / untreated phyllid samples
liquid FM medium (pH 5.8) / liquid Knop medium (pH 5.8)
dedifferentiation (24h) / untreated phyllid samples
protoplasting / liquid Knop medium (pH 5.8)
dedifferentiation (36h) / untreated phyllid samples
dedifferentiation (2h) / untreated phyllid samples
dedifferentiation (12h) / untreated phyllid samples
dedifferentiation (6h) / untreated phyllid samples
dedifferentiation (72h) / untreated phyllid samples
dedifferentiation (18h) / untreated phyllid samples
dedifferentiation (48h) / untreated phyllid samples
shift long day to short day (Gransden-WT 9) / untreated...
rehydration / untreated juvenile gametophore samples
rehydration / dehydration
dehydration / untreated juvenile gametophore samples
Reute-WT / Gransden-WT 9
Reute-WT / Gransden-WT 9
Gransden-WT 3 / Gransden-WT 9
HIP4
phy4
spore
protonema
gametophore
phyllid
protonema protoplast
germination (protonema development)
gametophore growth
gametangia development
eFP
HIP4
phy4
Sporophyte mature
Sporophyte S3
Archegonia
Caulonema
Chloronema
Sporophyte S1
Sporophyte S2
Rhizoids
Gametophore
Spores
Phytozome
HIP4
phy4
Pr BCD (so) ammonia glucose FR
Pr BCD (so) heat
Ga Knop (li) hydroponic auxin
Ga BCD (so) rehydration
Ga BCD (so) dehyration
Pr BCD (so) ammonia glucose D
Pr BCD (so) Lc GA9
Pr BCD (so) ammonia glucose B
Pp BCD (so) ammonia
L Knop (so)
Sph green Knop (so)
Pr BCD (so) strigolactone
Pr BCD (so) acetone
Pr BCD (so) ammonia OPDA
Pr BCD (so) ammonia glucose UV B
Ga Knop (so)
Sph brown Knop (so)
Pr BCD (so) ammonia glucose R
Pr BCD (so) ammonia
Pr BCD (so) Lc glucose
Ga Knop (li) hydroponic
Pr BCD (so) Lc
Pr BCD (so) ABA24hr
Pr Knop (li) ammonia light2hr
Pr Knop (li) light2hr
Sp BCD (so) ammonia Lc
Pr Knop (li)
Pr Knop (so) high light850
Ga BCD (so)
Pr Knop (so) lowlight10
Pr BCD (so)
Pr Knop (so)

HIP4 / PRL1 alignment tree

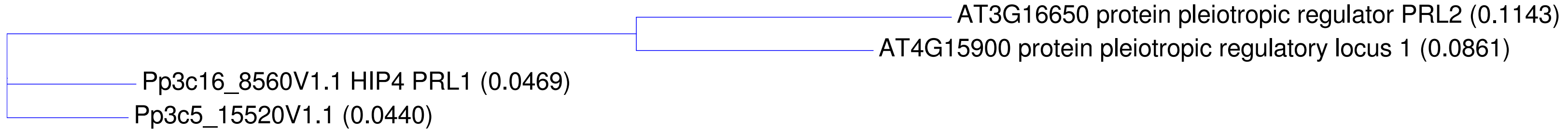

HIP4 / PRL1 alignment

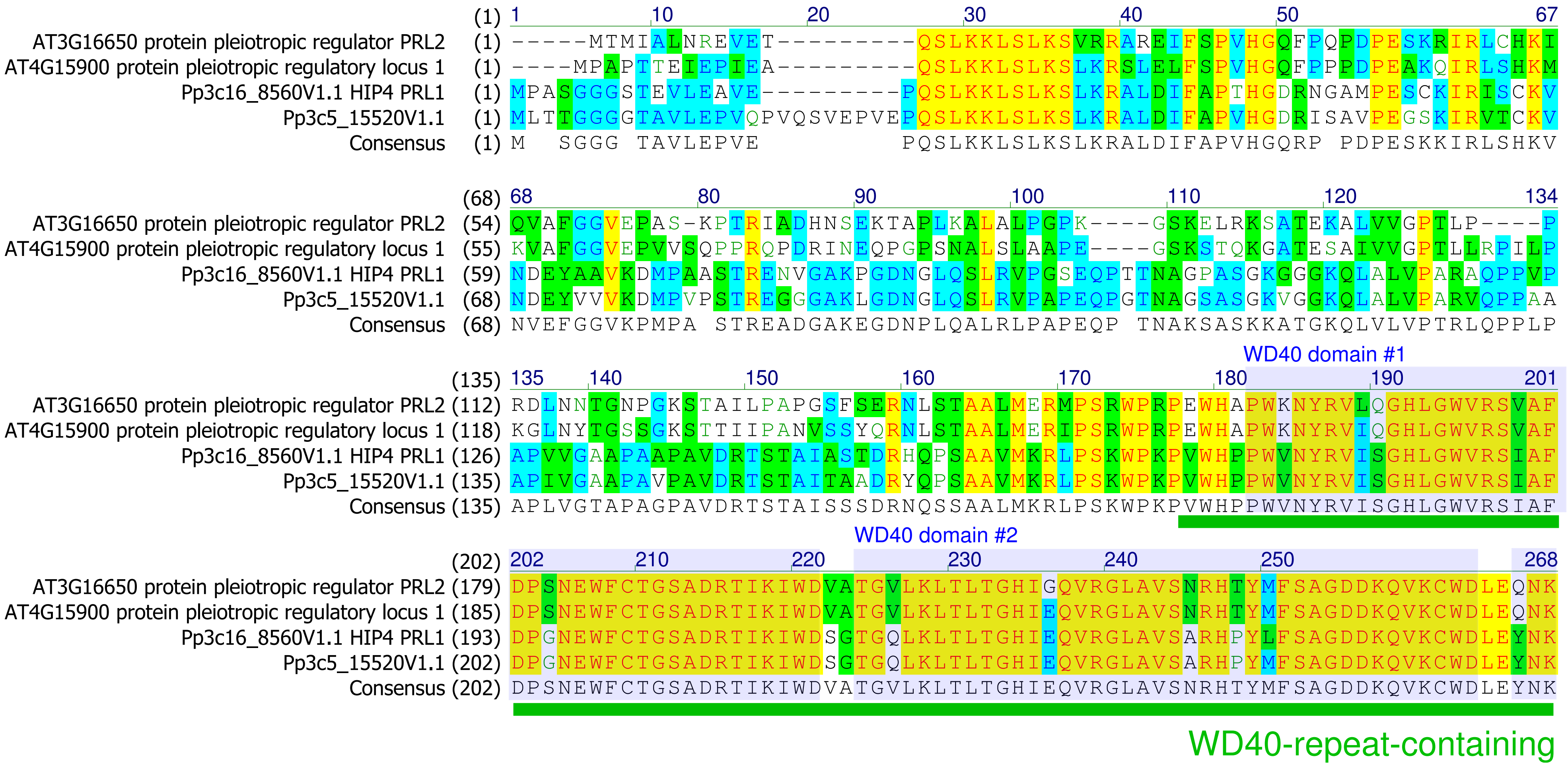

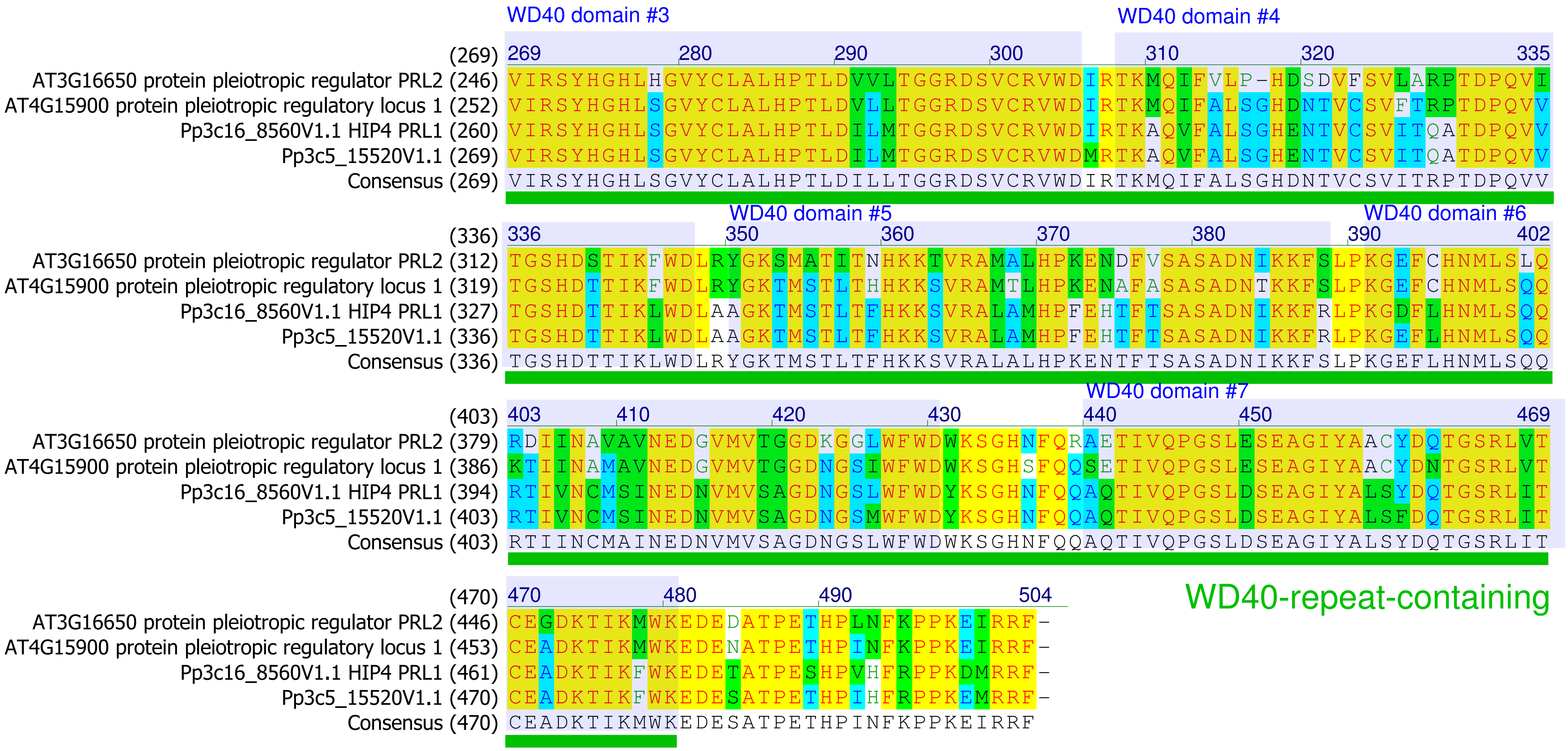

WD40 domain #3
WD40 domain #4
(269) 269 280 290 300 310 320 335
AT3G16650 protein pleiotropic regulator PRL2 (246) VIRSYHGLHGVYCLALHPTLDVVLTGGRDSVCRVWDIRTKMQIFVLP-HDSDVFSVLARPTDPQVI
AT4G15900 protein pleiotropic regulatory locus 1 (252) VIRSYHGLSGVYCLALHPTLDVLLTGGRDSVCRVWDIRTKMQIFALSGHDNTVCSVFTRPTDPQVV
Pp3c16_8560V1.1 HIP4 PRL1 (260) VIRSYHGLSGVYCLALHPTLDILMTGGRDSVCRVWDIRTKAQVFALSGHENTVCSVITQATDPQVV
Pp3c5_15520V1.1 (269) VIRSYHGLSGVYCLALHPTLDILMTGGRDSVCRVWDMRTKAQVFALSGHENTVCSVITQATDPQVV
Consensus (269) VIRSYHGLSGVYCLALHPTLDILLTGGRDSVCRVWDIRTKMQIFALSGHDNTVCSVITRPTDPQVV
WD40 domain #5
WD40 domain #6
(336) 336 350 360 370 380 390 402
AT3G16650 protein pleiotropic regulator PRL2 (312) TGSHDSTIKFWDLRYGKSMATITNHKKTVRAMALHPKENDFVSASADNIKKFSLPKGEFCHNMLSLQ
AT4G15900 protein pleiotropic regulatory locus 1 (319) TGSHDTTIKFWDLRYGKTMSTLTHHKKSVRAMTLHPKENAFASASADNTKKFSLPKGEFCHNMLSQQ
Pp3c16_8560V1.1 HIP4 PRL1 (327) TGSHDTTIKLWDLAAGKTMSTLTFHKKSVRALAMHPFEHTFTSASADNIKKFRLPKGDFLHNMLSQQ
Pp3c5_15520V1.1 (336) TGSHDTTIKLWDLAAGKTMSTLTFHKKSVRALAMHPFEHTFTSASADNIKKFRLPKGEFLHNMLSQQ
Consensus (336) TGSHDTTIKLWDLRYGKTMSTLTFHKKSVRALALHPKENTFTSASADNIKKFSLPKGEFLHNMLSQQ
WD40 domain #7
(403) 403 410 420 430 440 450 469
AT3G16650 protein pleiotropic regulator PRL2 (379) RDIINAVAVNEDGVMVTGGDKGGLWFWDWKSGHNFQRAETIVQPGSLESEAGIYAACYDQTGSRLVT
AT4G15900 protein pleiotropic regulatory locus 1 (386) KTIINAMAVNEDGVMVTGGDNGSIWFWDWKSGHSFQQSETIVQPGSLESEAGIYAACYDNTGSRLVT
Pp3c16_8560V1.1 HIP4 PRL1 (394) RTIVNCMSINEDNVMVSAGDNGSLWFWDYKSGHNFQQAQTIVQPGSLDSEAGIYALSYDQTGSRLIT
Pp3c5_15520V1.1 (403) RTIVNCMSINEDNVMVSAGDNGSMWFWDYKSGHNFQQAQTIVQPGSLDSEAGIYALSFDQTGSRLIT
Consensus (403) RTIINCMAINEDNVMVSAGDNGSLWFWDWKSGHNFQQAQTIVQPGSLDSEAGIYALSYDQTGSRLIT
(470) 470 480 490 504
AT3G16650 protein pleiotropic regulator PRL2 (446) CEGDKTIKMWKEDEDATPETHPLNFKPPKEIRRF-
AT4G15900 protein pleiotropic regulatory locus 1 (453) CEADKTIKMWKEDENATPETHPINFKPPKEIRRF-
Pp3c16_8560V1.1 HIP4 PRL1 (461) CEADKTIKFWKEDETATPESHPVHFRPPKDMRRF-
Pp3c5_15520V1.1 (470) CEADKTIKFWKEDESATPETHPIHFRPPKEMRRF-
Consensus (470) CEADKTIKMWKEDESATPETHPINFKPPKEIRRF
WD40-repeat-containing
