# Supplementary material for: Holophytochrome-Interacting Proteins in Physcomitrella: Putative Actors in Phytochrome Cytoplasmic Signaling
Source: Front Plant Sci. 2016 May 12;7:613. doi: 10.3389/fpls.2016.00613 (PMC4867686; doi:10.3389/fpls.2016.00613)
Supplement: Supplementary file 2 [file Data_Sheet_2.ZIP › SI/SI HIP5.pdf]

Supplementary Material

Holophytochrome-interacting proteins in *Physcomitrella*: putative actors in phytochrome cytoplasmic signaling

Anna Lena Ermert, Katharina Mailliet, and Jon Hughes*

*** Correspondence:** jon.hughes@uni-giessen.de

HIP5 (Pp3c7_3040V1.1)

```
ATGGAAGGGCAGTCTGAAGTGCAGGAACAGAAGAGGAAGGCTACAGGGAAGAGGAGGCTGAGCAGTAGCAGCAGCAGCCGT
AGCAGCAGTAGTAGTAGTAACGGAAACGCTAGCGCGGACGTGGAGATGGCTACTGCGACCTTGTCGGTCCCGAAGGAGCCG
TGTAGATCGTTTTCGGGTAGGAAAAGGAGTAATCACAAAAATAAAAAAAGCGCTAAGAAGGATGCTGCGTCGCGGAATACT
CAGGGCGAGTGTGTTGAGTCTTCCTCTGTAGTGGATGCCTCGCCGTCTCAAGAGGCAAATCGGTCTACTAGTCAATCCCCC
TTGGATGCAAGCGAATCTTCCACTGTCAGTGACCCTTCCCCTGCACCTGAGCCGGAAGTGGCTATGGTGGTGGCGGCGGCT
TCGTCTAGAGAGGACGTCCCAATCATCCCTGGCCTGTCGGATAACACGGCGTTGGTCATCCTAGCACTTATACCGCTTTCT
TATCATCAACCTCTGAAGAGAGTGTGTAAGAAATGGCAAAGATGTTTGACAACTGTAGAGTCTACGAACGAGGTACTGGAC
ATGCGAAAGTTTCAAGGTGTGAAAGAGACGTGGGTATTCTTGCTGGCCAGTGCAAGACAACAGCGCCAGCAGTGGCGGGCC
TTTGACCCGGTGTACAACAGGTGGAGGTGTTTGCCACAATGCCCTTGTGATTACACGTTTAACAGCTGCGACAAGGAGTCT
GCAGTAGCAGGAACTCATCTCTTGGTAACTGGTCACAGTTCGACTGGTACGACGGTCTGGAGATATGATCTGCACACCAAC
GAATGGGGGAAAGCTGCCAAGATGCTGCAGAGTCGATGCTTGTTTGCATCTGCAAGTCATGGAAAGTATGCGTACTTCGCT
GGTGGATCATGTGAAGGTTCAGTCATAAGCTCGGCAGAGCGGTATAACTCTCAGACCAGAAAGTGGGAGCCACTTCCCGAT
CTCCATGTGAGTCGGAAATGGTGCTCCGGATGTATTTTGGATAACAAGTTCTTTGTGATAGGTGGGCAAGGTAGTGAAAAG
CAAGCGTTGACGTCAGGAGAGTATTACGATGAGTCGGAAAACAGATGGGTTATAGTTGAAAACATGTGGCCGGCTGCAAGG
ACACAGCCTCCAGGCCAAACAGCACCACCTCTAGTTGCTGTCGTAAAAGATCAACTCTATGCTGCTGATGCCAGCACCATG
GAACTTAATGCTTACCACAAAGGAACAAACACCTGGAGACCTCTAGGACCTGTTCCATATCGTAGTGTTGATGCTAGTGGA
TGGGGCATGGGGTTCAAGGCCGTGGGAGACGAGATTTTTGTAATTGGAGGGTCCAGTGACAGGGGAAACGGGACTTTTTGC
GACCAGATTCATGCATGGCCTCCCGCGCAAATGCAAAATGCGGATGGCTGGCGCCTGGTCGGCCAACTCCCCAACACAAGT
GGCTTCATCTATAATTGCGCTGTCATGATAGTTTGA
```

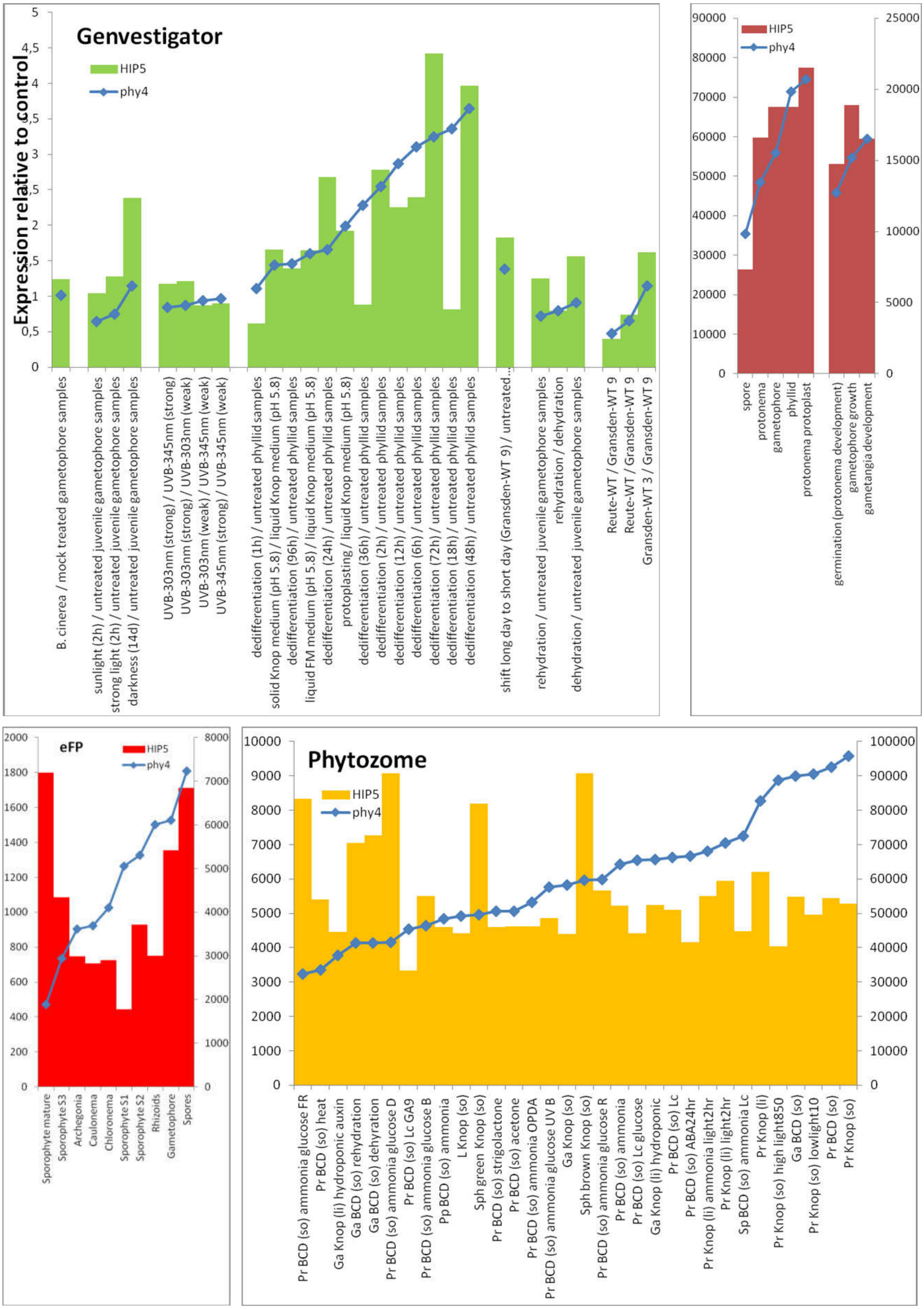
Genvestigator
HIP5
phy4
Expression relative to control
B. cinerea / mock treated gametophore samples
sunlight (2h) / untreated juvenile gametophore samples
strong light (2h) / untreated juvenile gametophore samples
darkness (14d) / untreated juvenile gametophore samples
UVB-303nm (strong) / UVB-345nm (strong)
UVB-303nm (strong) / UVB-303nm (weak)
UVB-303nm (weak) / UVB-345nm (weak)
UVB-345nm (strong) / UVB-345nm (weak)
dedifferentiation (1h) / untreated phyllid samples
solid Knop medium (pH 5.8) / liquid Knop medium (pH 5.8)
dedifferentiation (96h) / untreated phyllid samples
liquid FM medium (pH 5.8) / liquid Knop medium (pH 5.8)
dedifferentiation (24h) / untreated phyllid samples
protoplasting / liquid Knop medium (pH 5.8)
dedifferentiation (36h) / untreated phyllid samples
dedifferentiation (2h) / untreated phyllid samples
dedifferentiation (12h) / untreated phyllid samples
dedifferentiation (6h) / untreated phyllid samples
dedifferentiation (72h) / untreated phyllid samples
dedifferentiation (18h) / untreated phyllid samples
dedifferentiation (48h) / untreated phyllid samples
shift long day to short day (Gransden-WT 9) / untreated...
rehydration / untreated juvenile gametophore samples
rehydration / dehydration
dehydration / untreated juvenile gametophore samples
Reute-WT / Gransden-WT 9
Reute-WT / Gransden-WT 9
Gransden-WT 3 / Gransden-WT 9
HIP5
phy4
spore
protonema
gametophore
phyllid
protonema protoplast
germination (protonema development)
gametophore growth
gametangia development
eFP
HIP5
phy4
Sporophyte mature
Sporophyte S3
Archegonia
Caulonema
Chloronema
Sporophyte S1
Sporophyte S2
Rhizoids
Gametophore
Spores
Phytozome
HIP5
phy4
Pr BCD (so) ammonia glucose FR
Pr BCD (so) heat
Ga Knop (li) hydroponic auxin
Ga BCD (so) rehydration
Ga BCD (so) dehyration
Pr BCD (so) ammonia glucose D
Pr BCD (so) Lc GA9
Pr BCD (so) ammonia glucose B
Pp BCD (so) ammonia
L Knop (so)
Sph green Knop (so)
Pr BCD (so) strigolactone
Pr BCD (so) acetone
Pr BCD (so) ammonia OPDA
Pr BCD (so) ammonia glucose UV B
Ga Knop (so)
Sph brown Knop (so)
Pr BCD (so) ammonia glucose R
Pr BCD (so) ammonia
Pr BCD (so) Lc glucose
Ga Knop (li) hydroponic
Pr BCD (so) Lc
Pr BCD (so) ABA24hr
Pr Knop (li) ammonia light2hr
Pr Knop (li) light2hr
Sp BCD (so) ammonia Lc
Pr Knop (li)
Pr Knop (so) high light850
Ga BCD (so)
Pr Knop (so) lowlight10
Pr BCD (so)
Pr Knop (so)

HIP5 alignment tree

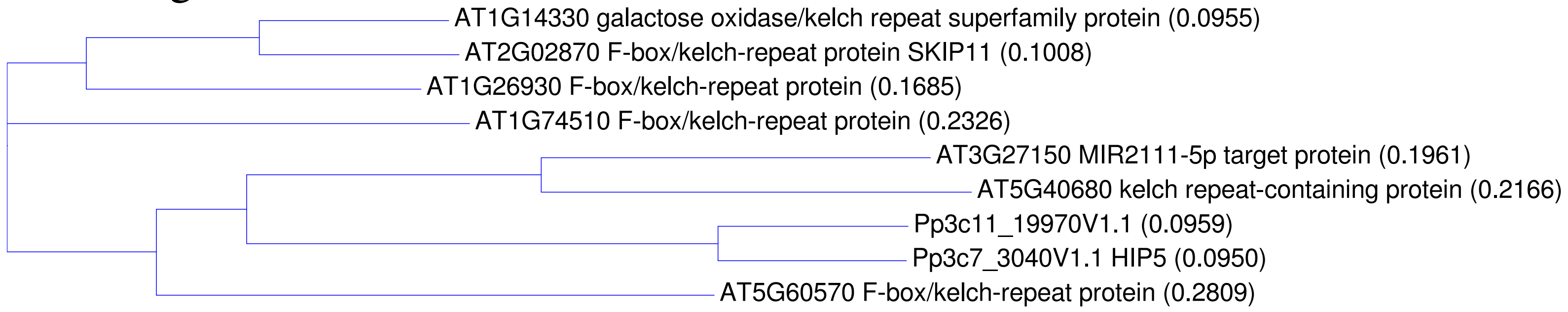

HIP5 alignment

```
                                                              (1)  1        10        20        30        40        50            64
AT1G14330 galactose oxidase/kelch repeat superfamily protein  (1)  -----MVEDR---TYLMSRIFSSSR------------LSESKWPYMYPQPEDSSESNLINGKRA
AT2G02870 F-box/kelch-repeat protein SKIP11                   (1)  -----MLEDRSPDSCLSTRVFSSSR------------LSESNWSNSYMYPEDD-DKLLGNGKRA
AT1G26930 F-box/kelch-repeat protein                          (1)  -----MFEGRPRDSCLVSTLFTMPS------------HKETKWSFLVS-----------GKRSF
AT1G74510 F-box/kelch-repeat protein                          (1)  -----MLEAP---SYLVSRDLPSSC------------EEESKWIYNAHCVLQLS----LRKRLL
AT3G27150 MIR2111-5p target protein                           (1)  -------------------MLTLGE------------DQIGRIGASSSWSRS-------KKLRV
AT5G40680 kelch repeat-containing protein                     (1)  ----------------MSLMLQKNQ------------ARGTRASLSQFEPRK-------LLIAS
Pp3c11_19970V1.1                                              (1)  MERQSEVQEAKRRATGKRRLRSSNN---CSSSCNGQGIGDVEMATATLSVPS---RSHGGRKKS
Pp3c7_3040V1.1 HIP5                                           (1)  MEGQSEVQEQKRKATGKRRLSSSSSSRSSSSSSNGNASADVEMATATLSVPKEPCRSFSGRKRS
AT5G60570 F-box/kelch-repeat protein                          (1)  --------------------------------------MAAEEHSN-------------KRREV
Consensus                                                     (1)        V      S   SRL SS                ESK S S                KKRS

                                                             (65)  65   70        80        90        100       110              128
AT1G14330 galactose oxidase/kelch repeat superfamily protein (45)  LENDVDELRQSKSPRLMGFSIHGNEAIEEDEQ--------------------EQDQSDSNNNG-
AT2G02870 F-box/kelch-repeat protein SKIP11                  (47)  LE-VVGEVRQTKSLKLMGFSIIYDSDSSDYSLSGGEEQADAAIGDGSSSRQEQEQQSDFNDNGG
AT1G26930 F-box/kelch-repeat protein                         (37)  LNNDESDLHFKKMYKLT------------------------------------TDSSEGEDNGS
AT1G74510 F-box/kelch-repeat protein                         (41)  DDTDVEGSSAKKMLRVDHGSRGESDKITDSLQLAKTYQ-------------------SSNQSQQGG
AT3G27150 MIR2111-5p target protein                          (27)  HRYEIPDLNVEPSLDWDGEETGEATKALSSTC------------------------LKPKDADYC
AT5G40680 kelch repeat-containing protein                    (30)  TKPTIPDLNAEPCSDSEEEETVEN---LTS--------------------------LAPQDAHN-
Pp3c11_19970V1.1                                             (59)  NHKKKKGAKKDAPCRNGLSERVETVSVVDASPSPDANRTTYLPLLGTR---ESLTGSDPGSPPE
Pp3c7_3040V1.1 HIP5                                          (65)  NHKNKKSAKKDAASRNTQGECVESSSVVDASPSQEANRSTSQSPLDAS---ESSTVSDPSPAPE
AT5G60570 F-box/kelch-repeat protein                         (14)  SASASASASASVVVNLRVG------------------------------------EEDNDGHRL
Consensus                                                    (65)      V DL        RL       E      DS                         SD
```

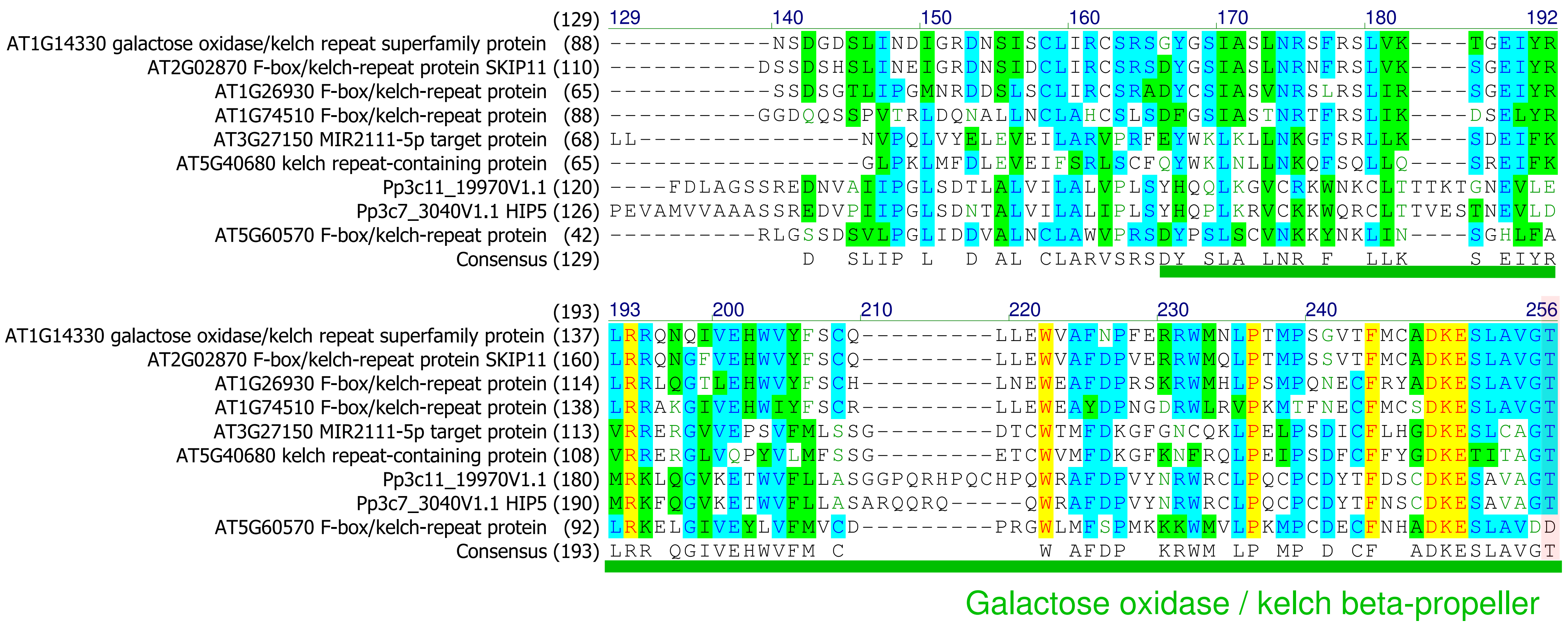

(129) 129 140 150 160 170 180 192
AT1G14330 galactose oxidase/kelch repeat superfamily protein (88) -----------NSDGDSLINDIGRDNSISCLIRCSRSGYGSIASLNRSFRSLVK----TGEIYR
AT2G02870 F-box/kelch-repeat protein SKIP11 (110) ----------DSSDSHSLINEIGRDNSIDCLIRCSRSDYGSIASLNRNFRSLVK----SGEIYR
AT1G26930 F-box/kelch-repeat protein (65) -----------SSDSGTLIPGMNRDDSLSCLIRCSRADYCSIASVNRSLRSLIR----SGEIYR
AT1G74510 F-box/kelch-repeat protein (88) ----------GGDQQSSPVTRLDQNALLNCLAHCSLSDFGSIASTNRTFRSLIK----DSELYR
AT3G27150 MIR2111-5p target protein (68) LL---------------NVPQLVYELEVEILARVPRFEYWKLKLLNKGFSRLLK----SDEIFK
AT5G40680 kelch repeat-containing protein (65) -----------------GLPKLMFDLEVEIFSRLSCFQYWKLNLLNKQFSQLLQ----SREIFK
Pp3c11_19970V1.1 (120) ----FDLAGSSREDNVAIIPGLSDTLALVILALVPLSYHQQLKGVCRKWNKCLTTTKTGNEVLE
Pp3c7_3040V1.1 HIP5 (126) PEVAMVVAAASSREDVPIIPGLSDNTALVILALIPLSYHQPLKRVCKKWQRCLTTVESTNEVLD
AT5G60570 F-box/kelch-repeat protein (42) ----------RLGSSDSVLPGLIDDVALNCLAWVPRSDYPSLSCVNKKYNKLIN----SGHLFA
Consensus (129) D SLIP L D AL CLARVSRSDY SLA LNR F LLK S EIYR
(193) 193 200 210 220 230 240 256
AT1G14330 galactose oxidase/kelch repeat superfamily protein (137) LRRQNQIVEHWVYFSCQ---------LLEWVAFNPFERRWMNLPTMPSGVTFMCADKESLAVGT
AT2G02870 F-box/kelch-repeat protein SKIP11 (160) LRRQNGFVEHWVYFSCQ---------LLEWVAFDPVERRWMQLPTMPSSVTFMCADKESLAVGT
AT1G26930 F-box/kelch-repeat protein (114) LRRLQGTLEHWVYFSCH---------LNEWEAFDPRSKRWMHLPSMPQNECFRYADKESLAVGT
AT1G74510 F-box/kelch-repeat protein (138) LRRAKGIVEHWIYFSCR---------LLEWEAYDPNGDRWLRVPKMTFNECFMCSDKESLAVGT
AT3G27150 MIR2111-5p target protein (113) VRRERGVVEPSVFMLSSG--------DTCWTMFDKGFGNCQKLPELPSDICFLHGDKESLCAGT
AT5G40680 kelch repeat-containing protein (108) VRRERGLVQPYVLMFSSG--------ETCWVMFDKGFKNFRQLPEIPSDFCFFYGDKETITAGT
Pp3c11_19970V1.1 (180) MRKLQGVKETWVFLLASGGPQRHPQCHPQWRAFDPVYNRWRCLPQCPCDYTFDSCDKESAVAGT
Pp3c7_3040V1.1 HIP5 (190) MRKFQGVKETWVFLLASARQQRQ-----QWRAFDPVYNRWRCLPQCPCDYTFNSCDKESAVAGT
AT5G60570 F-box/kelch-repeat protein (92) LRKELGIVEYLVFMVCD---------PRGWLMFSPMKKKWMVLPKMPCDECFNHADKESLAVDD
Consensus (193) LRR QGIVEHWVFM C W AFDP KRWM LP MP D CF ADKESLAVGT
Galactose oxidase / kelch beta-propeller

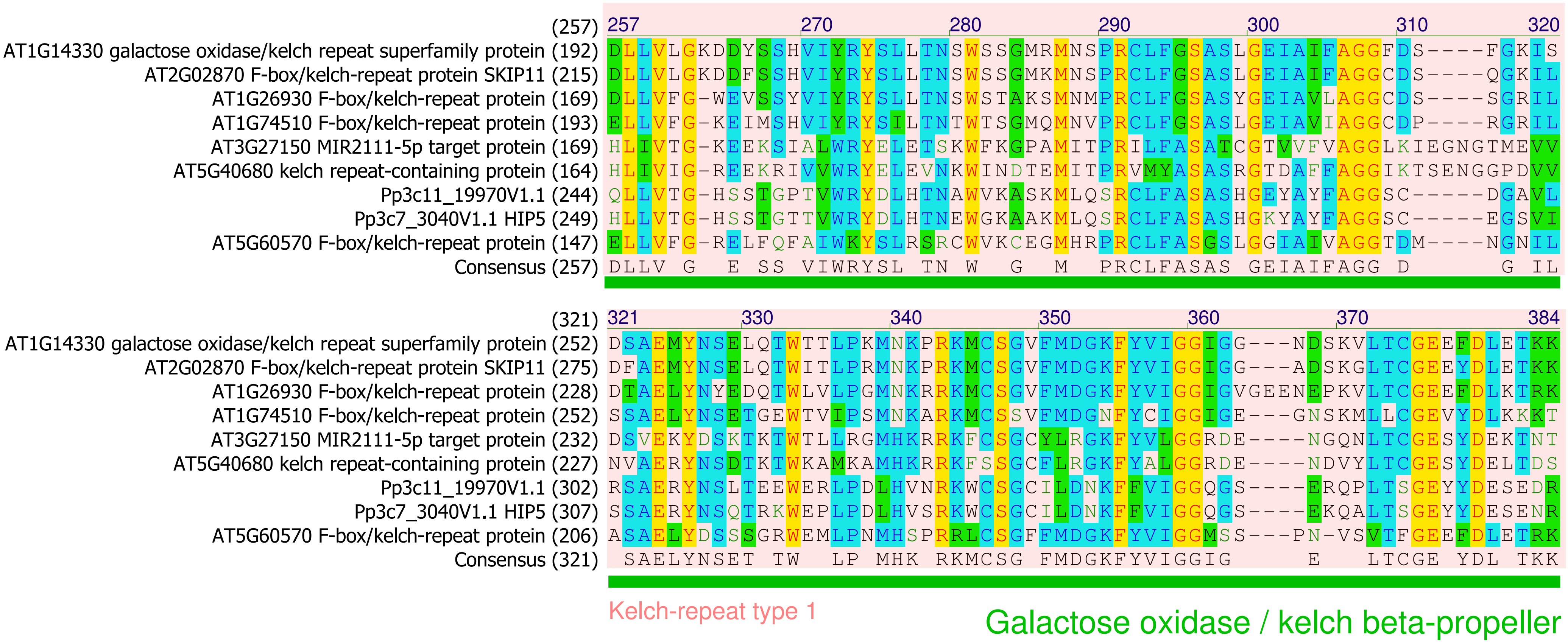
(257) 257 270 280 290 300 310 320
AT1G14330 galactose oxidase/kelch repeat superfamily protein (192) DLLVLGKDDYSSHVIYRYSLLTNSWSSGMRMNSPRCLFGSASLGEIAIFAGGFDS----FGKIS
AT2G02870 F-box/kelch-repeat protein SKIP11 (215) DLLVLGKDDFSSHVIYRYSLLTNSWSSGMKMNSPRCLFGSASLGEIAIFAGGCDS----QGKIL
AT1G26930 F-box/kelch-repeat protein (169) DLLVFG-WEVSSYVIYRYSLLTNSWSTAKSMNMPRCLFGSASYGEIAVLAGGCDS----SGRIL
AT1G74510 F-box/kelch-repeat protein (193) ELLVFG-KEIMSHVIYRYSILTNTWTSGMQMNVPRCLFGSASLGEIAVIAGGCDP----RGRIL
AT3G27150 MIR2111-5p target protein (169) HLIVTG-KEEKSIALWRYELETSKWFKGPAMITPRILFASATCGTVVFVAGGLKIEGNGTMEVV
AT5G40680 kelch repeat-containing protein (164) HLIVIG-REEKRIVVWRYELEVNKWINDTEMITPRVMYASASRGTDAFFAGGIKTSENGGPDVV
Pp3c11_19970V1.1 (244) QLLVTG-HSSTGPTVWRYDLHTNAWVKASKMLQSRCLFASASHGEYAYFAGGSC-----DGAVL
Pp3c7_3040V1.1 HIP5 (249) HLLVTG-HSSTGTTVWRYDLHTNEWGKAAKMLQSRCLFASASHGKYAYFAGGSC-----EGSVI
AT5G60570 F-box/kelch-repeat protein (147) ELLVFG-RELFQFAIWKYSLRSRCWVKCEGMHRPRCLFASGSLGGIAIVAGGTDM----NGNIL
Consensus (257) DLLV G E SS VIWRYSL TN W G M PRCLFASAS GEIAIFAGG D G IL
(321) 321 330 340 350 360 370 384
AT1G14330 galactose oxidase/kelch repeat superfamily protein (252) DSAEMYNSELQTWTTLPKMNKPRKMCSGVFMDGKFYVIGGIGG---NDSKVLTCGEEFDLETKK
AT2G02870 F-box/kelch-repeat protein SKIP11 (275) DFAEMYNSELQTWITLPRMNKPRKMCSGVFMDGKFYVIGGIGG---ADSKGLTCGEEYDLETKK
AT1G26930 F-box/kelch-repeat protein (228) DTAELYNYEDQTWLVLPGMNKRRKMCSGVFMDGKFYVIGGIGVGEENEPKVLTCGEEFDLKTRK
AT1G74510 F-box/kelch-repeat protein (252) SSAELYNSETGEWTVIPSMNKARKMCSSVFMDGNFYCIGGIGE---GNSKMLLCGEVYDLKKKT
AT3G27150 MIR2111-5p target protein (232) DSVEKYDSKTKTWTLLRGMHKRRKFCSGCYLRGKFYVLGGRDE----NGQNLTCGESYDEKTNT
AT5G40680 kelch repeat-containing protein (227) NVAERYNSDTKTWKAMKAMHKRRKFSSGCFLRGKFYALGGRDE----NDVYLTCGESYDELTDS
Pp3c11_19970V1.1 (302) RSAERYNSLTEEWERLPDLHVNRKWCSGCILDNKFFVIGGQGS----ERQPLTSGEYYDESEDR
Pp3c7_3040V1.1 HIP5 (307) SSAERYNSQTRKWEPLPDLHVSRKWCSGCILDNKFFVIGGQGS----EKQALTSGEYYDESENR
AT5G60570 F-box/kelch-repeat protein (206) ASAELYDSSSGRWEMLPNMHSPRRLCSGFFMDGKFYVIGGMSS---PN-VSVTFGEEFDLETRK
Consensus (321) SAELYNSET TW LP MHK RKMCSG FMDGKFYVIGGIG E LTCGE YDL TKK
Kelch-repeat type 1
Galactose oxidase / kelch beta-propeller

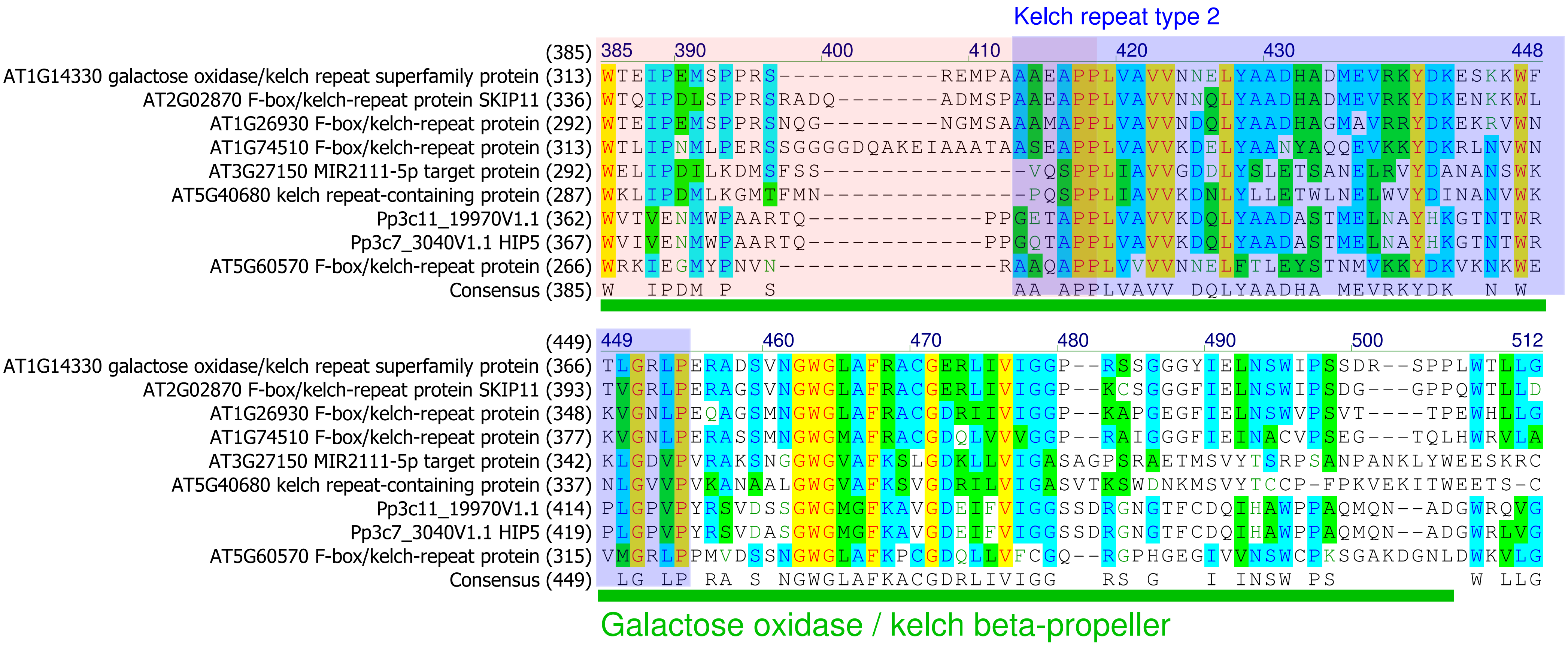

Kelch repeat type 2
(385) 385 390 400 410 420 430 448
AT1G14330 galactose oxidase/kelch repeat superfamily protein (313) WTEIPEMSPPRS-----------REMPAAAEAPPLVAVVNNELYAADHADMEVRKYDKESKKWF
AT2G02870 F-box/kelch-repeat protein SKIP11 (336) WTQIPDLSPPRSRADQ-------ADMSPAAEAPPLVAVVNNQLYAADHADMEVRKYDKENKKWL
AT1G26930 F-box/kelch-repeat protein (292) WTEIPEMSPPRSNQG--------NGMSAAAMAPPLVAVVNDQLYAADHAGMAVRRYDKEKRVWN
AT1G74510 F-box/kelch-repeat protein (313) WTLIPNMLPERSSGGGGDQAKEIAAATAASEAPPLVAVVKDELYAANYAQQEVKKYDKRLNVWN
AT3G27150 MIR2111-5p target protein (292) WELIPDILKDMSFSS--------------VQSPPLIAVVGDDLYSLETSANELRVYDANANSWK
AT5G40680 kelch repeat-containing protein (287) WKLIPDMLKGMTFMN--------------PQSPPLIAVVKDNLYLLETWLNELWVYDINANVWK
Pp3c11_19970V1.1 (362) WVTVENMWPAARTQ------------PPGETAPPLVAVVKDQLYAADASTMELNAYHKGTNTWR
Pp3c7_3040V1.1 HIP5 (367) WVIVENMWPAARTQ------------PPGQTAPPLVAVVKDQLYAADASTMELNAYHKGTNTWR
AT5G60570 F-box/kelch-repeat protein (266) WRKIEGMYPNVN---------------RAAQAPPLVVVVNNELFTLEYSTNMVKKYDKVKNKWE
Consensus (385) W IPDM P S AA APPLVAVV DQLYAADHA MEVRKYDK N W
(449) 449 460 470 480 490 500 512
AT1G14330 galactose oxidase/kelch repeat superfamily protein (366) TLGRLPERADSVNGWGLAFRACGERLIVIGGP--RSSGGGYIELNSWIPSSDR--SPPLWTLLG
AT2G02870 F-box/kelch-repeat protein SKIP11 (393) TVGRLPERAGSVNGWGLAFRACGERLIVIGGP--KCSGGGFIELNSWIPSDG---GPPQWTLLD
AT1G26930 F-box/kelch-repeat protein (348) KVGNLPEQAGSMNGWGLAFRACGDRIIVIGGP--KAPGEGFIELNSWVPSVT----TPEWHLLG
AT1G74510 F-box/kelch-repeat protein (377) KVGNLPERASSMNGWGMAFRACGDQLVVVGGP--RAIGGGFIEINACVPSEG---TQLHWRVLA
AT3G27150 MIR2111-5p target protein (342) KLGDVPVRAKSNGGWGVAFKSLGDKLLVIGASAGPSRAETMSVYTSRPSANPANKLYWEESKRC
AT5G40680 kelch repeat-containing protein (337) NLGVVPVKANAALGWGVAFKSVGDRILVIGASVTKSWDNKMSVYTCCP-FPKVEKITWEETS-C
Pp3c11_19970V1.1 (414) PLGPVPYRSVDSSGWGMGFKAVGDEIFVIGGSSDRGNGTFCDQIHAWPPAQMQN--ADGWRQVG
Pp3c7_3040V1.1 HIP5 (419) PLGPVPYRSVDASGWGMGFKAVGDEIFVIGGSSDRGNGTFCDQIHAWPPAQMQN--ADGWRLVG
AT5G60570 F-box/kelch-repeat protein (315) VMGRLPPMVDSSNGWGLAFKPCGDQLLVFCGQ--RGPHGEGIVVNSWCPKSGAKDGNLDWKVLG
Consensus (449) LG LP RA S NGWGLAFKACGDRLIVIGG RS G I INSW PS W LLG
Galactose oxidase / kelch beta-propeller

Sequence	513 … 520 … 530
(513)	
AT1G14330 galactose oxidase/kelch repeat superfamily protein (426)	R K H S S - N F V Y N C A V M G C -
AT2G02870 F-box/kelch-repeat protein SKIP11 (452)	R K H S P - T F V Y N C A V M G C -
AT1G26930 F-box/kelch-repeat protein (406)	K K Q S V - N F V Y N C A V M S C -
AT1G74510 F-box/kelch-repeat protein (436)	S K P S G - N F V Y N C A V M G C -
AT3G27150 MIR2111-5p target protein (406)	C G V R F N H F I L N C C V M I A -
AT5G40680 kelch repeat-containing protein (399)	D C V Q L G H F I R N C C V M L A -
Pp3c11_19970V1.1 (476)	Q L S N T S G F I Y N C A V M I V -
Pp3c7_3040V1.1 HIP5 (481)	Q L P N T S G F I Y N C A V M I V -
AT5G60570 F-box/kelch-repeat protein (377)	V K E N V G V F V Y N C A V M G C -
Consensus (513)	K F V Y N C A V M C
