# Supplementary material for: Holophytochrome-Interacting Proteins in Physcomitrella: Putative Actors in Phytochrome Cytoplasmic Signaling
Source: Front Plant Sci. 2016 May 12;7:613. doi: 10.3389/fpls.2016.00613 (PMC4867686; doi:10.3389/fpls.2016.00613)
Supplement: Supplementary file 2 [file Data_Sheet_2.ZIP › SI/SI HIP6.pdf]

frontiers

Supplementary Material

Holophytochrome-interacting proteins in *Physcomitrella*: putative actors in phytochrome cytoplasmic signaling

Anna Lena Ermert, Katharina Mailliet, and Jon Hughes*

*** Correspondence:** jon.hughes@uni-giessen.de

HIP6 (Pp3c6_4480V1.1)

ATGGTGGAAAAGAATGCATCTAGGGATGGCGGTACTCCTGCGAGAAATGGACATAATCAGCAGATGGCGGGGAATCAAGTA
GACGCAAGACATGGAGGATGTAACTTCTTTGCTGGTAATATGAAGGACCTTAGCAGCCTGAAGGAATGGAGAAAGTTATCA
ACTGCATGGAGGCTGTTGGTCTTGGTAGTCATTGGAGCTATTGGTTTATACACGTGTATGGTGGGCGTGGGCACAAGACAA
TTCTCATACGAGTCTGTGCAAGAAGTGATCGTGAAGGATTGGAGACGAGAAGATGATTCTTGTTCTCGACGTGGTCCTTCT
GTAGATTTCTACCAGCACTATCCACTTCCGCATGCATATGAGAGGCAAGAGTGCACTTGCACTCCTGTGCACTATTTTGTA
ATATTATCAATGCAAAGATCAGGAAGTGGATGGTTTGAGACTCTACTCAATAATCACCCAAATATTAGCTCCCATGGCGAG
ATCTTTTCCGTCAAGGATCGGAGAGAAAATTTTTCCTCTATCGCAAAAACCATGGATAAAGTCTACAACTTAGATTGGCTG
AATAGTGCTGCTAAGAATGAATGTACTGCAGCTGTTGGTTTCAAGTGGATGCTCAACCAGGGACCGATGGAATACAACAGG
GAGGTCAGGGAATACTTTGAGAGAATGGGTGTTTCTGTGATTCTTTTACTTCGACGGAACGTGTTGAAGCGGCTAATTTCA
ATCTTAGCAAATGCATACGATCGTGTAGTAAAGCCTCTGAATGGCACTCATAAGTCGCATGTTCACTCTGTTGAAGAGGCT
ATGAAACTGGCAGAATACAGGCCAGTCGTAGATGCGAAGCATTTGTTTGAAAACTTGGCGCGAGTTGAGCAGATAACTGAT
GACGCCCAACGGTTCTTCAATAACACTCGGATCAGAGTTGTTCACTACGAAGATCTTGTGGTGGATCCTAAGTATCTGTCC
GAAATCCAACATTTTTTGGGGGTGCAACCCCGGAAGCTAGAAAGCCAGCAAGTTAAGATCCATACACGACCACTGAGAGAA
CAAATCCAAAACTGGGATGAGGTACTGATTCATCTGAAAGGGACAAAGTATGAGAGTCTTCTTCAGGATGACGGATACCCG
TGA

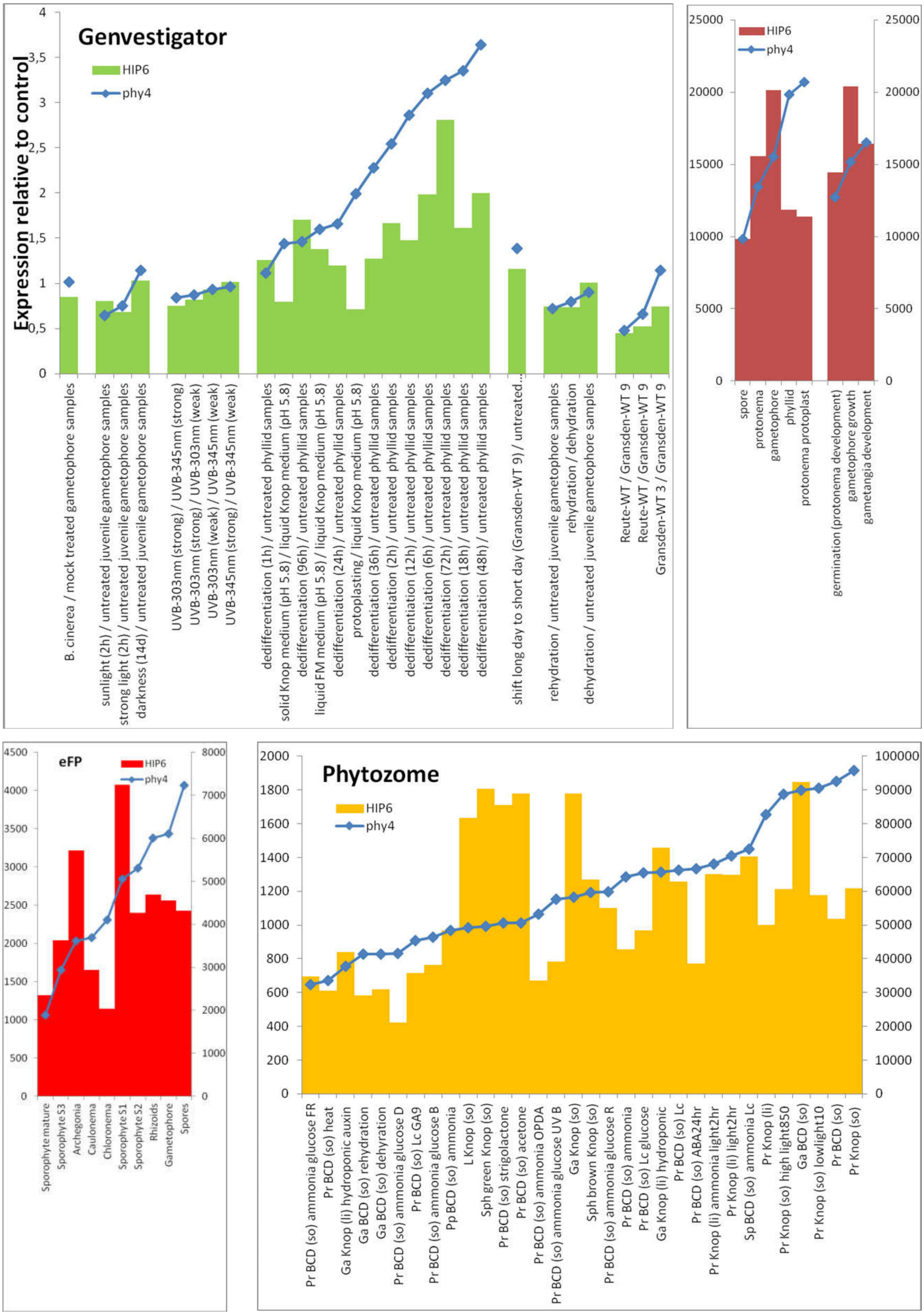

Genvestigator
HIP6
phy4
Expression relative to control
B. cinerea / mock treated gametophore samples
sunlight (2h) / untreated juvenile gametophore samples
strong light (2h) / untreated juvenile gametophore samples
darkness (14d) / untreated juvenile gametophore samples
UVB-303nm (strong) / UVB-345nm (strong)
UVB-303nm (strong) / UVB-303nm (weak)
UVB-303nm (weak) / UVB-345nm (weak)
UVB-345nm (strong) / UVB-345nm (weak)
dedifferentiation (1h) / untreated phyllid samples
solid Knop medium (pH 5.8) / liquid Knop medium (pH 5.8)
dedifferentiation (96h) / untreated phyllid samples
liquid FM medium (pH 5.8) / liquid Knop medium (pH 5.8)
dedifferentiation (24h) / untreated phyllid samples
protoplasting / liquid Knop medium (pH 5.8)
dedifferentiation (36h) / untreated phyllid samples
dedifferentiation (2h) / untreated phyllid samples
dedifferentiation (12h) / untreated phyllid samples
dedifferentiation (6h) / untreated phyllid samples
dedifferentiation (72h) / untreated phyllid samples
dedifferentiation (18h) / untreated phyllid samples
dedifferentiation (48h) / untreated phyllid samples
shift long day to short day (Gransden-WT 9) / untreated...
rehydration / untreated juvenile gametophore samples
rehydration / dehydration
dehydration / untreated juvenile gametophore samples
Reute-WT / Gransden-WT 9
Reute-WT / Gransden-WT 9
Gransden-WT 3 / Gransden-WT 9
HIP6
phy4
spore
protonema
gametophore
phyllid
protonema protoplast
germination (protonema development)
gametophore growth
gametangia development
eFP
HIP6
phy4
Sporophyte mature
Sporophyte S3
Archegonia
Caulonema
Chloronema
Sporophyte S1
Sporophyte S2
Rhizoids
Gametophore
Spores
Phytozome
HIP6
phy4
Pr BCD (so) ammonia glucose FR
Pr BCD (so) heat
Ga Knop (li) hydroponic auxin
Ga BCD (so) rehydration
Ga BCD (so) dehyration
Pr BCD (so) ammonia glucose D
Pr BCD (so) Lc GA9
Pr BCD (so) ammonia glucose B
Pp BCD (so) ammonia
L Knop (so)
Sph green Knop (so)
Pr BCD (so) strigolactone
Pr BCD (so) acetone
Pr BCD (so) ammonia OPDA
Pr BCD (so) ammonia glucose UV B
Ga Knop (so)
Sph brown Knop (so)
Pr BCD (so) ammonia glucose R
Pr BCD (so) ammonia
Pr BCD (so) Lc glucose
Ga Knop (li) hydroponic
Pr BCD (so) Lc
Pr BCD (so) ABA24hr
Pr Knop (li) ammonia light2hr
Pr Knop (li) light2hr
Sp BCD (so) ammonia Lc
Pr Knop (li)
Pr Knop (so) high light850
Ga BCD (so)
Pr Knop (so) lowlight10
Pr BCD (so)
Pr Knop (so)

HIP6 alignment tree

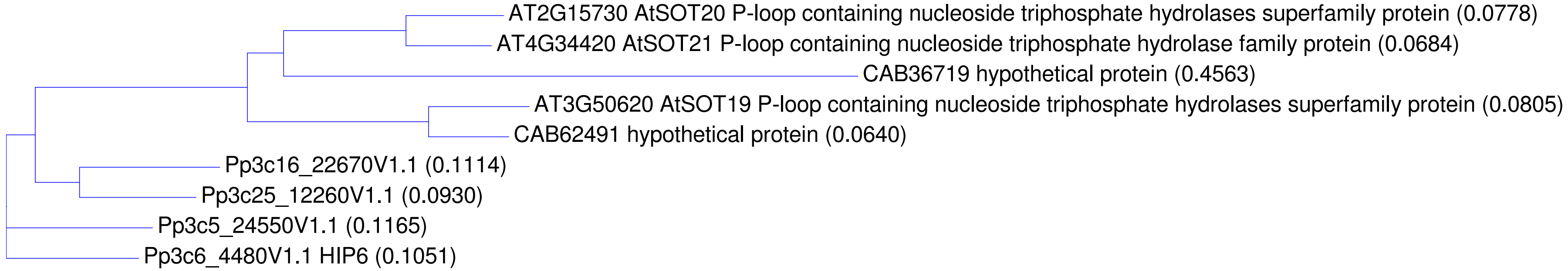

HIP6 alignment

```
                               (1)  1        10        20        30        40        50           64
AT2G15730 AtSOT20              (1)  -----------------------------------------MGDEHSLIPKDSFVFKLPKKSPL
AT3G50620 AtSOT19              (1)  ---------------------------------------MAEYICLFGKDSAAIVIKQPKKSPL
AT4G34420 AtSOT21              (1)  -----------------------------------------MAEDPSFLPKDGFVLKLPKKSSL
CAB36719 hypothetical protein  (1)  -------------------------------------------------------MKQHQYHGS
CAB62491 hypothetical protein  (1)  -----------------------------------------------------MSASKPPNLSK
Pp3c16_22670V1.1               (1)  -----------------------------MAASRKDQNSEVGCDIHVAALQDSCSLKGWRKATV
Pp3c25_12260V1.1               (1)  MVDKKTCLPLHFSPSLRPGGAPATNGYHQTAGSHVDTKLEGRYDFYAANMKDIWNAKEWRKVST
Pp3c5_24550V1.1                (1)  MVEKK---------GCRDGVPSAGNGFHQMTGIQSDSKYEGGHECFASTMKDLCSGKEWRKIST
Pp3c6_4480V1.1 HIP6            (1)  MVEKN---------ASRDGGTPARNGHNQQMAGNQVDARHGGCNFFAGNMKDLSSLKEWRKLST
Consensus                      (1)                                             D  A   KD   LK  RK S

                               (65) 65   70        80        90        100       110             128
AT2G15730 AtSOT20              (24) VLRTVVLLFVMVCTVYICSICLKQIGVVPSAGFLNVEVFERPCPE---PNIQPWDIPYVHYPKP
AT3G50620 AtSOT19              (26) FLRMIVLVFAMVCGLYICAVCLKQLSNVSFQTSQLVQTSPIDSHSLRFVTR-------IHYPKP
AT4G34420 AtSOT21              (24) VLRMVVLLFVMVCAVYICSICLKQIGVSPNYGFLNVEVFERPCPE---PNIEPWDIPFVHYPKP
CAB36719 hypothetical protein  (10) YAEPRISFSSGFAATKHEMIKYKEAPVSSDDFEFGVEN--------------------------
CAB62491 hypothetical protein  (12) LVLLILTRYALLLGFII---------------QSLRL---------------------------
Pp3c16_22670V1.1               (36) VWKLLVLVVIGGFGLYACMVGVEDRRFSYETVEETTVVKDWRREDNSCSRHG-SEGFRRHYPLP
Pp3c25_12260V1.1               (65) VWRLLVLVVVGGFGVYMCMVGLDRRQFSYEPVEE-LVLKDVRREDDSCPRR-DHLDFYQHYPLP
Pp3c5_24550V1.1                (56) VWRLLVLVVIGAVSLYMCMVGVGTRQFPFESVEG-AVVKDWRREDESCAPRGPSVDYYQHYPLP
Pp3c6_4480V1.1 HIP6            (56) AWRLLVLVVIGAIGLYTCMVGVGTRQFSYESVQE-VIVKDWRREDDSCSRRGPSVDFYQHYPLP
Consensus                      (65) V RLLVLV IG  GLYICMV L     S E     VVV D    D            Y HYP P
```

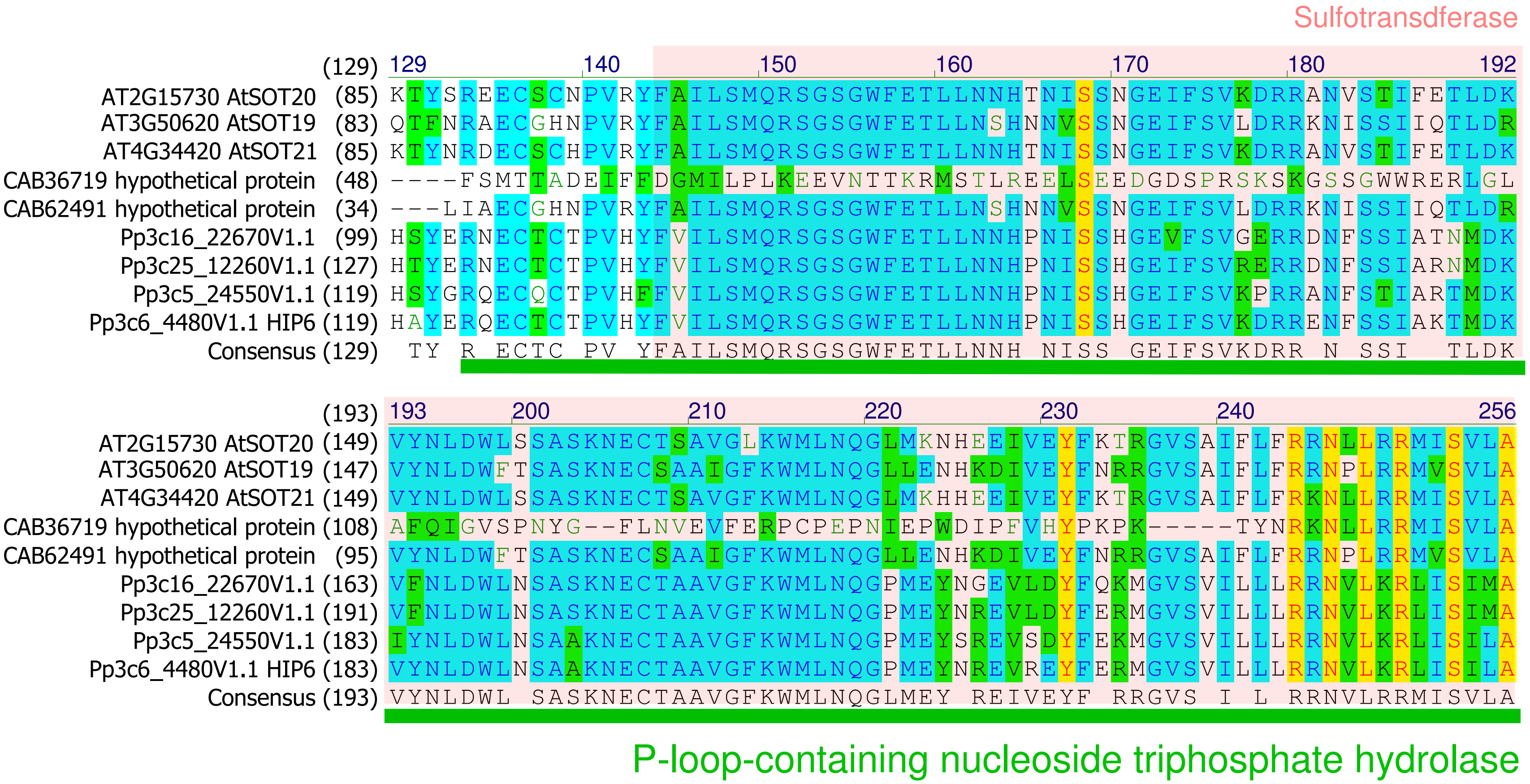
Sulfotransdferase
(129) 129 140 150 160 170 180 192
AT2G15730 AtSOT20 (85) KTYSREECSCNPVRYFAILSMQRSGSGWFETLLNNHTNISSNGEIFSVKDRRANVSTIFETLDK
AT3G50620 AtSOT19 (83) QTFNRAECGHNPVRYFAILSMQRSGSGWFETLLNSHNNVSSNGEIFSVLDRRKNISSIIQTLDR
AT4G34420 AtSOT21 (85) KTYNRDECSCHPVRYFAILSMQRSGSGWFETLLNNHTNISSNGEIFSVKDRRANVSTIFETLDK
CAB36719 hypothetical protein (48) ----FSMTTADEIFFDGMILPLKEEVNTTKRMSTLREELSEEDGDSPRSKSKGSSGWWRERLGL
CAB62491 hypothetical protein (34) ---LIAECGHNPVRYFAILSMQRSGSGWFETLLNSHNNVSSNGEIFSVLDRRKNISSIIQTLDR
Pp3c16_22670V1.1 (99) HSYERNECTCTPVHYFVILSMQRSGSGWFETLLNNHPNISSHGEVFSVGERRDNFSSIATNMDK
Pp3c25_12260V1.1 (127) HTYERNECTCTPVHYFVILSMQRSGSGWFETLLNNHPNISSHGEIFSVRERRDNFSSIARNMDK
Pp3c5_24550V1.1 (119) HSYGRQECQCTPVHFFVILSMQRSGSGWFETLLNNHPNISSHGEIFSVKPRRANFSTIARTMDK
Pp3c6_4480V1.1 HIP6 (119) HAYERQECTCTPVHYFVILSMQRSGSGWFETLLNNHPNISSHGEIFSVKDRRENFSSIAKTMDK
Consensus (129) TY R ECTC PV YFAILSMQRSGSGWFETLLNNH NISS GEIFSVKDRR N SSI TLDK
(193) 193 200 210 220 230 240 256
AT2G15730 AtSOT20 (149) VYNLDWLSSASKNECTSAVGLKWMLNQGLMKNHEEIVEYFKTRGVSAIFLFRRNLLRRMISVLA
AT3G50620 AtSOT19 (147) VYNLDWFTSASKNECSAAIGFKWMLNQGLLENHKDIVEYFNRRGVSAIFLFRRNPLRRMVSVLA
AT4G34420 AtSOT21 (149) VYNLDWLSSASKNECTSAVGFKWMLNQGLMKHHEEIVEYFKTRGVSAIFLFRKNLLRRMISVLA
CAB36719 hypothetical protein (108) AFQIGVSPNYG--FLNVEVFERPCPEPNIEPWDIPFVHYPKPK-----TYNRKNLLRRMISVLA
CAB62491 hypothetical protein (95) VYNLDWFTSASKNECSAAIGFKWMLNQGLLENHKDIVEYFNRRGVSAIFLFRRNPLRRMVSVLA
Pp3c16_22670V1.1 (163) VFNLDWLNSASKNECTAAVGFKWMLNQGPMEYNGEVLDYFQKMGVSVILLLRRNVLKRLISIMA
Pp3c25_12260V1.1 (191) VFNLDWLNSASKNECTAAVGFKWMLNQGPMEYNREVLDYFERMGVSVILLLRRNVLKRLISIMA
Pp3c5_24550V1.1 (183) IYNLDWLNSAAKNECTAAVGFKWMLNQGPMEYSREVSDYFEKMGVSVILLLRRNVLKRLISILA
Pp3c6_4480V1.1 HIP6 (183) VYNLDWLNSAAKNECTAAVGFKWMLNQGPMEYNREVREYFERMGVSVILLLRRNVLKRLISILA
Consensus (193) VYNLDWL SASKNECTAAVGFKWMLNQGLMEY REIVEYF RRGVS I L RRNVLRRMISVLA
P-loop-containing nucleoside triphosphate hydrolase

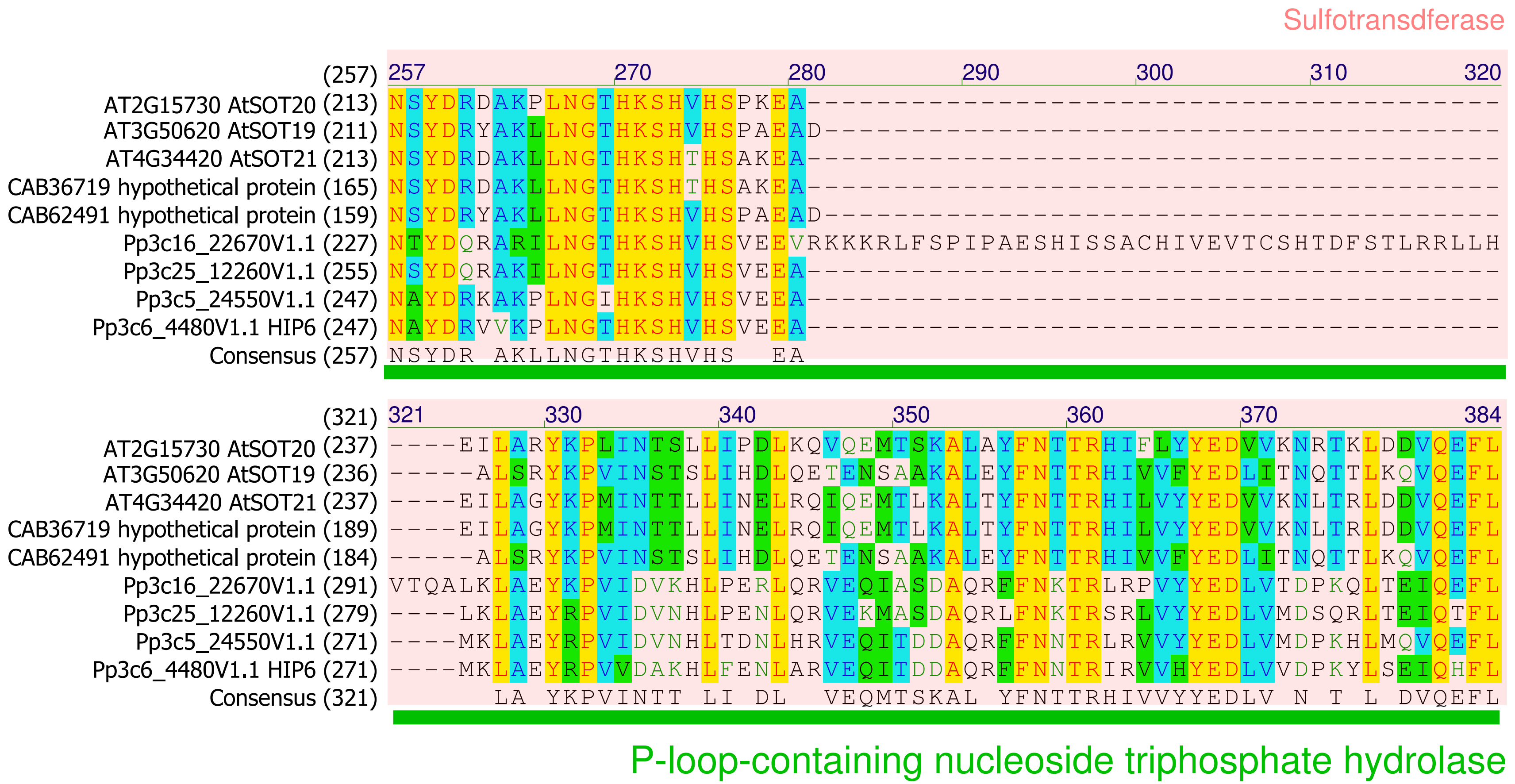
Sulfotransdferase
(257) 257 270 280 290 300 310 320
AT2G15730 AtSOT20 (213) NSYDRDAKPLNGTHKSHVHSPKEA--
AT3G50620 AtSOT19 (211) NSYDRYAKLLNGTHKSHVHSPAEAD---------------------------------------
AT4G34420 AtSOT21 (213) NSYDRDAKLLNGTHKSHTHSAKEA--
CAB36719 hypothetical protein (165) NSYDRDAKLLNGTHKSHTHSAKEA--
CAB62491 hypothetical protein (159) NSYDRYAKLLNGTHKSHVHSPAEAD---------------------------------------
Pp3c16_22670V1.1 (227) NTYDQRARILNGTHKSHVHSVEEVRKKKRLFSPIPAESHISSACHIVEVTCSHTDFSTLRRLLH
Pp3c25_12260V1.1 (255) NSYDQRAKILNGTHKSHVHSVEEA--
Pp3c5_24550V1.1 (247) NAYDRKAKPLNGIHKSHVHSVEEA--
Pp3c6_4480V1.1 HIP6 (247) NAYDRVVKPLNGTHKSHVHSVEEA--
Consensus (257) NSYDR AKLLNGTHKSHVHS EA
(321) 321 330 340 350 360 370 384
AT2G15730 AtSOT20 (237) ----EILARYKPLINTSLLIPDLKQVQEMTSKALAYFNTTRHIFLYYEDVVKNRTKLDDVQEFL
AT3G50620 AtSOT19 (236) -----ALSRYKPVINSTSLIHDLQETENSAAKALEYFNTTRHIVVFYEDLITNQTTLKQVQEFL
AT4G34420 AtSOT21 (237) ----EILAGYKPMINTTLLINELRQIQEMTLKALTYFNTTRHILVYYEDVVKNLTRLDDVQEFL
CAB36719 hypothetical protein (189) ----EILAGYKPMINTTLLINELRQIQEMTLKALTYFNTTRHILVYYEDVVKNLTRLDDVQEFL
CAB62491 hypothetical protein (184) -----ALSRYKPVINSTSLIHDLQETENSAAKALEYFNTTRHIVVFYEDLITNQTTLKQVQEFL
Pp3c16_22670V1.1 (291) VTQALKLAEYKPVIDVKHLPERLQRVEQIASDAQRFFNKTRLRPVYYEDLVTDPKQLTEIQEFL
Pp3c25_12260V1.1 (279) ----LKLAEYRPVIDVNHLPENLQRVEKMASDAQRLFNKTRSRLVYYEDLVMDSQRLTEIQTFL
Pp3c5_24550V1.1 (271) ----MKLAEYRPVIDVNHLTDNLHRVEQITDDAQRFFNNTRLRVVYYEDLVMDPKHLMQVQEFL
Pp3c6_4480V1.1 HIP6 (271) ----MKLAEYRPVVDAKHLFENLARVEQITDDAQRFFNNTRIRVVHYEDLVVDPKYLSEIQHFL
Consensus (321) LA YKPVINTT LI DL VEQMTSKAL YFNTTRHIVVYYEDLV N T L DVQEFL
P-loop-containing nucleoside triphosphate hydrolase

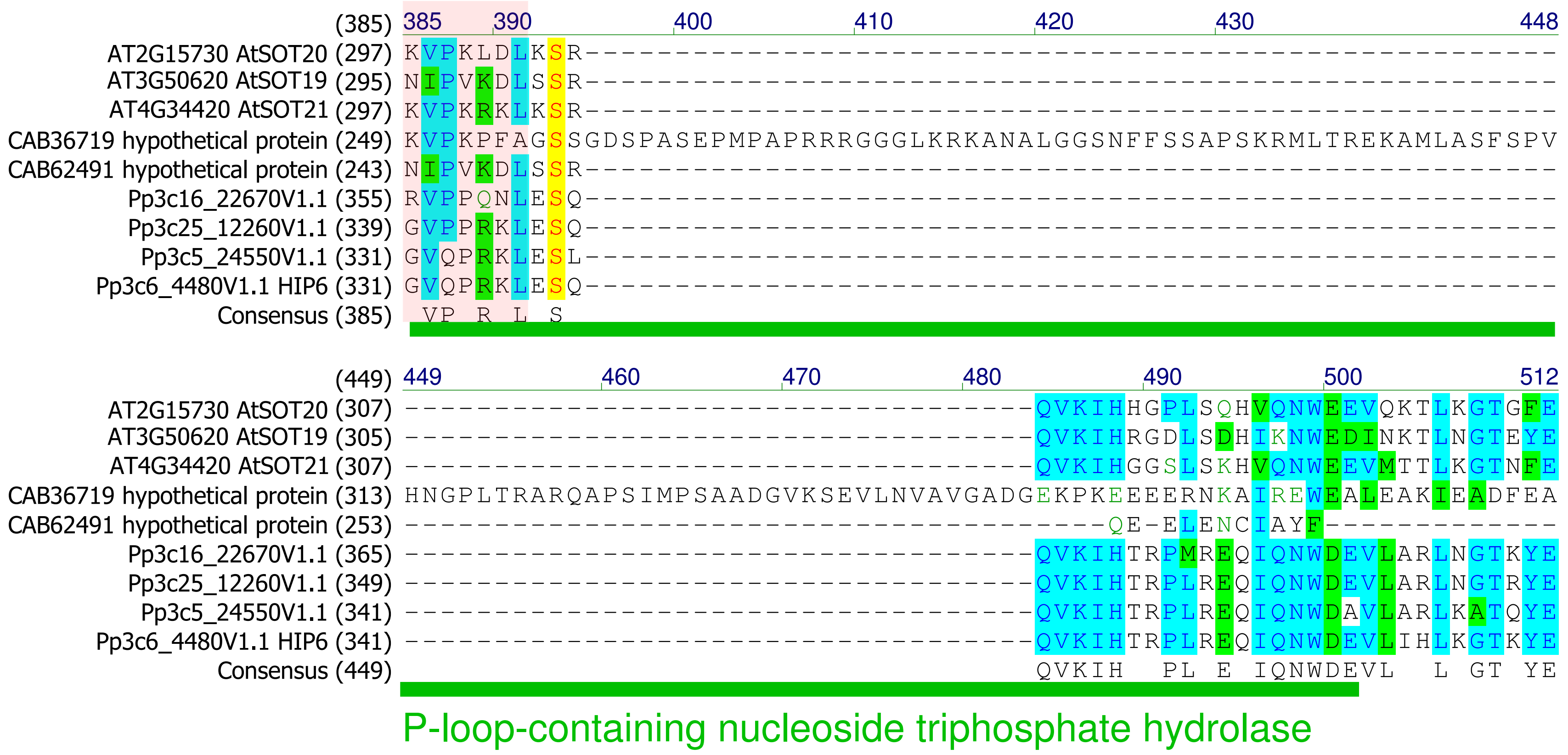
(385) 385 390 400 410 420 430 448
AT2G15730 AtSOT20 (297) KVPKLDLKSR
AT3G50620 AtSOT19 (295) NIPVKDLSSR
AT4G34420 AtSOT21 (297) KVPKRKLKSR
CAB36719 hypothetical protein (249) KVPKPFAGSSGDSPASEPMPAPRRRGGGLKRKANALGGSNFFSSAPSKRMLTREKAMLASFSPV
CAB62491 hypothetical protein (243) NIPVKDLSSR
Pp3c16_22670V1.1 (355) RVPPQNLESQ
Pp3c25_12260V1.1 (339) GVPPRKLESQ
Pp3c5_24550V1.1 (331) GVQPRKLESL
Pp3c6_4480V1.1 HIP6 (331) GVQPRKLESQ
Consensus (385) VP R L S
(449) 449 460 470 480 490 500 512
AT2G15730 AtSOT20 (307) QVKIHHGPLSQHVQNWEEVQKTLKGTGFE
AT3G50620 AtSOT19 (305) QVKIHRGDLSDHIKNWEDINKTLNGTEYE
AT4G34420 AtSOT21 (307) QVKIHGGSLSKHVQNWEEVMTTLKGTNFE
CAB36719 hypothetical protein (313) HNGPLTRARQAPSIMPSAADGVKSEVLNVAVGADGEKPKEEEERNKAIREWEALEAKIEADFEA
CAB62491 hypothetical protein (253) QE-ELENCIAYF
Pp3c16_22670V1.1 (365) QVKIHTRPMREQIQNWDEVLARLNGTKYE
Pp3c25_12260V1.1 (349) QVKIHTRPLREQIQNWDEVLARLNGTRYE
Pp3c5_24550V1.1 (341) QVKIHTRPLREQIQNWDAVLARLKATQYE
Pp3c6_4480V1.1 HIP6 (341) QVKIHTRPLREQIQNWDEVLIHLKGTKYE
Consensus (449) QVKIH PL E IQNWDEVL L GT YE
P-loop-containing nucleoside triphosphate hydrolase

```
                                  (513) 513    520                 539
           AT2G15730 AtSOT20 (336) NFLLEDYRR------------------
           AT3G50620 AtSOT19 (334) KFLRADY--------------------
           AT4G34420 AtSOT21 (336) NFLHQEF--------------------
 CAB36719 hypothetical protein (377) IRSRDSNVHVVPNHCGSFVLLLLPVFG
 CAB62491 hypothetical protein (264) ---------------------------
            Pp3c16_22670V1.1 (394) LLMHDDDYA------------------
            Pp3c25_12260V1.1 (378) LLMHDDDYT------------------
             Pp3c5_24550V1.1 (370) TLLNDGDST------------------
          Pp3c6_4480V1.1 HIP6 (370) SLLQDDGYP------------------
                   Consensus (513)   L DD
```
