# Supplementary material for: Holophytochrome-Interacting Proteins in Physcomitrella: Putative Actors in Phytochrome Cytoplasmic Signaling
Source: Front Plant Sci. 2016 May 12;7:613. doi: 10.3389/fpls.2016.00613 (PMC4867686; doi:10.3389/fpls.2016.00613)
Supplement: Supplementary file 2 [file Data_Sheet_2.ZIP › SI/SI HIP7.pdf]

Supplementary Material

Holophytochrome-interacting proteins in Physcomitrella: putative actors in phytochrome cytoplasmic signaling

Anna Lena Ermert, Katharina Mailliet, and Jon Hughes*

*** Correspondence:** jon.hughes@uni-giessen.de

HIP7 (Pp3c17_9390V1.1)

```
ATGTTAAGTACGAGGGCGTGCAGTAGCATCATGGGCTCGAAATCAGCTGAAACCAGTGGTGGGTCGAGTGGGGGGCGGCAA
CCTAGTCCTTTGGACCTGTACTCCAAAGGCACACAAATTATTGAGCTCGGATCCGCAGTGTGGCCGGAGAACCCCATTCGT
ACTGGTAGGTCTGAGGTGTACATATGGGGTTCTGAGCACGCGGTGAAGCTCTGCATGGGCCCGACTTTTGCCCTTCATGAA
TATACTATGAGCCGGGCAGCTAGGAACTATGCTGTGCGTACTGTAGCCATGTTCACAATTCATGGTAAACCAAATGGCATA
GTCATGGAACGGGGGAAGTCTGTGAACCCGGTGACGTGCGATTTAAAGCAGATTGCATTTGAGATGGTGCGAGCCGTTCAA
GGACTCTATAGCATAGGCATTATCCACGGGGATATAAAATTGTCTAGTTTCCTCGTTTGTCGTGATGGATGCGTTCGTCTG
TGTGACTTCGGAACTAGCGAGTACAAATGTGATAGTGTGAGCCACTCGGAAATGTCTATTCCATGGTCAAGGCCTTCATTG
TTGCGCAATCCTGACAGGCCGCGTGTCAAGGCGGACGATTTGTACTCGTTGGGGCTGACAATATGGGAATTGTATACGGGC
AAGGTTCCATTCGTACCACCTACATCTGAGGGATGGGAGTCACTGGATATAAACGAGGTAGCAGAAGAGGCGATACTGGCG
GGAGAGCAGGTGGACCTCAATGACATATTAGACCTTGAGATTCGCTGTGGTTGCTTCACTTGTGAGCGAGGAGGTCGAGCA
CCCACTCTGCCAGGTGTAGTAACTTGGGGCTTCCGAAAAGTAGCCAAATATTGA
```

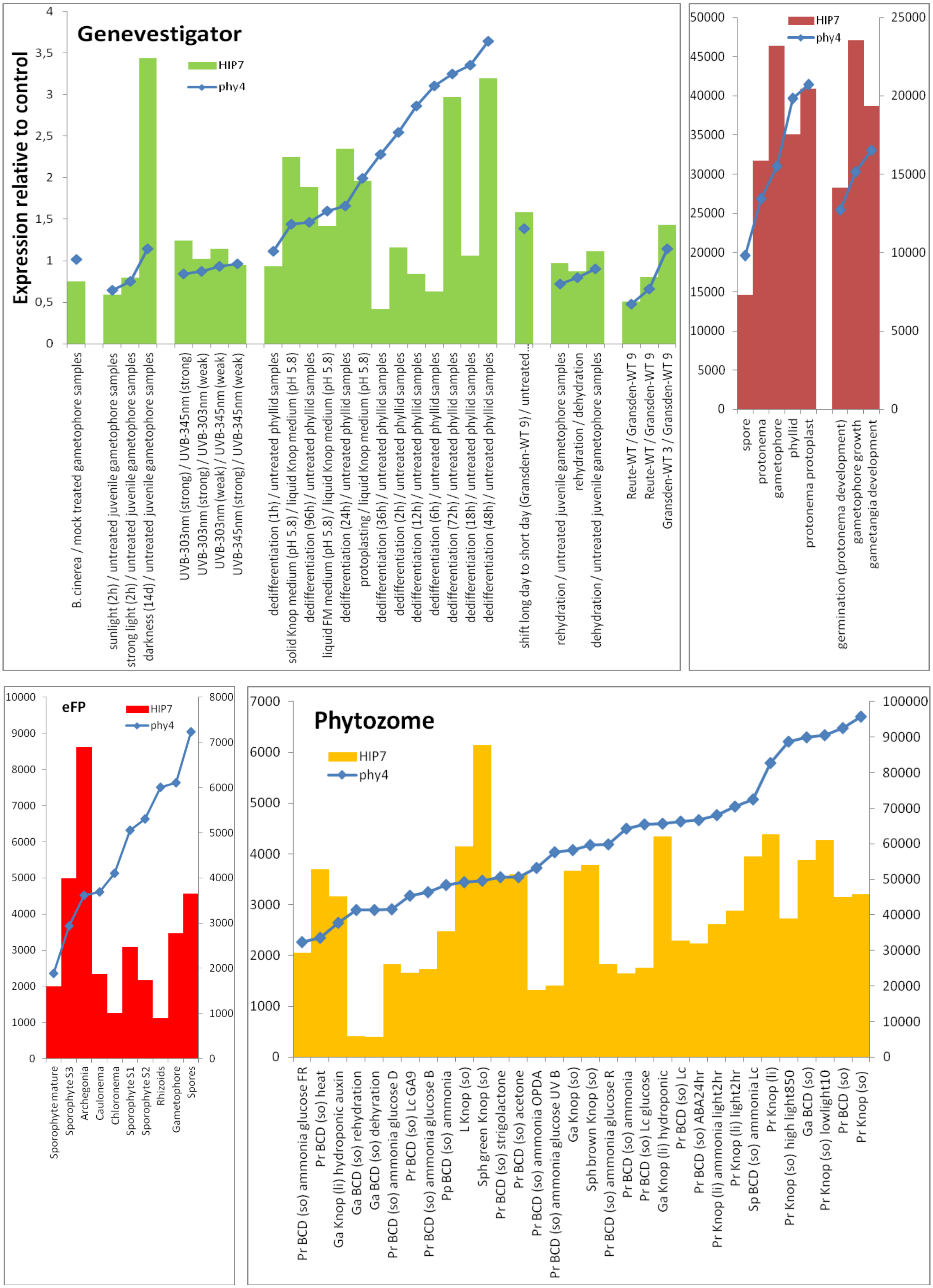
Genevestigator
Expression relative to control
HIP7
phy4
0
0,5
1
1,5
2
2,5
3
3,5
4
B. cinerea / mock treated gametophore samples
sunlight (2h) / untreated juvenile gametophore samples
strong light (2h) / untreated juvenile gametophore samples
darkness (14d) / untreated juvenile gametophore samples
UVB-303nm (strong) / UVB-345nm (strong)
UVB-303nm (strong) / UVB-303nm (weak)
UVB-303nm (weak) / UVB-345nm (weak)
UVB-345nm (strong) / UVB-345nm (weak)
dedifferentiation (1h) / untreated phyllid samples
solid Knop medium (pH 5.8) / liquid Knop medium (pH 5.8)
dedifferentiation (96h) / untreated phyllid samples
liquid FM medium (pH 5.8) / liquid Knop medium (pH 5.8)
dedifferentiation (24h) / untreated phyllid samples
protoplasting / liquid Knop medium (pH 5.8)
dedifferentiation (36h) / untreated phyllid samples
dedifferentiation (2h) / untreated phyllid samples
dedifferentiation (12h) / untreated phyllid samples
dedifferentiation (6h) / untreated phyllid samples
dedifferentiation (72h) / untreated phyllid samples
dedifferentiation (18h) / untreated phyllid samples
dedifferentiation (48h) / untreated phyllid samples
shift long day to short day (Gransden-WT 9) / untreated...
rehydration / untreated juvenile gametophore samples
rehydration / dehydration
dehydration / untreated juvenile gametophore samples
Reute-WT / Gransden-WT 9
Reute-WT / Gransden-WT 9
Gransden-WT 3 / Gransden-WT 9
HIP7
phy4
0
5000
10000
15000
20000
25000
30000
35000
40000
45000
50000
0
5000
10000
15000
20000
25000
spore
protonema
gametophore
phyllid
protonema protoplast
germination (protonema development)
gametophore growth
gametangia development
eFP
HIP7
phy4
0
1000
2000
3000
4000
5000
6000
7000
8000
9000
10000
0
1000
2000
3000
4000
5000
6000
7000
8000
Sporophyte mature
Sporophyte S3
Archegonia
Caulonema
Chloronema
Sporophyte S1
Sporophyte S2
Rhizoids
Gametophore
Spores
Phytozome
HIP7
phy4
0
1000
2000
3000
4000
5000
6000
7000
0
10000
20000
30000
40000
50000
60000
70000
80000
90000
100000
Pr BCD (so) ammonia glucose FR
Pr BCD (so) heat
Ga Knop (li) hydroponic auxin
Ga BCD (so) rehydration
Ga BCD (so) dehydration
Pr BCD (so) ammonia glucose D
Pr BCD (so) Lc GA9
Pr BCD (so) ammonia glucose B
Pp BCD (so) ammonia
L Knop (so)
Sph green Knop (so)
Pr BCD (so) strigolactone
Pr BCD (so) acetone
Pr BCD (so) ammonia OPDA
Pr BCD (so) ammonia glucose UV B
Ga Knop (so)
Sph brown Knop (so)
Pr BCD (so) ammonia glucose R
Pr BCD (so) ammonia
Pr BCD (so) Lc glucose
Ga Knop (li) hydroponic
Pr BCD (so) Lc
Pr BCD (so) ABA24hr
Pr Knop (li) ammonia light2hr
Pr Knop (li) light2hr
Sp BCD (so) ammonia Lc
Pr Knop (li)
Pr Knop (so) high light850
Ga BCD (so)
Pr Knop (so) lowlight10
Pr BCD (so)
Pr Knop (so)

HIP7 alignment tree

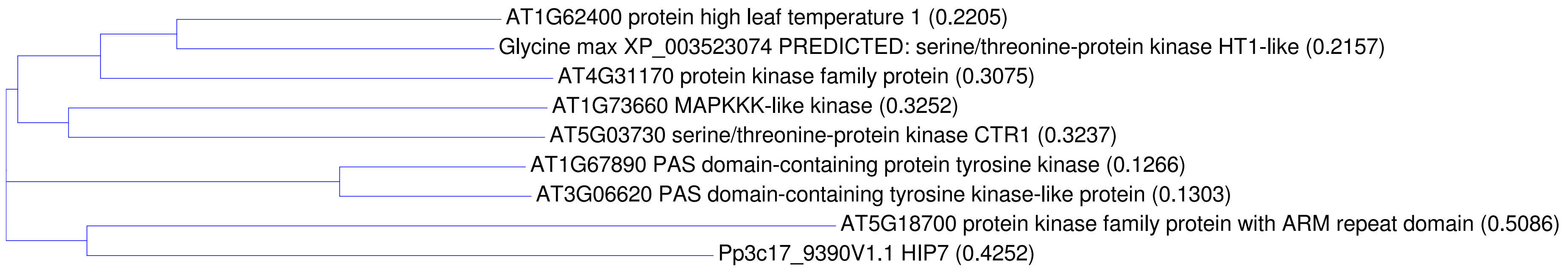

HIP7 alignment

Sequence	Start	1 … 10 … 20 … 30 … 40 … 50 … 62
AT1G62400 protein high leaf temperature 1	(1)	--
Glycine max XP_003523074 PREDICTED: HT1-like	(1)	--
AT4G31170 protein kinase family protein	(1)	--
AT1G73660 MAPKKK-like kinase	(1)	--
AT5G03730 serine/threonine-protein kinase CTR1	(1)	--
AT1G67890 PAS domain-containing protein tyrosine kinase	(1)	--
AT3G06620 PAS domain-containing tyrosine kinase-like	(1)	--
AT5G18700 protein kinase family protein with ARM repeat	(1)	MNQYHIYEAIGHGKCSTVYKGRKKKTIEYFACKSVDKSRKNKVLQEVRILHSLNHPNVLKFY
Pp3c17_9390V1.1 HIP7	(1)	--
Consensus	(1)	

Sequence	Start	63 … 70 … 80 … 90 … 100 … 110 … 124
AT1G62400 protein high leaf temperature 1	(1)	--
Glycine max XP_003523074 PREDICTED: HT1-like	(1)	--
AT4G31170 protein kinase family protein	(1)	--
AT1G73660 MAPKKK-like kinase	(1)	--
AT5G03730 serine/threonine-protein kinase CTR1	(1)	--
AT1G67890 PAS domain-containing protein tyrosine kinase	(1)	--
AT3G06620 PAS domain-containing tyrosine kinase-like	(1)	--
AT5G18700 protein kinase family protein with ARM repeat	(63)	AWYETSAHMWLVLEYCVGGDLRTLLQQDCKLPEESIYGLAYDLVIALQYLHSKGIIYCDLKP
Pp3c17_9390V1.1 HIP7	(1)	--
Consensus	(63)	

	(125)	125 130 140 150 160 170 186
AT1G62400 protein high leaf temperature 1	(1)	--
Glycine max XP_003523074 PREDICTED: HT1-like	(1)	--
AT4G31170 protein kinase family protein	(1)	--
AT1G73660 MAPKKK-like kinase	(1)	--
AT5G03730 serine/threonine-protein kinase CTR1	(1)	--
AT1G67890 PAS domain-containing protein tyrosine kinase	(1)	--
AT3G06620 PAS domain-containing tyrosine kinase-like	(1)	--
AT5G18700 protein kinase family protein with ARM repeat	(125)	SNILLDENGHIKLCDFGLSRKLDDISKSPSTGKRGTPYYMAPELYEDGGIHSFASDLWALGC
Pp3c17_9390V1.1 HIP7	(1)	--
Consensus	(125)	

	(187)	187 200 210 220 230 248
AT1G62400 protein high leaf temperature 1	(1)	--
Glycine max XP_003523074 PREDICTED: HT1-like	(1)	--
AT4G31170 protein kinase family protein	(1)	--
AT1G73660 MAPKKK-like kinase	(1)	--
AT5G03730 serine/threonine-protein kinase CTR1	(1)	--
AT1G67890 PAS domain-containing protein tyrosine kinase	(1)	--
AT3G06620 PAS domain-containing tyrosine kinase-like	(1)	--
AT5G18700 protein kinase family protein with ARM repeat	(187)	VLYECYTGRPPFVAREFTQLVKSIHSDPTPPLPGNASRSFVNLIESLLIKDPAQRIQWADLC
Pp3c17_9390V1.1 HIP7	(1)	--
Consensus	(187)	

```
                                                            (249) 249       260       270       280       290       300      310
                 AT1G62400 protein high leaf temperature 1    (1) --------------------------------------------------------------
           Glycine max XP_003523074 PREDICTED: HT1-like       (1) --------------------------------------------------------------
                 AT4G31170 protein kinase family protein      (1) --------------------------------------------------------------
                         AT1G73660 MAPKKK-like kinase         (1) -----MKVKEETLKNLGDGVVLRPVDHCSSIWSMKMNMKNFLKKLHIS----PNQSDEAEGS
        AT5G03730 serine/threonine-protein kinase CTR1         (1) MEMPGRRSNYTLLSQFSDDQVSVSVTGAPPPHYDSLSSENRSNHNSGNTGKAKAERGGFDWD
  AT1G67890 PAS domain-containing protein tyrosine kinase      (1) MENPNPPAEKLLKKIRELEESQEDLKREMSKLKVSAEIKRRSHSSSP---KRPSRRNSGEGT
     AT3G06620 PAS domain-containing tyrosine kinase-like      (1) MEN--PPAEELLKKILELEESQEHLKQEMSRLKVSTELRQRSHSVSP---HRPARRNIGEGA
  AT5G18700 protein kinase family protein with ARM repeat    (249) GHAFWKSKINLVQLPTQPAFDDMIGINTKPCLSERNGDRPNKTPPKYREKDRKGGSKQNENS
                                Pp3c17_9390V1.1 HIP7          (1) --------------------------------------------------------------
                                           Consensus        (249)                                                            E

                                                            (311) 311      320       330       340       350       360         372
                 AT1G62400 protein high leaf temperature 1    (1) --------------------------------------------------------------
           Glycine max XP_003523074 PREDICTED: HT1-like       (1) --------------------------------------------------------------
                 AT4G31170 protein kinase family protein      (1) --------------------------------------------------------------
                         AT1G73660 MAPKKK-like kinase        (54) ISTTKSNHHKSIDVSSSSSPRSHHSNSPEIKPFSGLSNWLSSVGHRKIPSPPNSFNAKNRAA
        AT5G03730 serine/threonine-protein kinase CTR1        (63) PSGGGGGDHRLNNQPNRVGNNMYASSLGLQRQSSGSSFGESSLSGDYYMPTLSAAANEIESV
  AT1G67890 PAS domain-containing protein tyrosine kinase     (60) PLWRKTGAASFRHASPLRKES----HSKDGVAGGGDGPSAGKFTDKQYLNILQSMAQAVHVF
     AT3G06620 PAS domain-containing tyrosine kinase-like     (58) PSWRKSGAASFRNASPLRKESRIQNSMRLRSEVGGGGPSAGKFTDKQYLNILQSMAQAVHAF
  AT5G18700 protein kinase family protein with ARM repeat    (311) IQGSKGHETPIKGTPGGSKAQAKLPSRATEEKHGGRPAANRQVNILRLSRIAKANLQKENEK
                                Pp3c17_9390V1.1 HIP7          (1) --------------------------------------------------------------
                                           Consensus        (311)                                   G                  S
```

```
                                                     (373) 373    380       390       400       410       420           434
                  AT1G62400 protein high leaf temperature 1   (1) --------------------------------------------------------------
        Glycine max XP_003523074 PREDICTED: HT1-like   (1) --------------------------------------------------------------
               AT4G31170 protein kinase family protein   (1) --------------------------------------------------------------
                           AT1G73660 MAPKKK-like kinase (116) TVDDTVVVNGSE--------------------HVDLGSKDPAVEEENQIQLALELSAREDPE
        AT5G03730 serine/threonine-protein kinase CTR1 (125) GFPQDDGFRLGFGGGGGDLRIQMAADSAGGSSSGKSWAQQTEESYQLQLALALRLSSEATCA
AT1G67890 PAS domain-containing protein tyrosine kinase (118) DLNGQIIFWNSM------------AEKLYGFSAAEALGKDSINILVDGQDAAVAKNIFQRCS
   AT3G06620 PAS domain-containing tyrosine kinase-like (120) DLNMRIIFWNAM------------AEKVYGYSAAEALGENPINVIADDRDAAFAMNIARRCV
AT5G18700 protein kinase family protein with ARM repeat (373) ENYRRPLPNSNENCAEVKIDNTDMELDFDENNDDEGPDESEGTENTSCAQEERVMSHNENHR
                                  Pp3c17_9390V1.1 HIP7   (1) --------------------------------------------------------------
                                              Consensus (373)

                                                     (435) 435  440       450       460       470       480             496
                  AT1G62400 protein high leaf temperature 1   (1) --------------------------------------------------------------
        Glycine max XP_003523074 PREDICTED: HT1-like   (1) --------------------------------------------------------------
               AT4G31170 protein kinase family protein   (1) --------------------------------------------------------------
                           AT1G73660 MAPKKK-like kinase (158) ATQIEAIKQFSLGSCAPENSPAELIAYRYWNYNCLGYDDKILDGFYDLYGVLNASSAERIPP
        AT5G03730 serine/threonine-protein kinase CTR1 (187) DDPNFLDPVPDESALRTSPSSAETVSHRFWVNGCLSYYDKVPDGFYMMNGLDPYIWTLCIDL
AT1G67890 PAS domain-containing protein tyrosine kinase (168) SGESWTGEFPVKNKMGERFSVVTTISPFYDDDGLLIGIICITNDSALFQRPRVPPAKNRWQ-
   AT3G06620 PAS domain-containing tyrosine kinase-like (170) RGESWTGEFPVKSKSGDRFSAVTTCSPFYDDDGALMGIICITSNTAPYLNPRISLAKLKAQE
AT5G18700 protein kinase family protein with ARM repeat (435) RQRVVSSNVPDENSSANETPTLGEARDCHEDQSEPMDMSAAPPSASPQLKTHRGRETSGVAV
                                  Pp3c17_9390V1.1 HIP7   (1) --------------------------------------------------------------
                                              Consensus (435)
```

```
                                                         (497) 497         510       520       530       540                 558
                     AT1G62400 protein high leaf temperature 1   (1) --------------------------------------------------------------
          Glycine max XP_003523074 PREDICTED: HT1-like   (1) --------------------------------------------------------------
              AT4G31170 protein kinase family protein   (1) --------------------------------------------------------------
                          AT1G73660 MAPKKK-like kinase (220) LLDLQGTPVSDGVTWEAVLVN-RSGDSNLLRLEQMALDIAAKSRSVSSSGFVNSELVRKLAI
        AT5G03730 serine/threonine-protein kinase CTR1 (249) HESGRIPSIESLRAVDSGVDSSLEAIIVDRRSDPAFKELHNRVHDISCSCITTKEVVDQLAK
AT1G67890 PAS domain-containing protein tyrosine kinase (229) EGDSSFCRGTNGVASRLGFDSKEAVVSKLGLDSQQPIQAAIASKISDLASKVGNKVRSKMRA
  AT3G06620 PAS domain-containing tyrosine kinase-like (232) EGETSSIPARNSFASKLGLDSRGAVISKLGLDSDQPIQVAIASKISDLASKVSNKVRSKMRA
 AT5G18700 protein kinase family protein with ARM repeat (497) NHDSSKAPTSLTDVFWHISDLSVRPVMPSRKSDKEAVHSLSFETPQPSDFSKKGKQELEPLN
                            Pp3c17_9390V1.1 HIP7   (1) --------------------------------------------------------------
                                         Consensus (497)

                                                         (559) 559       570       580       590       600       610       620
                     AT1G62400 protein high leaf temperature 1   (1) --------------------------------------------------------------
          Glycine max XP_003523074 PREDICTED: HT1-like   (1) --------------------------------------------------------------
              AT4G31170 protein kinase family protein   (1) --------------------------------------------------------------
                          AT1G73660 MAPKKK-like kinase (281) LVGDYMGGPVVHPESMLRAWRSLSYSLKATLGSMVLPLGSLTIGLARHRALLFKVLCDSVGV
        AT5G03730 serine/threonine-protein kinase CTR1 (311) LICNRMGGPVIMGEDELVPMWKECIDGLKEIFKVVVPIGSLSVGLCRHRALLFKVLADIIDL
AT1G67890 PAS domain-containing protein tyrosine kinase (291) GDNNASHPEGGNGGSHQSDQGFFDAAFSDQREDAETNDASTPRGNLIQSPFGVFLCNDDKSS
  AT3G06620 PAS domain-containing tyrosine kinase-like (294) GDNSATLSEGGSGDSHQKDHNVFGATLVDHRDDAASSGASTPRGDFIQSPFGVFTCNDEKFV
 AT5G18700 protein kinase family protein with ARM repeat (559) NRIITVLSGSSSGLSEKQNLIRYLETLSTNADAANILTNGPIMLVLVKVLRLSKTPAFRVQI
                            Pp3c17_9390V1.1 HIP7   (1) --------------------------------------------------------------
                                         Consensus (559)
```

```
                                                              (621) 621      630       640       650       660       670         682
AT1G62400 protein high leaf temperature 1                       (1) --------------------------------------------------------------
Glycine max XP_003523074 PREDICTED: HT1-like                    (1) --------------------------------------------------------------
AT4G31170 protein kinase family protein                         (1) --------MLENPKFDLHAVGNHNNDNNYYAFTQDFYQKLGEEGTNMS--------------
AT1G73660 MAPKKK-like kinase                                  (343) PCRIVKGQQYTGSEDVAMNFIKADDGREYIVDLMGDPGTLIPADAAGLQIDYDESAYSASPG
AT5G03730 serine/threonine-protein kinase CTR1                (373) PCRIAKGCKYCNRDDAASCLVRFGLDREYLVDLVGKPGHLWEPDSLLNGPSSISISSPLRFP
AT1G67890 PAS domain-containing protein tyrosine kinase      (353) SKASGESNDENDRNSVVPKKLTSKTEEWMVKKGLSWPWKGNEREGLER--------------
AT3G06620 PAS domain-containing tyrosine kinase-like         (356) SKPFKDSSDESDGKPAIHKVLTSKAEEWMVKKGLSWPWKGNEQEGSKG--------------
AT5G18700 protein kinase family protein with ARM repeat     (621) ASLIGLLIRHSTSIEDDLANSGILDSLTNGLRDKHEKVRRFSMAALGELLFYISTQNEHKDF
Pp3c17_9390V1.1 HIP7                                            (1) --------------------------------------------------------------
Consensus                                                     (621)

                                                              (683) 683   690       700       710       720       730             744
AT1G62400 protein high leaf temperature 1                       (1) --------------------------------------------------------------
Glycine max XP_003523074 PREDICTED: HT1-like                    (1) --------------------------------------M-----------------------
AT4G31170 protein kinase family protein                        (41) --------VDSMQTSNAGGSVSMSVDNSSVGSSDALIGH-----------------------
AT1G73660 MAPKKK-like kinase                                  (405) DNDSIHVASSSNGIESSYEENTEFRTGEHRSSTKSSGERNQSGGGGDLIVHPNISREDVKNQ
AT5G03730 serine/threonine-protein kinase CTR1                (435) RPKPVEPAVDFRLLAKQYFSDSQSLNLVFDPASDDMGFS-----------------------
AT1G67890 PAS domain-containing protein tyrosine kinase      (401) --------RNAHSVWPWVHNEQQKEEAHHSNSYNSVKSE-----------------------
AT3G06620 PAS domain-containing tyrosine kinase-like         (404) --------RPTNSVWPWVQNEQKKERCHQINPSAGVQYE-----------------------
AT5G18700 protein kinase family protein with ARM repeat     (683) KPPESPSKETRSASGWQVSNALISLVSSVLRKGEDDLTQVYALRTIENICSQGAYWATRFSS
Pp3c17_9390V1.1 HIP7                                            (1) --------------------------------------------------------------
Consensus                                                     (683)
```

```
                                                   (745) 745  750                 760                 770                 780                 790                           806
               AT1G62400 protein high leaf temperature 1   (1) --------------------------------------------------------------
        Glycine max XP_003523074 PREDICTED: HT1-like   (2) --------------------------------------------------------------
               AT4G31170 protein kinase family protein  (72) --------------------------------------------------------------
                          AT1G73660 MAPKKK-like kinase (467) KKVEKAPFQNLSSRPIHSFTHMRSPSWTEGVSSPAAQRMKVKDVSQYMIDAAKENPRLAQKL
         AT5G03730 serine/threonine-protein kinase CTR1 (474) --------------------------------------------------------------
AT1G67890 PAS domain-containing protein tyrosine kinase (432) --------------------------------------------------------------
   AT3G06620 PAS domain-containing tyrosine kinase-like (435) --------------------------------------------------------------
AT5G18700 protein kinase family protein with ARM repeat (745) QDLISNLCYIYKATGKQESMRQTAGSCLVRLARFNPPCIQTVVEKLSLKEIASSFVKGSARE
                                 Pp3c17_9390V1.1 HIP7   (1) --------------------------------------------------------------
                                                       (745)
```

```
                                                   (807) 807            820                 830                 840                 850                           868
               AT1G62400 protein high leaf temperature 1   (1) --------------------------------------------------------------
        Glycine max XP_003523074 PREDICTED: HT1-like   (2) --------------------------------------------------------------
               AT4G31170 protein kinase family protein  (72) --------------------------------------------------------------
                          AT1G73660 MAPKKK-like kinase (529) HDVLLESGVVAPPNLFSEVYPQQLEATVESKNSTEAKKERGKDLETTQEGRHQNGFGPVRFL
         AT5G03730 serine/threonine-protein kinase CTR1 (474) --------------------------------------------------------------
AT1G67890 PAS domain-containing protein tyrosine kinase (432) --------------------------------------------------------------
   AT3G06620 PAS domain-containing tyrosine kinase-like (435) --------------------------------------------------------------
AT5G18700 protein kinase family protein with ARM repeat (807) QQVCLNLLNMAMIGSHTFTSFGRHLVTLTEEKNLFPSLLSIIEQGTEVLRGKALLFVAFLCK
                                 Pp3c17_9390V1.1 HIP7   (1) --------------------------------------------------------------
                                           Consensus (807)
```

```
                                                  (869) 869        880       890       900       910       920       930
AT1G62400 protein high leaf temperature 1           (1) --------------------------------------------------------MEKKRF
Glycine max XP_003523074 PREDICTED: HT1-like        (2) -------------------KNLKWHKQISNSGNKLG------------------RRLSLGEY
AT4G31170 protein kinase family protein            (72) -------------------PGLKPMRHPYSLSDGQS------------------VFRPGKVT
AT1G73660 MAPKKK-like kinase                      (591) PPLPRVQSKTNAHDQRDNGKVVSQSDSSHSEASSTEYARTVPAAVAAAAVVASSMVAAAAAK
AT5G03730 serine/threonine-protein kinase CTR1    (474) -------------------MFHRQYDNPGGENDALAENGGGSLPPSANMPPQNMMRASNQIE
AT1G67890 PAS domain-containing protein tyrosine kinase (432) -------------------SLASESNKPANNEN----------------------MGSVNVN
AT3G06620 PAS domain-containing tyrosine kinase-like (435) -------------------SHAFESNKPINNEASSL------------------WSSPINAN
AT5G18700 protein kinase family protein with ARM repeat (869) NSRRWLTNFFCNARFLPVVDRLAKEKDSYLQQCLEAFVNVIASIIPGMLDTITNDIQQLMTG
Pp3c17_9390V1.1 HIP7                                (1) --------------------------------------------------------------
Consensus                                         (869)

                                                  (931) 931      940       950       960       970       980        992
AT1G62400 protein high leaf temperature 1           (7) DSMESWSMILE---------------------------------------------------
Glycine max XP_003523074 PREDICTED: HT1-like       (27) NRAVSWSKYLV---------------------------------------------------
AT4G31170 protein kinase family protein            (97) HALNDDALAQA---------------------------------------------------
AT1G73660 MAPKKK-like kinase                      (653) SANSDSSPIELPAAAAATATAAAVVATAAAVSRQLELGSNSDGDDGSGGHEPQGSGDSNHGP
AT5G03730 serine/threonine-protein kinase CTR1    (517) AAPMNAPPISQ---------------------------------------------------
AT1G67890 PAS domain-containing protein tyrosine kinase (453) SASSASSCGST---------------------------------------------------
AT3G06620 PAS domain-containing tyrosine kinase-like (460) STSSASSCGST---------------------------------------------------
AT5G18700 protein kinase family protein with ARM repeat (931) RRHGPVSPLNSRAPVKTNAHLFPVVLHLLGSSSFKNKMVTPQVLRQLANLTKLVEASFQGRD
Pp3c17_9390V1.1 HIP7                                (1) --------------------------------------------------------------
Consensus                                         (931)  A    S
```

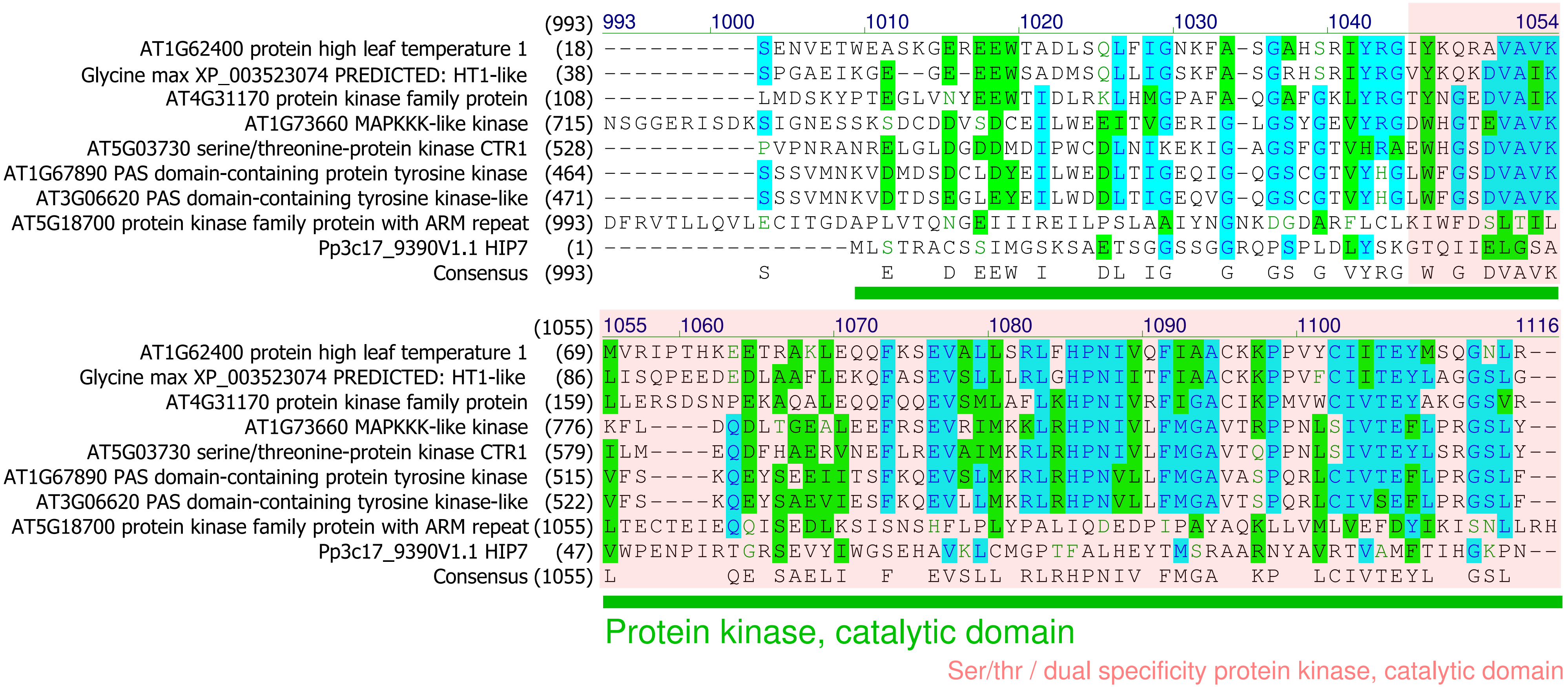
(993) 993 1000 1010 1020 1030 1040 1054
AT1G62400 protein high leaf temperature 1 (18) ----------SENVETWEASKGEREEWTADLSQLFIGNKFA-SGAHSRIYRGIYKQRAVAVK
Glycine max XP_003523074 PREDICTED: HT1-like (38) ----------SPGAEIKGE--GE-EEWSADMSQLLIGSKFA-SGRHSRIYRGVYKQKDVAIK
AT4G31170 protein kinase family protein (108) ----------LMDSKYPTEGLVNYEEWTIDLRKLHMGPAFA-QGAFGKLYRGTYNGEDVAIK
AT1G73660 MAPKKK-like kinase (715) NSGGERISDKSIGNESSKSDCDDVSDCEILWEEITVGERIG-LGSYGEVYRGDWHGTEVAVK
AT5G03730 serine/threonine-protein kinase CTR1 (528) ----------PVPNRANRELGLDGDDMDIPWCDLNIKEKIG-AGSFGTVHRAEWHGSDVAVK
AT1G67890 PAS domain-containing protein tyrosine kinase (464) ----------SSSVMNKVDMDSDCLDYEILWEDLTIGEQIG-QGSCGTVYHGLWFGSDVAVK
AT3G06620 PAS domain-containing tyrosine kinase-like (471) ----------SSSVMNKVDTDSEGLEYEILWDDLTIGEQVG-QGSCGTVYHGLWFGSDVAVK
AT5G18700 protein kinase family protein with ARM repeat (993) DFRVTLLQVLECITGDAPLVTQNGEIIIREILPSLAAIYNGNKDGDARFLCLKIWFDSLTIL
Pp3c17_9390V1.1 HIP7 (1) ----------------MLSTRACSSIMGSKSAETSGGSSGGRQPSPLDLYSKGTQIIELGSA
Consensus (993) S E D EEW I DL IG G GS G VYRG W G DVAVK
(1055) 1055 1060 1070 1080 1090 1100 1116
AT1G62400 protein high leaf temperature 1 (69) MVRIPTHKEETRAKLEQQFKSEVALLSRLFHPNIVQFIAACKKPPVYCIITEYMSQGNLR--
Glycine max XP_003523074 PREDICTED: HT1-like (86) LISQPEEDEDLAAFLEKQFASEVSLLLRLGHPNIITFIAACKKPPVFCIITEYLAGGSLG--
AT4G31170 protein kinase family protein (159) LLERSDSNPEKAQALEQQFQQEVSMLAFLKHPNIVRFIGACIKPMVWCIVTEYAKGGSVR--
AT1G73660 MAPKKK-like kinase (776) KFL----DQDLTGEALEEFRSEVRIMKKLRHPNIVLFMGAVTRPPNLSIVTEFLPRGSLY--
AT5G03730 serine/threonine-protein kinase CTR1 (579) ILM----EQDFHAERVNEFLREVAIMKRLRHPNIVLFMGAVTQPPNLSIVTEYLSRGSLY--
AT1G67890 PAS domain-containing protein tyrosine kinase (515) VFS----KQEYSEEIITSFKQEVSLMKRLRHPNVLLFMGAVASPQRLCIVTEFLPRGSLF--
AT3G06620 PAS domain-containing tyrosine kinase-like (522) VFS----KQEYSAEVIESFKQEVLLMKRLRHPNVLLFMGAVTSPQRLCIVSEFLPRGSLF--
AT5G18700 protein kinase family protein with ARM repeat (1055) LTECTEIEQQISEDLKSISNSHFLPLYPALIQDEDPIPAYAQKLLVMLVEFDYIKISNLLRH
Pp3c17_9390V1.1 HIP7 (47) VWPENPIRTGRSEVYIWGSEHAVKLCMGPTFALHEYTMSRAARNYAVRTVAMFTIHGKPN--
Consensus (1055) L QE SAELI F EVSLL RLRHPNIV FMGA KP LCIVTEYL GSL
Protein kinase, catalytic domain
Ser/thr / dual specificity protein kinase, catalytic domain

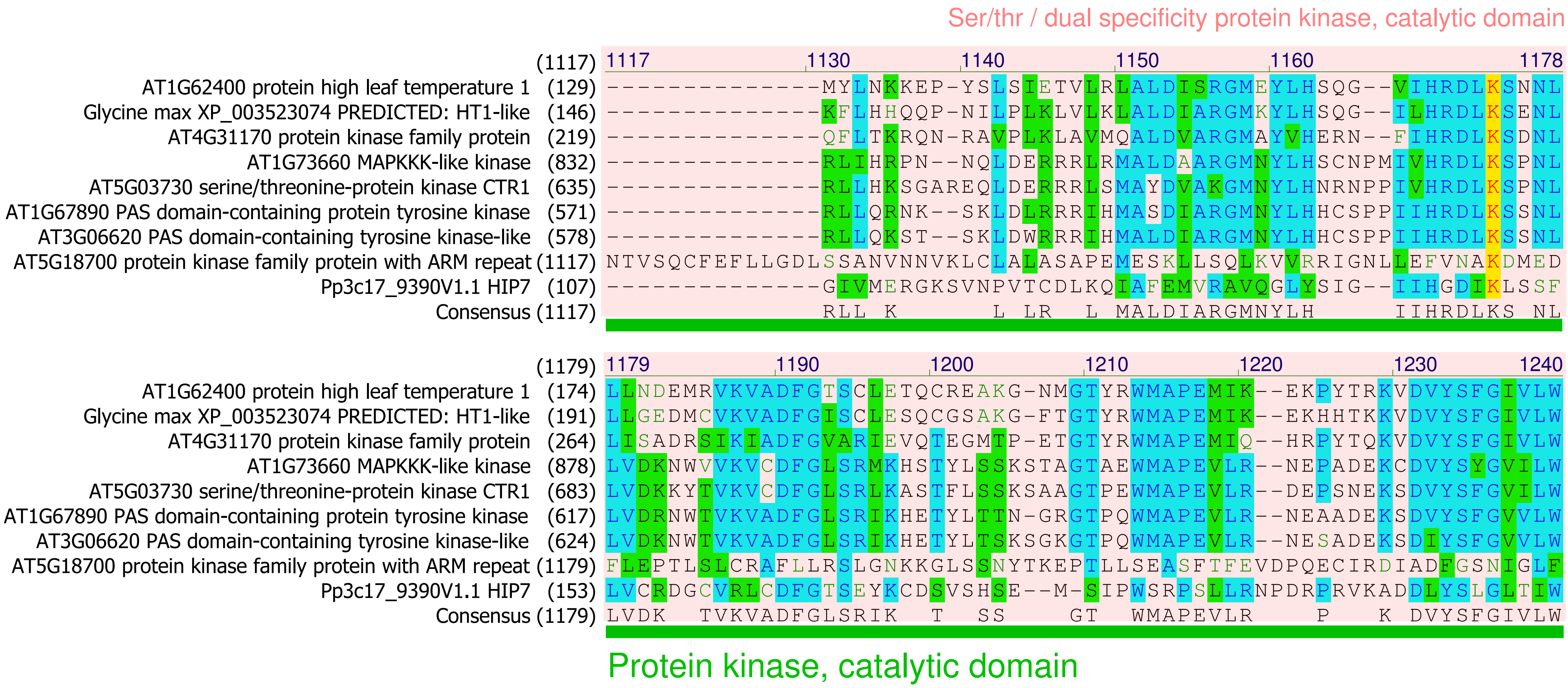
Ser/thr / dual specificity protein kinase, catalytic domain
(1117) 1117 1130 1140 1150 1160 1178
AT1G62400 protein high leaf temperature 1 (129) --------------MYLNKKEP-YSLSIETVLRLALDISRGMEYLHSQG--VIHRDLKSNNL
Glycine max XP_003523074 PREDICTED: HT1-like (146) --------------KFLHHQQP-NILPLKLVLKLALDIARGMKYLHSQG--ILHRDLKSENL
AT4G31170 protein kinase family protein (219) --------------QFLTKRQN-RAVPLKLAVMQALDVARGMAYVHERN--FIHRDLKSDNL
AT1G73660 MAPKKK-like kinase (832) --------------RLIHRPN--NQLDERRRLRMALDAARGMNYLHSCNPMIVHRDLKSPNL
AT5G03730 serine/threonine-protein kinase CTR1 (635) --------------RLLHKSGAREQLDERRRLSMAYDVAKGMNYLHNRNPPIVHRDLKSPNL
AT1G67890 PAS domain-containing protein tyrosine kinase (571) --------------RLLQRNK--SKLDLRRRIHMASDIARGMNYLHHCSPPIIHRDLKSSNL
AT3G06620 PAS domain-containing tyrosine kinase-like (578) --------------RLLQKST--SKLDWRRRIHMALDIARGMNYLHHCSPPIIHRDLKSSNL
AT5G18700 protein kinase family protein with ARM repeat (1117) NTVSQCFEFLLGDLSSANVNNVKLCLALASAPEMESKLLSQLKVVRRIGNLLEFVNAKDMED
Pp3c17_9390V1.1 HIP7 (107) --------------GIVMERGKSVNPVTCDLKQIAFEMVRAVQGLYSIG--IIHGDIKLSSF
Consensus (1117) RLL K L LR L MALDIARGMNYLH IIHRDLKS NL
(1179) 1179 1190 1200 1210 1220 1230 1240
AT1G62400 protein high leaf temperature 1 (174) LLNDEMRVKVADFGTSCLETQCREAKG-NMGTYRWMAPEMIK--EKPYTRKVDVYSFGIVLW
Glycine max XP_003523074 PREDICTED: HT1-like (191) LLGEDMCVKVADFGISCLESQCGSAKG-FTGTYRWMAPEMIK--EKHHTKKVDVYSFGIVLW
AT4G31170 protein kinase family protein (264) LISADRSIKIADFGVARIEVQTEGMTP-ETGTYRWMAPEMIQ--HRPYTQKVDVYSFGIVLW
AT1G73660 MAPKKK-like kinase (878) LVDKNWVVKVCDFGLSRMKHSTYLSSKSTAGTAEWMAPEVLR--NEPADEKCDVYSYGVILW
AT5G03730 serine/threonine-protein kinase CTR1 (683) LVDKKYTVKVCDFGLSRLKASTFLSSKSAAGTPEWMAPEVLR--DEPSNEKSDVYSFGVILW
AT1G67890 PAS domain-containing protein tyrosine kinase (617) LVDRNWTVKVADFGLSRIKHETYLTTN-GRGTPQWMAPEVLR--NEAADEKSDVYSFGVVLW
AT3G06620 PAS domain-containing tyrosine kinase-like (624) LVDKNWTVKVADFGLSRIKHETYLTSKSGKGTPQWMAPEVLR--NESADEKSDIYSFGVVLW
AT5G18700 protein kinase family protein with ARM repeat (1179) FLEPTLSLCRAFLLRSLGNKKGLSSNYTKEPTLLSEASFTFEVDPQECIRDIADFGSNIGLF
Pp3c17_9390V1.1 HIP7 (153) LVCRDGCVRLCDFGTSEYKCDSVSHSE--M-SIPWSRPSLLRNPDRPRVKADDLYSLGLTIW
Consensus (1179) LVDK TVKVADFGLSRIK T SS GT WMAPEVLR P K DVYSFGIVLW
Protein kinase, catalytic domain

Ser/thr / dual specificity protein kinase, catalytic domain

```
                                                           (1241) 1241     1250      1260      1270      1280      1290        1302
AT1G62400 protein high leaf temperature 1                   (233) ELTTALLPFQGMTPVQAAFAVAEKNERPPLPASCQPALAHLIKRCWSENPSKRPDFSNIVAV
Glycine max XP_003523074 PREDICTED: HT1-like                (250) ELLTGKTPFDNMTPEQAAYAVSHKNARPPLPSKCPWAFSDLINRCWSSNPDKRPHFDEIVSI
AT4G31170 protein kinase family protein                     (323) ELITGLLPFQNMTAVQAAFAVVNRGVRPTVPADCLPVLGEIMTRCWDADPEVRPCFAEIVNL
AT1G73660 MAPKKK-like kinase                                (938) ELFTLQQPWGKMNPMQVVGAVGFQHRRLDIPDFVDPAIADLISKCWQTDSKLRPSFAEIMAS
AT5G03730 serine/threonine-protein kinase CTR1              (743) ELATLQQPWGNLNPAQVVAAVGFKCKRLEIPRNLNPQVAAIIEGCWTNEPWKRPSFATIMDL
AT1G67890 PAS domain-containing protein tyrosine kinase    (676) ELVTEKIPWENLNAMQVIGAVGFMNQRLEVPKDVDPQWIALMESCWHSEPQCRPSFQELMDK
AT3G06620 PAS domain-containing tyrosine kinase-like       (684) ELATEKIPWETLNSMQVIGAVGFMDQRLEIPKDIDPRWISLMESCWHSDTKLRPTFQELMDK
AT5G18700 protein kinase family protein with ARM repeat   (1241) LHFAGLDDDTSIAVADIASECVVLLLKAASREATTGFLTNLPKITPILDSWRRRKSTELHLL
Pp3c17_9390V1.1 HIP7                                        (212) ELYTGKVPFVPPTSEGWESLDINEVAEEAILAGEQVDLNDILDLEIRCGCFTCERGGRAPTL
Consensus                                                  (1241) EL TG IPF  M  MQV  AVG    R  IP    P L  LI  CW  DP  RP F EIM L

                                                           (1303) 1303      1310      1320      1330      1340      1350        1364
AT1G62400 protein high leaf temperature 1                   (295) LEKYDECVKEGLPLTSHASLTKTKKAILDHLKGCVTSISSPFSSSSVPVNA-----------
Glycine max XP_003523074 PREDICTED: HT1-like                (312) LEYYTESLQQDP---EFFSTYKPSPTSSNTILGCLPKCNARHKFGACKV-------------
AT4G31170 protein kinase family protein                     (385) LEAAETEIMTNVRKARFRCCMTQPMTVD----------------------------------
AT1G73660 MAPKKK-like kinase                               (1000) LKRLQKPVTGSNIPRPVPSSSSLPTEHEQKD-------------------------------
AT5G03730 serine/threonine-protein kinase CTR1              (805) LRPLIKSAVPPPNRSDL---------------------------------------------
AT1G67890 PAS domain-containing protein tyrosine kinase    (738) LRELQRKYTIQFQAARAASIDNSSLKEK----------------------------------
AT3G06620 PAS domain-containing tyrosine kinase-like       (746) LRDLQRKYMIQFQATRAALSDNSLLKDN----------------------------------
AT5G18700 protein kinase family protein with ARM repeat   (1303) VLKRVLHCLGYACKQYLSQAMILSISGHDVSKINAIVSEMKNSDAAGLNSIASLVAMELQRL
Pp3c17_9390V1.1 HIP7                                        (274) PGVVTWGFRKVAKY------------------------------------------------
Consensus                                                  (1303) L  L
```

Protein kinase, catalytic domain

	(1365)		1366
AT1G62400 protein high leaf temperature 1	(346)	--	
Glycine max XP_003523074 PREDICTED: HT1-like	(358)	--	
AT4G31170 protein kinase family protein	(413)	--	
AT1G73660 MAPKKK-like kinase	(1031)	--	
AT5G03730 serine/threonine-protein kinase CTR1	(822)	--	
AT1G67890 PAS domain-containing protein tyrosine kinase	(766)	--	
AT3G06620 PAS domain-containing tyrosine kinase-like	(774)	--	
AT5G18700 protein kinase family protein with ARM repeat	(1365)	PR	
Pp3c17_9390V1.1 HIP7	(288)	--	
Consensus	(1365)		
