# Supplementary material for: Holophytochrome-Interacting Proteins in Physcomitrella: Putative Actors in Phytochrome Cytoplasmic Signaling
Source: Front Plant Sci. 2016 May 12;7:613. doi: 10.3389/fpls.2016.00613 (PMC4867686; doi:10.3389/fpls.2016.00613)
Supplement: Supplementary file 2 [file Data_Sheet_2.ZIP › SI/SI HIP8.pdf]

frontiers

*Supplementary Material*

# Holophytochrome-interacting proteins in *Physcomitrella*: putative actors in phytochrome cytoplasmic signaling

**Anna Lena Ermert, Katharina Mailliet, and Jon Hughes***

*** Correspondence:** jon.hughes@uni-giessen.de

**HIP8** (Pp3c11_25550V1.1)

```
ATGGCGGATTTTGAAGTATTGGTCGGCTTCCAGGAGCATGTGCCCCTCCCTGCGCTGTTGCAAAACGCGACGGATGATCTA
GAGAAACTATATTCTCTCGGGAAGAAACTGGGAGAGGGTCAATTTGGAACTACGTATCTCTGCACCGAGCGTGCGACAGGG
TTACAATTCGCTTGCAAGTGCATTCCCAAGCGAAAACTCATCTCCTCAGAGGAAATCGAAGATGTCGGCCGGGAGGTCGAG
GTTATGTATCACCTCTCTGGCCACCCTAACATCGTCACCCTCAAGGGTGCATACGAGGATGCCACCAATGTCTACTTGGTG
ATGGAGCTTTGCGAGGGTGGGGAGCTTTTCGATCGTATTATTGAACGCGGGACTTACACGGAAGCAGAAGCTGCTCGTCTG
ACAAGAACAATAGTTAGCGTCGTCGAAGCGTGTCACAAATCTGGTGTTGTGCACCGGGATCTGAAGCCGGAAAATTTTCTA
TTCAAGACCAAGGAAGACGATTCTGTTTTGAAGGCTGCGGATTTTGGCTCGGCTAGGTTTTTCGAGCCGGGAGATGTGTTT
ACTGATATTGTCGGAAGCCCCTACTATGTTGCGCCGGAGGTCTTGGATCGGCATTACGGGCCAGAAGCTGACATCTGGAGT
GCTGGGGTTATGTTGTACATTTTGTTGAGCGGTGCTCCACCGTTTTGGGCAGAGACAGTGCAGGGTATATTCGAGAAAGTC
ATGGAGGGGGAACCCCCGACCTTTACCGCCGATCCTTGGCCGAACATTTCGGAGGTGGCGAAGGATTTGATCCGAAAGATG
TTGGATCCCAATCCAGAAAAGCGGCTTAAAGCCCACGAGGTTTTGAATCACCCTTGGATTCGTGAGGATGGTGTGGCGCCT
AAGAAGCCTATCGCGTCCCTCGTCCAGTTCCGGATGAAGCAATTCGCTGCGATGAACAAGCTGAAGAAACTGGCAATTCGG
ATCATTGCCGAGACGCTCTCGGAGGAGGAGATAGCAAATTTAAAGGAAATTTTCACCGAGATGGATAGCGACAACGATGGT
GCCATAAGCTTCGAGGAGCTGAAAGCGGGTCTGCTCAGGGTGGGGACATCCCTCAAGGACGCTGAGCTATTCGACCTTATG
GATGCTGCAGATGTCGACCACGATGGCATGATTGATTGCGGGGAATTTCTAGCTGCCACTCTGAGCCTTAATCACATTGAA
TTGGAGGAGAATCTCATGGCGGCCTTTCAGTATCTCGATAAGAGCGGTAGCGGATATATCACCACCGATGAGCTCCTCGCT
GTCTGCTTCGAATTTCACATGGAGGACGTACGCTTGGAGGATCTTTTACATGACGTGAGCATTGGCGCAGACGGTAGTATT
GACTACAAGATGTTCGTGACCATGATGCGCAAGTGCAATGGCGGCATGGGTCATCAGAATCTGCGGTGTACCCTGGGCATC
ACAGACGTTCTGACACTAGAGGAGCAGTACTAG
```

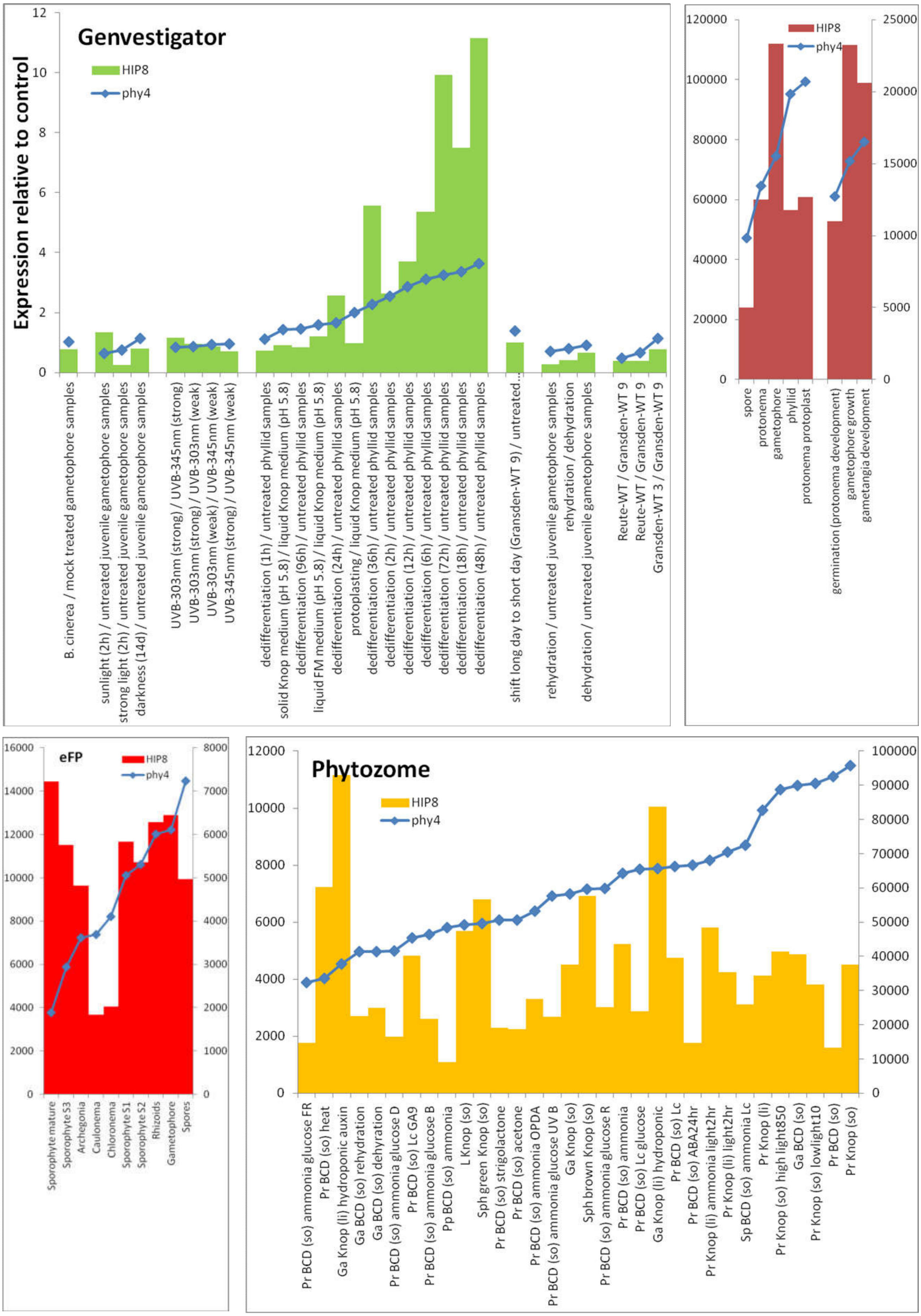

Genvestigator
HIP8
phy4
Expression relative to control
B. cinerea / mock treated gametophore samples
sunlight (2h) / untreated juvenile gametophore samples
strong light (2h) / untreated juvenile gametophore samples
darkness (14d) / untreated juvenile gametophore samples
UVB-303nm (strong) / UVB-345nm (strong)
UVB-303nm (strong) / UVB-303nm (weak)
UVB-303nm (weak) / UVB-345nm (weak)
UVB-345nm (strong) / UVB-345nm (weak)
dedifferentiation (1h) / untreated phyllid samples
solid Knop medium (pH 5.8) / liquid Knop medium (pH 5.8)
dedifferentiation (96h) / untreated phyllid samples
liquid FM medium (pH 5.8) / liquid Knop medium (pH 5.8)
dedifferentiation (24h) / untreated phyllid samples
protoplasting / liquid Knop medium (pH 5.8)
dedifferentiation (36h) / untreated phyllid samples
dedifferentiation (2h) / untreated phyllid samples
dedifferentiation (12h) / untreated phyllid samples
dedifferentiation (6h) / untreated phyllid samples
dedifferentiation (72h) / untreated phyllid samples
dedifferentiation (18h) / untreated phyllid samples
dedifferentiation (48h) / untreated phyllid samples
shift long day to short day (Gransden-WT 9) / untreated...
rehydration / untreated juvenile gametophore samples
rehydration / dehydration
dehydration / untreated juvenile gametophore samples
Reute-WT / Gransden-WT 9
Reute-WT / Gransden-WT 9
Gransden-WT 3 / Gransden-WT 9
HIP8
phy4
spore
protonema
gametophore
phyllid
protonema protoplast
germination (protonema development)
gametophore growth
gametangia development
eFP
HIP8
phy4
Sporophyte mature
Sporophyte S3
Archegonia
Caulonema
Chloronema
Sporophyte S1
Sporophyte S2
Rhizoids
Gametophore
Spores
Phytozome
HIP8
phy4
Pr BCD (so) ammonia glucose FR
Pr BCD (so) heat
Ga Knop (li) hydroponic auxin
Ga BCD (so) rehydration
Ga BCD (so) dehyration
Pr BCD (so) ammonia glucose D
Pr BCD (so) Lc GA9
Pr BCD (so) ammonia glucose B
Pp BCD (so) ammonia
L Knop (so)
Sph green Knop (so)
Pr BCD (so) strigolactone
Pr BCD (so) acetone
Pr BCD (so) ammonia OPDA
Pr BCD (so) ammonia glucose UV B
Ga Knop (so)
Sph brown Knop (so)
Pr BCD (so) ammonia glucose R
Pr BCD (so) ammonia
Pr BCD (so) Lc glucose
Ga Knop (li) hydroponic
Pr BCD (so) Lc
Pr BCD (so) ABA24hr
Pr Knop (li) ammonia light2hr
Pr Knop (li) light2hr
Sp BCD (so) ammonia Lc
Pr Knop (li)
Pr Knop (so) high light850
Ga BCD (so)
Pr Knop (so) lowlight10
Pr BCD (so)
Pr Knop (so)

# HIP8 alignment tree

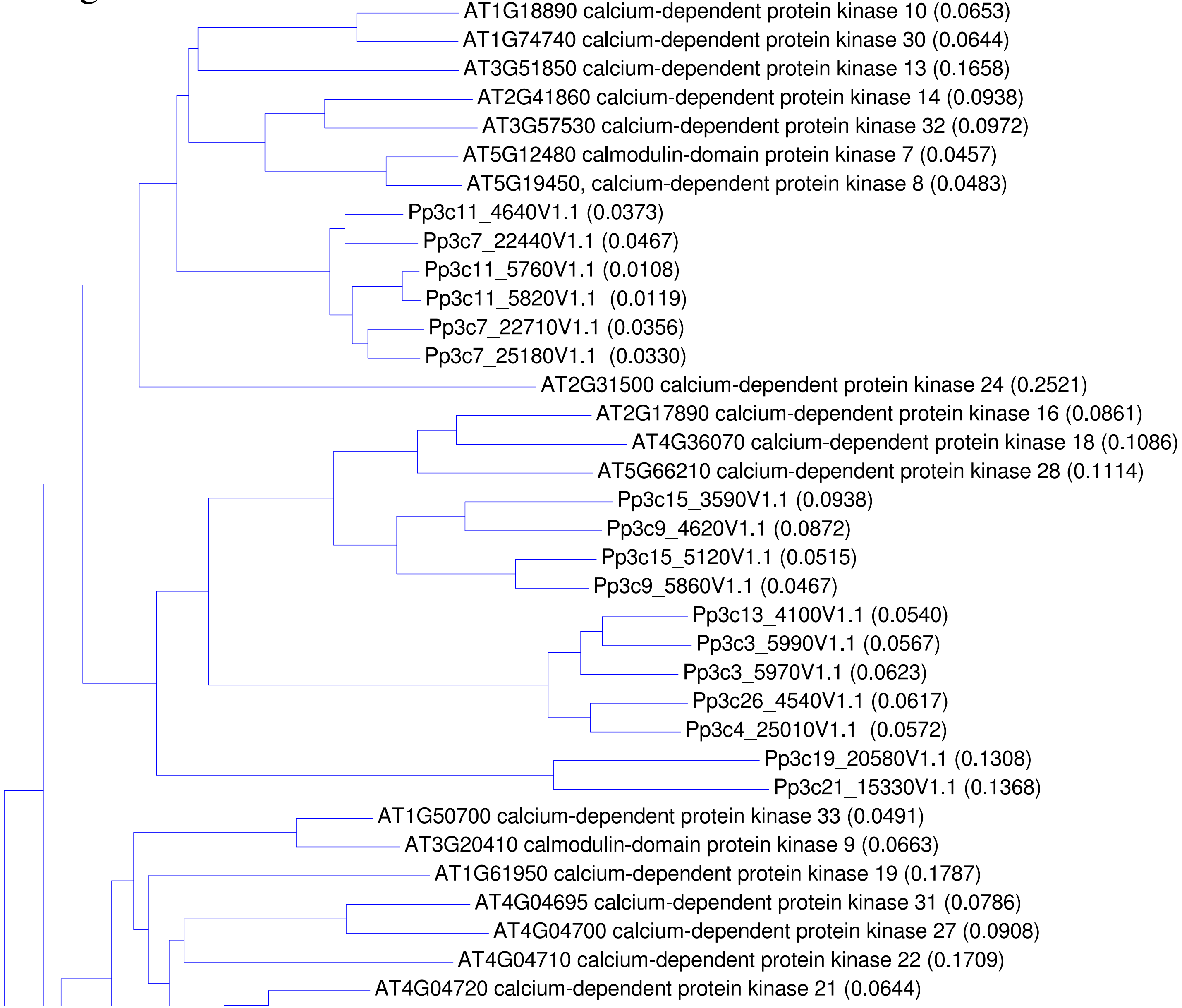

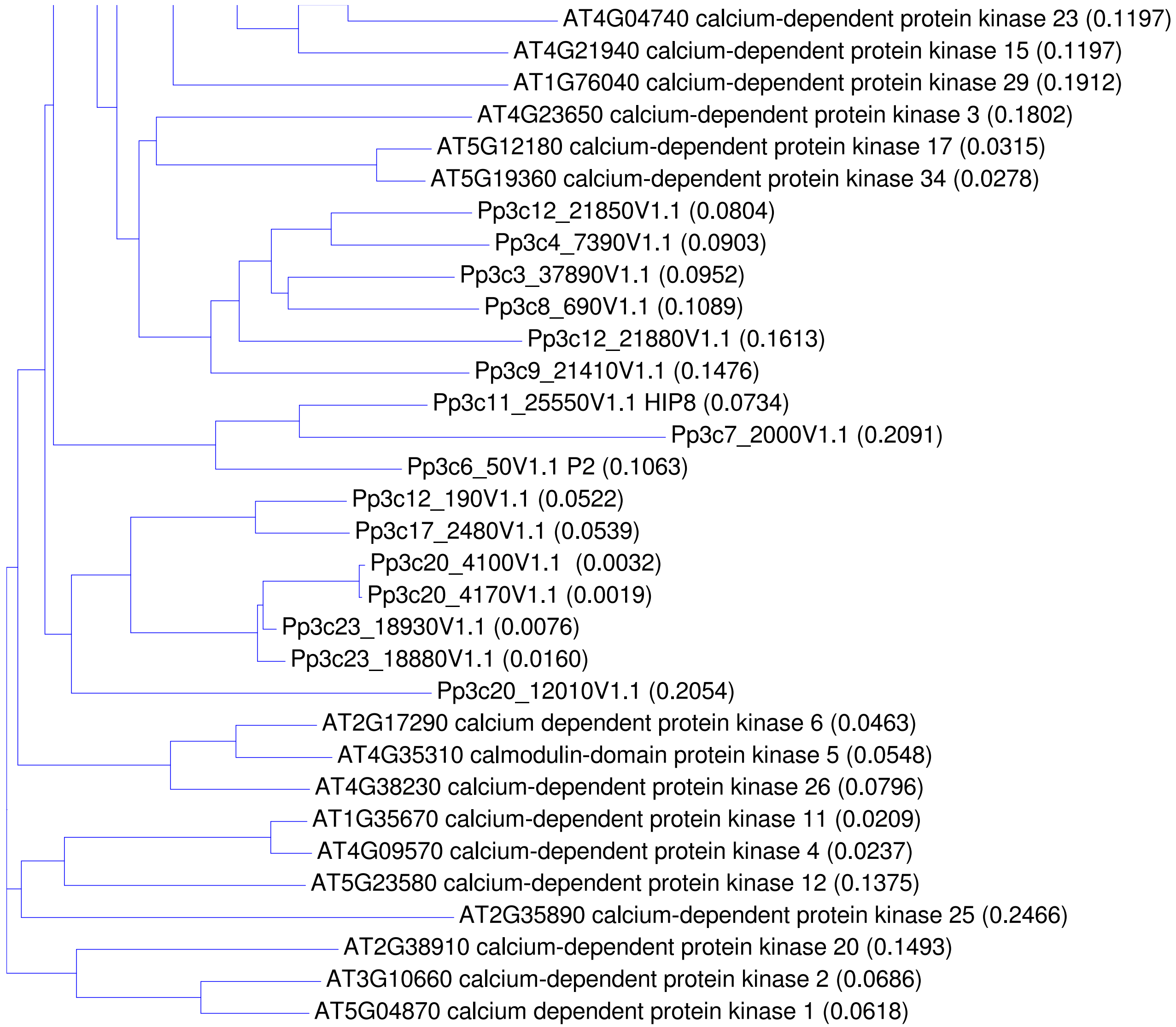

AT4G04740 calcium-dependent protein kinase 23 (0.1197)
AT4G21940 calcium-dependent protein kinase 15 (0.1197)
AT1G76040 calcium-dependent protein kinase 29 (0.1912)
AT4G23650 calcium-dependent protein kinase 3 (0.1802)
AT5G12180 calcium-dependent protein kinase 17 (0.0315)
AT5G19360 calcium-dependent protein kinase 34 (0.0278)
Pp3c12_21850V1.1 (0.0804)
Pp3c4_7390V1.1 (0.0903)
Pp3c3_37890V1.1 (0.0952)
Pp3c8_690V1.1 (0.1089)
Pp3c12_21880V1.1 (0.1613)
Pp3c9_21410V1.1 (0.1476)
Pp3c11_25550V1.1 HIP8 (0.0734)
Pp3c7_2000V1.1 (0.2091)
Pp3c6_50V1.1 P2 (0.1063)
Pp3c12_190V1.1 (0.0522)
Pp3c17_2480V1.1 (0.0539)
Pp3c20_4100V1.1 (0.0032)
Pp3c20_4170V1.1 (0.0019)
Pp3c23_18930V1.1 (0.0076)
Pp3c23_18880V1.1 (0.0160)
Pp3c20_12010V1.1 (0.2054)
AT2G17290 calcium dependent protein kinase 6 (0.0463)
AT4G35310 calmodulin-domain protein kinase 5 (0.0548)
AT4G38230 calcium-dependent protein kinase 26 (0.0796)
AT1G35670 calcium-dependent protein kinase 11 (0.0209)
AT4G09570 calcium-dependent protein kinase 4 (0.0237)
AT5G23580 calcium-dependent protein kinase 12 (0.1375)
AT2G35890 calcium-dependent protein kinase 25 (0.2466)
AT2G38910 calcium-dependent protein kinase 20 (0.1493)
AT3G10660 calcium-dependent protein kinase 2 (0.0686)
AT5G04870 calcium dependent protein kinase 1 (0.0618)

# HIP8 alignment

```
                                                  (1) 1        10        20        30        40        50              66
AT1G18890 calcium-dependent protein kinase 10     (1) ----------------------------MGNCNACVRPDSKESKPSSKPKKPNRDRKLNPFAG-DF
AT1G74740 calcium-dependent protein kinase 30     (1) ----------------------------MGNCIACVKFDPDNSKPNQK-KKPPRGRQRNPYDDPDG
AT3G51850 calcium-dependent protein kinase 13     (1) -------------------------------MGNCCRSPAAVAREDVKSNYSGHDHARKDAAG---
AT2G41860 calcium-dependent protein kinase 14     (1) ----------------------------MGNCCGTAGSLIQDKQK--KGFKLPNPFSNEYGNHHDG
AT3G57530 calcium-dependent protein kinase 32     (1) ----------------------------MGNCCGTAGSLAQNDNKPKKGRKKQNPFSIDYGLHHGG
AT5G12480 calmodulin-domain protein kinase 7      (1) ----------------------------MGNCCGNPSSATNQSKQG-KPKNKNNPFYSNEYATTDR
AT5G19450, calcium-dependent protein kinase 8     (1) ----------------------------MGNCCASPGSETG-SKKG-KPKIKSNPFYSEAYTTNG-
Pp3c11_4640V1.1                                   (1) ----------------------------MGNCCVGSTTKK--------PKQRPN-PFAQDGYQTNL
Pp3c7_22440V1.1                                   (1) ----------------------------MGNCCAGSATKK--------PKQRPN-PFAQDGYQANS
Pp3c11_5760V1.1                                   (1) ----------------------------MGNCCVGSSTKKS------RKERRQNPFAPQDGYQTNN
Pp3c11_5820V1.1                                   (1) ----------------------------MGNCCVGSSSNKS------RRARRAN-LFAHDRYYGNN
Pp3c7_22710V1.1                                   (1) ----------------------------MGNCCVGSSTKKS------RQERRTNPFAPQDGFQSTN
Pp3c7_25180V1.1                                   (1) ----------------------------MGNCCVGSSTKKS------RKERRPNPFAQGD-YHTNL
AT2G31500 calcium-dependent protein kinase 24     (1) ----------------------------MGSCVSSPLKGSPFGKRPVRRRHSSNSRTSSVPRFDSS
AT2G17890 calcium-dependent protein kinase 16     (1) ----------------------------MGLCFSSAAKS---SGHNR-SSRNPHPHPPLTVVKSRP
AT4G36070 calcium-dependent protein kinase 18     (1) ----------------------------MGLCFSSPK-A---TRRGT-GSRNPNPDSPTQGKASEK
AT5G66210 calcium-dependent protein kinase 28     (1) ----------------------------MGVCFSAIR-V---TGASS-SRRS---S-QTKSKAAPT
Pp3c15_3590V1.1                                   (1) ----------------------------MGSCCTKSSVV---EEPIP-VRKHQSSVQGSSKQGSST
Pp3c9_4620V1.1                                    (1) ----------------------------MGNCCKKSSVA---EEVT---RKYKAPEQWITKNEPSA
Pp3c15_5120V1.1                                   (1) ----------------------------MGGCCSKSSVV---EQRPQ--APHRTEANGKEKEAAYQ
Pp3c9_5860V1.1                                    (1) ----------------------------MGGFCSKTPVV---EDRKK--APHRKDVNGREKEGANG
Pp3c13_4100V1.1                                   (1) ----------------------------MGQCYGKYVGP-EGDDFNN-GDEFSASKTPISKHGSRR
Pp3c3_5990V1.1                                    (1) ----------------------------MGQCYGKYEGP-EGDDSFN-GDGHSVPKTPNSKHGSYN
Pp3c3_5970V1.1                                    (1) ----------------------------MGQCYGKYEGA-ERDDSFA-HDEFRVPKNP--KHGSYS
Pp3c26_4540V1.1                                   (1) ----------------------------MGQCYGKHGDEGDGEDSSEERHKVQVTRTP--KHGSWN
Pp3c4_25010V1.1                                   (1) ----------------------------MGQCYGKFDDGGEGEDSFE-RQKVQVSRTP--KHGSWS
Pp3c19_20580V1.1                                  (1) ------------------------------------------------------------------
Pp3c21_15330V1.1                                  (1) ------------------------------------------------------------------
AT1G50700 calcium-dependent protein kinase 33     (1) ---------------------------MGNCLAKKYGLVMKPQQNG---ERSVEIENRRRS-----
AT3G20410 calmodulin-domain protein kinase 9      (1) ---------------------------MGNCFAKNHGLMK-PQQNGNT-TRSVEVGVTNQDPPSYT
AT1G61950 calcium-dependent protein kinase 19     (1) ----------------------------MGCLCINLKKKVKKPTPDISGEQNTEVKSREITPKEQ-
AT4G04695 calcium-dependent protein kinase 31     (1) ----------------------------MGCYSSK-------------------------------
AT4G04700 calcium-dependent protein kinase 27     (1) ----------------------------MGCFSSK-------------------------------
AT4G04710 calcium-dependent protein kinase 22     (1) ---------------------------MGNCCGSKP------------------------------
AT4G04720 calcium-dependent protein kinase 21     (1) ----------------------------MGCFSSKHRKTQNDGG-----EKSIPINPVQTHVV---
AT4G04740 calcium-dependent protein kinase 23     (1) ----------------------------MGCFSSKHRKTQNDGGG----ERSIPIIPVQTHIVDQV
```

```
AT4G21940 calcium-dependent protein kinase 15 (1) ----------------------------MGCFSSKHRNTESDIINGSV-QSSIPTNQPENHVSRDV
AT1G76040 calcium-dependent protein kinase 29 (1) -MLQNQHKTTKNQRNKNIGTKYFLRKKIMGFCFSKFGKSQTHEIPISSSSDSSPPHHYQPLPKPTV
 AT4G23650 calcium-dependent protein kinase 3 (1) ----------------------------MGHRHSKSKSSDPPPSSSSSSSGNV-VHHVKPAGERRG
AT5G12180 calcium-dependent protein kinase 17 (1) ----------------------------MGNCCSHGRDSADNGDALENG------ASASNAANSTG
AT5G19360 calcium-dependent protein kinase 34 (1) ----------------------------MGNCCSHGRDSDDN---KEE-------PRPENGGGGVG
                           Pp3c12_21850V1.1 (1) ----------------------------MGNISGRPRKNRGHGTKGMNLGRPTDPRQKNESERPMY
                             Pp3c4_7390V1.1 (1) ----------------------------MGNVSGRQSKNRGQ-QGGMSQEGSADTRFKSESERPRA
                            Pp3c3_37890V1.1 (1) ----------------------------MGNTSSRGSR--KSTRQVNQGVGSQDTREKNDSVNPKT
                              Pp3c8_690V1.1 (1) ----------------------------MGNTSARPRD--GRKHKASQ--------G-NDSVQPKS
                           Pp3c12_21880V1.1 (1) ----------------------------MGNSSGRPRDSRKGSGGGSQGGSSQGSYPRNEGSYPRG
                            Pp3c9_21410V1.1 (1) ----------------------------MGNQCVGAIGGPYKQEKAHHHVG----HGRDGGVRGVG
                      Pp3c11_25550V1.1 HIP8 (1) ------------------------------------------------------------------
                             Pp3c7_2000V1.1 (1) ------------------------------------------------------------------
                            Pp3c6_50V1.1 P2 (1) ------------------------------------------------------------------
                             Pp3c12_190V1.1 (1) MGNTCVGAAG-----------------YFQGFTSAIALGG---RSSRSNSERSPTASKIDDSDRKV
                            Pp3c17_2480V1.1 (1) MGNTCIGAAG-----------------YFQGFSAAIALGG---RSFTSNSE-ISPSAKADGDRHKA
                            Pp3c20_4100V1.1 (1) MGNTCVGAAS-----------------KAGFFEFPQEGGSPSPSVTPKGTQPEEPAAKPNNPSPEE
                            Pp3c20_4170V1.1 (1) MGNTCVGAAS-----------------KAGFFEFPQERGSPSPSVTPKGTQPEEPAAKPNNPSPEE
                           Pp3c23_18930V1.1 (1) ------------------------------------------------------------------
                           Pp3c23_18880V1.1 (1) ------------------------------------------------------------------
                           Pp3c20_12010V1.1 (1) ------------------------------------------------------------------
 AT2G17290 calcium dependent protein kinase 6 (1) -------------------------------------------MGNSCRGSFKDKIYEGNHSRPEE
 AT4G35310 calmodulin-domain protein kinase 5 (1) -------------------------------------------MGNSCRGSFKDKLDEGDNNKPED
AT4G38230 calcium-dependent protein kinase 26 (1) -------------------------------------------MGLALFSSDGKLIWKGS------
AT1G35670 calcium-dependent protein kinase 11 (1) ------------------------------------------------------------------
 AT4G09570 calcium-dependent protein kinase 4 (1) ------------------------------------------------------------------
AT5G23580 calcium-dependent protein kinase 12 (1) ------------------------------------------------------------------
AT2G35890 calcium-dependent protein kinase 25 (1) MGNVCVHMVN-----------------NCVDTKSNS-----WVRPTDLIMD--HPLKPQLQDKPPQ
AT2G38910 calcium-dependent protein kinase 20 (1) MGNTCVGPNLNPNGFLQSVSAAVWRNQKPDDSIKSS----KDESSRKKN---DKSVNGDDSNGHVS
 AT3G10660 calcium-dependent protein kinase 2 (1) MGNACVGPNISGNGFLQTVTAAMWRPRIGAEQASSSSHGNGQVSKEAAS----EPATDQVQNKPPE
 AT5G04870 calcium dependent protein kinase 1 (1) MGNTCVGPSR--NGFLQSVSAAMWRPRDGDDSASMS---NGDIASEAVSGELRSRLSDEVQNKPPE
                                  Consensus (1)                             MG
```

```
                                                   (67) 67        80        90        100       110       120         132
AT1G18890 calcium-dependent protein kinase 10      (38) TR----------------------------------------------------------------
AT1G74740 calcium-dependent protein kinase 30      (38) LR----------------------------------------------------------------
AT3G51850 calcium-dependent protein kinase 13      (33) GK----------------------------------------------------------------
AT2G41860 calcium-dependent protein kinase 14      (37) ------------------------------------------------------------------
AT3G57530 calcium-dependent protein kinase 32      (39) G-----------------------------------------------------------------
AT5G12480 calmodulin-domain protein kinase 7       (38) ------------------------------------------------------------------
AT5G19450, calcium-dependent protein kinase 8      (36) ------------------------------------------------------------------
Pp3c11_4640V1.1                                    (30) ------------------------------------------------------------------
Pp3c7_22440V1.1                                    (30) ------------------------------------------------------------------
Pp3c11_5760V1.1                                    (33) ------------------------------------------------------------------
Pp3c11_5820V1.1                                    (32) ------------------------------------------------------------------
Pp3c7_22710V1.1                                    (33) ------------------------------------------------------------------
Pp3c7_25180V1.1                                    (32) ------------------------------------------------------------------
AT2G31500 calcium-dependent protein kinase 24      (39) TN----------------------------------------------------------------
AT2G17890 calcium-dependent protein kinase 16      (35) PRSPCSFM---------------AVTIQKDHRTQPR------------------------------
AT4G36070 calcium-dependent protein kinase 18      (34) VSN---------------------------------------------------------------
AT5G66210 calcium-dependent protein kinase 28      (30) PID---------------------------------------------------------------
Pp3c15_3590V1.1                                    (35) HRHQQQQHHY----QHHPHQNGTTNHTQRSREQPP--VVMHVQHRAKDVD---------------
Pp3c9_4620V1.1                                     (33) QTHQQKP-------------QNGATHTKPRSRKPPP--GVVHSRNRSKKVE---------------
Pp3c15_5120V1.1                                    (34) NKGRDLPV------------KVGGDPPEKKERDSPEHKGRDLVENTQGSPP---------------
Pp3c9_5860V1.1                                     (34) N----------------------GE-----------------------------------------
Pp3c13_4100V1.1                                    (37) NSNRASFNNGG-ASPMRQKTPFGSSHPSPRHPSASPLPPYASSPAPSTPRR---------------
Pp3c3_5990V1.1                                     (37) NSNRGSFNNGG-ASPMRHKASFGSSHPSPRHPSASPLPLYATSPTPSTPRR---------------
Pp3c3_5970V1.1                                     (35) S--KGSFNNGG-TSPMRQKTSFRSSQPSPRHPSASPLPQYASSPASTTPRR---------------
Pp3c26_4540V1.1                                    (37) NGNPGSSHNGG-ASPMRARTSFGSSHPSPRHPSGSPLPHYASSPAPSTPGRS--------------
Pp3c4_25010V1.1                                    (36) NSNRGSFNNGGGASPMRAKTSFGSSHPSPRHPSASPLPHYTSSPAPSTPRRN--------------
Pp3c19_20580V1.1                                   (1)  ------------------------------------------------------------------
Pp3c21_15330V1.1                                   (1)  ------------------------------------------------------------------
AT1G50700 calcium-dependent protein kinase 33      (32) ----THQDPSKIS-TGTNQPPP--------------------------------------------
AT3G20410 calmodulin-domain protein kinase 9       (38) PQARTTQQPEKPG-SVNSQPPP--------------------------------------------
AT1G61950 calcium-dependent protein kinase 19      (38) PRQRQPAPRAKFQIVVQPHKLPLPLP----------------------------------------
AT4G04695 calcium-dependent protein kinase 31      (8)  ------------------------------------------------------------------
AT4G04700 calcium-dependent protein kinase 27      (8)  ------------------------------------------------------------------
AT4G04710 calcium-dependent protein kinase 22      (10) ------------------------------------------------------------------
AT4G04720 calcium-dependent protein kinase 21      (31) PEHRKPQTPTPKPMTQPIHQQ---------------------------------------------
AT4G04740 calcium-dependent protein kinase 23      (35) PDHRKPQIPSP-------------------------------------------------------
```

| | | |
|---|---|---|
| AT4G21940 calcium-dependent protein kinase 15 | (38) | LKPQKPPSPQIPTTTQSNHHHQQESKPV-------------------------------------- |
| AT1G76040 calcium-dependent protein kinase 29 | (66) | SQGQTSNPTSNPQPKPKPAPPP-------------------------------------------- |
| AT4G23650 calcium-dependent protein kinase 3 | (38) | S-------SGSGTVGSSGSGTGGS------------------------------------------ |
| AT5G12180 calcium-dependent protein kinase 17 | (33) | P-------TAEASVPQSKHAPPSP------------------------------------------ |
| AT5G19360 calcium-dependent protein kinase 34 | (29) | --------AAEASVRASKHPPASP------------------------------------------ |
| Pp3c12_21850V1.1 | (39) | EGSTQRSSAVASSYGGGTQHKQDG------------------------------------------ |
| Pp3c4_7390V1.1 | (38) | EGSNQRSSATSSSRGASTHPNQGGSARPASTGSAHTGSAHNASTHHNQGGSTRPASTGSTHTGSSH |
| Pp3c3_37890V1.1 | (37) | R--------QGGSVGANNYGGKPS------------------------------------------ |
| Pp3c8_690V1.1 | (28) | H--------QGSSHGDSGHPGGSH------------------------------------------ |
| Pp3c12_21880V1.1 | (39) | SGHGGSTQPRGCNQGGSTQPRGSN------------------------------------------ |
| Pp3c9_21410V1.1 | (35) | HG-------QGQNQQHYRQQNVQE------------------------------------------ |
| Pp3c11_25550V1.1 HIP8 | (1) | ------------------------------------------------------------------ |
| Pp3c7_2000V1.1 | (1) | ------------------------------------------------------------------ |
| Pp3c6_50V1.1 P2 | (1) | ------------------------------------------------------------------ |
| Pp3c12_190V1.1 | (47) | EVDTPATQQNPPRQNHIPSVDTADQQQF------------KEVIEAMK------------------ |
| Pp3c17_2480V1.1 | (46) | GGDSTATQQKAPRQNYIPSVEATDQQEF------------RDVIEAMR------------------ |
| Pp3c20_4100V1.1 | (50) | RRKSGAGVEQEARAELKPQLSLTIQAPA------------VIRIAPTVYPS--------------- |
| Pp3c20_4170V1.1 | (50) | RRKSGAGVEQEARAELKPQLSLTIQAPA------------VIRIAPTVYPS--------------- |
| Pp3c23_18930V1.1 | (1) | ------------------------------------------------------------------ |
| Pp3c23_18880V1.1 | (1) | ------------------------------------------------------------------ |
| Pp3c20_12010V1.1 | (1) | --------------------------------------MNPQQSLCSSLFIRGLSLSQGSSCP--- |
| AT2G17290 calcium dependent protein kinase 6 | (24) | NSKSTTTTVSS--VHSP----TTDQDFS------------KQN----------------------- |
| AT4G35310 calmodulin-domain protein kinase 5 | (24) | YSKTSTTNLSSNSDHSPNAADIIAQEFS------------KDNNSNNN------------------ |
| AT4G38230 calcium-dependent protein kinase 26 | (18) | ---TQTGKRR-------------------------------------------------------- |
| AT1G35670 calcium-dependent protein kinase 11 | (1) | ------------------------------------------------------------------ |
| AT4G09570 calcium-dependent protein kinase 4 | (1) | ------------------------------------------------------------------ |
| AT5G23580 calcium-dependent protein kinase 12 | (1) | ------------------------------------------------------------------ |
| AT2G35890 calcium-dependent protein kinase 25 | (43) | PMLMNKDDDKTKLNDTHGDPKLLEGKEK-----------PAQKQTSQGQGG--------------- |
| AT2G38910 calcium-dependent protein kinase 20 | (60) | STVDPAPSTLPTPSTP----------------P------PPVKMANEEPPP--------------- |
| AT3G10660 calcium-dependent protein kinase 2 | (63) | PITMPSSKTNPETKLKPDLEIQPEEKKEKVLAEETKQKVVPEESKQEVPPEESKREVVVQPES--- |
| AT5G04870 calcium dependent protein kinase 1 | (62) | QVTMPKPGTDVETKDR---EIRTESKPE-----------TLEEISLESKPE--------------- |
| Consensus | (67) | |

```
                                            (133) 133    140       150       160       170       180             198
AT1G18890 calcium-dependent protein kinase 10 (40) ------------------------------------------SPAPIRVL-------KDVIPMSNQ
AT1G74740 calcium-dependent protein kinase 30 (40) ------------------------------------------THAPLR-----------VIPMSHQ
AT3G51850 calcium-dependent protein kinase 13 (35) ------------------------------------------KSAPIR-----------VLSDVPK
AT2G41860 calcium-dependent protein kinase 14 (37) ------------------------------------------------LK-------LIVLKEPTG
AT3G57530 calcium-dependent protein kinase 32 (40) ------------------------------------------DGGGRPLK-------LIVLNDPTG
AT5G12480 calmodulin-domain protein kinase 7 (38) --------------------------------------------SGAGFK-------LSVLKDPTG
AT5G19450, calcium-dependent protein kinase 8 (36) --------------------------------------------SGTGFK-------LSVLKDPTG
Pp3c11_4640V1.1 (30) ----------------------------------------------------------QILKNQPK
Pp3c7_22440V1.1 (30) ----------------------------------------------------------QILKNQPK
Pp3c11_5760V1.1 (33) ----------------------------------------------------------QILKNQPK
Pp3c11_5820V1.1 (32) ----------------------------------------------------------QILKNQPK
Pp3c7_22710V1.1 (33) ----------------------------------------------------------QILKNQPK
Pp3c7_25180V1.1 (32) ----------------------------------------------------------QILKNQPK
AT2G31500 calcium-dependent protein kinase 24 (41) ------------------------------------------LSRRLIFQP-----PSRVLPEPIG
AT2G17890 calcium-dependent protein kinase 16 (56) --------------------RNATAKKTPTRHTPPHGKVREKVISNNGRRHGETIPYGKRVDFGYA
AT4G36070 calcium-dependent protein kinase 18 (37) -------------------------------------KN-KKNTKKIQLRHQGGIPYGKRIDFGYA
AT5G66210 calcium-dependent protein kinase 28 (33) ----------------------------------------TKASTKRR--TGS-IPCGKRTDFGYS
Pp3c15_3590V1.1 (80) ---------------KLPEPKVPEPRRPPTQPGADPRKKPHVKVGSNVKDNKGTIPLGKRTNFGYE
Pp3c9_4620V1.1 (69) ---------------KLAESKQPEPWKSPPESTADRKKKPRVRAAINGKDNKEMAPLGKRTNFGYG
Pp3c15_5120V1.1 (73) ---------------EKKARETPDKQVGATKRPIPERKQSRISAANLKDNQSHSQPLGKRTNFGYE
Pp3c9_5860V1.1 (37) -------------------RHPPAKKIDPAKRPVPERKQSRIPAEHLKNNQSHSQPLGKRTNFGYE
Pp3c13_4100V1.1 (87) ---------------FFKRPFPPPSPAKHIQSSLVKRHGAKPKEAGATL-ESVDNEKPLDKHFGYP
Pp3c3_5990V1.1 (87) ---------------FFKRPFPPPSPAKHIQSSLVKRHGAKPKDGGSDP-ESLDNEKPLDKHFRYP
Pp3c3_5970V1.1 (83) ---------------FFKRPFPPPSPAKHIQSSLVKRHGAKPKEGGSVP-ESVENEKPLDKHFRYS
Pp3c26_4540V1.1 (88) ---------------TFKKPFPPPSPAKHIQSSLVKRHGAKPKEGGVIPAEVADGETSLDKHFHFP
Pp3c4_25010V1.1 (88) ---------------IFKRPFPPPSPAKHIQSSLVKRHGAKPKEGGAIP-EAVDGEKPLDKHFGYH
Pp3c19_20580V1.1 (1) ------------------------------------------------------------MSEYGR
Pp3c21_15330V1.1 (1) -----------------------------------------------------------MSDPYGR
AT1G50700 calcium-dependent protein kinase 33 (49) ----------------------------------WRNPAKHSGAA--------------AILEKPY
AT3G20410 calmodulin-domain protein kinase 9 (59) ----------------------------------WRAAAAAPGLSPKTTT------KSNSILENAF
AT1G61950 calcium-dependent protein kinase 19 (64) --------------------------------QPQEKQKLINHQKQSTLQ------QPEPILGRPF
AT4G04695 calcium-dependent protein kinase 31 (8) ----------------------------------------------NLKQ------SKRTILEKPF
AT4G04700 calcium-dependent protein kinase 27 (8) ----------------------------------------------ELQQ------SKRTILEKPL
AT4G04710 calcium-dependent protein kinase 22 (10) ----------------------------------------LTASDIVSDQ------KQETILGKPL
AT4G04720 calcium-dependent protein kinase 21 (52) --------------------------------------ISTPSSNPVSVR------DPDTILGKPF
AT4G04740 calcium-dependent protein kinase 23 (46) -------------------------------------------SIPISVR------DPETILGKPF
```

```
AT4G21940 calcium-dependent protein kinase 15  (66) ------------------------------NQQIEKKHVLTQPLKPIVFR------ETETILGKPF
AT1G76040 calcium-dependent protein kinase 29  (88) ------------------------------------------PPSTSSGS------QIGPILNRPM
AT4G23650 calcium-dependent protein kinase 3   (55) ------------------------------------------RSTTSTQQ-----NGR--ILGRPM
AT5G12180 calcium-dependent protein kinase 17  (50) ------------------------------------------PPATKQGP-----IGP--VLGRPM
AT5G19360 calcium-dependent protein kinase 34  (45) ------------------------------------------PPATKQGP-----IGP--VLGRPM
Pp3c12_21850V1.1                               (63) ----------STRPATTGSTHAEGGHAARPSSGATPSERPPGPMSAPMPRPRPTSYSANGVLGKPL
Pp3c4_7390V1.1                                (104) NASTHSNQGGSTRPASTGSSHGESGHSARPSSGTAPAERPRVPMAAPRPR----SVSNVGVLGKPL
Pp3c3_37890V1.1                                (53) ----------------SGAQAGERS---------T-----SAPAALPRPKP--ASRSVSGVLGKPL
Pp3c8_690V1.1                                  (44) ----------------RGGSYGG-----------------SQSRHVPKMK-----SSSTGILGKPL
Pp3c12_21880V1.1                               (63) ----------------QGGSTQPRGGSHGNAGGYR-----QAPKYTPPVPK--PKPVAPGFLGKPL
Pp3c9_21410V1.1                                (52) ----------------RGVETTTTR--------------TAPPVTMPKPRP--VNVAAGTVLGKPL
Pp3c11_25550V1.1 HIP8                           (1) ------------------------------------------MADFEVLVGFQEHVPLPALLQNAT
Pp3c7_2000V1.1                                  (1) ------------------------------------------MANVACLIGSREHLPLPVLLQNTR
Pp3c6_50V1.1 P2                                 (1) ------------------------------------------MAEVALLN-PLEQIHDFAVLQHKS
Pp3c12_190V1.1                                 (83) --------------------------------------------KGREIKSVSGQSLTHSVLQRKT
Pp3c17_2480V1.1                                (82) --------------------------------------------KGREIEPVPGQSLTHSVLQRKT
Pp3c20_4100V1.1                                (89) ------------------------------AANRRQENCVVPAMRRAGLNLIPGLSFTHSVLQRQT
Pp3c20_4170V1.1                                (89) ------------------------------AANRRQENCVVPAMRRAGLNLIPGLSFTHSVLQRQT
Pp3c23_18930V1.1                                (1) --------------------------------------------MRRGVNLVPGQSFTHSVLQRNT
Pp3c23_18880V1.1                                (1) --------------------------------------------MRRGVNLVPGQSFTHSVLQRNT
Pp3c20_12010V1.1                               (26) -----------SPDNTPRVAVEKKPEVKYTTQNSKQVEGVIQAIKASDNKDQKKGKLTVCVLQHVT
AT2G17290 calcium dependent protein kinase 6   (49) ------------------------------------TNPALVIPVKEPIMRRNVDNQSYYVLGHKT
AT4G35310 calmodulin-domain protein kinase 5   (60) -----------------------------------SKDPALVIPLREPIMRRNPDNQAYYVLGHKT
AT4G38230 calcium-dependent protein kinase 26  (25) -------------------------------------------PQEEATMKHSGGNQACYVLGQKT
AT1G35670 calcium-dependent protein kinase 11   (1) -----------------------------------------------METKPNPRRPSNTVLPYQT
AT4G09570 calcium-dependent protein kinase 4    (1) ------------------------------------------------MEKPNPRRPSNSVLPYET
AT5G23580 calcium-dependent protein kinase 12   (1) -----------------------------------------------MANKPR----TRWVLPYKT
AT2G35890 calcium-dependent protein kinase 25  (83) --------------------RK---CSDEEYKKRAIACANSKRKAHNVRRLMSAGLQAESVLKTKT
AT2G38910 calcium-dependent protein kinase 20  (89) --------------------KP-------ITENKEDPNSKPQKKEAHMKRMASAGLQIDSVLGRKT
AT3G10660 calcium-dependent protein kinase 2  (126) -----------AKPETKSESKPETTKPETTSETKPETKAEPQKP-KHMRRVSSAGLRTESVLQRKT
AT5G04870 calcium dependent protein kinase 1   (99) -----------TKQETKSETKP---------ESKPDPPAKPKKP-KHMKRVSSAGLRTESVLQRKT
Consensus                                     (133)                                                             VL
```

Protein kinase / ser/thr / dual specificity protein kinase, catalytic domain

```
                                                (199) 199       210       220       230       240       250            264
AT1G18890 calcium-dependent protein kinase 10    (57) TQISDKYILGRELGRGEFGITYLCTDRE---THEALACKSISKR---------------------K
AT1G74740 calcium-dependent protein kinase 30    (53) SQISDKYILGRELGRGEFGITYLCTDRE---TREALACKSISKR---------------------K
AT3G51850 calcium-dependent protein kinase 13    (48) ENIEDRYLLDRELGRGEFGVTYLCIERS---SRDLLACKSISKR---------------------K
AT2G41860 calcium-dependent protein kinase 14    (48) HEIKQKYKLGRELGRGEFGVTYLCTEIE---TGEIFACKSILKK---------------------K
AT3G57530 calcium-dependent protein kinase 32    (57) REIESKYTLGRELGRGEFGVTYLCTDKE---TDDVFACKSILKK---------------------K
AT5G12480 calmodulin-domain protein kinase 7     (53) HDISLQYDLGREVGRGEFGITYLCTDKE---TGEKYACKSISKK---------------------K
AT5G19450, calcium-dependent protein kinase 8   (51) HDISLMYDLGREVGRGEFGITYLCTDIK---TGEKYACKSISKK---------------------K
Pp3c11_4640V1.1                                  (38) SRILDKYNLGRELGRGEFGITYLCTDKE---TGEVFACKSISKK---------------------K
Pp3c7_22440V1.1                                  (38) AKILDKYILGRELGRGEFGITYLCTDKE---TGETLACKSISKK---------------------K
Pp3c11_5760V1.1                                  (41) ARILDKYVLGRELGRGEFGITYLCTDKE---TQEVFACKSISKK---------------------K
Pp3c11_5820V1.1                                  (40) ARILDKYVLGRELGRGEFGITYLCTDKE---TQEVFACKSISKK---------------------K
Pp3c7_22710V1.1                                  (41) ARILDKYVLGRELGRGEFGITYLCTDKE---TQDVFACKSISKK---------------------K
Pp3c7_25180V1.1                                  (40) ARILDKYVLGRELGRGEFGITYLCTDKE---TQEIFACKSISKK---------------------K
AT2G31500 calcium-dependent protein kinase 24    (60) DGIHLKYDLGKELGRGEFGVTHECIEIS---TRERFACKRISKE---------------------K
AT2G17890 calcium-dependent protein kinase 16   (102) KDFDHRYTIGKLLGHGQFGYTYVATDKK---TGDRVAVKKIDKA---------------------K
AT4G36070 calcium-dependent protein kinase 18    (65) KDFDNRYTIGKLLGHGQFGFTYVATDNN---NGNRVAVKRIDKA---------------------K
AT5G66210 calcium-dependent protein kinase 28    (56) KDFHDHYTIGKLLGHGQFGYTYVAIHRP---NGDRVAVKRLDKS---------------------K
Pp3c15_3590V1.1                                 (131) RDFKSKYTLGKLLGHGQFGYTYVALEKA---TGEKVAVKTIEKK---------------------Q
Pp3c9_4620V1.1                                  (120) RDFKSKYTLGKLLGHGQFGYTYVAIEKS---TGSRVAVKTIEKK---------------------Q
Pp3c15_5120V1.1                                 (124) RDFKEKYSLGKLLGHGQFGYTYVATEKA---TGNKVAAKCIEKK---------------------Q
Pp3c9_5860V1.1                                   (84) RDFKEKYTLGKLLGHGQFGYTYVAIEKS---TGNKVAAKCIEKK---------------------Q
Pp3c13_4100V1.1                                 (137) KNFTNKYELGHEVGRGHFGHTCYAKIKKGDHKGQPVAVKIISKA---------------------K
Pp3c3_5990V1.1                                  (137) KDFMSKYELGHEVGRGHFGHTCYAKIRKGENKGLPVAVKIISKA---------------------K
Pp3c3_5970V1.1                                  (133) KDFMNKYELGHEVGRGHFGHTCYAKIRKGENKGLPVAVKIISKA---------------------K
Pp3c26_4540V1.1                                 (139) KNFNSKYELGHEVGRGHFGHTCYAKMRKGEHKGQAVAVKIISKA---------------------K
Pp3c4_25010V1.1                                 (138) KNFATKYELGHEVGRGHFGHTCYAKVRKGEHKGQAVAVKIISKA---------------------K
Pp3c19_20580V1.1                                  (7) RAVSDDYQLGQVLGTGGFSVVRIGVRKEDR---LQVAIKTLKKYGYGTGKGKRKGKGHAGSEARMW
Pp3c21_15330V1.1                                  (8) RLLVDDFHVGPVLGTGGFSVVRAGVRKQDN---LQVAIKTLKKFGYGRGDHGRPG-------AQMS
AT1G50700 calcium-dependent protein kinase 33    (67) EDVKLFYTLSKELGRGQFGVTYLCTEKS---TGKRFACKSISKK---------------------K
AT3G20410 calmodulin-domain protein kinase 9     (85) EDVKLFYTLGKELGRGQFGVTYLCTENS---TGKKYACKSISKK---------------------K
AT1G61950 calcium-dependent protein kinase 19    (92) EDIKEKYSLGRELGRGQFGITYICTEIS---SGKNFACKSILKR---------------------K
AT4G04695 calcium-dependent protein kinase 31    (22) VDIGKVYILGDELGQGQFGITRKCVEKT---SGKTYACKTILKT---------------------N
AT4G04700 calcium-dependent protein kinase 27    (22) VDITKIYILGEELGRGNFGLTRKCVEKS---TGKTFACKTILKT---------------------K
AT4G04710 calcium-dependent protein kinase 22    (30) EDIKKHYSFGDELGKG---------KS--------YACKSIPKR---------------------T
AT4G04720 calcium-dependent protein kinase 21    (74) EDIRKFYSLGKELGRGQFGITYMCKEIG---TGNTYACKSILKR---------------------K
AT4G04740 calcium-dependent protein kinase 23    (63) EDIRKFYSLGRELGRGGLGITYMCKEIG---TGNIYACKSILKR---------------------K
```

```
AT4G21940 calcium-dependent protein kinase 15  (96) EEIRKLYTLGKELGRGQFGITYTCKENS---TGNTYACKSILKR---------------------K
AT1G76040 calcium-dependent protein kinase 29 (106) IDLSALYDLHKELGRGQFGITYKCTDKS---NGREYACKSISKR---------------------K
AT4G23650 calcium-dependent protein kinase 3   (72) EEVRRTYEFGRELGRGQFGVTYLVTHKE---TKQQVACKSIPTR---------------------R
AT5G12180 calcium-dependent protein kinase 17  (67) EDVKASYSLGKELGRGQFGVTHLCTQKA---TGHQFACKTIAKR---------------------K
AT5G19360 calcium-dependent protein kinase 34  (62) EDVKSSYTLGKELGRGQFGVTHLCTQKA---TGLQFACKTIAKR---------------------K
                          Pp3c12_21850V1.1 (119) VDIRQTYSLGRELGRGQFGVTYLCTHKT---TGEILACKSIAKR---------------------K
                           Pp3c4_7390V1.1 (166) VDIRQTYSLGKELGRGQFGVTYLCTHKE---TGEKLACKSIAKR---------------------K
                          Pp3c3_37890V1.1  (87) SDIRQSYILGRELGRGQFGVTYLCTDKM---TNEAYACKSIAKR---------------------K
                            Pp3c8_690V1.1  (72) RDIKLHYTLGRELGRGQFGVTYLCTDKE---TGISYACKTIAKR---------------------K
                          Pp3c12_21880V1.1 (106) SDILNSYTLGKELGRGEFGVTYTCTHKD---TNEVYACKTIAKR---------------------K
                           Pp3c9_21410V1.1  (86) SDVRSVYTLGKELGRGQFGVTYACTNIK---TGEHLACKSIAKR---------------------K
                    Pp3c11_25550V1.1 HIP8  (25) DDLEKLYSLGKKLGEGQFGTTYLCTERA---TGLQFACKCIPKR---------------------K
                           Pp3c7_2000V1.1  (25) DNFEQIYSVGQKLGAGQFGTPYLYTKRA---TGLEYASKCIPKR---------------------K
                          Pp3c6_50V1.1 P2  (24) EDVKDIYTLGKKLGEGQFGITYLCTEKA---TGLKYACKCIPKR---------------------K
                          Pp3c12_190V1.1 (105) ENLRDLYILGKKLGQGQFGTTYLCIEKA---TNKEYACKSIAKR---------------------K
                         Pp3c17_2480V1.1 (104) ENLRDLYTLGKKLGQGQFGTTYLCIEKA---TSKEYACKSIAKR---------------------K
                          Pp3c20_4100V1.1 (125) ENLKDLYRLERKLGQGQFGTTYLCVEKA---TGREYACKSIAKR---------------------K
                          Pp3c20_4170V1.1 (125) ENLKDLYRLGRKLGQGQFGTTYLCVEKA---TGREYACKSIAKR---------------------K
                         Pp3c23_18930V1.1  (23) ENLKDLYTLGRKLGQGQFGTTYLCVEKT---TGKEYACKSIAKR---------------------K
                         Pp3c23_18880V1.1  (23) ENLKDLYTLGRKLGQGQFGTTYLCVEKT---TGKEYACKSIAKR---------------------K
                         Pp3c20_12010V1.1  (81) EDVRDVYTLGKKLGAGNFGITYLCTEKS---TGDDYACKTISKK---------------------K
 AT2G17290 calcium dependent protein kinase 6  (79) PNIRDLYTLSRKLGQGQFGTTYLCTDIA---TGVDYACKSISKR---------------------K
 AT4G35310 calmodulin-domain protein kinase 5  (91) PNIRDIYTLSRKLGQGQFGTTYLCTEIA---SGVDYACKSISKR---------------------K
AT4G38230 calcium-dependent protein kinase 26  (48) PSIRDLYSLGHKLGQGQFGTTYMCKEIS---TGREYACKSITKR---------------------K
AT1G35670 calcium-dependent protein kinase 11  (20) PRLRDHYLLGKKLGQGQFGTTYLCTEKS---TSANYACKSIPKR---------------------K
 AT4G09570 calcium-dependent protein kinase 4  (19) PRLRDHYLLGKKLGQGQFGTTYLCTEKS---SSANYACKSIPKR---------------------K
AT5G23580 calcium-dependent protein kinase 12  (16) KNVEDNYFLGQVLGQGQFGTTFLCTHKQ---TGQKLACKSIPKR---------------------K
AT2G35890 calcium-dependent protein kinase 25 (126) GHLKEYYNLGSKLGHGQFGTTFVCVEKG---TGEEYACKSIPKR---------------------K
AT2G38910 calcium-dependent protein kinase 20 (128) ENLKDIYSVGRKLGQGQFGTTFLCVDKK---TGKEFACKTIAKR---------------------K
 AT3G10660 calcium-dependent protein kinase 2 (180) ENFKEFYSLGRKLGQGQFGTTFLCLEKG---TGNEYACKSISKR---------------------K
 AT5G04870 calcium dependent protein kinase 1 (144) ENFKEFYSLGRKLGQGQFGTTFLCVEKT---TGKEFACKSIAKR---------------------K
                                Consensus (199)  DIK  YTLGRELGRGQFGITYLCTEK   TG  YACKSISKR                     K
```

Protein kinase / ser/thr / dual specificity protein kinase, catalytic domain

```
                                                      (265) 265  270       280       290       300       310       320       330
AT1G18890 calcium-dependent protein kinase 10          (99) LRTAVDIEDVRREVAIMSTLPEHPNVVKLKASYEDNENVHLVMELCEGGELFDRIVARG----HYT
AT1G74740 calcium-dependent protein kinase 30          (95) LRTAVDVEDVRREVTIMSTLPEHPNVVKLKATYEDNENVHLVMELCEGGELFDRIVARG----HYT
AT3G51850 calcium-dependent protein kinase 13          (90) LRTAVDIEDVKREVAIMKHLPKSSSIVTLKEACEDDNAVHLVMELCEGGELFDRIVARG----HYT
AT2G41860 calcium-dependent protein kinase 14          (90) LKTSIDIEDVKREVEIMRQMPEHPNIVTLKETYEDDKAVHLVMELCEGGELFDRIVARG----HYT
AT3G57530 calcium-dependent protein kinase 32          (99) LRTAVDIEDVRREVEIMRHMPEHPNVVTLKETYEDEHAVHLVMELCEGGELFDRIVARG----HYT
AT5G12480 calmodulin-domain protein kinase 7           (95) LRTAVDIEDVRREVEIMKHMPKHPNVVSLKDSFEDDDAVHIVMELCEGGELFDRIVARG----HYT
AT5G19450, calcium-dependent protein kinase 8         (93) LRTAVDIEDVRREVEIMKHMPRHPNIVSLKDAFEDDDAVHIVMELCEGGELFDRIVARG----HYT
Pp3c11_4640V1.1                                       (80) LRTAVDVEDVRREVAIMKHLLEHPNIVTLKGAYEDDNAVHLVMELCEGGELFDRIIARG----HYT
Pp3c7_22440V1.1                                       (80) LRTAVDVEDVRREVAIMKHLPEHPNIVTLNGAFEDDNAVHLVMELCEGGELFDRIIARG----HYT
Pp3c11_5760V1.1                                       (83) LRTAVDVEDVRREVAIMKHLPHHPHIVTLEGAYEDDVAVHLVMELCEGGELFDRIIARG----HYT
Pp3c11_5820V1.1                                       (82) LRTAVDVEDVRREVAIMKHLPHHPHIVTLEGAYEDDVAVHLVMELCEGGELFDRIIARG----HYT
Pp3c7_22710V1.1                                       (83) LRTAVDVEDVRREFAIMKHLPEHPHVVTLKGAYEDDNAVHLVMELCEGGELFDRIIARG----HYT
Pp3c7_25180V1.1                                       (82) LRTAVDVEDVRREVAIMKHLPEHPHVVTLKGAYEDDNAVHLVMELCEGGELFDRIIARG----HYT
AT2G31500 calcium-dependent protein kinase 24        (102) LRTEIDVEDVRREVEIMRCLPKHPNIVSFKEAFEDKDAVYLVMEICEGGELFDRIVSRG----HYT
AT2G17890 calcium-dependent protein kinase 16        (144) MTIPIAVEDVKREVKILQALTGHENVVRFYNAFEDKNSVYIVMELCEGGELLDRILARK--DSRYS
AT4G36070 calcium-dependent protein kinase 18        (107) MTQPIEVEDVKREVKILQALGGHENVVGFHNAFEDKTYIYIVMELCDGGELLDRILAKK--DSRYT
AT5G66210 calcium-dependent protein kinase 28         (98) MVLPIAVEDVKREVQILIALSGHENVVQFHNAFEDDDYVYIVMELCEGGELLDRILSKK--GNRYS
Pp3c15_3590V1.1                                      (173) MTLQISVDDVKREVKILRTLSGHENVVQFYASFEDDDLVYIVMELCEGGELLDRILAKK--DSRYS
Pp3c9_4620V1.1                                       (162) MTLPISVEDVKREVKILRTLSGHENVVQFYASFEDDDLVYIVMELCEGGELLDRILAKK--DSRYS
Pp3c15_5120V1.1                                      (166) MKLPISVEDVKREVKILRTLSGHENVVQFFAAFEDDDLVYIVMELCEGGELLDRILAKK--DTRYS
Pp3c9_5860V1.1                                       (126) MTLPISVEDVKREVKILRTLSGHENVVQFYAAFEDDDLVYIVMELCEGGELLDRILAKK--DSRYS
Pp3c13_4100V1.1                                      (182) MTTAIAIEDVRREVKILKALTGHHNLVRFYDAREDALNVYIVMELCEGGELLDRILTR---GGRYT
Pp3c3_5990V1.1                                       (182) MTTAIAIEDVRREVKILKALTGHHNLVWFYDACEDDMNVYIVMELCEGGELLDRILSR---GGRYT
Pp3c3_5970V1.1                                       (178) MTTAIAIEDVQREVKILKALTGHHNLVRFYDACEDGLNVYIVMELCEGGELLDRILSR---GGRYT
Pp3c26_4540V1.1                                      (184) MTTAIAIEDVRREVRILKALTGHHNLVRFYDACEDNVNVYIVMELCEGGELLDRILSR---GGKYS
Pp3c4_25010V1.1                                      (183) MTTAIAIEDVGREVKILKALTGHQNLVRFYDSCEDHLNVYIVMELCEGGELLDRILSR---GGKYS
Pp3c19_20580V1.1                                      (70) QAEALVTNEILVMMRIVDYVSPHPNIIHLLDVYEDDDAVHLVLELCRGGELFDRIVEQE----RYS
Pp3c21_15330V1.1                                      (64) QAEALVKNEIMVMMRIVDEVSPHPNVIHLIDVYEDDGAVHLVLELCRGGELFDRIVQHE----RYS
AT1G50700 calcium-dependent protein kinase 33        (109) LVTKGDKEDMRREIQIMQHLSGQPNIVEFKGAYEDEKAVNLVMELCAGGELFDRILAKG----HYS
AT3G20410 calmodulin-domain protein kinase 9         (127) LVTKADKDDMRREIQIMQHLSGQPNIVEFKGAYEDEKAVNLVMELCAGGELFDRIIAKG----HYT
AT1G61950 calcium-dependent protein kinase 19        (134) LIRTKDREDVRREIQIMHYLSGQPNIVEIKGAYEDRQSVHLVMELCEGGELFDKITKRG----HYS
AT4G04695 calcium-dependent protein kinase 31         (64) LKSREDEEAVKREIRIMKHLSGEPNIVEFKKAYEDRDSVHIVMEYCGGGELFKKIEALSKDGKSYS
AT4G04700 calcium-dependent protein kinase 27         (64) LKDEECEEDVKREIRIMKQLSGEPNIVEFKNAYEDKDSVHIVMEYCGGGELYDKILALYDVGKSYS
AT4G04710 calcium-dependent protein kinase 22         (58) LSSEEEKEAVKTEIQIMDHVSGQPNIVQIKGSYEDNNSIHIVMELCGGGELFDKIDALVKSHSYYS
AT4G04720 calcium-dependent protein kinase 21        (116) LISKQDKEDVKREIQIMQYLSGQPNIVEIKGAYEDRQSIHLVMELCAGGELFDRIIAQG----HYS
AT4G04740 calcium-dependent protein kinase 23        (105) LISELGREDVKTEIQIMQHLSGQPNVVEIKGSYEDRHSVHLVMELCAGGELFDRIIAQG----HYS
```

| | |
|---|---|
| AT4G21940 calcium-dependent protein kinase 15 (138) | LTRKQDIDDVKREIQIMQYLSGQENIVEIKGAYEDRQSIHLVMELCGGSELFDRIIAQG----HYS |
| AT1G76040 calcium-dependent protein kinase 29 (148) | LIRRKDIEDVRREVMILQHLTGQPNIVEFRGAYEDKDNLHLVMELCSGGELFDRIIKKG----SYS |
| AT4G23650 calcium-dependent protein kinase 3 (114) | LVHKDDIEDVRREVQIMHHLSGHRNIVDLKGAYEDRHSVNLIMELCEGGELFDRIISKG----LYS |
| AT5G12180 calcium-dependent protein kinase 17 (109) | LVNKEDIEDVRREVQIMHHLTGQPNIVELKGAYEDKHSVHLVMELCAGGELFDRIIAKG----HYS |
| AT5G19360 calcium-dependent protein kinase 34 (104) | LVNKEDIEDVRREVQIMHHLTGQPNIVELKGAYEDKHSVHLVMELCAGGELFDRIIAKG----HYS |
| Pp3c12_21850V1.1 (161) | LTTKEDVEDVKREVQIMHHLSGTPNIVDLKGVYEDRHSVHLVMELCAGGELFDRIIAKG----HYS |
| Pp3c4_7390V1.1 (208) | LIAKEDIEDVKREVQIMHHLSGTPNIVDLKGVYEDRHSVHLVMELCAGGELFDRIIAKG----HYS |
| Pp3c3_37890V1.1 (129) | LTSKEDIEDVKREVQIMHHLSGTPNIVVLKDVFEDKHSVHLVMELCAGGELFDRIIAKG----HYS |
| Pp3c8_690V1.1 (114) | LTNKDDIEDVKREVQIMHHLSGTPNIVELKDVFEDKQNVNLVMELCAGGELFDRIIAKG----HYS |
| Pp3c12_21880V1.1 (148) | LTHKDDIEDVKREVQIMHHLSGTLNIVTLKAVFEDKHNIHLVMELCAGGELFDRIVAKK----CYS |
| Pp3c9_21410V1.1 (128) | LISKEDIEDVRREVQIMHHLSGTPNVVELKGVFEDKHHVHIVMELCAGGELFDRIIAKG----HYS |
| Pp3c11_25550V1.1 HIP8 (67) | LISSEEIEDVGREVEVMYHLSGHPNIVTLKGAYEDATNVYLVMELCEGGELFDRIIERG----TYT |
| Pp3c7_2000V1.1 (67) | LISLEEIEDVGREVEVMYHLSGHP------------------------------------------ |
| Pp3c6_50V1.1 P2 (66) | LISEEEIENVGREIEVMYHLAGHPNIVAIKGAYEDETMVYLVMELCEGGELFDRIIERG----TYT |
| Pp3c12_190V1.1 (147) | LISKEDVEDVRRELQIMHHLSGHPNIVMIKGAYEDPASVHLVMELCAGGELFDRIIQRG----QYS |
| Pp3c17_2480V1.1 (146) | LISKEDVEDVRRELQIMHHLSGHPNIVMIKGAYEDPASVHLVMELCAGGELFDRIIQRG----HYS |
| Pp3c20_4100V1.1 (167) | LISQEDVDDVRRELHIMHHLSGHPNIVTIKGAYEDQMAVHLVMELCAGGELFDRIIQRG----HYS |
| Pp3c20_4170V1.1 (167) | LISQEDVDDVRRELHIMHHLSGHPNIVTIKGAYEDQMAVHLVMELCAGGELFDRIIQRG----HYS |
| Pp3c23_18930V1.1 (65) | LISQEDVDDVRRELHIMHHLSGHPNIVTIKGAYEDQVSVHLVMELCAGGELFDRIIQRG----HYS |
| Pp3c23_18880V1.1 (65) | LISQEDVDDVRRELHIMHHLSGHPNIVTIKGAYEDQVSVHLVMELCAGGELFDRIIQRG----HYS |
| Pp3c20_12010V1.1 (123) | LKSKDDVADVRKELQIMHHLSGHPNIVAIKGAFEDEASVHFVMELCAGGELFTRITEKG----HYS |
| AT2G17290 calcium dependent protein kinase 6 (121) | LISKEDVEDVRREIQIMHHLAGHKNIVTIKGAYEDPLYVHIVMELCAGGELFDRIIHRG----HYS |
| AT4G35310 calmodulin-domain protein kinase 5 (133) | LISKEDVEDVRREIQIMHHLAGHGSIVTIKGAYEDSLYVHIVMELCAGGELFDRIIQRG----HYS |
| AT4G38230 calcium-dependent protein kinase 26 (90) | LISKEDVEDVRREIQIMHHLAGYKNIVTIKGAYEDPLYVHIVMELCSGGELFDRIIQRG----HYS |
| AT1G35670 calcium-dependent protein kinase 11 (62) | LVCREDYEDVWREIQIMHHLSEHPNVVRIKGTYEDSVFVHIVMEVCEGGELFDRIVSKG----HFS |
| AT4G09570 calcium-dependent protein kinase 4 (61) | LVCREDYEDVWREIQIMHHLSEHPNVVRIKGTYEDSVFVHIVMEVCEGGELFDRIVSKG----CFS |
| AT5G23580 calcium-dependent protein kinase 12 (58) | LLCQEDYDDVLREIQIMHHLSEYPNVVRIESAYEDTKNVHLVMELCEGGELFDRIVKRG----HYS |
| AT2G35890 calcium-dependent protein kinase 25 (168) | LENEEDVEDVRREIEIMKHLLGQPNVISIKGAYEDSVAVHMVMELCRGGELFDRIVERG----HYS |
| AT2G38910 calcium-dependent protein kinase 20 (170) | LTTPEDVEDVRREIQIMHHLSGHPNVIQIVGAYEDAVAVHVVMEICAGGELFDRIIQRG----HYT |
| AT3G10660 calcium-dependent protein kinase 2 (222) | LLTDEDVEDVRREIQIMHHLAGHPNVISIKGAYEDVVAVHLVMELCSGGELFDRIIQRG----HYT |
| AT5G04870 calcium dependent protein kinase 1 (186) | LLTDEDVEDVRREIQIMHHLAGHPNVISIKGAYEDVVAVHLVMECCAGGELFDRIIQRG----HYT |
| Consensus (265) | L T  DVEDVRREV IM HLSGHPNIV LKGAYED  AVHLVMELC GGELFDRIIARG    HYS |

Protein kinase / ser/thr / dual specificity protein kinase, catalytic domain

| | 331 340 350 360 370 380 396 |
|---|---|
| (331) | |
| AT1G18890 calcium-dependent protein kinase 10 (161) | ERAAAAVARTIAEVVMMCHSNGVMHRDLKPENFLFANKKEN-SPLKAIDFGLSVFFKPGDKFTEIV |
| AT1G74740 calcium-dependent protein kinase 30 (157) | ERAAATVARTIAEVVRMCHVNGVMHRDLKPENFLFANKKEN-SALKAIDFGLSVLFKPGERFTEIV |
| AT3G51850 calcium-dependent protein kinase 13 (152) | ERAAAGVTKTIVEVVQLCHKHGVIHRDLKPENFLFANKKEN-SPLKAIDFGLSIFFKPGEKFSEIV |
| AT2G41860 calcium-dependent protein kinase 14 (152) | ERAAASVIKTIIEVVQMCHKHGVMHRDLKPENFLFANKKET-ASLKAIDFGLSVFFKPGDRFNEIV |
| AT3G57530 calcium-dependent protein kinase 32 (161) | ERAAAAVTKTIMEVVQVCHKHGVMHRDLKPENFLFGNKKET-APLKAIDFGLSVFFKPGERFNEIV |
| AT5G12480 calmodulin-domain protein kinase 7 (157) | ERAAAAVMKTIVEVVQICHKQGVMHRDLKPENFLFANKKET-SALKAIDFGLSVFFKPGEQFNEIV |
| AT5G19450, calcium-dependent protein kinase 8 (155) | ERAAAAVMKTILEVVQICHKHGVMHRDLKPENFLFANKKET-SALKAIDFGLSVFFKPGEGFNEIV |
| Pp3c11_4640V1.1 (142) | ERGAAQVTRTIVEVVQACHRQGVMHRDLKPENFLFANKNEN-SPLKAIDFGLSVFFKPGERFSEIV |
| Pp3c7_22440V1.1 (142) | ERGAAQVTRTIVEVVQACHIQGVIHRDLKPENFLFANKNEN-SVLKAIDFGLSVFFKPGEKFSEIV |
| Pp3c11_5760V1.1 (145) | ERGAAQVTRTIVEVVQACHRQGVIHRDLKPENFLFANTNEN-APLKAIDFGLSVFFKPGERFSEIV |
| Pp3c11_5820V1.1 (144) | ERGAAQVTRTIVEVVQACHRQGVIHRDLKPENFLFANTNEN-APLKAIDFGLSVFFKPGERFSEIV |
| Pp3c7_22710V1.1 (145) | ERGAAQVTRTIMEVVQACHRQGVMHRDLKPENFLFANKDEN-SPLKAIDFGLSVFFKPGEKFSEIV |
| Pp3c7_25180V1.1 (144) | ERGAAQVTRTIVEVVQACHRQGVMHRDLKPENFLFANKNEN-SPLKAIDFGLSVFFKPGEKFSEIV |
| AT2G31500 calcium-dependent protein kinase 24 (164) | ERAAASVAKTILEVVKVCHEHGVIHRDLKPENFLFSNGTET-AQLKAIDFGLSIFFKPAQRFNEIV |
| AT2G17890 calcium-dependent protein kinase 16 (208) | ERDAAVVVRQMLKVAAECHLRGLVHRDMKPENFLFKSTEED-SPLKATDFGLSDFIKPGKKFHDIV |
| AT4G36070 calcium-dependent protein kinase 18 (171) | EKDAAVVVRQMLKVAAECHLRGLVHRDMKPENFLFKSTEEG-SSLKATDFGLSDFIKPGVKFQDIV |
| AT5G66210 calcium-dependent protein kinase 28 (162) | EKDAAVVVRQMLKVAGECHLHGLVHRDMKPENFLFKSAQLD-SPLKATDFGLSDFIKPGKRFHDIV |
| Pp3c15_3590V1.1 (237) | EKDAAKIVRQMLNVAARCHLNGVVHRDMKPENFLFKSPKED-SPLKATDFGLSDYIQPGKRFQDVV |
| Pp3c9_4620V1.1 (226) | EKDAAKIVRQMLNVAARCHLNGVVHRDMKPENFLFKSTSED-SPLKATDFGLSDYIRPGNRFHDVV |
| Pp3c15_5120V1.1 (230) | EKDAAKIVRQMLNVAARCHLNGVVHRDMKPENFLFKSSKDD-SPLKATDFGLSDYIKPGKRFRDVV |
| Pp3c9_5860V1.1 (190) | EKDAAKIVRQMLNVAARCHLNGVVHRDMKPENFLFKSPKED-SPLKATDFGLSDYIKPGKRFGDVV |
| Pp3c13_4100V1.1 (245) | EDDAKIVVQQILSIVAFCHLQGVVHRDLKPENFLFTTKDEY-AQLKAIDFGLSDFIKPDERLNDIV |
| Pp3c3_5990V1.1 (245) | EEDAKIVVQQILSIVAFCHLQGVVHRDLKPENFLFTTKDEH-AQLKAIDFGLSDFIKPDERLNDIV |
| Pp3c3_5970V1.1 (241) | EEDAKIVVRQILSIVAFCHLQGVVHRDLKPENFLFTSKEEH-AQLKAIDFGLSDFIKPDERLNDIV |
| Pp3c26_4540V1.1 (247) | EEDAKIVLRQILSVVAFCHLQGVVHRDLKPENFLFTTKDEH-AQLKAIDFGLSDFIKPDERLNDIV |
| Pp3c4_25010V1.1 (246) | EEDAKVVVRQILSVVAFCHLQGVVHRDLKPENFLFTTKDEY-AQLKAIDFGLSDFIKPDERLNDIV |
| Pp3c19_20580V1.1 (132) | ERDAATVVGQIAAGLAALHHCGIVHRDLKPENCLYADPHPE-APLKIMDFGLSHIHNVTNAVVGVF |
| Pp3c21_15330V1.1 (126) | ERDAAKVVRQIASGLAALHQAQIVHRDLKPENCLYVNPLAE-APLKIMDFGLSYIHHNTNSIVGIF |
| AT1G50700 calcium-dependent protein kinase 33 (171) | ERAAASVCRQIVNVVNICHFMGVMHRDLKPENFLLSSKDEK-ALIKATDFGLSVFIEEGRVYKDIV |
| AT3G20410 calmodulin-domain protein kinase 9 (189) | ERAAASVCRQIVNVVKICHFMGVLHRDLKPENFLLSSKDEK-ALIKATDFGLSVFIEEGKVYRDIV |
| AT1G61950 calcium-dependent protein kinase 19 (196) | EKAAAEIIRSVVKVVQICHFMGVIHRDLKPENFLLSSKDEASSMLKATDFGVSVFIEEGKVYEDIV |
| AT4G04695 calcium-dependent protein kinase 31 (130) | EKEAVEIIRPIVNVVKNCHYMGVMLRDLKPENFLLSSTDKN-ATVKAIDFGCSVFIEEGEVHRKFA |
| AT4G04700 calcium-dependent protein kinase 27 (130) | EKEAAGIIRSIVNVVKNCHYMGVMHRDLKPENFLLTSNDDN-ATVKVIDFGCSVFIEEGKVYQDLA |
| AT4G04710 calcium-dependent protein kinase 22 (124) | EKDAAGIFRSIVNAVKICHSLDVVHRDLKPENFLFSSKDEN-AMLKAIDFGCSVYIKEGKTFERVV |
| AT4G04720 calcium-dependent protein kinase 21 (178) | ERAAAGIIRSIVNVVQICHFMGVVHRDLKPENFLLSSKEEN-AMLKATDFGLSVFIEEGKVYRDIV |
| AT4G04740 calcium-dependent protein kinase 23 (167) | ERAAAGTIKSIVDVVQICHLNGVIHRDLKPENFLFSSKEEN-AMLKVTDFGLSAFIEEGKIYKDVV |

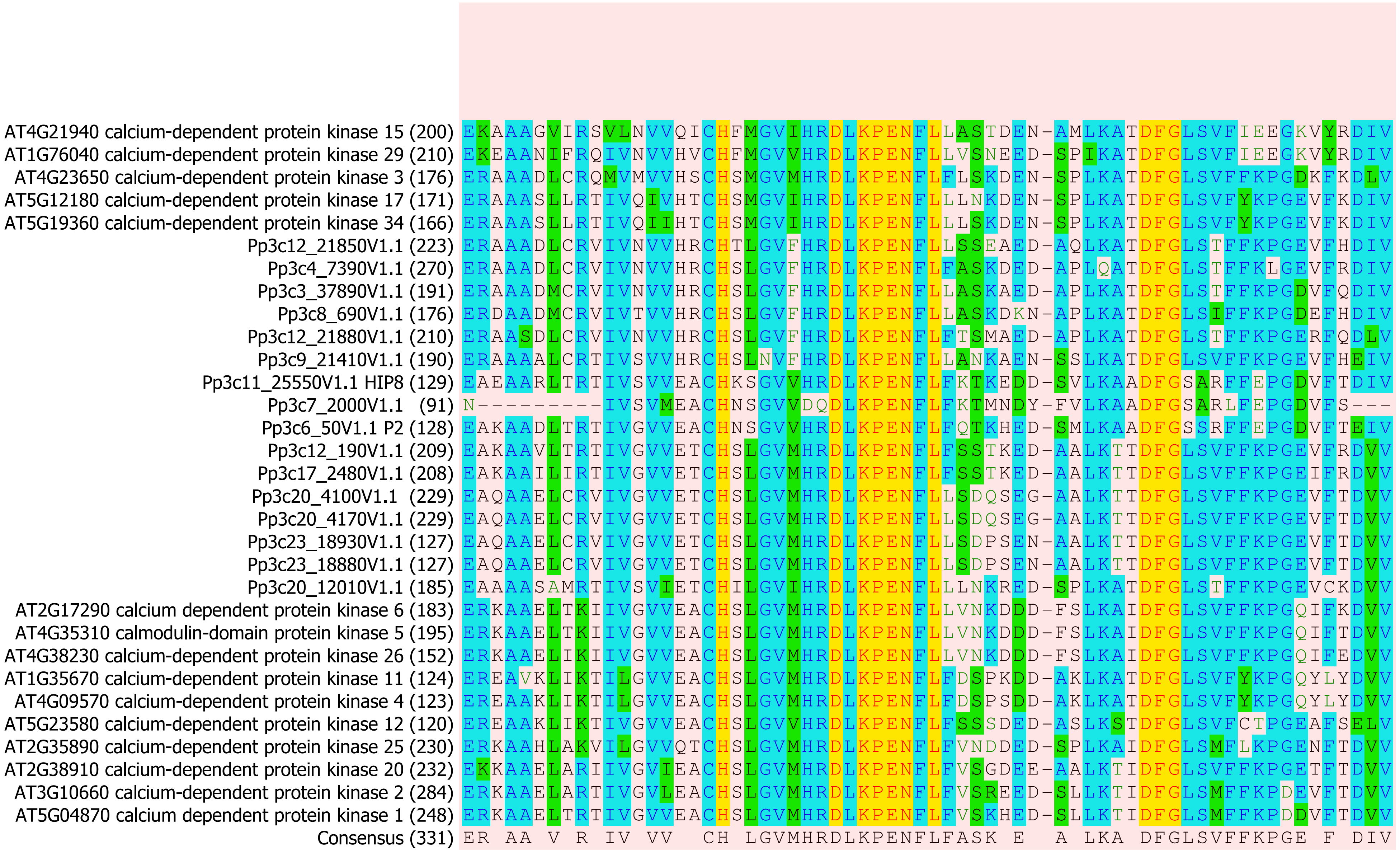
AT4G21940 calcium-dependent protein kinase 15 (200) EKAAAGVIRSVLNVVQICHFMGVIHRDLKPENFLLASTDEN-AMLKATDFGLSVFIEEGKVYRDIV
AT1G76040 calcium-dependent protein kinase 29 (210) EKEAANIFRQIVNVVHVCHFMGVVHRDLKPENFLLVSNEED-SPIKATDFGLSVFIEEGKVYRDIV
AT4G23650 calcium-dependent protein kinase 3 (176) ERAAADLCRQMVMVVHSCHSMGVMHRDLKPENFLFLSKDEN-SPLKATDFGLSVFFKPGDKFKDLV
AT5G12180 calcium-dependent protein kinase 17 (171) ERAAASLLRTIVQIVHTCHSMGVIHRDLKPENFLLLNKDEN-SPLKATDFGLSVFYKPGEVFKDIV
AT5G19360 calcium-dependent protein kinase 34 (166) ERAAASLLRTIVQIIHTCHSMGVIHRDLKPENFLLLSKDEN-SPLKATDFGLSVFYKPGEVFKDIV
Pp3c12_21850V1.1 (223) ERAAADLCRVIVNVVHRCHTLGVFHRDLKPENFLLSSEAED-AQLKATDFGLSTFFKPGEVFHDIV
Pp3c4_7390V1.1 (270) ERAAADLCRVIVNVVHRCHSLGVFHRDLKPENFLFASKDED-APLQATDFGLSTFFKLGEVFRDIV
Pp3c3_37890V1.1 (191) ERAAADMCRVIVNVVHRCHSLGVFHRDLKPENFLLASKAED-APLKATDFGLSTFFKPGDVFQDIV
Pp3c8_690V1.1 (176) ERDAADMCRVIVTVVHRCHSLGVFHRDLKPENFLLASKDKN-APLKATDFGLSIFFKPGDEFHDIV
Pp3c12_21880V1.1 (210) ERAASDLCRVIVNVVHRCHSLGVFHRDLKPENFLFTSMAED-APLKATDFGLSTFFKPGERFQDLV
Pp3c9_21410V1.1 (190) ERAAAALCRTIVSVVHRCHSLNVFHRDLKPENFLLANKAEN-SSLKATDFGLSVFFKPGEVFHEIV
Pp3c11_25550V1.1 HIP8 (129) EAEAARLTRTIVSVVEACHKSGVVHRDLKPENFLFKTKEDD-SVLKAADFGSARFFEPGDVFTDIV
Pp3c7_2000V1.1 (91) N---------IVSVMEACHNSGVVDQDLKPENFLFKTMNDY-FVLKAADFGSARLFEPGDVFS---
Pp3c6_50V1.1 P2 (128) EAKAADLTRTIVGVVEACHNSGVVHRDLKPENFLFQTKHED-SMLKAADFGSSRFFEPGDVFTEIV
Pp3c12_190V1.1 (209) EAKAAVLTRTIVGVVETCHSLGVMHRDLKPENFLFSSTKED-AALKTTDFGLSVFFKPGEVFRDVV
Pp3c17_2480V1.1 (208) EAKAAILIRTIVGVVETCHSLGVMHRDLKPENFLFSSTKED-AALKTTDFGLSVFFKPGEIFRDVV
Pp3c20_4100V1.1 (229) EAQAAELCRVIVGVVETCHSLGVMHRDLKPENFLLSDQSEG-AALKTTDFGLSVFFKPGEVFTDVV
Pp3c20_4170V1.1 (229) EAQAAELCRVIVGVVETCHSLGVMHRDLKPENFLLSDQSEG-AALKTTDFGLSVFFKPGEVFTDVV
Pp3c23_18930V1.1 (127) EAQAAELCRVIVGVVETCHSLGVMHRDLKPENFLLSDPSEN-AALKTTDFGLSVFFKPGEVFTDVV
Pp3c23_18880V1.1 (127) EAQAAELCRVIVGVVETCHSLGVMHRDLKPENFLLSDPSEN-AALKTTDFGLSVFFKPGEVFTDVV
Pp3c20_12010V1.1 (185) EAAAASAMRTIVSVIETCHILGVIHRDLKPENFLLLNKRED-SPLKATDFGLSTFFKPGEVCKDVV
AT2G17290 calcium dependent protein kinase 6 (183) ERKAAELTKIIVGVVEACHSLGVMHRDLKPENFLLVNKDDD-FSLKAIDFGLSVFFKPGQIFKDVV
AT4G35310 calmodulin-domain protein kinase 5 (195) ERKAAELTKIIVGVVEACHSLGVMHRDLKPENFLLVNKDDD-FSLKAIDFGLSVFFKPGQIFTDVV
AT4G38230 calcium-dependent protein kinase 26 (152) ERKAAELIKIIVGVVEACHSLGVMHRDLKPENFLLVNKDDD-FSLKAIDFGLSVFFKPGQIFEDVV
AT1G35670 calcium-dependent protein kinase 11 (124) EREAVKLIKTILGVVEACHSLGVMHRDLKPENFLFDSPKDD-AKLKATDFGLSVFYKPGQYLYDVV
AT4G09570 calcium-dependent protein kinase 4 (123) EREAAKLIKTILGVVEACHSLGVMHRDLKPENFLFDSPSDD-AKLKATDFGLSVFYKPGQYLYDVV
AT5G23580 calcium-dependent protein kinase 12 (120) EREAAKLIKTIVGVVEACHSLGVVHRDLKPENFLFSSSDED-ASLKSTDFGLSVFCTPGEAFSELV
AT2G35890 calcium-dependent protein kinase 25 (230) ERKAAHLAKVILGVVQTCHSLGVMHRDLKPENFLFVNDDED-SPLKAIDFGLSMFLKPGENFTDVV
AT2G38910 calcium-dependent protein kinase 20 (232) EKKAAELARIIVGVIEACHSLGVMHRDLKPENFLFVSGDEE-AALKTIDFGLSVFFKPGETFTDVV
AT3G10660 calcium-dependent protein kinase 2 (284) ERKAAELARTIVGVLEACHSLGVMHRDLKPENFLFVSREED-SLLKTIDFGLSMFFKPDEVFTDVV
AT5G04870 calcium dependent protein kinase 1 (248) ERKAAELTRTIVGVVEACHSLGVMHRDLKPENFLFVSKHED-SLLKTIDFGLSMFFKPDDVFTDVV
Consensus (331) ER AA V R IV VV CH LGVMHRDLKPENFLFASK E A LKA DFGLSVFFKPGE F DIV

Protein kinase / ser/thr / dual specificity protein kinase, catalytic domain

```
                                                  (397) 397          410       420       430       440       450         462
AT1G18890 calcium-dependent protein kinase 10 (226) GSPYYMAPEVLKRD-YGPGVDVWSAGVIIYILLCGVPPFWAETEQGVAL--------AILRGVLDFK
AT1G74740 calcium-dependent protein kinase 30 (222) GSPYYMAPEVLKRN-YGPEVDVWSAGVILYILLCGVPPFWAETEQGVAL--------AILRGVLDFK
AT3G51850 calcium-dependent protein kinase 13 (217) GSPYYMAPEVLKRN-YGPEIDIWSAGVILYILLCGVPPFWAESEQGVAQ--------AILRGVIDFK
AT2G41860 calcium-dependent protein kinase 14 (217) GSPYYMAPEVLRRS-YGQEIDIWSAGVILYILLCGVPPFWAETEHGVAK--------AILKSVIDFK
AT3G57530 calcium-dependent protein kinase 32 (226) GSPYYMAPEVLKRN-YGPEVDIWSAGVILYILLCGVPPFWAETEQGVAQ--------AIIRSVLDFR
AT5G12480 calmodulin-domain protein kinase 7 (222) GSPYYMAPEVLRRN-YGPEIDVWSAGVILYILLCGVPPFWAETEQGVAQ--------AIIRSVIDFK
AT5G19450, calcium-dependent protein kinase 8 (220) GSPYYMAPEVLRRN-YGPEVDIWSAGVILYILLCGVPPFWAETEQGVAQ--------AIIRSVIDFK
Pp3c11_4640V1.1 (207) GSPYYMAPEVLKRN-YGPEVDVWSAGVILYILLCGVPPFWAETEQGVAQ--------AILRGILDFK
Pp3c7_22440V1.1 (207) GSPYYMAPEVLKRN-YGPEVDVWSAGVILYILLCGVPPFWAETEQGVAQ--------AILRGVLDFK
Pp3c11_5760V1.1 (210) GSPYYMAPEVLKRN-YGPEVDVWSAGVILYILLCGVPPFWAETEQGVAQ--------AILRGILDFK
Pp3c11_5820V1.1  (209) GSPYYMAPEVLKRN-YGPEVDVWSAGVILYILLCGVPPFWAETEQGVAQ--------AILRGILDFK
Pp3c7_22710V1.1 (210) GSPYYMAPEVLKRN-YGPEVDVWSAGVILYILLCGVPPFWAETEQGVAQ--------AILRGILDFK
Pp3c7_25180V1.1  (209) GSPYYMAPEVLKRN-YGPEVDVWSAGVILYILLCGVPPFWAETEQGVAQ--------AILRGLLDFK
AT2G31500 calcium-dependent protein kinase 24 (229) GSPYYMAPEVLRRN-YGPEIDVWSAGVILYILLCGVPPFWAETEEGIAH--------AIVRGNIDFE
AT2G17890 calcium-dependent protein kinase 16 (273) GSAYYVAPEVLKRR-SGPESDVWSIGVISYILLCGRRPFWDKTEDGIFK--------EVLKNKPDFR
AT4G36070 calcium-dependent protein kinase 18 (236) GSAYYVAPEVLKRR-SGPESDVWSIGVITYILLCGRRPFWDKTQDGIFN--------EVMRKKPDFR
AT5G66210 calcium-dependent protein kinase 28 (227) GSAYYVAPEVLKRR-SGPESDVWSIGVITYILLCGRRPFWDRTEDGIFK--------EVLRNKPDFS
Pp3c15_3590V1.1 (302) GSAYYVAPEVLKRK-SGPESDVWSIGVITYILLCGRRPFWDKTEQGIFN--------EVLKKKPDFR
Pp3c9_4620V1.1 (291) GSAYYVAPEVLKKK-SGPESDVWSIGVITYILLCGRRPFWDKTEKGIFD--------EVLKKNPDYG
Pp3c15_5120V1.1 (295) GSAYYVAPEVLNRK-SGPESDVWSIGVITYILLCGRRPFWDKTEAGIFN--------EVLKKKPDFR
Pp3c9_5860V1.1 (255) GSAYYVAPEVLNRK-SGPESDVWSIGVITYILLCGRRPFWDKTEAGIFN--------EVLKKKPDFR
Pp3c13_4100V1.1 (310) GSAYYVAPEVLHRS-YSMEADVWSIGVITYILLCGSRPFWARTESGIFR--------AVLRADPSFD
Pp3c3_5990V1.1 (310) GSAYYVAPEVLHRS-YSMEADVWSIGVITYILLCGSRPFWARTESGIFR--------AVLRADPSFE
Pp3c3_5970V1.1 (306) GSAYYVAPEVLHRS-YSMEADVWSIGVITYILLCGSRPFWARTESGIFR--------AVLRADPSFE
Pp3c26_4540V1.1 (312) GSAYYVAPEVLHRS-YSMEADVWSVGVITYILLCGSRPFWARTESGIFR--------AVLRADPSFE
Pp3c4_25010V1.1  (311) GSAYYVAPEVLHRL-YSMEADVWSIGVITYILLCGSRPFWARTESGIFR--------AVLRADPSFE
Pp3c19_20580V1.1 (197) GSMDYMAPEQLNMSGTMPANDRWSLGVILYILLCGYPPFRAKTTRDKQR--------RILTGAYSME
Pp3c21_15330V1.1 (191) GSIDYMAPEQLSLSGIMPANDMWSLGVILYILLCGYPPFRARSSRDKQM--------QILTGSYSME
AT1G50700 calcium-dependent protein kinase 33 (236) GSAYYVAPEVLKRR-YGKEIDIWSAGIILYILLSGVPPFWAETEKGIFD--------AILEGEIDFE
AT3G20410 calmodulin-domain protein kinase 9 (254) GSAYYVAPEVLRRR-YGKEVDIWSAGIILYILLSGVPPFWAETEKGIFD--------AILEGHIDFE
AT1G61950 calcium-dependent protein kinase 19 (262) GSAYYVAPEVLKRN-YGKAIDIWSAGVILYILLCGNPPFWAETDKGIFE--------EILRGEIDFE
AT4G04695 calcium-dependent protein kinase 31 (195) GSAYYIAPEVLQGK-YGKEADIWSAGIILYILLCGKPPFVTEPEAQMFS--------EIKSAKIDVD
AT4G04700 calcium-dependent protein kinase 27 (195) GSDYYIAPEVLQGN-YGKEADIWSAGIILYILLCGKSPFVKEPEGQMFN--------EIKSLEIDYS
AT4G04710 calcium-dependent protein kinase 22 (189) GSKYYIAPEVLEGS-YGKEIDIWSAGVILYILLSGVPPFQTGIESIIVSTLCIVDAEIKECRLDFE
AT4G04720 calcium-dependent protein kinase 21 (243) GSAYYVAPEVLRRS-YGKEIDIWSAGVILYILLSGVPPFWAENEKGIFD--------EVIKGEIDFV
AT4G04740 calcium-dependent protein kinase 23 (232) GSPYYVAPEVLRQS-YGKEIDIWSAGVILYILLCGVPPFWADNEEGVFV--------EILKCKIDFV
```

```
AT4G21940 calcium-dependent protein kinase 15 (265) GSAYYVAPEVLRRS-YGKEIDIWSAGIILYILLCGVPPFWSETEKGIFN-------EIIKGEIDFD
AT1G76040 calcium-dependent protein kinase 29 (275) GSAYYVAPEVLHRN-YGKEIDVWSAGVMLYILLSGVPPFWGETEKTIFE-------AILEGKLDLE
 AT4G23650 calcium-dependent protein kinase 3 (241) GSAYYVAPEVLKRN-YGPEADIWSAGVILYILLSGVPPFWGENETGIFD-------AILQGQLDFS
AT5G12180 calcium-dependent protein kinase 17 (236) GSAYYIAPEVLKRK-YGPEADIWSIGVMLYILLCGVPPFWAESENGIFN-------AILRGHVDFS
AT5G19360 calcium-dependent protein kinase 34 (231) GSAYYIAPEVLRRK-YGPEADIWSIGVMLYILLCGVPPFWAESENGIFN-------AILSGQVDFS
                           Pp3c12_21850V1.1 (288) GSAYYVAPEVLRRN-YGPEADVWSAGVIVYILLCGVPPFWAETEQGIFD-------AVLRGHIDFV
                             Pp3c4_7390V1.1 (335) GSAYYVAPEVLKRN-YGPEADVWSAGVIVYILLCGVPPFWAESEQGIFD-------AVLKGHIDFE
                            Pp3c3_37890V1.1 (256) GSAYYVAPEVLKRS-YGPEADVWSAGVIVYILLCGVPPFWAETEQGIFD-------AVLKGHIDFE
                              Pp3c8_690V1.1 (241) GSAYYVAPEVLKRS-YGPEADVWSAGVIVYILLCGVPPFWAETEKEIFD-------TIMRGHIDFK
                           Pp3c12_21880V1.1 (275) GTAYYIAPEVLRKD-YGPEADVWSAGVILYILLCGVPPFWAETEKGIFD-------AIMRGTLDFT
                            Pp3c9_21410V1.1 (255) GSAYYVAPEVLRRN-YGPEADVWSAGVIVYILLCGVPPFWAESEQGIFD-------AVLKGYIDFK
                      Pp3c11_25550V1.1 HIP8 (194) GSPYYVAPEVLDRH-YGPEADIWSAGVMLYILLSGAPPFWAETVQGIFEK------VMEGEPPTFT
                             Pp3c7_2000V1.1 (144) -----------------------------------ERAQGILER------KMEGVPPVFT
                          Pp3c6_50V1.1 P2 (193) GSPFYVAPEVLDRH-YGPEADIWSAGVILYILLSGVPPFWAETVQGIFEE------VMKGEPPSFA
                            Pp3c12_190V1.1 (274) GSPYYVAPEVLRKN-YGPEADVWSAGVILYILLSGVPPFWAETEQGIFEQ------VLKSELD-FA
                           Pp3c17_2480V1.1 (273) GSPYYVAPEVLRKN-YGPEADVWSAGVILYILLCGVPPFWAETEQGIFEQ------VLKSELD-FA
                           Pp3c20_4100V1.1  (294) GSPYYVAPEVLRKH-YGPEADVWSAGVILYILLSGVPPFWAETEQGIFEQ------VLKGELD-FV
                           Pp3c20_4170V1.1 (294) GSPYYVAPEVLRKH-YGPEADVWSAGVILYILLSGVPPFWAETEQGIFEQ------VLKGELD-FV
                          Pp3c23_18930V1.1 (192) GSPYYVAPEVLRKH-YGPEADVWSAGVILYILLSGVPPFWAETEQGIFEQ------VLAGELD-FV
                          Pp3c23_18880V1.1 (192) GSPYYVAPEVLRKH-YGPEADVWSAGVILYILLSGVPPFWAETEQGIFEQ------VLAGELD-FV
                          Pp3c20_12010V1.1 (250) GSAFYVAPEVLRRK-YGPESDIWSAGVILYILLSGVPPFWADTEDGIFAE------VLKAKVD-FD
 AT2G17290 calcium dependent protein kinase 6 (248) GSPYYVAPEVLLKH-YGPEADVWTAGVILYILLSGVPPFWAETQQGIFDA------VLKGYID-FD
 AT4G35310 calmodulin-domain protein kinase 5 (260) GSPYYVAPEVLLKR-YGPEADVWTAGVILYILLSGVPPFWAETQQGIFDA------VLKGYID-FE
AT4G38230 calcium-dependent protein kinase 26 (217) GSPYYVAPEVLLKH-YGPEADVWTAGVILYILVSGVPPFWAETQQGIFDA------VLKGHID-FD
AT1G35670 calcium-dependent protein kinase 11 (189) GSPYYVAPEVLKKC-YGPEIDVWSAGVILYILLSGVPPFWAETESGIFRQ------ILQGKLD-FK
 AT4G09570 calcium-dependent protein kinase 4 (188) GSPYYVAPEVLKKC-YGPEIDVWSAGVILYILLSGVPPFWAETESGIFRQ------ILQGKID-FK
AT5G23580 calcium-dependent protein kinase 12 (185) GSAYYVAPEVLHKH-YGPECDVWSAGVILYILLCGFPPFWAESEIGIFRK------ILQGKLE-FE
AT2G35890 calcium-dependent protein kinase 25 (295) GSPYYIAPEVLNKN-YGPEADIWSAGVMIYVLLSGSAPFWGETEEEIFNE------VLEGELD-LT
AT2G38910 calcium-dependent protein kinase 20 (297) GSPYYVAPEVLRKH-YSHECDVWSAGVIIYILLSGVPPFWDETEQGIFEQ------VLKGDLD-FI
 AT3G10660 calcium-dependent protein kinase 2 (349) GSPYYVAPEVLRKR-YGPESDVWSAGVIVYILLSGVPPFWAETEQGIFEQ------VLHGDLD-FS
 AT5G04870 calcium dependent protein kinase 1 (313) GSPYYVAPEVLRKR-YGPEADVWSAGVIVYILLSGVPPFWAETEQGIFEQ------VLHGDLD-FS
                                Consensus (397) GS YYVAPEVLKR  YGPEADVWSAGVILYILLCGVPPFWAETEQGIF         ILR   DF
```

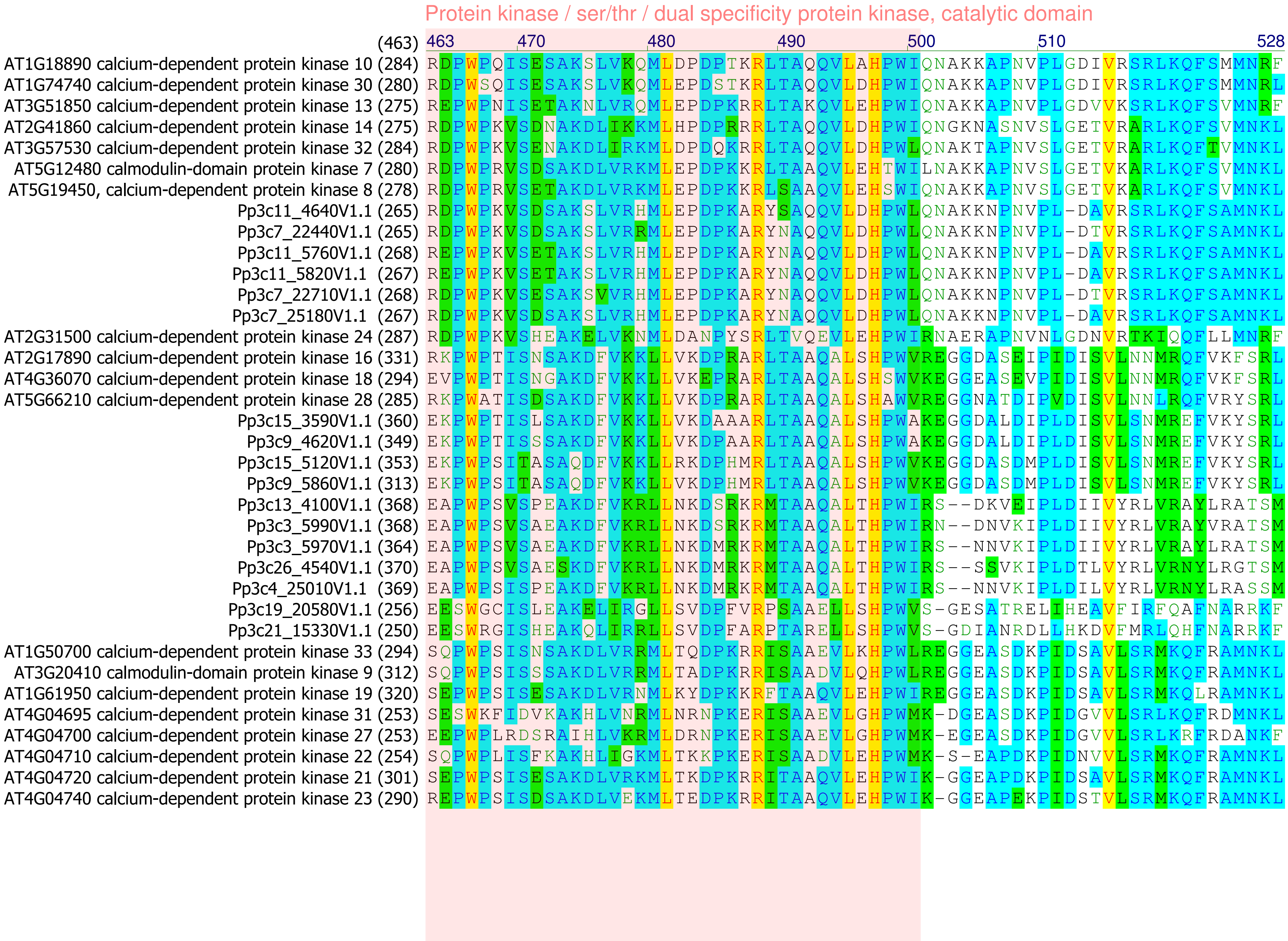
Protein kinase / ser/thr / dual specificity protein kinase, catalytic domain
(463) 463 470 480 490 500 510 528
AT1G18890 calcium-dependent protein kinase 10 (284) RDPWPQISESAKSLVKQMLDPDPTKRLTAQQVLAHPWIQNAKKAPNVPLGDIVRSRLKQFSMMNRF
AT1G74740 calcium-dependent protein kinase 30 (280) RDPWSQISESAKSLVKQMLEPDSTKRLTAQQVLDHPWIQNAKKAPNVPLGDIVRSRLKQFSMMNRL
AT3G51850 calcium-dependent protein kinase 13 (275) REPWPNISETAKNLVRQMLEPDPKRRLTAKQVLEHPWIQNAKKAPNVPLGDVVKSRLKQFSVMNRF
AT2G41860 calcium-dependent protein kinase 14 (275) RDPWPKVSDNAKDLIKKMLHPDPRRRLTAQQVLDHPWIQNGKNASNVSLGETVRARLKQFSVMNKL
AT3G57530 calcium-dependent protein kinase 32 (284) RDPWPKVSENAKDLIRKMLDPDQKRRLTAQQVLDHPWLQNAKTAPNVSLGETVRARLKQFTVMNKL
AT5G12480 calmodulin-domain protein kinase 7 (280) RDPWPRVSDSAKDLVRKMLEPDPKKRLTAAQVLEHTWILNAKKAPNVSLGETVKARLKQFSVMNKL
AT5G19450, calcium-dependent protein kinase 8 (278) RDPWPRVSETAKDLVRKMLEPDPKKRLSAAQVLEHSWIQNAKKAPNVSLGETVKARLKQFSVMNKL
Pp3c11_4640V1.1 (265) RDPWPKVSDSAKSLVRHMLEPDPKARYSAQQVLDHPWLQNAKKNPNVPL-DAVRSRLKQFSAMNKL
Pp3c7_22440V1.1 (265) RDPWPKVSDSAKSLVRRMLEPDPKARYNAQQVLDHPWLQNAKKNPNVPL-DTVRSRLKQFSAMNKL
Pp3c11_5760V1.1 (268) REPWPKVSETAKSLVRHMLEPDPKARYNAQQVLDHPWLQNAKKNPNVPL-DAVRSRLKQFSAMNKL
Pp3c11_5820V1.1 (267) REPWPKVSETAKSLVRHMLEPDPKARYNAQQVLDHPWLQNAKKNPNVPL-DAVRSRLKQFSAMNKL
Pp3c7_22710V1.1 (268) RDPWPKVSESAKSVVRHMLEPDPKARYNAQQVLDHPWLQNAKKNPNVPL-DTVRSRLKQFSAMNKL
Pp3c7_25180V1.1 (267) RDPWPKVSDSAKSLVRHMLEPDPKARYNAQQVLDHPWLQNAKKNPNVPL-DAVRSRLKQFSAMNKL
AT2G31500 calcium-dependent protein kinase 24 (287) RDPWPKVSHEAKELVKNMLDANPYSRLTVQEVLEHPWIRNAERAPNVNLGDNVRTKIQQFLLMNRF
AT2G17890 calcium-dependent protein kinase 16 (331) RKPWPTISNSAKDFVKKLLVKDPRARLTAAQALSHPWVREGGDASEIPIDISVLNNMRQFVKFSRL
AT4G36070 calcium-dependent protein kinase 18 (294) EVPWPTISNGAKDFVKKLLVKEPRARLTAAQALSHSWVKEGGEASEVPIDISVLNNMRQFVKFSRL
AT5G66210 calcium-dependent protein kinase 28 (285) RKPWATISDSAKDFVKKLLVKDPRARLTAAQALSHAWVREGGNATDIPVDISVLNNLRQFVRYSRL
Pp3c15_3590V1.1 (360) EKPWPTISLSAKDFVKKLLVKDAAARLTAAQALSHPWAKEGGDALDIPLDISVLSNMREFVKYSRL
Pp3c9_4620V1.1 (349) EKPWPTISSSAKDFVKKLLVKDPAARLTAAQALSHPWAKEGGDALDIPLDISVLSNMREFVKYSRL
Pp3c15_5120V1.1 (353) EKPWPSITASAQDFVKKLLRKDPHMRLTAAQALSHPWVKEGGDASDMPLDISVLSNMREFVKYSRL
Pp3c9_5860V1.1 (313) EKPWPSITASAQDFVKKLLVKDPHMRLTAAQALSHPWVKEGGDASDMPLDISVLSNMREFVKYSRL
Pp3c13_4100V1.1 (368) EAPWPSVSPEAKDFVKRLLNKDSRKRMTAAQALTHPWIRS--DKVEIPLDIIVYRLVRAYLRATSM
Pp3c3_5990V1.1 (368) EAPWPSVSAEAKDFVKRLLNKDSRKRMTAAQALTHPWIRN--DNVKIPLDIIVYRLVRAYVRATSM
Pp3c3_5970V1.1 (364) EAPWPSVSAEAKDFVKRLLNKDMRKRMTAAQALTHPWIRS--NNVKIPLDIIVYRLVRAYLRATSM
Pp3c26_4540V1.1 (370) EAPWPSVSAESKDFVKRLLNKDMRKRMTAAQALTHPWIRS--SSVKIPLDTLVYRLVRNYLRGTSM
Pp3c4_25010V1.1 (369) EAPWPSISPEAKDFVKRLLNKDMRKRMTAAQALTHPWIRS--NNVKIPLDILVYRLVRNYLRASSM
Pp3c19_20580V1.1 (256) EESWGCISLEAKELIRGLLSVDPFVRPSAAELLSHPWVS-GESATRELIHEAVFIRFQAFNARRKF
Pp3c21_15330V1.1 (250) EESWRGISHEAKQLIRRLLSVDPFARPTARELLSHPWVS-GDIANRDLLHKDVFMRLQHFNARRKF
AT1G50700 calcium-dependent protein kinase 33 (294) SQPWPSISNSAKDLVRRMLTQDPKRRISAAEVLKHPWLREGGEASDKPIDSAVLSRMKQFRAMNKL
AT3G20410 calmodulin-domain protein kinase 9 (312) SQPWPSISSSAKDLVRRMLTADPKRRISAADVLQHPWLREGGEASDKPIDSAVLSRMKQFRAMNKL
AT1G61950 calcium-dependent protein kinase 19 (320) SEPWPSISESAKDLVRNMLKYDPKKRFTAAQVLEHPWIREGGEASDKPIDSAVLSRMKQLRAMNKL
AT4G04695 calcium-dependent protein kinase 31 (253) SESWKFIDVKAKHLVNRMLNRNPKERISAAEVLGHPWMK-DGEASDKPIDGVVLSRLKQFRDMNKL
AT4G04700 calcium-dependent protein kinase 27 (253) EEPWPLRDSRAIHLVKRMLDRNPKERISAAEVLGHPWMK-EGEASDKPIDGVVLSRLKRFRDANKF
AT4G04710 calcium-dependent protein kinase 22 (254) SQPWPLISFKAKHLIGKMLTKKPKERISAADVLEHPWMK-S-EAPDKPIDNVVLSRMKQFRAMNKL
AT4G04720 calcium-dependent protein kinase 21 (301) SEPWPSISESAKDLVRKMLTKDPKRRITAAQVLEHPWIK-GGEAPDKPIDSAVLSRMKQFRAMNKL
AT4G04740 calcium-dependent protein kinase 23 (290) REPWPSISDSAKDLVEKMLTEDPKRRITAAQVLEHPWIK-GGEAPEKPIDSTVLSRMKQFRAMNKL

```
AT4G21940 calcium-dependent protein kinase 15 (323) SQPWPSISESAKDLVRKLLTKDPKQRISAAQALEHPWIR-GGEAPDKPIDSAVLSRMKQFRAMNKL
AT1G76040 calcium-dependent protein kinase 29 (333) TSPWPTISESAKDLIRKMLIRDPKKRITAAEALEHPWMT-DTKISDKPINSAVLVRMKQFRAMNKL
AT4G23650 calcium-dependent protein kinase 3 (299) ADPWPALSDGAKDLVRKMLKYDPKDRLTAAEVLNHPWIREDGEASDKPLDNAVLSRMKQFRAMNKL
AT5G12180 calcium-dependent protein kinase 17 (294) SDPWPSISPQAKDLVKKMLNSDPKQRLTAAQVLNHPWIKEDGEAPDVPLDNAVMSRLKQFKAMNNF
AT5G19360 calcium-dependent protein kinase 34 (289) SDPWPVISPQAKDLVRKMLNSDPKQRLTAAQVLNHPWIKEDGEAPDVPLDNAVMSRLKQFKAMNNF
Pp3c12_21850V1.1 (346) SDPWPKISSGAKDLVRKMLNMNVKERLTAYQVLNHPWMEEGGDASDTPLDNAVLTRLKNFSTANKM
Pp3c4_7390V1.1 (393) SEPWPRISSGAVDLVRNMLNPNVKERLTAYQVLNHPWMQEGGDASDEPLDNAVLDRLKNFSAANKM
Pp3c3_37890V1.1 (314) NDPWPKISNGAKDLVRKMLNPNVKIRLTAQQVLNHPWMKEDGDAPDVPLDNAVLTRLKNFSAANKM
Pp3c8_690V1.1 (299) SDPWPKISDEAKDLVKKMLNSNVKERLTAQEVLNHPWMQRDG-VPDVPLDNAVLTRLRNFSAANKM
Pp3c12_21880V1.1 (333) SDPWPRISDDAKVLVKGMLNPDVNARLTAQQVLDHPWMKEDG-ASNAPLDNAVLTRLKNFSAANKM
Pp3c9_21410V1.1 (313) SDPWPKVSAAAKDLVSKMLKQDPKERLTAQEVLKHPWMKEDGDAPDEPLDNAVLTRLKNFSSANKM
Pp3c11_25550V1.1 HIP8 (253) ADPWPNISEVAKDLIRKMLDPNPEKRLKAHEVLNHPWIREDGVAPKKPIASLVQFRMKQFAAMNKL
Pp3c7_2000V1.1 (163) ANPWPNIFEGAKDLTRMMLNPDLKQRLNAHEILEHSCIREDGGTLKKQVTSLVQFRMNQFALLNKL
Pp3c6_50V1.1 P2 (252) ADPWPNISEGAKDLIRQMLNPDPRKRLTAAEVLKHPWIREDGVASNKPIASLVQFRLKQFSAMNKL
Pp3c12_190V1.1 (332) SDPWPKISDSAKDLIRKMLNPQASKRLKAHQVLCHPWICEDGVAPDRPIDSAVQSRLKHFSAMNKL
Pp3c17_2480V1.1 (331) TDPWPKISDSAKDLIRKMLNPEASKRMKAHHVLCHPWICEDGVAPDRPIDSAVQSRLKHFSAMNKL
Pp3c20_4100V1.1 (352) SEPWPSISDSAKDLIRRMLDPNAKRRLKAHQVLSHPWIGEEGVAPDRPMDPAVQSRLKQFSAMNKL
Pp3c20_4170V1.1 (352) SEPWPSISDSAKDLIRRMLDPNAKRRLKAHQVLSHPWIGEEGVAPDRPMDPAVQSRLKQFSAMNKL
Pp3c23_18930V1.1 (250) SEPWPSISESAKDLIRRMLDPVAKRRLKAHQVLSHPWIREAGVAPDRPMDPAVQSRLKQFSAMNKL
Pp3c23_18880V1.1 (250) SEPWPSISESAKDLIRRMLDPVAKRRLKAHQVLSHPWIREAGVAPDRPMDPAVQSRLKQFSAMNKL
Pp3c20_12010V1.1 (308) TDPWPKISKDAKDLIRKILNPDVKARLTASEVLAHPWVREKGVASTKPMDSSVQNRLKRFAAMNKM
AT2G17290 calcium dependent protein kinase 6 (306) TDPWPVISDSAKDLIRKMLCSSPSERLTAHEVLRHPWICENGVAPDRALDPAVLSRLKQFSAMNKL
AT4G35310 calmodulin-domain protein kinase 5 (318) SDPWPVISDSAKDLIRRMLSSKPAERLTAHEVLRHPWICENGVAPDRALDPAVLSRLKQFSAMNKL
AT4G38230 calcium-dependent protein kinase 26 (275) SDPWPLISDSAKNLIRGMLCSRPSERLTAHQVLRHPWICENGVAPDRALDPAVLSRLKQFSAMNKL
AT1G35670 calcium-dependent protein kinase 11 (247) SDPWPTISEAAKDLIYKMLERSPKKRISAHEALCHPWIVDEQAAPDKPLDPAVLSRLKQFSQMNKI
AT4G09570 calcium-dependent protein kinase 4 (246) SDPWPTISEGAKDLIYKMLDRSPKKRISAHEALCHPWIVDEHAAPDKPLDPAVLSRLKQFSQMNKI
AT5G23580 calcium-dependent protein kinase 12 (243) INPWPSISESAKDLIKKMLESNPKKRLTAHQVLCHPWIVDDKVAPDKPLDCAVVSRLKKFSAMNKL
AT2G35890 calcium-dependent protein kinase 25 (353) SDPWPQVSESAKDLIRKMLERNPIQRLTAQQVLCHPWIRDEGNAPDTPLDTTVLSRLKKFSATDKL
AT2G38910 calcium-dependent protein kinase 20 (355) SEPWPSVSESAKDLVRRMLIRDPKKRMTTHEVLCHPWARVDGVALDKPLDSAVLSRLQQFSAMNKL
AT3G10660 calcium-dependent protein kinase 2 (407) SDPWPSISESAKDLVRKMLVRDPKRRLTAHQVLCHPWVQIDGVAPDKPLDSAVLSRMKQFSAMNKF
AT5G04870 calcium dependent protein kinase 1 (371) SDPWPSISESAKDLVRKMLVRDPKKRLTAHQVLCHPWVQVDGVAPDKPLDSAVLSRMKQFSAMNKF
Consensus (463)  DPWP ISESAKDLVRKML  DPK RLTA QVL HPWIRE G APD PLD AVLSRLKQFSAMNKL
```

Calcium-binding EF-hand #1

```
                                                       529        540       550       560       570       580         594
                                               (529)   KKKVLRVIAEHLSIQEVEVIKNMFSLMDDDKDGKITYPEL--------KAGLQKVG-SQLGEPEIK
AT1G18890 calcium-dependent protein kinase 10 (350)   KKKVLRVIAEHLSIQEVEVIKNMFSLMDDDKDGKITYPEL--------KAGLQKVG-SQLGEPEIK
AT1G74740 calcium-dependent protein kinase 30 (346)   KKKALRVIAEHLSIQEVEVIRNMFTLMDDDNDGKISYLEL--------RAGLRKVG-SQLGEPEIK
AT3G51850 calcium-dependent protein kinase 13 (341)   KRKALRVIAEFLSTEEVEDIKVMFNKMDTDNDGIVSIEEL--------KAGLRDFS-TQLAESEVQ
AT2G41860 calcium-dependent protein kinase 14 (341)   KKRALRVIAEHLSVEETSCIKERFQVMDTSNRGKITITEL--------GIGLQKLG-IVVPQDDIQ
AT3G57530 calcium-dependent protein kinase 32 (350)   KKRALRVIAEHLSDEEASGIREGFQIMDTSQRGKINIDEL--------KIGLQKLG-HAIPQDDLQ
AT5G12480 calmodulin-domain protein kinase 7 (346)    KKRALRVIAEHLSVEEAAGIKEAFEMMDVNKRGKINLEEL--------KYGLQKAG-QQIADTDLQ
AT5G19450, calcium-dependent protein kinase 8 (344) KKRALRVIAEHLSVEEVAGIKEAFEMMDSKKTGKINLEEL--------KFGLHKLGQQQIPDTDLQ
Pp3c11_4640V1.1 (330)                               KKRALQVIAEHLGGEEIDGLKEIFEKLDSDKTGTITFEKL--------KMGLIEIG-SQLTEHEVR
Pp3c7_22440V1.1 (330)                               KKRALQVIAESLGGEEMNGLKEMFEKLDSDNAGVITFEKL--------KMGLIEIG-SQLTEHEVR
Pp3c11_5760V1.1 (333)                               KKRALQVIAEHLGGEEIDGLKEMFEKLDSDKTGTITFEKL--------KMGLIEIG-SQLTEHEVR
Pp3c11_5820V1.1  (332)                              KKRALQVIAEHLGGEEIDGLKEMFEKLDSDKTGTITFEKL--------KMGLIEIG-SQLTEHEVR
Pp3c7_22710V1.1 (333)                               KKRALQVIAEHLGGEEIDGLKEMFEKLDSDNTGTITFEKL--------KMGLIEIG-SQLTEHEVR
Pp3c7_25180V1.1  (332)                              KKRALQVIAERLGGEEIDGLKEILEKLDIDNMGVITFEKL--------KMGLIEIG-SQLTEHEVR
AT2G31500 calcium-dependent protein kinase 24 (353)   KKKVLRIVADNLPNEEIAAIVQMFQTMDTDKNGHLTFEEL--------RDGLKKIG-QVVPDGDVK
AT2G17890 calcium-dependent protein kinase 16 (397)   KQFALRALATTLDEEELADLRDQFDAIDVDKNGVISLEEM--------RQALAKDHPWKLKDARVA
AT4G36070 calcium-dependent protein kinase 18 (360)   KQIALRALAKTINEDELDDLRDQFDAIDIDKNGSISLEEM--------RQALAKDVPWKLKDARVA
AT5G66210 calcium-dependent protein kinase 28 (351)   KQFALRALASTLDEAEISDLRDQFDAIDVDKNGVISLEEM--------RQALAKDLPWKLKDSRVA
Pp3c15_3590V1.1 (426)                               KQLALRALASTLEDSDIADLRDQFNAIDIDRNGTITLEEM--------REALQKDRPWVIKESRVG
Pp3c9_4620V1.1 (415)                                KQLALRALASTLDSSDIADLQDQFNAIDIDRNGKITLEEM--------REALQKDRPWSIQESRIV
Pp3c15_5120V1.1 (419)                               KQLALRALASTLESDEIRDLRDQFDAMDVDRNGTITLEEI--------KHALQKDRPWAVKESRVL
Pp3c9_5860V1.1 (379)                                KQLALRALASTLEPEEIRDLRDQFDAMDVDRNGTITLEEI--------RHALQKDRPWAVKESRVL
Pp3c13_4100V1.1 (432)                               RRAALKALSKTLTEDELSYLQTQFSLLEPNKSGRISYDNF--------KQALMKNSTEAMKEARVF
Pp3c3_5990V1.1 (432)                                RRAALKALSKTLTEDDLFYLQSQFSLLEPSRSGRISFDNF--------RQALAKNSTEAMKEARVF
Pp3c3_5970V1.1 (428)                                RKAALKALSKTLTEEELFYLHTQFMLLEPNRSGRITFENF--------RQALFKNSTEAMKESRVF
Pp3c26_4540V1.1 (434)                               RKAALKALSKTLTEDETFFLHTQFMLLEPNKSGRVTFENF--------RQALLKNSTEAMKESRVF
Pp3c4_25010V1.1  (433)                              RKAALKALSKTLTEDETFYLRTQFMLLEPSNNGRVTFENF--------RQALLKNSTEAMKESRVF
Pp3c19_20580V1.1 (321)                              RAAAYASIVRTKFQLRTKYLKELIGDCILTGAELEDFTS-------NGRTATLEEFQHILCSMDLQ
Pp3c21_15330V1.1 (315)                              RATAYASIVRTKFLLRTRYLKELLGDRVLTDSELEALRVNFMRISSNGQTATLKEFEEVLRSINLH
AT1G50700 calcium-dependent protein kinase 33 (360)   KKLALKVIAENIDTEEIQGLKAMFANIDTDNSGTITYEEL--------KEGLAKLG-SRLTEAEVK
AT3G20410 calmodulin-domain protein kinase 9 (378)    KKLALKVIAENIDTEEIQGLKAMFANIDTDNSGTITYEEL--------KEGLAKLG-SKLTEAEVK
AT1G61950 calcium-dependent protein kinase 19 (386)   KKLAFKFIAQNLKEEELKGLKTMFANMDTDKSGTITYDEL--------KSGLEKLG-SRLTETEVK
AT4G04695 calcium-dependent protein kinase 31 (318)   KKVALKVIAANLSEEEIKGLKTLFTNIDTDKSGTITLEEL--------KTGLTRLG-SNLSKTEVE
AT4G04700 calcium-dependent protein kinase 27 (318)   KKVVLKFIAANLSEEEIKGLKTLFTNIDTDKSGNITLEEL--------KTGLTRLG-SNLSKTEVE
AT4G04710 calcium-dependent protein kinase 22 (318)   KKLALKVIAEGLSEEEIKGLKTMFENMDMDKSGSITYEEL--------KMGLNRHG-SKLSETEVK
AT4G04720 calcium-dependent protein kinase 21 (366)   KKLALKVIAESLSEEEIKGLKTMFANIDTDKSGTITYEEL--------KTGLTRLG-SRLSETEVK
AT4G04740 calcium-dependent protein kinase 23 (355)   KKLALKVSAVSLSEEEIKGLKTLFANMDTNRSGTITYEQL--------QTGLSRLR-SRLSETEVQ
```

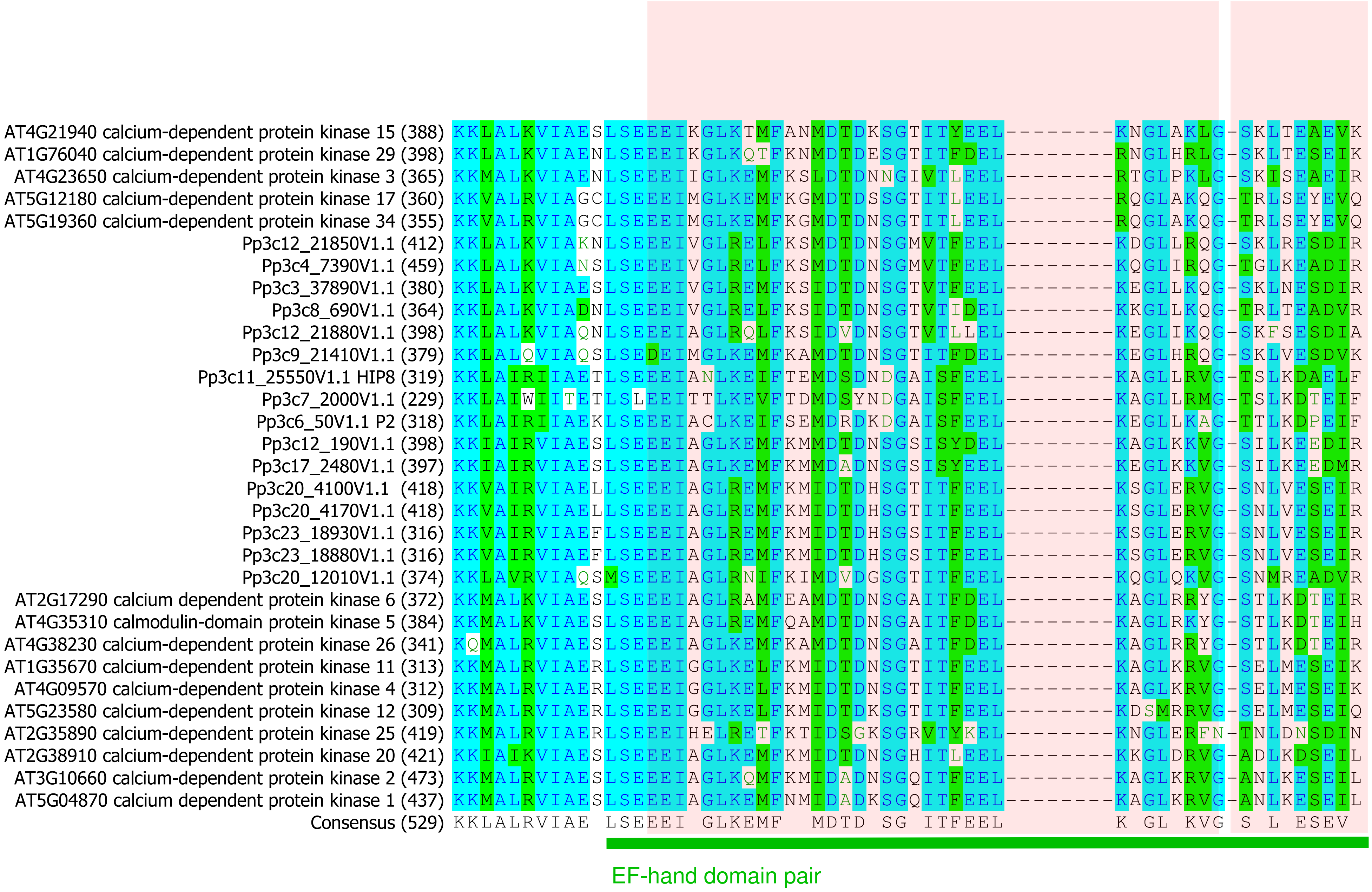
AT4G21940 calcium-dependent protein kinase 15 (388) KKLALKVIAESLSEEEIKGLKTMFANMDTDKSGTITYEEL--------KNGLAKLG-SKLTEAEVK
AT1G76040 calcium-dependent protein kinase 29 (398) KKLALKVIAENLSEEEIKGLKQTFKNMDTDESGTITFDEL--------RNGLHRLG-SKLTESEIK
AT4G23650 calcium-dependent protein kinase 3 (365) KKMALKVIAENLSEEEIIGLKEMFKSLDTDNNGIVTLEEL--------RTGLPKLG-SKISEAEIR
AT5G12180 calcium-dependent protein kinase 17 (360) KKVALRVIAGCLSEEEIMGLKEMFKGMDTDSSGTITLEEL--------RQGLAKQG-TRLSEYEVQ
AT5G19360 calcium-dependent protein kinase 34 (355) KKVALRVIAGCLSEEEIMGLKEMFKGMDTDNSGTITLEEL--------RQGLAKQG-TRLSEYEVQ
Pp3c12_21850V1.1 (412) KKLALKVIAKNLSEEEIVGLRELFKSMDTDNSGMVTFEEL--------KDGLLRQG-SKLRESDIR
Pp3c4_7390V1.1 (459) KKLALKVIANSLSEEEIVGLRELFKSMDTDNSGMVTFEEL--------KQGLIRQG-TGLKEADIR
Pp3c3_37890V1.1 (380) KKLALKVIAESLSEEEIVGLREMFKSIDTDNSGTVTFEEL--------KEGLLKQG-SKLNESDIR
Pp3c8_690V1.1 (364) KKLALKVIADNLSEEEIVGLRELFKSIDTDNSGTVTIDEL--------KKGLLKQG-TRLTEADVR
Pp3c12_21880V1.1 (398) KKLALKVIAQNLSEEEIAGLRQLFKSIDVDNSGTVTLLEL--------KEGLIKQG-SKFSESDIA
Pp3c9_21410V1.1 (379) KKLALQVIAQSLSEDEIMGLKEMFKAMDTDNSGTITFDEL--------KEGLHRQG-SKLVESDVK
Pp3c11_25550V1.1 HIP8 (319) KKLAIRIIAETLSEEEIANLKEIFTEMDSDNDGAISFEEL--------KAGLLRVG-TSLKDAELF
Pp3c7_2000V1.1 (229) KKLAIWIITETLSLEEITTLKEVFTDMDSYNDGAISFEEL--------KAGLLRMG-TSLKDTEIF
Pp3c6_50V1.1 P2 (318) KKLAIRIIAEKLSEEEIACLKEIFSEMDRDKDGAISFEEL--------KEGLLKAG-TTLKDPEIF
Pp3c12_190V1.1 (398) KKIAIRVIAESLSEEEIAGLKEMFKMMDTDNSGSISYDEL--------KAGLKKVG-SILKEEDIR
Pp3c17_2480V1.1 (397) KKIAIRVIAESLSEEEIAGLKEMFKMMDADNSGSISYEEL--------KEGLKKVG-SILKEEDMR
Pp3c20_4100V1.1 (418) KKVAIRVIAELLSEEEIAGLREMFKMIDTDHSGTITFEEL--------KSGLERVG-SNLVESEIR
Pp3c20_4170V1.1 (418) KKVAIRVIAELLSEEEIAGLREMFKMIDTDHSGTITFEEL--------KSGLERVG-SNLVESEIR
Pp3c23_18930V1.1 (316) KKVAIRVIAEFLSEEEIAGLREMFKMIDTDHSGSITFEEL--------KSGLERVG-SNLVESEIR
Pp3c23_18880V1.1 (316) KKVAIRVIAEFLSEEEIAGLREMFKMIDTDHSGSITFEEL--------KSGLERVG-SNLVESEIR
Pp3c20_12010V1.1 (374) KKLAVRVIAQSMSEEEIAGLRNIFKIMDVDGSGTITFEEL--------KQGLQKVG-SNMREADVR
AT2G17290 calcium dependent protein kinase 6 (372) KKMALKVIAESLSEEEIAGLRAMFEAMDTDNSGAITFDEL--------KAGLRRYG-STLKDTEIR
AT4G35310 calmodulin-domain protein kinase 5 (384) KKMALKVIAESLSEEEIAGLREMFQAMDTDNSGAITFDEL--------KAGLRKYG-STLKDTEIH
AT4G38230 calcium-dependent protein kinase 26 (341) KQMALRVIAESLSEEEIAGLKEMFKAMDTDNSGAITFDEL--------KAGLRRYG-STLKDTEIR
AT1G35670 calcium-dependent protein kinase 11 (313) KKMALRVIAERLSEEEIGGLKELFKMIDTDNSGTITFEEL--------KAGLKRVG-SELMESEIK
AT4G09570 calcium-dependent protein kinase 4 (312) KKMALRVIAERLSEEEIGGLKELFKMIDTDNSGTITFEEL--------KAGLKRVG-SELMESEIK
AT5G23580 calcium-dependent protein kinase 12 (309) KKMALRVIAERLSEEEIGGLKELFKMIDTDKSGTITFEEL--------KDSMRRVG-SELMESEIQ
AT2G35890 calcium-dependent protein kinase 25 (419) KKMALRVIAERLSEEEIHELRETFKTIDSGKSGRVTYKEL--------KNGLERFN-TNLDNSDIN
AT2G38910 calcium-dependent protein kinase 20 (421) KKIAIKVIAESLSEEEIAGLKEMFKMIDTDNSGHITLEEL--------KKGLDRVG-ADLKDSEIL
AT3G10660 calcium-dependent protein kinase 2 (473) KKMALRVIAESLSEEEIAGLKQMFKMIDADNSGQITFEEL--------KAGLKRVG-ANLKESEIL
AT5G04870 calcium dependent protein kinase 1 (437) KKMALRVIAESLSEEEIAGLKEMFNMIDADKSGQITFEEL--------KAGLKRVG-ANLKESEIL
Consensus (529) KKLALRVIAE LSEEEI GLKEMF MDTD SG ITFEEL K GL KVG S L ESEV
EF-hand domain pair

Calcium-binding EF-hand #2 / Calcium-binding EF-hand #3

```
                                                (595) 595  600       610       620       630       640       650       660
AT1G18890 calcium-dependent protein kinase 10 (407) MLMEVA-------DVDGNGFLDYGEFVAVIIHLQKIEN--DELFK-------LAFMFFDKDGSTYI
AT1G74740 calcium-dependent protein kinase 30 (403) LLMEVA-------DVNGNGCLDYGEFVAVIIHLQKMEN--DEHFR-------QAFMFFDKDGSGYI
AT3G51850 calcium-dependent protein kinase 13 (398) MLIEAV-------DTKGKGTLDYGEFVAVSLHLQKVAN--DEHLR-------KAFSYFDKDGNGYI
AT2G41860 calcium-dependent protein kinase 14 (398) ILMDAG-------DVDKDGYLDVNEFVAISVHIRKLGN--DEHLK-------KAFTFFDKNKSGYI
AT3G57530 calcium-dependent protein kinase 32 (407) ILMDAG-------DIDRDGYLDCDEFIAISVHLRKMGN--DEHLK-------KAFAFFDQNNNGYI
AT5G12480 calmodulin-domain protein kinase 7 (403) ILMEAT-------DVDGDGTLNYSEFVAVSVHLKKMAN--DEHLH-------KAFNFFDQNQSGYI
AT5G19450, calcium-dependent protein kinase 8 (402) ILMEAA-------DVDGDGTLNYGEFVAVSVHLKKMAN--DEHLH-------KAFSFFDQNQSDYI
Pp3c11_4640V1.1 (387) MLMEAA-------DVDGNGTLDYGEFVAATVHLQRLDD--DEHLR-------RAFDFFDVDRSGYI
Pp3c7_22440V1.1 (387) MLMGAA-------DVDGNGALDYGEFVAAAVHLQRLGD--DEYLR-------KAFDVFDVDGSGYI
Pp3c11_5760V1.1 (390) MLMEAA-------DVEGNGTLDYGEFVAATVHLQRLDD--DEHLR-------RAFDVFDVDGSGYI
Pp3c11_5820V1.1  (389) MLMEAA-------DVEGNGTLDYGEFVAATVHLQRLDD--DEHLR-------RAFDVFDVDGSGYI
Pp3c7_22710V1.1 (390) MLMEAA-------DVDGNRTLDYGEFVAATVHLQRLDD--DDHLR-------RAFDVFDVNESGFI
Pp3c7_25180V1.1  (389) LLMEAA-------DVDGNGTLDYGEFVAAAVHLQRLDD--DEHLR-------KAFDVFDVNESGFI
AT2G31500 calcium-dependent protein kinase 24 (410) MLMDAA-------DTDGNGMLSCDEFVTLSIHLKRMGC--DEHLQ-------EAFKYFDKNGNGFI
AT2G17890 calcium-dependent protein kinase 16 (455) EILQA-------IDSNTDGFVDFGEFVAAALHVNQLEEHDSEKWQQRSR---AAFEKFDIDGDGFI
AT4G36070 calcium-dependent protein kinase 18 (418) EILQA-------NDSNTDGLVDFTEFVVAALHVNQLEEHDSEKWQQRSR---AAFDKFDIDGDGFI
AT5G66210 calcium-dependent protein kinase 28 (409) EILEA-------IDSNTDGLVDFTEFVAAALHVHQLEEHDSEKWQLRSR---AAFEKFDLDKDGYI
Pp3c15_3590V1.1 (484) EILQA-------MDSNRDGIVDFNEFVAATLHVHQLEETDSEKWQKRSR---AAFSKFDFDGDGYI
Pp3c9_4620V1.1 (473) EILQA-------MDSNSDGLVDFDEFVAATLHVHQLEETDSEKWQSRSQ---AAFSQFDFDGDGYI
Pp3c15_5120V1.1 (477) EILQA-------MDSNADGMIDFDEFVAATLHVHQLEQANSAKWQQRSK---AAFSKFDVDGDGFI
Pp3c9_5860V1.1 (437) EILQA-------MDSNADGIVDFDEFVAATLHVHQLEQSNTTKWQHRSK---AAFSKFDVDGDGYI
Pp3c13_4100V1.1 (490) DILNS-------MDALSLKKMDFSEFCAAAISVHQLEG--TDRWEQHAR---AAFDIFEKEGNRSI
Pp3c3_5990V1.1 (490) EILNS-------MNSLSHKKMDFTEFSAAAISVHQLEG--TDRWERHAR---AAYDIFDKEGNRVI
Pp3c3_5970V1.1 (486) EILTS-------MDALSFKKMDLSEFCAAAISVHQLEG--TDRWEQHAR---AAYDIFEKEGNRVI
Pp3c26_4540V1.1 (492) EVLIS-------MDGLNFKKMDFSEFCAAAISVHHLEA--TDRWDQRAR---AAYDIFEKEGNRVI
Pp3c4_25010V1.1  (491) EILES-------MDGLHFKKMDFSEFCAAAISVLQLEA--TERWEQHAR---AAYDIFEKEGNRVI
Pp3c19_20580V1.1 (380) GLMPLVSRIFELFDSNHDGWIDLREVMCGFSLLRTSHAGEVLR---------LCSRMYDYDDSGYI
Pp3c21_15330V1.1 (381) CFVPLAPRIFELFDYNHDGGIDLREVVCGFSLLRTSHLDDALQ---------MCFKIYDRDDSGYI
AT1G50700 calcium-dependent protein kinase 33 (417) QLMDAA-------DVDGNGSIDYIEFITATMHRHRLES--NEN-------VYKAFQHFDKDGSGYI
AT3G20410 calmodulin-domain protein kinase 9 (435) QLMDAA-------DVDGNGSIDYIEFITATMHRHRLES--NEN-------LYKAFQHFDKDSSGYI
AT1G61950 calcium-dependent protein kinase 19 (443) QLLEDA-------DVDGNGTIDYIEFISATMNRFRVER--EDN-------LFKAFQHFDKDNSGFI
AT4G04695 calcium-dependent protein kinase 31 (375) QLMEAA-------DVDGNGTIDIDEFISATMHRYRLDR--DDH-------VYQAFQHFDKDNDGHI
AT4G04700 calcium-dependent protein kinase 27 (375) QLMEAA-------DMDGNGTIDIDEFISATMHRYKLDR--DEH-------VYKAFQHFDKDNDGHI
AT4G04710 calcium-dependent protein kinase 22 (375) QLMEAVS-----ADVDGNGTIDYIEFISATMHRHRLER--DEH-------LYKAFQYFDKDGSGHI
AT4G04720 calcium-dependent protein kinase 21 (423) QLMEAA-------DVDGNGTIDYYEFISATMHRYKLDR--DEH-------VYKAFQHFDKDNSGHI
AT4G04740 calcium-dependent protein kinase 23 (412) QLVEAS-------DVDGNGTIDYYEFISATMHRYKLHH--DEH-------VHKAFQHLDKDKNGHI
```

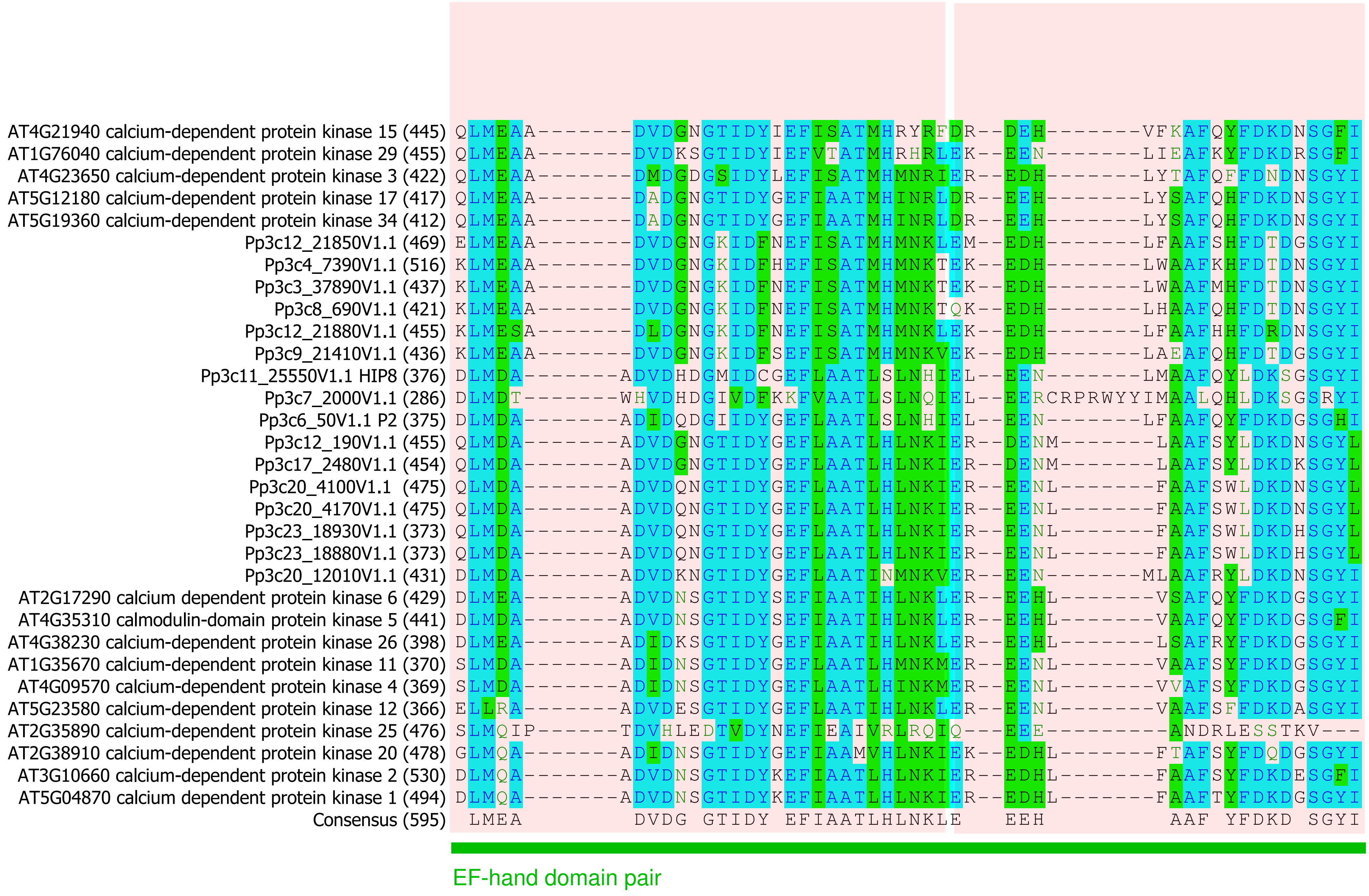
AT4G21940 calcium-dependent protein kinase 15 (445) QLMEAA-------DVDGNGTIDYIEFISATMHRYRFDR--DEH-------VFKAFQYFDKDNSGFI
AT1G76040 calcium-dependent protein kinase 29 (455) QLMEAA-------DVDKSGTIDYIEFVTATMHRHRLEK--EEN-------LIEAFKYFDKDRSGFI
AT4G23650 calcium-dependent protein kinase 3 (422) QLMEAA-------DMDGDGSIDYLEFISATMHMNRIER--EDH-------LYTAFQFFDNDNSGYI
AT5G12180 calcium-dependent protein kinase 17 (417) QLMEAA-------DADGNGTIDYGEFIAATMHINRLDR--EEH-------LYSAFQHFDKDNSGYI
AT5G19360 calcium-dependent protein kinase 34 (412) QLMEAA-------DADGNGTIDYGEFIAATMHINRLDR--EEH-------LYSAFQHFDKDNSGYI
Pp3c12_21850V1.1 (469) ELMEAA-------DVDGNGKIDFNEFISATMHMNKLEM--EDH-------LFAAFSHFDTDGSGYI
Pp3c4_7390V1.1 (516) KLMEAA-------DVDGNGKIDFHEFISATMHMNKTEK--EDH-------LWAAFKHFDTDNSGYI
Pp3c3_37890V1.1 (437) KLMEAA-------DVDGNGKIDFNEFISATMHMNKTEK--EDH-------LWAAFMHFDTDNSGYI
Pp3c8_690V1.1 (421) KLMEAA-------DVDGNGKIDFNEFISATMHMNKTQK--EDH-------LHAAFQHFDTDNSGYI
Pp3c12_21880V1.1 (455) KLMESA-------DLDGNGKIDFNEFISATMHMNKLEK--EDH-------LFAAFHHFDRDNSGYI
Pp3c9_21410V1.1 (436) KLMEAA-------DVDGNGKIDFSEFISATMHMNKVEK--EDH-------LAEAFQHFDTDGSGYI
Pp3c11_25550V1.1 HIP8 (376) DLMDA-------ADVDHDGMIDCGEFLAATLSLNHIEL--EEN-------LMAAFQYLDKSGSGYI
Pp3c7_2000V1.1 (286) DLMDT-------WHVDHDGIVDFKKFVAATLSLNQIEL--EERCRPRWYYIMAALQHLDKSGSRYI
Pp3c6_50V1.1 P2 (375) DLMDA-------ADIDQDGIIDYGEFLAATLSLNHIEL--EEN-------LFAAFQYFDKDGSGHI
Pp3c12_190V1.1 (455) QLMDA-------ADVDGNGTIDYGEFLAATLHLNKIER--DENM-------LAAFSYLDKDNSGYL
Pp3c17_2480V1.1 (454) QLMDA-------ADVDGNGTIDYGEFLAATLHLNKIER--DENM-------LAAFSYLDKDKSGYL
Pp3c20_4100V1.1 (475) QLMDA-------ADVDQNGTIDYGEFLAATLHLNKIER--EENL-------FAAFSWLDKDNSGYL
Pp3c20_4170V1.1 (475) QLMDA-------ADVDQNGTIDYGEFLAATLHLNKIER--EENL-------FAAFSWLDKDNSGYL
Pp3c23_18930V1.1 (373) QLMDA-------ADVDQNGTIDYGEFLAATLHLNKIER--EENL-------FAAFSWLDKDHSGYL
Pp3c23_18880V1.1 (373) QLMDA-------ADVDQNGTIDYGEFLAATLHLNKIER--EENL-------FAAFSWLDKDHSGYL
Pp3c20_12010V1.1 (431) DLMDA-------ADVDKNGTIDYGEFLAATINMNKVER--EEN-------MLAAFRYLDKDNSGYI
AT2G17290 calcium dependent protein kinase 6 (429) DLMEA-------ADVDNSGTIDYSEFIAATIHLNKLER--EEHL-------VSAFQYFDKDGSGYI
AT4G35310 calmodulin-domain protein kinase 5 (441) DLMDA-------ADVDNSGTIDYSEFIAATIHLNKLER--EEHL-------VAAFQYFDKDGSGFI
AT4G38230 calcium-dependent protein kinase 26 (398) DLMEA-------ADIDKSGTIDYGEFIAATIHLNKLER--EEHL-------LSAFRYFDKDGSGYI
AT1G35670 calcium-dependent protein kinase 11 (370) SLMDA-------ADIDNSGTIDYGEFLAATLHMNKMER--EENL-------VAAFSYFDKDGSGYI
AT4G09570 calcium-dependent protein kinase 4 (369) SLMDA-------ADIDNSGTIDYGEFLAATLHINKMER--EENL-------VVAFSYFDKDGSGYI
AT5G23580 calcium-dependent protein kinase 12 (366) ELLRA-------ADVDESGTIDYGEFLAATIHLNKLER--EENL-------VAAFSFFDKDASGYI
AT2G35890 calcium-dependent protein kinase 25 (476) SLMQIP------TDVHLEDTVDYNEFIEAIVRLRQIQ---EEE---------ANDRLESSTKV---
AT2G38910 calcium-dependent protein kinase 20 (478) GLMQA-------ADIDNSGTIDYGEFIAAMVHLNKIEK--EDHL-------FTAFSYFDQDGSGYI
AT3G10660 calcium-dependent protein kinase 2 (530) DLMQA-------ADVDNSGTIDYKEFIAATLHLNKIER--EDHL-------FAAFSYFDKDESGFI
AT5G04870 calcium dependent protein kinase 1 (494) DLMQA-------ADVDNSGTIDYKEFIAATLHLNKIER--EDHL-------FAAFTYFDKDGSGYI
Consensus (595) LMEA DVDG GTIDY EFIAATLHLNKLE EEH AAF YFDKD SGYI
EF-hand domain pair

Calcium-binding EF-hand #3 (positions 661–673); Calcium-binding EF-hand #4 (positions 680–726)

```
                                              (661) 661      670       680       690       700       710            726
AT1G18890 calcium-dependent protein kinase 10 (457) ELDELREALADELG--EPD-A--SVLSDIMREVDTDKDG---------RINYDEFVTMMKAGTDWR
AT1G74740 calcium-dependent protein kinase 30 (453) ESEELREALTDELG--EPD-N--SVIIDIMREVDTDKDG---------KINYDEFVVMMKAGTDWR
AT3G51850 calcium-dependent protein kinase 13 (448) LPQELCDALKEDGG--DDC-V--DVANDIFQEVDTDKDG---------RISYEEFAAMMKTGTDWR
AT2G41860 calcium-dependent protein kinase 14 (448) EIEELRDALADDVDT-TS--E--EVVEAIILDVDTNKDG---------KISYDEFATMMKTGTDWR
AT3G57530 calcium-dependent protein kinase 32 (457) EIEELREALSDELG--TS--E--EVVDAIIRDVDTDKDG---------RISYEEFVTMMKTGTDWR
   AT5G12480 calmodulin-domain protein kinase 7 (453) EIDELREALNDELDN-TSS-E--EVIAAIMQDVDTDKDG---------RISYEEFVAMMKAGTDWR
 AT5G19450, calcium-dependent protein kinase 8 (452) EIEELREALNDEVDT-NS--E--EVVAAIMQDVDTDKDG---------RISYEEFAAMMKAGTDWR
                              Pp3c11_4640V1.1 (437) ETEELREAVGEPLNGSPSE-T--DVVQAILLEVDIDKDG---------RISYEEFATMMRRGTDWR
                              Pp3c7_22440V1.1 (437) ETEELRVAVGEPLNGSPSE-S--DVVQGILLEVDVDKDG---------RISYEEFSAMMRRGTDWR
                              Pp3c11_5760V1.1 (440) ETEELREAVGEAMTELSSE-P--DVVQAILSEVDLDKDG---------RISYEEFAVMMRRGTDWR
                              Pp3c11_5820V1.1 (439) ETEELREAVGEAMTELSSE-P--DVVQAILSEVDLDKDG---------RISYEEFAVMMRRGTDWR
                              Pp3c7_22710V1.1 (440) EVEELREAVGESLMGSSSE-S--DVVQAILSEVDLDKDG---------RISYEEFAMMMRRGTDWR
                              Pp3c7_25180V1.1 (439) EVEELREAVGESLTGSPSE-S--DVVQGILSEVDLDKDG---------RISYEEFATMMRRGTDWR
AT2G31500 calcium-dependent protein kinase 24 (460) ELDELKVALCDDKLGHANGND--QWIKDIFFDVDLNKDG---------RISFDEFKAMMKSGTDWK
AT2G17890 calcium-dependent protein kinase 16 (511) TAEELRMHTGLKGSIEP-----------LLEEADIDNDG---------KISLQEFRRLLRTASIKS
AT4G36070 calcium-dependent protein kinase 18 (474) TPEELRLQTGLKGSIEP-----------LLEEADVDEDG---------RISINEFRRLLRSASLKS
AT5G66210 calcium-dependent protein kinase 28 (465) TPEELRMHTGLRGSIDP-----------LLDEADIDRDG---------KISLHEFRRLLRTASISS
                              Pp3c15_3590V1.1 (540) TTEELKIATGLKGSMDS-----------FLGEADIDGDG---------RISLPEFQKLLRQASLGS
                               Pp3c9_4620V1.1 (529) TADELKIATGLNGSMDS-----------ILVEADIDGDG---------KISLSEFQKLLRQASLGS
                              Pp3c15_5120V1.1 (533) TAEELKIATGLKGSVGT-----------LLEEADSDKDG---------RISLSEFQKLLHQASFGS
                               Pp3c9_5860V1.1 (493) TAEELKIATGIKGSVET-----------LLEEADVDGDK---------RISLPEFQKLLHQASFGS
                              Pp3c13_4100V1.1 (544) SVEELAREVGLAPNVPAQ----------VFHEWLRHSDG---------RLSFVGFTKLLHGITTRT
                               Pp3c3_5990V1.1 (544) SVEELAHEVGLATTVPAQ----------VFHEWLRHSDG---------RLSFVGFTKLLHGVTTRT
                               Pp3c3_5970V1.1 (540) SADELAKEMGLAPTVPAQ----------VFQEWVRHSDG---------RLSFIGFTKLLHGVTFRP
                              Pp3c26_4540V1.1 (546) NYDELAKEMKLPPTVPAQ----------LFLDWIRQSDG---------RLSFTGFTKLLHGISSRA
                              Pp3c4_25010V1.1 (545) YPDELAKEMGLAPNVPAQ----------VFLDWIRQSDG---------RLSFTGFTKLLHGISSRA
                             Pp3c19_20580V1.1 (437) SKDELALVLGALPEDYLPPDISEVGMDEIFEHIDTDCDG---------RISYQEFEQALCLQD---
                             Pp3c21_15330V1.1 (438) SKGELARVLGALPEDYLPADISQVQMDDIFEHVDTDSDG---------RISYQEFRRALSLQD---
AT1G50700 calcium-dependent protein kinase 33 (467) TTDELEAALKEYGMGDDAT------IKEILSDVDADNDG---------RINYDEFCAMMRSGN---
   AT3G20410 calmodulin-domain protein kinase 9 (485) TIDELESALKEYGMGDDAT------IKEVLSDVDSDNDG---------RINYEEFCAMMRSGN---
AT1G61950 calcium-dependent protein kinase 19 (493) SRQELETAMKEYNMGDDIM------IKEIISEVDADNDG---------SINYQEFCNMMKSCS---
AT4G04695 calcium-dependent protein kinase 31 (425) TKEELEMAMKEHGVGDEVS------IKQIITEVDTDNDG---------KINFEEFRTMMRSGS---
AT4G04700 calcium-dependent protein kinase 27 (425) TKEELEMAMKEDGAGDEGS------IKQIIADADTDNDG---------KINFEEFRTMMRTES---
AT4G04710 calcium-dependent protein kinase 22 (427) TKEEVEIAMKEHGMGDEAN------AKDLISEFDKNNDG---------KIDYEEFCTMMRNG----
AT4G04720 calcium-dependent protein kinase 21 (473) TRDELESAMKEYGMGDEAS------IKEVISEVDTDNDG---------RINFEEFCAMMRSGS---
AT4G04740 calcium-dependent protein kinase 23 (462) TRDELESAMKEYGMGDEAS------IKEVISEVDTDNALSPVEIMREVALEIGVPVNTFKQNN---
```

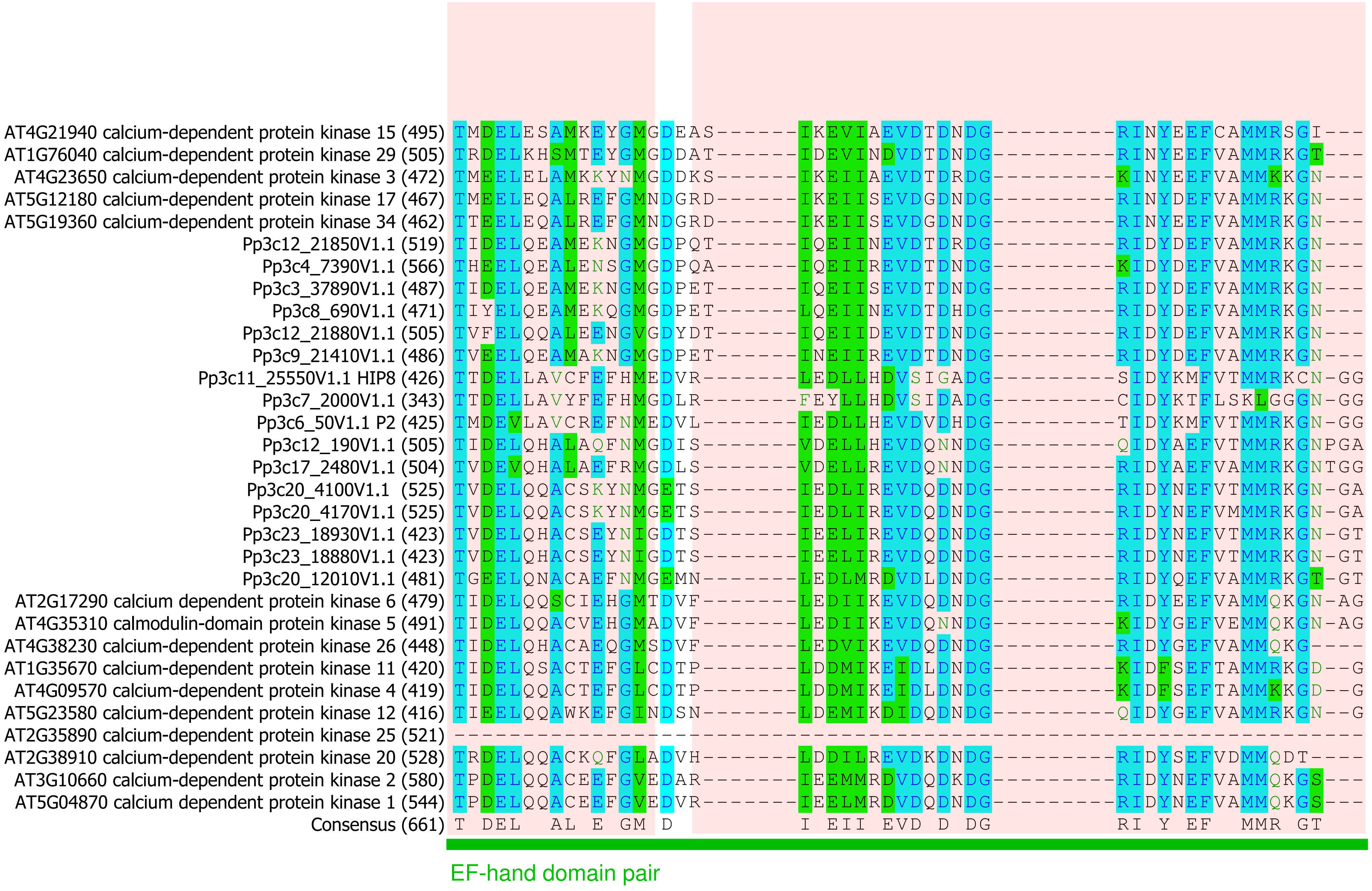
AT4G21940 calcium-dependent protein kinase 15 (495) TMDELESAMKEYGMGDEAS------IKEVIAEVDTDNDG---------RINYEEFCAMMRSGI---
AT1G76040 calcium-dependent protein kinase 29 (505) TRDELKHSMTEYGMGDDAT------IDEVINDVDTDNDG---------RINYEEFVAMMRKGT---
AT4G23650 calcium-dependent protein kinase 3 (472) TMEELELAMKKYNMGDDKS------IKEIIAEVDTDRDG---------KINYEEFVAMMKKGN---
AT5G12180 calcium-dependent protein kinase 17 (467) TMEELEQALREFGMNDGRD------IKEIISEVDGDNDG---------RINYDEFVAMMRKGN---
AT5G19360 calcium-dependent protein kinase 34 (462) TTEELEQALREFGMNDGRD------IKEIISEVDGDNDG---------RINYEEFVAMMRKGN---
Pp3c12_21850V1.1 (519) TIDELQEAMEKNGMGDPQT------IQEIINEVDTDRDG---------RIDYDEFVAMMRKGN---
Pp3c4_7390V1.1 (566) THEELQEALENSGMGDPQA------IQEIIREVDTDNDG---------KIDYDEFVAMMRKGN---
Pp3c3_37890V1.1 (487) TIDELQEAMEKNGMGDPET------IQEIISEVDTDNDG---------RIDYDEFVAMMRKGN---
Pp3c8_690V1.1 (471) TIYELQEAMEKQGMGDPET------LQEIINEVDTDHDG---------RIDYDEFVAMMRKGN---
Pp3c12_21880V1.1 (505) TVFELQQALEENGVGDYDT------IQEIIDEVDTDNDG---------RIDYDEFVAMMRKGN---
Pp3c9_21410V1.1 (486) TVEELQEAMAKNGMGDPET------INEIIREVDTDNDG---------RIDYDEFVAMMRKGN---
Pp3c11_25550V1.1 HIP8 (426) TTDELLAVCFEFHMEDVR-------LEDLLHDVSIGADG---------SIDYKMFVTMMRKCN-GG
Pp3c7_2000V1.1 (343) TTDELLAVYFEFHMGDLR-------FEYLLHDVSIDADG---------CIDYKTFLSKLGGGN-GG
Pp3c6_50V1.1 P2 (425) TMDEVLAVCREFNMEDVL-------IEDLLHEVDVDHDG---------TIDYKMFVTMMRKGN-GG
Pp3c12_190V1.1 (505) TIDELQHALAQFNMGDIS-------VDELLHEVDQNNDG---------QIDYAEFVTMMRKGNPGA
Pp3c17_2480V1.1 (504) TVDEVQHALAEFRMGDLS-------VDELLREVDQNNDG---------RIDYAEFVAMMRKGNTGG
Pp3c20_4100V1.1 (525) TVDELQQACSKYNMGETS-------IEDLIREVDQDNDG---------RIDYNEFVTMMRKGN-GA
Pp3c20_4170V1.1 (525) TVDELQQACSKYNMGETS-------IEDLIREVDQDNDG---------RIDYNEFVMMMRKGN-GA
Pp3c23_18930V1.1 (423) TVDELQHACSEYNIGDTS-------IEELIREVDQDNDG---------RIDYNEFVTMMRKGN-GT
Pp3c23_18880V1.1 (423) TVDELQHACSEYNIGDTS-------IEELIREVDQDNDG---------RIDYNEFVTMMRKGN-GT
Pp3c20_12010V1.1 (481) TGEELQNACAEFNMGEMN-------LEDLMRDVDLDNDG---------RIDYQEFVAMMRKGT-GT
AT2G17290 calcium dependent protein kinase 6 (479) TIDELQQSCIEHGMTDVF-------LEDIIKEVDQDNDG---------RIDYEEFVAMMQKGN-AG
AT4G35310 calmodulin-domain protein kinase 5 (491) TIDELQQACVEHGMADVF-------LEDIIKEVDQNNDG---------KIDYGEFVEMMQKGN-AG
AT4G38230 calcium-dependent protein kinase 26 (448) TIDELQHACAEQGMSDVF-------LEDVIKEVDQDNDG---------RIDYGEFVAMMQKG----
AT1G35670 calcium-dependent protein kinase 11 (420) TIDELQSACTEFGLCDTP-------LDDMIKEIDLDNDG---------KIDFSEFTAMMRKGD--G
AT4G09570 calcium-dependent protein kinase 4 (419) TIDELQQACTEFGLCDTP-------LDDMIKEIDLDNDG---------KIDFSEFTAMMKKGD--G
AT5G23580 calcium-dependent protein kinase 12 (416) TIEELQQAWKEFGINDSN-------LDEMIKDIDQDNDG---------QIDYGEFVAMMRKGN--G
AT2G35890 calcium-dependent protein kinase 25 (521) ------------------------------------------------------------------
AT2G38910 calcium-dependent protein kinase 20 (528) TRDELQQACKQFGLADVH-------LDDILREVDKDNDG---------RIDYSEFVDMMQDT----
AT3G10660 calcium-dependent protein kinase 2 (580) TPDELQQACEEFGVEDAR-------IEEMMRDVDQDKDG---------RIDYNEFVAMMQKGS---
AT5G04870 calcium dependent protein kinase 1 (544) TPDELQQACEEFGVEDVR-------IEELMRDVDQDNDG---------RIDYNEFVAMMQKGS---
Consensus (661) T DEL AL E GM D I EII EVD D DG RI Y EF MMR GT
EF-hand domain pair

```
                                               (727) 727          740       750       760       770       780         792
AT1G18890 calcium-dependent protein kinase 10 (509) KASRQYSRERFKSLSINLMKDGSLHLHDALTGQTVPV-----------------------------
AT1G74740 calcium-dependent protein kinase 30 (505) KASRQYSRERFKSLSLNLMKDGSMHLHDALTGQSIAV-----------------------------
AT3G51850 calcium-dependent protein kinase 13 (500) KASRHYSRGRFNSLSIKLMKDGSLNLGNE-------------------------------------
AT2G41860 calcium-dependent protein kinase 14 (500) KASRQYSRDLFKCLSLKLMQDGSLQSNGDTK-----------------------------------
AT3G57530 calcium-dependent protein kinase 32 (508) KASRQYSRERFNSISLKLMQDASLQVNGDTR-----------------------------------
 AT5G12480 calmodulin-domain protein kinase 7 (506) KASRQYSRERFNSLSLKLMRDGSLQLEGET------------------------------------
 AT5G19450, calcium-dependent protein kinase 8 (504) KASRQYSRERFNSLSLKLMREGSLQLEGEN------------------------------------
                           Pp3c11_4640V1.1 (491) KASRQYSRDRFNSLSMRLLRDGSLNPDKIVAVR---------------------------------
                           Pp3c7_22440V1.1 (491) KASRQYSRDRFNSLSMRLVREGSLNPDKSVAVR---------------------------------
                           Pp3c11_5760V1.1 (494) KASRQYSRDRFNSLSMRLLRDGSLNPPSYSMSSMR-------------------------------
                           Pp3c11_5820V1.1 (493) KASRQYSRDRFNSLSMRLLRDGSLNPPSYSMSSMR-------------------------------
                           Pp3c7_22710V1.1 (494) KASRQYSRDRFNSLSTRLLRDGSLNPSSYSTR----------------------------------
                           Pp3c7_25180V1.1 (493) KASRQYSRDRFNSLSMRLLRDGSLNPPSYSMR----------------------------------
AT2G31500 calcium-dependent protein kinase 24 (515) MASRQYSRALLNALSIKMFKEDFGDNGPKSHSMEFPIARKRAKLLDAPKNKSMELQISKTYKPSGL
AT2G17890 calcium-dependent protein kinase 16 (557) RNVRSPPG--YLISRKV-------------------------------------------------
AT4G36070 calcium-dependent protein kinase 18 (520) KNVKSPPGTEHIICHNLLDGICIEDTEERTSAVRFEYVSQVL------------------------
AT5G66210 calcium-dependent protein kinase 28 (511) QRAPSPAGHRNLR-----------------------------------------------------
                           Pp3c15_3590V1.1 (586) RNNSEHQAPVSHNPRRREAHHHHVAS----------------------------------------
                            Pp3c9_4620V1.1 (575) RTN-EHHTLVTHNHRKC-------------------------------------------------
                           Pp3c15_5120V1.1 (579) RTNTDHNRQRT-------------------------------------------------------
                            Pp3c9_5860V1.1 (539) RTNTDHNRHRT-------------------------------------------------------
                           Pp3c13_4100V1.1 (591) NRNHH-------------------------------------------------------------
                            Pp3c3_5990V1.1 (591) NRNHH-------------------------------------------------------------
                            Pp3c3_5970V1.1 (587) VRNQQQ------------------------------------------------------------
                           Pp3c26_4540V1.1 (593) IRNYQH------------------------------------------------------------
                           Pp3c4_25010V1.1 (592) IKNLQQ------------------------------------------------------------
                          Pp3c19_20580V1.1 (491) ----------AIMNKFRIPENREVN-----------------------------------------
                          Pp3c21_15330V1.1 (492) ----------AVMNHFRMEGQVQ-------------------------------------------
AT1G50700 calcium-dependent protein kinase 33 (515) ---------PQQ--PRLF------------------------------------------------
 AT3G20410 calmodulin-domain protein kinase 9 (533) ---------PQQQQPRLF------------------------------------------------
AT1G61950 calcium-dependent protein kinase 19 (541) ---------QSHQSKLVQPN----------------------------------------------
AT4G04695 calcium-dependent protein kinase 31 (473) ---------SLQPQRELLPIK---------------------------------------------
AT4G04700 calcium-dependent protein kinase 27 (473) ---------SLQPEGELLPIIN--------------------------------------------
AT4G04710 calcium-dependent protein kinase 22 (474) ---------ILQPQGKLLKRLYMNLEELKTGLTRLGSRLSETEIDKAFQHFDKDNSGHITRDELES
AT4G04720 calcium-dependent protein kinase 21 (521) ---------TQPQ-GKLLPFH---------------------------------------------
AT4G04740 calcium-dependent protein kinase 23 (519) ---------VQED-GLYLPVLNNAA-----------------------------------------
```

```
AT4G21940 calcium-dependent protein kinase 15 (543) ---------TLPQQGKILPPCKRVADLN--------------------------------------
AT1G76040 calcium-dependent protein kinase 29 (553) ---------TDSDPKLIR------------------------------------------------
AT4G23650 calcium-dependent protein kinase 3 (520)  ---------P------ELVPNRRRM-----------------------------------------
AT5G12180 calcium-dependent protein kinase 17 (515) ---------P------DPIPKKRRELSFK-------------------------------------
AT5G19360 calcium-dependent protein kinase 34 (510) ---------P------DPNPKKRRELSFK-------------------------------------
Pp3c12_21850V1.1 (567)                            ---------PITEDG---GKHRHR------------------------------------------
Pp3c4_7390V1.1 (614)                              ---------PDTEDGVMVVPPRHR------------------------------------------
Pp3c3_37890V1.1 (535)                             ---------PGAENGGTVNKPRHR------------------------------------------
Pp3c8_690V1.1 (519)                               ---------PGTEDGGNLKSPRHRW-----------------------------------------
Pp3c12_21880V1.1 (553)                            ---------PGAEEG-EKHNHRHRY-----------------------------------------
Pp3c9_21410V1.1 (534)                             ------------E-VPQMTTRRHRR-----------------------------------------
Pp3c11_25550V1.1 HIP8 (475)                       MGHQN----LRCTLGITDVLTLEEQY----------------------------------------
Pp3c7_2000V1.1 (392)                              MGHQN----VRCIPGITNVLSLEVQYWSPLFFFVHLG-----------------------------
Pp3c6_50V1.1 P2 (474)                             VGHQT----LRCTLGITDVLAHDMT-----------------------------------------
Pp3c12_190V1.1 (555)                              AGR----SSFRNSQSLSLNDVLMMG-----------------------------------------
Pp3c17_2480V1.1 (554)                             AGR----SSLRNSQSLSLNDVLMVG-----------------------------------------
Pp3c20_4100V1.1  (574)                            VGR----TTLRN--SLSLSDALMNPQ----------------------------------------
Pp3c20_4170V1.1 (574)                             VGR----TTLRN--SLSLSDALMNPQ----------------------------------------
Pp3c23_18930V1.1 (472)                            VGR----ATLRN--SLSLSDALMHTN----------------------------------------
Pp3c23_18880V1.1 (472)                            VGR----ATLRN--SLRMGNGKATAAVDGSERGGASAAMSVQRCGGVDSIRENAARNSKGVGGEGG
Pp3c20_12010V1.1 (530)                            APPQRG---ARMEPSFGFSAALQL------------------------------------------
AT2G17290 calcium dependent protein kinase 6 (528) VGR----RTMKNSLNISMRDV---------------------------------------------
AT4G35310 calmodulin-domain protein kinase 5 (540) VGR----RTMRNSLNISMRDA---------------------------------------------
AT4G38230 calcium-dependent protein kinase 26 (494) IVG----RTMRKSINMSIRNNAVSQ-----------------------------------------
AT1G35670 calcium-dependent protein kinase 11 (468) VGR---SRTMMKNLNFNIADAFGVDGE----KSDD-------------------------------
AT4G09570 calcium-dependent protein kinase 4 (467)  VGR---SRTMRNNLNFNIAEAFGVEDTSSTAKSDDSPK----------------------------
AT5G23580 calcium-dependent protein kinase 12 (464) TGGGIGRRTMRNSLNFGTTLPDESMNV---------------------------------------
AT2G35890 calcium-dependent protein kinase 25 (521) ------------------------------------------------------------------
AT2G38910 calcium-dependent protein kinase 20 (574) -----------GFGKMGLKVS---------------------------------------------
AT3G10660 calcium-dependent protein kinase 2 (627)  I--------MGGPVKMGLENSISISLKH--------------------------------------
AT5G04870 calcium dependent protein kinase 1 (591)  I--------TGGPVKMGLEKSFSIALKL--------------------------------------
Consensus (727)                                                    L
```

EF-hand domain pair

| (793) | 793 800 810 820 837 |
| --- | --- |
| AT1G18890 calcium-dependent protein kinase 10 (546) | --------------------------------------------- |
| AT1G74740 calcium-dependent protein kinase 30 (542) | --------------------------------------------- |
| AT3G51850 calcium-dependent protein kinase 13 (529) | --------------------------------------------- |
| AT2G41860 calcium-dependent protein kinase 14 (531) | --------------------------------------------- |
| AT3G57530 calcium-dependent protein kinase 32 (539) | --------------------------------------------- |
| AT5G12480 calmodulin-domain protein kinase 7 (536) | --------------------------------------------- |
| AT5G19450, calcium-dependent protein kinase 8 (534) | --------------------------------------------- |
| Pp3c11_4640V1.1 (524) | --------------------------------------------- |
| Pp3c7_22440V1.1 (524) | --------------------------------------------- |
| Pp3c11_5760V1.1 (529) | --------------------------------------------- |
| Pp3c11_5820V1.1 (528) | --------------------------------------------- |
| Pp3c7_22710V1.1 (526) | --------------------------------------------- |
| Pp3c7_25180V1.1 (525) | --------------------------------------------- |
| AT2G31500 calcium-dependent protein kinase 24 (581) | RN------------------------------------------- |
| AT2G17890 calcium-dependent protein kinase 16 (572) | --------------------------------------------- |
| AT4G36070 calcium-dependent protein kinase 18 (562) | --------------------------------------------- |
| AT5G66210 calcium-dependent protein kinase 28 (524) | --------------------------------------------- |
| Pp3c15_3590V1.1 (612) | --------------------------------------------- |
| Pp3c9_4620V1.1 (591) | --------------------------------------------- |
| Pp3c15_5120V1.1 (590) | --------------------------------------------- |
| Pp3c9_5860V1.1 (550) | --------------------------------------------- |
| Pp3c13_4100V1.1 (596) | --------------------------------------------- |
| Pp3c3_5990V1.1 (596) | --------------------------------------------- |
| Pp3c3_5970V1.1 (593) | --------------------------------------------- |
| Pp3c26_4540V1.1 (599) | --------------------------------------------- |
| Pp3c4_25010V1.1 (598) | --------------------------------------------- |
| Pp3c19_20580V1.1 (506) | --------------------------------------------- |
| Pp3c21_15330V1.1 (505) | --------------------------------------------- |
| AT1G50700 calcium-dependent protein kinase 33 (522) | --------------------------------------------- |
| AT3G20410 calmodulin-domain protein kinase 9 (542) | --------------------------------------------- |
| AT1G61950 calcium-dependent protein kinase 19 (552) | --------------------------------------------- |
| AT4G04695 calcium-dependent protein kinase 31 (485) | --------------------------------------------- |
| AT4G04700 calcium-dependent protein kinase 27 (486) | --------------------------------------------- |
| AT4G04710 calcium-dependent protein kinase 22 (531) | AMKEYGMGDEASIKEVISEVDTDNVSCTLQHIANISNIKQVLETL |
| AT4G04720 calcium-dependent protein kinase 21 (532) | --------------------------------------------- |
| AT4G04740 calcium-dependent protein kinase 23 (534) | --------------------------------------------- |

| | |
|---|---|
| AT4G21940 calcium-dependent protein kinase 15 (562) | --------------------------------------------- |
| AT1G76040 calcium-dependent protein kinase 29 (562) | --------------------------------------------- |
| AT4G23650 calcium-dependent protein kinase 3 (530) | --------------------------------------------- |
| AT5G12180 calcium-dependent protein kinase 17 (529) | --------------------------------------------- |
| AT5G19360 calcium-dependent protein kinase 34 (524) | --------------------------------------------- |
| Pp3c12_21850V1.1 (579) | --------------------------------------------- |
| Pp3c4_7390V1.1 (629) | --------------------------------------------- |
| Pp3c3_37890V1.1 (550) | --------------------------------------------- |
| Pp3c8_690V1.1 (535) | --------------------------------------------- |
| Pp3c12_21880V1.1 (568) | --------------------------------------------- |
| Pp3c9_21410V1.1 (546) | --------------------------------------------- |
| Pp3c11_25550V1.1 HIP8 (497) | --------------------------------------------- |
| Pp3c7_2000V1.1 (425) | --------------------------------------------- |
| Pp3c6_50V1.1 P2 (495) | --------------------------------------------- |
| Pp3c12_190V1.1 (576) | --------------------------------------------- |
| Pp3c17_2480V1.1 (575) | --------------------------------------------- |
| Pp3c20_4100V1.1 (594) | --------------------------------------------- |
| Pp3c20_4170V1.1 (594) | --------------------------------------------- |
| Pp3c23_18930V1.1 (492) | --------------------------------------------- |
| Pp3c23_18880V1.1 (532) | RVCWLSGRGGLETMPRLGTVGVHEVKNGGLERAAEQGVG------ |
| Pp3c20_12010V1.1 (551) | --------------------------------------------- |
| AT2G17290 calcium dependent protein kinase 6 (545) | --------------------------------------------- |
| AT4G35310 calmodulin-domain protein kinase 5 (557) | --------------------------------------------- |
| AT4G38230 calcium-dependent protein kinase 26 (515) | --------------------------------------------- |
| AT1G35670 calcium-dependent protein kinase 11 (496) | --------------------------------------------- |
| AT4G09570 calcium-dependent protein kinase 4 (502) | --------------------------------------------- |
| AT5G23580 calcium-dependent protein kinase 12 (491) | --------------------------------------------- |
| AT2G35890 calcium-dependent protein kinase 25 (521) | --------------------------------------------- |
| AT2G38910 calcium-dependent protein kinase 20 (584) | --------------------------------------------- |
| AT3G10660 calcium-dependent protein kinase 2 (647) | --------------------------------------------- |
| AT5G04870 calcium dependent protein kinase 1 (611) | --------------------------------------------- |
| Consensus (793) | |
