# Supplementary material for: Holophytochrome-Interacting Proteins in Physcomitrella: Putative Actors in Phytochrome Cytoplasmic Signaling
Source: Front Plant Sci. 2016 May 12;7:613. doi: 10.3389/fpls.2016.00613 (PMC4867686; doi:10.3389/fpls.2016.00613)
Supplement: Supplementary file 2 [file Data_Sheet_2.ZIP › SI/SI HIP9.pdf]

frontiers

Supplementary Material

Holophytochrome-interacting proteins in Physcomitrella: putative actors in phytochrome cytoplasmic signaling

Anna Lena Ermert, Katharina Mailliet, and Jon Hughes*

*** Correspondence:** jon.hughes@uni-giessen.de

HIP9 (Pp1s254_10V6.2)

```
ATGGCCCTGGTGCATGGCATGCAGGCTGTGCTCTTTAGTCACATTCGACAGCTTCAACCTCCACATCAGTTTATTGATTCT
AATAGCAATTTGAAGGACTTTTCCTGTAGAGTCTTTAGGGATGATCACAGCTTGGGCTCTCAGACTGAGCAAATTATTAGT
TTTGAAGTGAGAAAACGAAGGGTTACTCGTGGGGCACATCGTCTGAGGTTCCCCCAGCCGGACCCGGAAGGTCTGACGTAT
CGCAGTGCATACAGCAGGAGGAGGCAAATACTACCTGCAGGTCCTGTGAATTGTCTGCAAACAAGTCGAACGGAACAGTTG
GATGACAGGTCGGTTGAGGAAGAAACTGTAAATTCTACAATCGAAGTCAAAGAGGATGTTGCAGAGACAGATGAATGGGCT
GTTAAGAAGAGTTTGAGTGCACTGGATGCCTACTTTGACAAACTCCATGCGAGTAGAGGAGAAGAACCCTCAAGCTCTACA
CCATATTCGCAAGGAACAGTAAATGGGGCTTCCGCAGCTGTGTCGCAATCCAAAACCGGCAGTAGCGGGCCAGTTATGGGC
AGTCAGACTTCTCAAGATTTCGGTGCAAGAAACGTGGTGAAGGATCGTGTAGAAAGTGATGATAAAAATAGTGCAGGTGGA
CTCAATGCACTGGATGCATACTTCGACAAGCTACGGCCCCGTGAATCTGAAAAAACTTCAACCTCCTACGAGAATACAAAG
AATGTAGTGGAAGAGGACAAACCGACCATAGGCAAAGACGAGAAAGAAGTAAGTATCAAGGTTGTTAGTGAGTTGGACATC
ACTGTAGGGGAGGAGGAGGAAGCAGAATATAGAGAATTTATGGAAGAGTTGGAGAAGGCCCTTCAGCAACAAGTGGAGTCG
GGAAAACTTTTGTCCGCAGACGATGACAACTTCATGCAGGAATCCTCTTGGGGCTTGCAATCTGCCAATTCGAATTCTTAT
GTTGTGAATGGCTTGGTAGCTTTAAATGTTGCAGTATACTTATTTGGTTTGGCTAGTCCACAAGAGGTGCCCGGTATGGTA
GATGCCTCTCTTCCTTACCTGTATGGAGCGAAAGTTAACGAGTTGATTGTAAATGGAGAGTGGTGGCGTCTAATTACCCCA
ACGTTTCTGCATTCAGGCTTTTTGCACTTAGGTTTGAGCACGTGGGCGTTGCTAGAATTTGGACCAGCAGTTGGAAGTGCT
TTTGGCACTTTAGGATTTTCCGCGATATATCTACTAGGCGGTTTGTATGGTAATCTTTTGAGCTTCTTCCACACGCCTCAA
GGCACCGTTGGCGGCTCTGGTCCTATTTTTGCCTTAATGGCTGCTTGGGTGGTCTATATACTACGCAACCGAGATATTATT
GGTCTGGATGTAGCTGGTGAAATAATTCGGAAAGTGGTCATCTTTACTGCTATCACTTATGCATTATGTAACTCTTTCCCT
GTTGACGATTGGACCCATCTGGGAGCAGCAATAAGTGGATTGATCTTTGGCCTACTCACTTGTCCTCTGGTGCGAGTTAAT
GTTCGTGTTGAAGACGTGAGTGACGAAAATGACGATGACGTAATGGAGTCATTTCTTCTTTTGAACGAGGGGATCGATCCA
TATCGGCTTCTTCTTGTGTTTGCCCTTGCTGTGGCGGTCTTTTCCGCTCTCTTCAGTGTGGGGGTGCCATATGCAACAGAG
TATCACTTTCTCCATGGCGGCCTTGGTGATGAGTATTGG
```

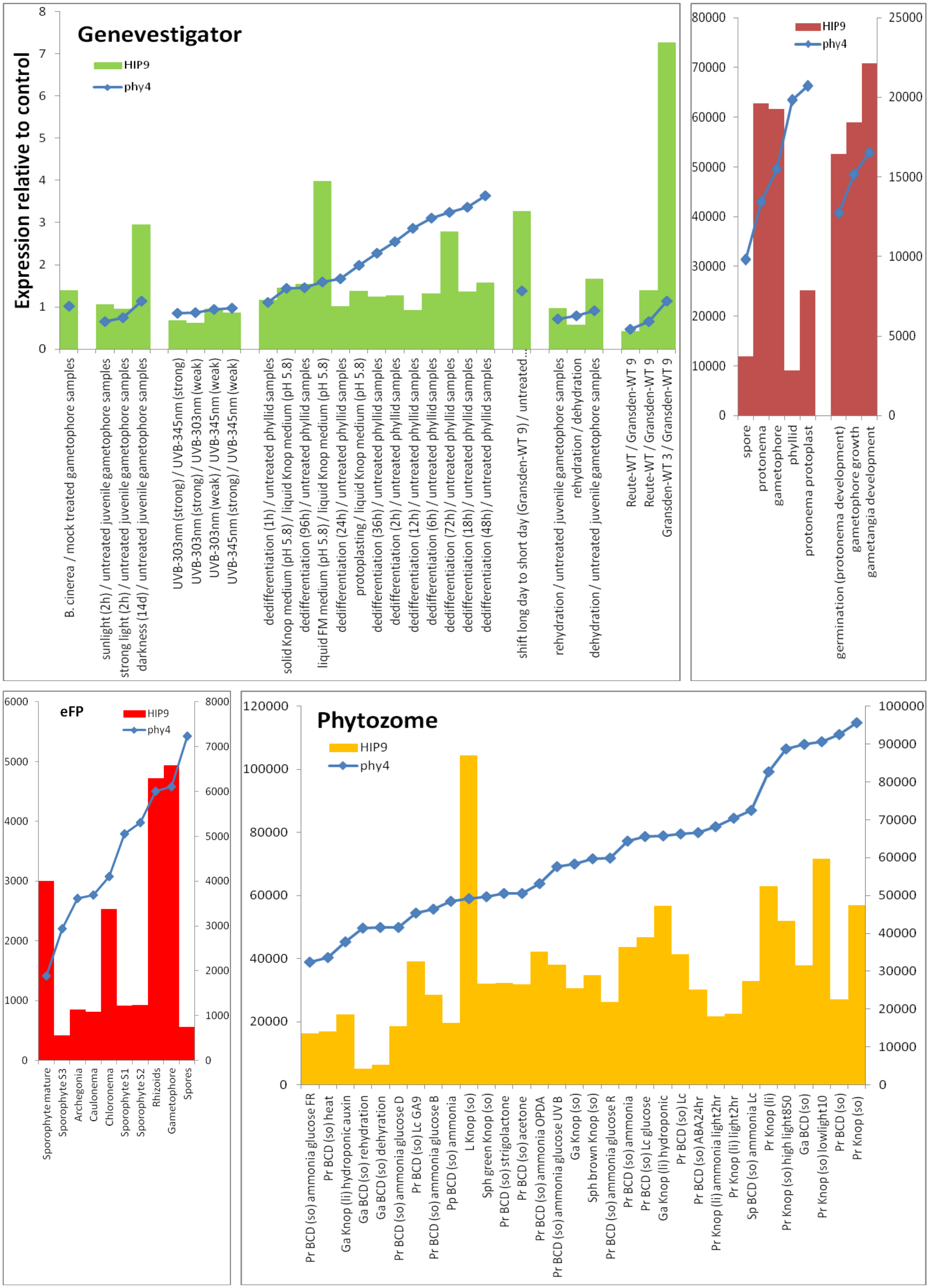

Genevestigator
Expression relative to control
HIP9
phy4
B. cinerea / mock treated gametophore samples
sunlight (2h) / untreated juvenile gametophore samples
strong light (2h) / untreated juvenile gametophore samples
darkness (14d) / untreated juvenile gametophore samples
UVB-303nm (strong) / UVB-345nm (strong)
UVB-303nm (strong) / UVB-303nm (weak)
UVB-303nm (weak) / UVB-345nm (weak)
UVB-345nm (strong) / UVB-345nm (weak)
dedifferentiation (1h) / untreated phyllid samples
solid Knop medium (pH 5.8) / liquid Knop medium (pH 5.8)
dedifferentiation (96h) / untreated phyllid samples
liquid FM medium (pH 5.8) / liquid Knop medium (pH 5.8)
dedifferentiation (24h) / untreated phyllid samples
protoplasting / liquid Knop medium (pH 5.8)
dedifferentiation (36h) / untreated phyllid samples
dedifferentiation (2h) / untreated phyllid samples
dedifferentiation (12h) / untreated phyllid samples
dedifferentiation (6h) / untreated phyllid samples
dedifferentiation (72h) / untreated phyllid samples
dedifferentiation (18h) / untreated phyllid samples
dedifferentiation (48h) / untreated phyllid samples
shift long day to short day (Gransden-WT 9) / untreated...
rehydration / untreated juvenile gametophore samples
rehydration / dehydration
dehydration / untreated juvenile gametophore samples
Reute-WT / Gransden-WT 9
Reute-WT / Gransden-WT 9
Gransden-WT 3 / Gransden-WT 9
HIP9
phy4
spore
protonema
gametophore
phyllid
protonema protoplast
germination (protonema development)
gametophore growth
gametangia development
eFP
HIP9
phy4
Sporophyte mature
Sporophyte S3
Archegonia
Caulonema
Chloronema
Sporophyte S1
Sporophyte S2
Rhizoids
Gametophore
Spores
Phytozome
HIP9
phy4
Pr BCD (so) ammonia glucose FR
Pr BCD (so) heat
Ga Knop (li) hydroponic auxin
Ga BCD (so) rehydration
Ga BCD (so) dehyration
Pr BCD (so) ammonia glucose D
Pr BCD (so) Lc GA9
Pr BCD (so) ammonia glucose B
Pp BCD (so) ammonia
L Knop (so)
Sph green Knop (so)
Pr BCD (so) strigolactone
Pr BCD (so) acetone
Pr BCD (so) ammonia OPDA
Pr BCD (so) ammonia glucose UV B
Ga Knop (so)
Sph brown Knop (so)
Pr BCD (so) ammonia glucose R
Pr BCD (so) ammonia
Pr BCD (so) Lc glucose
Ga Knop (li) hydroponic
Pr BCD (so) Lc
Pr BCD (so) ABA24hr
Pr Knop (li) ammonia light2hr
Pr Knop (li) light2hr
Sp BCD (so) ammonia Lc
Pr Knop (li)
Pr Knop (so) high light850
Ga BCD (so)
Pr Knop (so) lowlight10
Pr BCD (so)
Pr Knop (so)

HIP9 alignment tree

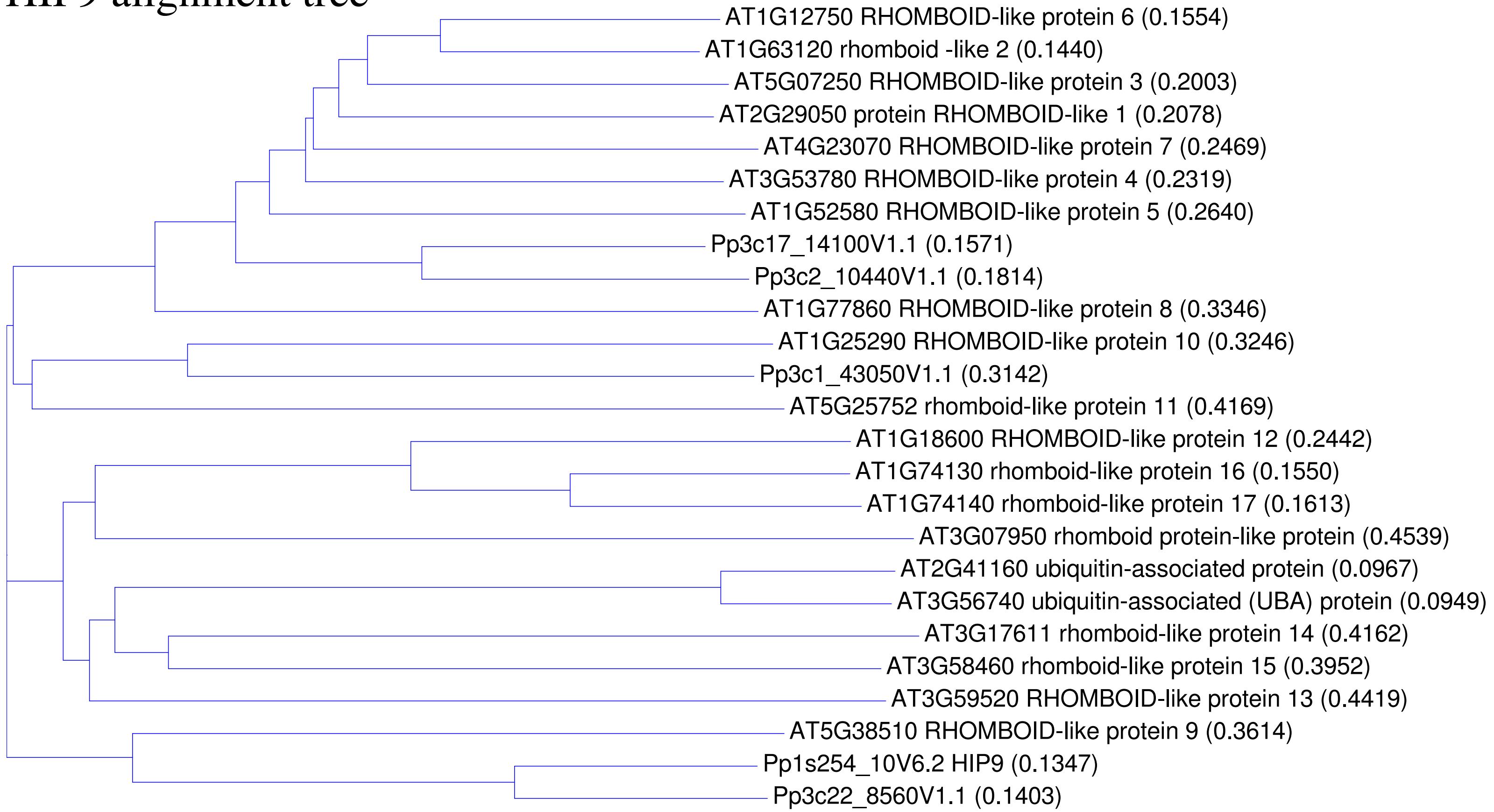

HIP9 alignment

```
                                               (1) 1       10        20        30        40        50                68
            AT1G12750 RHOMBOID-like protein 6  (1) --------------------------------------------------------------------
                AT1G63120 rhomboid -like 2     (1) --------------------------------------------------------------------
            AT5G07250 RHOMBOID-like protein 3  (1) --------------------------------------------------------------------
            AT2G29050 protein RHOMBOID-like 1  (1) --------------------------------------------------------------------
            AT4G23070 RHOMBOID-like protein 7  (1) --------------------------------------------------------------------
            AT3G53780 RHOMBOID-like protein 4  (1) --------------------------------------------------------------------
            AT1G52580 RHOMBOID-like protein 5  (1) --------------------------------------------------------------------
                           Pp3c17_14100V1.1    (1) --------------------------------------------------------------------
                            Pp3c2_10440V1.1    (1) --------------------------------------------------------------------
            AT1G77860 RHOMBOID-like protein 8  (1) --------------------------------------------------------------------
           AT1G25290 RHOMBOID-like protein 10  (1) --------------------------------------------------------------------
                            Pp3c1_43050V1.1    (1) --------------------------------------------------------------------
           AT5G25752 rhomboid-like protein 11  (1) --------------------------------------------------------------------
           AT1G18600 RHOMBOID-like protein 12  (1) --------------------------------------------------------------------
           AT1G74130 rhomboid-like protein 16  (1) --------------------------------------------------------------------
           AT1G74140 rhomboid-like protein 17  (1) --------------------------------------------------------------------
          AT3G07950 rhomboid protein-like protein (1) --------------------------------------------------------------------
          AT2G41160 ubiquitin-associated protein (1) --------------------------------------------------------------------
 AT3G56740 ubiquitin-associated (UBA) protein (1) --------------------------------------------------------------------
           AT3G17611 rhomboid-like protein 14  (1) --------------------------------------------------------------------
           AT3G58460 rhomboid-like protein 15  (1) --------------------------------------------------------------------
           AT3G59520 RHOMBOID-like protein 13  (1) --------------------------------------------------------------------
            AT5G38510 RHOMBOID-like protein 9  (1) --------------------------------------------------------------------
                        Pp1s254_10V6.2 HIP9    (1) MALVHGMQAVLFSHIRQLQPPHQFIDSNSNLKDFSCRVFRDDHSLGSQTEQIISFEVRKRRVTRGAHR
                           Pp3c22_8560V1.1     (1) MALVQGIQTVLYSHMRHLQPPHQPLECSSNLKDFSCKDFRGNRGLGSQTKRIISAEARKQRFAR----
                                 Consensus     (1)
```

Peptidase S54, rhomboid superfamily

	(69)	69 80 90 100 110 120 136
AT1G12750 RHOMBOID-like protein 6	(1)	--
AT1G63120 rhomboid -like 2	(1)	--
AT5G07250 RHOMBOID-like protein 3	(1)	--
AT2G29050 protein RHOMBOID-like 1	(1)	--
AT4G23070 RHOMBOID-like protein 7	(1)	--
AT3G53780 RHOMBOID-like protein 4	(1)	--
AT1G52580 RHOMBOID-like protein 5	(1)	--
Pp3c17_14100V1.1	(1)	--
Pp3c2_10440V1.1	(1)	--
AT1G77860 RHOMBOID-like protein 8	(1)	--
AT1G25290 RHOMBOID-like protein 10	(1)	--
Pp3c1_43050V1.1	(1)	--
AT5G25752 rhomboid-like protein 11	(1)	--
AT1G18600 RHOMBOID-like protein 12	(1)	--
AT1G74130 rhomboid-like protein 16	(1)	--
AT1G74140 rhomboid-like protein 17	(1)	--
AT3G07950 rhomboid protein-like protein	(1)	--
AT2G41160 ubiquitin-associated protein	(1)	--
AT3G56740 ubiquitin-associated (UBA) protein	(1)	--
AT3G17611 rhomboid-like protein 14	(1)	--
AT3G58460 rhomboid-like protein 15	(1)	--
AT3G59520 RHOMBOID-like protein 13	(1)	--
AT5G38510 RHOMBOID-like protein 9	(1)	--
Pp1s254_10V6.2 HIP9	(69)	LRFPQPDPEGLTYRSAYSRRRQILPAGPVNCLQTSRTEQLDDRSVEEETVNSTIEVKE-DVAETDEWA
Pp3c22_8560V1.1	(65)	LRFFHQEPVWLTYRSKLRGEKFVLGSGPLNCLQTRQVEHAEDTLPKEKTLDSCVEIKKGDVTETDEWA
Consensus	(69)	

Peptidase S54, rhomboid superfamily

```
                                             (137) 137          150       160       170       180       190          204
AT1G12750 RHOMBOID-like protein 6              (1) --------------------------------------------------------------------
AT1G63120 rhomboid -like 2                     (1) --------------------------------------------------------------------
AT5G07250 RHOMBOID-like protein 3              (1) --------------------------------------------------------------------
AT2G29050 protein RHOMBOID-like 1              (1) --------------------------------------------------------------------
AT4G23070 RHOMBOID-like protein 7              (1) --------------------------------------------------------------------
AT3G53780 RHOMBOID-like protein 4              (1) --------------------------------------------------------------------
AT1G52580 RHOMBOID-like protein 5              (1) --------------------------------------------------------------------
Pp3c17_14100V1.1                               (1) --------------------------------------------------------------------
Pp3c2_10440V1.1                                (1) --------------------------------------------------------------------
AT1G77860 RHOMBOID-like protein 8              (1) --------------------------------------------------------------------
AT1G25290 RHOMBOID-like protein 10             (1) --------------------------------------------------------------------
Pp3c1_43050V1.1                                (1) ----------------------------------MEMFGGAIRMTGCHKNPAWMAMLAGMMPTQGDLR
AT5G25752 rhomboid-like protein 11             (1) --------------------------------------------------------------------
AT1G18600 RHOMBOID-like protein 12             (1) -------------------------------------------------------------------M
AT1G74130 rhomboid-like protein 16             (1) -------------------------------------------------------------------M
AT1G74140 rhomboid-like protein 17             (1) -------------------------------------------------------------------M
AT3G07950 rhomboid protein-like protein        (1) --------------------------------------------------------------------
AT2G41160 ubiquitin-associated protein        (1) --------------------------------------------------------------------
AT3G56740 ubiquitin-associated (UBA) protein  (1) --------------------------------------------------------------------
AT3G17611 rhomboid-like protein 14             (1) --------------------------------------------------------------------
AT3G58460 rhomboid-like protein 15             (1) -----------------------------------------------------------------MTY
AT3G59520 RHOMBOID-like protein 13             (1) --------------------------------------------------------------------
AT5G38510 RHOMBOID-like protein 9              (1) --MALFPLHHEVPCKGEVLFDSNGLRRFSSGLKHRTMAEATTLGRDCRMKSYMKSIPYCRSPRRRLCL
Pp1s254_10V6.2 HIP9                          (136) VKKSLSALDAYFDKLHASRGEEPSSSTPYSQGTVNGASAAVSQSKTGSSGPVMGSQTSQDFGARNVVK
Pp3c22_8560V1.1                              (133) VKKGLNALDAYFDKLHASRAEEKSISTLRSQGTDTGVPTTALPPKTDSSG---LVVTSEVADAGNVVK
Consensus                                    (137)
```

Peptidase S54, rhomboid superfamily

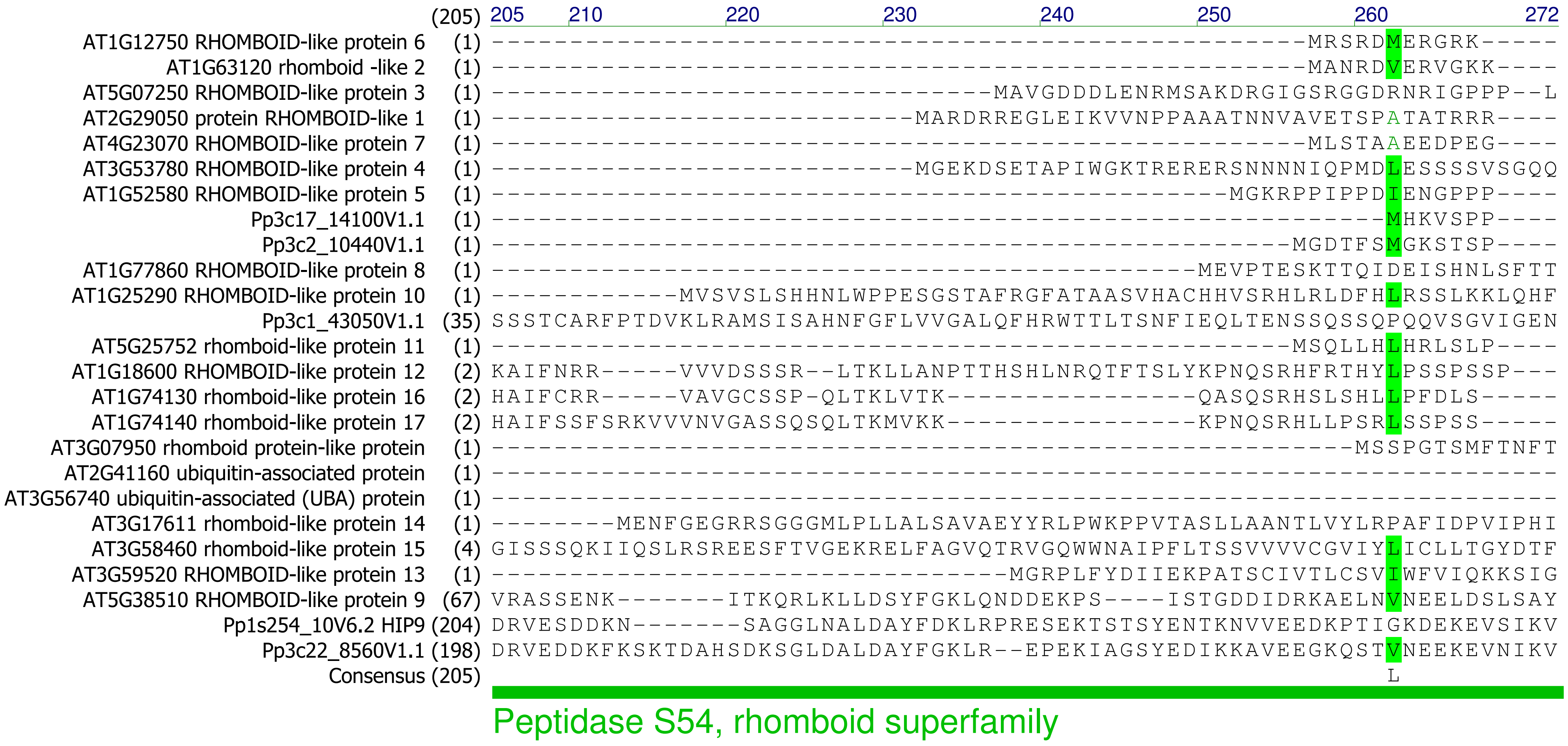
(205) 205 210 220 230 240 250 260 272
AT1G12750 RHOMBOID-like protein 6 (1) ---MRSRDMERGRK-----
AT1G63120 rhomboid -like 2 (1) ---MANRDVERVGKK----
AT5G07250 RHOMBOID-like protein 3 (1) --------------------------------MAVGDDDLENRMSAKDRGIGSRGGDRNRIGPPP--L
AT2G29050 protein RHOMBOID-like 1 (1) ---------------------------MARDRREGLEIKVVNPPAAATNNVAVETSPATATRRR----
AT4G23070 RHOMBOID-like protein 7 (1) ---MLSTAAEEDPEG----
AT3G53780 RHOMBOID-like protein 4 (1) ---------------------------MGEKDSETAPIWGKTRERERSNNNNIQPMDLESSSSVSGQQ
AT1G52580 RHOMBOID-like protein 5 (1) --MGKRPPIPPDIENGPPP----
Pp3c17_14100V1.1 (1) --MHKVSPP----
Pp3c2_10440V1.1 (1) ---MGDTFSMGKSTSP----
AT1G77860 RHOMBOID-like protein 8 (1) ---MEVPTESKTTQIDEISHNLSFTT
AT1G25290 RHOMBOID-like protein 10 (1) ------------MVSVSLSHHNLWPPESGSTAFRGFATAASVHACHHVSRHLRLDFHLRSSLKKLQHF
Pp3c1_43050V1.1 (35) SSSTCARFPTDVKLRAMSISAHNFGFLVVGALQFHRWTTLTSNFIEQLTENSSQSSQPQQVSGVIGEN
AT5G25752 rhomboid-like protein 11 (1) ---MSQLLHLHRLSLP----
AT1G18600 RHOMBOID-like protein 12 (2) KAIFNRR-----VVVDSSSR--LTKLLANPTTHSHLNRQTFTSLYKPNQSRHFRTHYLPSSPSSP---
AT1G74130 rhomboid-like protein 16 (2) HAIFCRR-----VAVGCSSP-QLTKLVTK----------------QASQSRHSLSHLLPFDLS-----
AT1G74140 rhomboid-like protein 17 (2) HAIFSSFSRKVVVNVGASSQSQLTKMVKK----------------KPNQSRHLLPSRLSSPSS-----
AT3G07950 rhomboid protein-like protein (1) ---MSSPGTSMFTNFT
AT2G41160 ubiquitin-associated protein (1) --
AT3G56740 ubiquitin-associated (UBA) protein (1) --
AT3G17611 rhomboid-like protein 14 (1) --------MENFGEGRRSGGGMLPLLALSAVAEYYRLPWKPPVTASLLAANTLVYLRPAFIDPVIPHI
AT3G58460 rhomboid-like protein 15 (4) GISSSQKIIQSLRSREESFTVGEKRELFAGVQTRVGQWWNAIPFLTSSVVVVCGVIYLICLLTGYDTF
AT3G59520 RHOMBOID-like protein 13 (1) ---------------------------------MGRPLFYDIIEKPATSCIVTLCSVIWFVIQKKSIG
AT5G38510 RHOMBOID-like protein 9 (67) VRASSENK-------ITKQRLKLLDSYFGKLQNDDEKPS----ISTGDDIDRKAELNVNEELDSLSAY
Pp1s254_10V6.2 HIP9 (204) DRVESDDKN-------SAGGLNALDAYFDKLRPRESEKTSTSYENTKNVVEEDKPTIGKDEKEVSIKV
Pp3c22_8560V1.1 (198) DRVEDDKFKSKTDAHSDKSGLDALDAYFGKLR--EPEKIAGSYEDIKKAVEEGKQSTVNEEKEVNIKV
Consensus (205) L
Peptidase S54, rhomboid superfamily

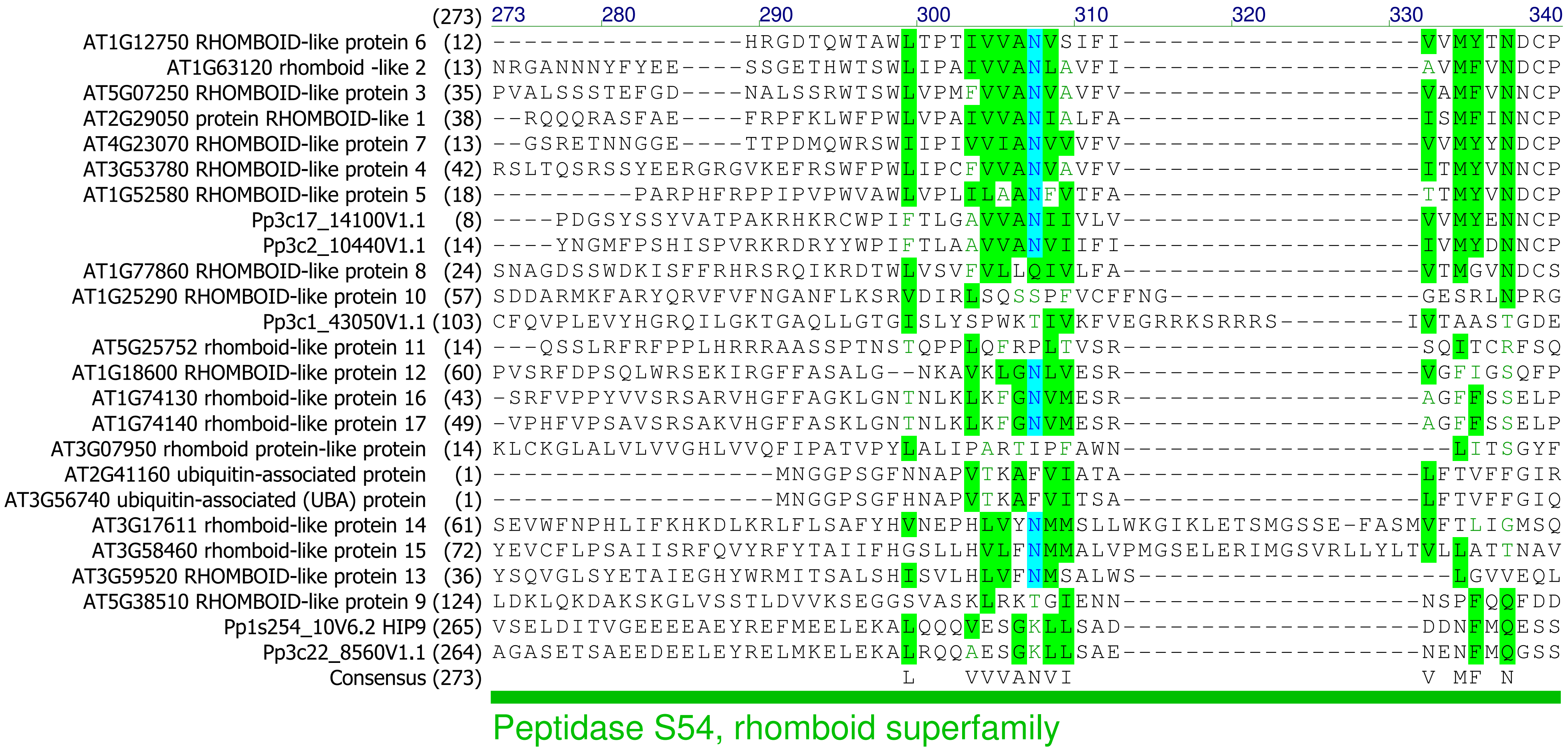
(273) 273 280 290 300 310 320 330 340
AT1G12750 RHOMBOID-like protein 6 (12) ----------------HRGDTQWTAWLTPTIVVANVSIFI-------------------VVMYTNDCP
AT1G63120 rhomboid -like 2 (13) NRGANNNYFYEE----SSGETHWTSWLIPAIVVANLAVFI-------------------AVMFVNDCP
AT5G07250 RHOMBOID-like protein 3 (35) PVALSSSTEFGD----NALSSRWTSWLVPMFVVANVAVFV-------------------VAMFVNNCP
AT2G29050 protein RHOMBOID-like 1 (38) --RQQQRASFAE----FRPFKLWFPWLVPAIVVANIALFA-------------------ISMFINNCP
AT4G23070 RHOMBOID-like protein 7 (13) --GSRETNNGGE----TTPDMQWRSWIIPIVVIANVVVFV-------------------VVMYYNDCP
AT3G53780 RHOMBOID-like protein 4 (42) RSLTQSRSSYEERGRGVKEFRSWFPWLIPCFVVANVAVFV-------------------ITMYVNNCP
AT1G52580 RHOMBOID-like protein 5 (18) ---------PARPHFRPPIPVPWVAWLVPLILAANFVTFA-------------------TTMYVNDCP
Pp3c17_14100V1.1 (8) ----PDGSYSSYVATPAKRHKRCWPIFTLGAVVANIIVLV-------------------VVMYENNCP
Pp3c2_10440V1.1 (14) ----YNGMFPSHISPVRKRDRYYWPIFTLAAVVANVIIFI-------------------IVMYDNNCP
AT1G77860 RHOMBOID-like protein 8 (24) SNAGDSSWDKISFFRHRSRQIKRDTWLVSVFVLLQIVLFA-------------------VTMGVNDCS
AT1G25290 RHOMBOID-like protein 10 (57) SDDARMKFARYQRVFVFNGANFLKSRVDIRLSQSSPFVCFFNG----------------GESRLNPRG
Pp3c1_43050V1.1 (103) CFQVPLEVYHGRQILGKTGAQLLGTGISLYSPWKTIVKFVEGRRKSRRRS---------IVTAASTGDE
AT5G25752 rhomboid-like protein 11 (14) ---QSSLRFRFPPLHRRRAASSPTNSTQPPLQFRPLTVSR-------------------SQITCRFSQ
AT1G18600 RHOMBOID-like protein 12 (60) PVSRFDPSQLWRSEKIRGFFASALG--NKAVKLGNLVESR-------------------VGFIGSQFP
AT1G74130 rhomboid-like protein 16 (43) -SRFVPPYVVSRSARVHGFFAGKLGNTNLKLKFGNVMESR-------------------AGFFSSELP
AT1G74140 rhomboid-like protein 17 (49) -VPHFVPSAVSRSAKVHGFFASKLGNTNLKLKFGNVMESR-------------------AGFFSSELP
AT3G07950 rhomboid protein-like protein (14) KLCKGLALVLVVGHLVVQFIPATVPYLALIPARTIPFAWN--------------------LITSGYF
AT2G41160 ubiquitin-associated protein (1) ------------------MNGGPSGFNNAPVTKAFVIATA-------------------LFTVFFGIR
AT3G56740 ubiquitin-associated (UBA) protein (1) ------------------MNGGPSGFHNAPVTKAFVITSA-------------------LFTVFFGIQ
AT3G17611 rhomboid-like protein 14 (61) SEVWFNPHLIFKHKDLKRLFLSAFYHVNEPHLVYNMMSLLWKGIKLETSMGSSE-FASMVFTLIGMSQ
AT3G58460 rhomboid-like protein 15 (72) YEVCFLPSAIISRFQVYRFYTAIIFHGSLLHVLFNMMALVPMGSELERIMGSVRLLYLTVLLATTNAV
AT3G59520 RHOMBOID-like protein 13 (36) YSQVGLSYETAIEGHYWRMITSALSHISVLHLVFNMSALWS--------------------LGVVEQL
AT5G38510 RHOMBOID-like protein 9 (124) LDKLQKDAKSKGLVSSTLDVVKSEGGSVASKLRKTGIENN-------------------NSPFQQFDD
Pp1s254_10V6.2 HIP9 (265) VSELDITVGEEEEAEYREFMEELEKALQQQVESGKLLSAD-------------------DDNFMQESS
Pp3c22_8560V1.1 (264) AGASETSAEEDEELEYRELMKELEKALRQQAESGKLLSAE-------------------NENFMQGSS
Consensus (273) L VVVANVI V MF N
Peptidase S54, rhomboid superfamily

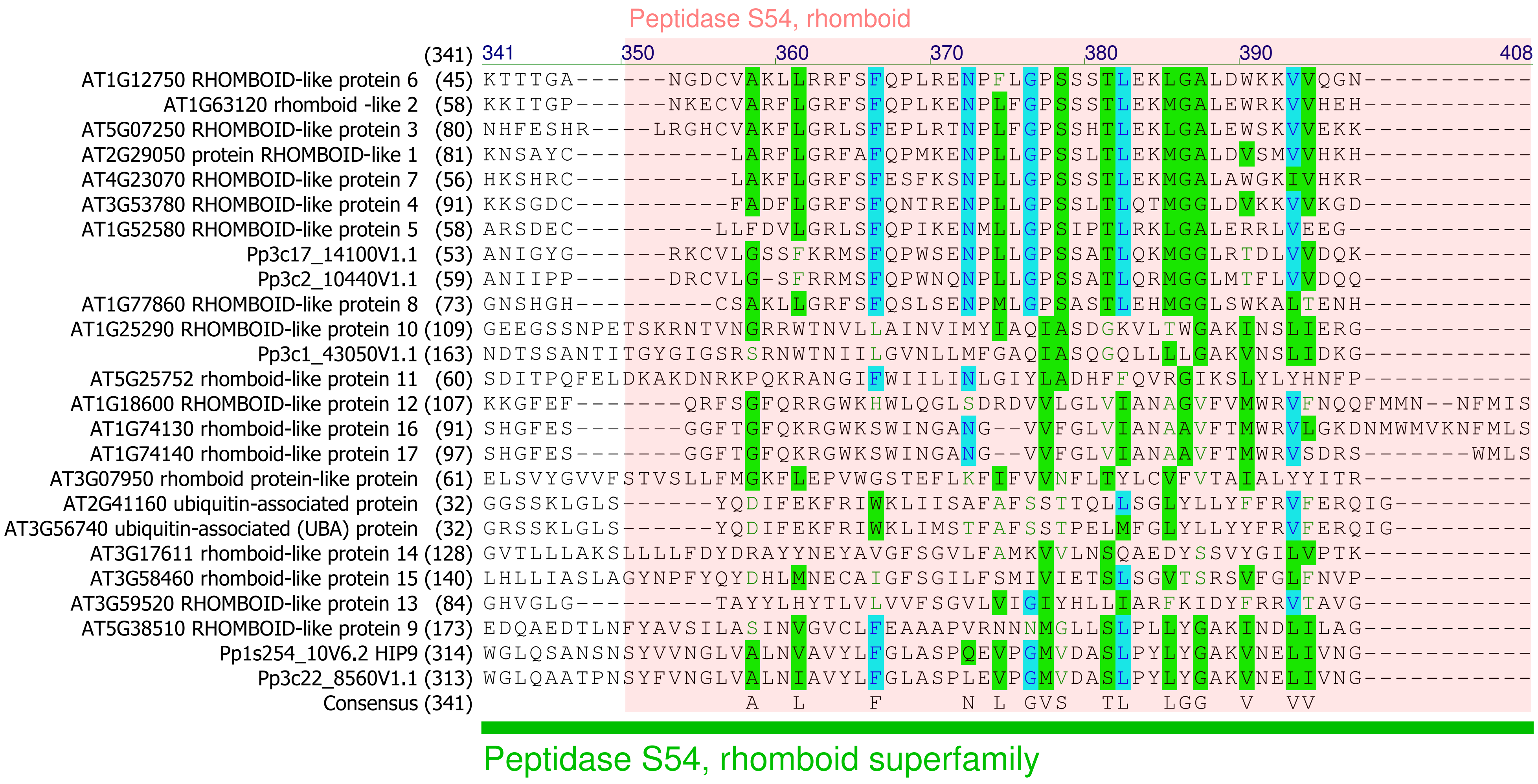

Peptidase S54, rhomboid
(341) 341 350 360 370 380 390 408
AT1G12750 RHOMBOID-like protein 6 (45) KTTTGA------NGDCVAKLLRRFSFQPLRENPFLGPSSSTLEKLGALDWKKVVQGN-----------
AT1G63120 rhomboid -like 2 (58) KKITGP------NKECVARFLGRFSFQPLKENPLFGPSSSTLEKMGALEWRKVVHEH-----------
AT5G07250 RHOMBOID-like protein 3 (80) NHFESHR---LRGHCVAKFLGRLSFEPLRTNPLFGPSSHTLEKLGALEWSKVVEKK-----------
AT2G29050 protein RHOMBOID-like 1 (81) KNSAYC----------LARFLGRFAFQPMKENPLLGPSSLTLEKMGALDVSMVVHKH-----------
AT4G23070 RHOMBOID-like protein 7 (56) HKSHRC----------LAKFLGRFSFESFKSNPLLGPSSSTLEKMGALAWGKIVHKR-----------
AT3G53780 RHOMBOID-like protein 4 (91) KKSGDC----------FADFLGRFSFQNTRENPLLGPSSLTLQTMGGLDVKKVVKGD-----------
AT1G52580 RHOMBOID-like protein 5 (58) ARSDEC---------LLFDVLGRLSFQPIKENMLLGPSIPTLRKLGALERRLVEEG-----------
Pp3c17_14100V1.1 (53) ANIGYG------RKCVLGSSFKRMSFQPWSENPLLGPSSATLQKMGGLRTDLVVDQK-----------
Pp3c2_10440V1.1 (59) ANIIPP------DRCVLG-SFRRMSFQPWNQNPLLGPSSATLQRMGGLMTFLVVDQQ-----------
AT1G77860 RHOMBOID-like protein 8 (73) GNSHGH---------CSAKLLGRFSFQSLSENPMLGPSASTLEHMGGLSWKALTENH-----------
AT1G25290 RHOMBOID-like protein 10 (109) GEEGSSNPETSKRNTVNGRRWTNVLLAINVIMYIAQIASDGKVLTWGAKINSLIERG-----------
Pp3c1_43050V1.1 (163) NDTSSANTITGYGIGSRSRNWTNIILGVNLLMFGAQIASQGQLLLLGAKVNSLIDKG-----------
AT5G25752 rhomboid-like protein 11 (60) SDITPQFELDKAKDNRKPQKRANGIFWIILINLGIYLADHFFQVRGIKSLYLYHNFP-----------
AT1G18600 RHOMBOID-like protein 12 (107) KKGFEF--------QRFSGFQRRGWKHWLQGLSDRDVVLGLVIANAGVFVMWRVFNQQFMMN--NFMIS
AT1G74130 rhomboid-like protein 16 (91) SHGFES--------GGFTGFQKRGWKSWINGANG--VVFGLVIANAAVFTMWRVLGKDNMWMVKNFMLS
AT1G74140 rhomboid-like protein 17 (97) SHGFES--------GGFTGFQKRGWKSWINGANG--VVFGLVIANAAVFTMWRVSDRS-------WMLS
AT3G07950 rhomboid protein-like protein (61) ELSVYGVVFSTVSLLFMGKFLEPVWGSTEFLKFIFVVNFLTYLCVFVTAIALYYITR-----------
AT2G41160 ubiquitin-associated protein (32) GGSSKLGLS------YQDIFEKFRIWKLIISAFAFSSTTQLLSGLYLLYFFRVFERQIG---------
AT3G56740 ubiquitin-associated (UBA) protein (32) GRSSKLGLS------YQDIFEKFRIWKLIMSTFAFSSTPELMFGLYLLYYFRVFERQIG---------
AT3G17611 rhomboid-like protein 14 (128) GVTLLLAKSLLLLFDYDRAYYNEYAVGFSGVLFAMKVVLNSQAEDYSSVYGILVPTK-----------
AT3G58460 rhomboid-like protein 15 (140) LHLLIASLAGYNPFYQYDHLMNECAIGFSGILFSMIVIETSLSGVTSRSVFGLFNVP-----------
AT3G59520 RHOMBOID-like protein 13 (84) GHVGLG---------TAYYLHYTLVLVVFSGVLVIGIYHLLIARFKIDYFRRVTAVG-----------
AT5G38510 RHOMBOID-like protein 9 (173) EDQAEDTLNFYAVSILASINVGVCLFEAAAPVRNNNMGLLSLPLLYGAKINDLILAG-----------
Pp1s254_10V6.2 HIP9 (314) WGLQSANSNSYVVNGLVALNVAVYLFGLASPQEVPGMVDASLPYLYGAKVNELIVNG-----------
Pp3c22_8560V1.1 (313) WGLQAATPNSYFVNGLVALNIAVYLFGLASPLEVPGMVDASLPYLYGAKVNELIVNG-----------
Consensus (341) A L F N L GVS TL LGG V VV
Peptidase S54, rhomboid superfamily

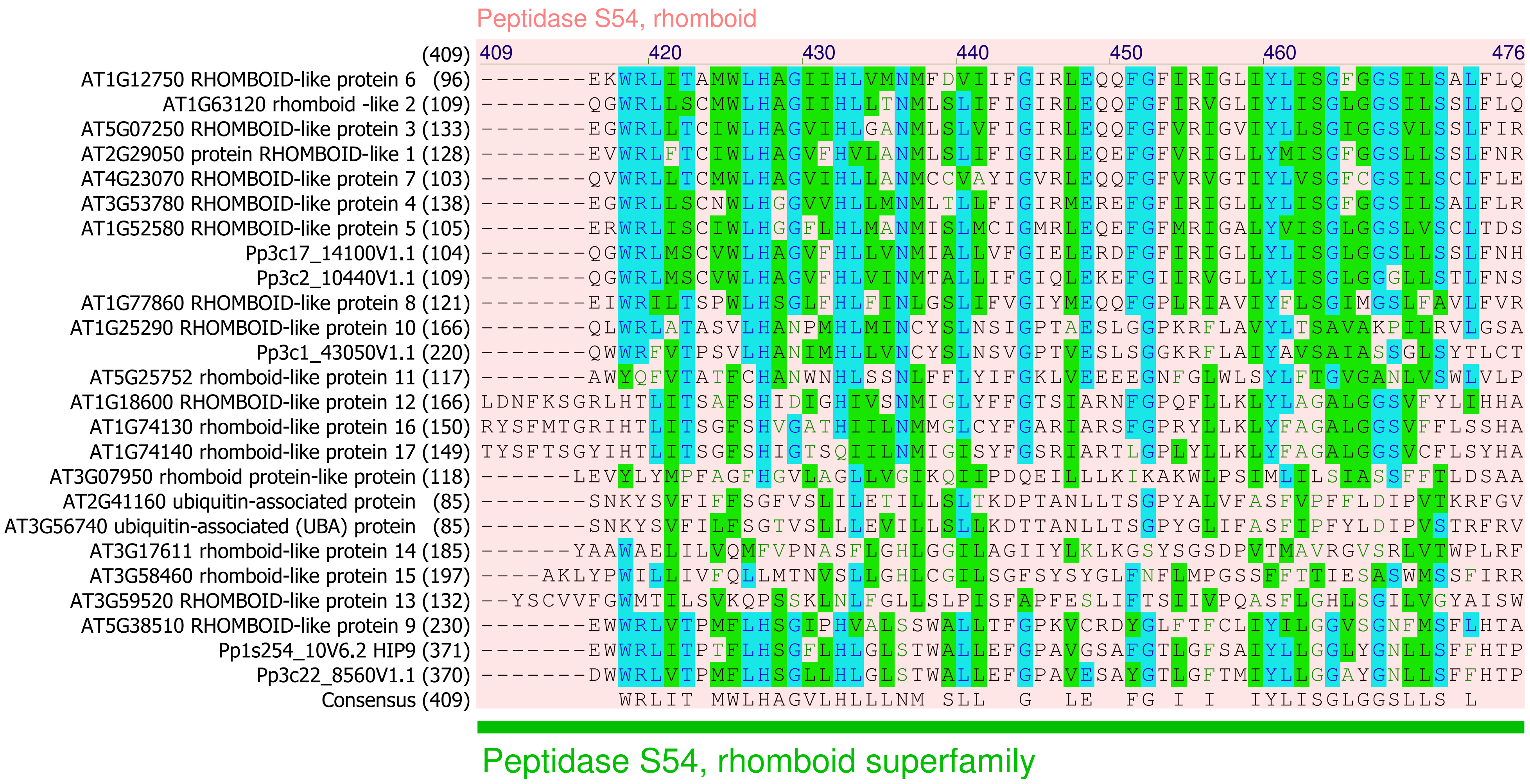

Peptidase S54, rhomboid
(409) 409 420 430 440 450 460 476
AT1G12750 RHOMBOID-like protein 6 (96)
AT1G63120 rhomboid -like 2 (109)
AT5G07250 RHOMBOID-like protein 3 (133)
AT2G29050 protein RHOMBOID-like 1 (128)
AT4G23070 RHOMBOID-like protein 7 (103)
AT3G53780 RHOMBOID-like protein 4 (138)
AT1G52580 RHOMBOID-like protein 5 (105)
Pp3c17_14100V1.1 (104)
Pp3c2_10440V1.1 (109)
AT1G77860 RHOMBOID-like protein 8 (121)
AT1G25290 RHOMBOID-like protein 10 (166)
Pp3c1_43050V1.1 (220)
AT5G25752 rhomboid-like protein 11 (117)
AT1G18600 RHOMBOID-like protein 12 (166)
AT1G74130 rhomboid-like protein 16 (150)
AT1G74140 rhomboid-like protein 17 (149)
AT3G07950 rhomboid protein-like protein (118)
AT2G41160 ubiquitin-associated protein (85)
AT3G56740 ubiquitin-associated (UBA) protein (85)
AT3G17611 rhomboid-like protein 14 (185)
AT3G58460 rhomboid-like protein 15 (197)
AT3G59520 RHOMBOID-like protein 13 (132)
AT5G38510 RHOMBOID-like protein 9 (230)
Pp1s254_10V6.2 HIP9 (371)
Pp3c22_8560V1.1 (370)
Consensus (409) WRLIT MWLHAGVLHLLLNM SLL G LE FG I I IYLISGLGGSLLS L
Peptidase S54, rhomboid superfamily

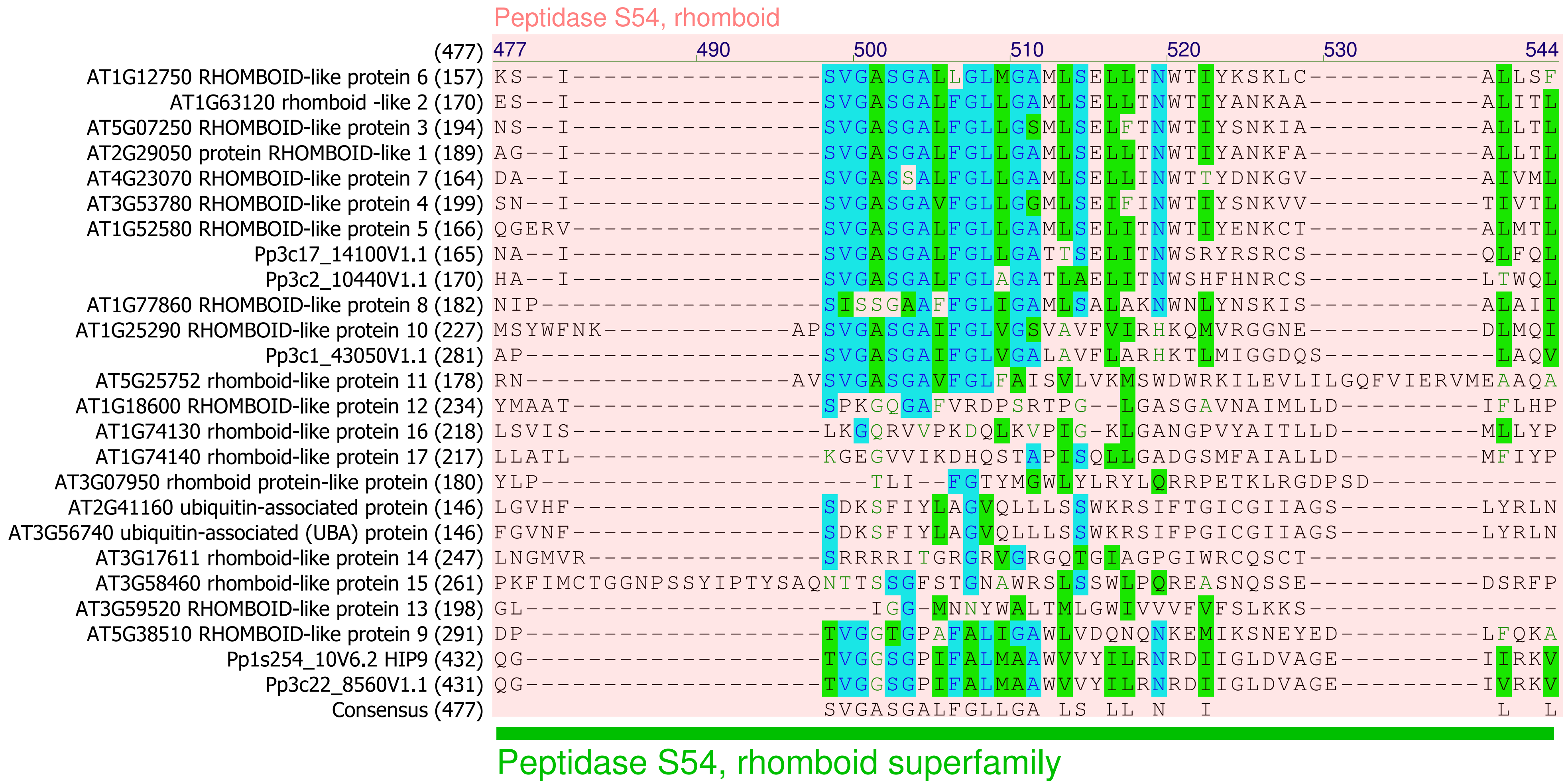
Peptidase S54, rhomboid
(477) 477 490 500 510 520 530 544
AT1G12750 RHOMBOID-like protein 6 (157) KS--I----------------SVGASGALLGLMGAMLSELLTNWTIYKSKLC-----------ALLSF
AT1G63120 rhomboid -like 2 (170) ES--I----------------SVGASGALFGLLGAMLSELLTNWTIYANKAA-----------ALITL
AT5G07250 RHOMBOID-like protein 3 (194) NS--I----------------SVGASGALFGLLGSMLSELFTNWTIYSNKIA-----------ALLTL
AT2G29050 protein RHOMBOID-like 1 (189) AG--I----------------SVGASGALFGLLGAMLSELLTNWTIYANKFA-----------ALLTL
AT4G23070 RHOMBOID-like protein 7 (164) DA--I----------------SVGASSALFGLLGAMLSELLINWTTYDNKGV-----------AIVML
AT3G53780 RHOMBOID-like protein 4 (199) SN--I----------------SVGASGAVFGLLGGMLSEIFINWTIYSNKVV-----------TIVTL
AT1G52580 RHOMBOID-like protein 5 (166) QGERV----------------SVGASGALFGLLGAMLSELITNWTIYENKCT-----------ALMTL
Pp3c17_14100V1.1 (165) NA--I----------------SVGASGALFGLLGATTSELITNWSRYRSRCS-----------QLFQL
Pp3c2_10440V1.1 (170) HA--I----------------SVGASGALFGLAGATLAELITNWSHFHNRCS-----------LTWQL
AT1G77860 RHOMBOID-like protein 8 (182) NIP------------------SISSGAAFFGLIGAMLSALAKNWNLYNSKIS-----------ALAII
AT1G25290 RHOMBOID-like protein 10 (227) MSYWFNK------------APSVGASGAIFGLVGSVAVFVIRHKQMVRGGNE-----------DLMQI
Pp3c1_43050V1.1 (281) AP-------------------SVGASGAIFGLVGALAVFLARHKTLMIGGDQS-----------LAQV
AT5G25752 rhomboid-like protein 11 (178) RN-----------------AVSVGASGAVFGLFAISVLVKMSWDWRKILEVLILGQFVIERVMEAAQA
AT1G18600 RHOMBOID-like protein 12 (234) YMAAT----------------SPKGQGAFVRDPSRTPG--LGASGAVNAIMLLD---------IFLHP
AT1G74130 rhomboid-like protein 16 (218) LSVIS----------------LKGQRVVPKDQLKVPIG-KLGANGPVYAITLLD---------MLLYP
AT1G74140 rhomboid-like protein 17 (217) LLATL----------------KGEGVVIKDHQSTAPISQLLGADGSMFAIALLD---------MFIYP
AT3G07950 rhomboid protein-like protein (180) YLP---------------------TLI--FGTYMGWLYLRYLQRRPETKLRGDPSD------------
AT2G41160 ubiquitin-associated protein (146) LGVHF----------------SDKSFIYLAGVQLLLSSWKRSIFTGICGIIAGS---------LYRLN
AT3G56740 ubiquitin-associated (UBA) protein (146) FGVNF----------------SDKSFIYLAGVQLLLSSWKRSIFPGICGIIAGS---------LYRLN
AT3G17611 rhomboid-like protein 14 (247) LNGMVR---------------SRRRRITGRGRVGRGQTGIAGPGIWRCQSCT----------------
AT3G58460 rhomboid-like protein 15 (261) PKFIMCTGGNPSSYIPTYSAQNTTSSGFSTGNAWRSLSSWLPQREASNQSSE-----------DSRFP
AT3G59520 RHOMBOID-like protein 13 (198) GL----------------------IGG-MNNYWALTMLGWIVVVFVFSLKKS----------------
AT5G38510 RHOMBOID-like protein 9 (291) DP-------------------TVGGTGPAFALIGAWLVDQNQNKEMIKSNEYED---------LFQKA
Pp1s254_10V6.2 HIP9 (432) QG-------------------TVGGSGPIFALMAAWVVYILRNRDIIGLDVAGE---------IIRKV
Pp3c22_8560V1.1 (431) QG-------------------TVGGSGPIFALMAAWVVYILRNRDIIGLDVAGE---------IVRKV
Consensus (477) SVGASGALFGLLGA LS LL N I L L
Peptidase S54, rhomboid superfamily

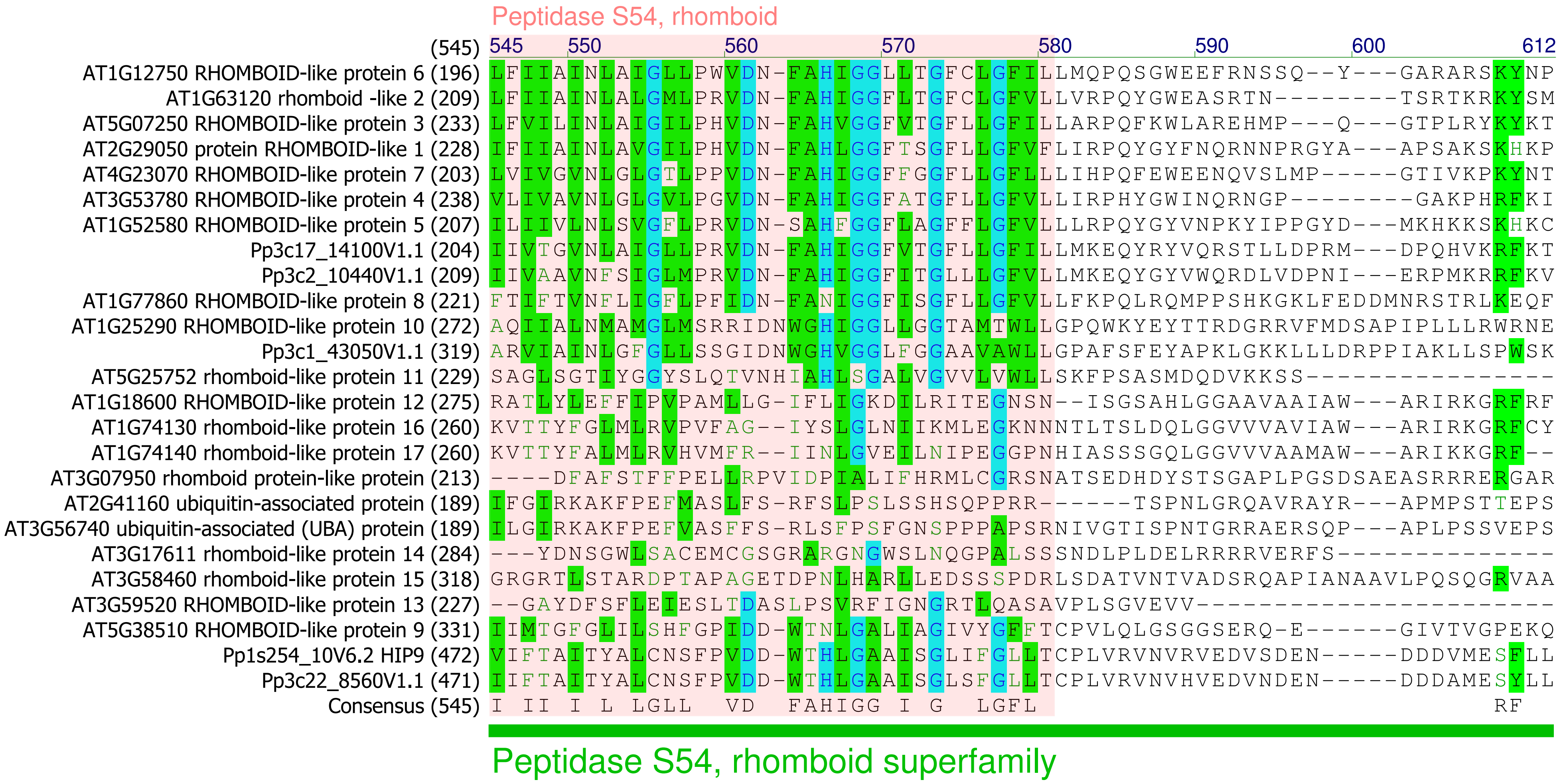
Peptidase S54, rhomboid
(545) 545 550 560 570 580 590 600 612
AT1G12750 RHOMBOID-like protein 6 (196) LFIIAINLAIGLLPWVDN-FAHIGGLLTGFCLGFILLMQPQSGWEEFRNSSQ--Y---GARARSKYNP
AT1G63120 rhomboid -like 2 (209) LFIIAINLALGMLPRVDN-FAHIGGFLTGFCLGFVLLVRPQYGWEASRTN--------TSRTKRKYSM
AT5G07250 RHOMBOID-like protein 3 (233) LFVILINLAIGILPHVDN-FAHVGGFVTGFLLGFILLARPQFKWLAREHMP---Q---GTPLRYKYKT
AT2G29050 protein RHOMBOID-like 1 (228) IFIIAINLAVGILPHVDN-FAHLGGFTSGFLLGFVFLIRPQYGYFNQRNNPRGYA---APSAKSKHKP
AT4G23070 RHOMBOID-like protein 7 (203) LVIVGVNLGLGTLPPVDN-FAHIGGFFGGFLLGFLLLIHPQFEWEENQVSLMP-----GTIVKPKYNT
AT3G53780 RHOMBOID-like protein 4 (238) VLIVAVNLGLGVLPGVDN-FAHIGGFATGFLLGFVLLIRPHYGWINQRNGP--------GAKPHRFKI
AT1G52580 RHOMBOID-like protein 5 (207) ILIIVLNLSVGFLPRVDN-SAHFGGFLAGFFLGFVLLLRPQYGYVNPKYIPPGYD---MKHKKSKHKC
Pp3c17_14100V1.1 (204) IIVTGVNLAIGLLPRVDN-FAHIGGFVTGFLLGFILLMKEQYRYVQRSTLLDPRM---DPQHVKRFKT
Pp3c2_10440V1.1 (209) IIVAAVNFSIGLMPRVDN-FAHIGGFITGLLLGFVLLMKEQYGYVWQRDLVDPNI---ERPMKRRFKV
AT1G77860 RHOMBOID-like protein 8 (221) FTIFTVNFLIGFLPFIDN-FANIGGFISGFLLGFVLLFKPQLRQMPPSHKGKLFEDDMNRSTRLKEQF
AT1G25290 RHOMBOID-like protein 10 (272) AQIIALNMAMGLMSRRIDNWGHIGGLLGGTAMTWLLGPQWKYEYTTRDGRRVFMDSAPIPLLLRWRNE
Pp3c1_43050V1.1 (319) ARVIAINLGFGLLSSGIDNWGHVGGLFGGAAVAWLLGPAFSFEYAPKLGKKLLLDRPPIAKLLSPWSK
AT5G25752 rhomboid-like protein 11 (229) SAGLSGTIYGGYSLQTVNHIAHLSGALVGVVLVWLLSKFPSASMDQDVKKSS----------------
AT1G18600 RHOMBOID-like protein 12 (275) RATLYLEFFIPVPAMLLG-IFLIGKDILRITEGNSN--ISGSAHLGGAAVAAIAW---ARIRKGRFRF
AT1G74130 rhomboid-like protein 16 (260) KVTTYFGLMLRVPVFAG--IYSLGLNIIKMLEGKNNNTLTSLDQLGGVVVAVIAW---ARIRKGRFCY
AT1G74140 rhomboid-like protein 17 (260) KVTTYFALMLRVHVMFR--IINLGVEILNIPEGGPNHIASSSGQLGGVVVAAMAW---ARIKKGRF--
AT3G07950 rhomboid protein-like protein (213) ----DFAFSTFFPELLRPVIDPIALIFHRMLCGRSNATSEDHDYSTSGAPLPGSDSAEASRRRERGAR
AT2G41160 ubiquitin-associated protein (189) IFGIRKAKFPEFMASLFS-RFSLPSLSSHSQPPRR------TSPNLGRQAVRAYR---APMPSTTEPS
AT3G56740 ubiquitin-associated (UBA) protein (189) ILGIRKAKFPEFVASFFS-RLSFPSFGNSPPPAPSRNIVGTISPNTGRRAERSQP---APLPSSVEPS
AT3G17611 rhomboid-like protein 14 (284) ---YDNSGWLSACEMCGSGRARGNGWSLNQGPALSSSNDLPLDELRRRRVERFS--------------
AT3G58460 rhomboid-like protein 15 (318) GRGRTLSTARDPTAPAGETDPNLHARLLEDSSSPDRLSDATVNTVADSRQAPIANAAVLPQSQGRVAA
AT3G59520 RHOMBOID-like protein 13 (227) --GAYDFSFLEIESLTDASLPSVRFIGNGRTLQASAVPLSGVEVV-----------------------
AT5G38510 RHOMBOID-like protein 9 (331) IIMTGFGLILSHFGPIDD-WTNLGALIAGIVYGFFTCPVLQLGSGGSERQ-E------GIVTVGPEKQ
Pp1s254_10V6.2 HIP9 (472) VIFTAITYALCNSFPVDD-WTHLGAAISGLIFGLLTCPLVRVNVRVEDVSDEN-----DDDVMESFLL
Pp3c22_8560V1.1 (471) IIFTAITYALCNSFPVDD-WTHLGAAISGLSFGLLTCPLVRVNVHVEDVNDEN-----DDDAMESYLL
Consensus (545) I II I L LGLL VD FAHIGG I G LGFL RF
Peptidase S54, rhomboid superfamily

```
                                              (613) 613    620       630       640       650       660       670      680
     AT1G12750 RHOMBOID-like protein 6 (258) -CQYVLFFVAAVLVVAGLTVGLVMLFDGENGNKHCKWCHRLDCYPTSKWSC-----------------
              AT1G63120 rhomboid -like 2 (268) -YQYVLFVVSVVLLVVGLTVALVMLFKGENGNKHCKWCHYLSCFPTSKWTC-----------------
     AT5G07250 RHOMBOID-like protein 3 (294) -YQYLLWLLSLVLLIAGFVVALLMLFRGENGNDHCRWCHYLRCVPTSSWRCDDV--------------
     AT2G29050 protein RHOMBOID-like 1 (292) -YQYVLWITSLVLLIAGYTAGLVVLLRGTDLNKHCSWCHYLSCIPTSLWSCKSQNVYCESSQIGQQMN
     AT4G23070 RHOMBOID-like protein 7 (265) -CQLVLCIVASIVFVAGFTSGLVILFRGDSLNRYCKWCHKLSYSSKSQWT------------------
     AT3G53780 RHOMBOID-like protein 4 (297) -YQGILWTISLLILVAGFIVGLISLFNNVDGNEHCSWCHYLSCVPTSKWSCNREPASCTTTQLGNQLS
     AT1G52580 RHOMBOID-like protein 5 (271) -YQHIFRFTSLAILLAGFIAGYTKLLR----------EHTIQSMPFRDFN------------------
                      Pp3c17_14100V1.1 (268) -YQFILLLVSLLLLIAGFAGGFVALYSGVDAYNRCSWCHYLNCVPSSHWTCDSQSG------------
                       Pp3c2_10440V1.1 (273) -YQIVLFVASILLLITGFIAGFIALYNNVDINEICRWCRRINCVPSPRWSCNSTSG------------
     AT1G77860 RHOMBOID-like protein 8 (288) -DRPVLRIICLLVFCGILAGVLLAACWGVNLNRHCHWCRYVDCVPTKKWSCSDMTTSCEVYSSKP---
    AT1G25290 RHOMBOID-like protein 10 (340) -QRRL---------------------------------------------------------------
                       Pp3c1_43050V1.1 (387) -EKSDQ--------------------------------------------------------------
     AT5G25752 rhomboid-like protein 11 (281) --------------------------------------------------------------------
    AT1G18600 RHOMBOID-like protein 12 (337) --------------------------------------------------------------------
     AT1G74130 rhomboid-like protein 16 (323) --------------------------------------------------------------------
     AT1G74140 rhomboid-like protein 17 (321) --------------------------------------------------------------------
  AT3G07950 rhomboid protein-like protein (277) ALEERLGTERLVPARNKDELQSDGLDNV----------------------------------------
  AT2G41160 ubiquitin-associated protein (247) -EEAIATLVSMGFDQNAARQALVHARNDVNAATNILLEAHSH--------------------------
AT3G56740 ubiquitin-associated (UBA) protein (253) -EEAITTLVSMGFDRNAARQALVHARNDVNAATNILLEAQSH--------------------------
     AT3G17611 rhomboid-like protein 14 (335) --------------------------------------------------------------------
     AT3G58460 rhomboid-like protein 15 (386) SEEQIQKLVAMGFDRTQVEVALAAADDDLTVAVEILMSQQA---------------------------
    AT3G59520 RHOMBOID-like protein 13 (270) --------------------------------------------------------------------
     AT5G38510 RHOMBOID-like protein 9 (391) -NSADPCKSFLLFTIFVAVIVTSLLLIGDGPLDFPTYDDVVYSLI-----------------------
                  Pp1s254_10V6.2 HIP9 (534) -LNEGIDPYRLLLVFALAVAVFSALFS--VGVPYATEYHFLHGGLGDEYW------------------
                       Pp3c22_8560V1.1 (533) -LNEGIHPYRLLLVFGLSVAVFFALYS--VGVPYATEFHFLHGGLGDEYW------------------
                             Consensus (613)     I     LL            L               L
```

Peptidase S54, rhomboid superfamily

		681 ... 690 ... 700 ... 711
	(681)	
AT1G12750 RHOMBOID-like protein 6	(308)	-------------------------------
AT1G63120 rhomboid -like 2	(318)	-------------------------------
AT5G07250 RHOMBOID-like protein 3	(347)	-------------------------------
AT2G29050 protein RHOMBOID-like 1	(359)	LTCITNGKTEMYKLSNDIPSRIQQLCSQLCR
AT4G23070 RHOMBOID-like protein 7	(314)	-------------------------------
AT3G53780 RHOMBOID-like protein 4	(364)	MTCLRNGKSASYILANPSDSRINSLCVQLCR
AT1G52580 RHOMBOID-like protein 5	(310)	-------------------------------
Pp3c17_14100V1.1	(323)	-------------------------------
Pp3c2_10440V1.1	(328)	-------------------------------
AT1G77860 RHOMBOID-like protein 8	(352)	-------------------------------
AT1G25290 RHOMBOID-like protein 10	(344)	-------------------------------
Pp3c1_43050V1.1	(392)	-------------------------------
AT5G25752 rhomboid-like protein 11	(281)	-------------------------------
AT1G18600 RHOMBOID-like protein 12	(337)	-------------------------------
AT1G74130 rhomboid-like protein 16	(323)	-------------------------------
AT1G74140 rhomboid-like protein 17	(321)	-------------------------------
AT3G07950 rhomboid protein-like protein	(305)	-------------------------------
AT2G41160 ubiquitin-associated protein	(288)	-------------------------------
AT3G56740 ubiquitin-associated (UBA) protein	(294)	-------------------------------
AT3G17611 rhomboid-like protein 14	(335)	-------------------------------
AT3G58460 rhomboid-like protein 15	(427)	-------------------------------
AT3G59520 RHOMBOID-like protein 13	(270)	-------------------------------
AT5G38510 RHOMBOID-like protein 9	(435)	-------------------------------
Pp1s254_10V6.2 HIP9	(581)	-------------------------------
Pp3c22_8560V1.1	(580)	-------------------------------
Consensus	(681)	
