# Supplementary material for: Holophytochrome-Interacting Proteins in Physcomitrella: Putative Actors in Phytochrome Cytoplasmic Signaling
Source: Front Plant Sci. 2016 May 12;7:613. doi: 10.3389/fpls.2016.00613 (PMC4867686; doi:10.3389/fpls.2016.00613)

frontiers

Supplementary Material

Holophytochrome-interacting proteins in *Physcomitrella*: putative actors in phytochrome cytoplasmic signaling

Anna Lena Ermert, Katharina Mailliet, and Jon Hughes*

phy4 (Pp3c27_7830V1.1)

```
ATGTCGACCACCAAGTTGGCATACTCGTCAGGGAGTTCTGTGAAGTCGAAGCATAGTGTACGGGTTGCGCAAACTACAGCA
GATGCCAAGCTGCAGGCAGTGTATGAAGAATCCGGAGATTCGGGAGACTCATTTGATTATTCCAAATCAGTTCATGCGTCC
AAATCCACTGGCGAGAATGTCCCTGCCCTAGCAGTCACCGCGTACCTTCAGCGCATGCAAAGAGGGGGTTTGGTGCAGACA
TTCGGGTGTATGCTATGTGTGGACGAGAGTAGTTTTAGGGTTATTGCTTACAGCGAGAATGCGCCAGAGATGCTTGACCTG
ATGCCACAAGCGGTGCCCAGTGTGGGTCAGCAGGAAGTTCTGGGCATTGGAACCGATGCAAGAACTTTGTTCACCCCTTCC
AGTGCGGCCGCCCTGGAGAAATGTGCCGGAGCGGTGGATGTGACCATGCTGAACCCTATCTCAGTGCACTGTCGAAGCTCG
GGGAAGCCGTTCTATGCGATTCTGCACCGTATTGACGTAGGACTGGTAATTGATTTTGAACCAGTGCGCCCGAATGATGCC
GTTGTGTCCTCCGCAGGGGCTTTGCAGTCGCATAAGTTGGCTGCAAAAGCGATATCTCGGCTGCAGGCACTTCCTGGCGGA
GACATTGGCCTCCTTTGTGATACAGTTGTAGAAGAGGTACGCCAGCTTTCCGGTTACGATCGTGTCATGGCATACAAATTC
CACGAAGACGAACATGGTGAGGTCTTGGCGGAGATTCGAAGATCAGATTTAGAACCTTACTTAGGTCTACATTACCCAGCA
ACAGATATCCCACAGGCGTCAAGATTCCTGTTTATGAAGAACAGGGTGCGAATGATAGGTGATTGCTATGCTCCTCCTGTT
AAAGTGGTTCAGGACAAGGATTTGAGGCAGCCTATTAGTCTGGCCGGGTCTACTTTACGGGCCCCTCATGGCTGCCATGCG
CAATACATGGGCAACATGAACTCCATCGCTTCGTTGGTGATGGCAGTTATCGTGAATGATCCGGATGAGGATCCTAATTCA
CGTGGGGGCCAGCAGAGAGGGCGCAAGCTCTGGGGATTGGTGGTTTGTCACCACACGTCTCCGAGGACCGTCTCCTTTCCT
TTGAGATCGGCCTGTGAGTTCCTGATGCAGGTGTTCGGTCTGCAGCTGAACATGGAAGTTGAACTTGCAGCTCAGCTAAGA
GAAAAACATATTTTGAGAACACAAACCCTTCTCTGTGACATGTTATTGCGGGATGCACCCATCGGAATTGTTTCTCAGTCA
CCCAATATCATGGACTTGGTTAAATGCGACGGTGCAGCACTTTACTATGGGAAAAGGTTTTGGCTTTTGGGAATCACACCT
AACGAGGTCCAGATTAAGGAAATCGCGGACTGGCTCCTAGAACATCATCAAGATTCGACCGGTTTGAGTACAGACAGCCTG
GCCGATGCTGGTTACCCTGGCGCAGCGCAACTTGGTGATGCTGTGTGTGGAATGGCTGCGGCAAAGATCACTCCAAGGGAC
TTTCTTTTCTGGTTCAGGTCACACACCGCCAAGGAGATTAAGTGGGGTGGTGCCAAGCACGATCCTGATGAAAAGGATGAT
GGGAGGAAAATGCATCCCCGCTCCTCGTTCAAGGCCTTTCTAGAAGTTGTGAAGAGAAGAAGTTTGCCATGGGAGGACATA
GAAATGGACGCAATTCATTCTCTTCAACTCATTTTGAGAGGTTCATTCCAGGATATAGACGACAGTGATACCAAAACTATG
ATACACGCCCGACTCAACGACTTAAAGCTCCAGGGCATGGATGAACTCAGTACAGTTGCTAATGAAATGGTTAGATTGATA
GAGACAGCGACTGCACCAATTCTCGCTGTCGATTCGAGTGGATTCATCAATGGTTGGAATGCCAAAGTAGCTGAACTGACT
GGACTTCCAGTTGGAGAAGCCATGGGTCGGTCGCTGGTTAAAGATTTAATTTTAGAGGAATCAATTGATGTTGTTCAGCGA
CTTCTCTACCTTGCATTACAAGGTGAAGAAGAACAAAATATTGAGATTCAGTTGAAGACCTTTGGGCCACAAAAAGAGAAG
GGCGCAGTGATTTTGATAGTGAATGCGTGCTCCAGCAGGGATGTACAAGACAATGTCGTTGGAGTTTGTTTTGTGGGGCAA
GATGTGACAGGTCAAAAACAAGTTTTGGATAAATTTACTCGGATACAAGGCGACTACAAAGCAATAGTTCAGAACCCAAAC
CCTTTGATTCCTCCTATATTTGGCACTGATGAATATGGATATTGCTCTGAGTGGAATCCTTCAATGGAGAAGCTGACTGGA
TGGAAGAGAGAAGAGGTGCTTGGGAAACTGCTAGTGGGTGAAATTTTTGGAATGCAACTTATGTGTTGCCGACTGAAAGGT
CAAGATGCAATGACGAAATTTATGATTGCATTAAATAGTGCAATGGATGGTCAAGATACAGATCGGTTTCCCTTTTCCTTC
TTTGATCGACAAGGGAAATACGTAGATGCCCTACTCACAGTCAATAAAAGAACGGATGCAGAGGGAAGCATCACTGGCGTA
TTCTGCTTTTTGCACACAACCAGTGTGGAATTATTGCAAGCATTGACTGTCCAACGTGCGACGGAGAAAGTAGCCTTCGCA
AAACTCAAGGAGTTGGCTTACATTCGACAGGAGATCAAGAATCCTTTGTATGGGATTATGTTTACACGTAATTTAATGGAA
GATACGGACTTATCTGAGGACCAAAGGCAGTTTGTGGAGACTAGTGCTGTGTGTGAACGTCAATTGCGCAAAGTATTGGAT
GATATGGATCTTGAGAGTATTGAGGACGGGTACTTAGAGCTGGATACAAATGAATTTGTGATGGGAACAGTGATGGATGCC
GTTGTAAGTCAAGGAATGATCACATCAAGAGAAAAAGGTCTGCAATTGATACGGGAGACTCCCAGGGAGATAAAGAACATG
TGTTTATTTGGGGACCAAGTCCGTTTACAACAAGTGCTCGCGGACTTTTTGTTGAATGCAGTGAGATTCACCCCATCCTCT
GAAGGCTGGGTCGGGATCAAGGTGGTCCCAACGAAGAAGCGCCTGGGTGGAGGCATTCATGTCATGCATCTGGAATTCAGG
GTTACACATTCAGGAATGGGACTTCCTGAGGAGCTTGTGCATGAGATGTTTGATCGAGGACGAGGCATGACTCAAGAAGGC
CTGGGGCTGAGTATGTGTCGTAAACTTGTAAAGCTAATGAATGGTAATGTTCAATATATTAGAGAAACAGGTAAGAGCTAC
TTTTTAGTTGAAGTGGAGCTTCCTTTGGCACAACGAGATGATGCAGGCAGTGTGAGATAG
```

Expression data

eFP

Expression data for various developmental stages were retrieved from the Physcomitrella electronic Fluorescent Pictograph (eFP) browser (http://bar.utoronto.ca/efp_physcomitrella/cgi-bin/efpWeb.cgi) on the free Bio-analytic Resource for Plant Biology (BAR) server; see Ortiz-Ramírez et al. (2016)). The eFP-browser provides microarray data of most developmental stages of the *Physcomitrella* life cycle including protonema (choro- and dark-grown caulonema), rhizoids, gametophores, archegonia, 4 stages of sporophyte development (based on morphological characteristics such as size, shape and degree of maturation: sporophyte S1 5-6 days after fertilization (dAF); S2 9-11 dAF, S3 18-20 dAF; S4 28-33 dAF) and spores. Raw data were normalized using robust multi-array average (RMA).

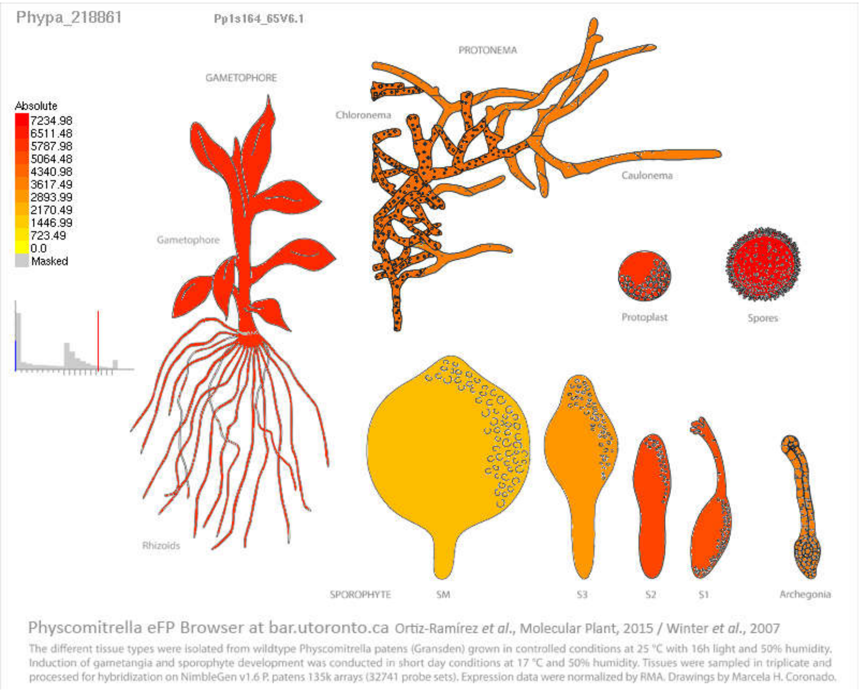

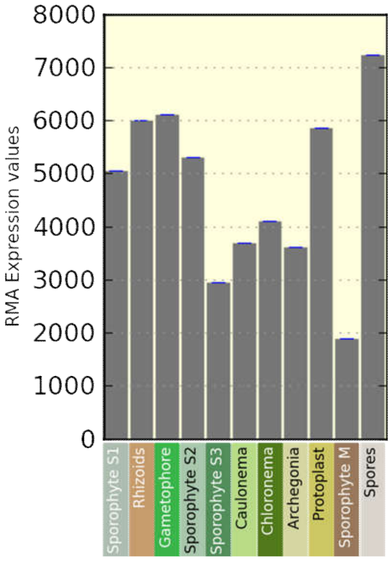

JGI Phytozome 11 Gene Atlas Gene-level expression

Expression data for different culture conditions and developmental stages were retrieved from JGI´s Phytozome, which hosts RNAseq data aligned to the *Physcomitrella patens* reference genome. The *Physcomitrella* dataset includes experiments with gametophores on different media, (de/re)-hydration & treatment with NAA, detached leaflets, protonemata varied by medium and different (light-) treatments, protoplasts from protonemata, spores and green and brown sporophytes (see https://phytozome.jgi.doe.gov/phytomine/aspect.do?name=Expression). Normalized data are given as FPKM (fragments per kilobase of exon per million reads mapped) values.

FPKM Coefficient of variance: 0.296 (across samples below)

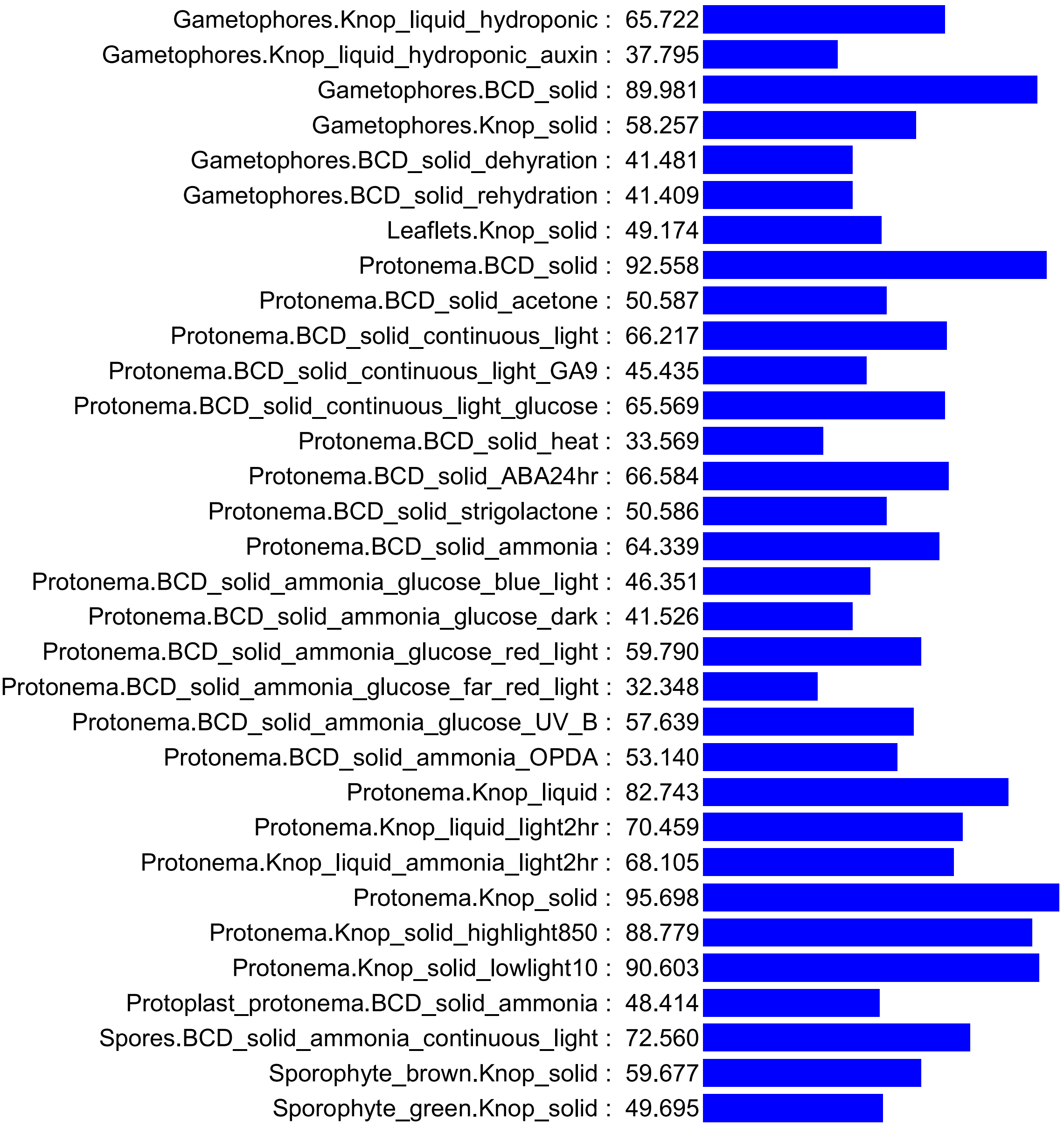

Genevestigator (Phypa_218861)

Genevestigator (www.genevestigator.com) includes *Physcomitrella* microarray expression data of 74 samples from 6 studies including anatomical structures (protonemata (protoplasts), gametophores phyllids and spores), different conditions (biotic and abiotic stress, different light intensisties/qualities, photoperiod, dedifferentiation vs. untreated phyllids & comparison of genetic backgrounds) and different developmental stages. Data are shown here in log2 scale.

Anatomy

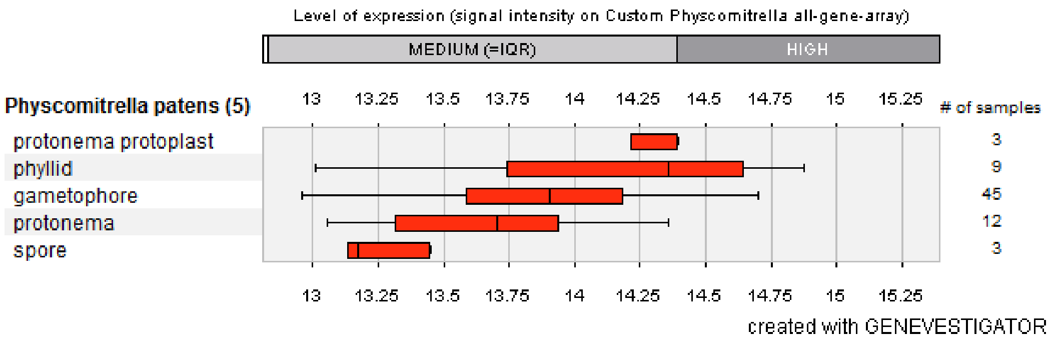

Conditions

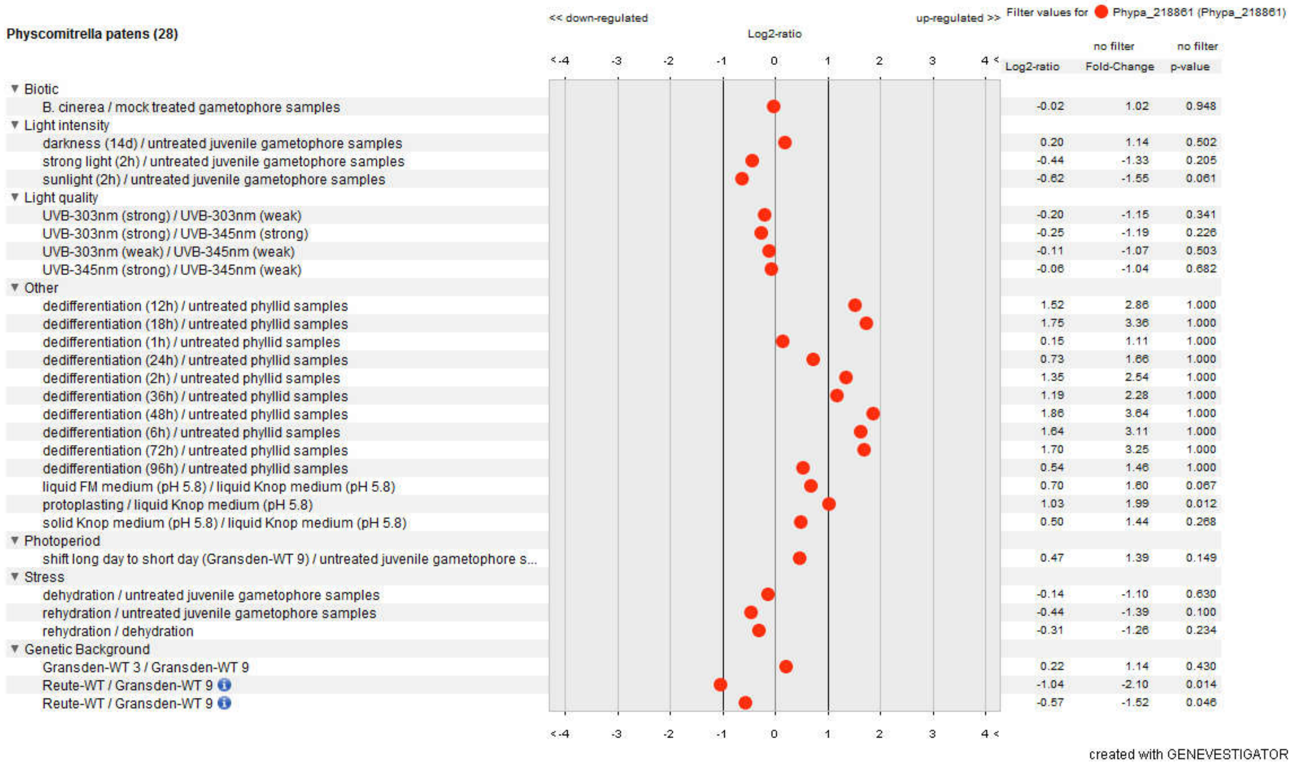

	no filter Log2-ratio	no filter Fold-Change	p-value
B. cinerea / mock treated gametophore samples	-0.02	1.02	0.948
darkness (14d) / untreated juvenile gametophore samples	0.20	1.14	0.502
strong light (2h) / untreated juvenile gametophore samples	-0.44	-1.33	0.205
sunlight (2h) / untreated juvenile gametophore samples	-0.62	-1.55	0.061
UVB-303nm (strong) / UVB-303nm (weak)	-0.20	-1.15	0.341
UVB-303nm (strong) / UVB-345nm (strong)	-0.25	-1.19	0.226
UVB-303nm (weak) / UVB-345nm (weak)	-0.11	-1.07	0.503
UVB-345nm (strong) / UVB-345nm (weak)	-0.06	-1.04	0.682
dedifferentiation (12h) / untreated phyllid samples	1.52	2.86	1.000
dedifferentiation (18h) / untreated phyllid samples	1.75	3.36	1.000
dedifferentiation (1h) / untreated phyllid samples	0.15	1.11	1.000
dedifferentiation (24h) / untreated phyllid samples	0.73	1.66	1.000
dedifferentiation (2h) / untreated phyllid samples	1.35	2.54	1.000
dedifferentiation (36h) / untreated phyllid samples	1.19	2.28	1.000
dedifferentiation (48h) / untreated phyllid samples	1.86	3.64	1.000
dedifferentiation (6h) / untreated phyllid samples	1.64	3.11	1.000
dedifferentiation (72h) / untreated phyllid samples	1.70	3.25	1.000
dedifferentiation (96h) / untreated phyllid samples	0.54	1.46	1.000
liquid FM medium (pH 5.8) / liquid Knop medium (pH 5.8)	0.70	1.60	0.067
protoplasting / liquid Knop medium (pH 5.8)	1.03	1.99	0.012
solid Knop medium (pH 5.8) / liquid Knop medium (pH 5.8)	0.50	1.44	0.268
shift long day to short day (Gransden-WT 9) / untreated juvenile gametophore s...	0.47	1.39	0.149
dehydration / untreated juvenile gametophore samples	-0.14	-1.10	0.630
rehydration / untreated juvenile gametophore samples	-0.44	-1.39	0.100
rehydration / dehydration	-0.31	-1.26	0.234
Gransden-WT 3 / Gransden-WT 9	0.22	1.14	0.430
Reute-WT / Gransden-WT 9	-1.04	-2.10	0.014
Reute-WT / Gransden-WT 9	-0.57	-1.52	0.046

Development

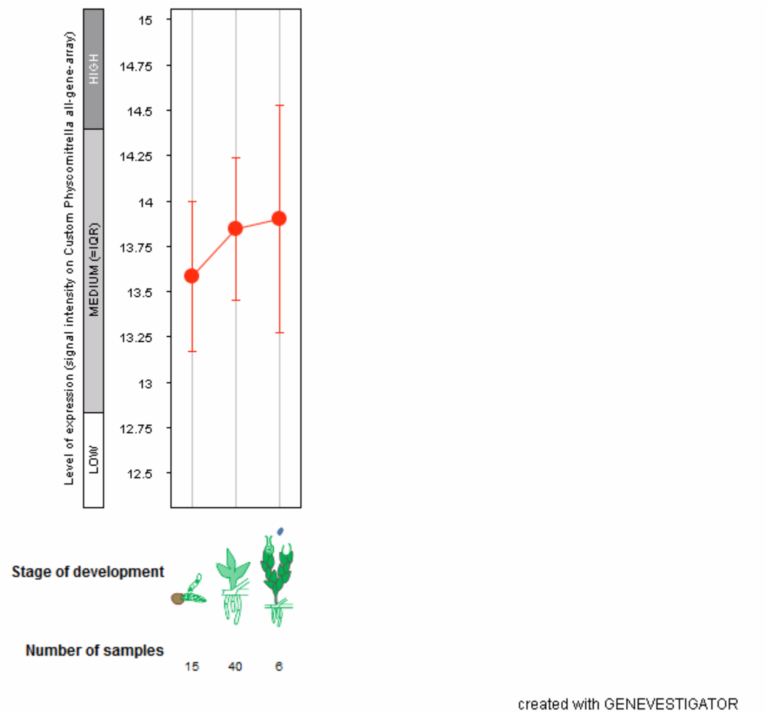

Supplement: Supplementary file 2 [file Data_Sheet_2.ZIP › SI/SI phy4.pdf]
